# New Hydrogenated Phenanthrene Glycosides from the Edible Vegetable *Elatostema tenuicaudatum* W.T.Wang with DPP-IV Inhibitory and Hepatoprotective Activity

**DOI:** 10.1021/acs.jafc.4c08713

**Published:** 2025-01-06

**Authors:** Quoc-Dung Tran Huynh, Su-Jung Hsu, Truc-Ly Thi Duong, Hui-Kang Liu, Ta-Wei Liu, Man-Hsiu Chu, Yun-Han Wang, Dang-Khoa Nguyen, Thuy-Tien Thi Phan, Nguyen-Khanh Huynh Tran, Thanh-Hoa Vo, Hsiao-Yang Hsi, Tz-Wei Yeh, Ching-Kuo Lee

**Affiliations:** † Ph.D. Program in Clinical Drug Development of Herbal Medicine, College of Pharmacy, Taipei Medical University, Taipei 11031, Taiwan; ‡ Institute of Pharmaceutical Education and Research, Binh Duong University, Thu Dau Mot 820000, Binh Duong, Vietnam; § School of Pharmacy, College of Pharmacy, Taipei Medical University, Taipei 11042, Taiwan; ∥ Faculty of Traditional medicine, Can Tho University of Medicine and Pharmacy, Can Tho 900000, Vietnam; ⊥ National Research Institute of Chinese Medicine, Ministry of Health and Welfare, Taipei 11221, Taiwan; # Faculty of Pharmacy, Ton Duc Thang University, Ho Chi Minh 700000, Vietnam; ∇ Graduate Institute of Biomedical Materials and Tissue Engineering, College of Biomedical Engineering, Taipei Medical University, Taipei 11031, Taiwan; ○ University of Health Sciences, Vietnam National University Ho Chi Minh City, Ho Chi Minh 700000, Vietnam; ◆ Center for Discovery and Development of Healthcare Product, Vietnam National University Ho Chi Minh City, Ho Chi Minh 700000, Vietnam; ¶ Institute of Fisheries Science, National Taiwan University, Taipei 106, Taiwan; ⋈ Graduate Institute of Pharmacognosy, College of Pharmacy, Taipei Medical University, Taipei 11042, Taiwan; ⧓ Department of Chemistry, Chung Yuan Christian University, Zhongli District, Taoyuan 32023, Taiwan

**Keywords:** elatostema tenuicaudatum, molecular networking, DPP-IV inhibition, HepG2, hepatoprotection

## Abstract

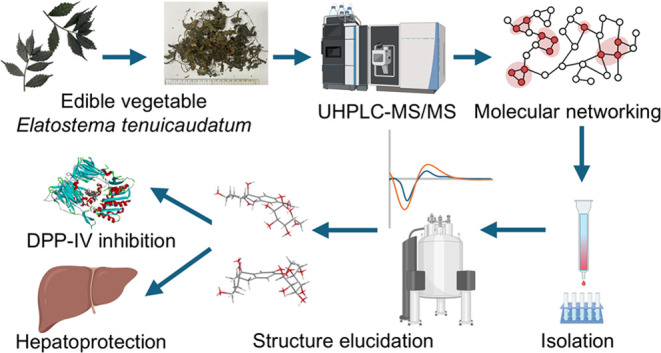

Based on molecular networking-guided isolation, 15 previously undescribed hydrogenated phenanthrene glycosides, including eight hexahydro-phenanthrenone glycosides, four tetrahydro-phenanthrenone glycosides, one dihydro-phenanthrenol glycoside, two dimers, and two known dihydrophenanthrene glycosides, were isolated from *Elatostema tenuicaudatum* W.T.Wang, a popular regional edible vegetable at the northwest region of Vietnam. Their chemical structures were determined using extensive spectroscopic data: NMR and ECD calculations. Notably, the crude extract, along with compounds **5**, **6**, **8**, and **14**, demonstrated dipeptidyl peptidase IV inhibitory activity with IC_50_ values of 220.5 ± 39.6 μg/mL, 141.7 ± 15.6, 151.2 ± 11.8, 107.9 ± 19.6, and 71.9 ± 8.9 μM, respectively. Molecular docking indicates compound **14** possesses the highest binding affinity with DPP-IV. Besides, compounds **1**, **9**, **11**, and **14** exhibited significant hepatoprotective effects in acetaminophen-induced hepatotoxicity in HepG2. These findings suggested that *E. tenuicaudatum* can serve as a beneficial vegetable for individuals at risk of diabetes and chronic liver disease.

## 1 Introduction

Diabetes mellitus (DM) is a chronic metabolic disorder that is often accompanied by liver damage and inflammation. A growing body of research highlights a strong connection between diabetes and chronic liver diseases, such as nonalcoholic fatty liver disease (NAFLD), alcoholic liver cirrhosis, and liver cancer.^1^ Furthermore, diabetes is recognized as a leading cause of end-stage liver cirrhosis.^2^

An effective strategy for managing type 2 diabetes (T2D) is targeting the enzyme dipeptidyl peptidase IV (DPP-IV), also known as CD26. DPP-IV is a serine protease that modulates peptide activity by cleaving proline or alanine from the N-terminal. This enzyme inactivates incretins such as glucagon-like peptide-1 (GLP-1) and glucose-dependent insulinotropic polypeptide (GIP), which play vital roles in regulating blood glucose. GLP-1 in particular stimulates insulin production by pancreatic β-cells, inhibits glucagon release, reduces glycogen synthesis, and slows gastric emptying, thereby delaying glucose absorption. However, research has shown that GLP-1 levels are lower in T2D patients than in healthy individuals and are rapidly degraded by DPP-IV. Hence, inhibition of DPP-IV activity is a crucial strategy for controlling blood glucose levels.^3^

DPP-IV is also significantly involved in the progression of chronic liver diseases, suggesting that DPP-IV inhibitors can enhance liver health and function.^4,5^ In recent years, DPP-IV inhibitors such as sitagliptin have been approved for use by the US FDA as monotherapy or as part of combination therapy for T2D; however, the potential side effects may include diarrhea, nausea, vomiting, abdominal pain, headache, and upper respiratory tract infections.^6^ Consequently, the search for natural compounds with fewer side effects as alternative treatments has become an urgent and intriguing research area.

Acetaminophen (APAP) is a widely used medication that is recognized for its pain-relieving and fever-reducing properties. However, its excessive consumption can cause liver damage, leading to hepatotoxicity and acute liver failure.^7^ The HepG2 hepatoma cell line is extensively employed in research focusing on liver function, metabolism, and the effects of drugs, owing to its biochemical and morphological traits with typical hepatocytes; this makes HepG2 cells highly valuable for studying the hepatoprotective properties.^8−12^ Therefore, APAP-treated HepG2 cells are often used as models of hepatoprotective activity assessment.^13^ Numerous studies have utilized these models to assess the liver-protective and antidiabetic properties of various foods, particularly vegetables.^14−17^

The genus *Elatostema*, belonging to the family Urticaceae, contains a variety of phytochemicals, such as sterols, phenolics, terpenoids, and alkaloids, which exhibit valuable bioactivities, such as antioxidant, anti-inflammatory, and anti-Alzheimer’s effects.^18−21^ In northwest Vietnamese cuisine, the aerial parts of *Elatostema tenuicaudatum*, also known as Com khia, Com kia, La dang, or Mat vit in Vietnamese, which means bitter vegetable, are popular ingredients in stir-fried vegetables and soups.^21^ Because of its characteristic bitter taste, this vegetable is typically prepared with fatty and protein-rich ingredients to balance its flavor. Traditionally, it is considered beneficial for digestion, diabetes management, and liver detoxification. Many studies have shown that the bitter taste of many plants is associated with their phytochemical components, such as alkaloids, terpenes, phenolics, and glucosinolates.^22−25^ Some compounds have demonstrated significant bioactive effects, including the antihyperglycemic properties of bitter melon (*Momordica charantia* L.),^26^ the antimalarial effects of quinine extracted from *Cinchona officinalis*,^27^ and the antihyperglycemic and anti-inflammatory properties of *Andrographis paniculata*.^28−31^ Nevertheless, some bitter compounds have demonstrated high toxicity such as strychnine from *Strychnos nux-vomica* or certain glycoalkaloid compounds found in potatoes.^32,33^ Thus, vegetables with a bitter taste, such as *E. tenuicaudatum*, can be considered rich sources of phytochemicals and need to be studied carefully in terms of their chemical and biological properties to ensure their safety and effectiveness for utilization. In our search for vegetables that are suitable for individuals with diabetes or chronic liver disease, we identified *E. tenuicaudatum*, an edible bitter vegetable, as a potential candidate. However, there is currently a lack of scientific studies specifically examining the chemical components or bioactivities of *E. tenuicaudatum*. Consequently, the initial crucial step in exploring its potential is to identify its chemical constituents and evaluate the bioactivity of its phytochemicals in the management of diabetes and their hepatoprotective effects.

In this study, we examined the phytochemicals and bioactivity of *E. tenuicaudatum* by focusing on DPP-IV inhibition and hepatoprotective activity. Using molecular networking-guided chromatography techniques for isolation as well as extensive spectroscopic data and ECD calculations for structure identification, we isolated two known dihydrophenanthrene glycosides and 15 previously undescribed compounds. Interestingly, these compounds belong to the glycosidic hydrogenated phenanthrene family and are thus rarely found in plants. Among these, four compounds demonstrated mild-to-moderate DPP-IV inhibitory effects. The binding mechanisms of these compounds to DPP-IV were further analyzed using molecular docking models. In addition, the four compounds exhibited hepatoprotective activity in *in vitro* tests. Based on these findings, we propose that *E. tenuicaudatum* may be used in meals to support individuals with diabetes or chronic liver disease.

## 2 Materials and Methods

We have described the detailed information for general experimental procedures (3.1), materials (3.2), UPLC MS/MS and molecular network (3.3), extraction and isolation (3.4), ECD calculation (3.5), determination of sugar by hydrolysis (3.6), DPP-IV inhibition activity (3.7), molecular docking (3.8), and hepatoprotective effects against acetaminophen-induced hepatotoxicity in the HepG2 cell model (3.9) in the relevant sections in the Supporting Information.

## 3 Results and Discussions

### 3.1 UHPLC-MS/MS and Molecular Networking-Guided Isolation

**Figure 1:**
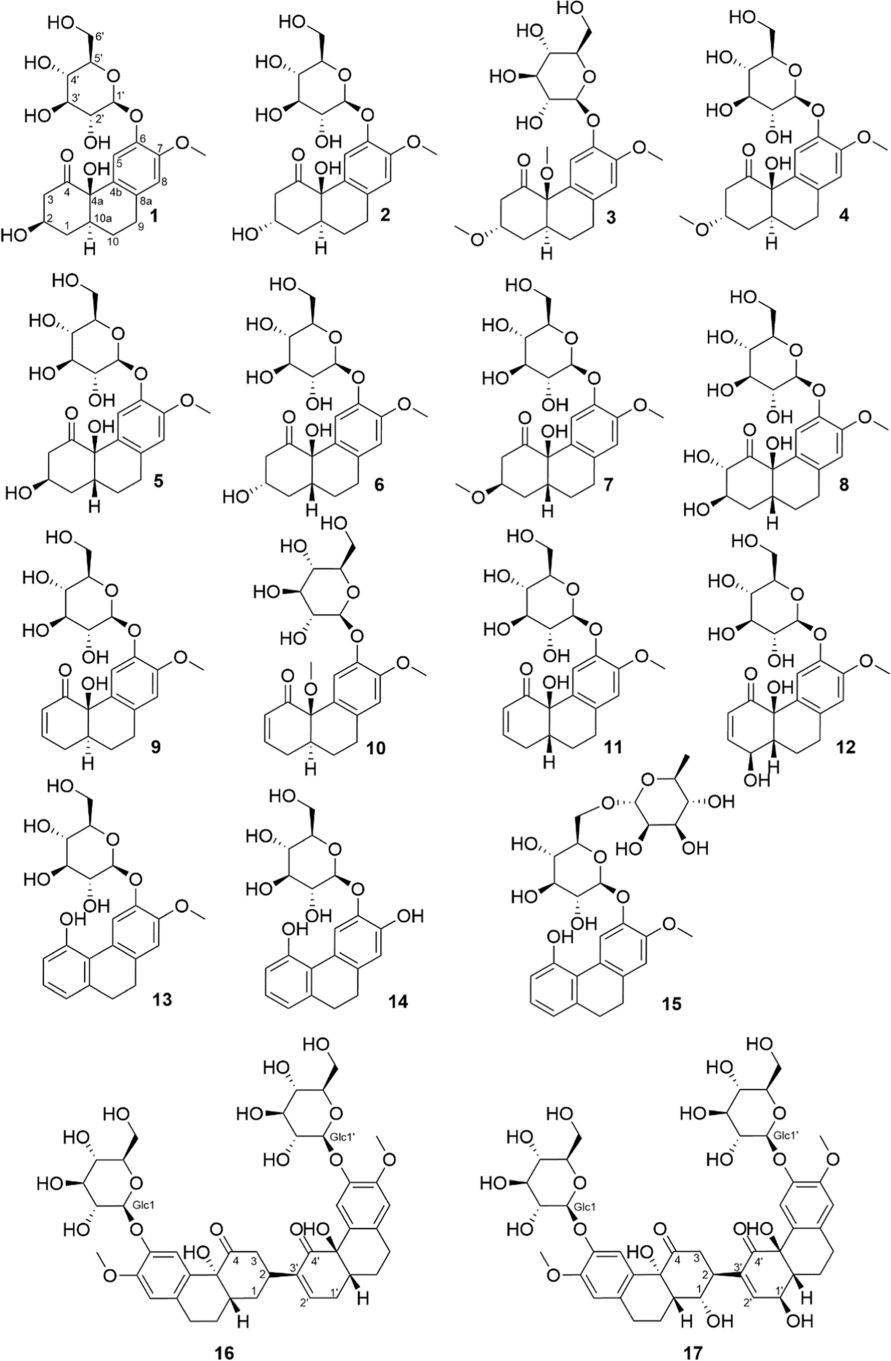
Chemical structures of compounds **1–17**.

In the present study, molecular network analysis was conducted to guide the isolation of target compounds,^34−36^ with the prioritization of nodes based on three clear criteria. First, emphasis was placed on the major peaks and large clusters because they represented the primary components of the sample; owing to the limited research on this vegetable, prioritizing the study of these major components was essential in our investigation. Second, we focused on secondary metabolites, as these compounds are more likely to exhibit bioactivities of interest, making them the primary focus of this study. Third, the novelty of the nodes was a critical factor. Nodes not identified in the GNPS database were given special attention because the lack of matching strongly suggests that these nodes may represent previously undescribed compounds. This criterion increased the likelihood of identifying novel bioactive secondary metabolites (Figure 1).

**Figure 2:**
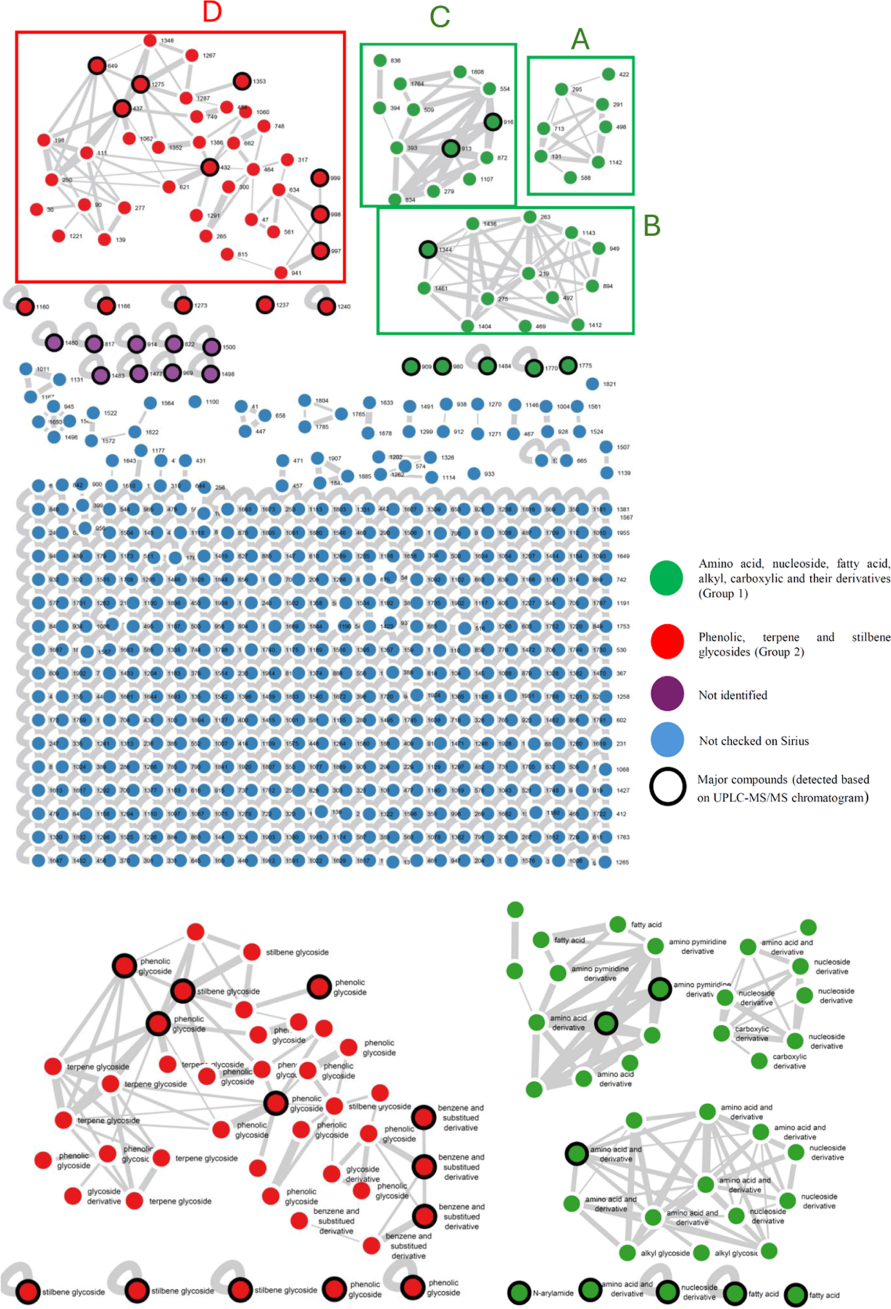
Molecular networking analysis by GNPS and chemical group classification of clusters and nodes by Sirius of *E. tenuicaudatum* extract.

**Figure 3:**
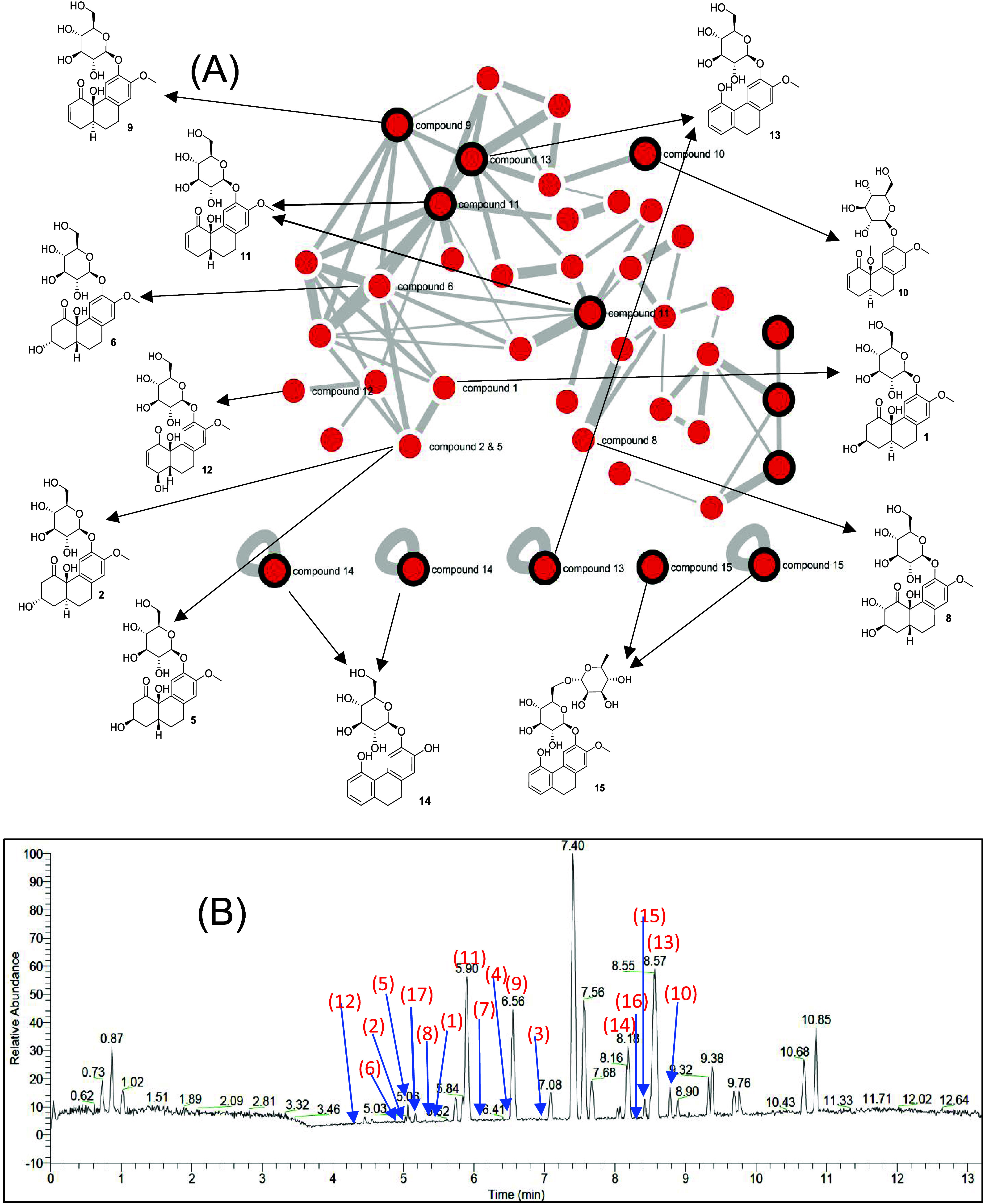
Mapping results of isolated compounds into molecular network (A) and UPLC-MS/MS chromatogram (B).

In this study, the sample extract underwent UHPLC-MS/MS analysis and was then subjected to Global Natural Products Social Molecular Networking (GNPS) to create a molecular network. The results revealed four main clusters, with Cluster D being the largest (Figure 2). Subsequently, each node within these clusters and nodes (related to major peaks in the UHPLC chromatogram) was analyzed using the ClassyFire algorithm in Sirius for automated chemical class identification. Clusters A, B, and C primarily consisted of nodes associated with the “amino acid and derivative” group (nodes 279, 293, 295, 1344, 1461, 275, 219, 263, and 1143) and the “nucleoside derivative” group (nodes 291, 496, 713, 1142, and 894). A smaller number of nodes within these clusters were also related to the “amino pymiridine derivative” group (nodes 393, 509, 1808), the “fatty acid” group (nodes 1176, 1808), the “alkyl glycoside” group (nodes 1404, 469), and the “carboxylic derivative” group (nodes 568, 1142). Because these chemical groups are commonly associated with primary metabolites, Clusters A–C were collectively grouped into Group 1 (represented by green nodes) for simplification. Besides, based on the UHPLC chromatogram, some major nodes—node 980 (retention time 7.56 min—“amino acid and derivative”), node 909 (retention time 7.40 min—“N-arylamide”), node 1484 (retention time 9.32 min—“nucleoside derivative”), and nodes 1770 and 1775 (retention time 10.68 min—“fatty acid”)—were also placed into Group 1 (Figures 2 and 3). Other major nodes at retention of 7.04, 7.08, 9.32, and 9.38 (nodes 1480, 817, 914, 822, 1500, 1483, 1477, 969, 1498) were not identified chemical classes due to low matching of the fragmentation pattern or the simplicity of the MS/MS spectra (Figures 2 and 3). By analyzing the largest cluster, D, most nodes (33 of 38) were identified as glycosidic compounds, including terpene, phenolic, and stilbene glycosides. The remaining five nodes in the same cluster were related to “benzene and substituted derivatives,” which are suggested to be the aglycon forms of glycoside groups. Additionally, some key nodes (based on UHPLC chromatogram) at retention times of 8.18, 8.42, and 8.57 min were determined to belong to the “stilbene glycoside” or “phenolic glycoside” groups (nodes 1160, 1166, 1273, 1237, and 1240) (Figures 2 and 3). These patterns are commonly associated with phytochemicals known for their diverse bioactivities, including antioxidant, antihyperglycemic, anti-inflammatory, and anticancer properties.^37−39^ Consequently, these nodes were grouped into Group 2, as represented by red nodes in Figure 2.

Based on criterion 1, the nodes in Groups 1 and 2 were selected because they represent the major clusters and primary components of the sample. When further analyzed under criterion 2, Group 1, which comprised nodes predominantly associated with primary metabolites such as “amino acid, nucleoside, fatty acid, alkyl, carboxylic, and their derivatives”, was deemed of lesser interest in this study due to its association with primary metabolism. Conversely, Group 2 contained glycosidic compounds predominantly classified as secondary metabolites, which became the primary targets for isolation. Additionally, the absence of matching Group 2 nodes in the GNPS database strongly suggests the presence of previously undescribed glycosidic compounds, further emphasizing their prioritization. Based on these criteria, the nodes within Group 2 were selected as the primary targets for isolation. When mapping the peaks onto the UHPLC chromatogram, the Group 2 nodes were found to cluster within the retention time ranges of 4–7 and 8–9 min. This observation suggested the isolation of fractions and compounds within these specific intervals.

### 3.2 Structural Elucidation

#### 3.2.1 Determination of Sugar Type

Peaks corresponding to d-glucose-naphthylimidazole (NAIM) (retention time = 4.92 min) were observed in the hydrolysate of all compounds. However, the peak corresponding to l-rhamnose-NAIM (retention time of 7.16 min) was detected only in the hydrolysate of compound **15**. These peaks were compared with the reference peaks.

#### 3.2.2 Structural Elucidation of Compounds **1**–**17**

**Figure 4:**
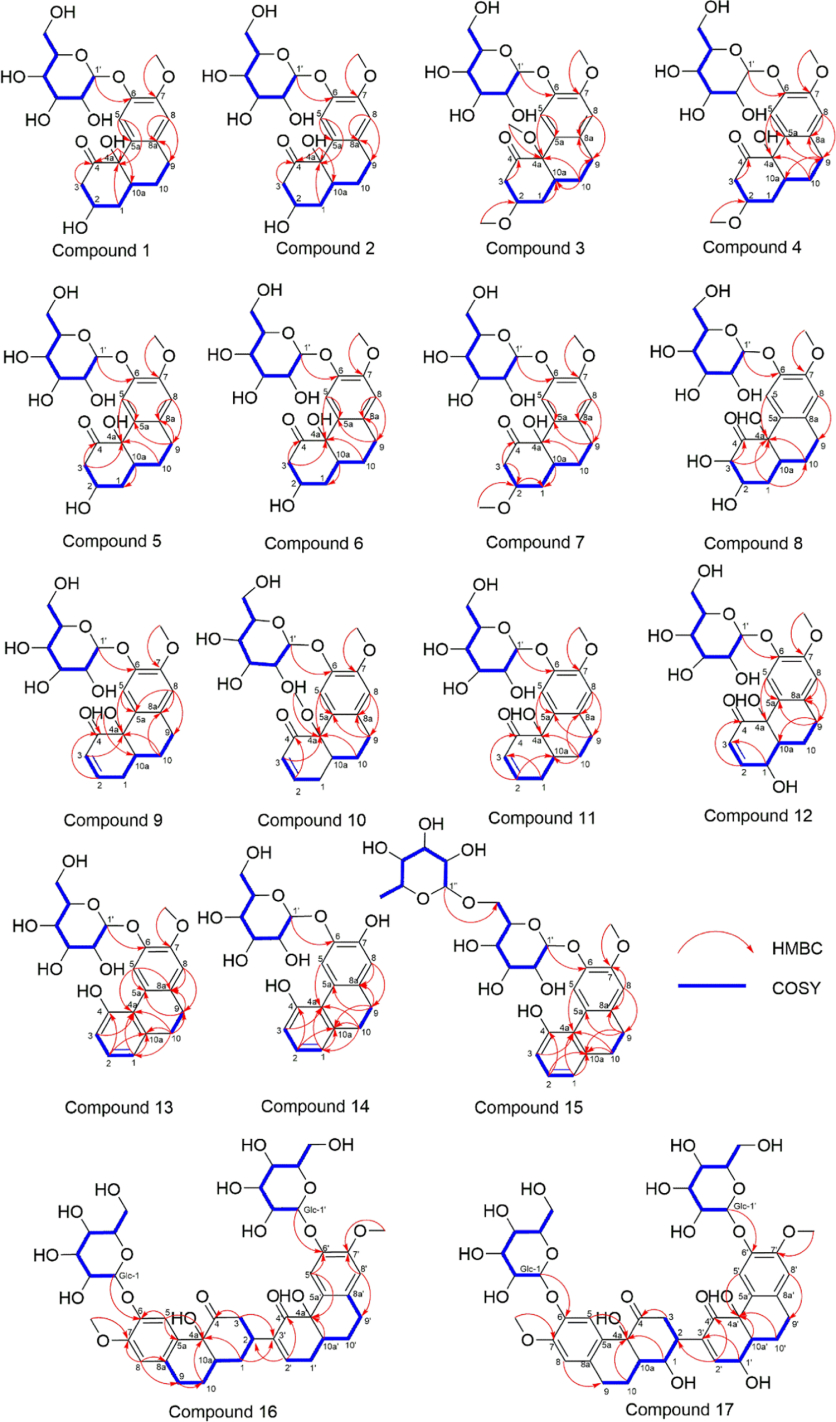
COSY and key HMBC correlation of compounds **1–17**.

Compound **1** was isolated as a white, amorphous powder. Its molecular formula was established as C_21_H_28_O_10_ (degree of unsaturation = 8) from the ion peak [M + Na]^+^ at *m*/*z* 463.15732 (calculated for C_21_H_28_O_10_Na) and [M + HCOO]^−^ at *m*/*z* 485.16531 (calculated for C_22_H_29_O_12_) based on the HR-ESI-MS data. The UV spectrum of **1** showed absorption maxima at 206, 226, and 278 nm. Its IR spectrum exhibited the presence of hydroxy and carbonyl groups (3367 and 1712 cm^–1^, respectively). The ^1^H NMR of compound **1** revealed the signals of two olefinic protons at the *para* position of the benzene ring [δ_H_ 7.82 (1H, s, H-5 and δ_H_ 6.71) (1H, s, H-8)], one methoxy [δ_H_ 3.71 (3H, s, 7-OMe)], and one anomeric proton [δ_H_ 5.46 (1H, d, *J* = 7.6, H-1′)]. The ^13^C NMR and DEPT displayed 21 carbon signals, including one ketone carbon [δ_C_ 210.4 (C-4)], one methoxy signal [δ_C_ 56.3 (7-OMe)], 6 carbons of sugar moieties, and 6 aromatic carbons. Based on the 2D (HSQC, COSY, and HMBC) NMR data, the planar structure of **1** is shown in Figure 4. The HMBC showed a correlation from the H-1′ anomeric proton to C-6 and a correlation between the OMe signal and C-7, confirming the conjugated position of the sugar and methoxy in the *para* tetrasubstituted benzene ring. In addition, the HMBC correlations of H-5/C-4a, C-4b; H-8/C-8a, C-9; 4aOH/C-4a, C-10a, and C-4; and H-3/C-4 confirmed the positions of C-4, C-5, C-8, C-4a, and C-9. The aliphatic connections of H-9/H-10/H-10a/H-1/H-2/H3 and H-1′/H-2′/H-3′/H-4′/H-5′/H-6′ were established based on the COSY spectrum (Figure 4). The anomeric proton at δ_H_ 5.46 (1H, d, *J* = 7.6, H-1′) combined with the coincidence with a d-glucose standard on LC-MS confirmed a β-d-glucose sugar unit in the structure. For the determination of the relative configuration of **1**, on the NOESY spectrum, due to the correlations of H-10_ax_ (δ_H_ 2.29)/H-1_ax_(δ_H_ 2.82); H-9_ax_/H-10a/H-2 and without cross peak between H-9 and H-1, the *trans* orientation of OH-4a and H-10a was confirmed.^40^ In addition, the NOE correlations between H-1′/H-5 and H-8/OMe provided further evidence for the positions of H-5, H-1′, the OMe group, and H-8. Furthermore, the NOESY cross-peaks of H-3_ax_/H-1_ax_/H-10_ax_; H-2_ax_/H10a/H-9_ax_ showed that each correlating group was in the same orientation. The coupling constants of H-3_ax_ and H-3_eq_ at δ_H_ 4.15 (H-3_ax_, dd,10.7, 10.7) and δ_H_ 3.13 (H-3_eq_, dd,10.7, 5.2) indicated the axial orientation of H3_ax_ which was reinforced by the NOESY correlation of H3_ax_/H-1_ax_/H-10_ax_. In addition to the NOESY cross-peak of H-2/H-1_eq_, H-2/H-10a suggested an axial position for H-2 and H-10a (Figure 4). The absolute configuration of **1** was determined to be (2*R*,4a*R*,10a*S*) by comparing its CD spectrum with that of its calculated ECD (Figure 9). This structure was named elatostemanoside A and is shown in Figure 1. The NMR chemical shift data for this compound are presented in Table 1.

**Table 1:** ^1^H NMR and ^13^C NMR Data of Compounds **1**–**4**

	1 (C_5_D_5_N_5_, 500 MHz)		2 (C_5_D_5_N_5_, 600 MHz)		3 (C_5_D_5_N_5_, 600 MHz)		4 (CD_3_OD, 500 MHz)	
position	δ_C_	δ_H_ (*J* in Hz)	δ_C_	δ_H_ (*J* in Hz)	δ_C_	δ_H_ (*J* in Hz)	δ_C_	δ_H_ (*J* in Hz)
1	38.2	2.16, m – α	35.9	2.01, m – α	32.7	1.78, dq (13.2, 2.8) – α	32.0	1.80, dq (13.8, 2.7) – α
		2.82, m – β		2.75, m – β		2.29, td (13.2, 2.7) – β		2.25, ddd (13.8, 12.7, 2.8) – β
2	70.7	4.33, m	69.8	4.82, brs	79.6	3.85, brs	80.0	3.94, dt (5.7, 2.8)
3	50.0	3.13, dd (10.7, 5.2) – α	47.7	2.85, overlap – α	43.6	2.68, overlap – α	43.2	2.53, dt (12.7, 2.8) – α
		4.15, dd (10.7, 10.7) – β		4.03, dd (12.2, 3.8) – β		3.20, overlap – β		3.49, overlap – β
4	210.4		211.0		210.3		211.7	
4a	77.4		78.5		83.2		78.7	
4b	132.7		132.8		134.5		134.1	
5	119.9	7.82, s	120.2	7.88, s	122.7	7.85, s	120.5	7.04, s
6	145.6		145.5		144.2		145.1	
7	150.3		150.2		150.9		150.6	
8	113.0	6.71, s	113.1	6.68, s	113.7	6.74, s	113.1	6.72, s
8a	129.8		130.1		123.1		129.1	
9	30.5	2.77, m – α	30.5	2.72, m – α	30.1	2.70, ovl	30.6	2.75, m
		2.70, m – β		2.67, m – β				
10	25.2	1.65, m – α	25.2	1.59, m – α	25.1	1.47, m – α		1.57, m – α
		2.29, ddd (12.7, 12.6, 5.3) – β		2.31, ddd (12.7, 12.6, 5.4) – β		2.18, qd (12.3, 6.7) – β		
10a	42.0	1.76, tt (12.7, 2.9)	41.6	2.85, overlap	42.3	2.37, tt (12.5, 3.0)	42.3	2.09, tt (12.6, 2.9)
1′	102.5	5.46, d (7.6)	102.6	5.44, d (7.7)	103.7	5.65, d (7.0)	102.7	4.83, d (7.5)
2′	75.3	4.28, m	75.3	4.28, m	75.5	4.37, overlap	74.9	3.47, overlap
3′	78.9	4.19, t (9.0)	78.8	4.19, t (9.0)	79.0	4.38, overlap	77.8	3.42, overlap
4′	71.4	4.39, overlap	71.4	4.36, t (9.0)	71.6	4.44, overlap	71.1	3.43, overlap
5′	78.9	3.77, ddd (9.7, 4.3, 2.5)	78.8	3.77 m	79.1	4.04, ddd (9.5, 4.5, 2.6)	78.1	3.31, overlap
6′	62.7	4.60, dd (11.8, 2.5)	62.7	4.39, dd (11.0, 3.6)	62.8	4.63, dd (11.8, 2.6)	62.4	3.73, dd (12.1, 5.0)
		4.41, overlap		4.61, dd (11.0, 2.5)		4.45, overlap		3.91, dd (12.1, 2.4)
7-OMe	56.3	3.71, s	56.3	3.71, s	56.2	3.73, s	55.5	3.72, s
4a–OH		7.67, s		7.54, s				
4a-OMe					56.1	3.22, s		
2-OMe					53.4	3.22, s	56.0	3.29, s

**Figure 5:**
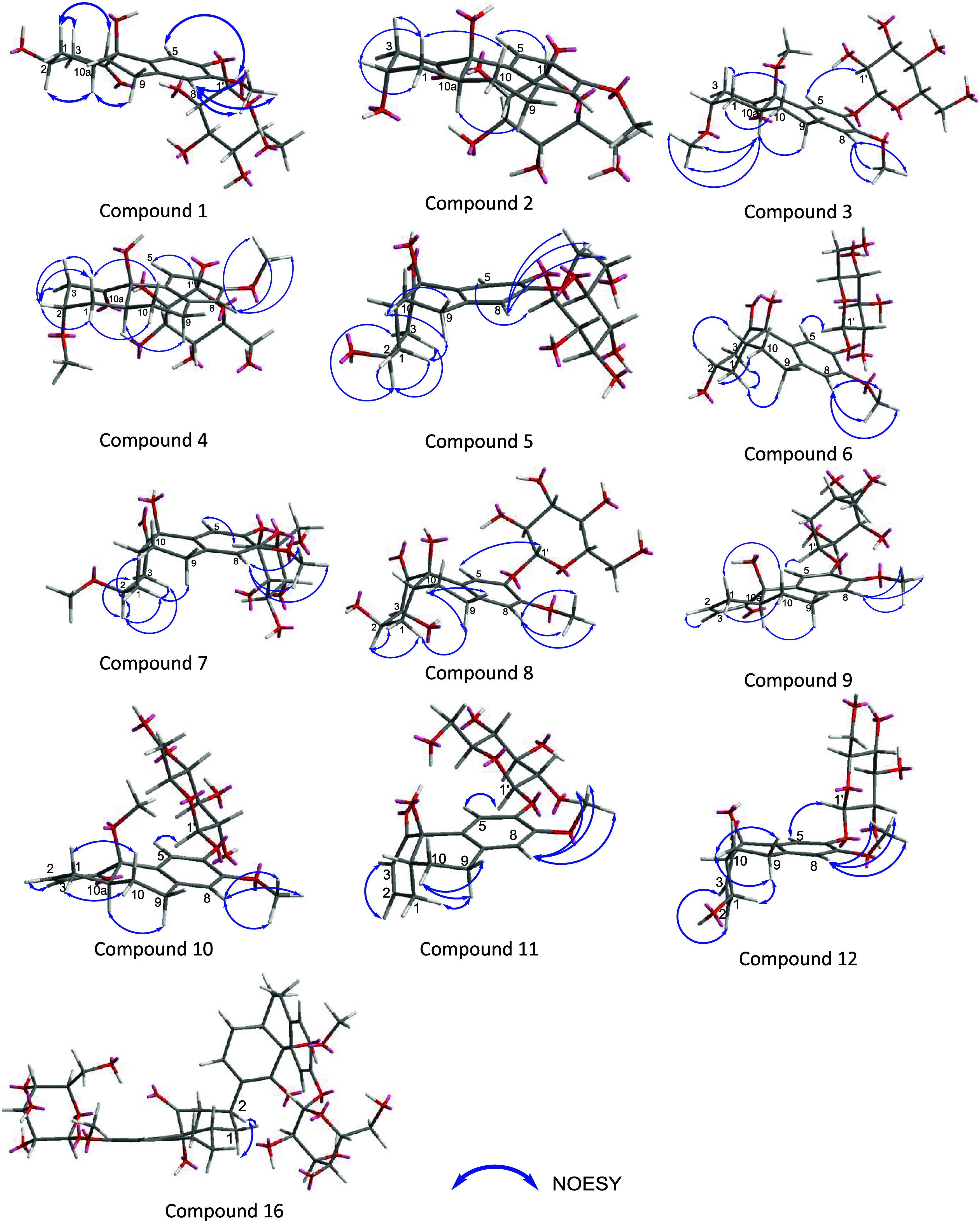
Key NOESY correlation of compounds **1–12, 16**.

Compound **2** was isolated as a white, amorphous powder and gave a molecular formula of C_21_H_28_O_10_ (degree of unsaturation = 8), as deduced from its HR-ESI-MS *m*/*z* (423.16458 [M-H_2_O + H]^+^, calculated for C_21_H_27_O_9_; *m*/*z* 485.16525 [M + HCOO]^−^, calculated for C_22_H_29_O_12_). Based on the 1D (^1^H, ^13^C) and 2D (DEPT, HSQC, HMBC, COSY, and NOESY) NMR spectra, the planar structure of compound **2** was determined to be **1** and as shown in (Figure 4). The ^13^C NMR data of compound **2** were similar to those of compound **1**, except for the signals at C-1, C-2, and C-3, suggesting that they were stereoisomers at the C-2 position. Furthermore, H-2 was assigned an equatorial orientation based on the NOE correlations with both H-1_ax_ and H-1_eq_ and the lack of NOE correlation with H-10a.^41,42^ In addition, H-1_ax_, H-3_ax_, H10_ax_, and H-9_ax_/H-10a were also indicated as the same planar, according to the cross peaks in the NOESY NMR spectra of H-1_ax_/H-3_ax_, H-10_ax_, and H-9_ax_/H-10_ax_ (Figure 5). Moreover, the similarity between the experimental CD spectrum and the calculated ECD spectrum (Figure 9) suggested the absolute configuration of **2** as shown in Figure 1, named elatostemanoside B. The NMR chemical shift data for this compound are presented in Table 1.

Compound **3** was obtained as a white, amorphous powder. Its molecular formula was determined as C_23_H_32_O_10_ (degree of unsaturation = 8) by the HRESIMS adduct molecule ion peak at *m*/*z* 491.18829 [M + Na]^+^ and *m*/*z* 513.19617 [M + HCOO]^−^. Its 1D NMR assembled those NMR data of **2**, except for the replacement of two hydroxy groups at C-2 and C-4a position by two methoxy groups which caused the C-2 signal to shift from δ_C_ 69.8 to δ_C_ 79.6 and the C-4a signal to shift from δ_C_ 78.5 to δ_C_ 83.2. The structure of **3** was determined by 2D NMR spectroscopy, and its planar structure is shown in Figure 4. Furthermore, based on the NOE correlation of H-3_ax_/H-1_ax_/H-10_ax_ and H-9_ax_/H-10a/OMe (attached to C-2), H-2 was detected in an equatorial orientation (Figure 5). In addition, according to the CD spectrum of compound **3**, comparing to the calculated ECD spectrum (Figure 9) revealed that the absolute structure, named elatostemanoside C, was in the (2*S*,4a*R*,10a*S*) form, as shown in Figure 1. The NMR chemical shift data for this compound are presented in Table 1.

Compound **4** was acquired as a white amorphous powder and had a molecular formula of C_22_H_30_O_10_ (degree of unsaturation = 8). It was concluded based on HRESIMS (*m*/*z* 477.17300 [M + Na]^+^; *m*/*z* 499.18122 [M + HCOO]^−^). By analyzing the 1D and 2D NMR spectra, **4** was found to have an overall structure similar to those of **2** and **3**; the main differences were that **4** contains a methoxy and a hydroxy group conjugated to the C-2 and C-4a positions, respectively. From the NOESY spectrum, the cross peaks of H-3_ax_/H-1_ax_/H10_ax_ and H-9/H-10a were used to detect the relative positions of the inequivalent protons of H-1, H-3, and H-10a. In addition, an H-2 signal was observed at δ_H_ 3.94 (1H, dt, 5.7, 2.8) with small coupling constants, and it exhibited NOE correlation of H-2/H-1_ax_, H-1_eq_, H-3_ax_, and H-3_eq_, suggesting the equatorial orientation of H-2 (Figure 5). Based on the similarity of the CD spectrum to that of **3** (Figure 9), the absolute structure, named elatostemanoside D, was determined and is shown in Figure 1. The NMR chemical shift data for this compound are presented in Table 1.

Compound **5** was isolated as a white amorphous powder, and its molecular formula was determined as C_21_H_28_O_10_ (degree of unsaturation = 8), based on HRESIMS data with ion peaks [M + Na]^+^ at *m*/*z* 463.15714 (calcd for C_21_H_28_O_10_Na) and [M+HCOO]^−^ at *m*/*z* 485.16513 (calcd for C_22_H_29_O_12_). The planar structure of **5** was determined to be identical to that of **1** based on analysis of the 1D and 2D NMR spectra (Figure 4). The main difference between them was the *cis* position of OH-4a and H-10a, which was identified by the NOESY cross-peak of H-1_ax_/H-9_ax_.^40^ In addition, the NOE correlation of H-2/H-1_ax_, H-1_eq_, H-3_ax_, and H-3_eq_ and the coupling constants of H-2 suggested the equatorial orientation of H-2, while OH (attached to C-2) had an axial orientation. In contrast, H-10_eq_ indicated an AN equatorial orientation by having NOESY cross-peaks with H-9_ax_ and H-9_eq_. H-3_ax_ was also confirmed by the NOE correlation between H-1_ax_/H-9_ax_, H-3_ax_ (Figure 5). In addition, the NOE signals of H-10a for both H-10_ax_ and H-10_eq_ suggested that H-10a adopts axial and *cis* positions between H-10a and OH-2. Furthermore, analysis of the 3D structure of the two stereoisomers, (2*R*,4a*R*,10a*R*) and (2*S*,4a*S*,10a*S*), revealed that only the former showed a strong correlation between H-1_ax_ and H-9_ax_ (Figure S169), and good agreement between the experimental CD and calculated ECD spectra (Figure 9) and confirmed the (2*R*,4a*R*,10a*R*) configuration of **5**. From the above analysis, the structure of **5** was deduced as shown in Figure 1 and named elatostemanoside E. The NMR chemical shift data for this compound are presented in Table 2.

**Table 2:** ^1^H NMR and ^13^C NMR Data of Compounds **5**–**8**

	5 (C_5_D_5_N_5_, 500 MHz)		6 (C_5_D_5_N_5_, 600 MHz)		7 (C_5_D_5_N_5_, 600 MHz)		8 (CD_3_OD, 600 MHz)	
position	δ_C_	δ_H_ (*J* in Hz)	δ_C_	δ_H_ (*J* in Hz)	δ_C_	δ_H_ (*J* in Hz)	δ_C_	δ_H_ (*J* in Hz)
1	34.8	2.01, td (13.4, 2.5) – α	37.4	2.09, td (12.9, 10.6) – α	31.0	1.79, m – α	26.8	1.93, ddd (15.4, 12.1, 1.5) – α
		2.12, dq (13.4, 3.0) – β		2.25, m – β		1.99, dq (14.4, 3.7) – β		2.24, ddd (15.4, 4.9, 2.5) – β
2	69.0	4.62, p (3.0)	69.8	4.31, overlap	78.3	3.77, overlap	55.0	3.43, overlap
3	47.6	2.76, overlap – α	49.9	2.93, overlap – α	43.7	2.58, dd (13.9, 3.3) – α	53.5	3.29, d (3.4)
		2.97, overlap – β		3.17, ddd (12.6, 4.6, 2.3) – β		2.88, dt (13.9, 3.3) – β		
4	212.5		211.2		211.4		205.8	
4a	79.5		79.0		79.4		77.0	
4b	131.3		131.1		131.2		133.4	
5	117.2	7.44, s	117.3	7.40, s	117.4	7.39, s	117.7	6.84, s
6	147.1		147.2		147.1		146.2	
7	150.8		150.9		150.9		151.4	
8	114.2	6.83, s	114.2	6.76, s	114.2	6.81, s	115.0	6.85, s
8a	129.2		128.9		128.8		125.3	
9	25.4	2.78, overlap – β	25.2	2.74, dd (6.7, 17.2) – β	25.2	2.76, m – β	25.0	2.82, dd (18.0, 7.2) – β
		2.95, overlap – α		2.93, overlap – α		2.92, m – α		2.94, ddd (18.0, 11.6, 7.2) – α
10	23.5	1.70, dddd (12.8, 6.3, 4.1, 2.1) – α	23.4	1.78, dt (12.4, 4.8) – α	23.2	1.67, m – α	21.7	1.66, m – α
		2.66, tdd (12.8, 6.3, 3.5) – β		2.68, m – β		2.63, m – β		2.37, m – β
10a	39.6	3.17, dq (12.2, 4.0)	39.8	2.30, m	39.4	2.80, m	32.4	2.31, m – α
1′	103.6	5.55, d (7.1)	103.8	5.49, d (7.2)	103.8	5.51, d (7.0)	103.2	4.72, d (7.2)
2′	75.4	4.30, overlap	75.4	4.28, overlap	75.4	4.30, overlap	74.9	3.46, overlap
3′	78.8	4.29, overlap	78.8	4.27, overlap	78.9	4.29, overlap	77.9	3.43, overlap
4′	71.5	4.36, overlap	71.5	4.35, overlap	71.6	4.36, overlap	71.3	3.40, t (8.6)
5′	78.8	3.93, ddd (9.6, 4.5, 2.6)	78.9	3.90, ddd (9.7, 4.5, 2.6)	79.0	3.92, ddd (9.7, 4.5, 2.7)	78.4	3.36, m
6′	62.6	4.34, overlap	62.6	4.33 (dd, 11.9, 4.44)	62.7	4.43, dd (11.8, 2.6)	62.6	3.96, dd (12.1, 2.2)
		4.43, dd (11.9, 2.6)		4.42 (dd, 11.9, 2.6)		4.34, overlap		3.72, dd (12.1, 5.3)
7-OMe	56.4	3.76, s	56.3	3.74 (s)	56.4	3.76, s	56.7	3.84 (s)
2-OMe					56.0	3.21, s		

Compound **6** was obtained as a white, amorphous powder. Its molecular formula was C_21_H_28_O_10_ (degree of unsaturation = 8) based on HRESIMS (*m*/*z* 485.16522 [M + HCOO]^−^, calcd for C_22_H_29_O_12_). Analysis of the 1D and 2D NMR spectra revealed that the planar structure of **6** was identical to that of **5** (Figure 4). However, based on the NOE correlation between H-2/H-10a, the orientation of H-2 was suggested to be axial. The other positions of the absolute configuration were identical to those of compound **5** by analyzing the NOESY spectrum similar to that of **5** (Figure 5). From the above analysis, the structure of **6** was identified as shown in Figure 1 and named elatostemanoside F. The NMR chemical shift data for this compound are presented in Table 2.

Compound **7** was obtained as a white, amorphous powder. It exhibited a molecular formula of C_22_H_30_O_10_ (degree of unsaturation = 8) from HRESIMS [*m*/*z* 477.17307 [M + Na]^+^ (calcd for C_22_H_30_O_10_Na); *m*/*z* 499.18091 [M + HCOO]^−^ (calculated for C_23_H_31_O_12_)]. Compounds **7** and **5** showed closely comparable NMR data. The major difference was the presence of an additional methoxy group at C-2 in **7** compared to the hydroxy group at C-2 in **5**. The HMBC correlation from the OMe group (δ_H_ 3.21, s) to C-2 (δ_C_ 78.3) supported this deduction (Figure 4). The absolute configuration was analyzed in a manner similar to that of **5**, which indicated that the structure of **7** was elatostemanoside G (Figure 1). The NMR chemical shift data for this compound are presented in Table 2.

Compound **8** was isolated as a white amorphous powder with a molecular formula of C_21_H_28_O_11_ (degree of unsaturation = 8), as deduced from HRESIMS (*m*/*z* 461.14154 [M-H_2_O + Na]^+^, calculated for C_21_H_26_O_10_Na; *m*/*z* 421.14903 [M-H_2_O + H]^+^, calculated for C_21_H_25_O_9_). Based on 1D and 2D NMR analyses, the planar structure of **8** closely resembled that of **5**. The major difference was the hydroxy substitution at the C-3 position in **8** (Figure 4). Based on the NOESY spectrum, the correlation between H-1/H-9 confirmed the *cis* orientation of OH-4a and H-10a, and the correlation between H-2 and both H-1_ax_ and H-1_eq_ identified the equatorial orientation of H-2. For the absolute configuration of H-3, DP4+ analysis indicated that the (3S—equatorial) configuration had a DP4+ probability of 100%, significantly outperforming the (3*R—*axial) stereoisomer, which had a probability of 0% (Supporting Information, Section 12). This result confirms the equatorial orientation of H-3. From the above analysis, the structure of **8** was established, as shown in Figure 1, and the compound was named elatostemanoside H. The NMR chemical shift data for this compound are presented in Table 2.

In addition, the similar CD spectra of **5**–**8** provided further evidence that these compounds share the same (4aR,10aR) absolute configuration (Figure 9).

Compounds **1**–**8** are glycosidic derivatives of the hexahydro-4(1*H*)-phenanthrenone group.^40^ This group has been previously used as a model for morphine synthesis.^43−48^ Interestingly, this is the first time that this structural subclass has been found in plants in both the aglycon and glycoside forms.

Compound **9** was obtained as a white powder. Its molecular formula was determined to be C_21_H_26_O_9_ (degree of unsaturation = 9) from HRESIMS [*m*/*z* 467.15457 [M + HCOO]^−^, calculated for C_22_H_27_O_11_]. The IR spectrum showed absorption bands consistent with the presence of hydroxyl (3388 cm^–1^) and conjugated carbonyl (1676 cm^–1^) groups. The UV spectrum shows absorption maxima at 206, 226, and 280 nm. Based on the ^1^H NMR spectrum, the signal of two protons at *para* position (8.35 and 6.73 ppm), two olefin protons (6.90 and 6.26 ppm), one anomeric proton [δ_H_ 5.45 (1H, d, *J =* 7.7, H-1′)], and one methoxy group (3.73 ppm) were observed. Combined with the DEPT and HSQC data, 21 carbon signals in the ^13^C NMR spectrum were assigned to be a double bond (δ_C_ 149.3, C-2; 129.0, C-3), a glucopyranosyl unit (δ_C_ 102.2, 75.2, 78.8, 71.3, 78.9, 62.7), three methylenes (δ_C_ 31.6, 30.6, 25.5), five methines (δ_C_ 149.3, 129.0, 120.6, 112.8, 44.2), one methoxy carbons (δ_C_ 56.3), and six quaternary carbons (δ_C_ 198.7, 73.2, 132.7, 145.5, 150.0, 129.7). According to the 2D spectrum, the planar structure of **9** was established as in Figure 4. The HMBC analysis revealed a correlation between the H-1′ anomeric proton and C-6, and the correlation between the OMe signal and C-7 confirmed the positions of the sugar and methoxy groups on the para-substituted benzene ring. In addition, the HMBC of H-5/C-4a; H-8/C-8a, C-9; 4a–OH/C-4, C-10a, H10/C-8a, and H-2/C-4 confirmed the positions of C-4, C-5, C-8, C-4a, C-5a, C-8a, and C9. The connections between C-9/C-10/C-10a/C-1/C-2/C-3 and C-1′/C-2′/C-3′/C4′/C-5′/C-6′ were established according to the COSY spectrum. Furthermore, based on the NOE correlation between the anomeric protons H-1′ and H-5, as well as the HMBC correlation between H-1′ and C-6, the positions of C-5, C-6 and C-1′ of sugar were identified. Similarly, the NOESY correlations of the methoxy group/H-8 and the HMBC cross-peak of methoxy/C-7 confirmed the positions of the methoxy group, C-6, and C-7 (Figures 4 and 5). The *J* value of 10.1 Hz for H-2 and H-3 suggested a *cis* conformation. Specifically, in the NOESY spectrum, the correlation between H-10_ax_/H1_ax_ and H-10_eq_/H-1_eq_ confirmed the *trans* positions of H-10a and OH-4a. The absolute configuration of **9** was identified to be (4a*R*,10a*S*), based on the experimental CD and calculated ECD spectra (Figure 9). The structure of **9** is shown in Figure 1, and it was named elatostemanoside I. The NMR chemical shift data for this compound are presented in Table 3.

**Table 3:** ^1^H NMR and ^13^C NMR Data of Compounds **9**–**12**

	9 (C_5_D_5_N_5_, 600 MHz)		10 (C_5_D_5_N_5_, 600 MHz)		11 (C_5_D_5_N_5_, 600 MHz)		12 (C_5_D_5_N_5_, 600 MHz)	
position	δ_C_	δ_H_ (*J* in Hz)	δ_C_	δ_H_ (*J* in Hz)	δ_C_	δ_H_ (*J* in Hz)	δ_C_	δ_H_ (*J* in Hz)
1	31.6	2.14, overlap – α	31.5	2.57, m – β	29.8	2.27, ddt (19.4, 8.9, 2.9) – α	66.9	4.68, brd (9.4)
		2.80, m – β		2.08, m – α		2.47, dtd (19.4, 5.4, 1.6) – β		
2	149.3	6.90, ddd (10.1, 5.3, 2.2)	149.4	6.81, ddd (10.1, 5.3, 2.2)	150.7	6.79, ddd (10.1, 4.9, 2.8)	151.8	7.16, dd (10.3, 2.1)
3	129.0	6.26, dd (10.1, 2.5)	128.3	6.08, dd (10.1, 2.8)	128.0	6.18, ddd (10.0, 2.8, 1.3)	126.7	6.24, dd (10.2, 2.4)
4	198.7		198.1		201.1		201.7	
4a	73.2		77.2		76.4		76.1	
4b	132.7		134.6		131.4		131.9	
5	120.6	8.35, s	122.5	8.46, s	117.3	7.75, s	116.7	7.66, s
6	145.5		144.6		146.7		146.7	
7	150.0		150.7		150.7		150.8	
8	112.8	6.73, s	113.5	6.78, s	114.0	6.74, s	113.9	6.76, s
8a	129.7		123.8		128.8		128.1	
9	30.6	2.70, m	30.2	2.74, m	25.9	2.90, ddd (16.9, 9.5, 7.0) – α	25.1	2.91, brdd (7.5, 7.3) – β
						2.81, ddd (17.6, 7.0, 3.8) – β		3.19, ddd (18.1, 11.4, 7.3) – α
10	25.5	2.20, overlap – β	25.6	2.12, overlap – β	23.2	1.74, m – α	19.0	2.85, m – β
		1.58, m – α		1.51, m – α		2.52, m – β		2.63, m – α
10a	44.2	2.18, overlap	43.7	2.21, overlap	41.3	2.68, m	51.2	3.01, dt (9.4, 3.4)
1′	102.2	5.45, d (7.7)	103.4	5.74, d (7.2)	103.6	5.54, d (7.5)	103.8	5.51, d (7.3)
2′	75.2	4.29, dd (9.0, 7.7)	75.4	4.40, overlap	75.4	4.29, t (8.3)	75.3	4.29, overlap
3′	78.8	4.17, t (9.0)	79.1	4.40, overlap	79.0	4.25, t (8.8)	78.9	4.28, overlap
4′	71.3	4.41, t (9.0)	71.6	4.48, overlap	71.6	4.38, overlap	71.5	4.40, overlap
5′	78.9	3.76, ddd (9.0, 4.2, 2.6)	79.2	4.14, ddd (9.6, 4.3, 2.6)	78.9	3.90, ddd (9.6, 4.3, 2.6)	78.9	3.97, ddd (9.7, 4.2, 2.7)
6′	62.7	4.69, dd (11.9, 2.6)	62.8	4.72, dd (11.9, 2.6)	62.8	4.37, overlap	62.7	4.48, dd (11.9, 2.7)
		4.46, dd (11.9, 4.2)		4.52, overlap		4.46, dd (11.9, 2.6)		4.40, overlap
7-OMe	56.3	3.73, s	56.2	3.75, s	56.4	3.70, s	56.3	3.68, s
4a–OH		7.69, s						
4a-OCH_3_			53.6	3.26, s				

Compound **10** was acquired as a white powder with the molecular formula of C_22_H_28_O_9_ (degree of unsaturation = 9) that was established by HRESIMS data [*m*/*z* 459.16220 [M + Na]^+^ (calculated for C_22_H_28_O_9_Na, *m*/*z* 481.17001 [M + HCOO]^−^, calculated for C_23_H_29_O_11_)]. The NMR data of **10** were similar to those of **9**, except for the methoxy group (δ_H_ 3.26; δ_C_ 53.6–4a-OMe) in **10** replacing the 4a–OH group in **9**. Thus, **10** was a 4a-methoxy derivative of **9**, as confirmed by the HMBC correlation from the methoxy group to C-4a (Figure 4). Similar to the case for **9**, the *trans* positions of 4a-OMe and H-10a were confirmed by the NOE correlations between H10_ax_/H1_ax_ and H10_eq_/H-1_eq_ (Figure 5). Because of the similarity of the experimental CD spectra to those of **9** (Figure 9), the absolute configuration of **10** was determined to be the (4a*R*,10a*S*) form, which is shown in Figure 1 and named elatostemanoside J. The NMR chemical shift data for this compound are presented in Table 3.

Compound **11** was obtained as a white, amorphous powder. It was found to possess a molecular formula of C_21_H_26_O_9_ (degree of unsaturation = 9) which was identified from its HRESIMS data (*m*/*z* 467.15457 [M + HCOO]^−^, calculated for C_22_H_27_O_11_). Analysis of the 1D and 2D NMR spectra revealed that the planar structure of **11** was the same as that of **9** as shown in Figure 4. Furthermore, NOESY correlation between H-1_ax_/H-9_ax_, and H-10a/H-10_ax,eq_ suggested *cis* position of H-4a and H-10a with the (4a*R*,10a*R*) configuration because these correlation cannot be found at the (4a*S*,10a*S*) orientation (Figure S170) and the similarity of experimental CD to calculated ECD spectrum of (4a*R*,10a*R*) form confirmed the (4a*R*,10a*R*) configuration of **11** (Figure 9). Based on the above analyses, the chemical structure of **11** was identified as elatostemanoside K, as shown in Figure 1. The NMR chemical shift data for this compound are presented in Table 3.

Compound **12**, a white amorphous powder, showed a molecular formula of C_21_H_26_O_10_ (degree of unsaturation = 9) which was determined by HRESIMS data (*m*/*z* 461.14108 [M + Na]^+^, calculated for C_21_H_26_O_10_Na, *m*/*z* 483.14954 [M + HCOO]^−^, calculated for C_22_H_27_O_12_), and had one more oxygen atom than **11**. The NMR data of compound **12** closely resembled those of compound **11**. The major difference was the downfield of C-1 (δ_C_ = 66.9) compared to that of **11**(δ_C_ = 29.8). Consequently, **12** was identified as the 1-hydroxy derivative of **11**, and its structure was established based on the 1D and 2D NMR spectral data, as shown in Figure 4. In addition, by an analysis similar to that of **11**, the (4a*R*,10a*R*) orientation was determined by the NOE correlation between H-1_ax_/H-9_ax_ and H10a/H-10_ax,eq_ (Figure S170), and experimental CD—calculated ECD comparison (Figure 9). Besides, the NOE signals of H-1_ax_/H-9_ax_ (Figure 5) also suggested the β-*pseudoequatorial*-orientated 1-OH group. The structure of compound **12** was named elatostemanoside L, as displayed in Figure 1. The NMR chemical shift data for this compound are presented in Table 3.

In addition, the similar CD spectra of compounds **5**–**8**, **11**, and **12** provide further evidence that compounds **5**–**8**, **11**, and **12** share the same (4a*R*,10a*R*) configuration in their absolute structure (Figure 9).

Compounds **9**–**12** belonged to the glycosidic tetrahydro-4(1*H*)-phenanthrenone group. Although tetrahydro-4(1*H*)-phenanthrenone was synthesized as a model for morphine synthesis, similar to compounds **1**–**8**, this is the first time that this group has been isolated from plants.^45^

Compounds **13** (molecular formula C_21_H_24_O_8_, degree of saturation = 10) and **14** (C_27_H_34_O_12_, degree of saturation = 10) were identified as 7-methoxy-4-hydroxy-9,10-dihydrophenanthrene 6-*O*-β-d-glucopyranoside and 7,4-dihydroxy-9,10-dihydrophenanthrene 6-*O*-β-d-glucopyranoside, respectively. They were isolated from the same plant, as described in a previous study.^21^ However, they had not been named; therefore, in this study, we named them elatostemanosides M and N (the elucidation is detailed in Supporting Information (Section 4)). The NMR chemical shift data for **13**–**14** are presented in Table 4.

**Table 4:** ^1^H NMR and ^13^C NMR Data of Compounds **13**–**15**

	13 (C_5_D_5_N_5_, 600 MHz)		14 (CD_3_OD, 500 MHz)		15 (C_5_D_5_N_5_, 600 MHz)	
position	δ_C_	δ_H_ (*J* in Hz)	δ_C_	δ_H_ (*J* in Hz)	δ_C_	δ_H_ (*J* in Hz)
1	120.1	6.90, dd (8.0, 1.3)	120.4	7.70, overlap	120.1	6.87, d (7.3)
2	128.0	7.14, t (8.0)	128.0	6.94, dd (8.1, 7.3)	128.0	7.12, t (7.3)
3	116.5	7.19, dd (8.0, 1.3)	115.9	6.74, dd (8.1, 1.2)	116.5	7.23, overlap
4	156.4		155.3		156.4	
4a	122.9		122.6		122.8	
4b	133.4		135.6		133.9	
5	118.7	9.30, s	119.7	8.36, s	119.9	9.29, s
6	146.3		144.6		146.3	
7	148.9		146.6		149.4	
8	112.9	6.94, s	115.8	6.71, s	112.9	6.89, s
8a	127.5		126.4		127.5	
9	30.2	2.76, m	30.4	2.64, m	30.2	2.70, m
10	31.4	2.82, m	31.5	2.70, m	31.4	2.80, m
10a	140.6		140.7		140.5	
1′	103.5	5.81, *d* (7.4)	104.8	4.79, d (7.5)	104.3	5.70, d (6.9)
2′	75.4	4.36, overlap	74.9	3.51, overlap	75.5	4.33, overlap
3′	79.1	4.36, overlap	77.8	3.48, overlap	79.0	4.32, overlap
4′	71.3	4.47, overlap	70.9	3.54, overalp	71.4	4.30, overlap
5′	79.0	4.08, ddd (9.6, 4.0, 2.6)	78.1	3.38, ddd (9.5, 4.0, 2.5)	77.7	4.12, m
6′	62.6	4.36, overlap	62.1	3.92, dd (12.1, 2.5)	67.7	4.55, brd (11.0)
		4.49, overlap		3.81, dd (12.1, 4.0)		4.20, overlap
1″					102.7	5.43, s
2″					72.5	4.48, overlap
3″					73.0	4.50, overlap
4″					74.6	4.20, overlap
5″					70.0	4.30, overlap
6″					19.0	1.53, d (6.2)
7-OMe	56.5	3.79, s	56.5	3.78, s		

Compound **15** was obtained as a white, amorphous powder. It exhibited the molecular formula of C_27_H_34_O_12_ (degree of saturation = 11), which was deduced from the HRESIMS data (*m*/*z* 551.21246 [M + H]^+^, calculated for C_27_H_35_O_12_; *m*/*z* 573.18421 [M + Na]^+^, calculated for C_27_H_34_O_12_Na) and may have one more sugar (C_6_H_10_O_4_) group than **13**. In addition, the NMR data of **15** closely resembled those of **13**. The major difference was the downfield shift of C-6′ to δC 67.7, along with the hexose group signals at δ_C_ 102.7, 74.6, 73.0, 72.5, 70.0, and 19.0. These data suggest that **15** had more than one 6′-hexose derivative of **13**. This conclusion was confirmed by the COSY correlation of H-1″/H-2″/H-3″/H-4″/H-5″/H-6″ and the HMBC correlation from the anomeric proton H-1″ to C-6′ as well as the aforementioned determination of the sugar structure (Figure 4). In addition, the anomeric proton H-1′ with a *J* value of 6.9 and the singlet anomeric proton H-1″ indicated the β-orientation of glucose and the α-orientation of rhamnose, respectively. Therefore, the structure of **15**, as shown in Figure 1, was named elatostemanoside O. The NMR chemical shift data for this compound are presented in Table 4.

Unlike hexahydro- and tetrahydro-4(1*H*)-phenanthrenones, which are rarely found in plants, dihydrophenanthrenes are mainly found in the Orchidaceae, Juncaceae, Dioscoreaceae, and Berberidaceae families with a variety of pharmacological activities including cytotoxic, anti-inflammatory, antimicrobial, and antioxidant effects. Dihydrophenanthrenes typically exist as aglycones, whereas glycosides such as compounds **13**–**15** are rarely found.^49^

By examining the difference in chemical shifts between H-9_ax_ and H-9_eq_, a small difference (approximately 0.06 ppm) or no separation was observed in the (4a*R*,10a*S*) configuration (compounds **1–4, 9–10**), while the (4a*R*,10a*R*) configuration showed clear separation (Δδ_H9_ = 0.15 ± 0.03 ppm for compounds **5–8 (**hexahydro-4(1*H*)-phenanthrenone group) and 0.1 ppm for compound **11** (tetrahydro-4(1*H*)-phenanthrenone group)). This discrepancy was attributed to the 3D structure of the (4a*R*,10a*R*) form, which significantly increased the correlation between H-1_ax_ and H-9_ax_, thereby affecting the chemical shifts of both the protons of H-9.^40^

Compound **16**, obtained as a white amorphous powder, had a molecular formula of C_42_H_52_O_18_ (degree of saturation = 17), which was identified by HRIESIMS data (*m*/*z* 867.30370 [M + Na]^+^, calculated for C_42_H_52_O_18_Na). Analysis of the 1D and 2D NMR spectra, similar to compounds, the planar structure of **16**, shown in Figure 4, was similar to those of **1** and **11**. For absolute configuration, the (4a*R*,10a*S*), (4a′*R*,10a′*R*) orientation was also deduced by the Δδ_H-9_ and Δδ_H-9′_ values of 0.05 and 0.16 ppm, respectively. According to the NOESY spectrum, the correlation between H-2/H-1_ax,eq_ suggested α-equatorial orientation of H-2 (Figure 5). From the above analysis, the structure of compound **16**, as exhibited in Figure 1, was named elatostemanoside P. The NMR chemical shift data for this compound are presented in Table 5.

**Table 5:** ^1^H NMR and ^13^C NMR Data of Compounds **16**–**17**

16 (CD_3_OD, 600 MHz)						17 (CD_3_OD, 600 MHz)					
position	δ_C_	δ_H_ (*J* in Hz)	position	δ_C_	δ_H_ (*J* in Hz)	position	δ_C_	δ_H_ (*J* in Hz)	position	δ_C_	δ_H_ (*J* in Hz)
1	31.4	1.86, m	1′	28.9	2.33, ovl	1	72.2	4.13, t (4.1)	1′	66.7	4.18, d (9.6)
		2.27, m			2.47, ovl						
2	36.0	3.28, m	2′	146.2	6.75, m	2	39.6	3.39, overlap	2′	151.5	6.84, m
3	43.8	2.49, overlap	3′	139.6		3	38.5	2.28, dd (14.8, 5.6)	3′	136.0	
		2.83, overlap						3.00, dd (14.8, 4.8)			
4	213.0		4′	201.2		4	211.5		4′	201.4	
4a	79.8		4a′	76.5		4a	79.3		4a′	76.3	
4b	132.9		4b′	132.3		4b	133.1		4b′	132.9	
5	117.4	6.86, s	5′	116.6	6.82, s	5	117.4	6.89, s	5′	116.7	6.72, s
6	146.2		6′	146.5		6	146.3		6′	146.3	
7	150.7		7′	150.8		7	150.9		7′	151.1	
8	113.6	6.78, s	8′	114.4	6.81, s	8	113.5	6.81, s	8′	114.6	6.82, s
8a	129.6		8a′	127.1		8a	129.0		8a′	127.0	
9	27.5	2.83, overlap	9′	25.1	2.84, overlap	9	27.9	2.87, overlap	9′	24.8	2.85, overlap
		2.88, overlap			3.00, ddd (18.2, 11.0, 7.4)			2.95, overlap			3.04, overlap
10	25.1	1.71, m	10′	22.5	1.76, m	10	23.8	1.91, m	10′	18.4	2.21, m
		2.04, m			2.39, m			2.05, brs			2.32, m
10a	42.6	2.33, overlap	10a′	40.8	2.47, overlap	10a	49.5	2.46, dt (8.2, 4.2)	10a′	50.1	2.37, overlap
Glc1	102.7	4.81, d (7.8)	Glc1′	103.1	4.87, d (7.2)	Glc1	102.7	4.83 overlap	Glc1′	103.3	4.72, d (6.2)
glc2	74.9	3.47, overlap	glc2′	74.9	3.47, overlap	glc2	74.9	3.47 overlap	glc2′	74.9	3.44, overlap
glc3	78.2	3.38, overlap	glc3′	78.4	3.38, overlap	glc3	77.7	3.38 overlap	glc3′	77.7	3.44, overlap
glc4	71.4	3.37, overlap	glc4′	71.2	3.39, overlap	glc4	71.3	3.37 overlap	glc4′	71.1	3.38, overlap
glc5	77.7	3.47, overlap	glc5′	77.7	3.47, overlap	glc5	78.1	3.47 overlap	glc5′	78.4	3.47, overlap
glc6	62.6	3.66, dd (12.2, 4.8)	glc6′	62.5	3.72, dd (12.2, 4.7)	glc6	62.4	3.67 overlap	glc6′	62.6	3.67, overlap
		3.88, dd (12.2, 1.8)			3.95, dd (12.2, 1.7)			3.87 overlap			3.87, overlap
7-OMe	56.3	3.84, s	7′-OMe	56.6	3.84, s	7-OMe	56.7	3.86 s	7′-OMe	56.6	3.83, s

Compound **17** was obtained as a white, amorphous powder. It exhibited the molecular formula C_42_H_52_O_20_ (degree of saturation = 17), which was deduced from the HRESIMS data (*m*/*z* 899.29510 [M + Na]^+^, calculated for C_42_H_52_O_20_Na). Through analysis of the 1D and 2D NMR spectra, the 2D structure of **17**, comprising two monomeric units, was determined (Figure 4). One of these units resembled **12**, except for differences at the C3′ position. By comparing the ^13^C NMR spectra of **17** to **12**, the (4a′*R*,10a′*R*) configuration and the α-axial orientation of H-1′ were established. The large *J* coupling of H-1′ by 9.4 Hz and Δδ_H-9′_ of 0.19 ppm gave more evidence for this conclusion. In addition, the (4a*R*,10a*S*) orientation was also established by small Δδ_H-9′_ of 0.08 ppm. Besides, the small *J* coupling of H-1 by 4.1 Hz and H-3 by 5.6 and 3.6 Hz suggested the equatorial orientation of H-1 and H-2. The structure of **17**, as shown in Figure 1, was named elatostemanoside Q. The NMR chemical shift data for this compound are presented in Table 5.

Based on the molecular networking (MN)-guided isolation of Group 1 nodes, we successfully isolated 17 glycosidic compounds from *E. tenuicaudatum*. Notably, the results showed a good match between the predicted and the experimental results. First, none of the Group 1 nodes were identified by the GNPS databases, indicating that many of the compounds in this group could be new structures. In this group, 15 isolated compounds were found to be novel. Second, in silico fragmentation analysis of Group 1 nodes suggested a relationship between the terpene, stilbene, and phenolic glycoside patterns, and most of these nodes were closely connected in Cluster D. Interestingly, the isolated compounds were hydrogenated phenanthrene glycosides, which shared these structural patterns. For example, beyond being glycosidic derivatives, compounds **13**–**15**, if lacking a bridge between C-4a and C-4b, can be classified as stilbenes; all of them exhibit phenolic structures (compounds **13**–**15** contain phenolic rings, while the others are methoxy phenolic derivatives) and the core structure of the isolated compounds consists of a tricyclic 6/6/6 scaffold, commonly found in diterpenes or triterpenes.^50−53^ However, the in silico fragmentation software did not suggest hydrogenated phenanthrene glycosidic derivatives, likely because of the rarity of these structures and the limited MS/MS data available in natural product databases. Finally, Group 1, which contained the largest cluster in MN, was predicted to represent the major chemical group in this vegetable. By matching the isolated compounds with the MN and UHPLC chromatograms, we confirmed that this group was indeed the predominant chemical class, corresponding to the major peaks and retention times in the UHPLC analysis. This analysis demonstrates that combining UHPLC-MS/MS with MN is an effective approach for identifying the chemical structure classes of a material, supporting targeted isolation, and elucidating structures.

### 3.3 DPP-IV Inhibition Activity

**Figure 6:**
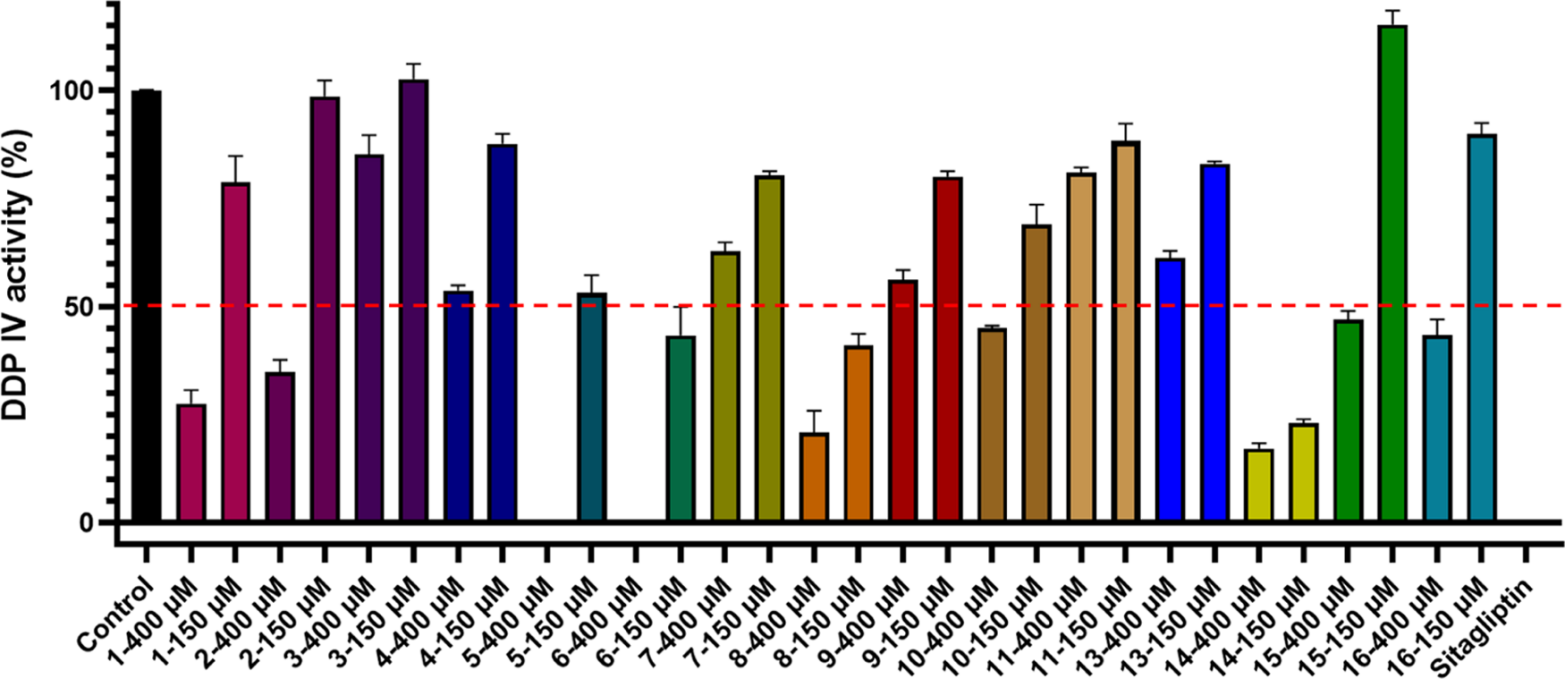
Effect of compounds **1–11, 13–16** on DPP-IV activity at 400 μM and 150 μM.

Due to the limited yields of compounds **12** and **17**, only 15 compounds (compounds **1**–**11**, **13**–**16**) were screened for DPP-IV inhibitory activity. These compounds were tested at concentrations of 400 and 150 μM, showing a dose-dependent effect, with stronger inhibition at higher concentrations (Figure 6). At 400 μM, compounds **1**, **2**, **5**, **6**, **8**, **10**, **14**, **15**, and **16** inhibited DPP-IV activity by more than 50%. At 150 μM, only compounds **5**, **6**, **8**, and **14** maintained inhibition levels at or above 50%. These four compounds, along with the crude extract, were further evaluated for their IC_50_ values (Table 6), revealing moderate inhibition, with IC_50_ values ranging from 71.9 to 151.2 μM, and compound **14** displaying the strongest activity with an IC_50_ of 71.9 ± 8.9 μM. This is the first study to demonstrate that these compounds inhibit DPP-IV activity. Compared to classical diabetes drugs, such as sulfonylureas, biguanides, thiazolidinediones, and insulin therapy, DPP-IV inhibitors offer a better safety profile, particularly for stabilizing blood sugar in patients with type 2 diabetes without causing hypoglycemia or weight gain. Several studies have highlighted the benefits of combining Western medicine with plant-based treatments.^54^ In this study, *E. tenuicaudatum* showed mild DPP-IV inhibition, with compounds **5**, **6**, **8**, and **14** contributing to its antidiabetic effects. These findings support the potential of *E. tenuicaudatum* as a promising natural resource for blood sugar regulation.

**Table 6:** IC_50_ Values of Compounds **5**,**6**,**8**,**14** and Crude Extract on DPP-IV Inhibition Activity

compound	IC_50_ value
5	141.7 ± 15.6 (μM)
6	151.2 ± 11.8 (μM)
8	107.9 ± 19.6 (μM)
14	71.9 ± 8.9 (μM)
crude extract	220.5 ± 39.6 (μg/mL)
positive control (sitagliptin)	13.5 ± 3.6 (nM)

### 3.4 Mechanism of Four DPP-IV Inhibitors in the Binding Potency of DPP-IV

**Figure 7:**
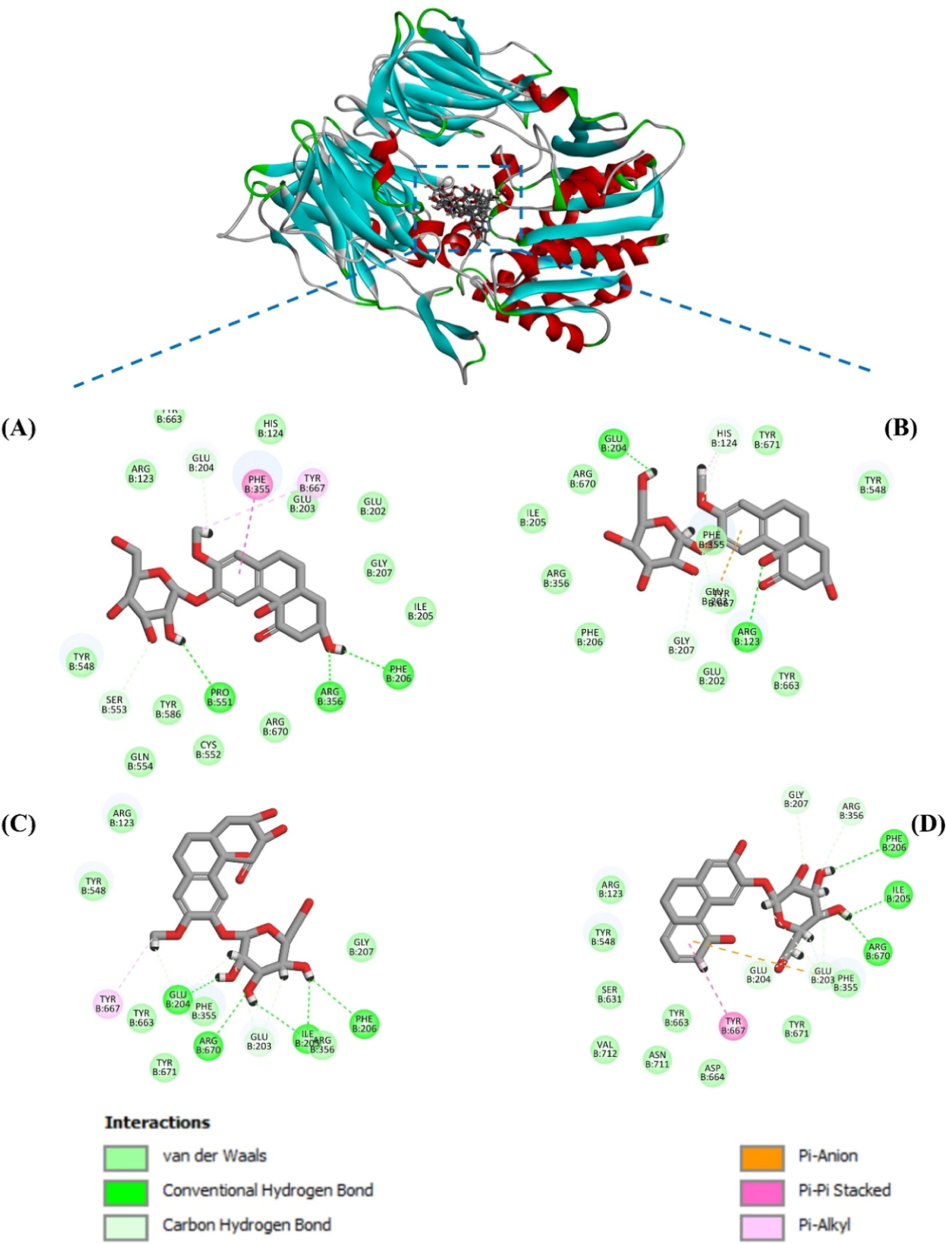
Binding modes between dipeptidyl peptidase IV (PDB code: 4FFW) and DPP-IV inhibiting components were calculated by molecular docking. View of docking conformations for the four inhibitors binding to the active sites of dipeptidyl peptidase IV (PDB code: 4FFW): (A) Compound 5; (B) Compound 6; (C) Compound 8; and (D) Compound 14.

The molecular docking study elucidated the possible molecular interaction patterns of the four potential DPP-IV inhibitors (compounds **5**, **6**, **8**, and **14**) with DPP-IV protein, with the cocrystal structure of dipeptidyl peptidase IV (DPP-IV) in complex with Fab and sitagliptin (PDB ID: 4FFW). Sitagliptin was used as a reference ligand for comparison. Focusing particularly on interactions with structural amino acid residues in the active cavity is important for effective and selective inhibition of DPP-IV activity. Each compound’s most appropriate docking pose (with the lowest CDOCKER interaction energy) was selected from the corresponding ten molecular docking simulations for action mechanism interpretation. Images of the docking conformation of the pose chosen for each compound are shown in Figure 7.

As a result, CDOCKER binding energy and Δ*G* of four compounds have the same trend as their IC_50_ values in the in vitro method, and the latter supported the molecular docking predictions were reliable.

The CDOCKER interaction energy and binding affinity (Δ*G*) for the four potential compounds were as follows: **14** (−35.8064 and −45.2788 kcal/mol), **8** (−34.6053 and −44.2976 kcal/mol), **5** (−39.3408 and −38.5752 kcal/mol), and **6** (−36.424 and −33.3263 kcal/mol). These negative values suggest that all of the tested compounds have potential as DPP-IV inhibitors (Table 7). According to previous reports, DPP-IV binding sites include the S1, S2, S1′, and S2′ sites.^55^ We observed that these four potential DPP-IV inhibitors also exhibited efficacy at these binding sites.

**Table 7:** Interaction Energy and Binding Affinity of Potential Ligands with DPP-IV (PDB Code: 4FFW) by Molecular Docking

			interaction residue				
compound	CDOCKER interaction energy (kcal/mol)	binding affinity (Δ*G*, kcal/mol)	hydrogen bond	Pi–pi	Pi–alkyl	Pi–anion	halogen
compound **5**	–39.3408	–38.5752	Glu204, Ser553, Pro551, Phe206,, Arg356	Phe355	Tyr667		
compound **6**	–36.424	–33.3263	Glu203,, Glu204, Arg123, Gly207, His124		His124	Glu203	
compound **8**	–34.6053	–44.2976	Glu204, Glu203, Arg670, Ile205, Phe206		Tyr667		
compound **14**	–35.8064	–45.2788	Glu203, Glu204, Ile205, Phe206, Gly207, Arg356, Arg670	Tyr667		Glu203	
sitagliptin	–45.8343	–46.517	Arg356, Arg123, Glu204, Arg670, Tyr663, Asn711, Ser631, Glu203	Tyr548, Phe206	Tyr667, Val712, Tyr663		Ile205, Phe355, Glu204, Arg670, Asn711, His741

Among them, compound **14** exhibited the highest docking score, indicating the strongest binding interaction with DPP-IV. It formed extensive hydrogen bonds with residues Glu203, Glu204, Ile205, Phe206, Gly207, Arg356, and Arg670. Additionally, pi–pi interactions with Tyr667 and pi–anion interactions with Glu203 contributed to its robust binding profile (Table 7 and Figure 5). The compound **8** complex also demonstrated strong binding characteristics, forming significant hydrogen bonds with key residues such as Glu204, Glu203, Arg670, Ile205, and Phe206. The pi–pi interaction with Tyr667 further supports its effective binding to DPP-IV. The compound **5** complex showed moderate binding interactions, including hydrogen bonding with Glu204, Ser553, Pro551, and Phe206. Pi–pi interactions with Arg356 and Phe355, as well as a pi–alkyl interaction with Tyr667, suggest moderate binding capability to DPP-IV. The complex **6** exhibited the lowest binding affinity and interaction energy. It formed hydrogen bonds with Glu203, Glu204, Arg123, Gly207, and His124, along with pi–alkyl interactions with His124 and pi–anion interactions with Glu203. Despite its lower binding affinity, these interactions provided valuable insights into its binding mechanism.

The docking results indicated that hydrogen bonds and hydrophobic interactions play crucial roles in the effective binding of the DPP-IV inhibitors. Key residues in the active site, such as Glu203, Glu204, Arg356, Arg670, and Tyr667, are particularly important for the strong and selective inhibition of DPP-IV activity. Previous reports indicate that the active binding sites of the sitagliptin inhibitor are located at S1, S2, and S2′. The binding site of compound **14** was similar to that of sitagliptin, which may account for its superior DPP-IV inhibitory activity.^56^ Additionally, halogen bonding was observed in the sitagliptin complex. It has been reported that halogen groups form halogen bonds with the carboxyl group (C=O) of amino acid residues and that their electrophilicity boosts the efficacy of agonists. These interactions influence the specific properties and affinity of the compound, resulting in increased potency.^57^

### 3.5 Assessment of the Safety and Hepatoprotective Effects of Compounds in HepG2 Cells

Safety is a primary consideration when assessing food or drug compounds, particularly regarding their potential impact on liver health.^58^ To investigate the liver safety and hepatoprotective effects of compounds **1**–**17**, we measured their inhibitory effects on HepG2 cell viability using the MTT assay. We also evaluated their protective effects against acetaminophen-induced liver cell injury.^59^

**Figure 8:**
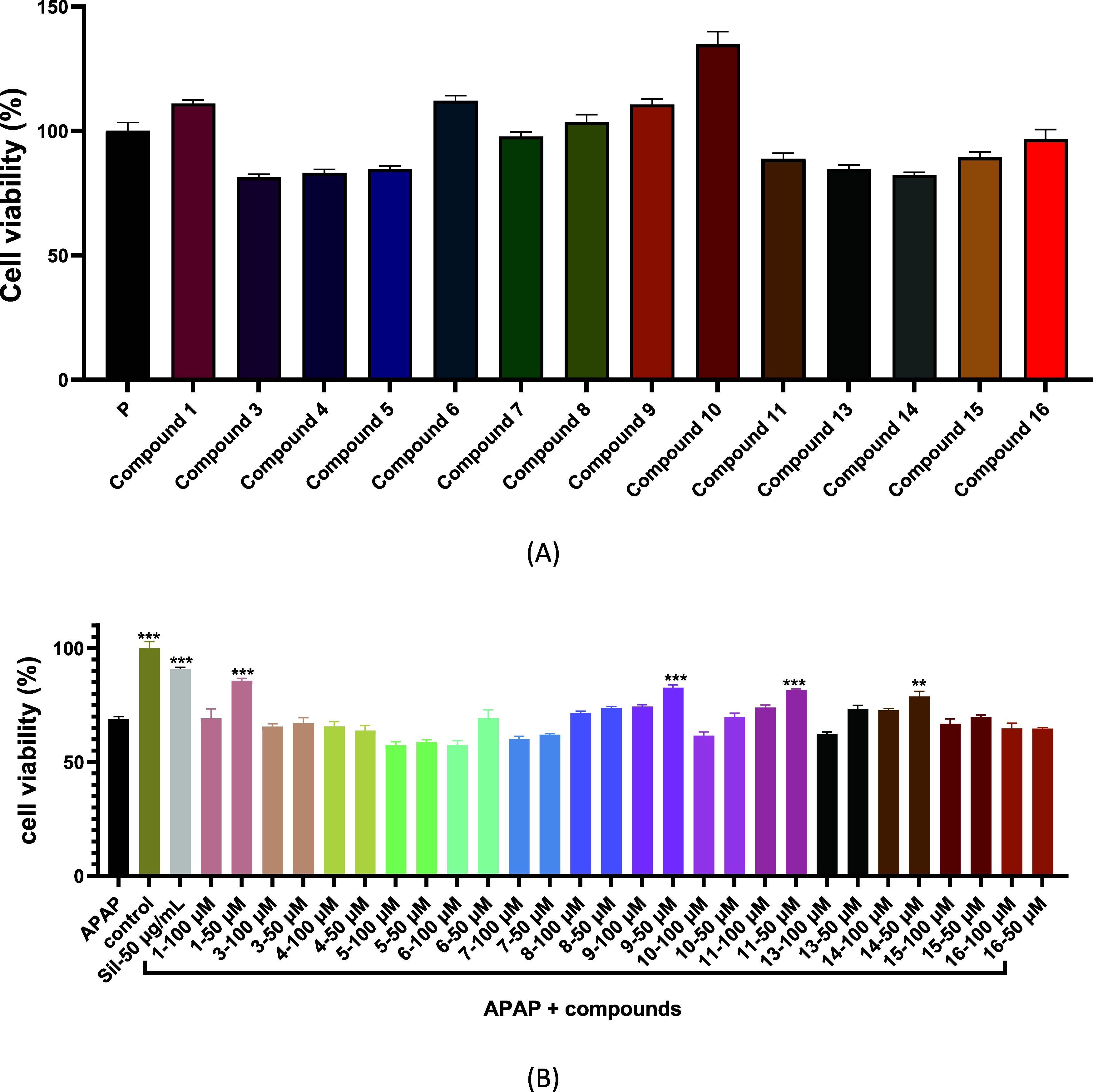
HepG2 cytotoxicity test at 100 μM (A) and hepatoprotective prevent APAP toxicity of compounds **1**, **3**–**11**, **13**–**16** (100 and 50 μM, pretreated for 8 h) by 6 mM APAP for 36 h (**P < 0.01 vs APAP; ***P < 0.001 vs APAP) (B).

The viability of HepG2 cells was initially tested at a concentration of 100 μM for the pure compounds. Because of the insufficient amounts of compounds **2**, **12**, and **17**, we screened only compounds **1**, **3**–**11**, and **13**–**16**. Results indicated that all screened compounds exhibited cell viabilities of >80% (Figure 8A), demonstrating that most of the major phytochemicals showed favorable safety profiles. Based on these findings, we further evaluated whether the compounds attenuated cellular injury induced by APAP in cultured HepG2 cells at concentrations of 100 and 50 μM. Compounds **1**, **9**, **11**, and **14** showed significant cytoprotection with improving cell viability of 85.6, 82.6, 81.6, and 78.8%, respectively, at 50 μM (Figure 8B). Notably, compounds **9**, **11** and **14** were the major components of this plant (Figure 2B). Previous studies have reported that DPP-IV inhibitors not only help regulate blood glucose levels but also inhibit DPP-IV activity in the liver tissues and hepatocytes of diabetic mice. Moreover, they reduce cytokine production and inflammation, which may prevent the progression of diabetic liver disease.^5^ Our preliminary findings showed that the novel DPP-IV inhibitors in **14** demonstrated the potential for multiple functions, but further exploration is needed to elucidate the mechanism and enhance confidence through in vivo models.

**Figure 9:**
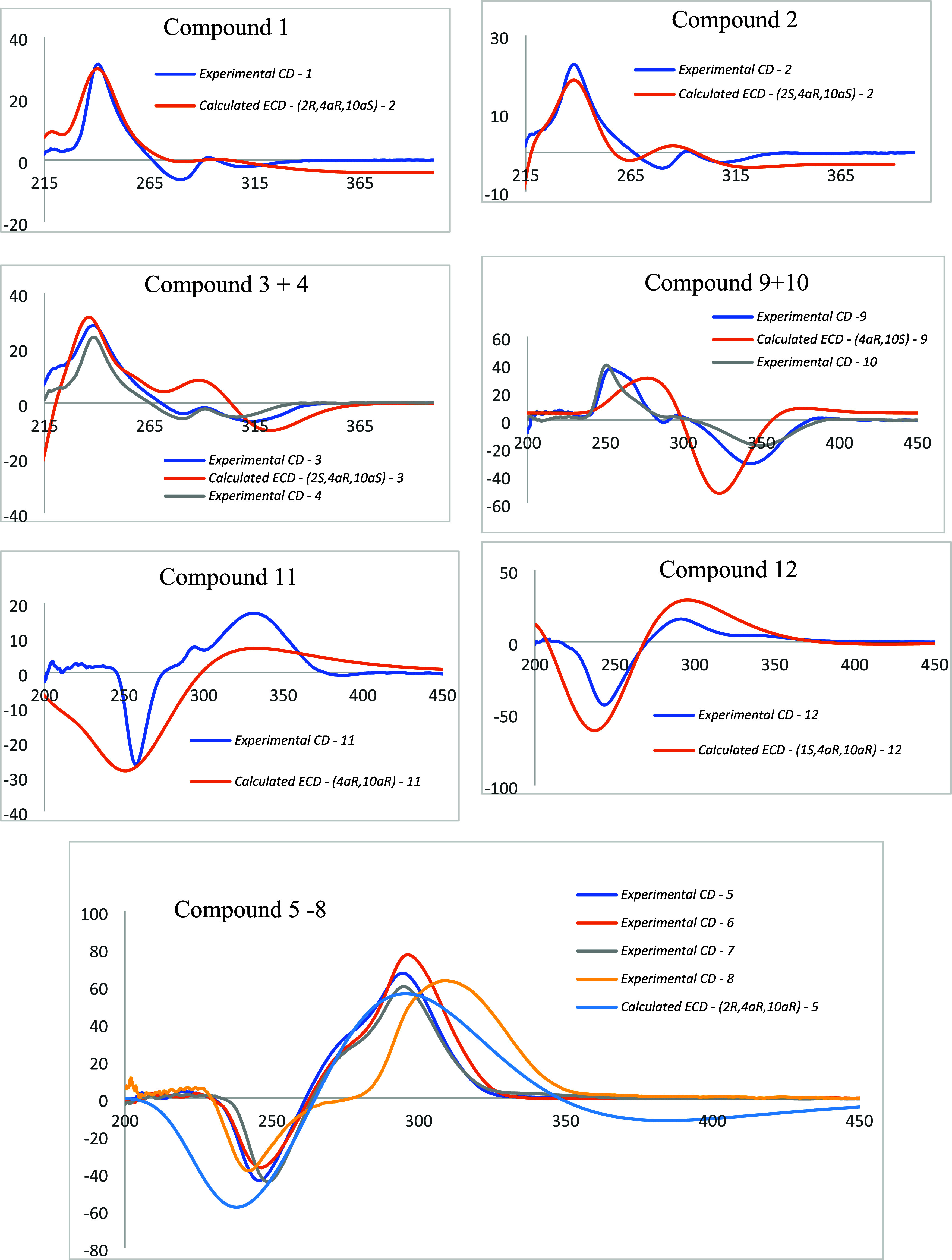
Comparison of experimental CD spectrum and calculated ECD spectrum of compounds **1**–**12**.

In summary, based on UHPLC-MS/MS and MN-guided isolation 15 new and two known glycosidic compounds, namely, elatostemanosides A–Q, were isolated from the aerial parts of *E. tenuicaudatum*. Screening for DPP-IV inhibition revealed a mild activity of the crude extract. Among the isolated compounds, elatostemanoside N exhibited moderate inhibition of DPP-IV, whereas elatostemanosides E, F, and H exhibited weak effects. Mechanisms underlying the binding of these compounds to DPP-IV were explored using molecular docking models. Our results demonstrated a strong correlation between in silico predictions and experimental outcomes, in both the MN analysis and docking studies. A cell viability assay confirmed the safety of the pure compounds in liver cells. Furthermore, three major compounds (elatostemanosides I, K, and N) and one minor compound (elatostemanoside A) demonstrated significant hepatoprotective effects against acetaminophene-induced hepatotoxicity in the HepG2 model. These findings suggest that the aerial part of *E. tenuicaudatum* is not only safe as a vegetable but also potentially beneficial for individuals with hyperglycemia and chronic liver disease (Figure 9).

## Supporting Information Available

Spectroscopic data of isolated compounds; materials and methods; elucidate the structure of compounds **13**, **14**; ECD calculation; UHPLC-MS data for sugar identification; DP4+ analysis data of compound 8 (PDF)

## Abbreviations used

MN: molecular networking
FBMN: feature-based molecular networking
GNPS: global natural product social molecular networking
UHPLC: ultra-high-performance liquid chromatography
MS: mass spectrometry
NMR: nuclear magnetic resonance
UV: ultraviolet
IR: infrared spectroscopy
CD: circular dichroism
ECD: electronic circular dichroism
APAP: acetaminophen (*N*-acetyl-*p*-aminophenol)
DPP-IV: dipeptidyl peptidase 4
