# Supplementary material for: New Hydrogenated Phenanthrene Glycosides from the Edible Vegetable Elatostema tenuicaudatum W.T.Wang with DPP-IV Inhibitory and Hepatoprotective Activity
Source: J Agric Food Chem. 2025 Jan 6;73(2):1273–92. doi: 10.1021/acs.jafc.4c08713 (PMC11741115; doi:10.1021/acs.jafc.4c08713)
Supplement: Supplementary file 1 — jf4c08713_si_001.pdf [file jf4c08713_si_001.pdf]

Supporting information

New Hydrogenated Phenanthrene Glycosides from the Edible Vegetable *Elatostema tenuicaudatum* W.T.Wang with DPP-IV Inhibitory and Hepatoprotective Activity

Quoc-Dung Tran Huynh [a,b], Su-Jung Hsu [c], Truc-Ly Thi Duong [d], Hui-Kang Liu [a,e], Ta-Wei Liu [c], Man-Hsiu Chu [c], Yun-Han Wang [a], Dang-Khoa Nguyen [c,f], Thuy-Tien Thi Phan [b,g], Nguyen-Khanh Huynh Tran [h], Thanh-Hoa Vo [h,i], Hsiao-Yang Hsi [k], Tz-Wei Yeh [k], Ching-Kuo Lee [a,c,l,m*]

[a] Ph.D. Program in Clinical Drug Development of Herbal Medicine, College of Pharmacy, Taipei Medical University, Taipei 11031, Taiwan

[b] Institute of Pharmaceutical Education and Research, Binh Duong University, Thu Dau Mot 820000, Binh Duong, Vietnam

[c] School of Pharmacy, College of Pharmacy, Taipei Medical University, Taipei 11042, Taiwan

[d] Faculty of Traditional medicine, Can Tho University of Medicine and Pharmacy, Can Tho 900000, Vietnam

[e] National Research Institute of Chinese Medicine, Ministry of Health and Welfare, Taipei 11221, Taiwan

[f] Faculty of Pharmacy, Ton Duc Thang University, Ho Chi Minh 700000, Vietnam

[g] Graduate Institute of Biomedical Materials and Tissue Engineering, College of Biomedical Engineering, Taipei Medical University, Taipei 11031, Taiwan

[h] University of Health Sciences, Vietnam National University Ho Chi Minh City, Ho Chi Minh 700000, Vietnam

[i] Center for Discovery and Development of Healthcare Product, Vietnam National University Ho Chi Minh City, Ho Chi Minh 700000, Vietnam

[k] Institute of Fisheries Science, National Taiwan University, Taipei 106, Taiwan

[l] Graduate Institute of Pharmacognosy, College of Pharmacy, Taipei Medical University, Taipei 11042, Taiwan

[m] Department of Chemistry, Chung Yuan Christian University, Zhongli District, Taoyuan 32023, Taiwan

* Correspondence: cklee@tmu.edu.tw; Tel.: + 886-2-27361661 ext. 6150

1. Spectroscopic data of isolated compounds **1**
Figure S1. ^{1}H-NMR spectrum of compound 1 1
Figure S2. ^{13}C-NMR spectrum of compound 1 1
Figure S3. DEPT NMR spectrum of compound 1 2
Figure S4. HSQC NMR spectrum of compound 1 2
Figure S5. COSY NMR spectrum of compound 1 3
Figure S6. HMBC NMR spectrum of compound 1 3
Figure S7. NOESY NMR spectrum of compound 1 4
Figure S8. HR-ESI-MS spectrum of compound 1 5
Figure S9. UV spectrum of compound 1 7
Figure S10. IR spectrum of compound 1 7
Figure S11. ^{1}H-NMR spectrum of compound 2 8
Figure S12. ^{13}C-NMR spectrum of compound 2 8
Figure S13. DEPT NMR spectrum of compound 2 9
Figure S14. HSQC NMR spectrum of compound 2 9
Figure S15. COSY NMR spectrum of compound 2 10
Figure S16. HMBC NMR spectrum of compound 2 10
Figure S17. NOESY NMR spectrum of compound 2 11
Figure S18. HR-ESI-MS spectrum of compound 2 12
Figure S19. UV spectrum of compound 2 14
Figure S20. IR spectrum of compound 2 14
Figure S21. ^{1}H-NMR spectrum of compound 3 15
Figure S22. ^{13}C-NMR spectrum of compound 3 15
Figure S23. DEPT NMR spectrum of compound 3 15
Figure S24. HSQC NMR spectrum of compound 3 16
Figure S25. COSY NMR spectrum of compound 3 17
Figure S26. HMBC NMR spectrum of compound 3 17
Figure S27. NOESY NMR spectrum of compound 3 18
Figure S28. HR-ESI-MS spectrum of compound 3 19
Figure S29. UV spectrum of compound 3 21
Figure S30. IR spectrum of compound 3 22
Figure S31. ^{1}H-NMR spectrum of compound 4 22
Figure S32. ^{13}C-NMR spectrum of compound 4 23
Figure S33. HSQC NMR spectrum of compound 4 23
Figure S34. HMBC NMR spectrum of compound 4 24
Figure S35. COSY NMR spectrum of compound 4 24
Figure S36. NOESY NMR spectrum of compound 4 25
Figure S37. HR-ESI-MS spectrum of compound 4 26
Figure S38. UV spectrum of compound 4 28
Figure S39. IR spectrum of compound 4 29

Figure S40. ^{1}H-NMR spectrum of compound 5 29
Figure S41. ^{13}C-NMR spectrum of compound 5 30
Figure S42. DEPT NMR spectrum of compound 5 30
Figure S43. HSQC NMR spectrum of compound 5 31
Figure S44. HMBC NMR spectrum of compound 5 31
Figure S45. COSY NMR spectrum of compound 5 32
Figure S46. NOESY NMR spectrum of compound 5 32
Figure S47. HR-ESI-MS spectrum of compound 5 34
Figure S48. UV spectrum of compound 5 35
Figure S49. IR spectrum of compound 5 36
Figure S50. ^{1}H-NMR spectrum of compound 6 36
Figure S51. ^{13}C-NMR spectrum of compound 6 37
Figure S52. DEPT NMR spectrum of compound 6 37
Figure S53. HSQC NMR spectrum of compound 6 38
Figure S54. HMBC NMR spectrum of compound 6 38
Figure S55. COSY NMR spectrum of compound 6 39
Figure S56. NOESY NMR spectrum of compound 6 39
Figure S57. HR-ESI-MS spectrum of compound 6 41
Figure S58. UV spectrum of compound 6 42
Figure S59. IR spectrum of compound 6 42
Figure S60. ^{1}H-NMR spectrum of compound 7 43
Figure S61. ^{13}C-NMR spectrum of compound 7 43
Figure S62. DEPT NMR spectrum of compound 7 44
Figure S63. HSQC NMR spectrum of compound 7 44
Figure S64. HMBC NMR spectrum of compound 7 45
Figure S65. COSY NMR spectrum of compound 7 45
Figure S66. NOESY NMR spectrum of compound 7 46
Figure S67. HR-ESI-MS spectrum of compound 7 47
Figure S68. UV spectrum of compound 7 49
Figure S69. IR spectrum of compound 7 49
Figure S70. ^{1}H-NMR spectrum of compound 8 50
Figure S71. ^{13}C-NMR spectrum of compound 8 50
Figure S72. DEPT NMR spectrum of compound 8 51
Figure S73. HSQC NMR spectrum of compound 8 51
Figure S74. HMBC NMR spectrum of compound 8 52
Figure S75. COSY NMR spectrum of compound 8 52
Figure S76. NOESY NMR spectrum of compound 8 53
Figure S77. HR-ESI-MS spectrum of compound 8 54
Figure S78. UV spectrum of compound 8 57
Figure S79. IR spectrum of compound 8 57

Figure S80. ^{1}H-NMR spectrum of compound 9 58
Figure S81. ^{13}C-NMR spectrum of compound 9 58
Figure S82. DEPT NMR spectrum of compound 9 59
Figure S83. HSQC NMR spectrum of compound 9 59
Figure S84. HMBC NMR spectrum of compound 9 60
Figure S85. COSY NMR spectrum of compound 9 60
Figure S86. NOESY NMR spectrum of compound 9 61
Figure S87. HR-ESI-MS spectrum of compound 9 62
Figure S88. UV spectrum of compound 9 63
Figure S89. IR spectrum of compound 9 64
Figure S90. ^{1}H-NMR spectrum of compound 10 64
Figure S91. ^{13}C-NMR spectrum of compound 10 65
Figure S92. DEPT NMR spectrum of compound 10 65
Figure S93. HSQC NMR spectrum of compound 10 66
Figure S94. HMBC NMR spectrum of compound 10 66
Figure S95. COSY NMR spectrum of compound 10 67
Figure S96. NOESY NMR spectrum of compound 10 67
Figure S97. HR-ESI-MS spectrum of compound 10 69
Figure S98. UV spectrum of compound 10 70
Figure S99. IR spectrum of compound 10 71
Figure S100. ^{1}H-NMR spectrum of compound 11 71
Figure S101. ^{13}C-NMR spectrum of compound 11 72
Figure S102. DEPT NMR spectrum of compound 11 72
Figure S103. HSQC NMR spectrum of compound 11 73
Figure S104. HMBC NMR spectrum of compound 11 73
Figure S105. COSY NMR spectrum of compound 11 74
Figure S106. NOESY NMR spectrum of compound 11 74
Figure S107. HR-ESI-MS spectrum of compound 11 76
Figure S108. UV spectrum of compound 11 77
Figure S109. IR spectrum of compound 11 78
Figure S110. ^{1}H-NMR spectrum of compound 12 78
Figure S111. ^{13}C-NMR spectrum of compound 12 79
Figure S112. DEPT NMR spectrum of compound 12 79
Figure S113. HSQC NMR spectrum of compound 12 80
Figure S114. HMBC NMR spectrum of compound 12 80
Figure S115. COSY NMR spectrum of compound 12 81
Figure S116. NOESY NMR spectrum of compound 12 81
Figure S117. HR-ESI-MS spectrum of compound 12 83
Figure S118. UV spectrum of compound 12 84
Figure S119. IR spectrum of compound 12 85

Figure S120. ^{1}H-NMR spectrum of compound 13........85
Figure S121. ^{13}C-NMR spectrum of compound 13........86
Figure S122. DEPT NMR spectrum of compound 13........86
Figure S123. HSQC NMR spectrum of compound 13........87
Figure S124. HMBC NMR spectrum of compound 13........87
Figure S125. COSY NMR spectrum of compound 13........88
Figure S126. NOESY NMR spectrum of compound 13........88
Figure S127. HR-ESI-MS spectrum of compound 13........89
Figure S128. UV spectrum of compound 13........92
Figure S129. IR spectrum of compound 13........92
Figure S130. ^{1}H-NMR spectrum of compound 14........93
Figure S131. ^{13}C-NMR spectrum of compound 14........93
Figure S132. HSQC NMR spectrum of compound 14........94
Figure S133. HMBC NMR spectrum of compound 14........94
Figure S134. COSY NMR spectrum of compound 14........94
Figure S135. NOESY NMR spectrum of compound 14........95
Figure S136. HR-ESI-MS spectrum of compound 14........96
Figure S137. UV spectrum of compound 14........99
Figure S138. IR spectrum of compound 14........100
Figure S139. ^{1}H-NMR spectrum of compound 15........100
Figure S140. ^{13}C-NMR spectrum of compound 15........101
Figure S141. DEPT NMR spectrum of compound 15........101
Figure S142. HSQC NMR spectrum of compound 15........102
Figure S143. HMBC NMR spectrum of compound 15........102
Figure S144. COSY NMR spectrum of compound 15........103
Figure S145. NOESY NMR spectrum of compound 15........103
Figure S146. HR-ESI-MS spectrum of compound 15........104
Figure S147. UV spectrum of compound 15........107
Figure S148. IR spectrum of compound 15........108
Figure S149. ^{1}H-NMR spectrum of compound 16........108
Figure S150. ^{13}C-NMR spectrum of compound 16........109
Figure S151. DEPT NMR spectrum of compound 16........109
Figure S152. HSQC NMR spectrum of compound 16........110
Figure S153. HMBC NMR spectrum of compound 16........110
Figure S154. COSY NMR spectrum of compound 16........111
Figure S155. NOESY NMR spectrum of compound 16........111
Figure S156. HR-ESI-MS spectrum of compound 16........113
Figure S157. UV spectrum of compound 16........114
Figure S158. IR spectrum of compound 16........115
Figure S159. ^{1}H-NMR spectrum of compound 17........115

Figure S160. ^{13}C-NMR spectrum of compound 17 116
Figure S161. DEPT NMR spectrum of compound 17 116
Figure S162. HSQC NMR spectrum of compound 17 117
Figure S163. HMBC NMR spectrum of compound 17 117
Figure S164. COSY NMR spectrum of compound 17 118
Figure S165. NOESY NMR spectrum of compound 17 118
Figure S166. HR-ESI-MS spectrum of compound 17 120
Figure S167. UV spectrum of compound 17 121
Figure S168. IR spectrum of compound 17 121
Figure S169. Key NOESY correlation for distinguishing (4a*R*,10a*R*) form and (4a*S*,10a*S*) form of compound 5 122
Figure S170. Key NOESY correlation for distinguishing (4a*R*,10a*R*) form and (4a*S*,10a*S*) form of compounds 11 – 12 122
2. MATERIALS AND METHODS 124
3. ELUCIDATE THE STRUCTURE OF COMPOUND 13, 14 132
4. ECD CALCULATION DATA OF COMPOUND 1 133
Figure S171. The energies and equilibrium populations of stable conformers 1_1 to 1_5 133
Figure S172. Cartesian Coordinates of low energy conformers 1_1 to 1_5 134
5. ECD CALCULATION DATA OF COMPOUND 2 138
Figure S173. The energies and equilibrium populations of stable conformers 2_1, 2_2 138
Figure S174. Cartesian Coordinates of low energy conformers 2_1, 2_2 139
6. ECD CALCULATION DATA OF COMPOUND 3 141
Figure S175. The energies and equilibrium populations of stable conformers 3_1 - 3_3 141
Figure S176. Cartesian Coordinates of low energy conformers 3_1 - 3_3 142
7. ECD CALCULATION DATA OF COMPOUND 5 145
Figure S177. The energies and equilibrium populations of stable conformers 5_1 - 5.6 145
Figure S178. Cartesian Coordinates of low energy conformers 5_1 - 5_6 146
8. ECD CALCULATION DATA OF COMPOUND 9 151
Figure S179. The energies and equilibrium populations of stable conformers 9_1 - 3_7 151
Figure S180. Cartesian Coordinates of low energy conformers 9_1 - 9_7 153
9. ECD CALCULATION DATA OF COMPOUND 11 158
Figure S181. The energies and equilibrium populations of stable conformers 11_1 - 11_9 158
Figure S182. Cartesian Coordinates of low energy conformers 11_1 - 11_9 160
10. ECD CALCULATION DATA OF COMPOUND 12 167
Figure S183. The energies and equilibrium populations of stable conformers 12_1 - 12_8 167
Figure S184. Cartesian Coordinates of low energy conformers 12_1 - 12_8 169
11. UHPLC-MS DATA FOR SUGAR IDENTIFICATION 175
Figure S185. UHPLC-MS of D-Glc-NAIM (4.92 min) and L-Rha-NAIM (7.16 min) 175
Figure S186. UHPLC-MS of compound **1**'s sugar - NAIM 175
Figure S187. UHPLC-MS of compound **2**'s sugar - NAIM 175
Figure S188. UHPLC-MS of compound **3**'s sugar - NAIM 176

Figure S189. UHPLC-MS of compound **4**'s sugar - NAIM 176
Figure S190. UHPLC-MS of compound **5**'s sugar - NAIM 176
Figure S191. UHPLC-MS of compound **6**'s sugar - NAIM 177
Figure S192. UHPLC-MS of compound **7**'s sugar - NAIM 177
Figure S193. UHPLC-MS of compound **8**'s sugar - NAIM 177
Figure S194. UHPLC-MS of compound **9**'s sugar - NAIM 178
Figure S195. UHPLC-MS of compound **10**'s sugar - NAIM 178
Figure S196. UHPLC-MS of compound **11**'s sugar – NAIM 178
Figure S197. UHPLC-MS of compound **12**'s sugar – NAIM 179
Figure S198. UHPLC-MS of compound **13**'s sugar – NAIM 179
Figure S199. UHPLC-MS of compound **14**'s sugar – NAIM 179
Figure S200. UHPLC-MS of compound **15**'s sugar – NAIM 180
Figure S201. UHPLC-MS of compound **16**'s sugar – NAIM 180
Figure S202. UHPLC-MS of compound **17**'s sugar – NAIM 180
12. DP4+ ANALYSIS DATA OF COMPOUND 8 181
Figure S203. The energies and equilibrium populations of the stable conformers of the (3*R*)-**8** and (3*S*)-**8** configurations 181
Figure S204. Cartesian coordinates of low energy conformers (3*R*)-**8**-1 to (3*R*)-**8**-3 and (3*S*)-**8**-1 to (3S)-**8**-5 183
Figure S205. DP4+ probability distribution for the stereoisomers (3*R*)-**8** and (3*S*)-**8** 189

1. Spectroscopic data of isolated compounds

Figure S1. ^{1}H-NMR spectrum of compound 1

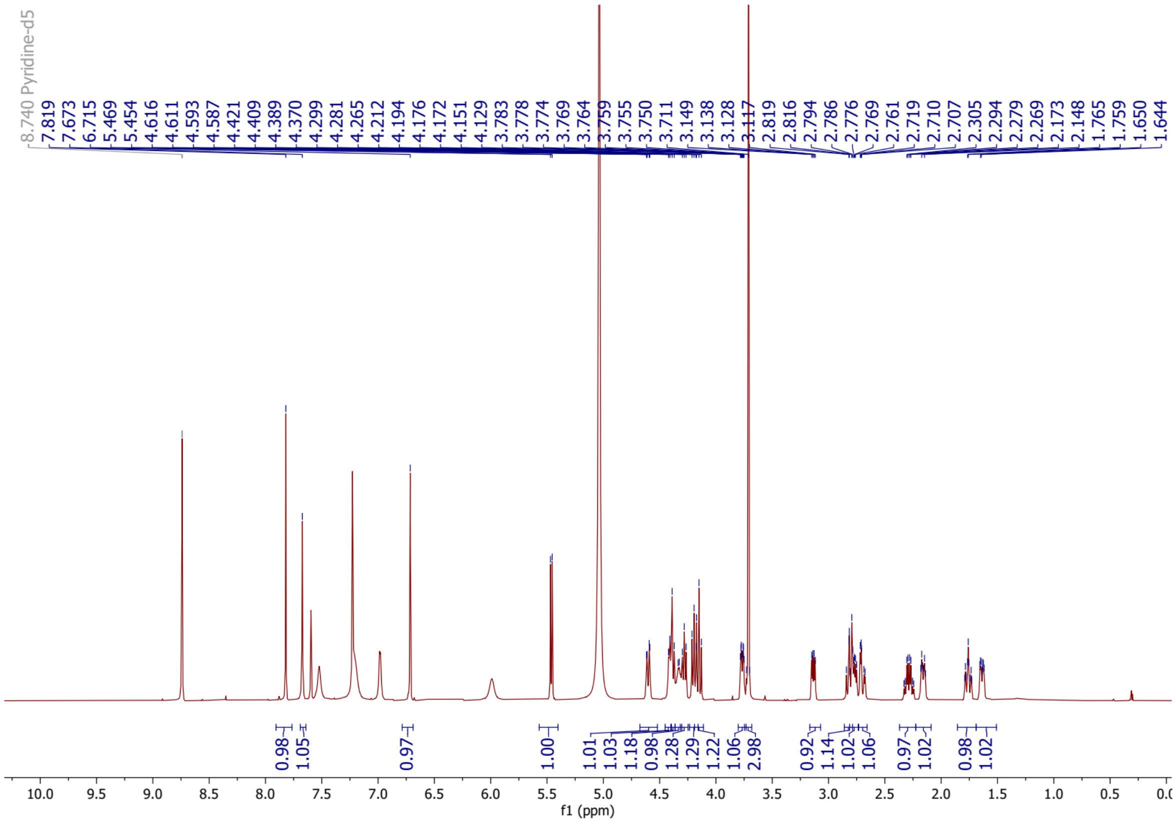

Figure S2. ^{13}C-NMR spectrum of compound 1

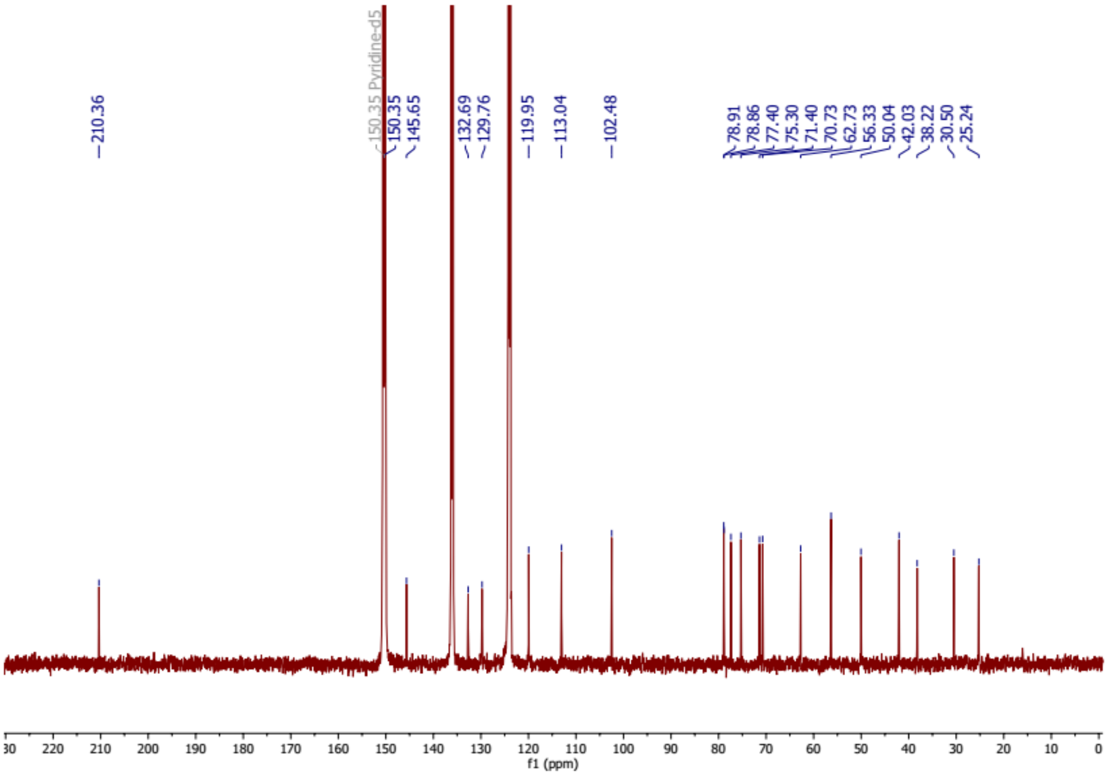

Figure S3. DEPT NMR spectrum of compound 1

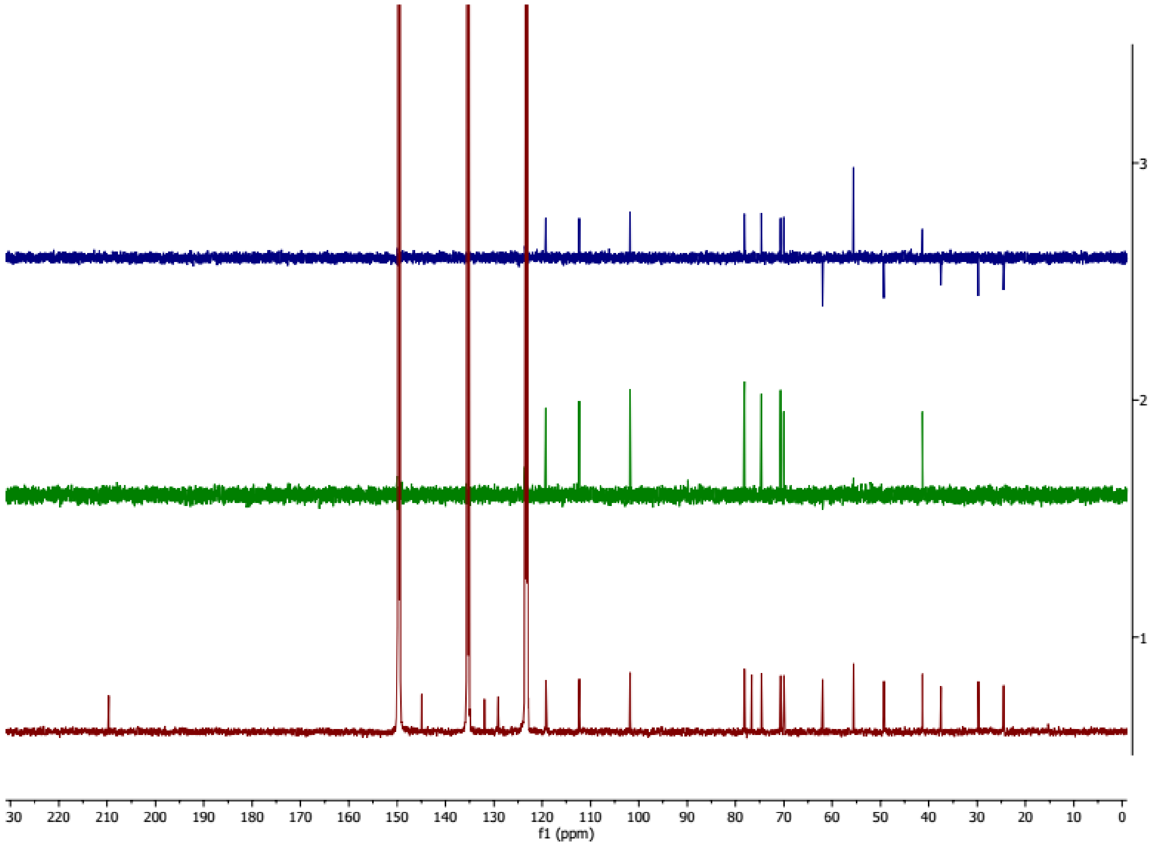

Figure S4. HSQC NMR spectrum of compound 1

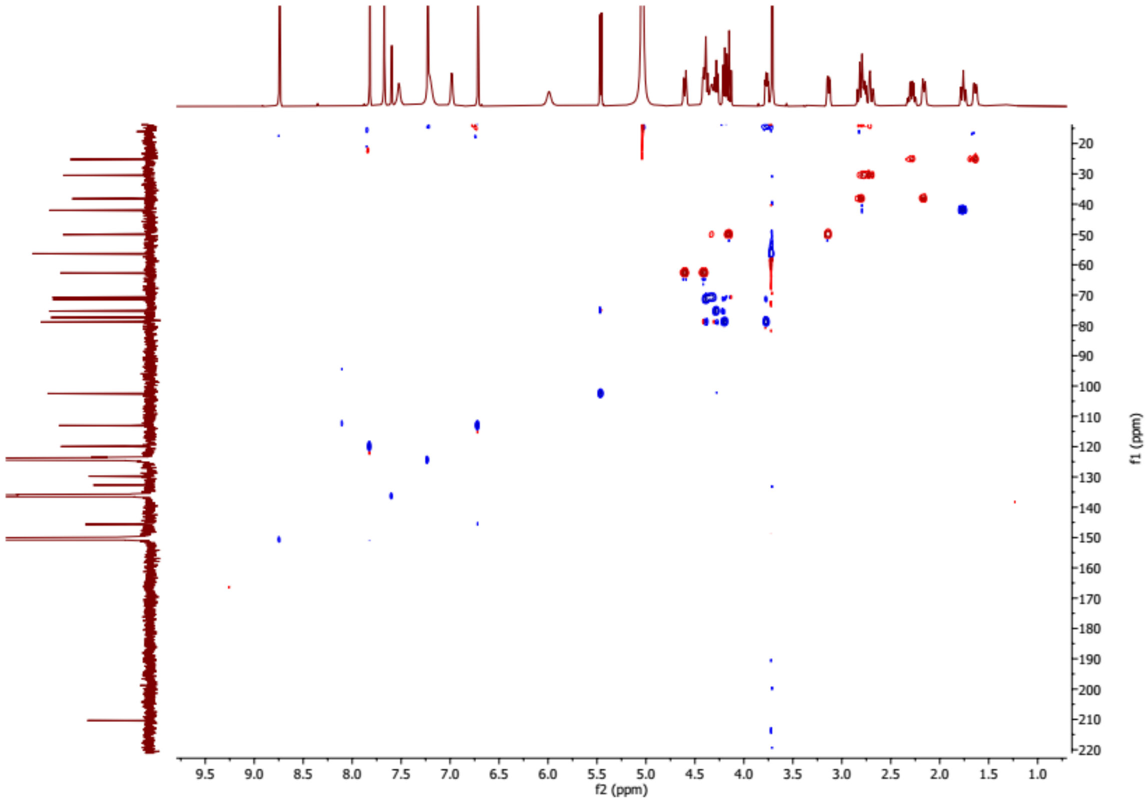

Figure S5. COSY NMR spectrum of compound 1

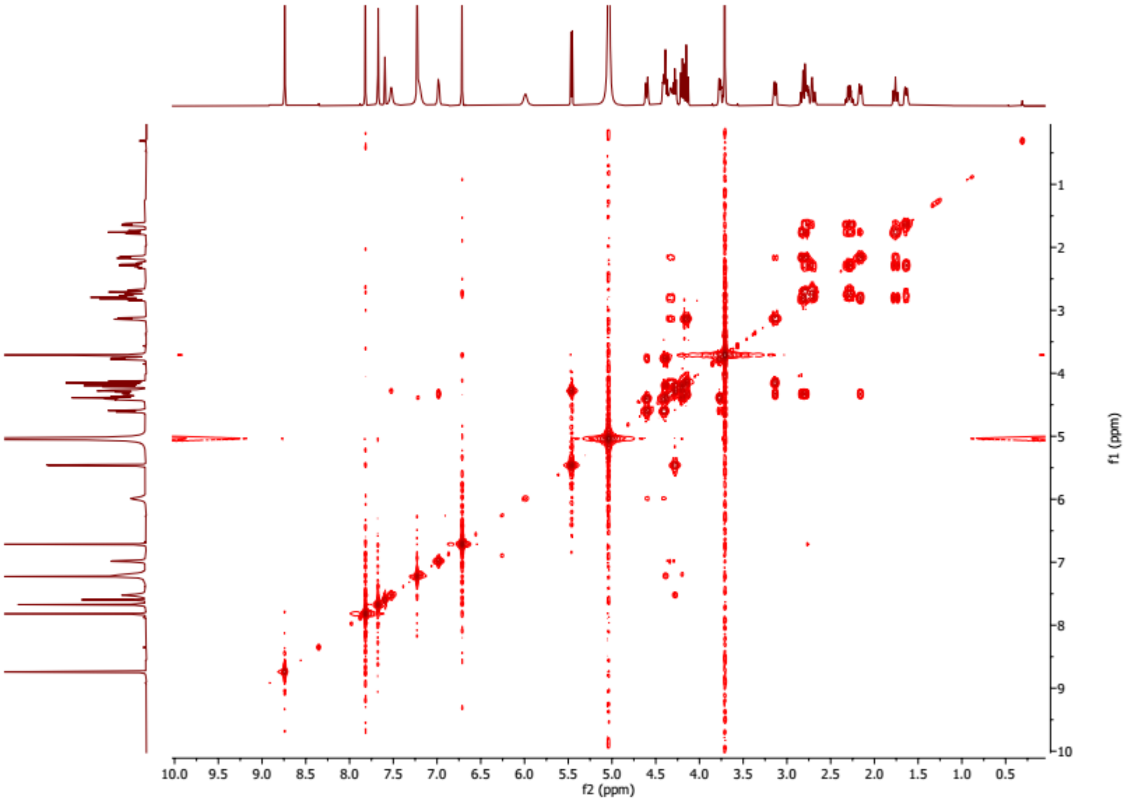

Figure S6. HMBC NMR spectrum of compound 1

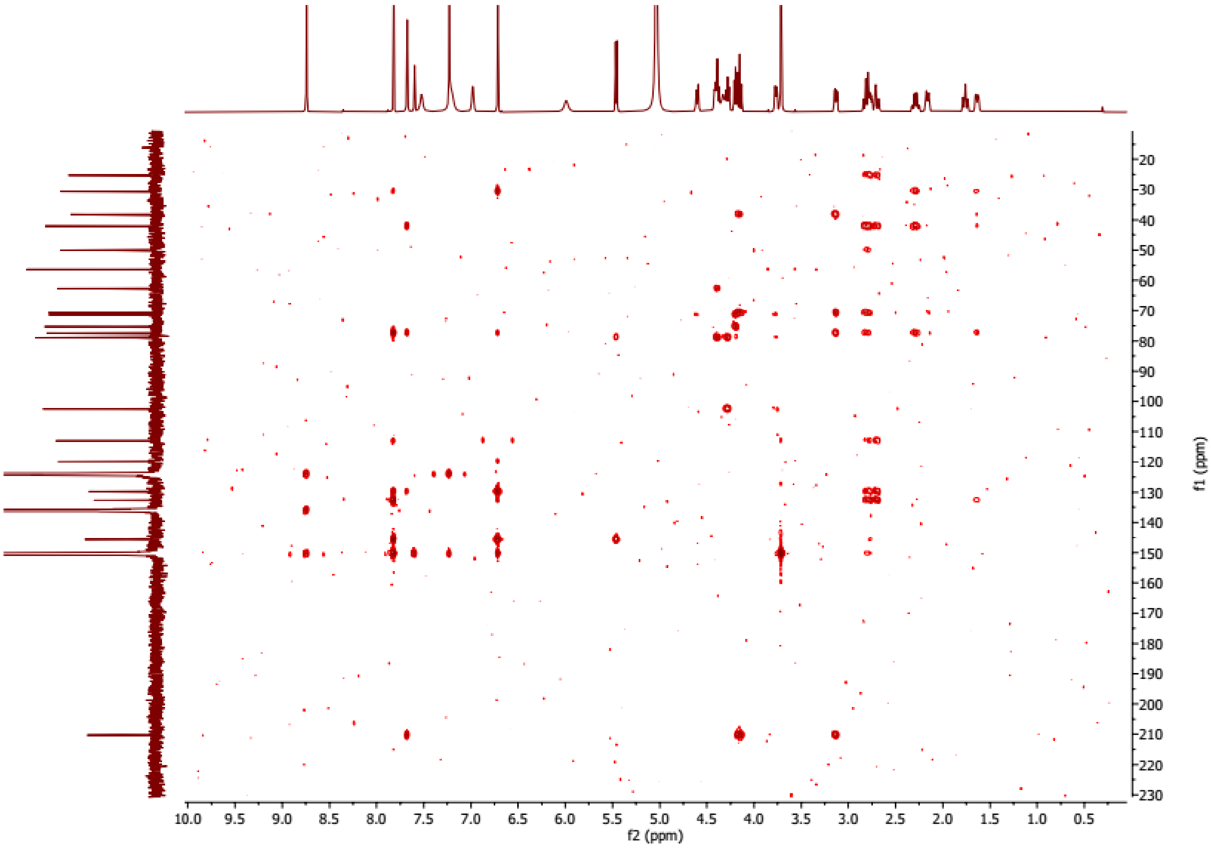

Figure S7. NOESY NMR spectrum of compound 1

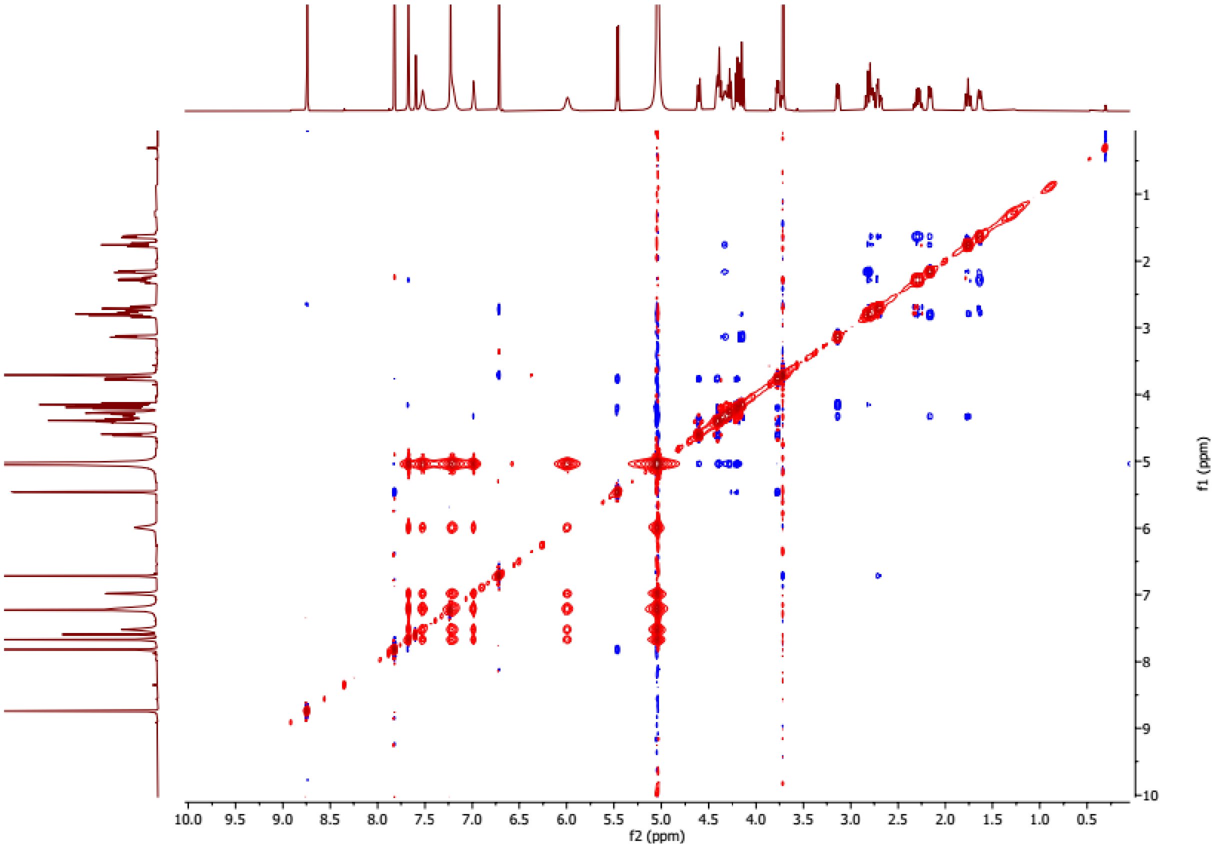

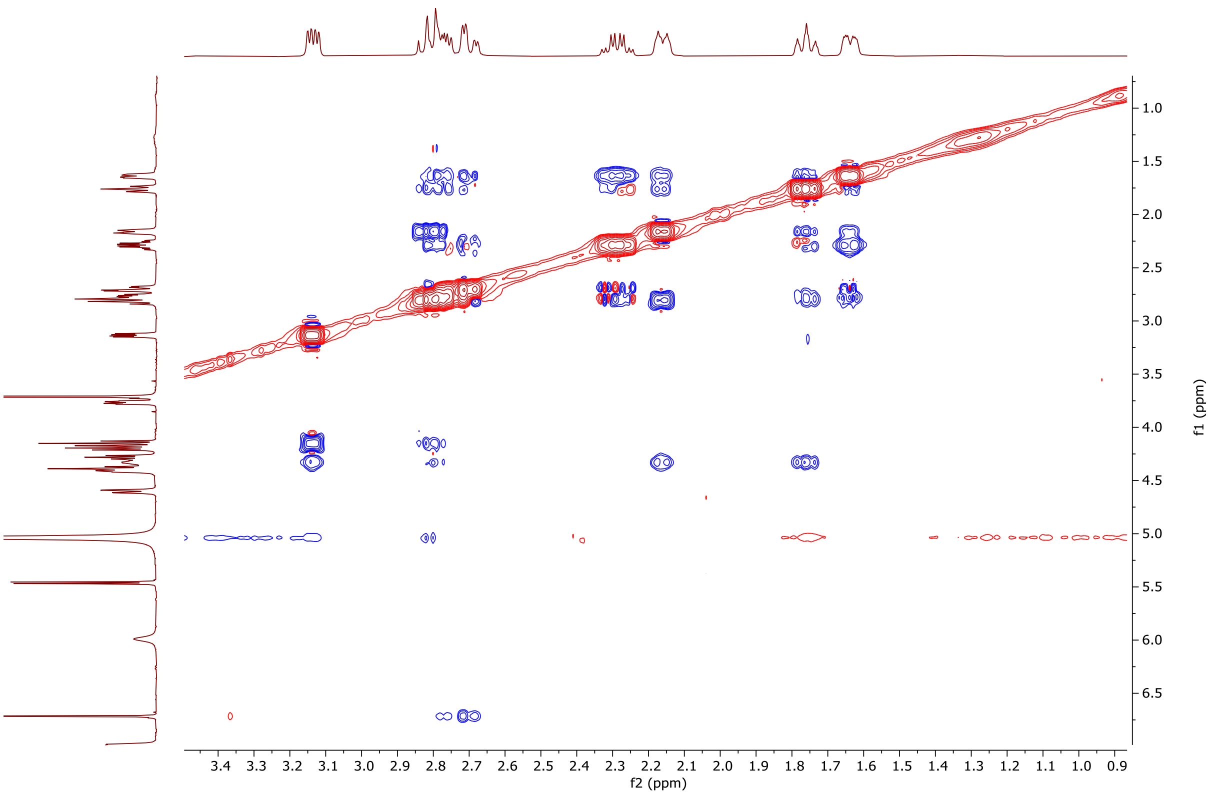

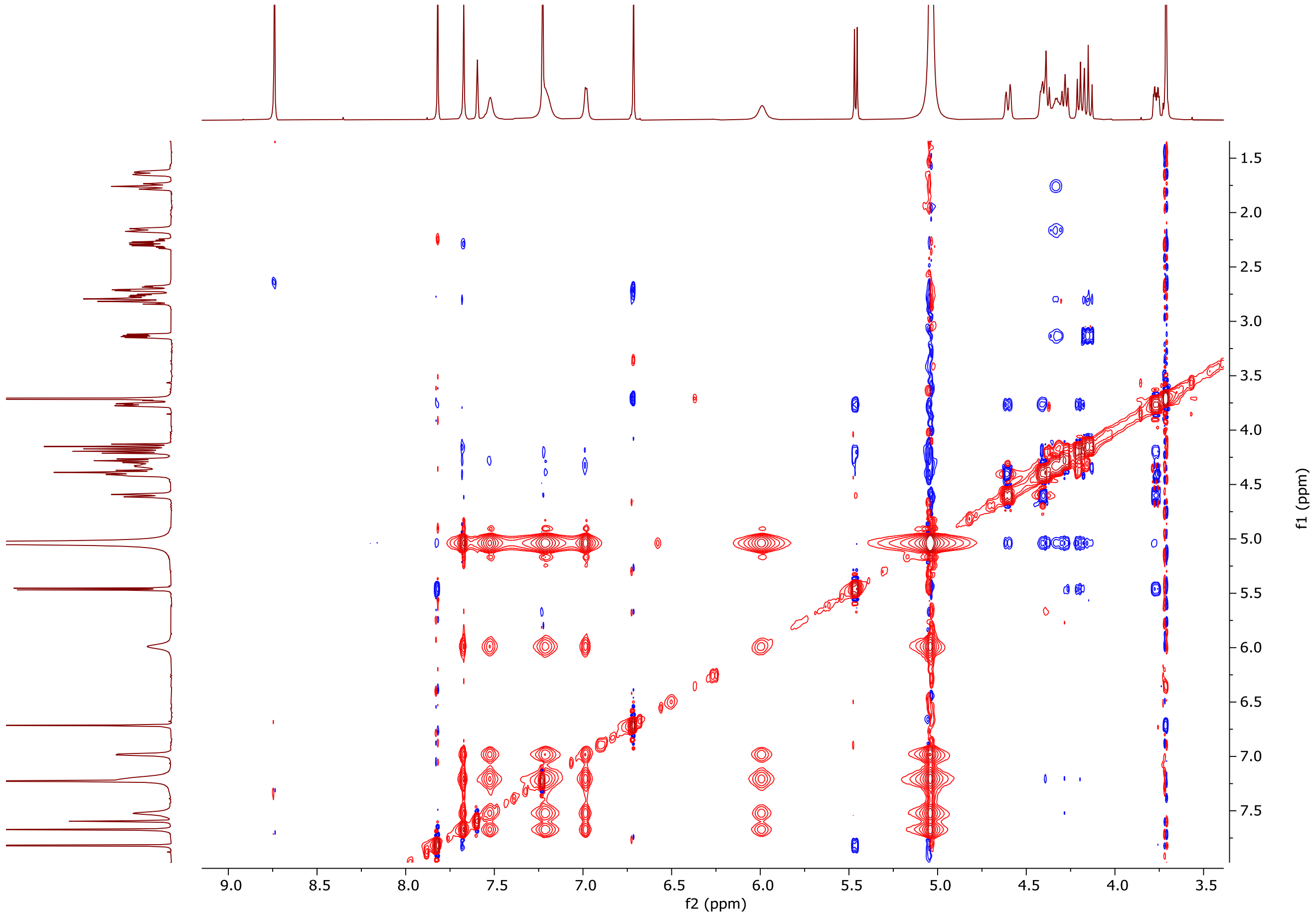

Figure S8. HR-ESI-MS spectrum of compound 1

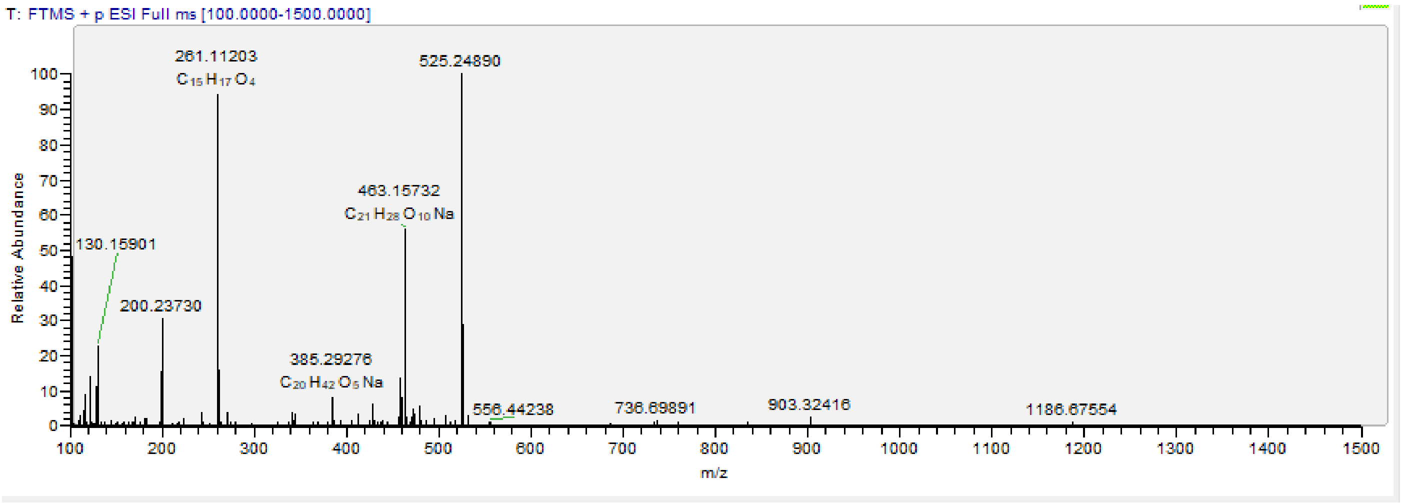

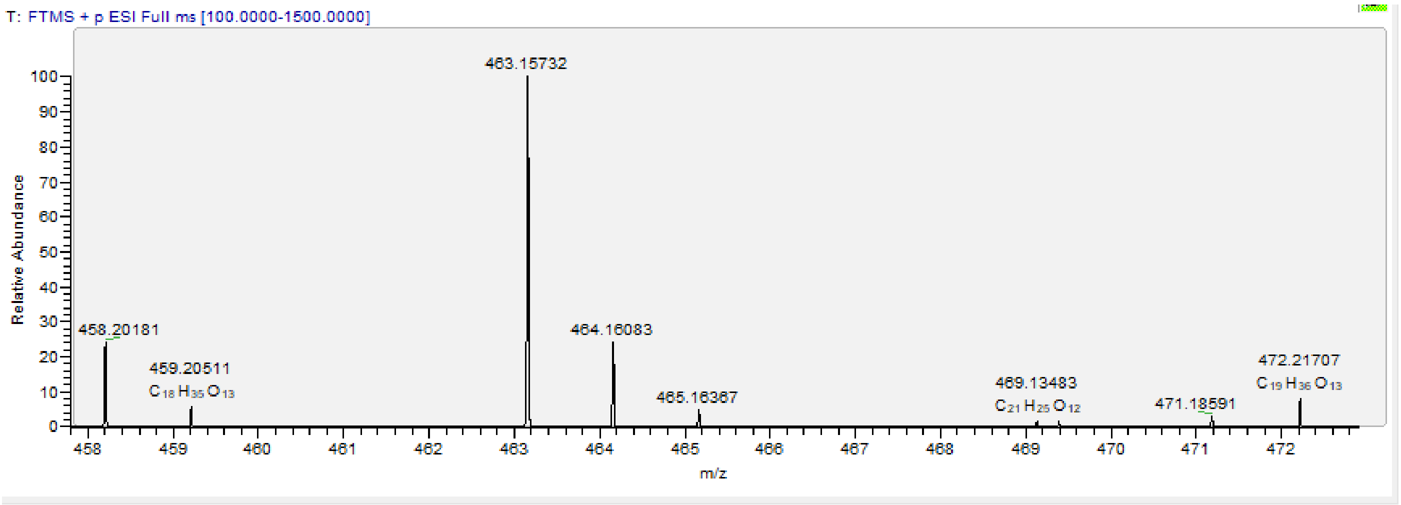

Elemental composition

Single mass

Mass: 463.15732

Max. results 10

Calculate

Idx	Formula	RDB	Delta ppm
1	$C_{21}H_{28}O_{10}Na$	7.5	-0.320

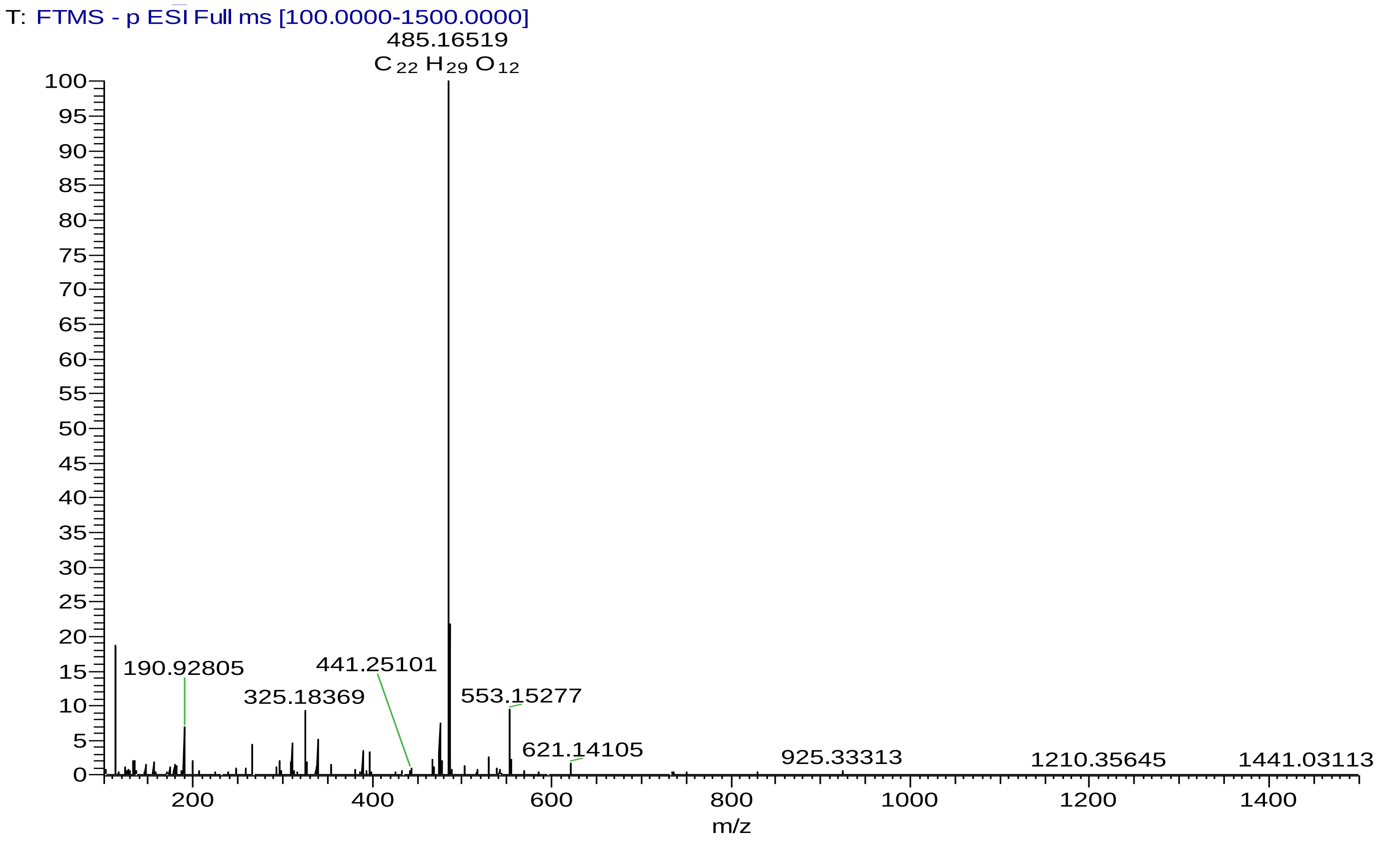

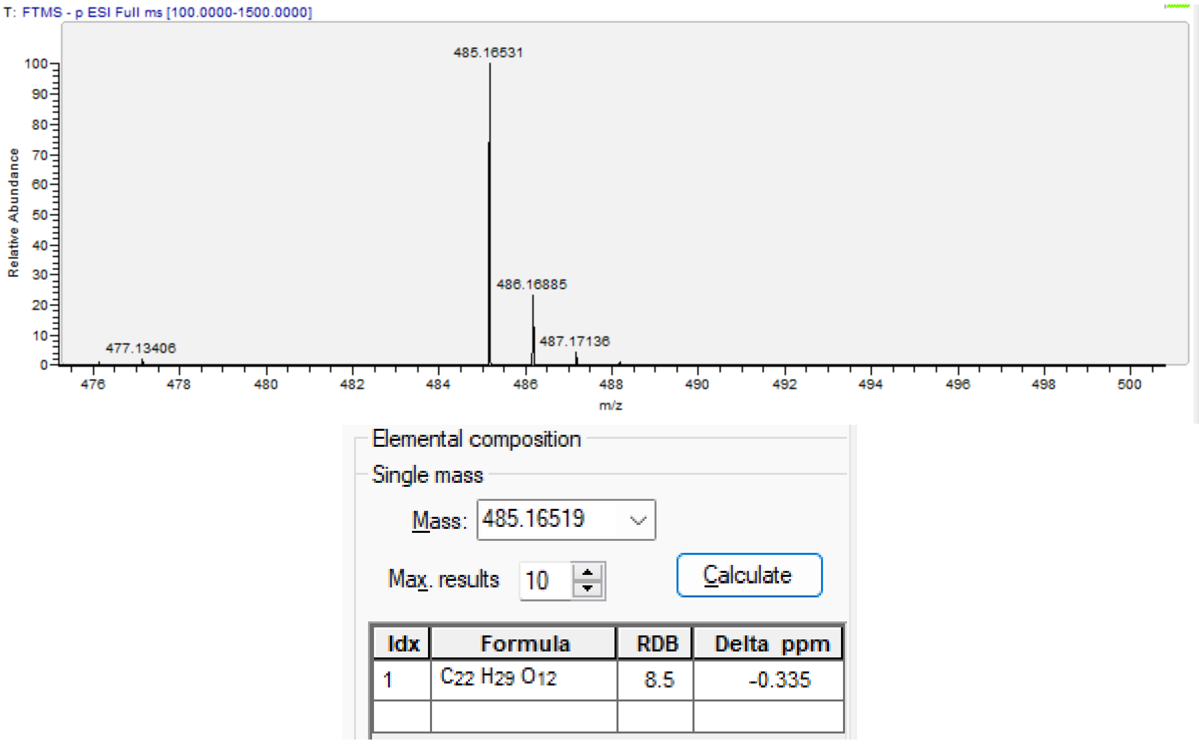

Figure S9. UV spectrum of compound 1

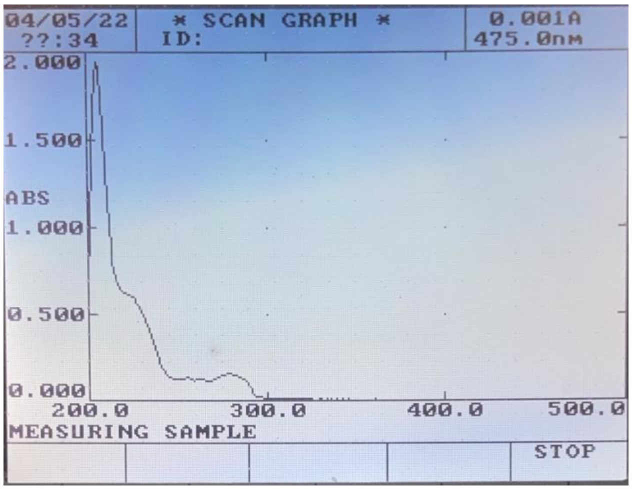

Figure S10. IR spectrum of compound 1

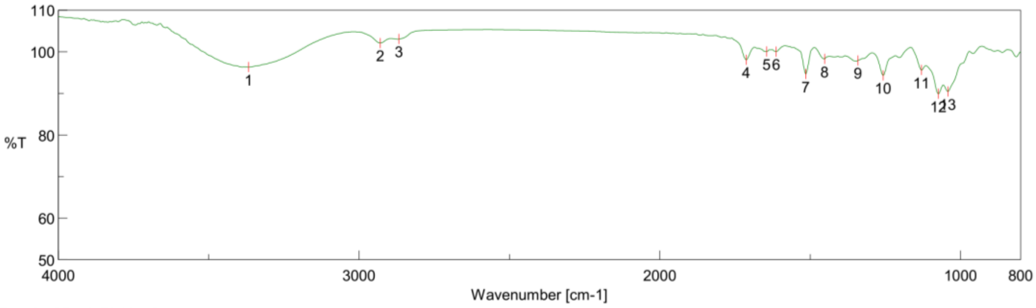

[Result of Peak Picking]

No.	Position	Intensity	No.	Position	Intensity	No.	Position	Intensity
1	3367.1	96.3307	2	2929.34	102.084	3	2867.63	103.052
4	1712.48	98.0778	5	1644.98	100.055	6	1613.16	100.036
7	1514.81	94.6578	8	1452.14	98.2864	9	1341.25	97.8817
10	1257.36	94.3248	11	1130.08	95.6054	12	1073.19	89.8965
13	1042.34	90.455	14	585.29	95.4618			

Figure S11. ^{1}H-NMR spectrum of compound 2

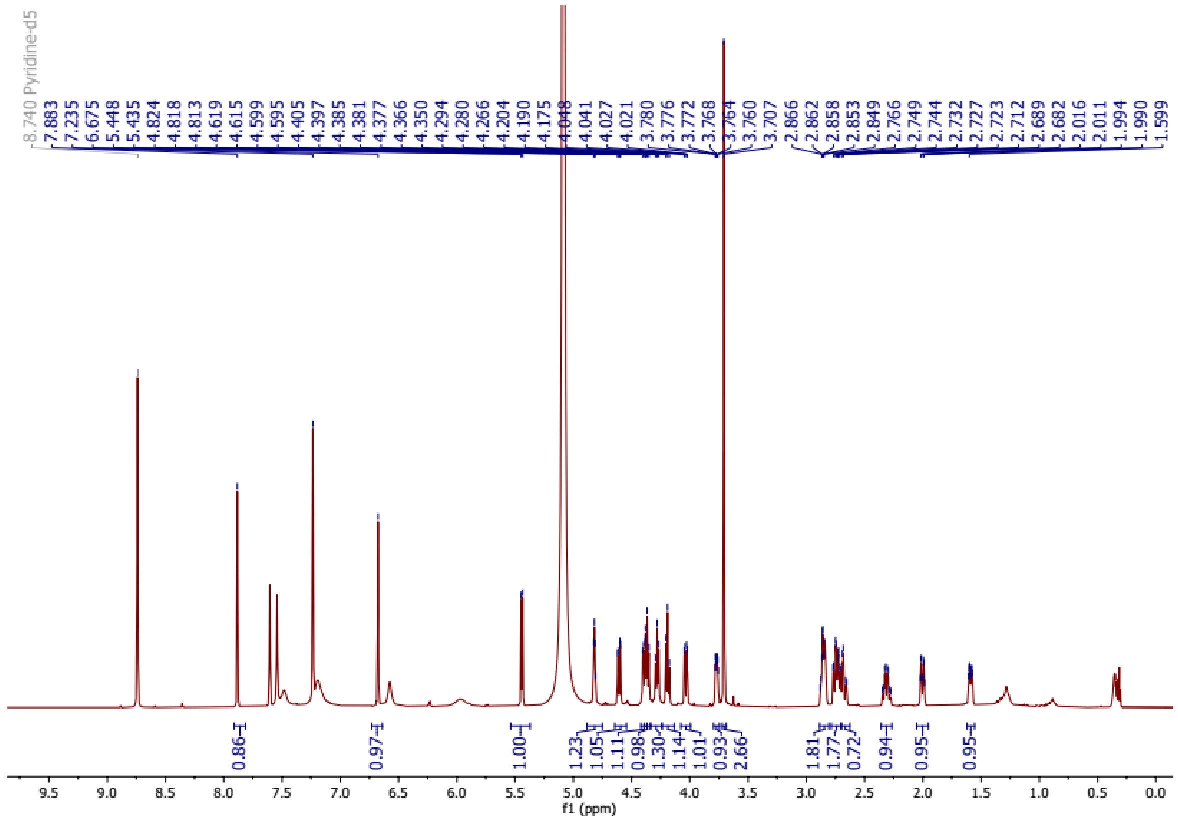

Figure S12. ^{13}C-NMR spectrum of compound 2

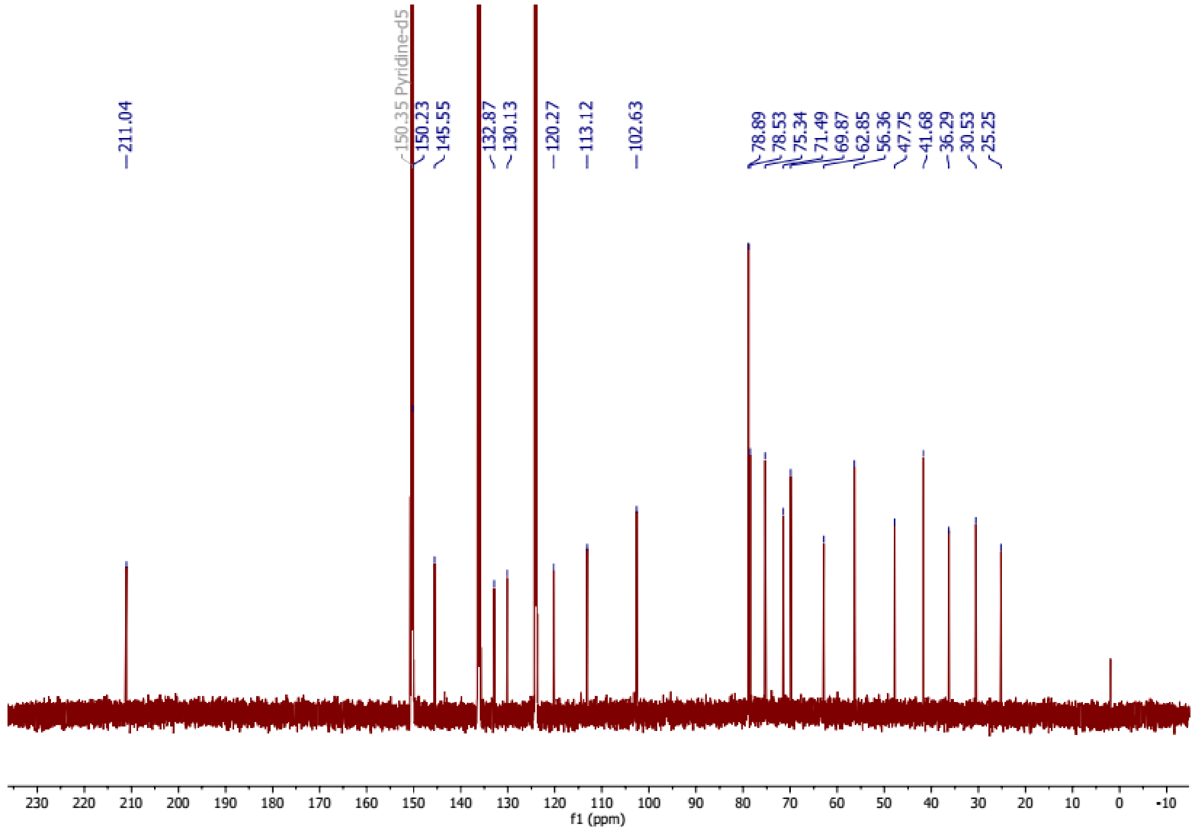

Figure S13. DEPT NMR spectrum of compound 2

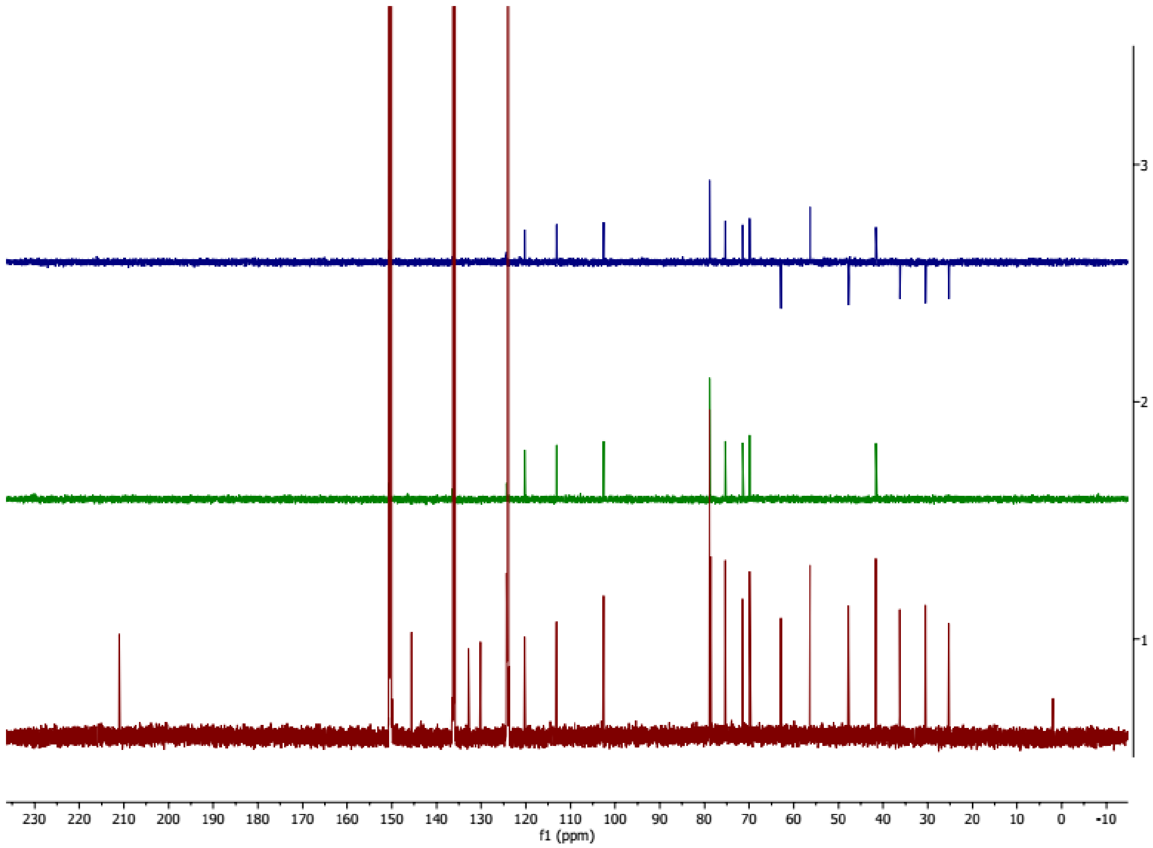

Figure S14. HSQC NMR spectrum of compound 2

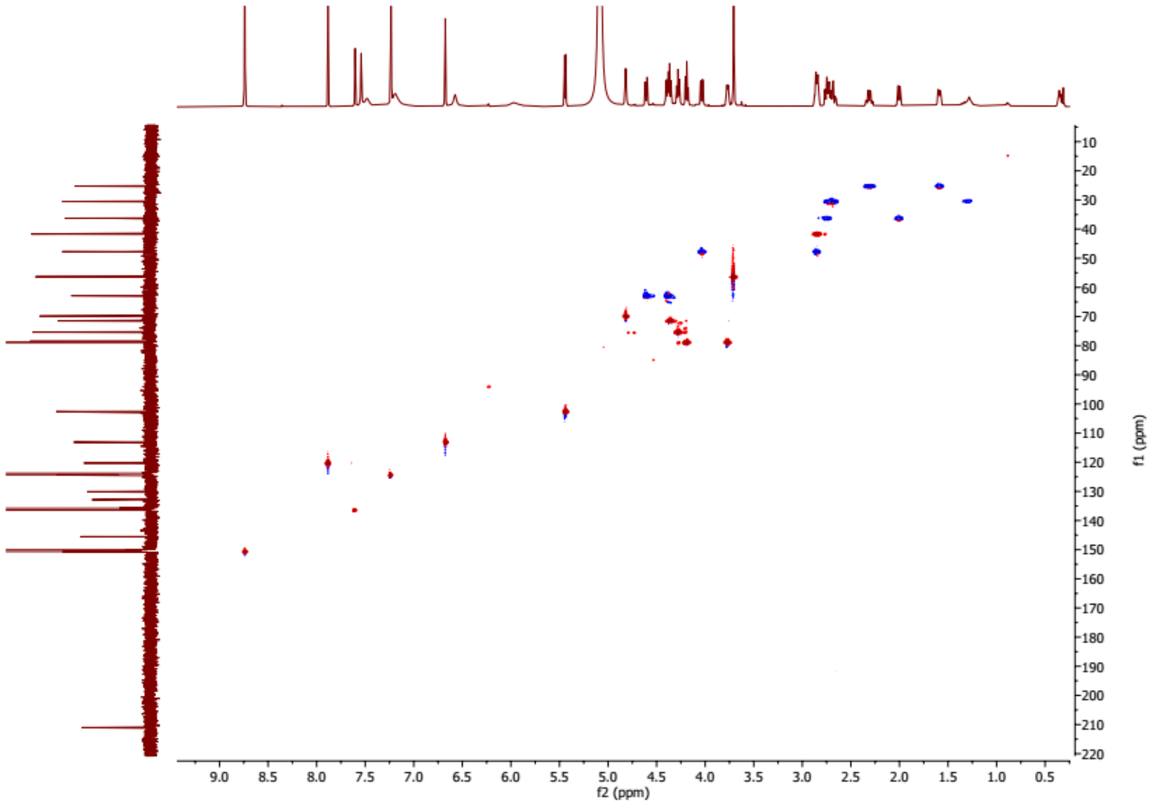

Figure S15. COSY NMR spectrum of compound 2

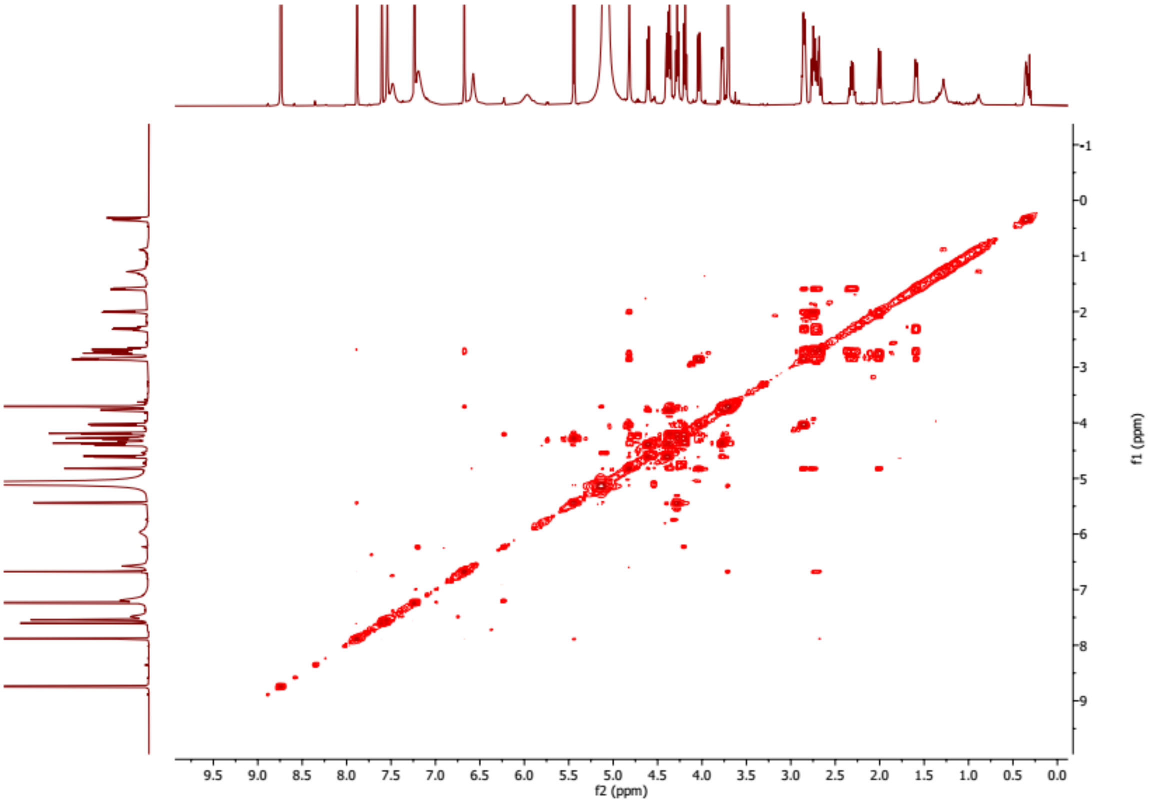

Figure S16. HMBC NMR spectrum of compound 2

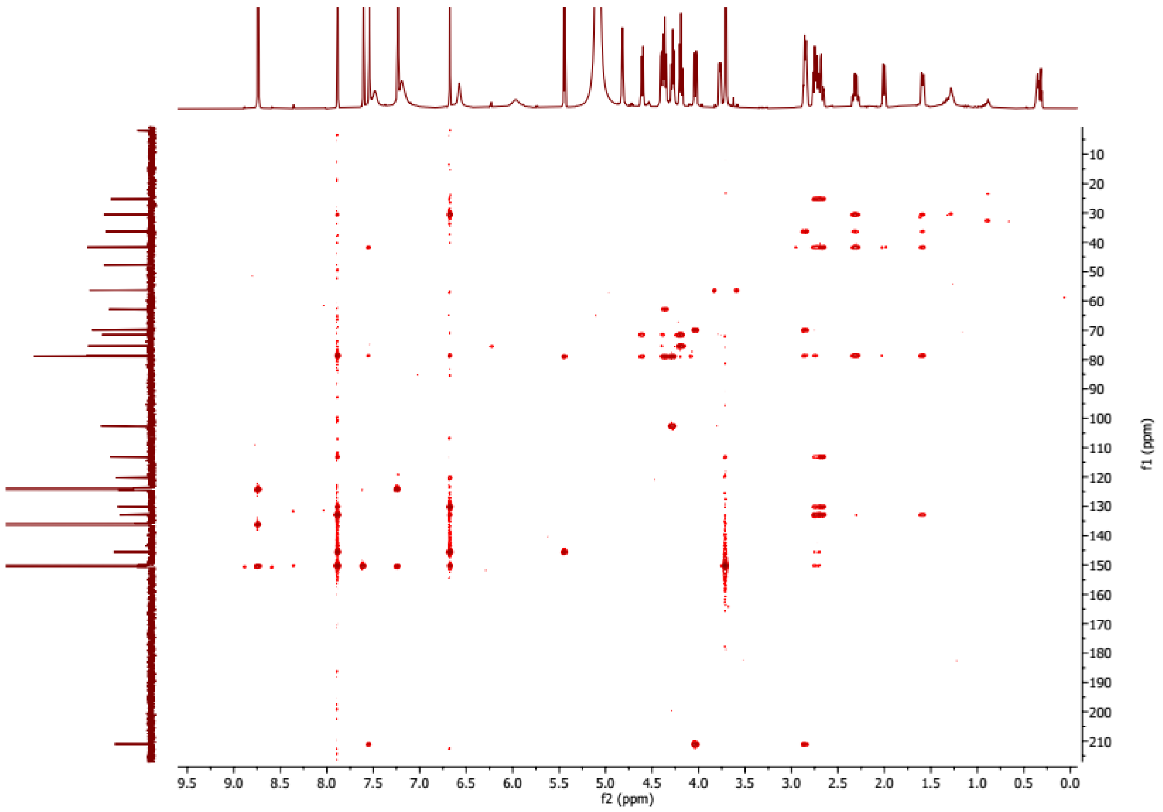

Figure S17. NOESY NMR spectrum of compound 2

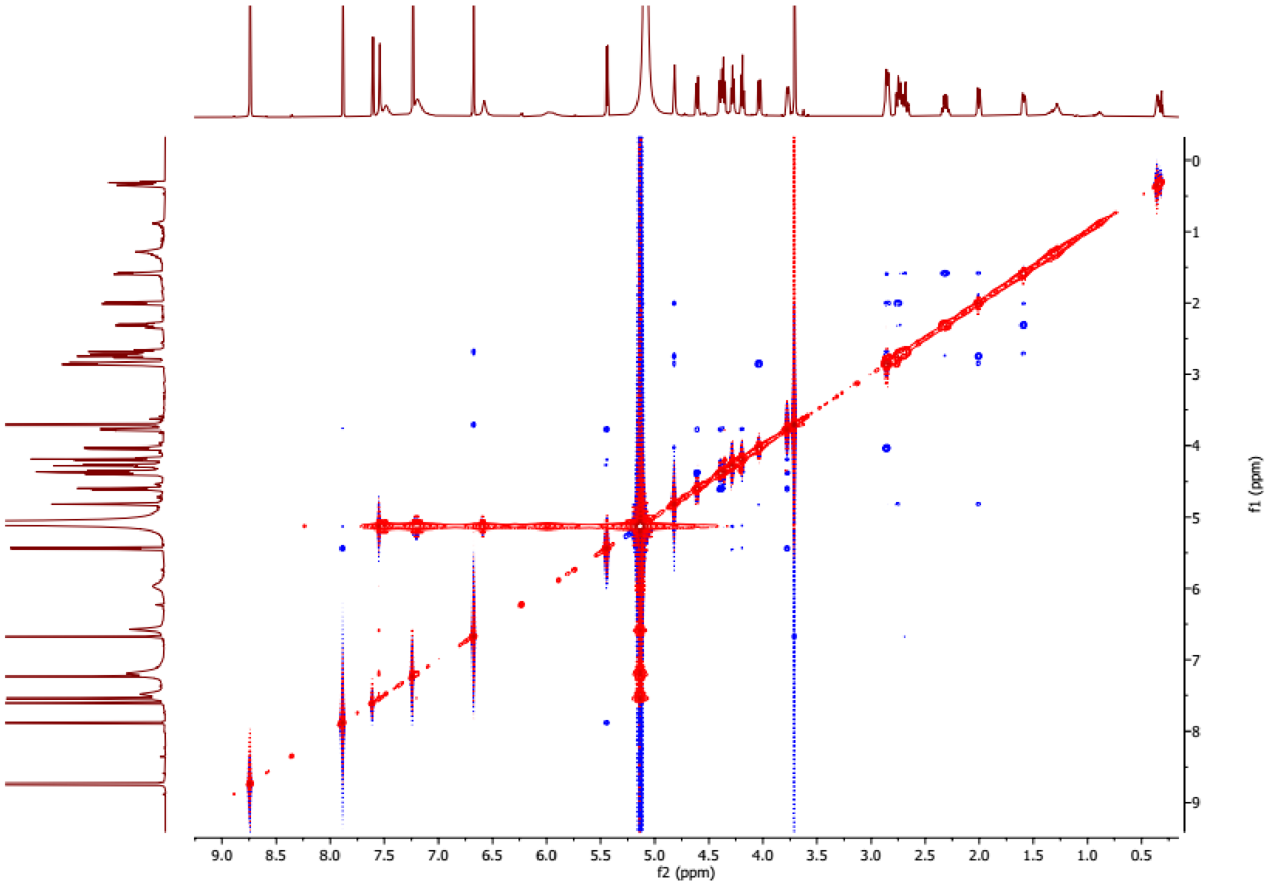

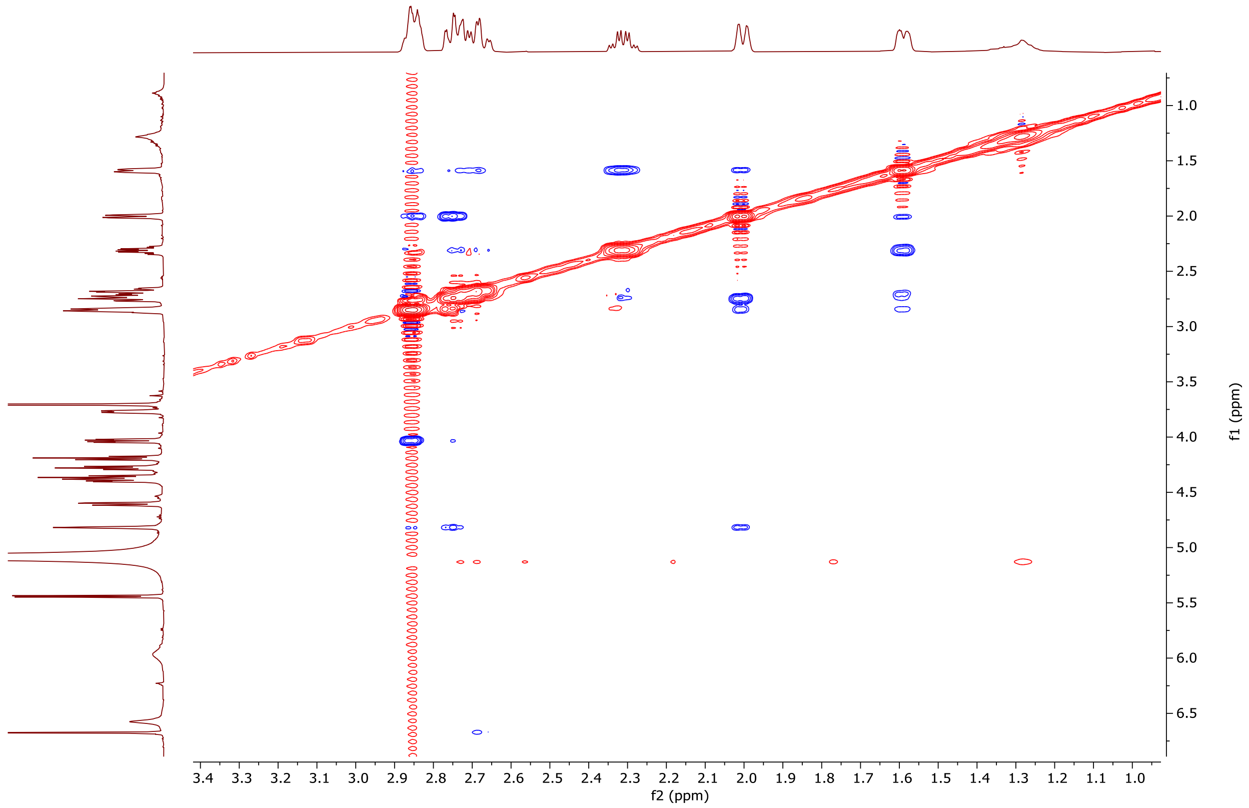

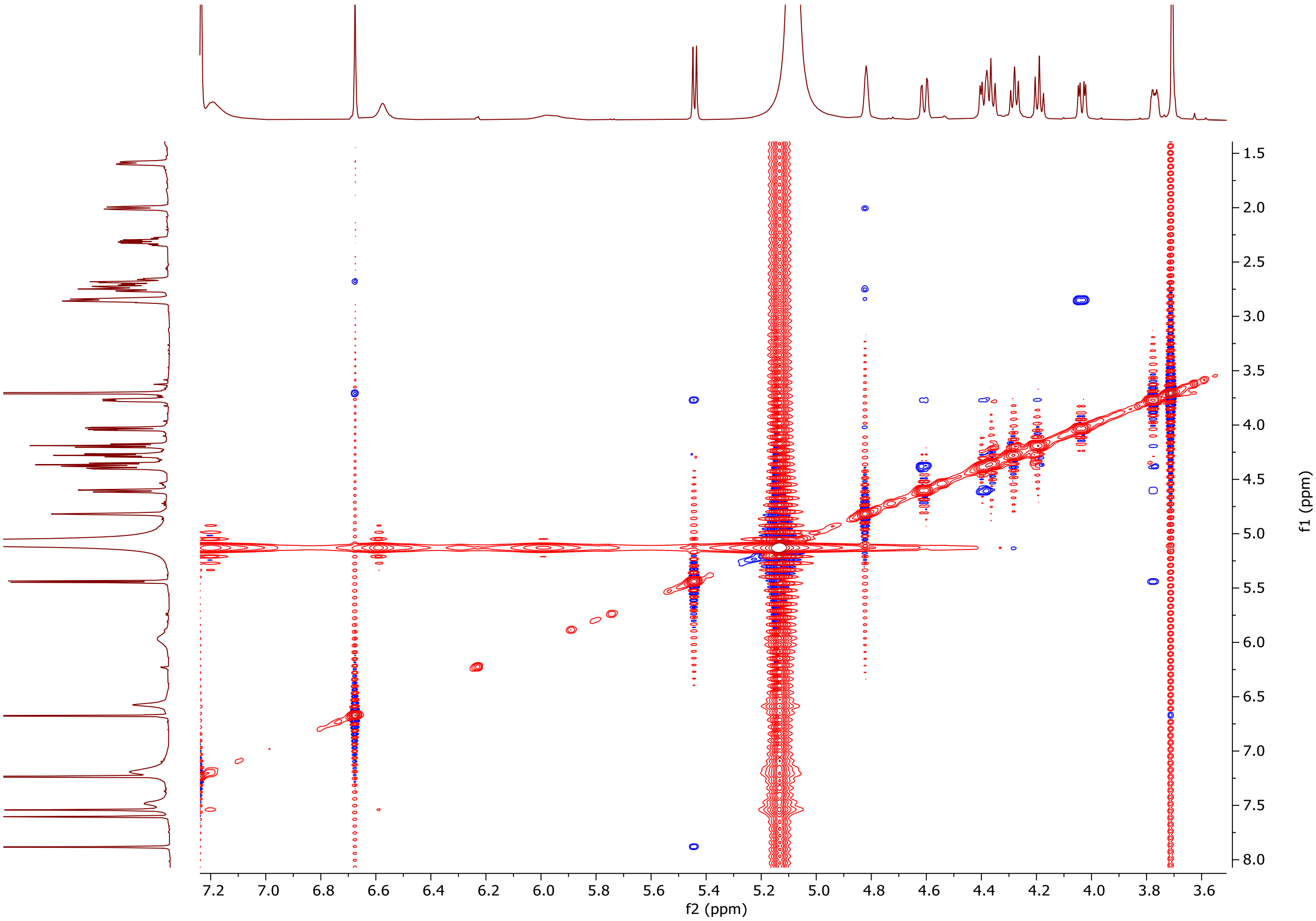

Figure S18. HR-ESI-MS spectrum of compound 2

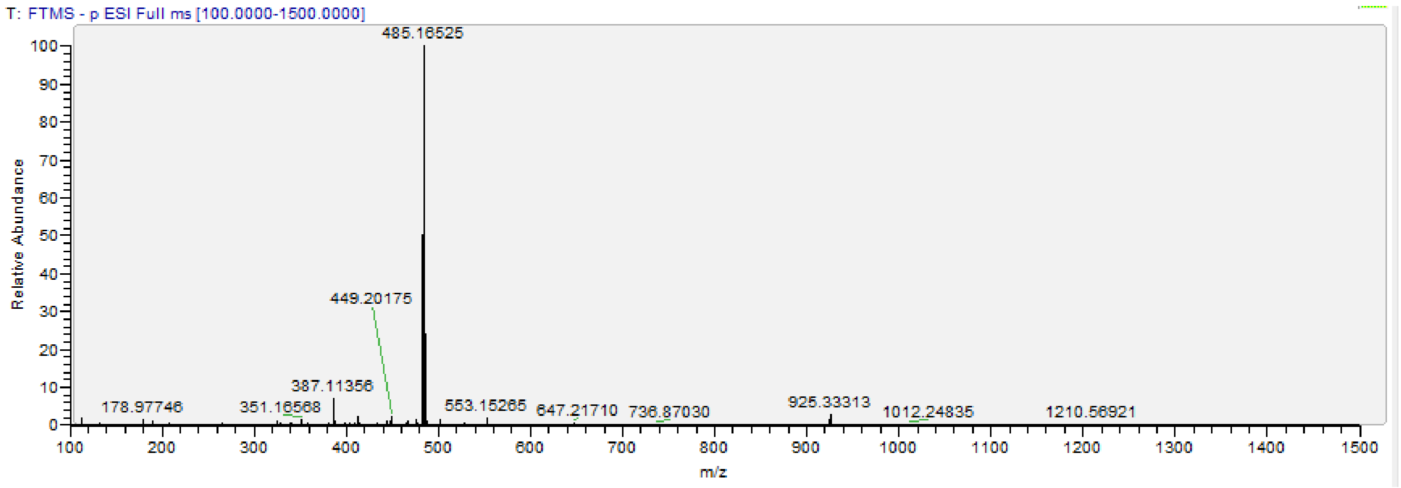

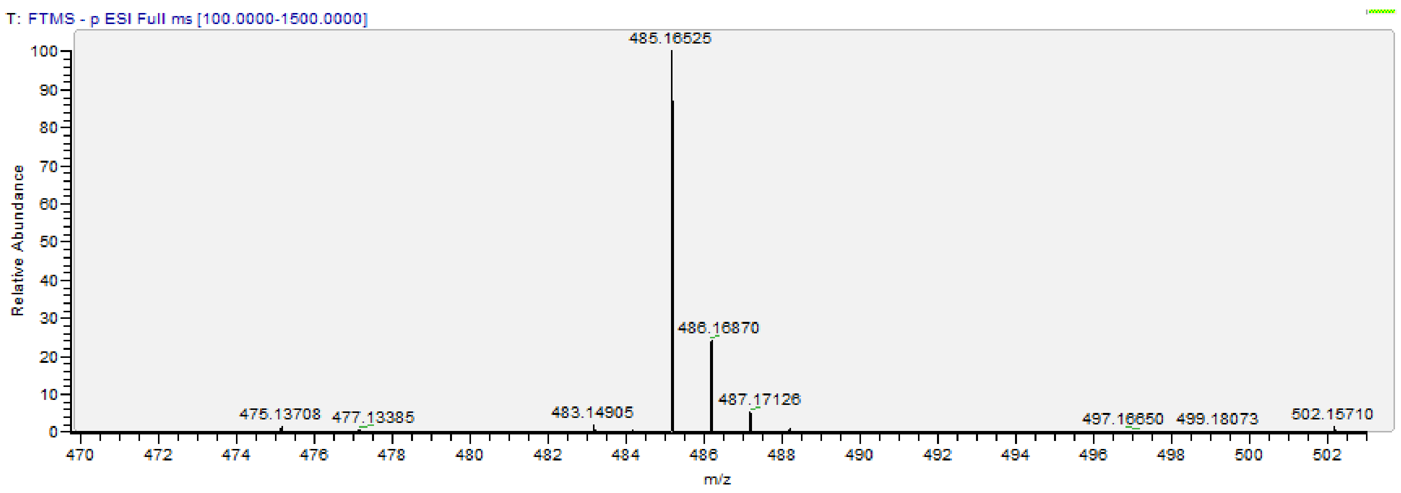

Elemental composition

Single mass

Mass: 485.16525

Max. results 10

Calculate

Idx	Formula	RDB	Delta ppm
1	$C_{22}H_{29}O_{12}$	8.5	-0.212

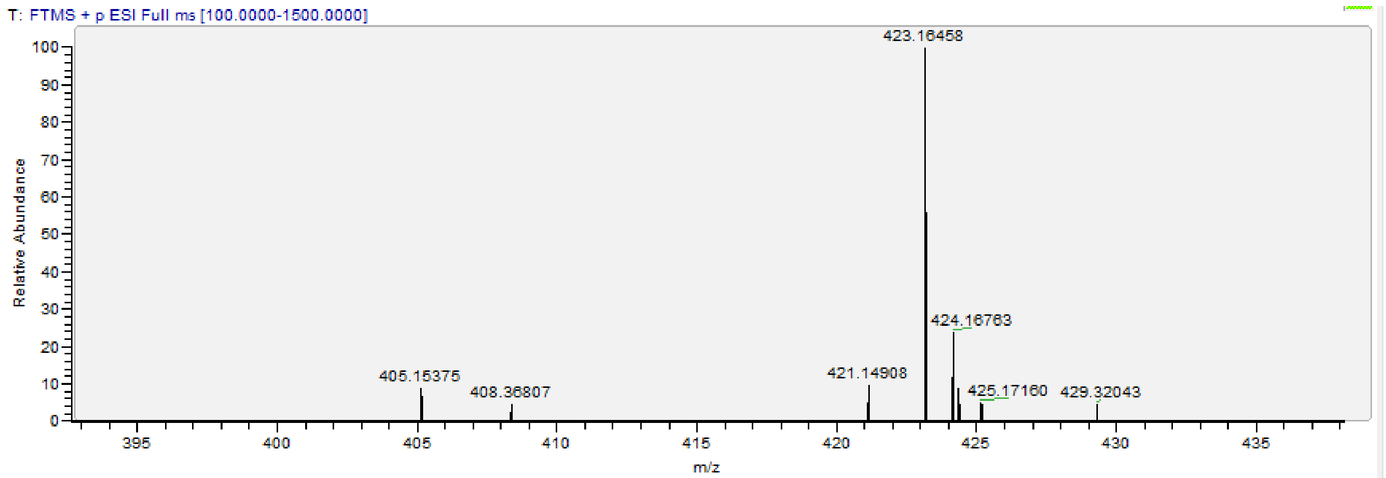

Elemental composition

Single mass

Mass: 423.16458

Max. results 10

Calculate

Idx	Formula	RDB	Delta ppm
1	$C_{21}H_{27}O_{9}$	8.5	-0.895

Figure S19. UV spectrum of compound 2

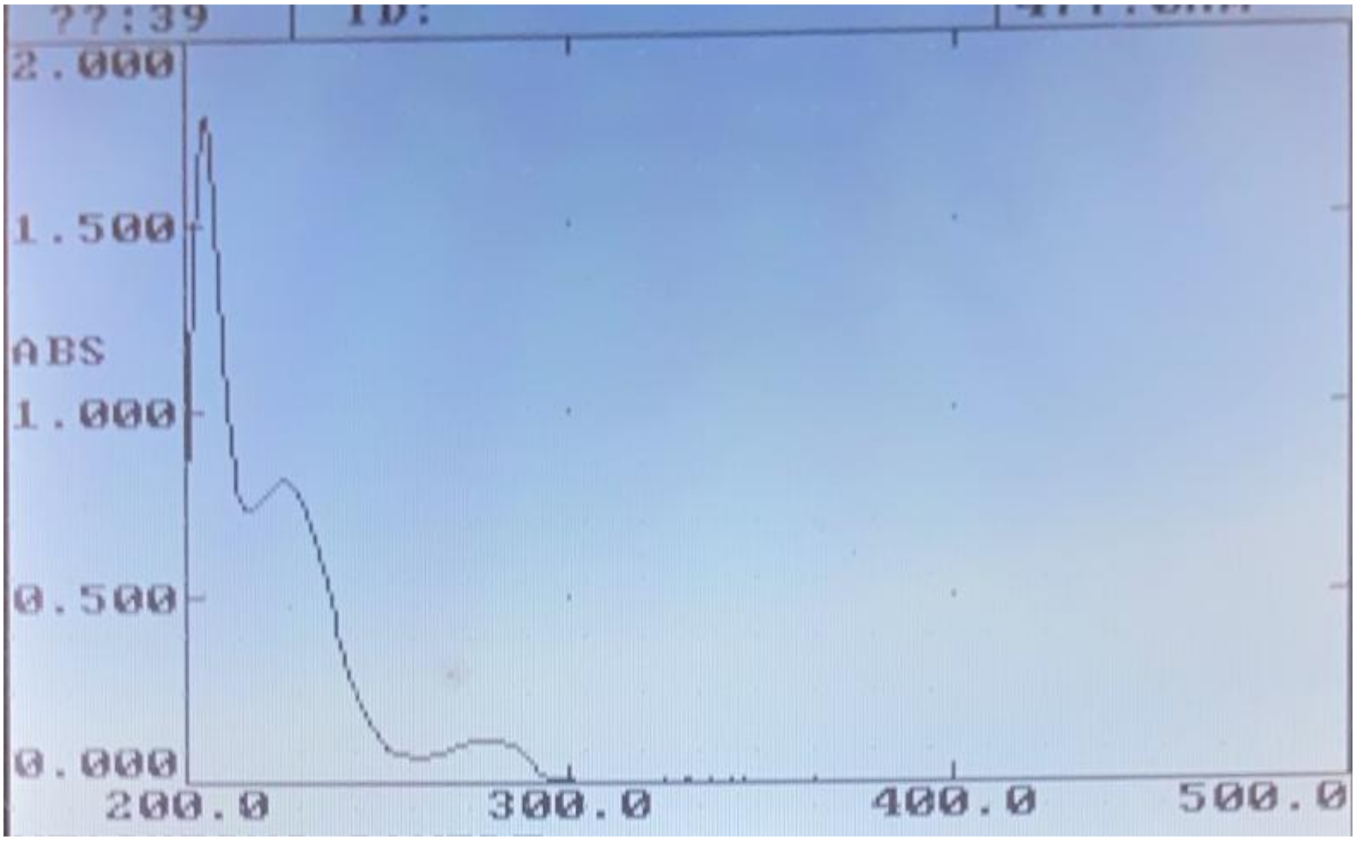

Figure S20. IR spectrum of compound 2

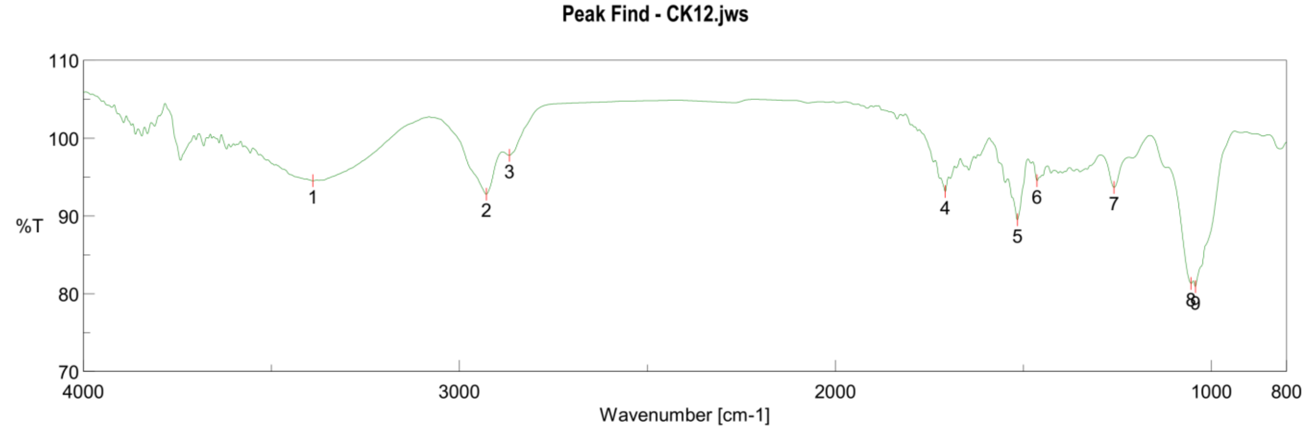

[Result of Peak Picking]

No.	Position	Intensity	No.	Position	Intensity	No.	Position	Intensity
1	3389.28	94.5115	2	2928.38	92.7747	3	2866.67	97.7729
4	1707.66	93.1156	5	1515.78	89.4929	6	1463.71	94.5119
7	1259.29	93.6527	8	1053.91	81.3038	9	1042.34	80.9077

Figure S21. ^{1}H-NMR spectrum of compound 3

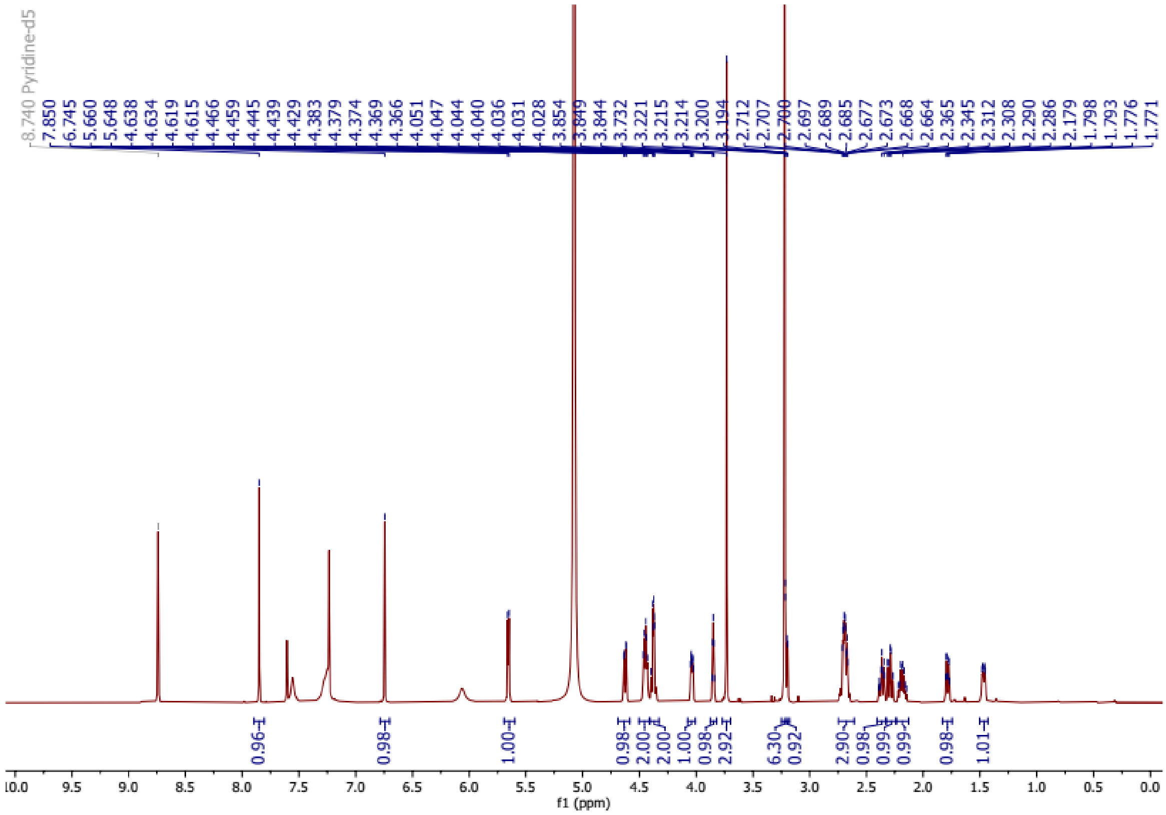

Figure S22. ^{13}C-NMR spectrum of compound 3

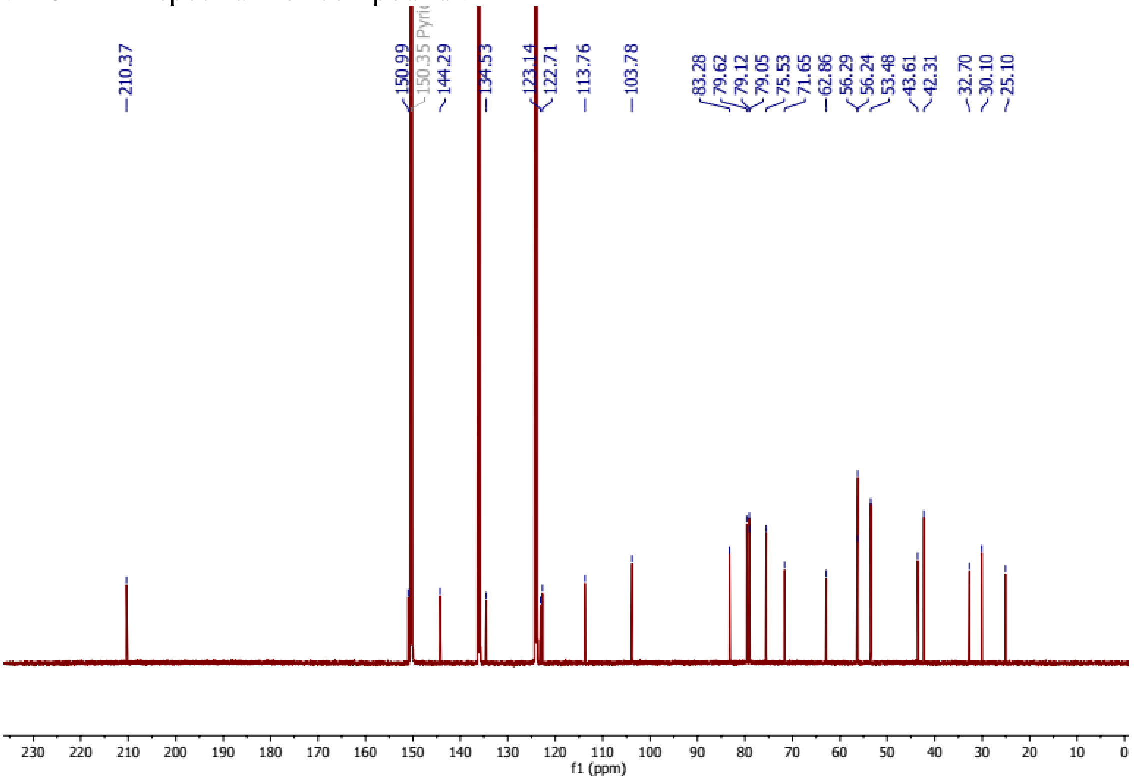

Figure S23. DEPT NMR spectrum of compound 3

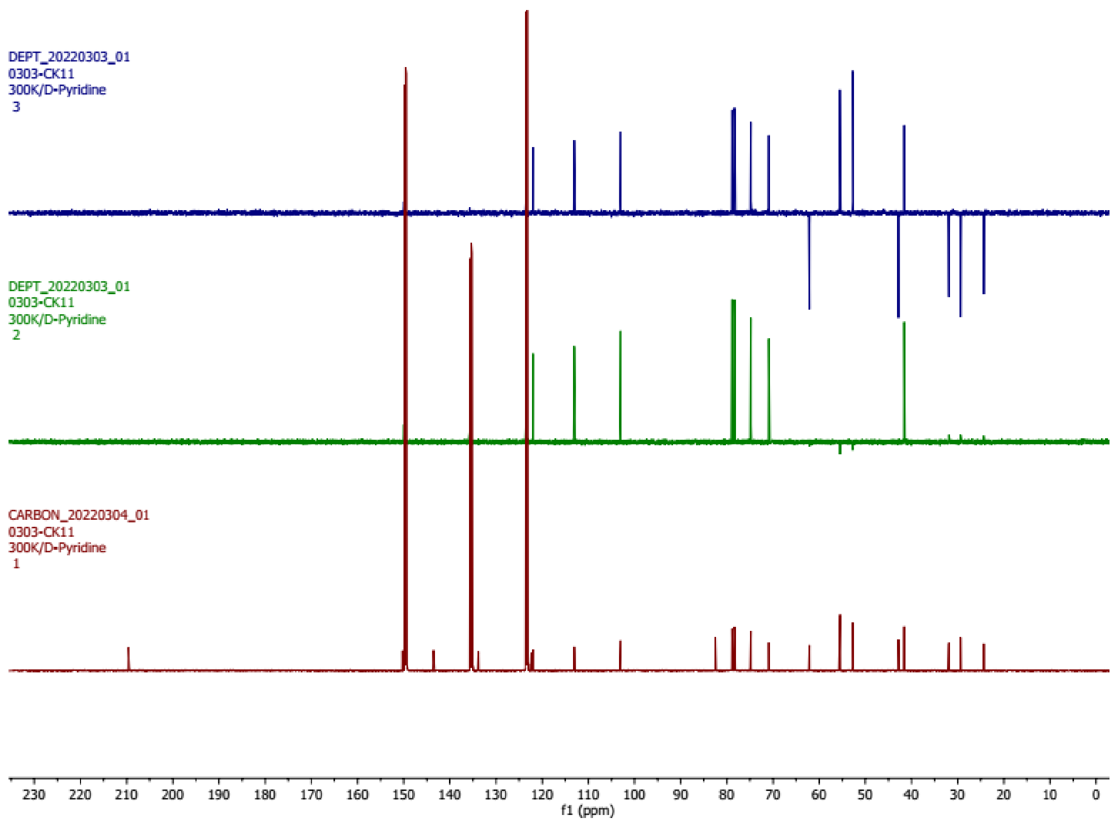

Figure S24. HSQC NMR spectrum of compound 3

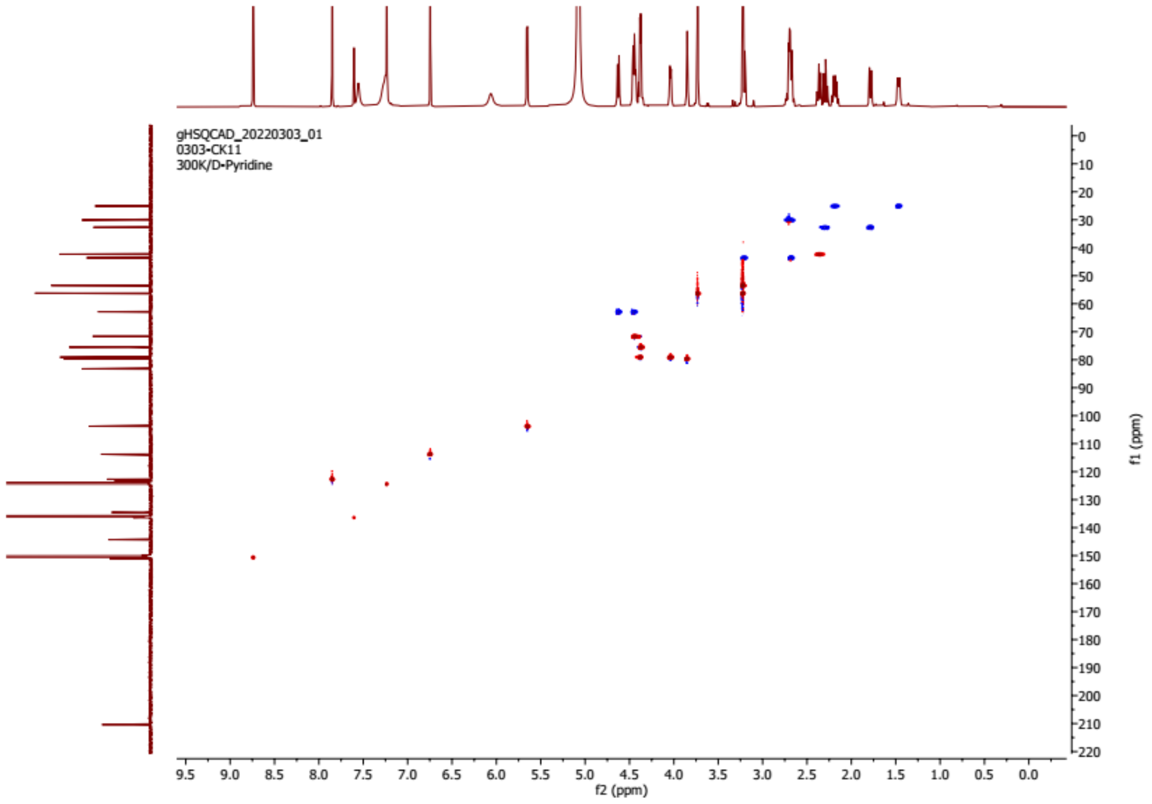

Figure S25. COSY NMR spectrum of compound 3

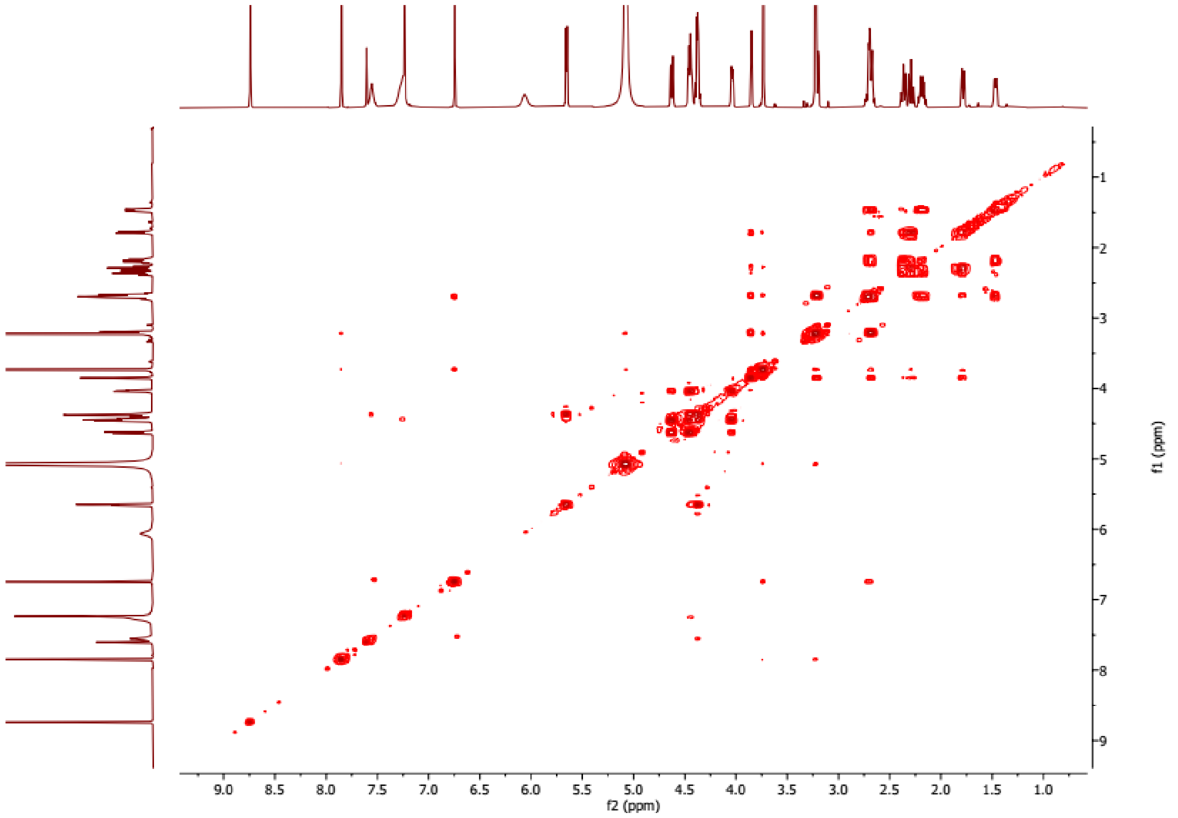

Figure S26. HMBC NMR spectrum of compound 3

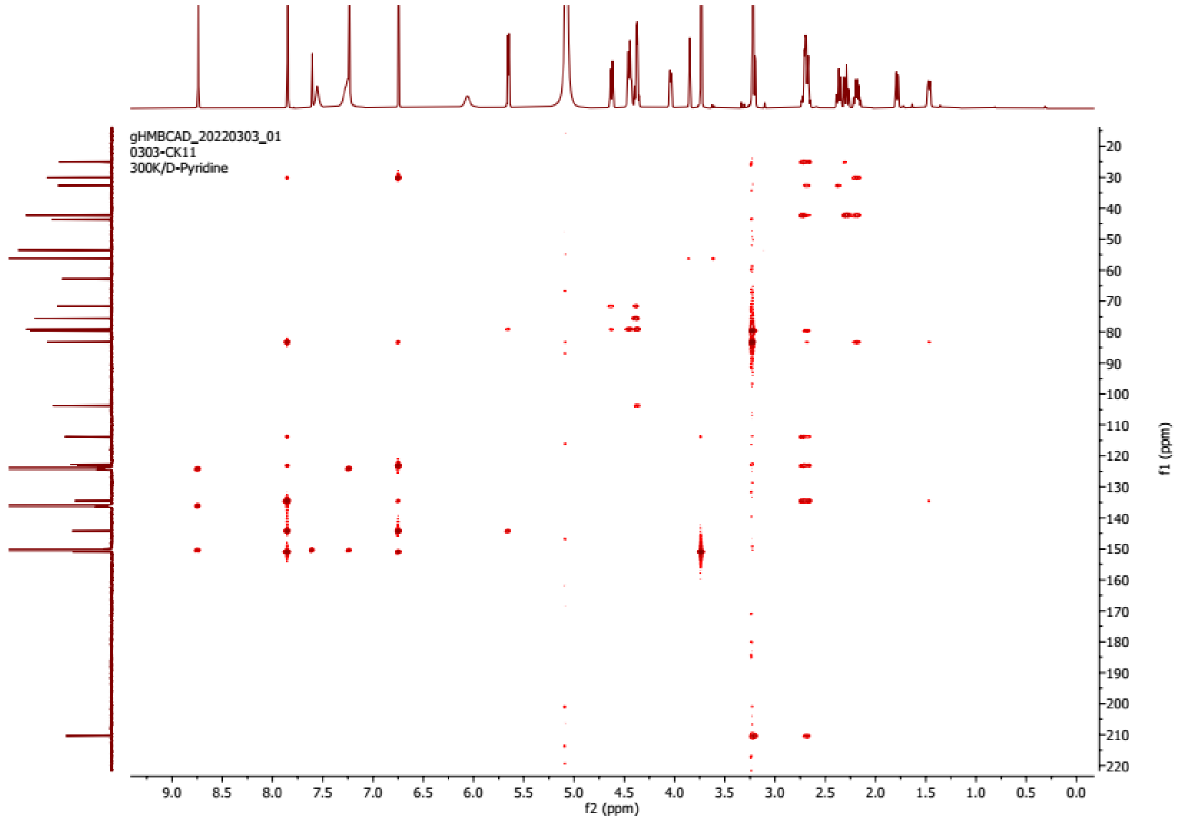

Figure S27. NOESY NMR spectrum of compound 3

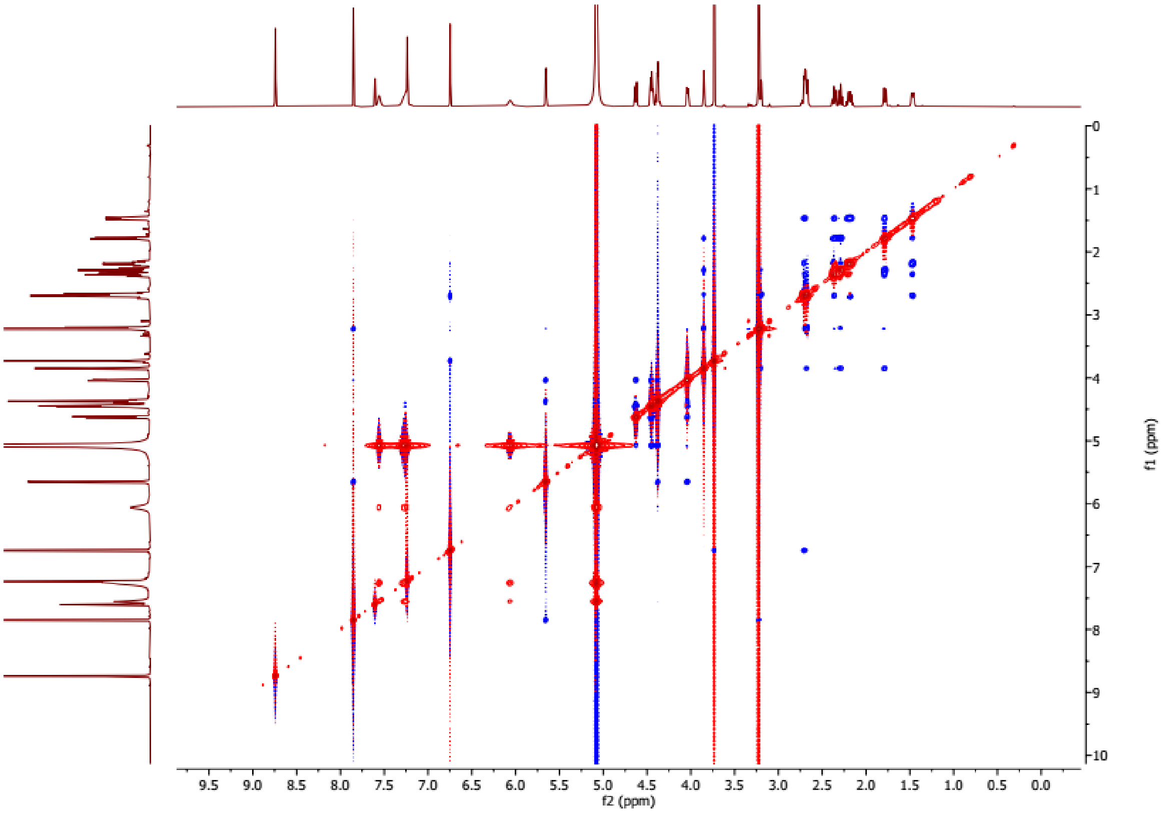

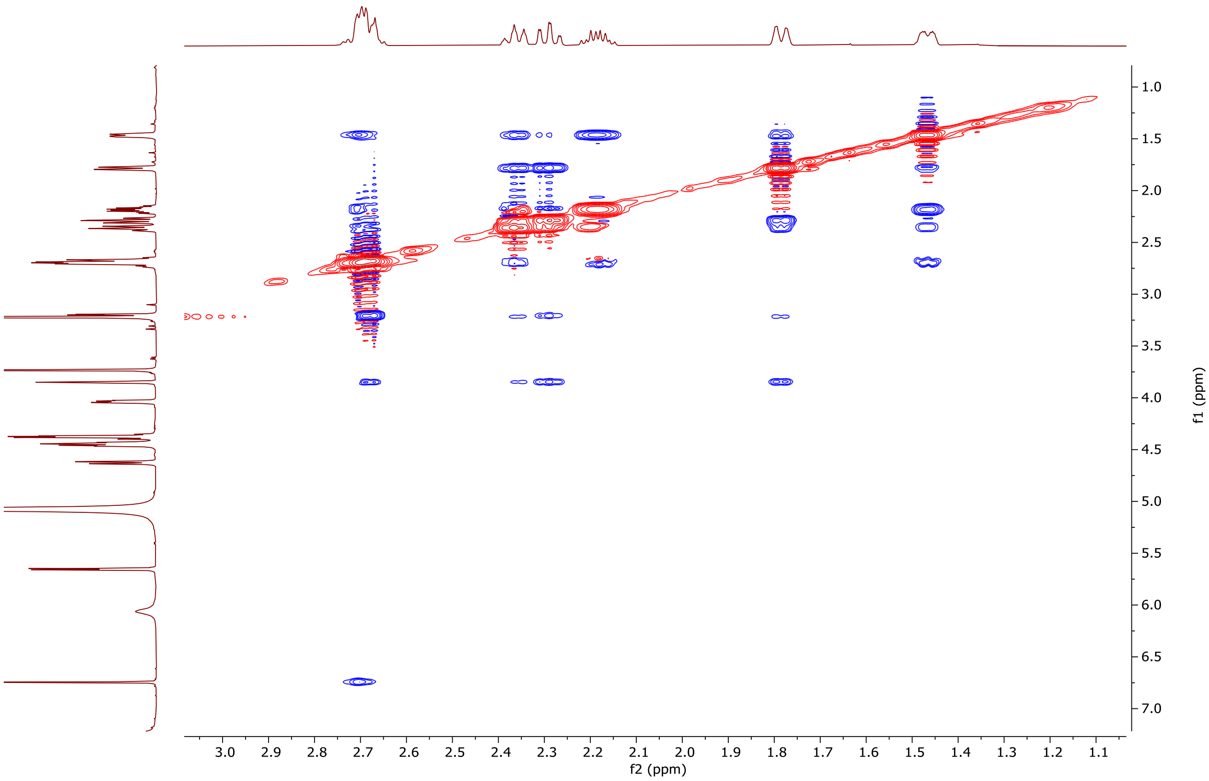

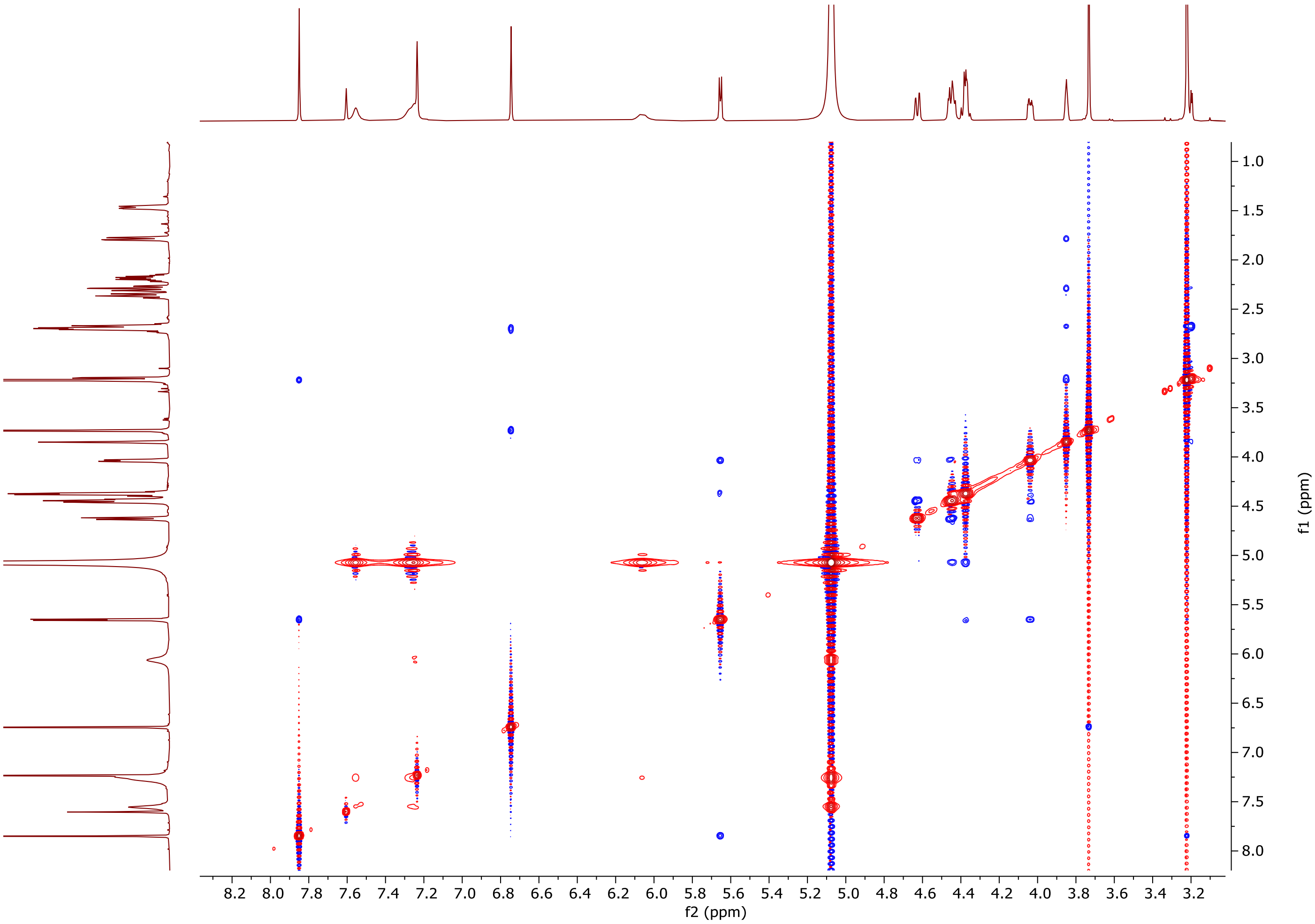

Figure S28. HR-ESI-MS spectrum of compound 3

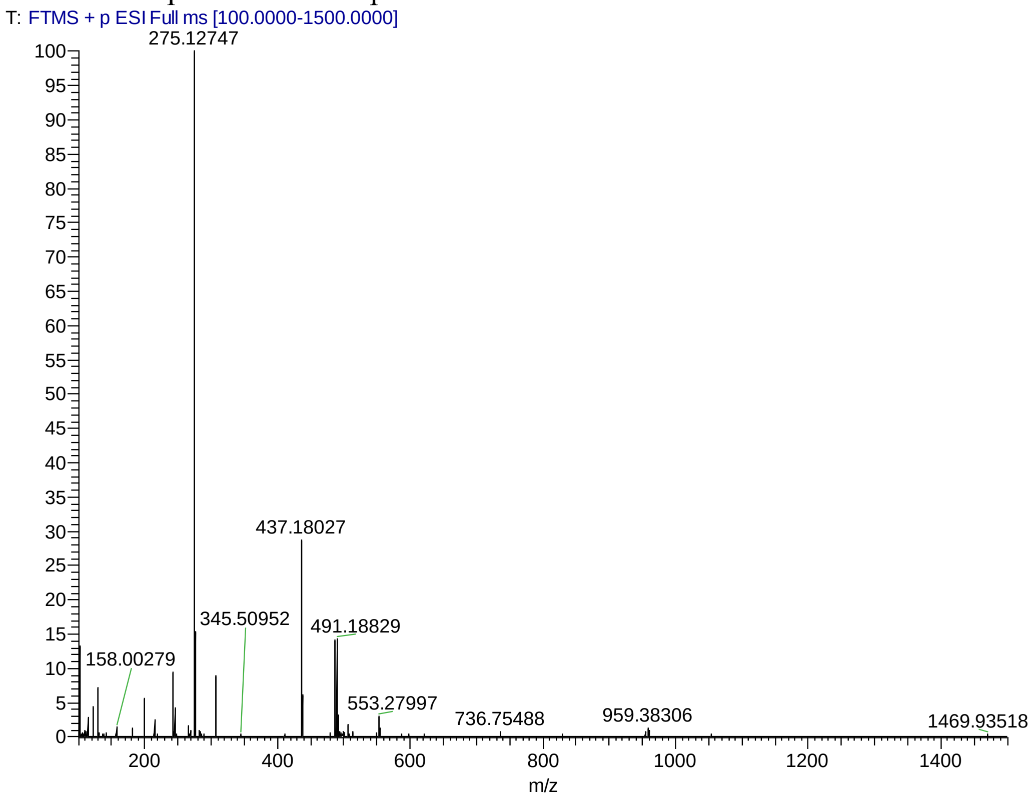

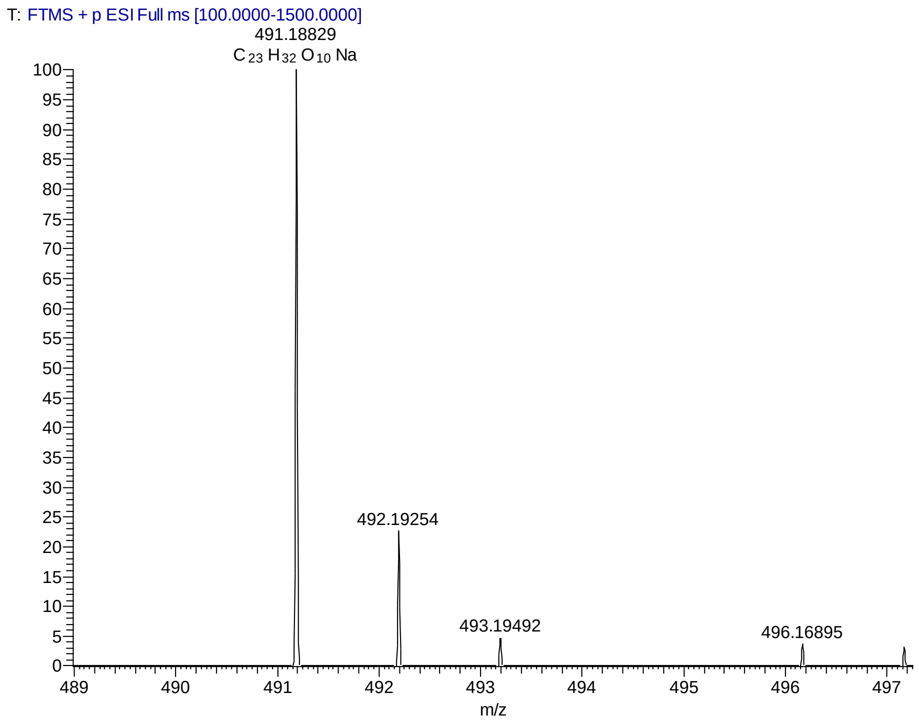

Elemental composition

Single mass

Mass: 491.18829

Max. results 10

Calculate

Idx	Formula	RDB	Delta ppm
1	$C_{23}H_{32}O_{10}Na$	7.5	-0.974

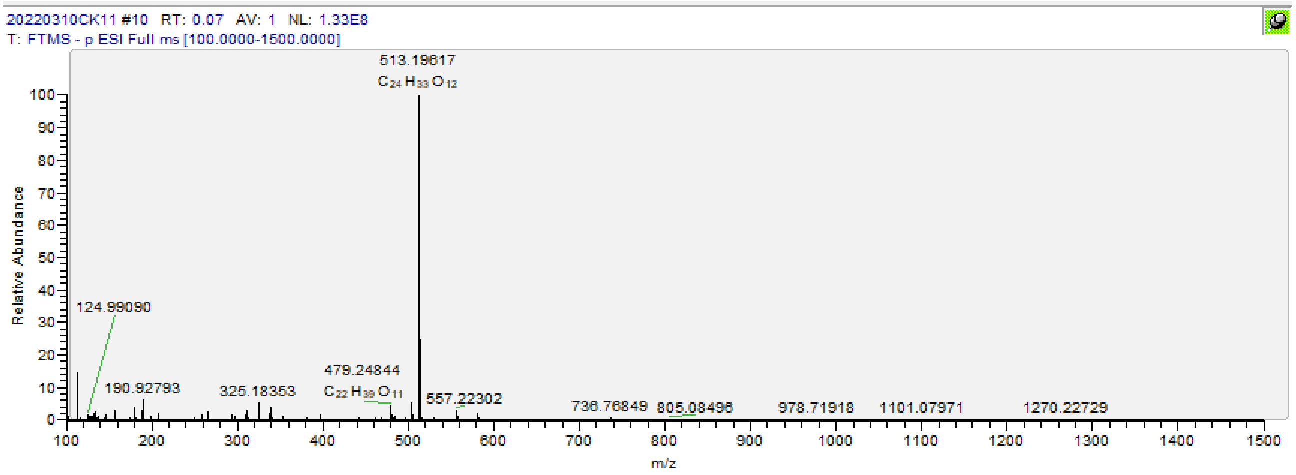

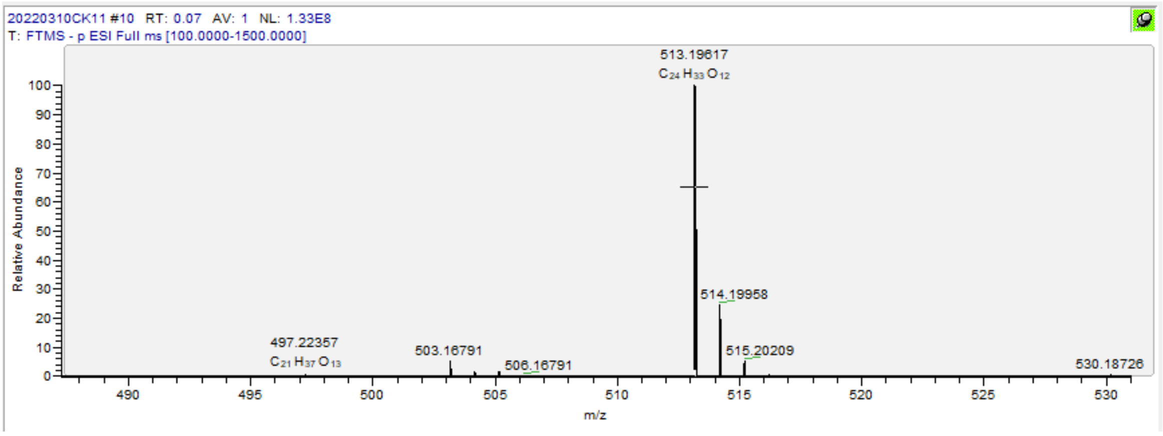

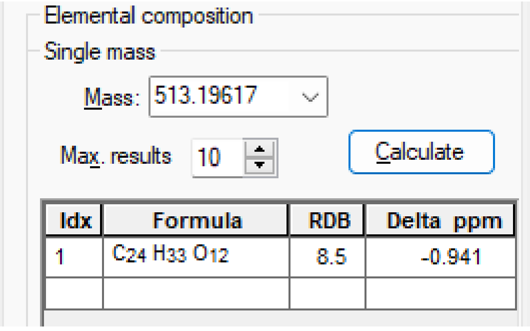

Figure S29. UV spectrum of compound 3

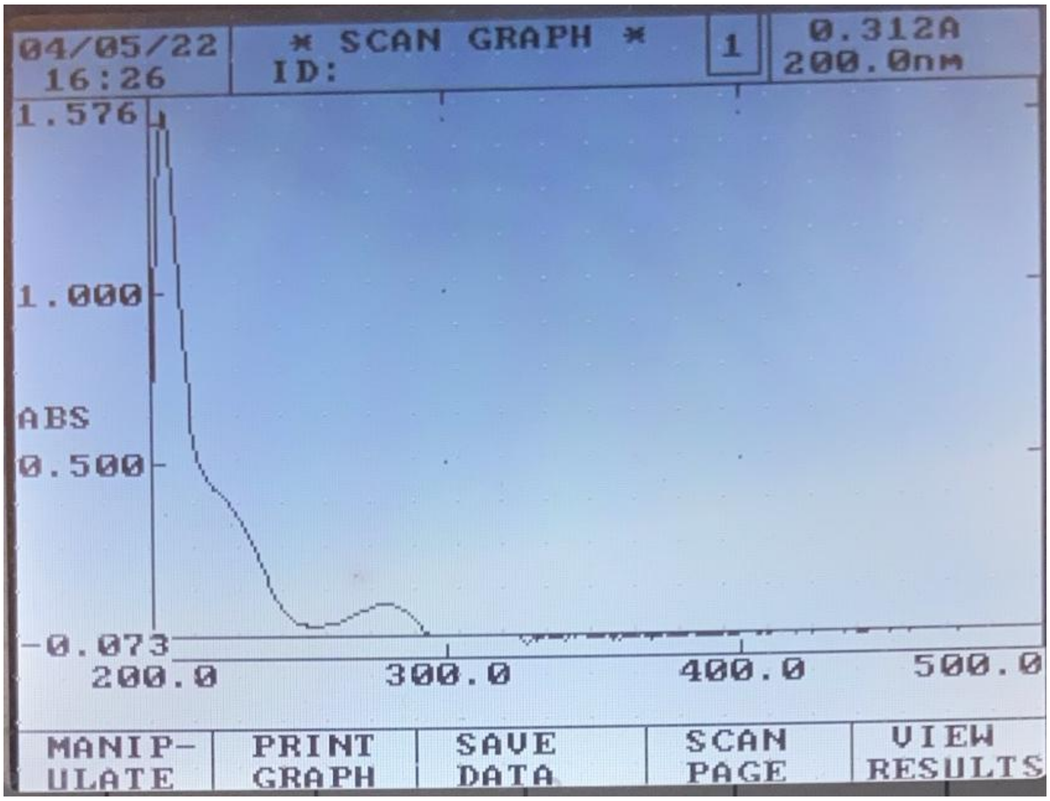

Figure S30. IR spectrum of compound 3

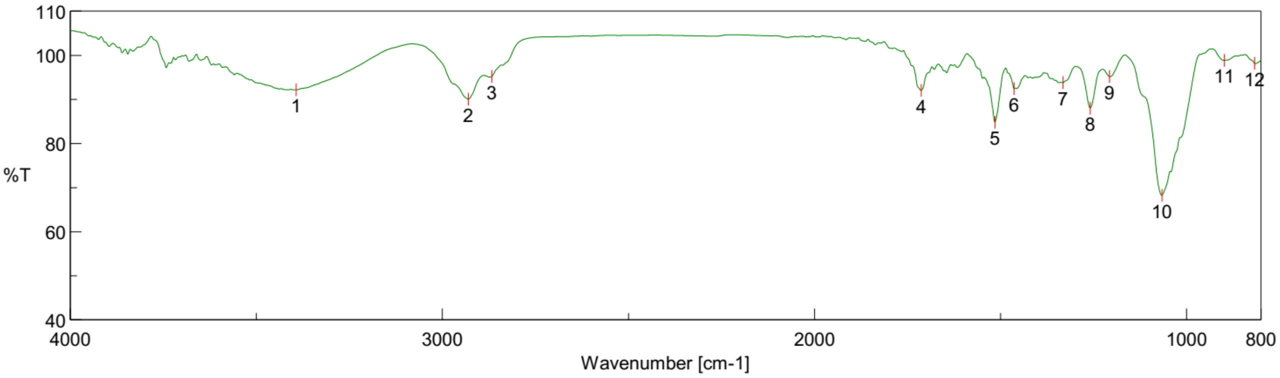

[Result of Peak Picking]

No.	Position	Intensity	No.	Position	Intensity	No.	Position	Intensity
1	3393.14	92.1475	2	2930.31	90.0377	3	2867.63	95.1485
4	1713.44	91.9985	5	1514.81	84.7888	6	1463.71	92.4195
7	1332.57	93.772	8	1259.29	88.0071	9	1207.22	95.144
10	1066.44	68.224	11	898.666	98.823	12	817.67	98.1719
13	676.892	95.0048	14	559.255	93.3342			

Figure S31. ^{1}H-NMR spectrum of compound 4

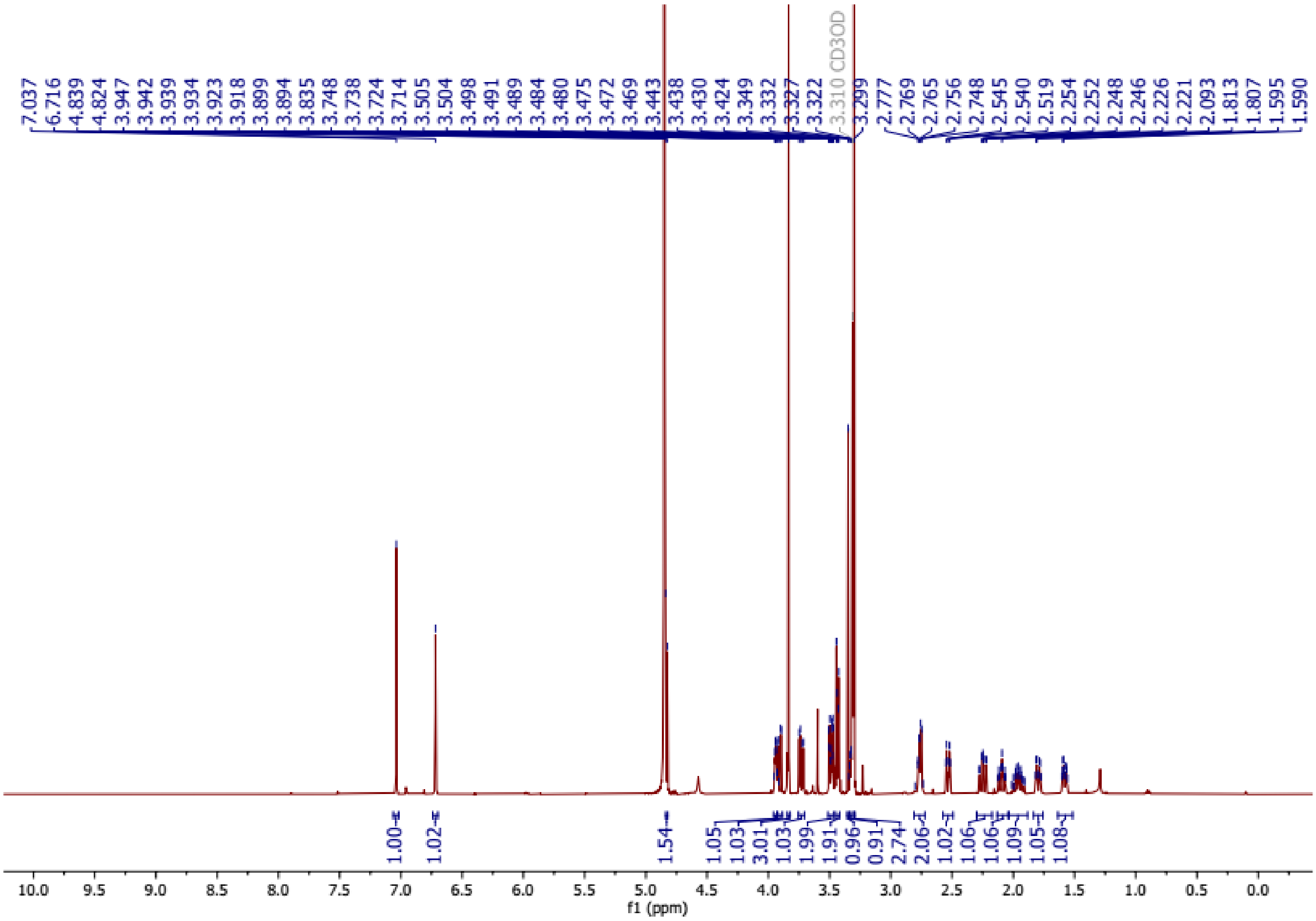

Figure S32. ^{13}C-NMR spectrum of compound 4

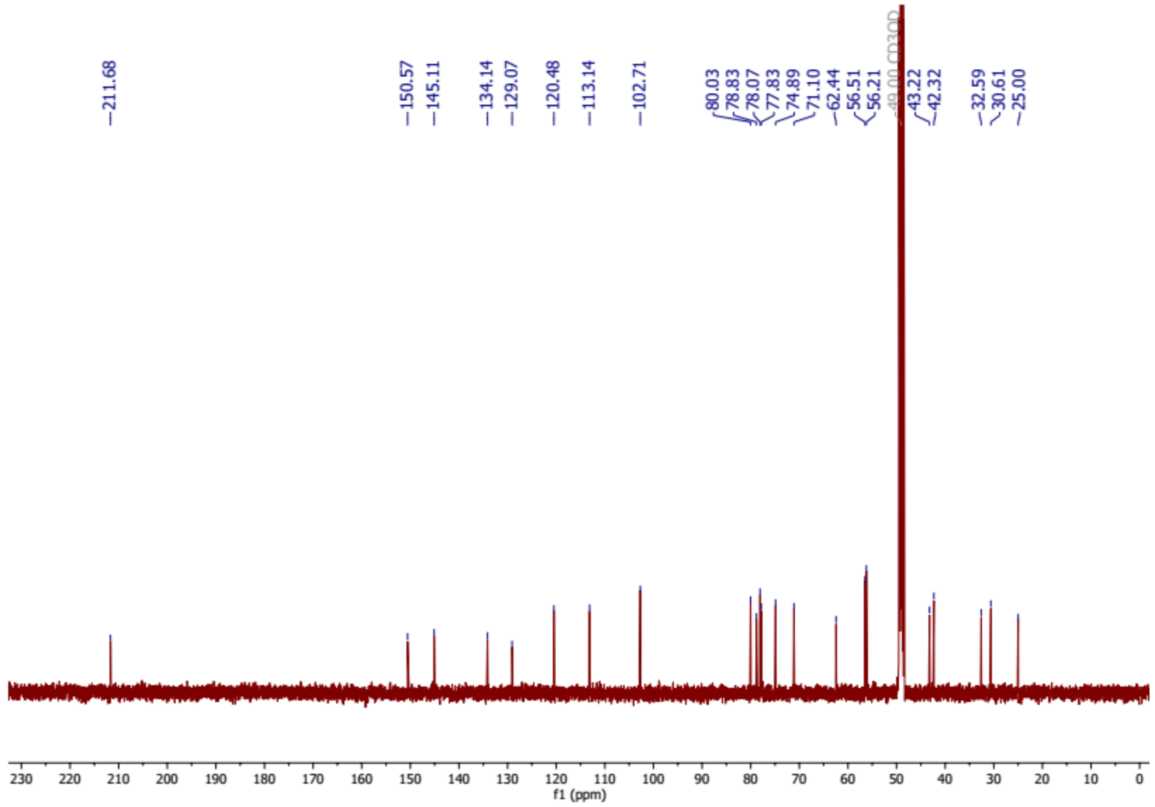

Figure S33. HSQC NMR spectrum of compound 4

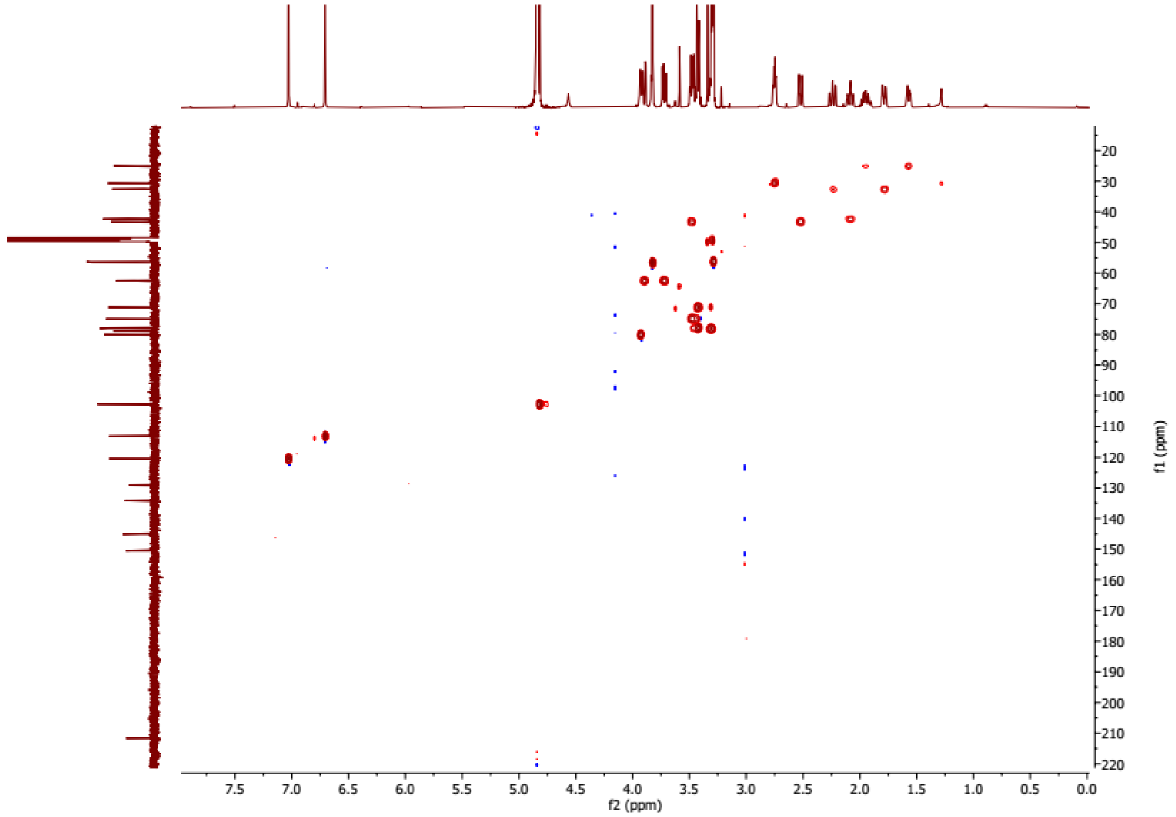

Figure S34. HMBC NMR spectrum of compound 4

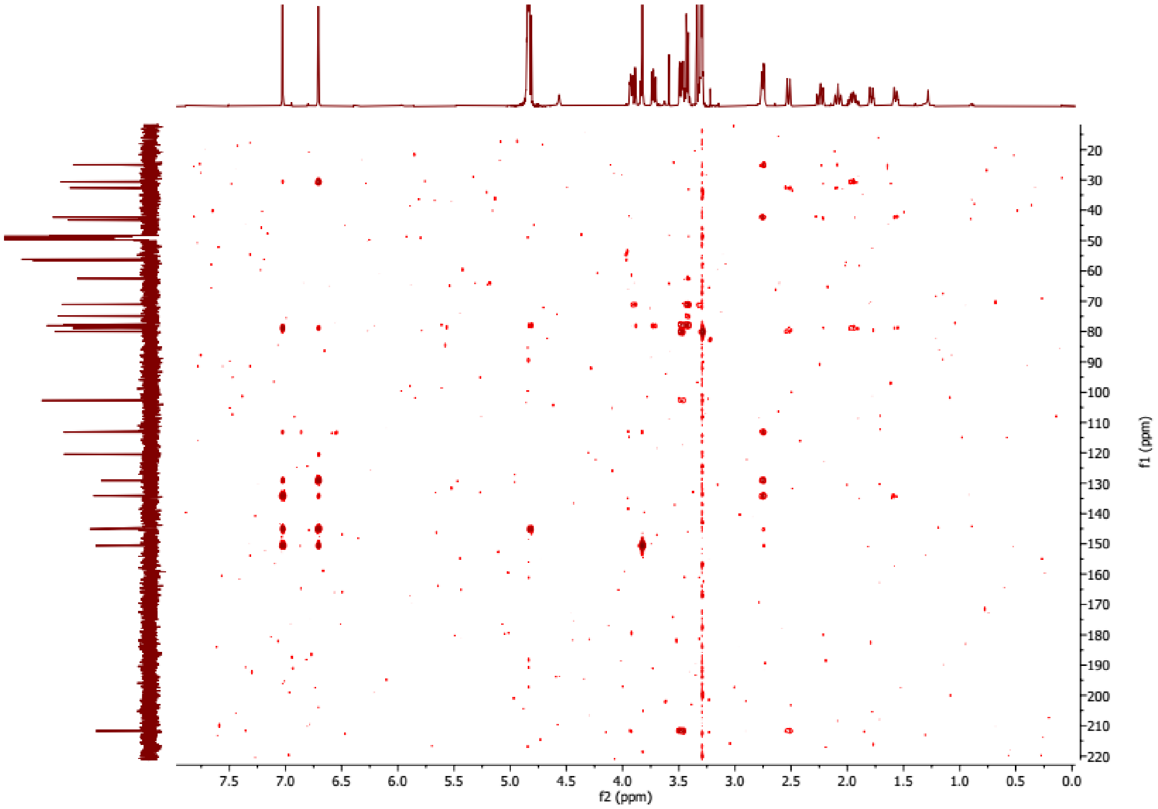

Figure S35. COSY NMR spectrum of compound 4

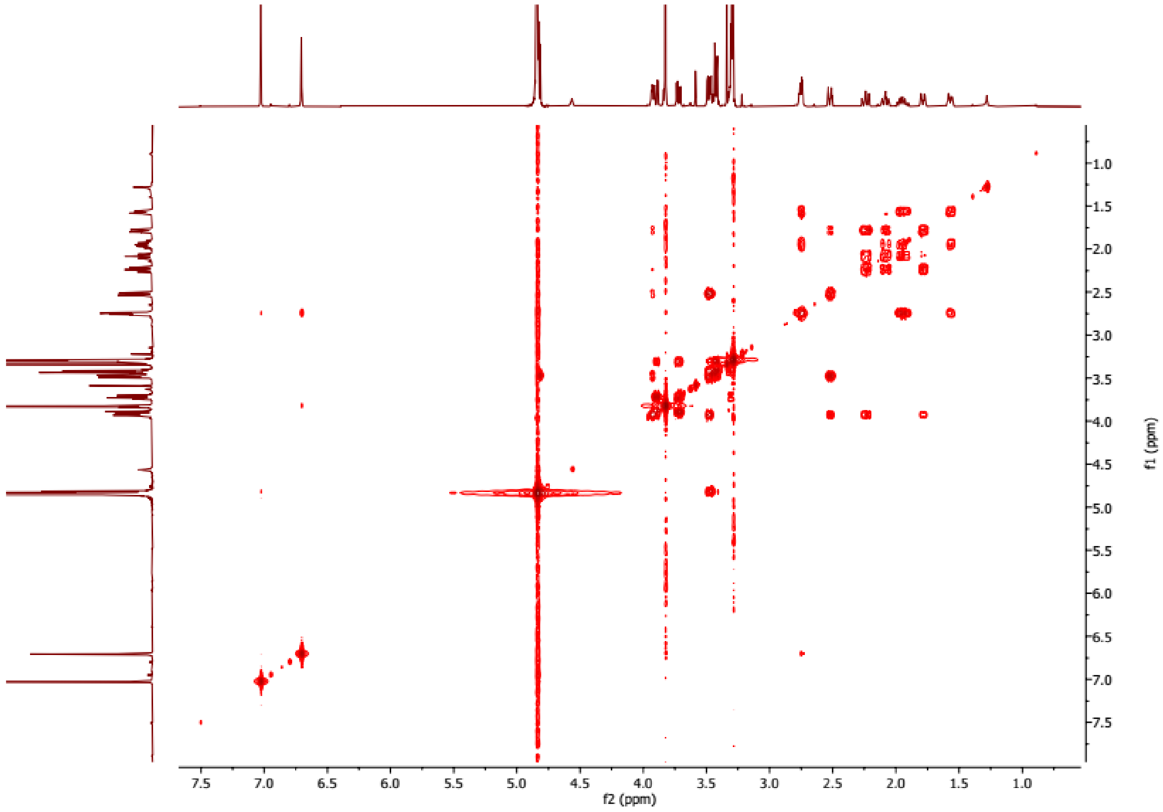

Figure S36. NOESY NMR spectrum of compound 4

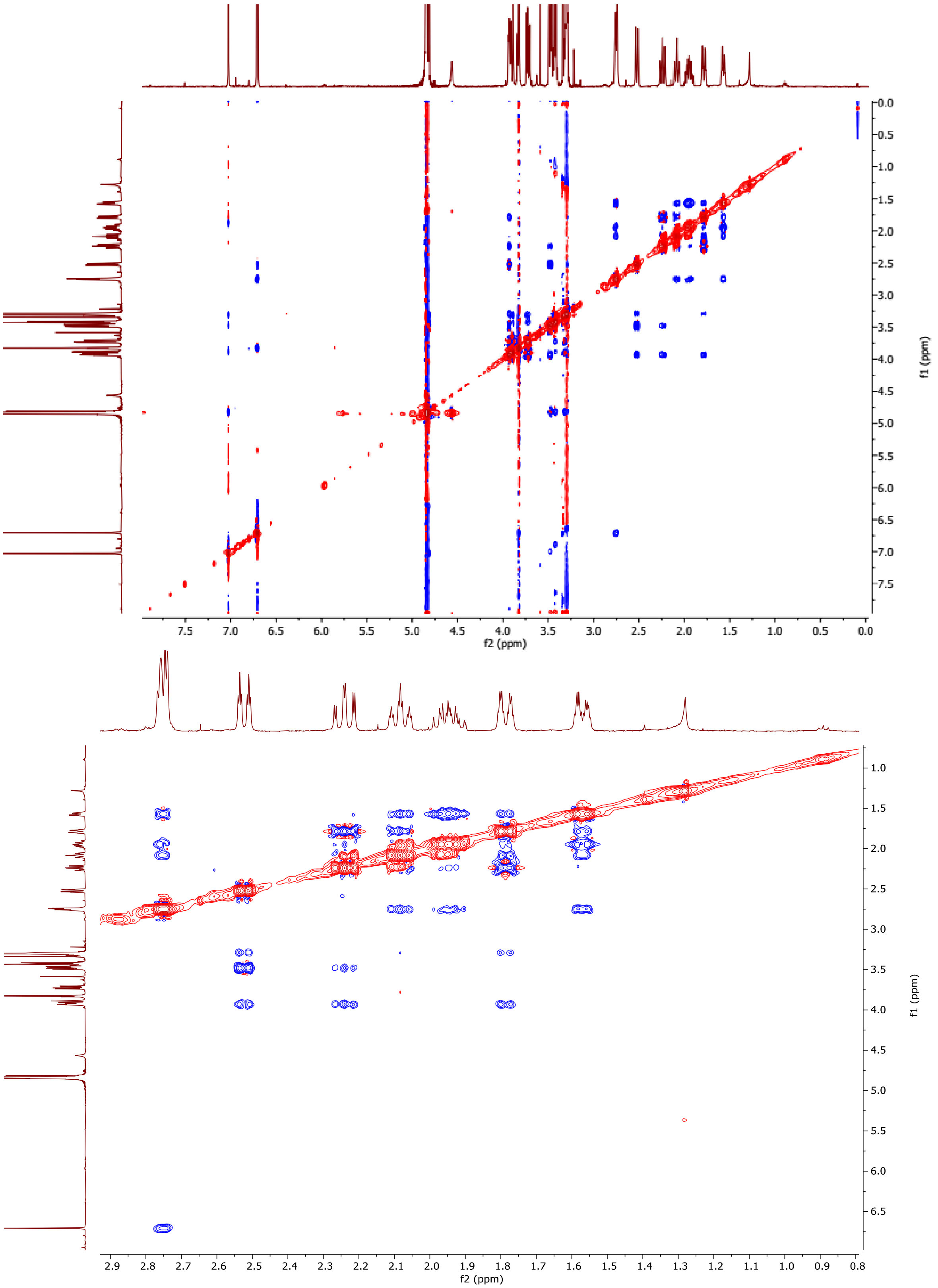

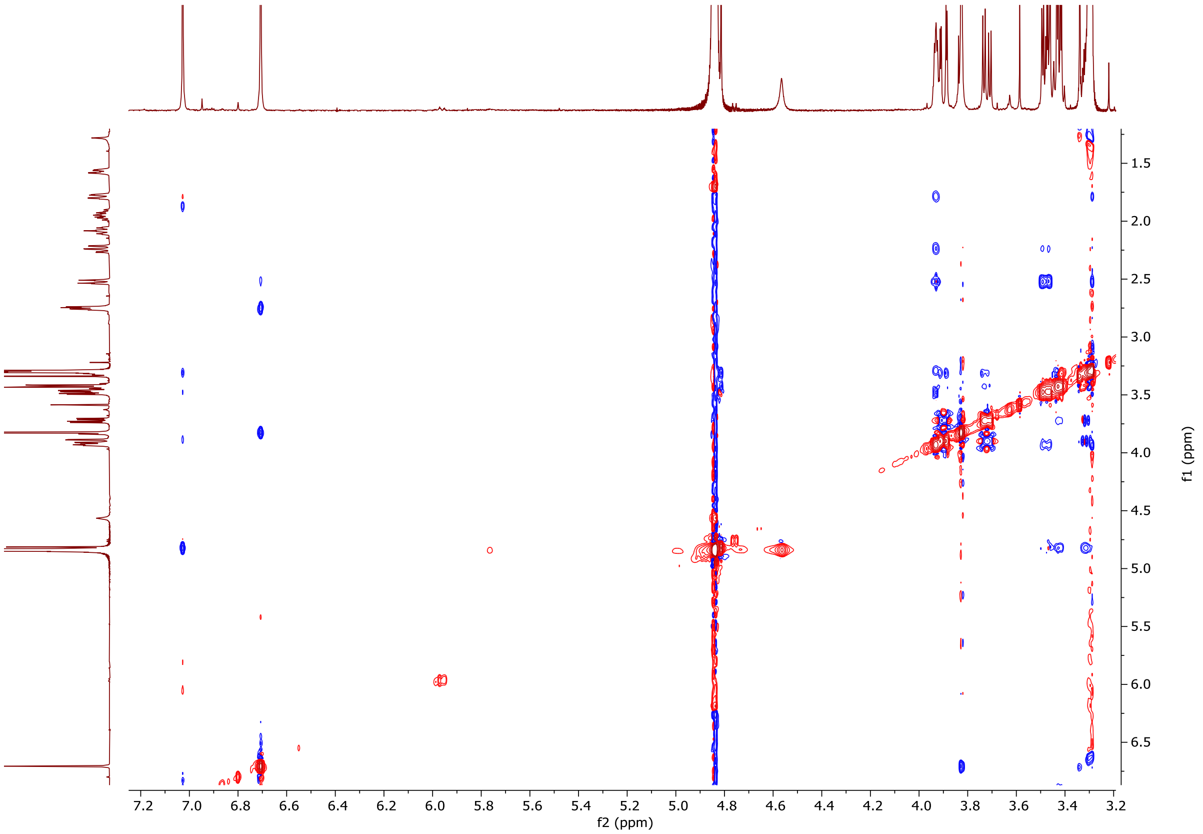

Figure S37. HR-ESI-MS spectrum of compound 4

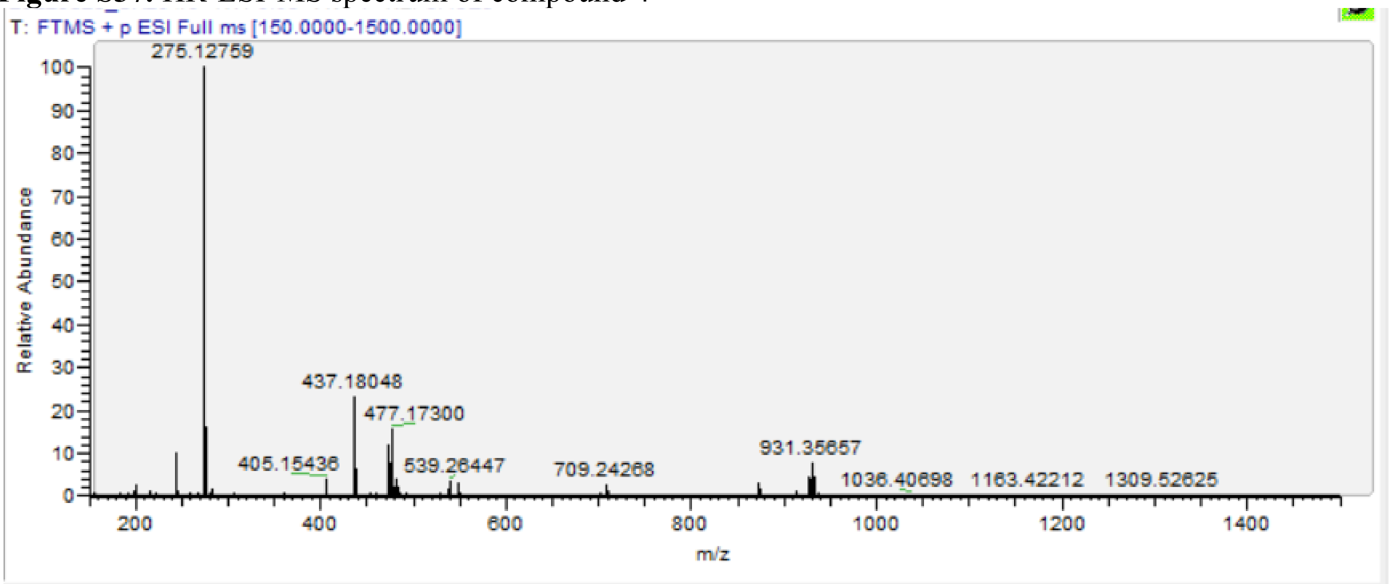

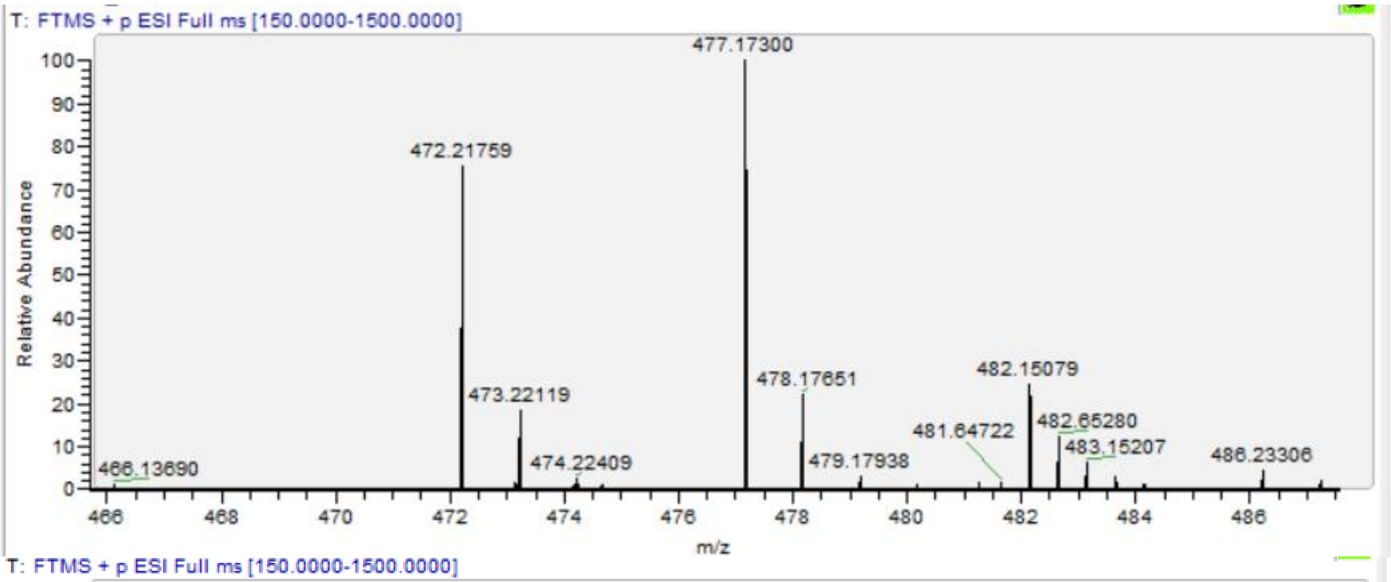

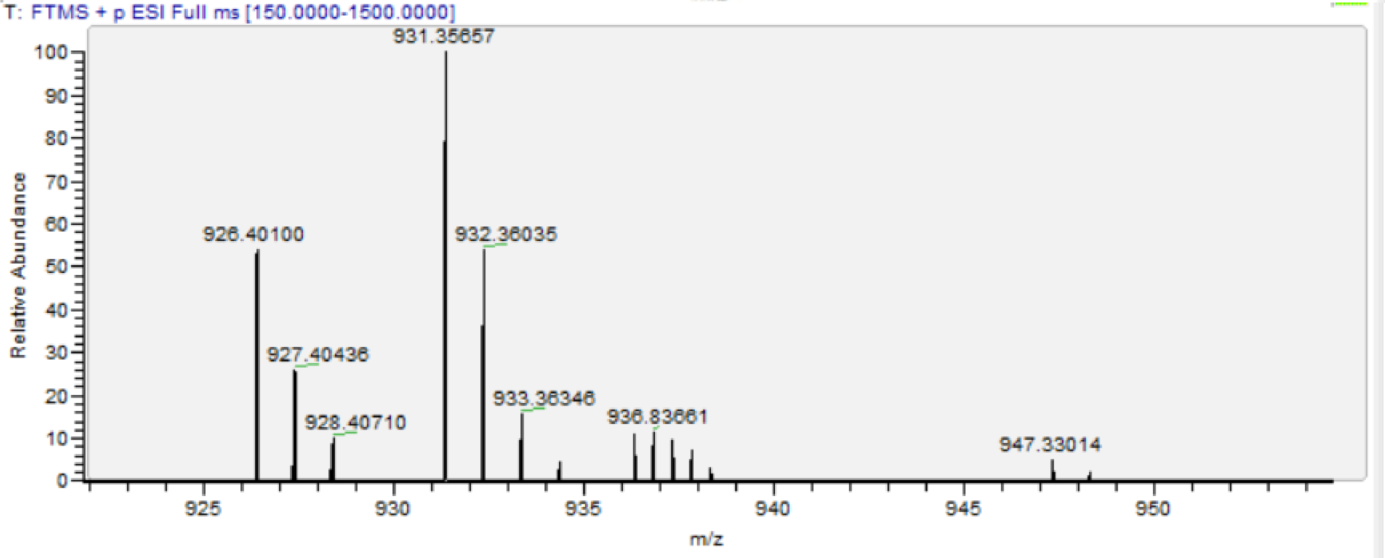

Elemental composition

Single mass

Mass: 477.17300

Max. results 10

Calculate

Idx	Formula	RDB	Delta ppm
1	$C_{22}H_{30}O_{10}Na$	7.5	-0.248

Elemental composition

Single mass

Mass: 931.35657

Max. results 10

Calculate

Idx	Formula	RDB	Delta ppm
1	$C_{44}H_{60}O_{20}Na$	14.5	-0.478

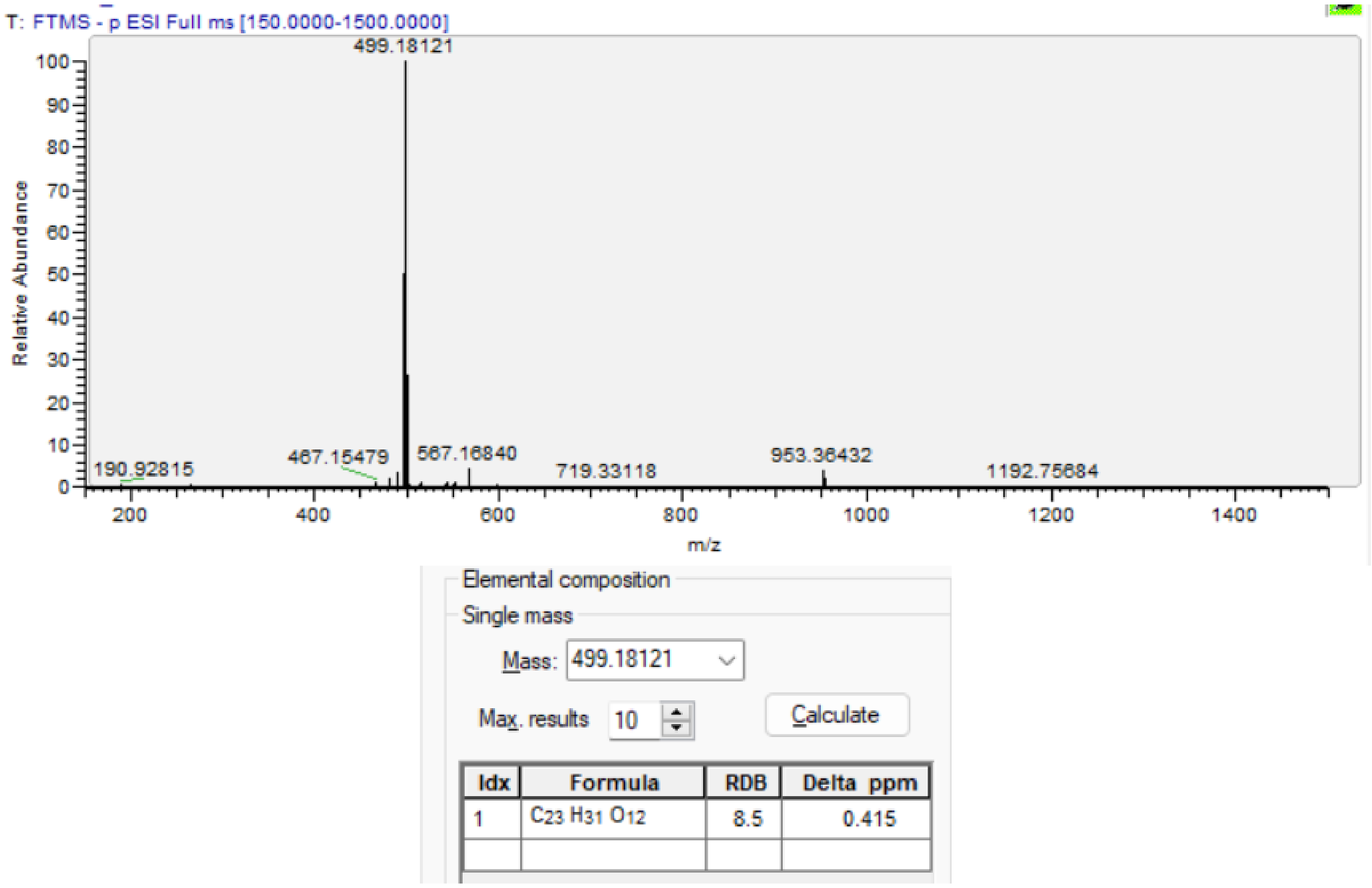

Figure S38. UV spectrum of compound 4

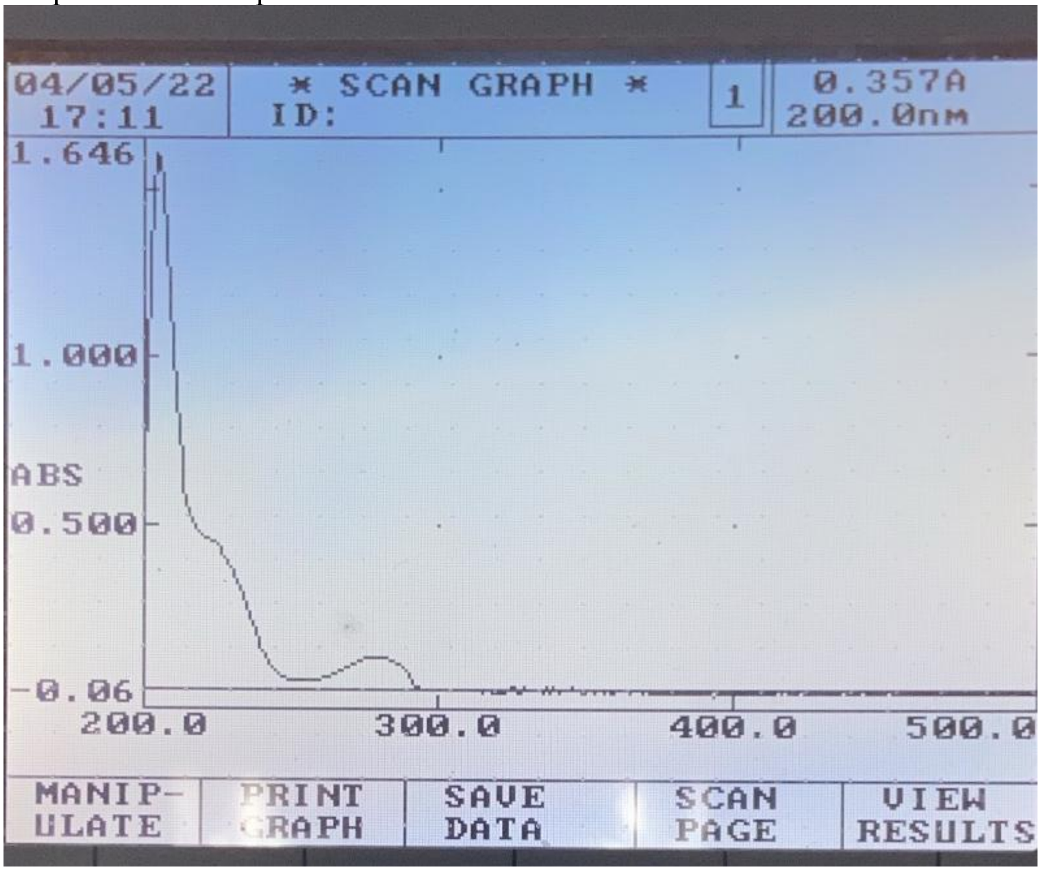

Figure S39. IR spectrum of compound 4

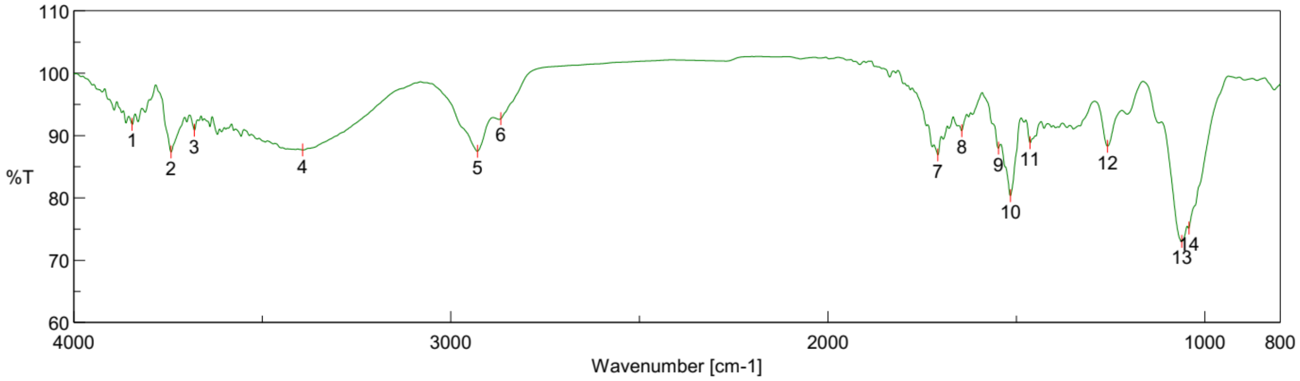

[Result of Peak Picking]

No.	Position	Intensity	No.	Position	Intensity	No.	Position	Intensity
1	3845.36	91.7778	2	3742.19	87.3521	3	3680.48	90.8358
4	3393.14	87.6597	5	2929.34	87.457	6	2867.63	92.668
7	1708.62	86.8828	8	1644.98	90.769	9	1547.59	87.9542
10	1515.78	80.348	11	1463.71	88.8174	12	1258.32	88.2646
13	1061.62	73.0089	14	1042.34	75.1538	15	672.071	92.831
16	560.22	92.2924	17	471.51	45.3382	18	449.333	57.4211
19	413.656	48.7862						

Figure S40. ^{1}H-NMR spectrum of compound 5

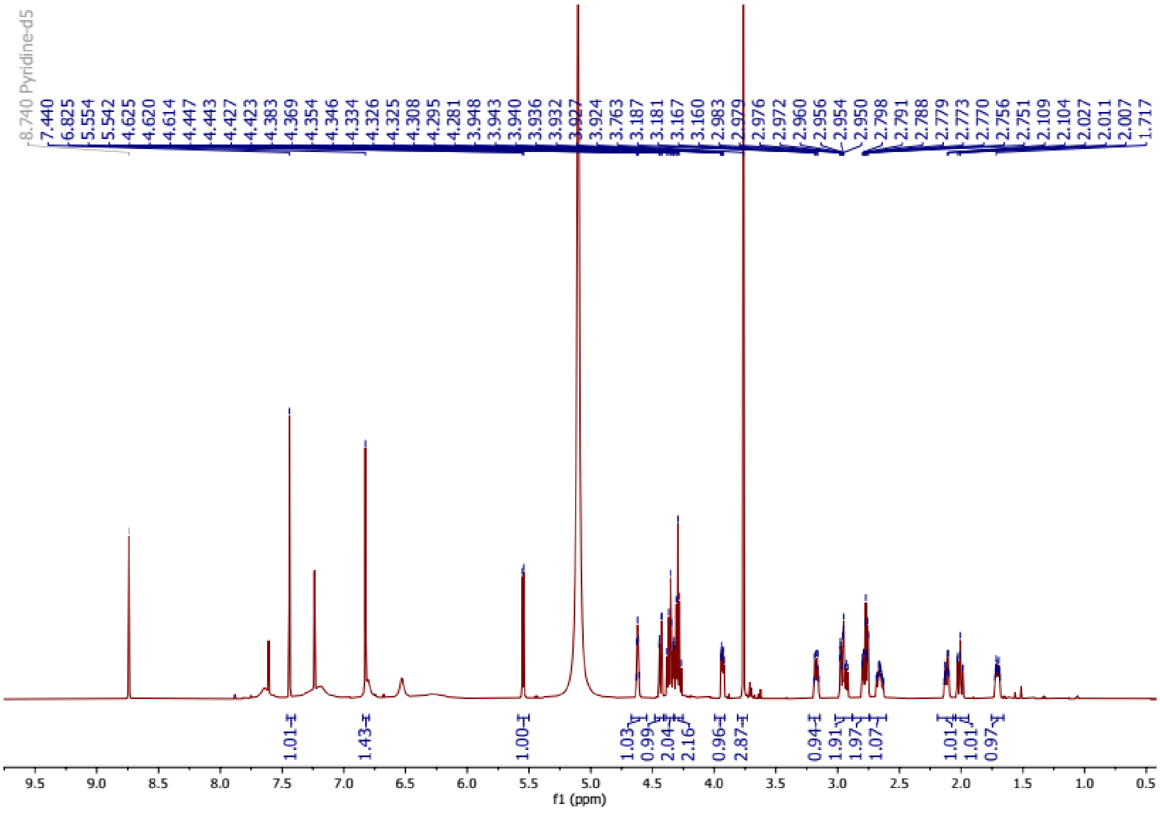

Figure S41. ^{13}C-NMR spectrum of compound 5

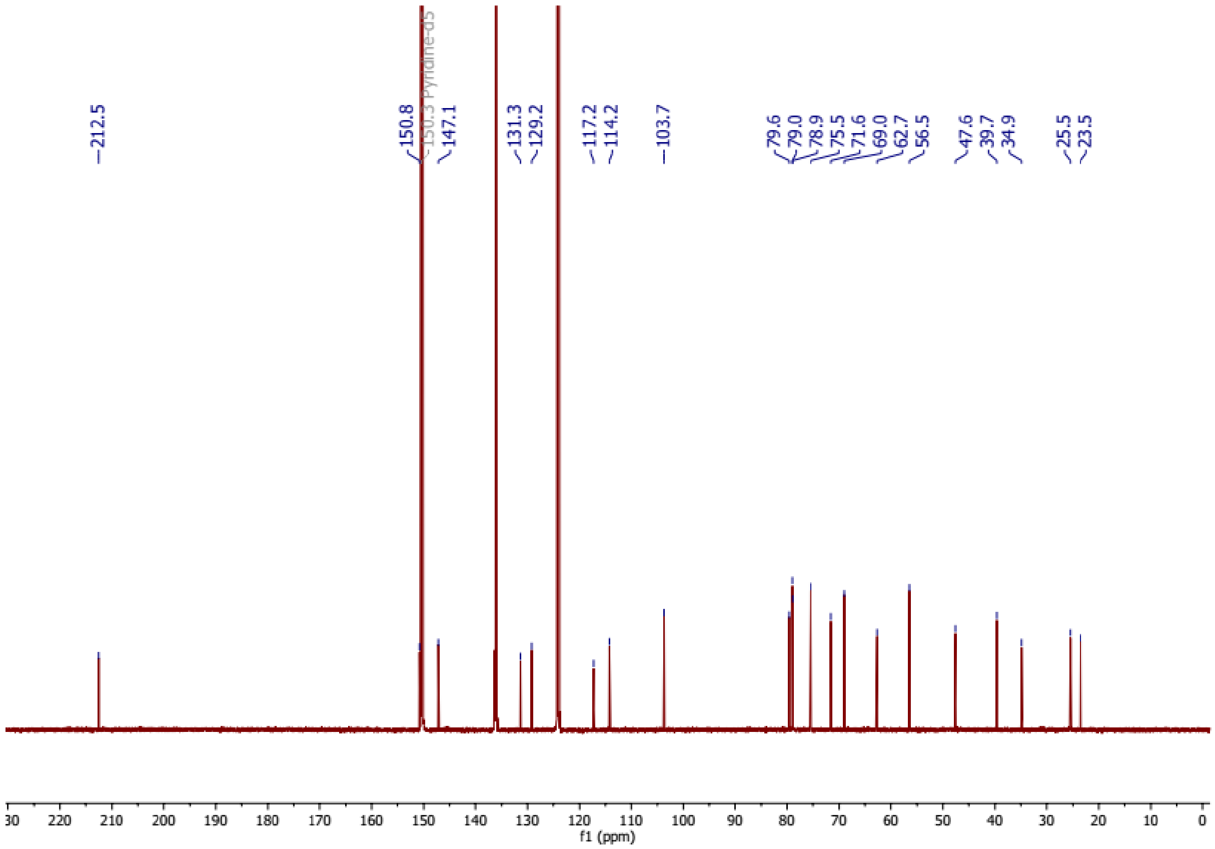

Figure S42. DEPT NMR spectrum of compound 5

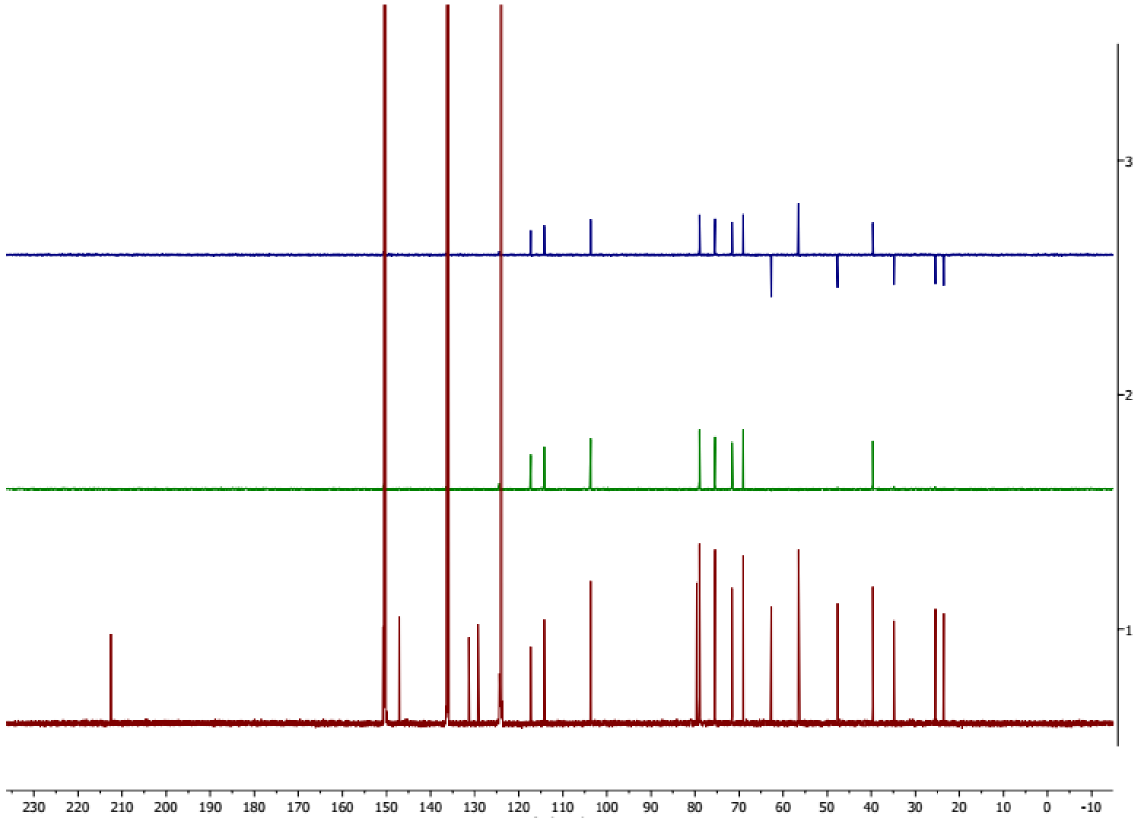

Figure S43. HSQC NMR spectrum of compound 5

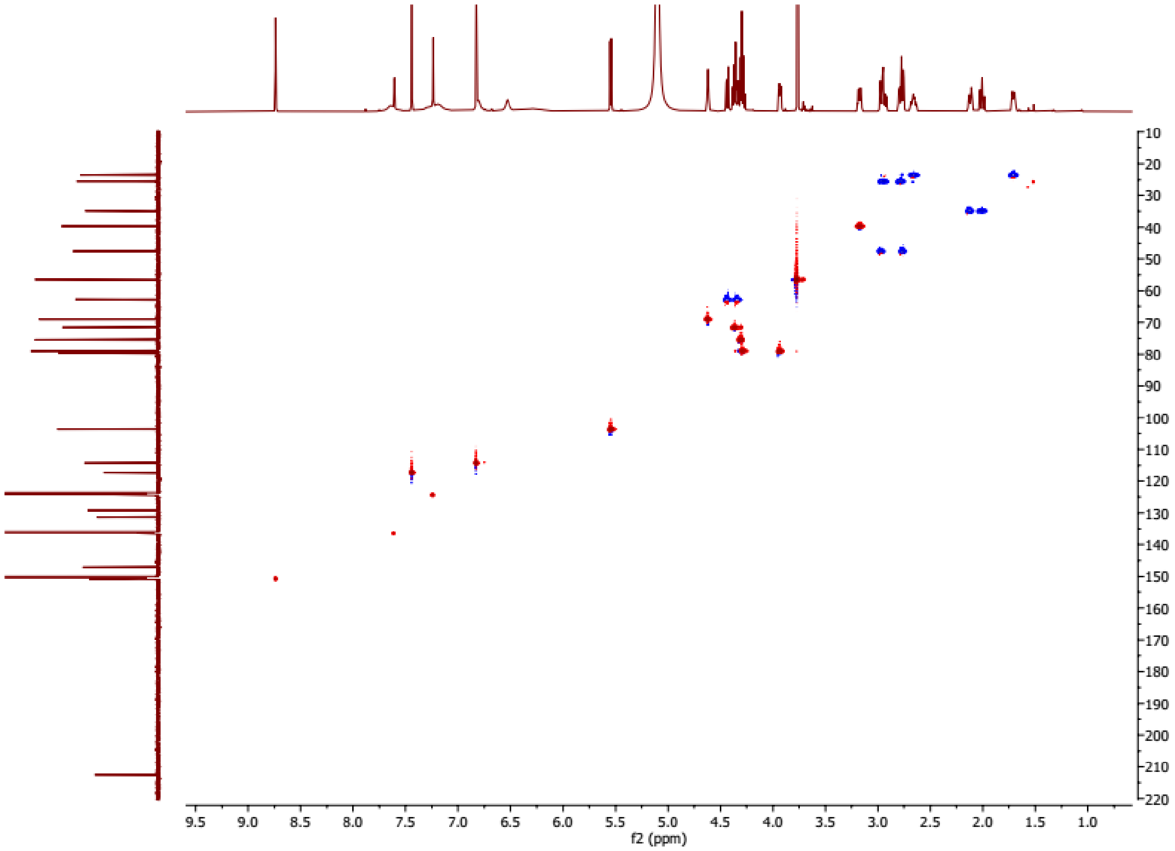

Figure S44. HMBC NMR spectrum of compound 5

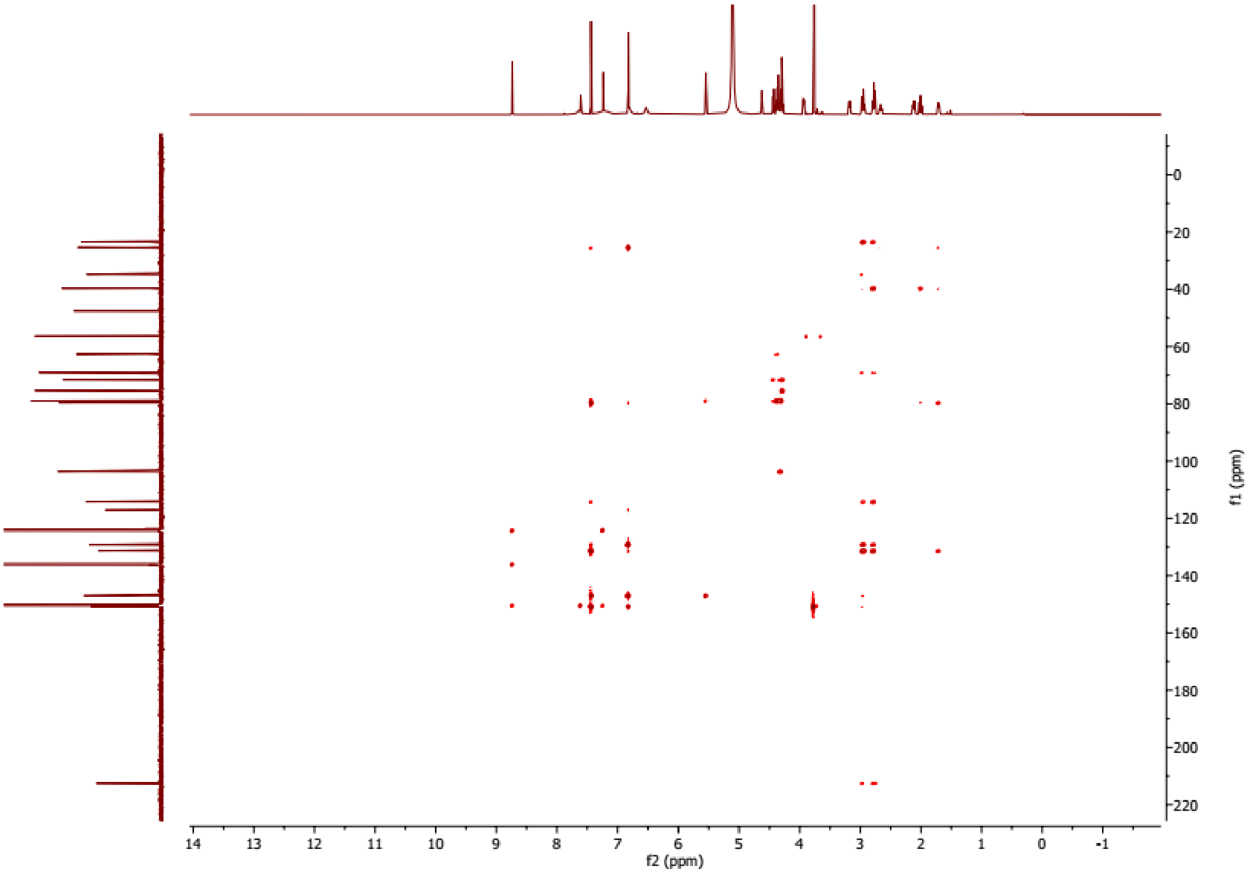

Figure S45. COSY NMR spectrum of compound 5

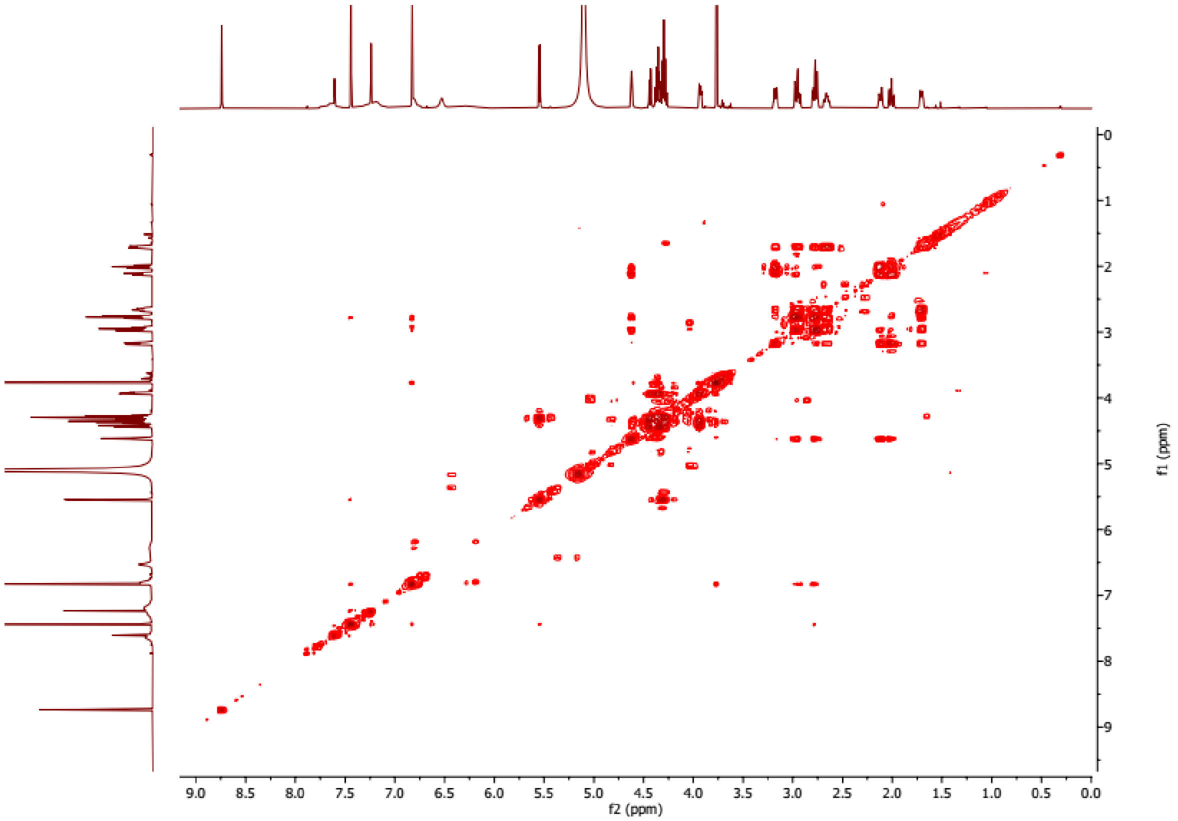

Figure S46. NOESY NMR spectrum of compound 5

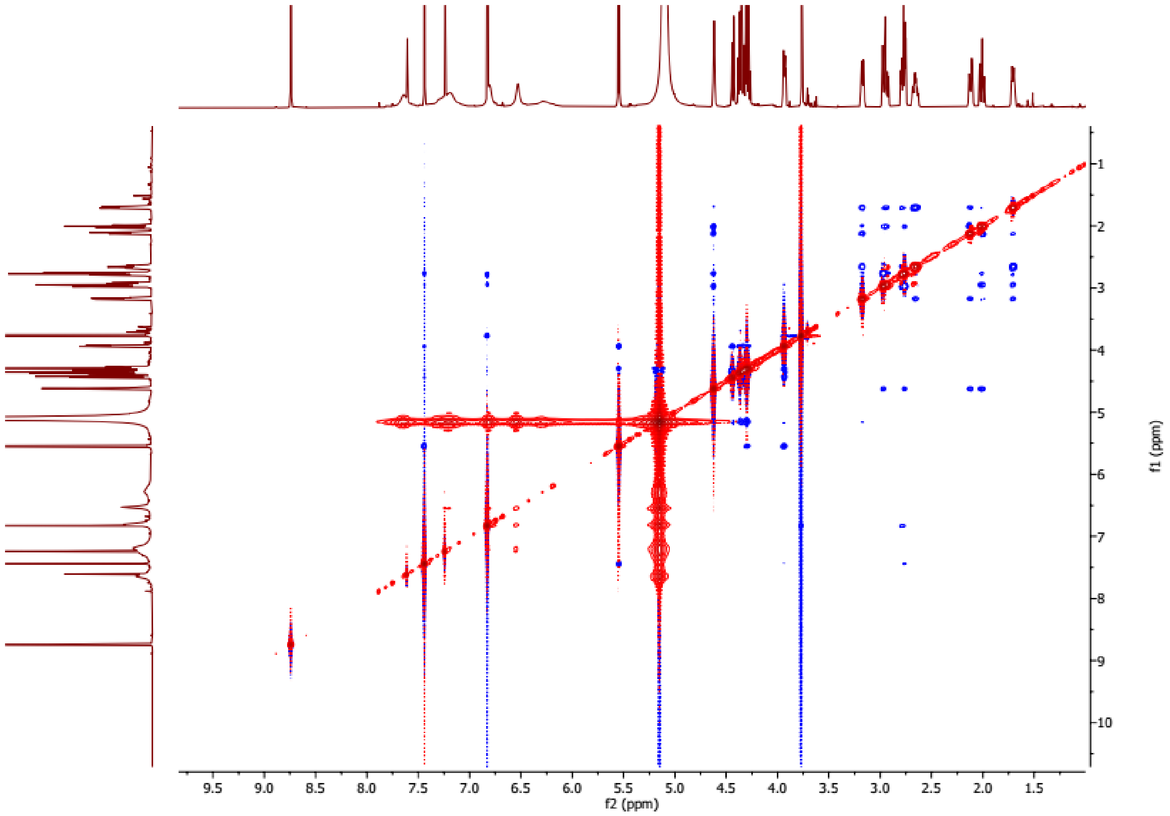

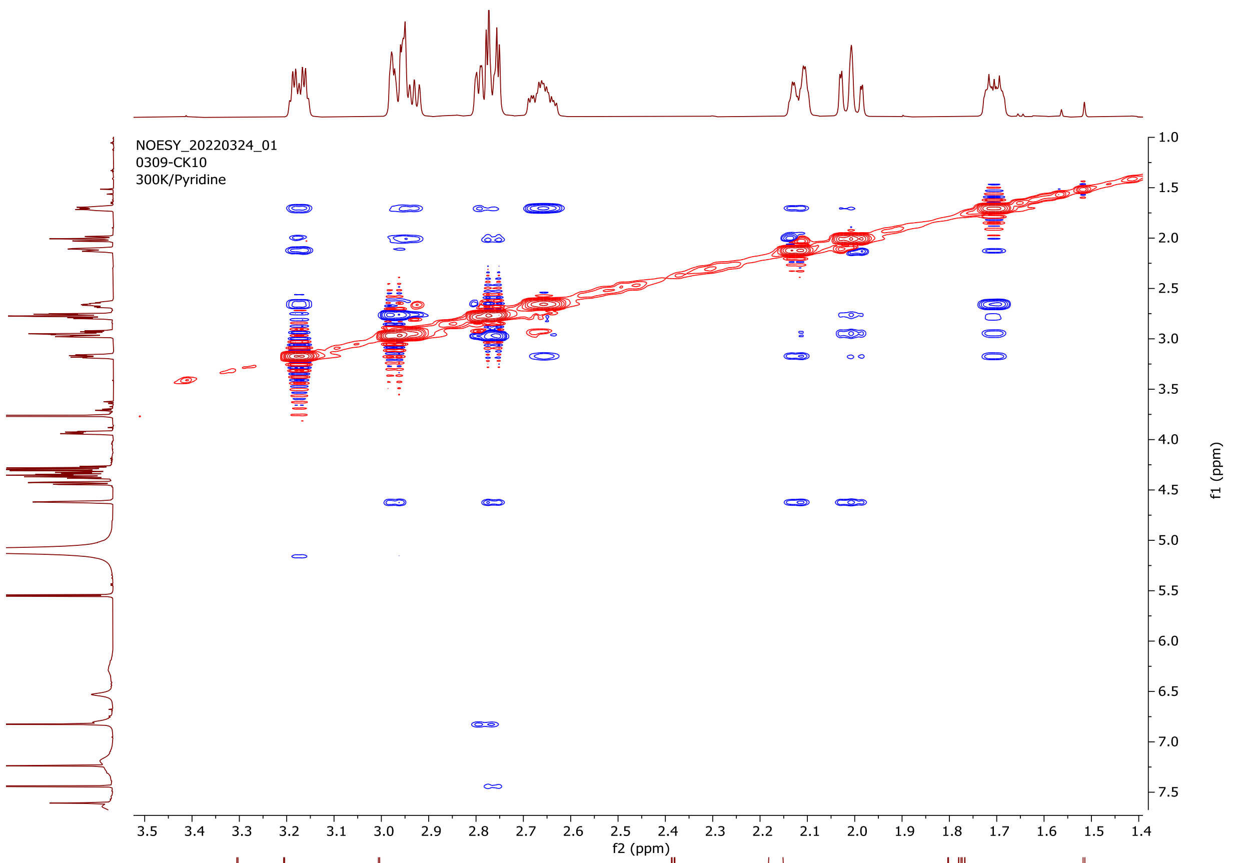
NOESY_20220324_01
0309-CK10
300K/Pyridine
f2 (ppm)
f1 (ppm)

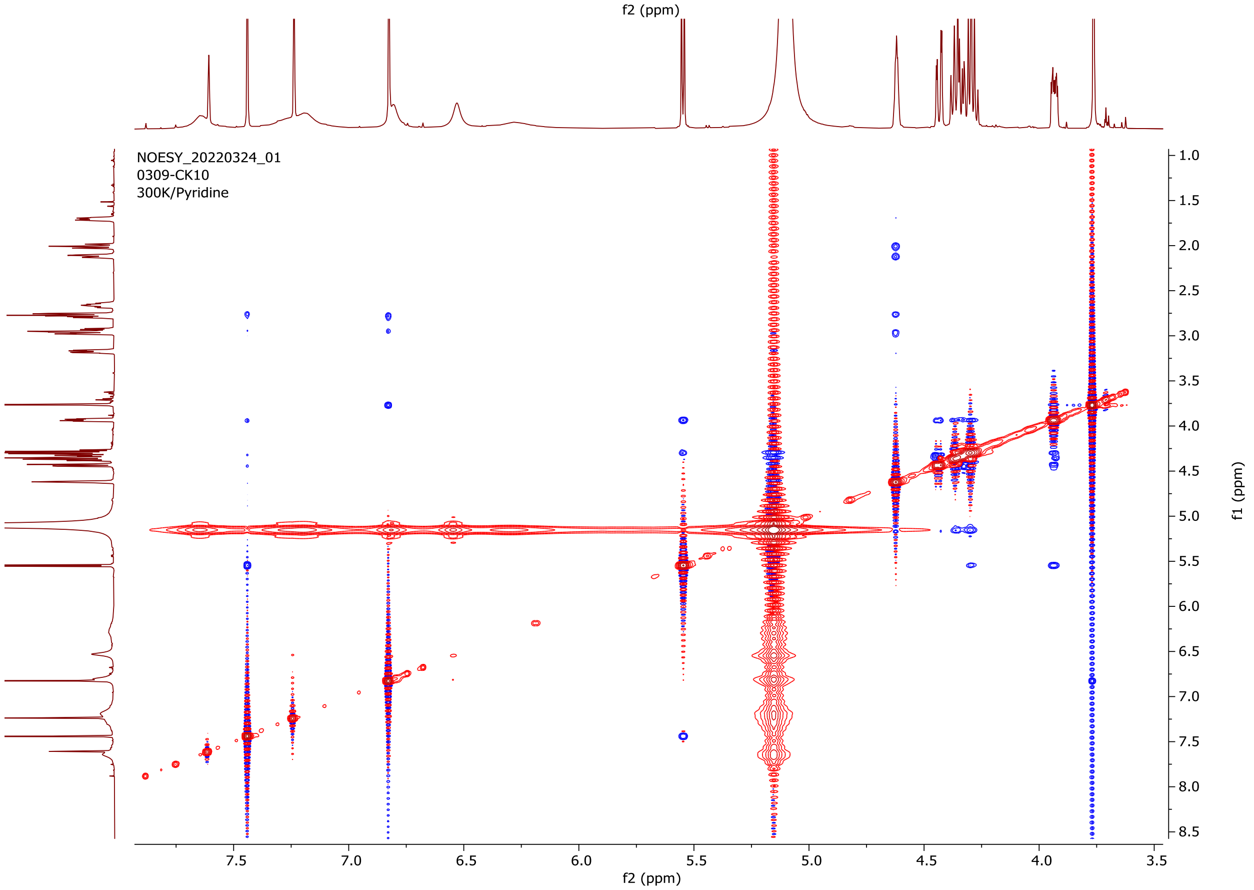
NOESY_20220324_01
0309-CK10
300K/Pyridine
f2 (ppm)
f1 (ppm)

Figure S47. HR-ESI-MS spectrum of compound 5

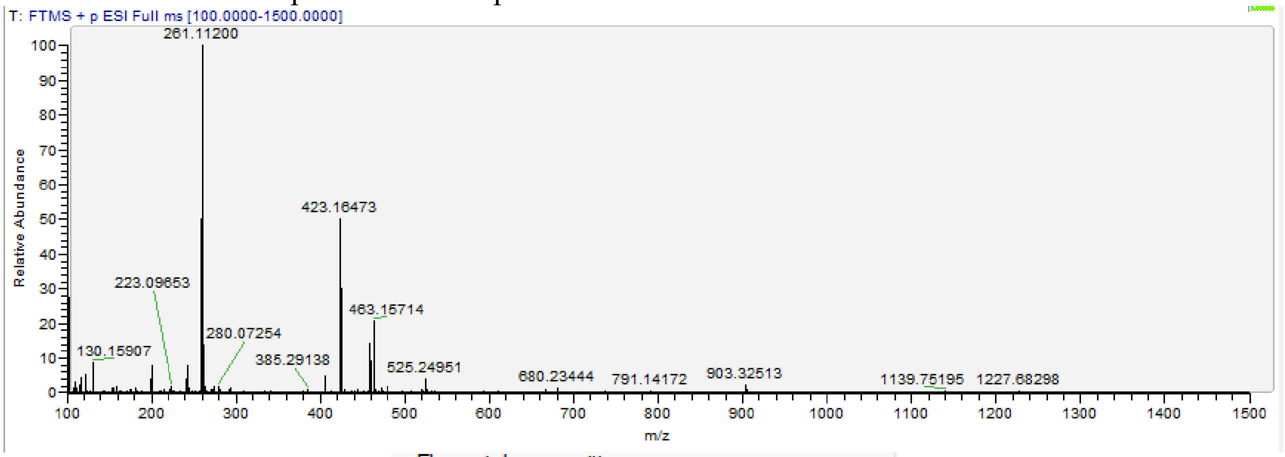

Elemental composition

Single mass

Mass: 463.15714

Max. results 10

Calculate

Idx	Formula	RDB	Delta ppm
1	$C_{21}H_{28}O_{10}Na$	7.5	-0.708

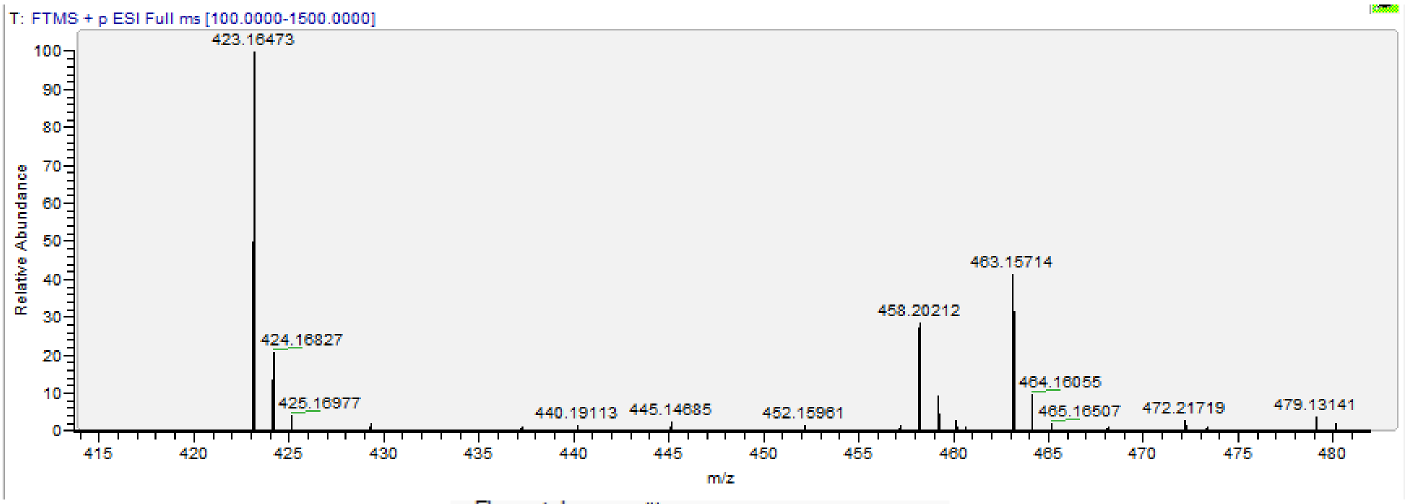

Elemental composition

Single mass

Mass: 423.16473

Max. results 10

Calculate

Idx	Formula	RDB	Delta ppm
1	$C_{21}H_{27}O_9$	8.5	-0.541

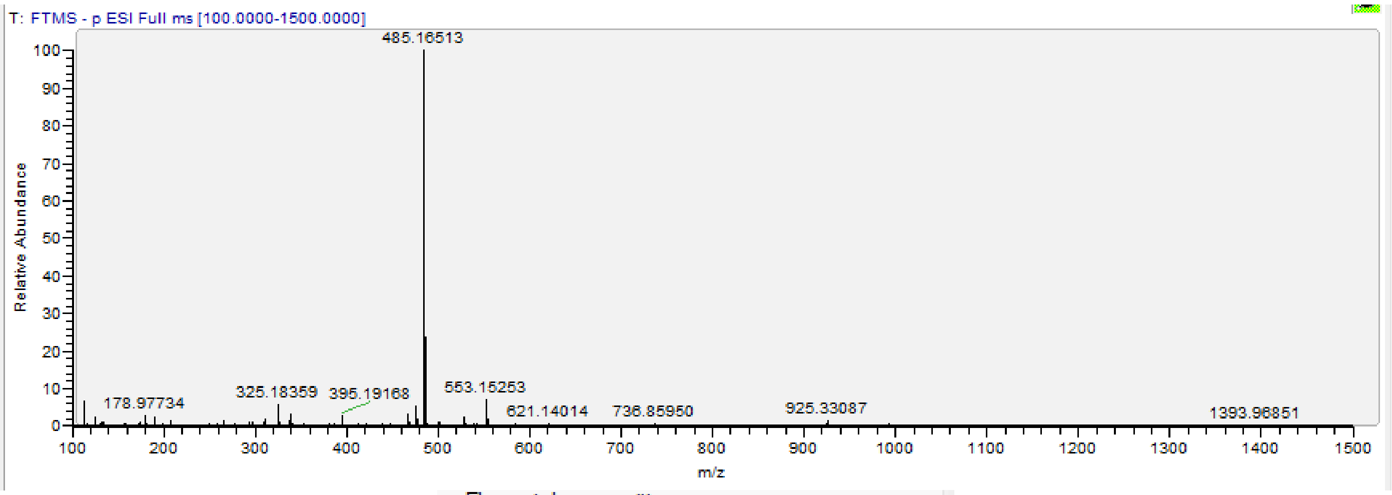

Elemental composition

Single mass

Mass: 485.16513

Max. results 10

Calculate

Idx	Formula	RDB	Delta ppm
1	$C_{22}H_{29}O_{12}$	8.5	-0.459

Figure S48. UV spectrum of compound 5

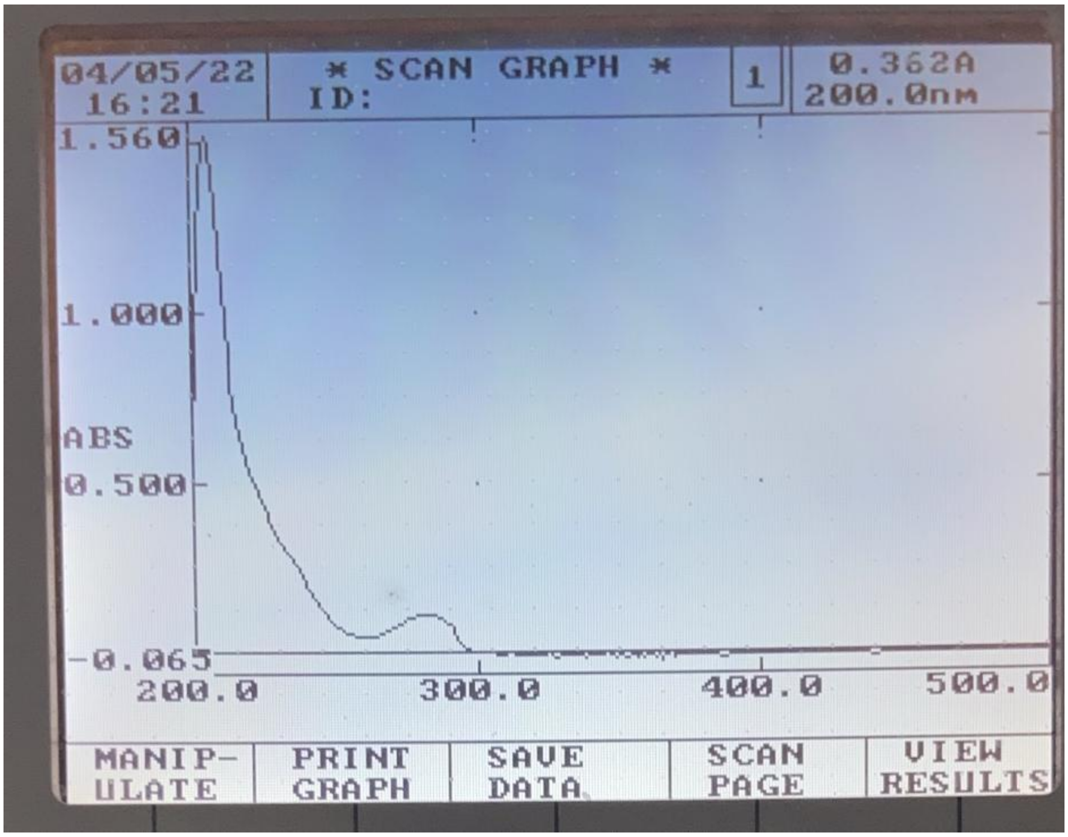

Figure S49. IR spectrum of compound 5

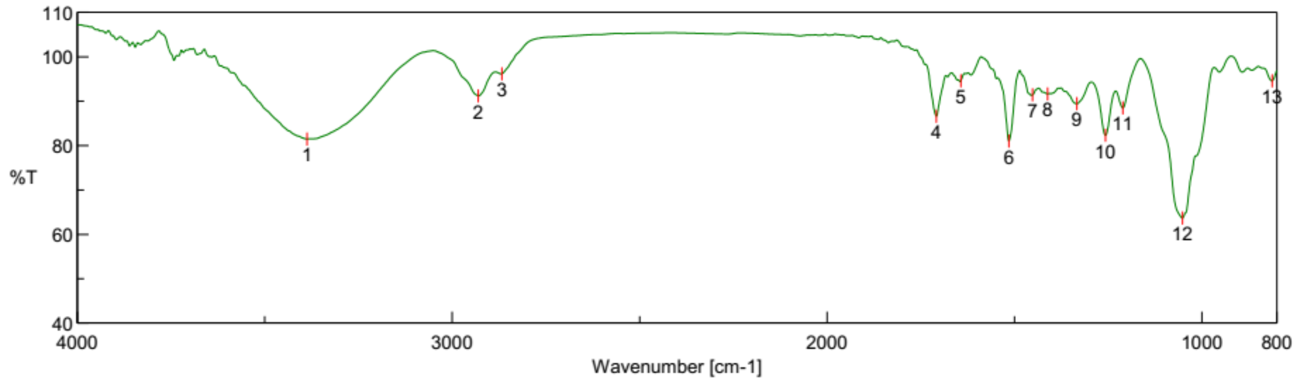

[Result of Peak Picking]

No.	Position	Intensity	No.	Position	Intensity	No.	Position	Intensity
1	3387.35	81.451	2	2930.31	91.1524	3	2867.63	96.1389
4	1708.62	86.5894	5	1643.05	94.5388	6	1514.81	80.9933
7	1452.14	91.3833	8	1411.64	91.5883	9	1333.53	89.3975
10	1257.36	82.2853	11	1211.08	88.4551	12	1051.98	63.6391
13	812.849	94.541	14	589.147	88.9637	15	486.938	61.383
16	454.154	47.0112						

Figure S50. ^{1}H-NMR spectrum of compound 6

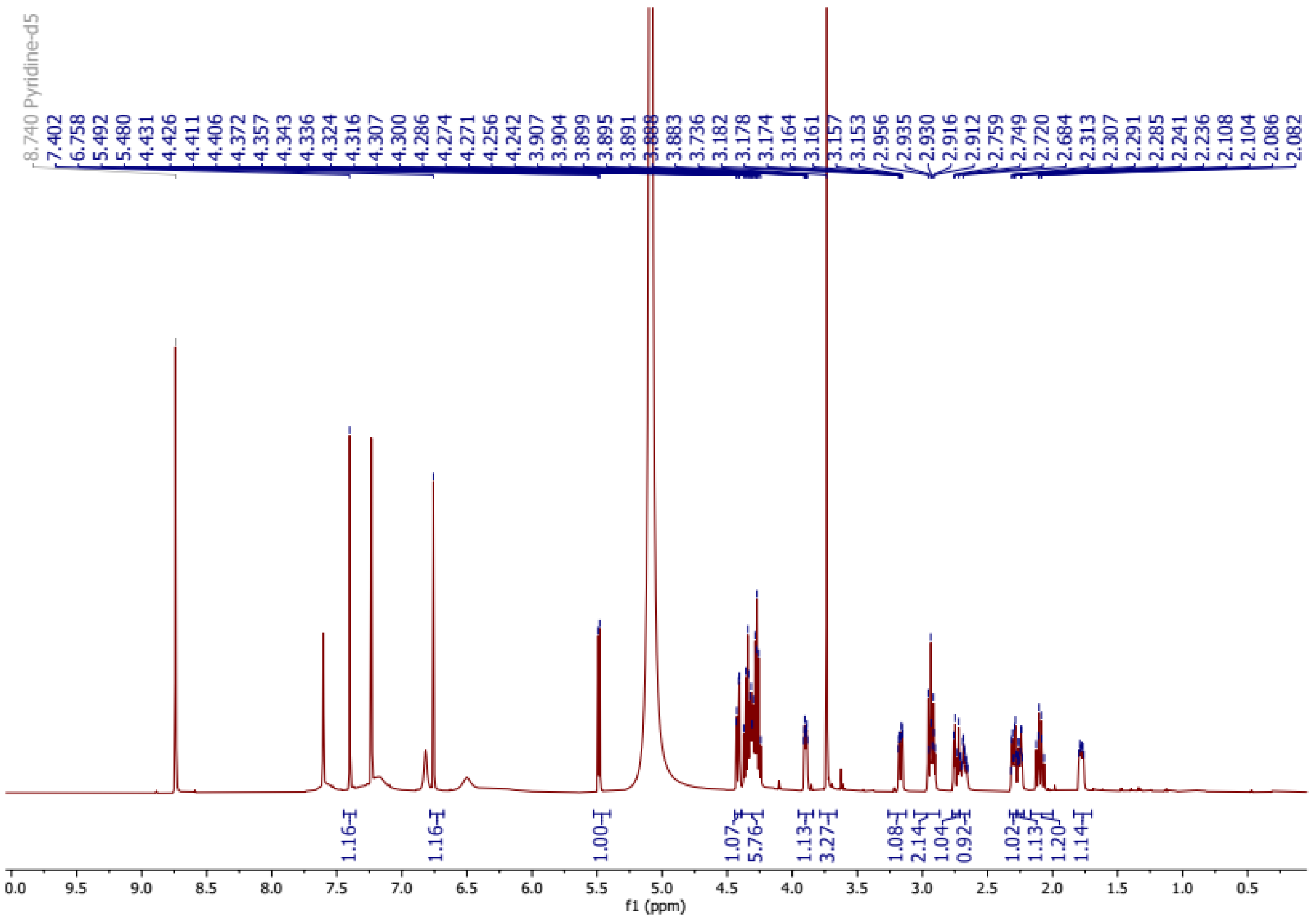

Figure S51. ^{13}C-NMR spectrum of compound 6

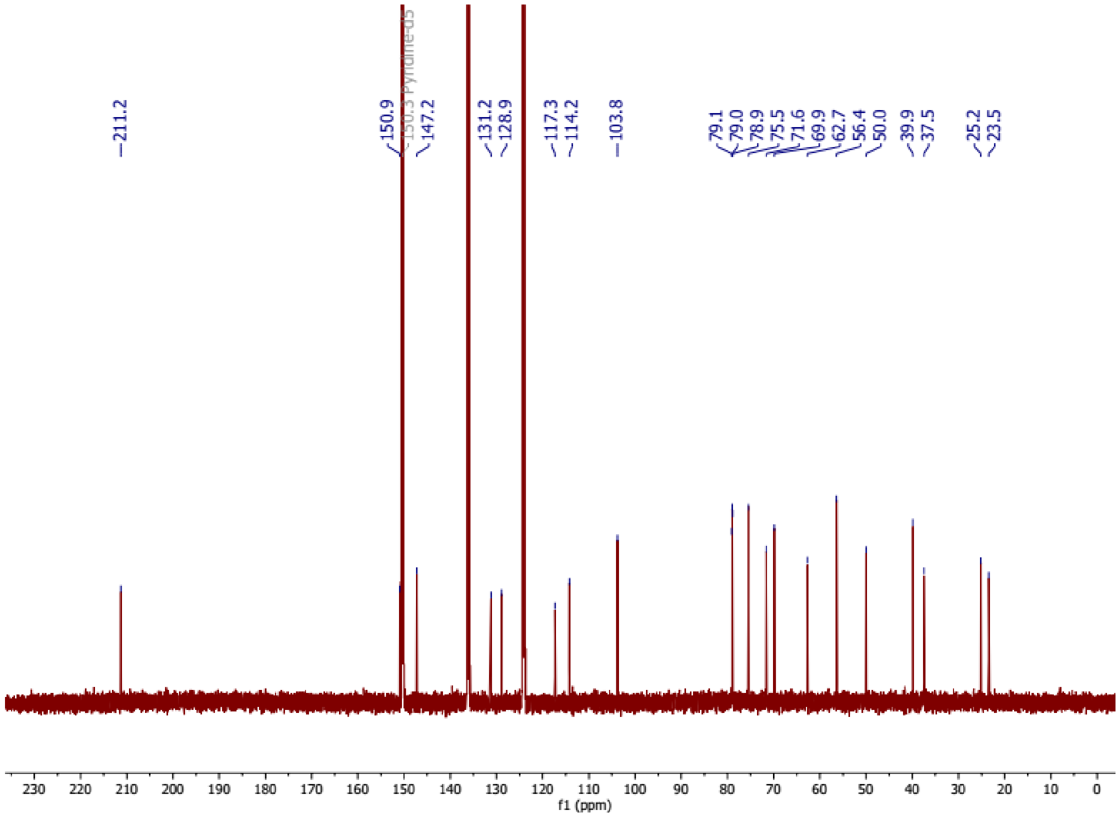

Figure S52. DEPT NMR spectrum of compound 6

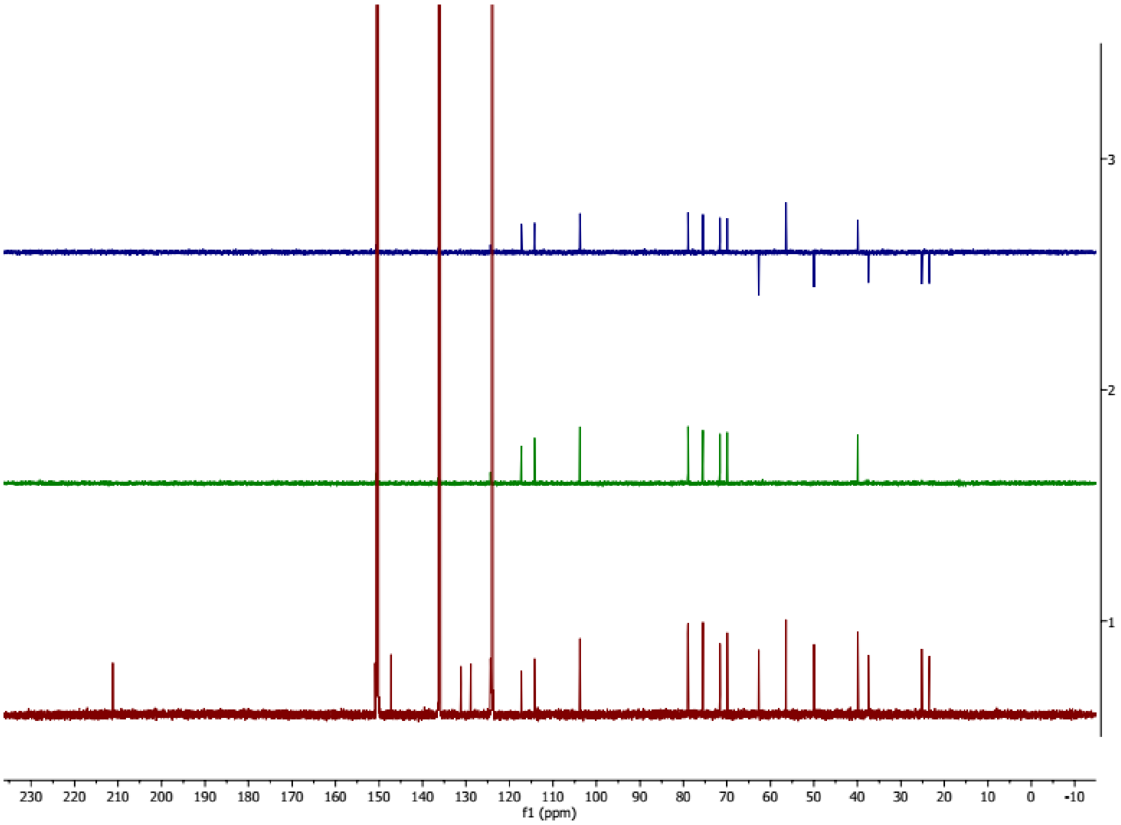

Figure S53. HSQC NMR spectrum of compound 6

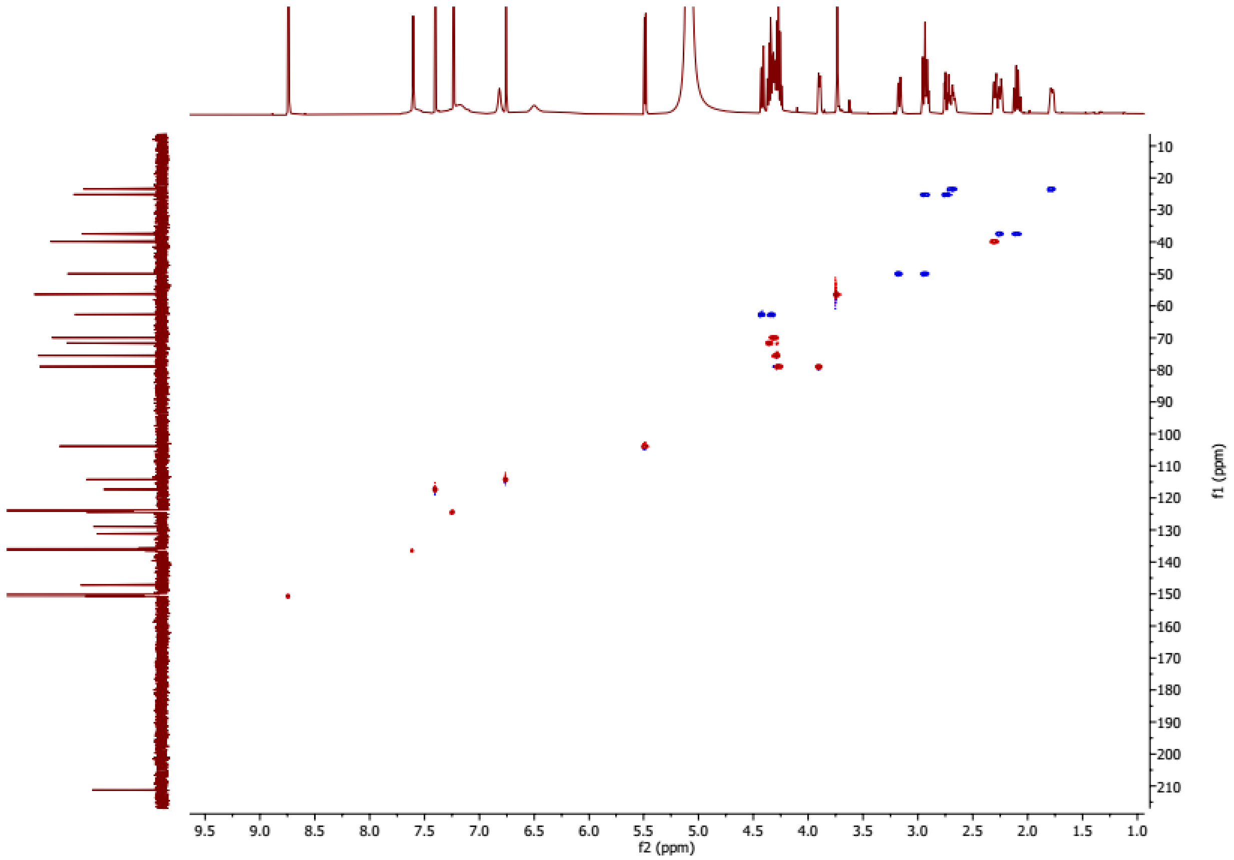

Figure S54. HMBC NMR spectrum of compound 6

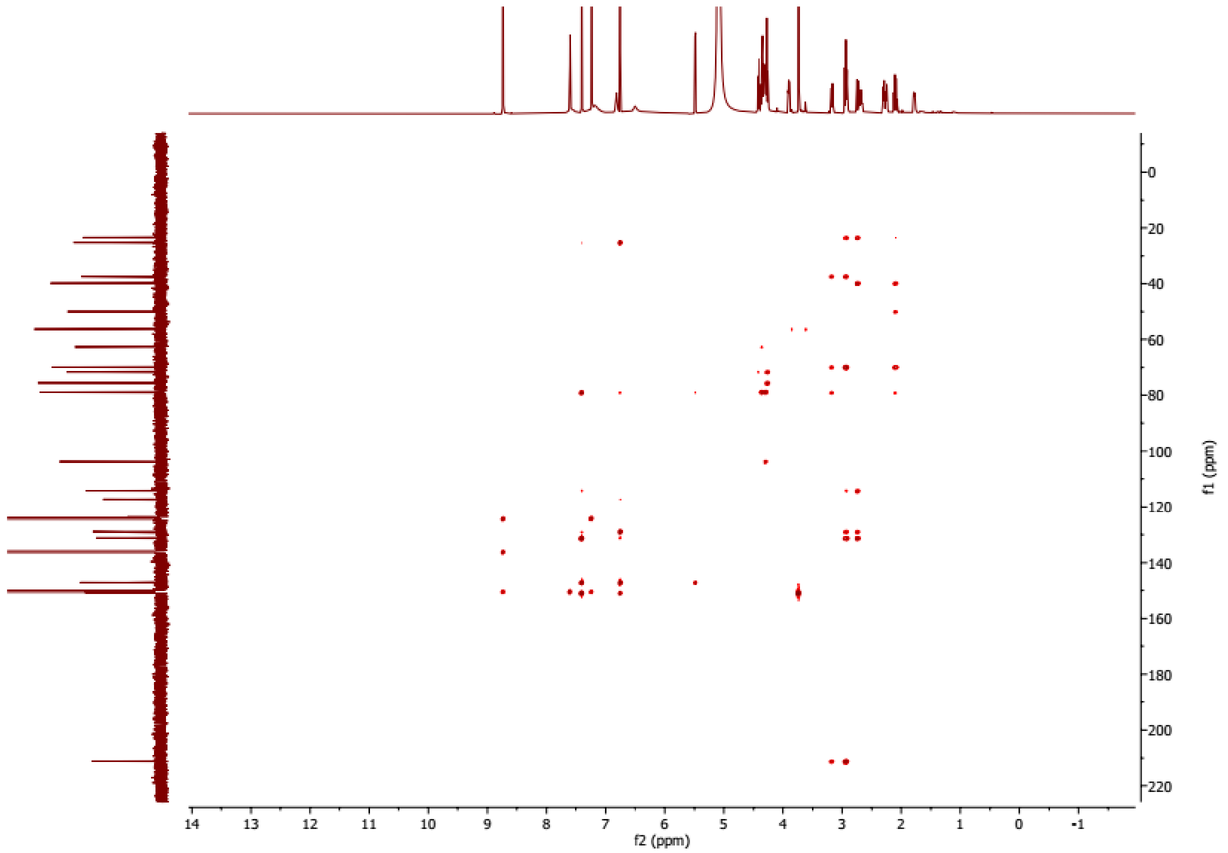

Figure S55. COSY NMR spectrum of compound 6

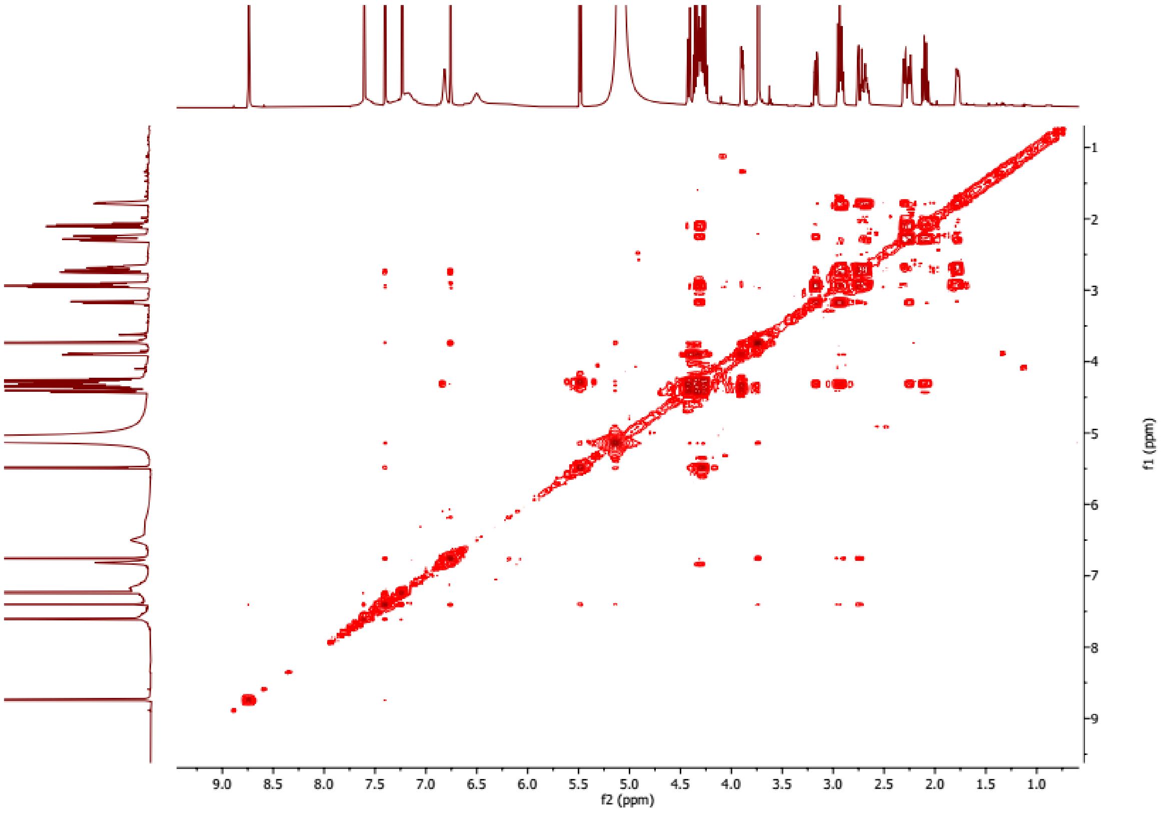

Figure S56. NOESY NMR spectrum of compound 6

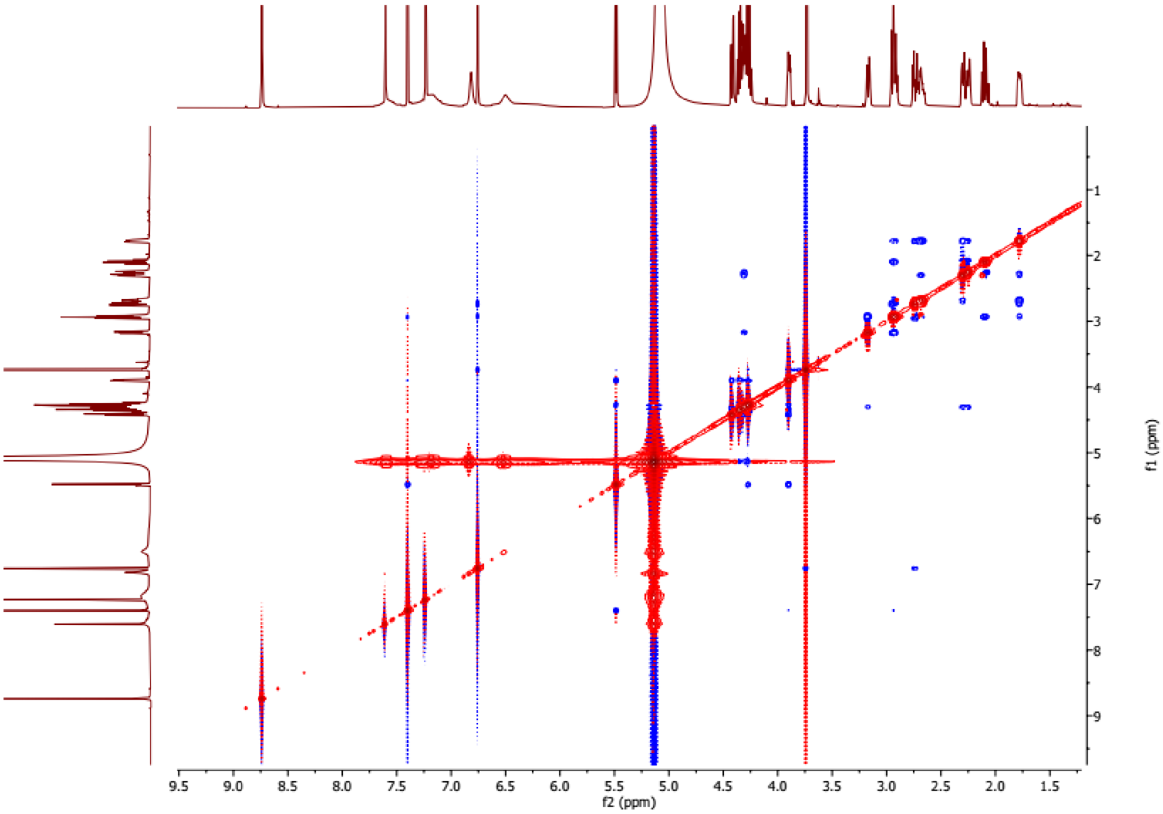

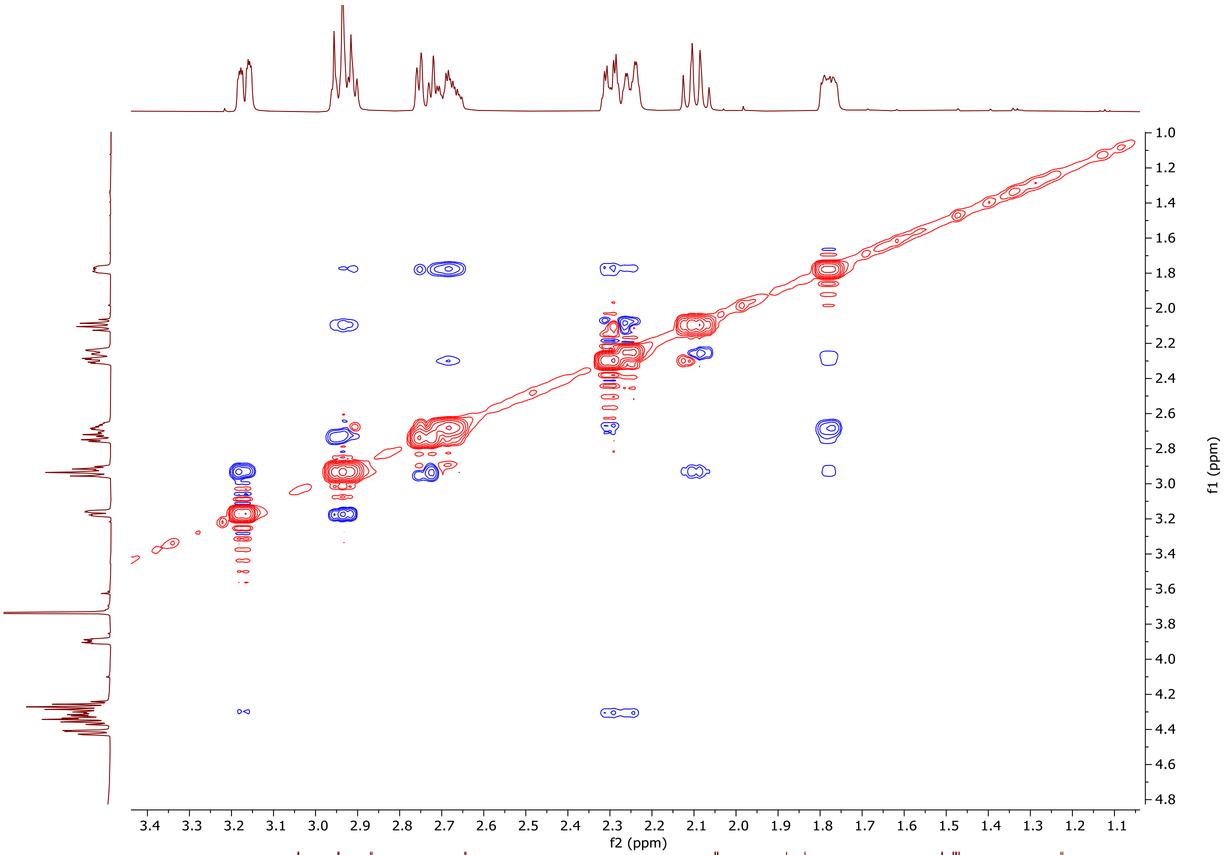
1.0 1.2 1.4 1.6 1.8 2.0 2.2 2.4 2.6 2.8 3.0 3.2 3.4 3.6 3.8 4.0 4.2 4.4 4.6 4.8
f1 (ppm)
3.4 3.3 3.2 3.1 3.0 2.9 2.8 2.7 2.6 2.5 2.4 2.3 2.2 2.1 2.0 1.9 1.8 1.7 1.6 1.5 1.4 1.3 1.2 1.1
f2 (ppm)

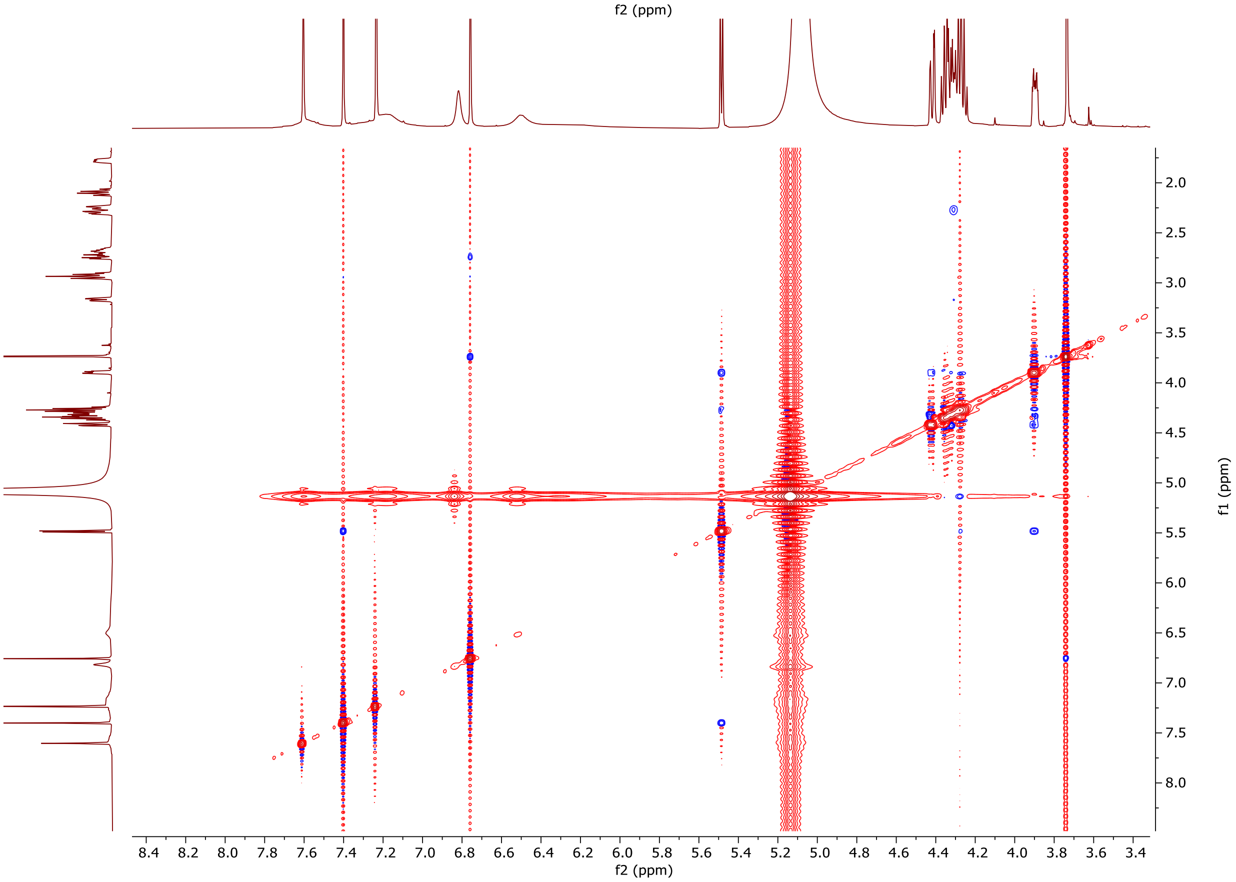
2.0 2.5 3.0 3.5 4.0 4.5 5.0 5.5 6.0 6.5 7.0 7.5 8.0
f1 (ppm)
8.4 8.2 8.0 7.8 7.6 7.4 7.2 7.0 6.8 6.6 6.4 6.2 6.0 5.8 5.6 5.4 5.2 5.0 4.8 4.6 4.4 4.2 4.0 3.8 3.6 3.4
f2 (ppm)

Figure S57. HR-ESI-MS spectrum of compound 6

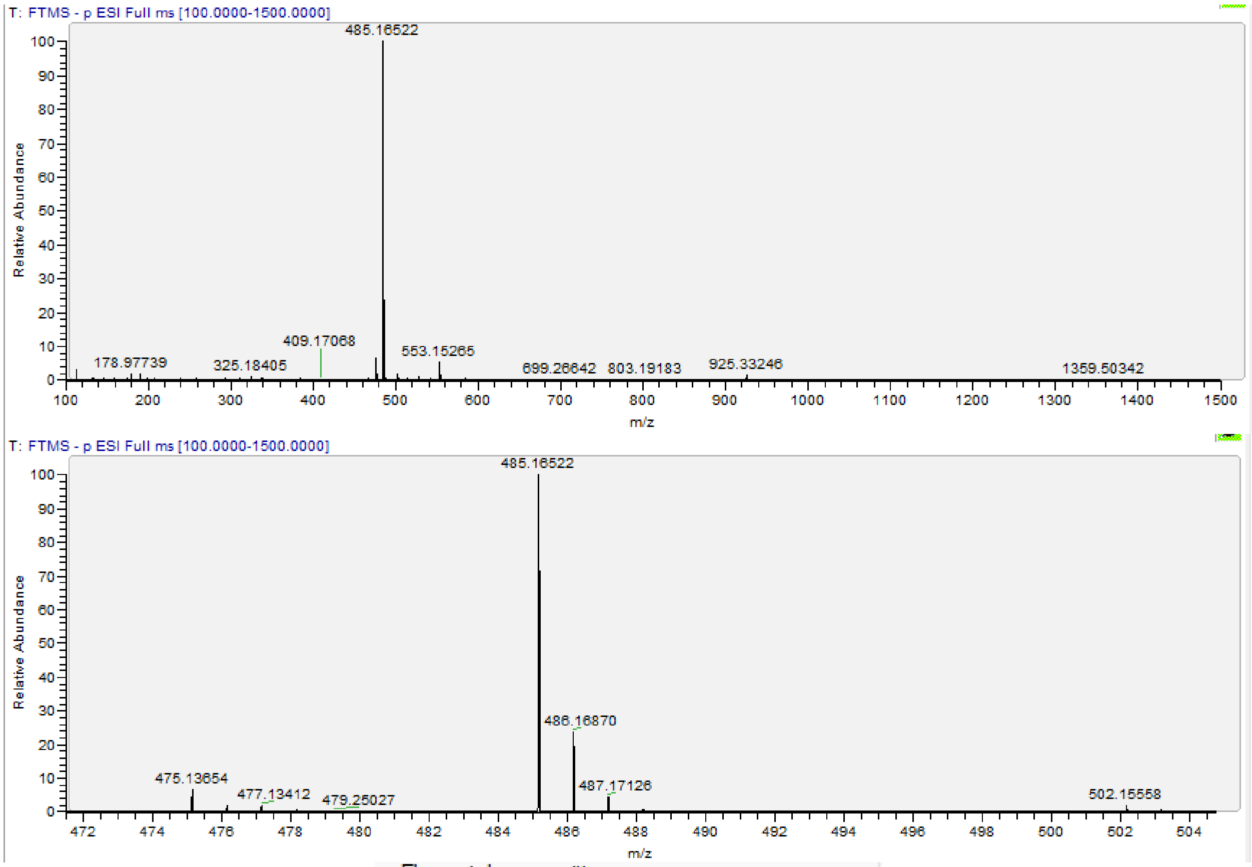

Elemental composition

Single mass

Mass: 485.16522

Max. results 10 Calculate

Idx	Formula	RDB	Delta ppm
1	$C_{22}H_{29}O_{12}$	8.5	-0.273

Figure S58. UV spectrum of compound 6

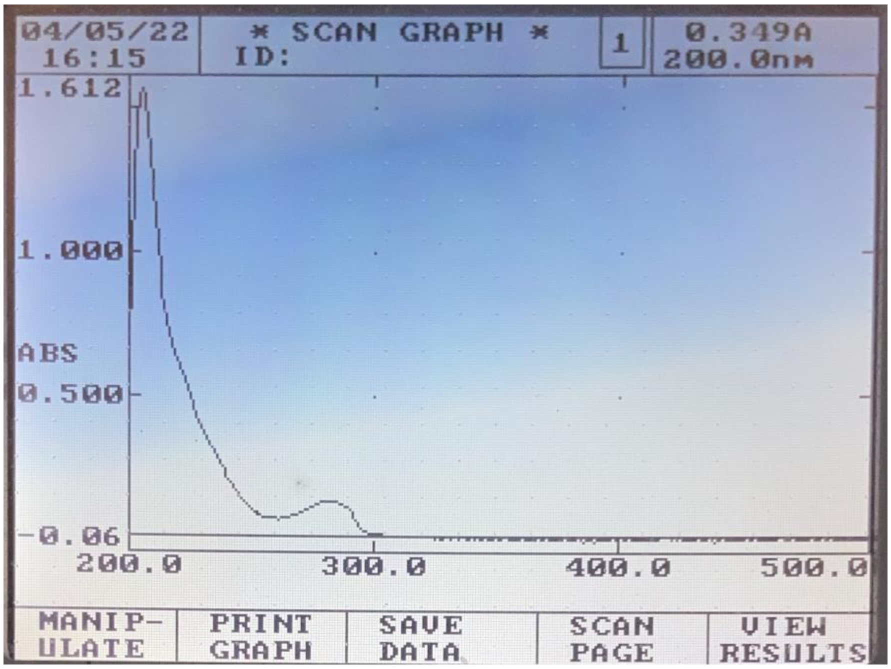

Figure S59. IR spectrum of compound 6

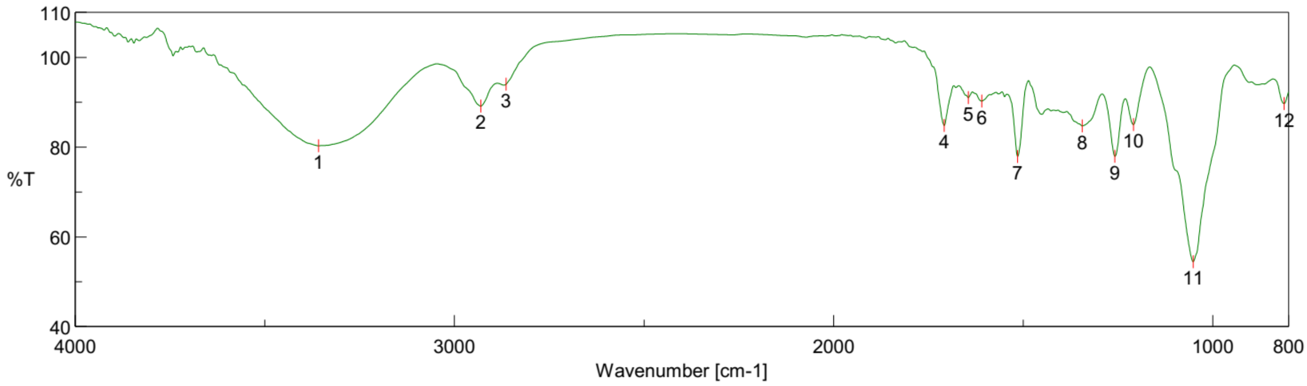

[Result of Peak Picking]

No.	Position	Intensity	No.	Position	Intensity	No.	Position	Intensity
1	3358.43	80.2595	2	2930.31	89.0845	3	2863.77	93.9723
4	1708.62	84.7836	5	1644.98	90.9692	6	1609.31	90.2271
7	1514.81	77.8697	8	1344.14	84.7083	9	1258.32	77.9366
10	1209.15	84.9743	11	1051.98	54.4407	12	811.885	89.6517
13	631.573	81.4473						

Figure S60. ^{1}H-NMR spectrum of compound 7

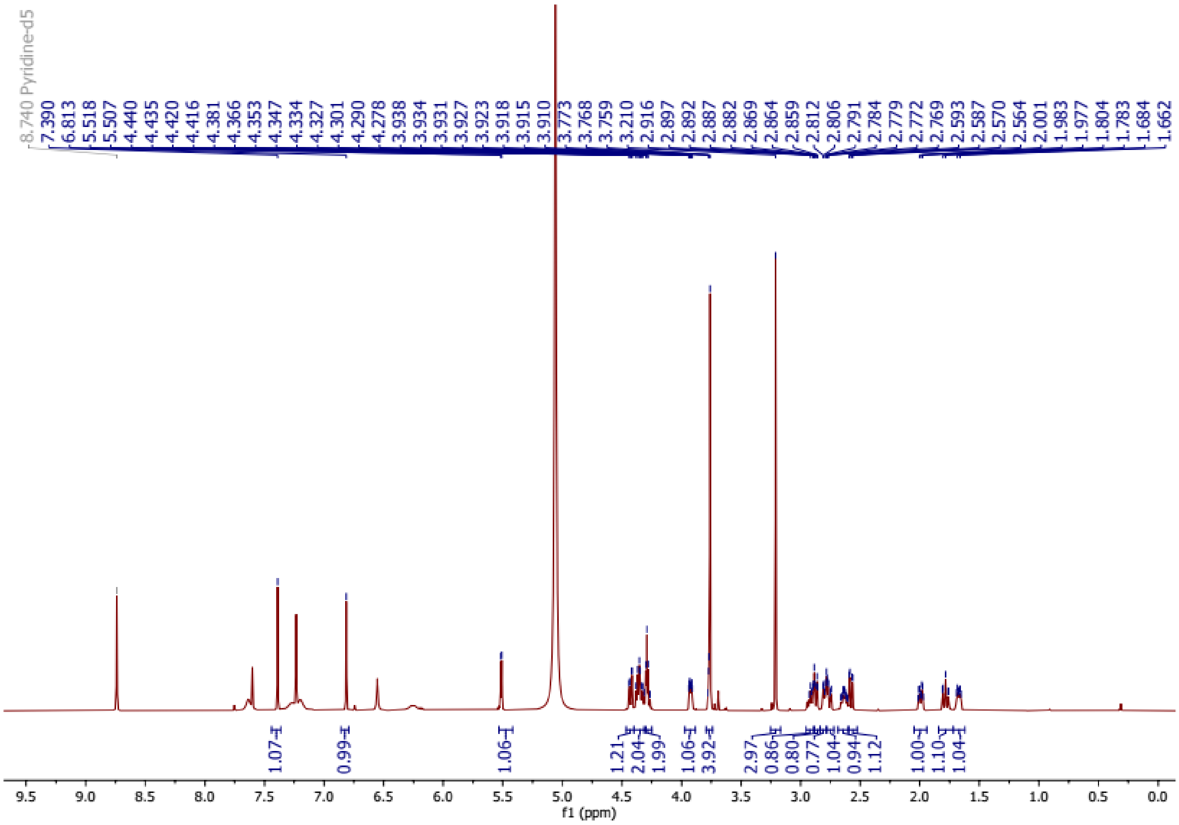

Figure S61. ^{13}C-NMR spectrum of compound 7

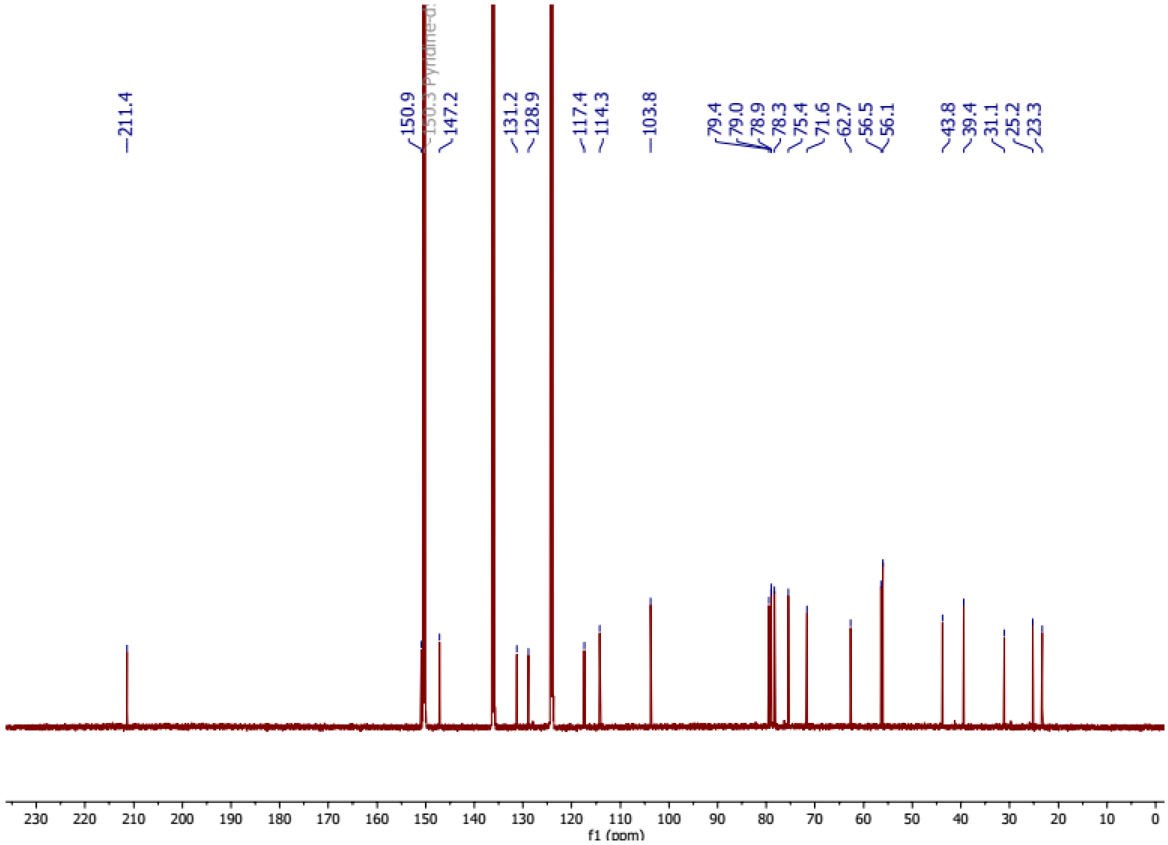

Figure S62. DEPT NMR spectrum of compound 7

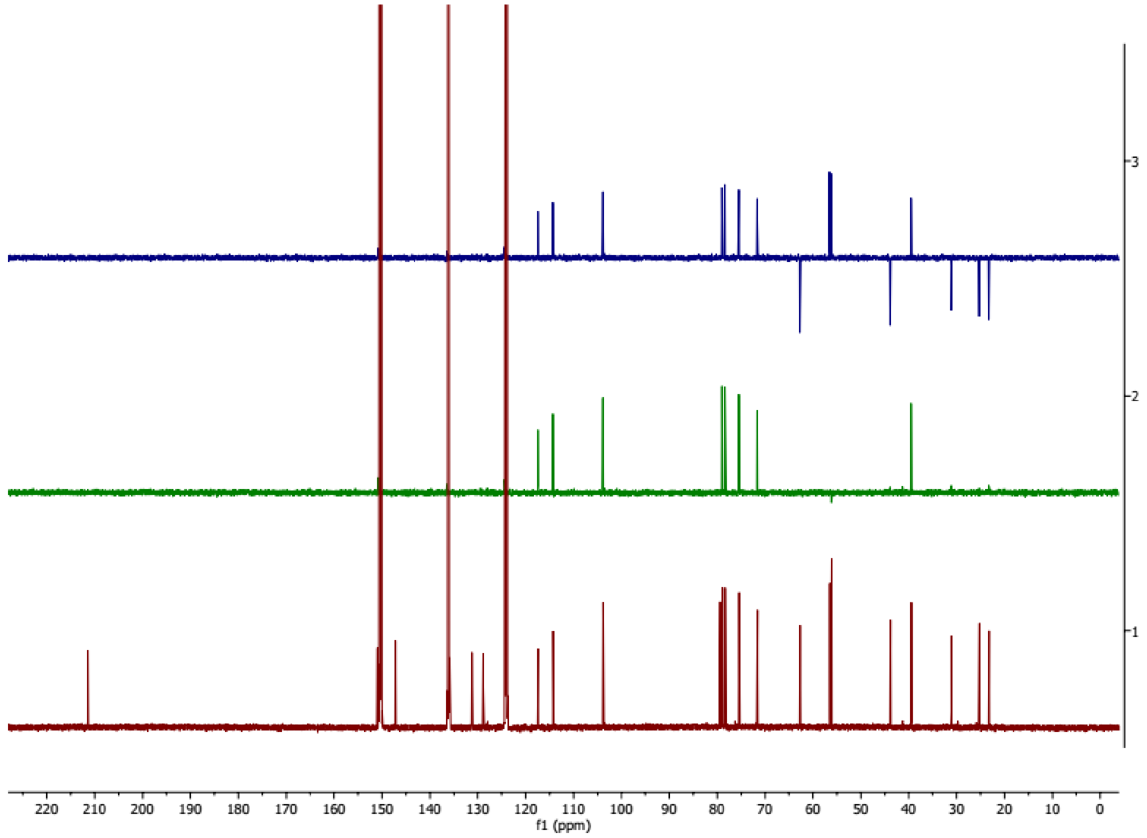

Figure S63. HSQC NMR spectrum of compound 7

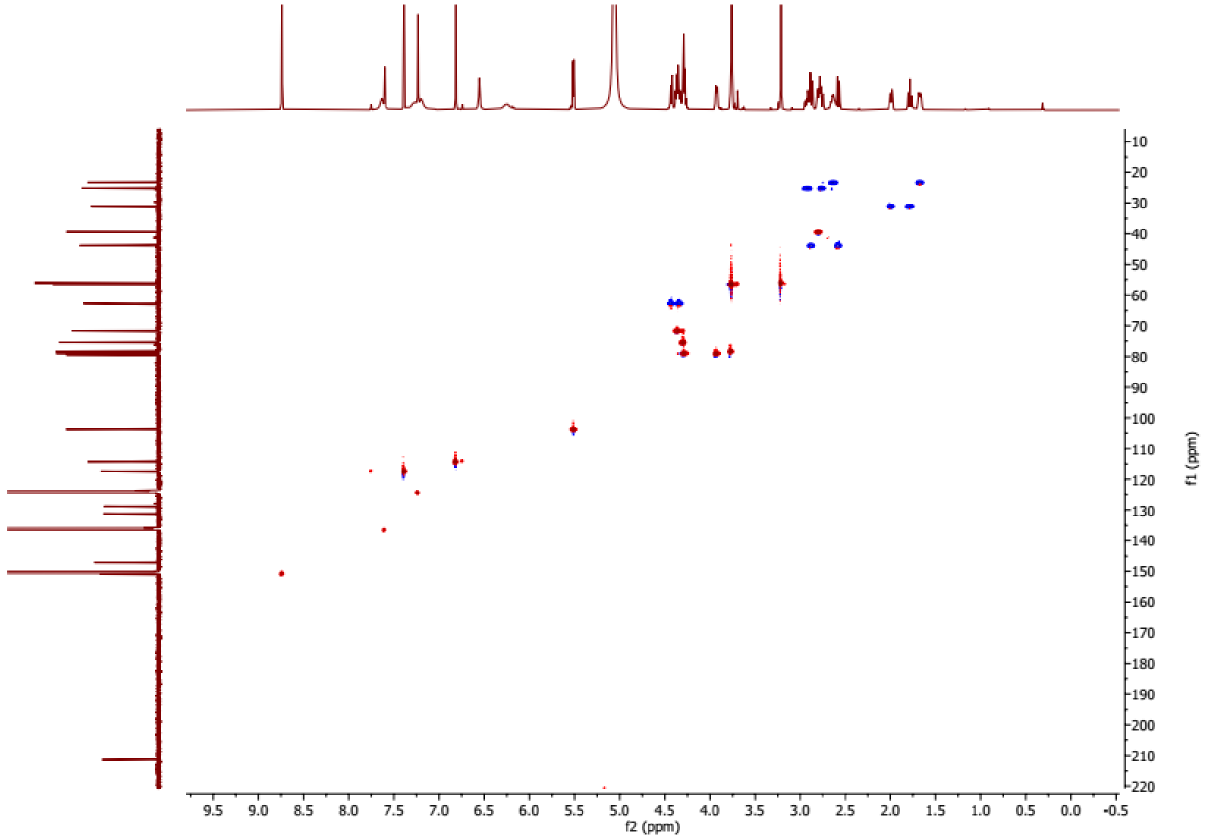

Figure S64. HMBC NMR spectrum of compound 7

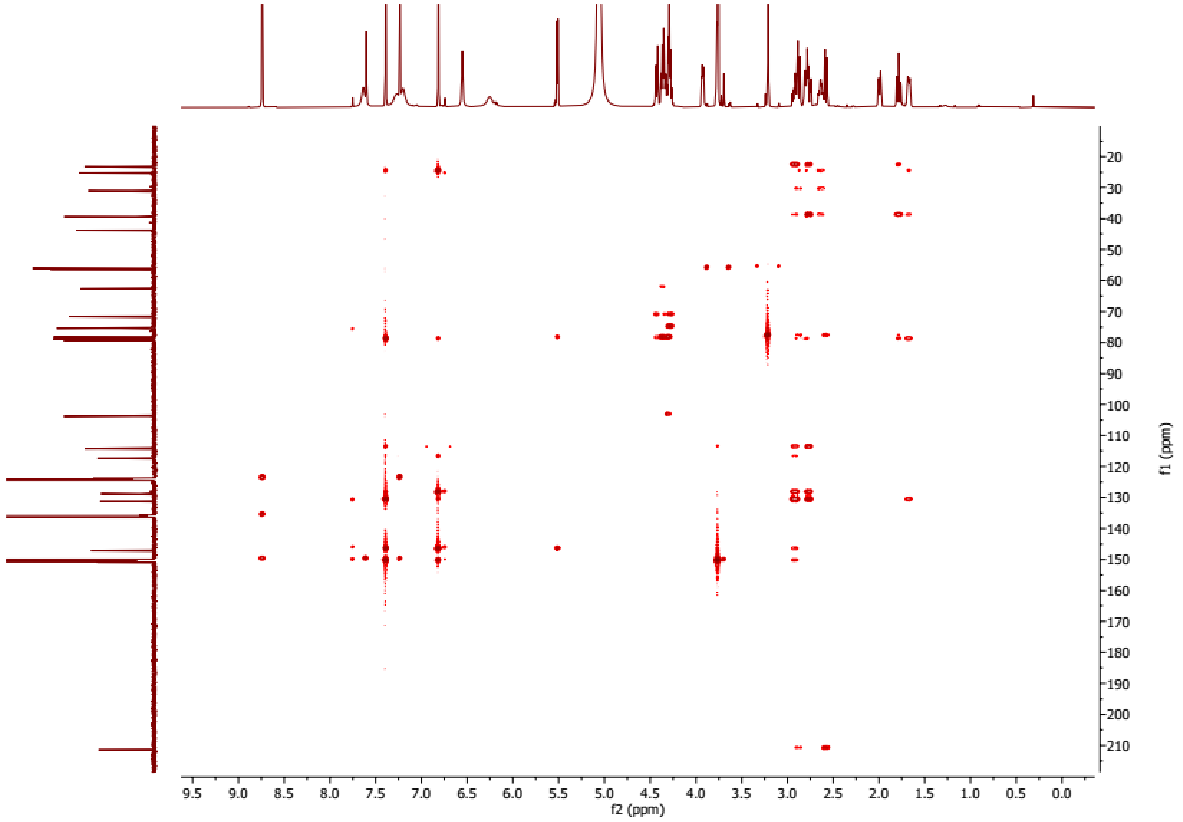

Figure S65. COSY NMR spectrum of compound 7

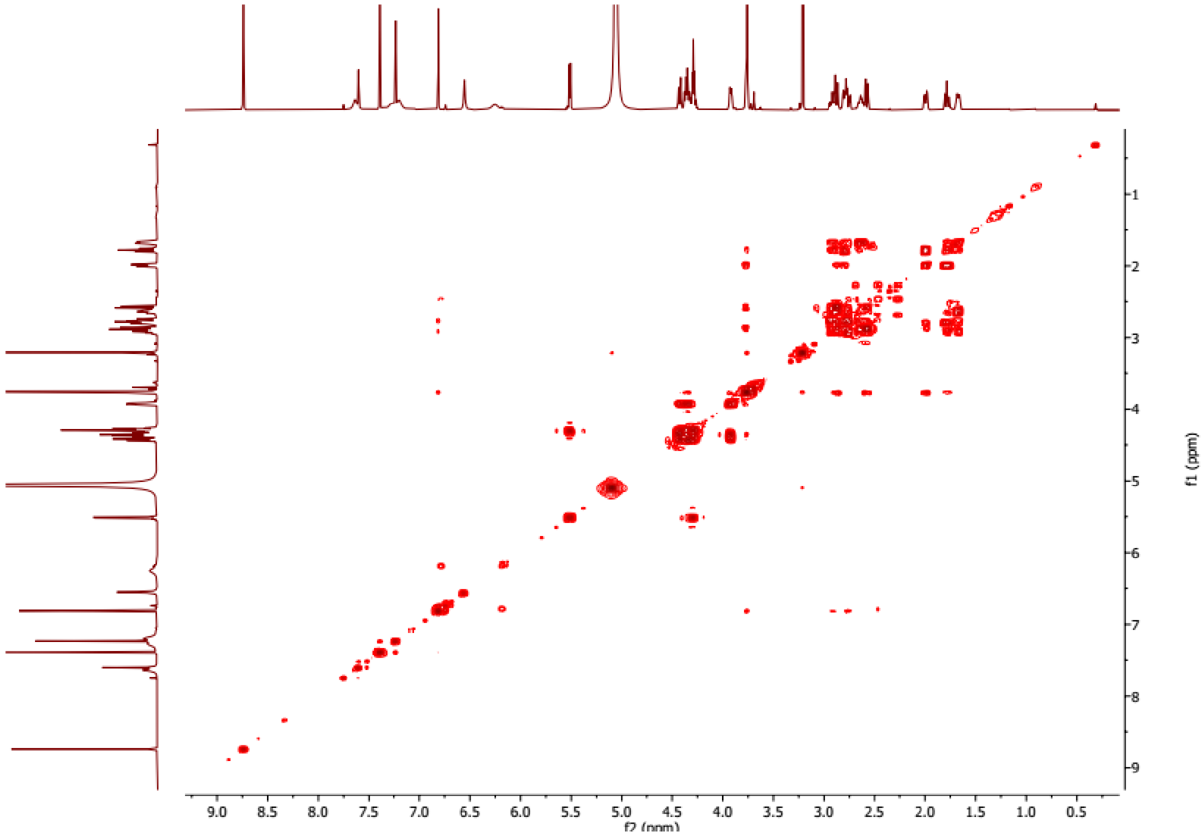

Figure S66. NOESY NMR spectrum of compound 7

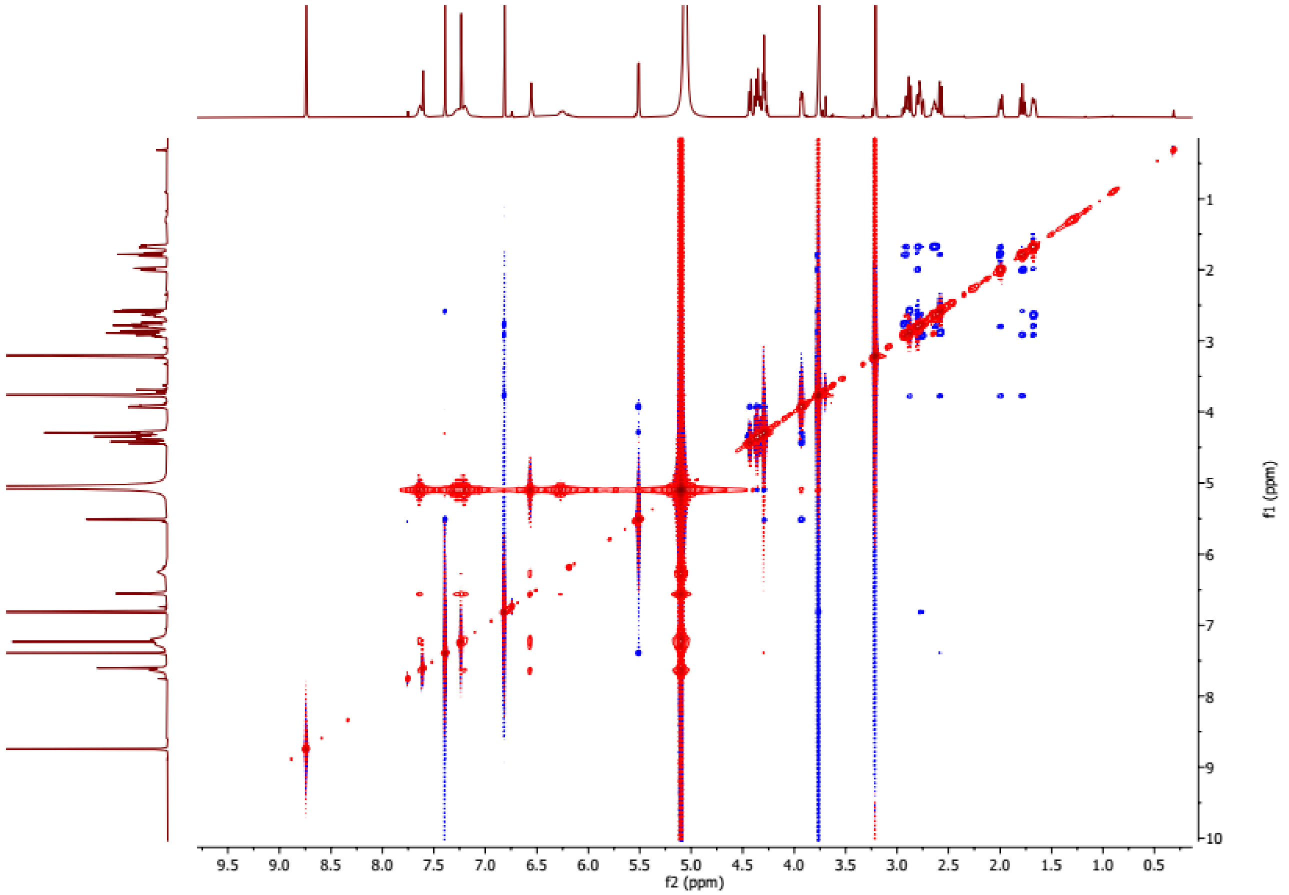

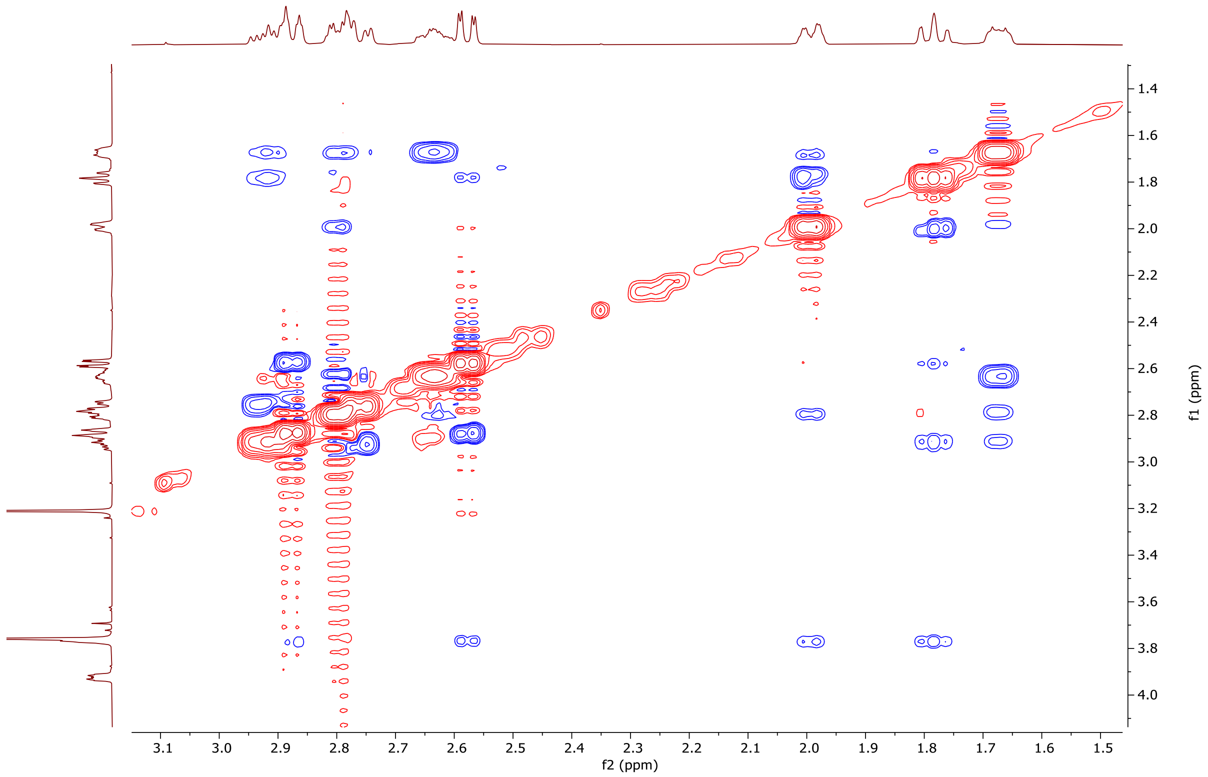

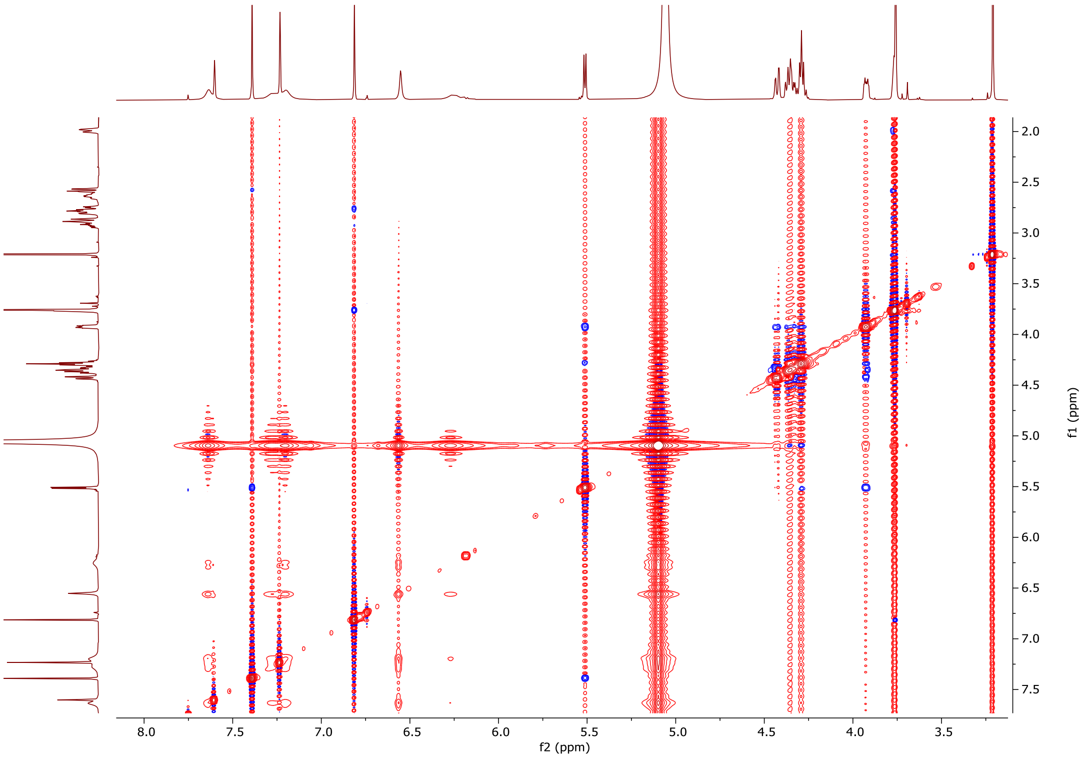

Figure S67. HR-ESI-MS spectrum of compound 7

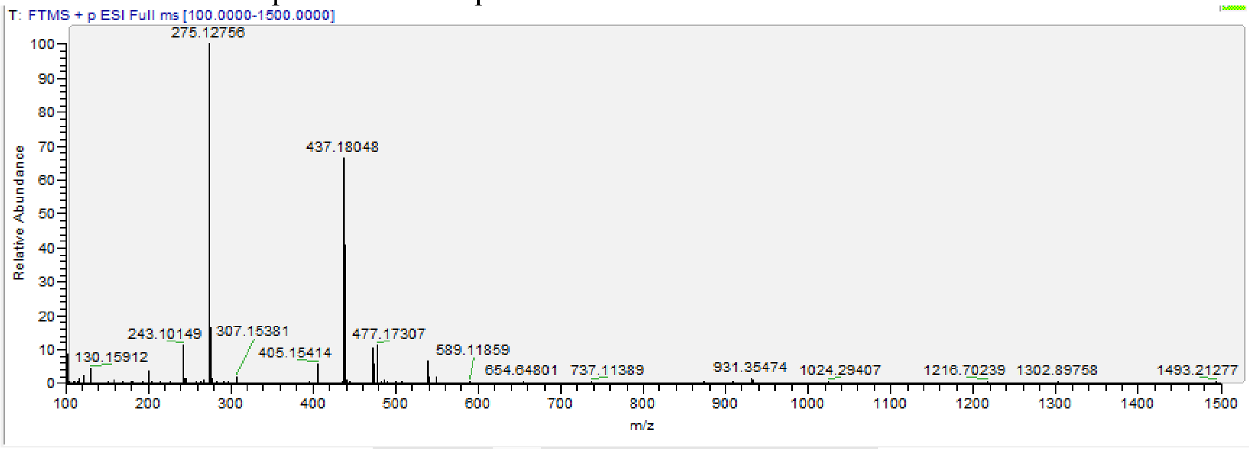

Elemental composition

Single mass

Mass: 477.17307

Max. results 10

Calculate

Idx	Formula	RDB	Delta ppm
1	$C_{22}H_{30}O_{10}Na$	7.5	-0.101

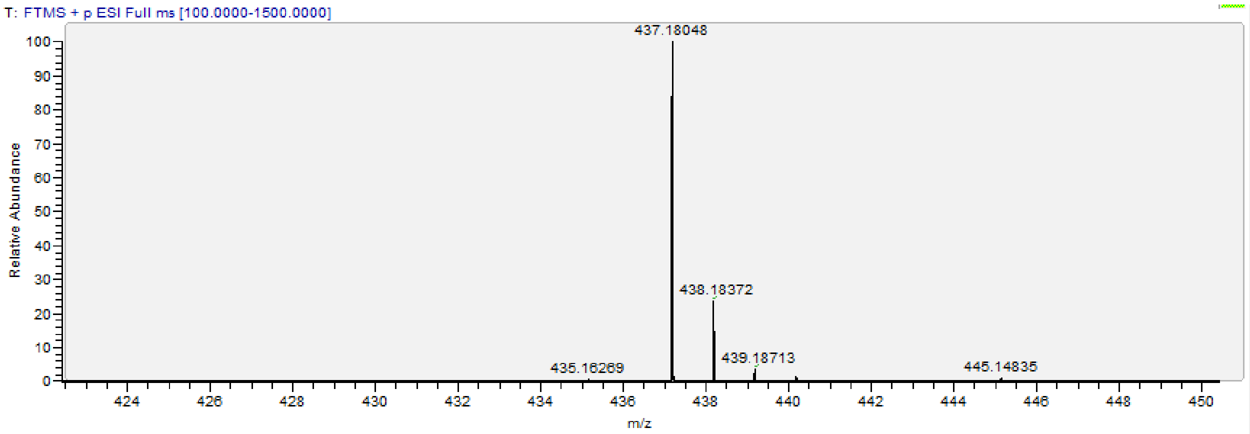

Elemental composition

Single mass

Mass: 437.18048

Max. results 10

Calculate

Idx	Formula	RDB	Delta ppm
1	$C_{22}H_{29}O_9$	8.5	-0.295

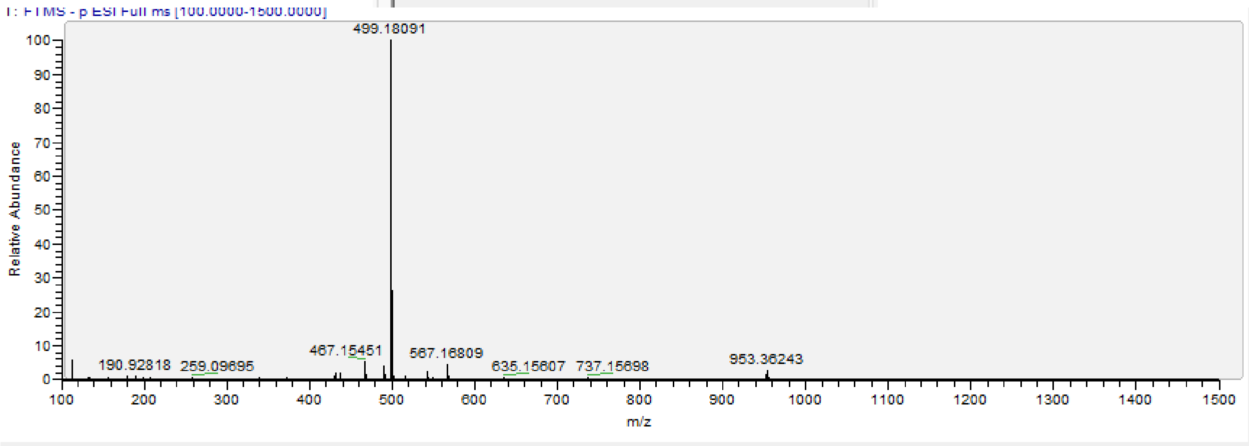

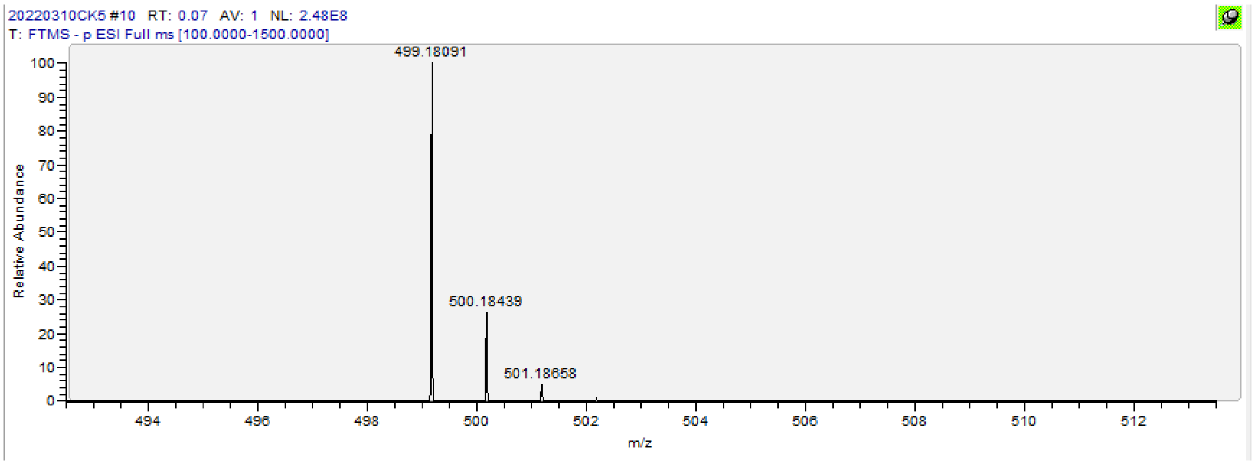

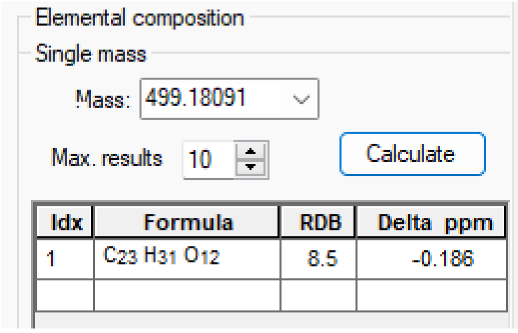

Figure S68. UV spectrum of compound 7

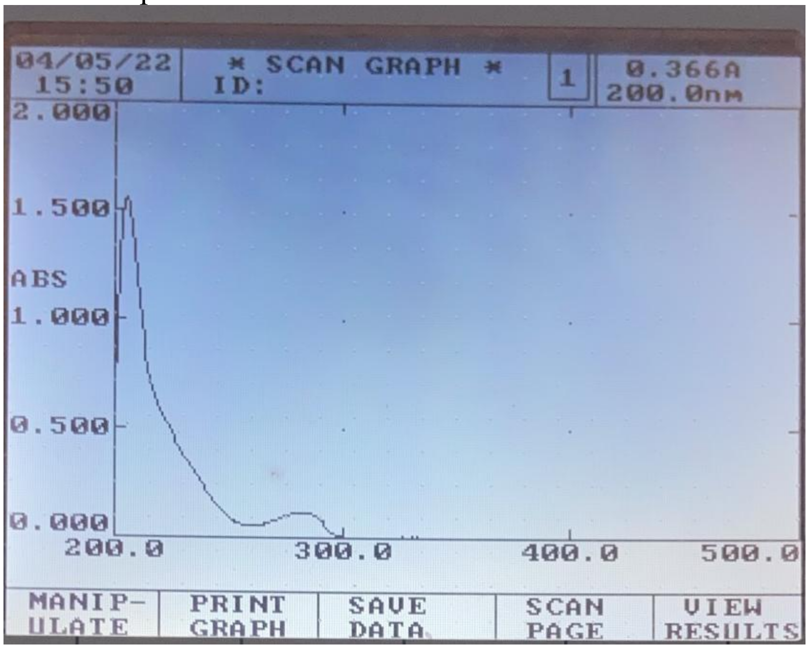

Figure S69. IR spectrum of compound 7

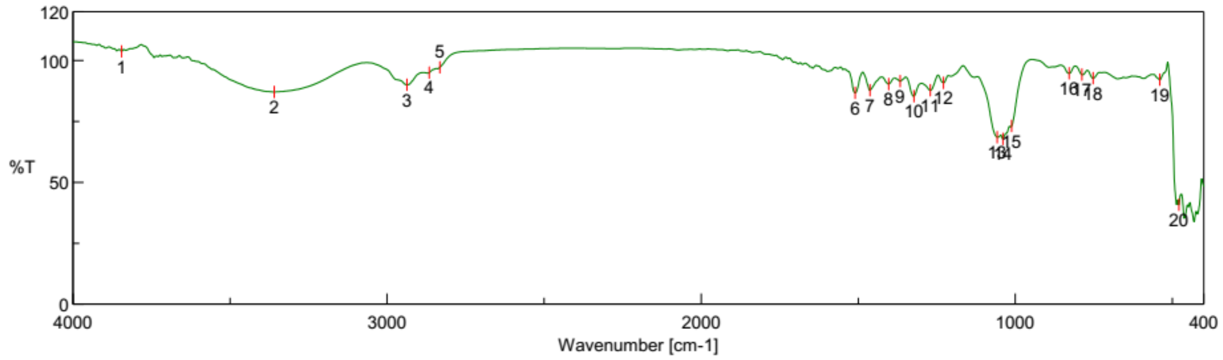

[Result of Peak Picking]

No.	Position	Intensity	No.	Position	Intensity	No.	Position	Intensity
1	3845.36	103.721	2	3359.39	87.0457	3	2937.06	90.0537
4	2865.7	94.9553	5	2831.95	97.2086	6	1509.99	86.7484
7	1462.74	87.7539	8	1402.96	90.294	9	1366.32	91.4898
10	1322.93	85.278	11	1270.86	87.7599	12	1228.43	90.842
13	1057.76	68.5196	14	1039.44	67.8312	15	1011.48	73.0801
16	828.277	94.6972	17	787.779	94.162	18	752.102	92.7154
19	539.971	92.078	20	479.224	40.7483			

Figure S70. ^{1}H-NMR spectrum of compound 8

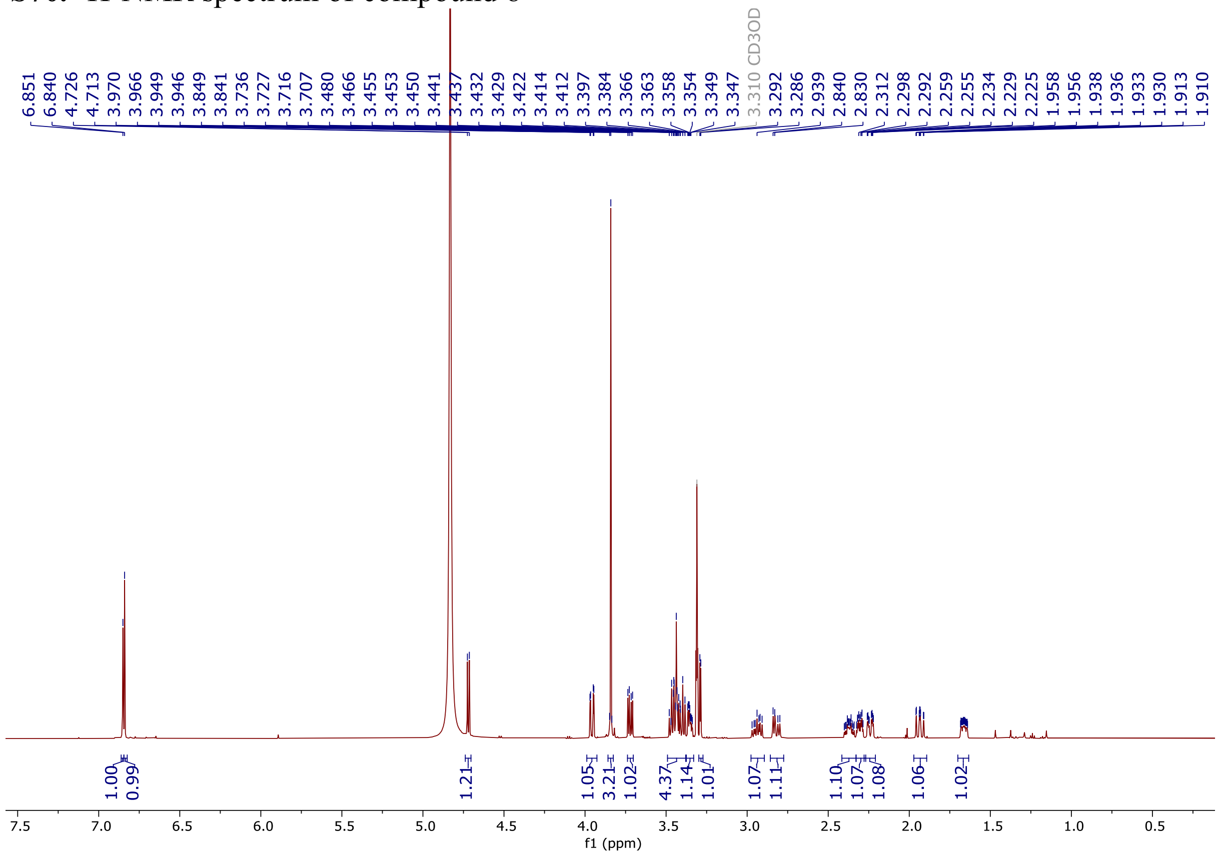

Figure S71. ^{13}C-NMR spectrum of compound 8

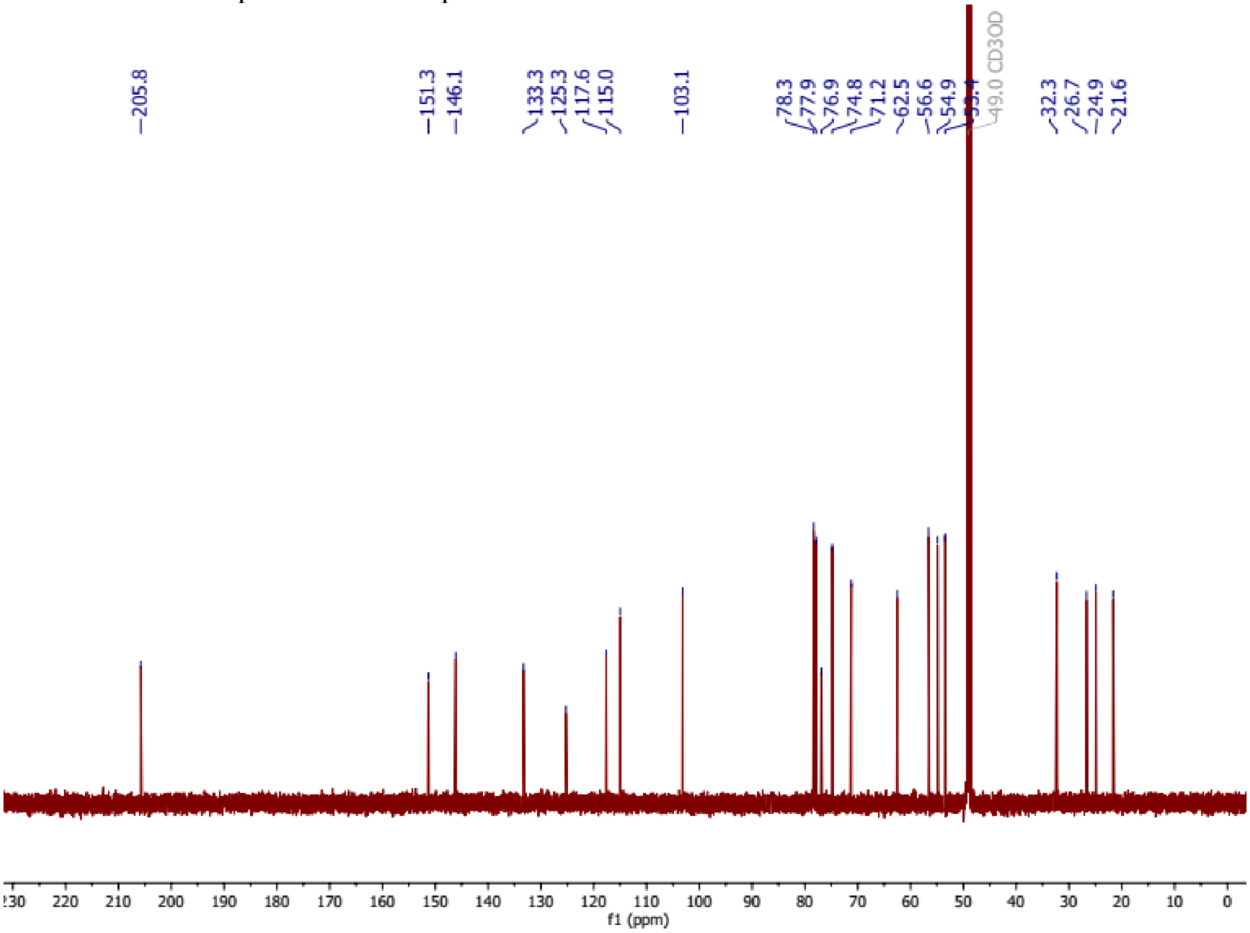

Figure S72. DEPT NMR spectrum of compound 8

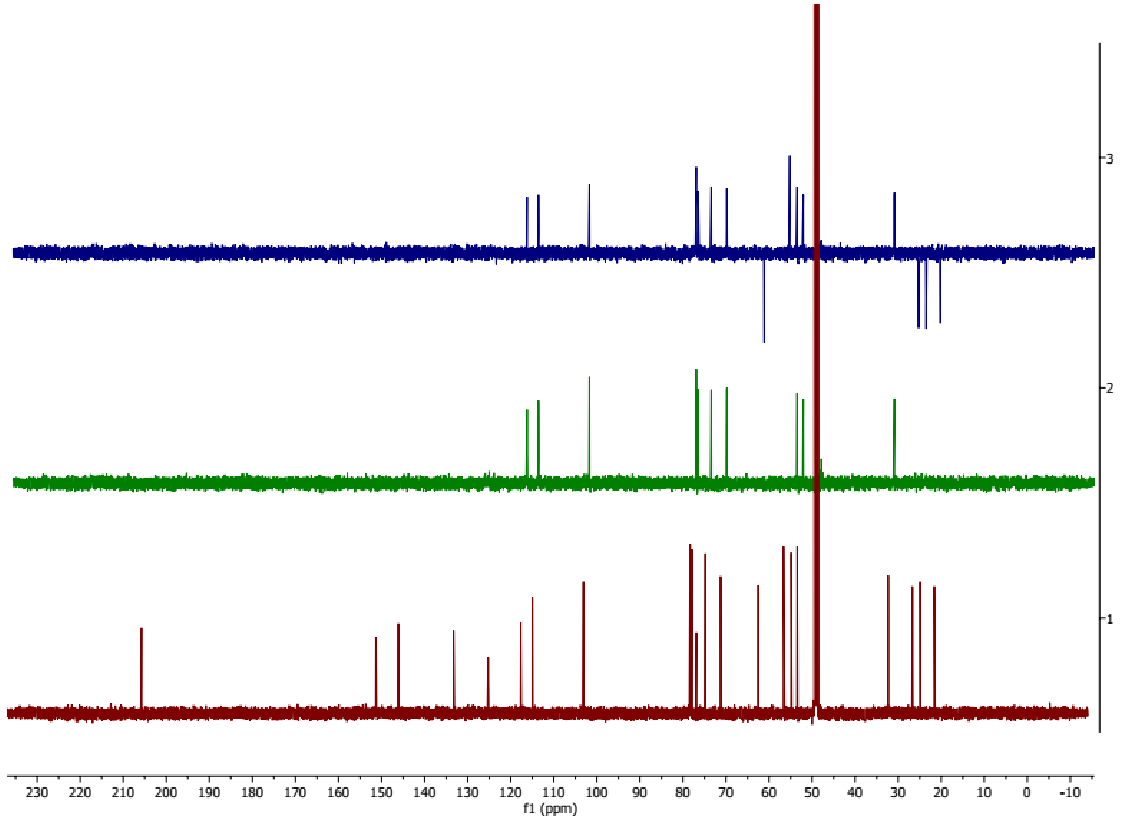

Figure S73. HSQC NMR spectrum of compound 8

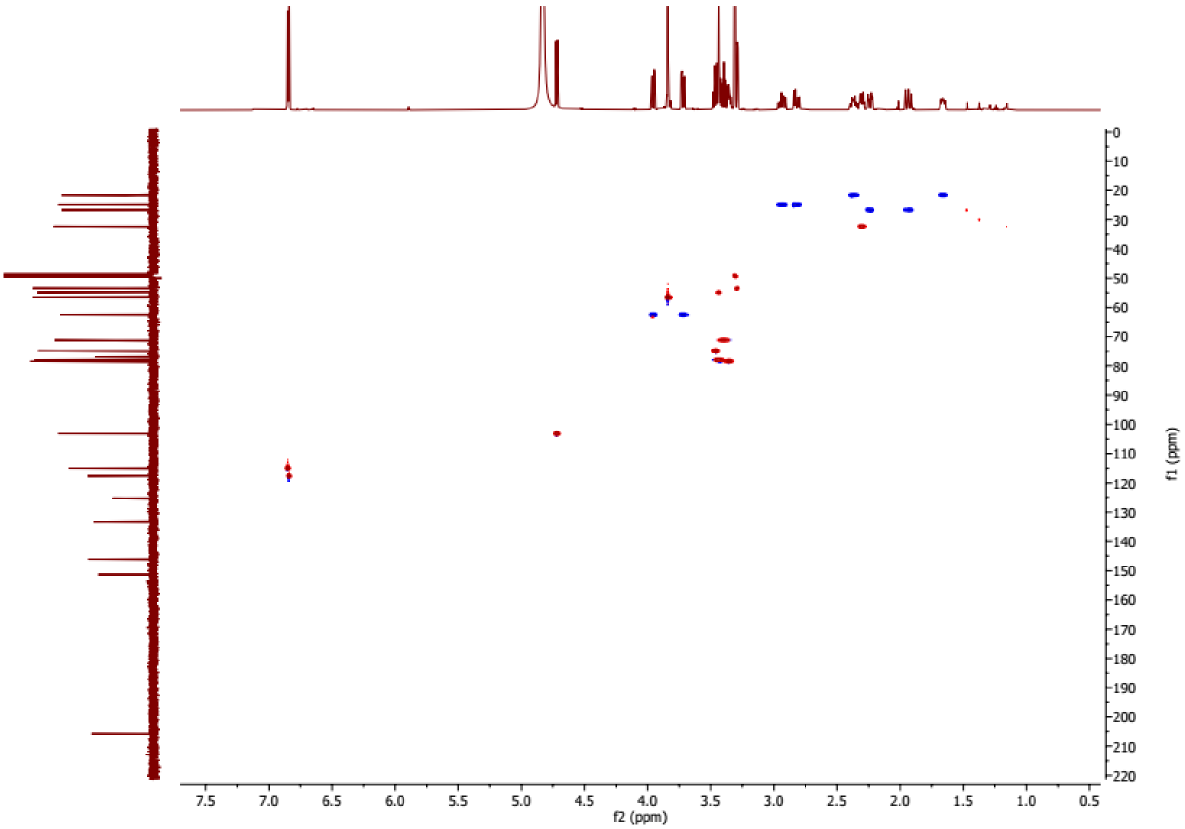

Figure S74. HMBC NMR spectrum of compound 8

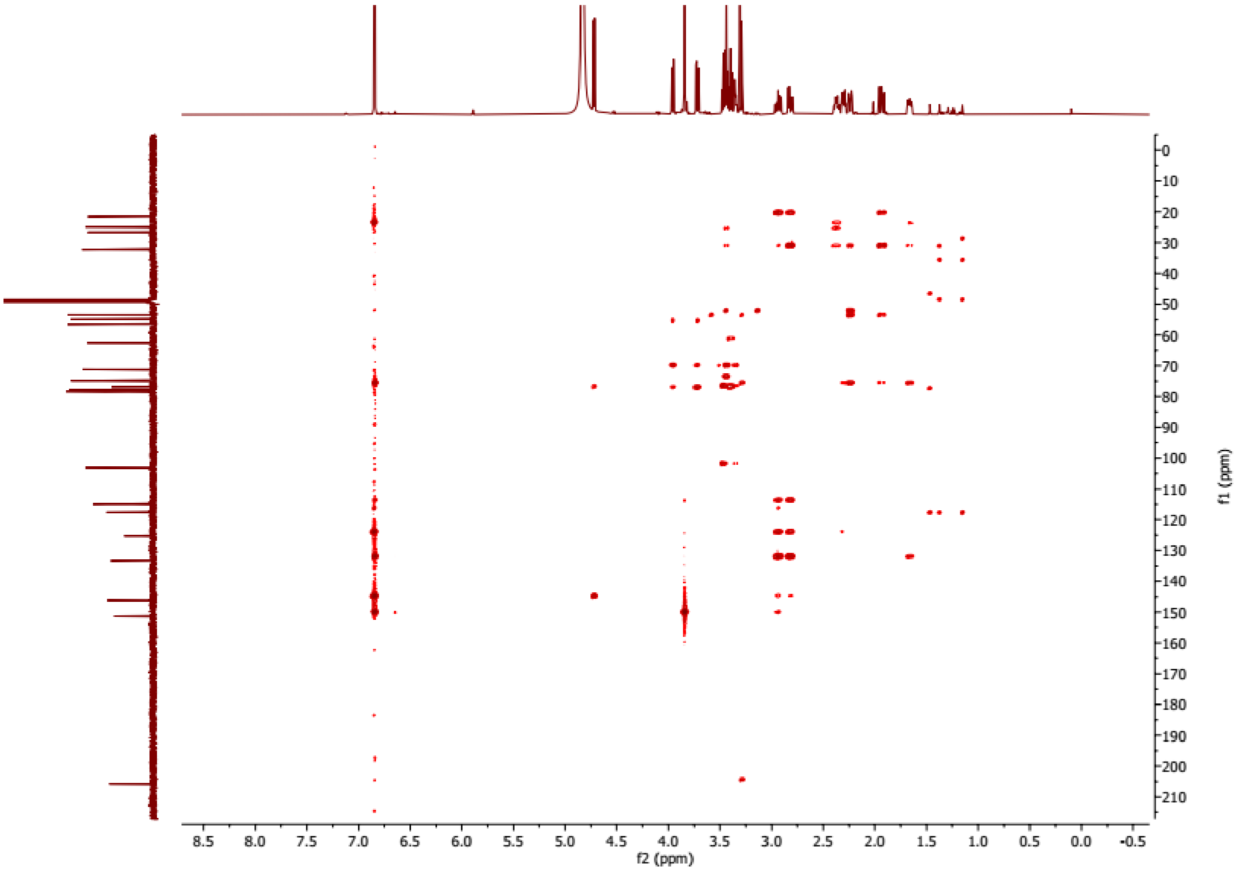

Figure S75. COSY NMR spectrum of compound 8

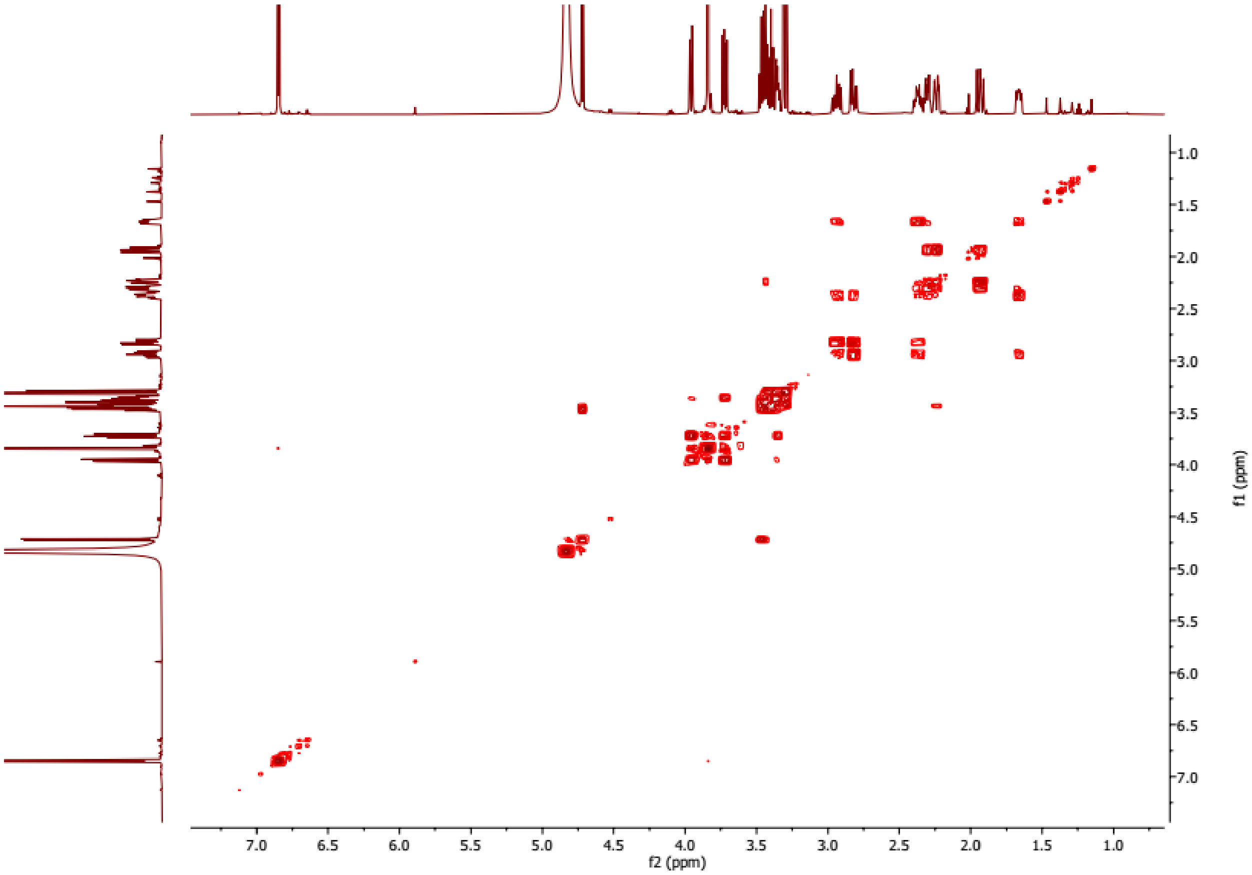

Figure S76. NOESY NMR spectrum of compound 8

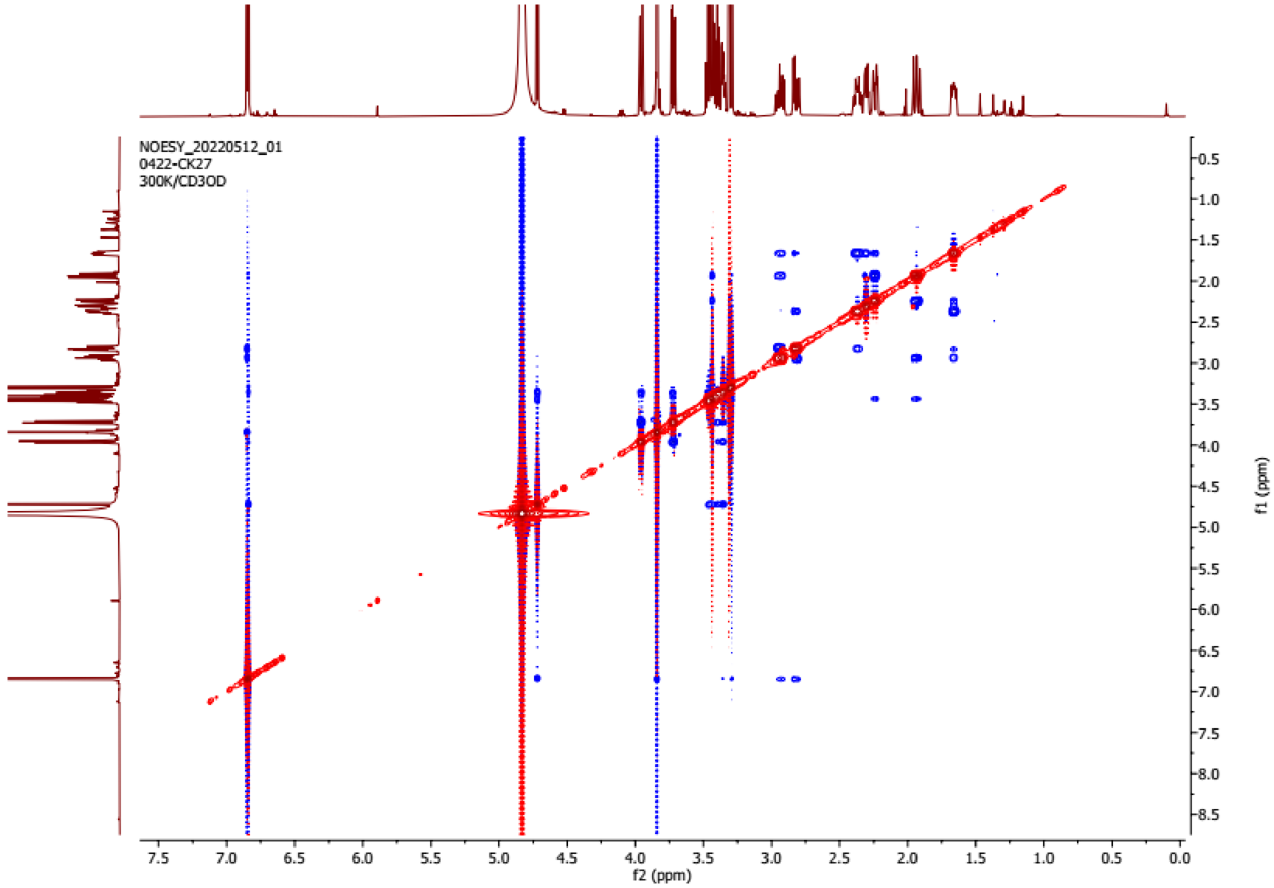

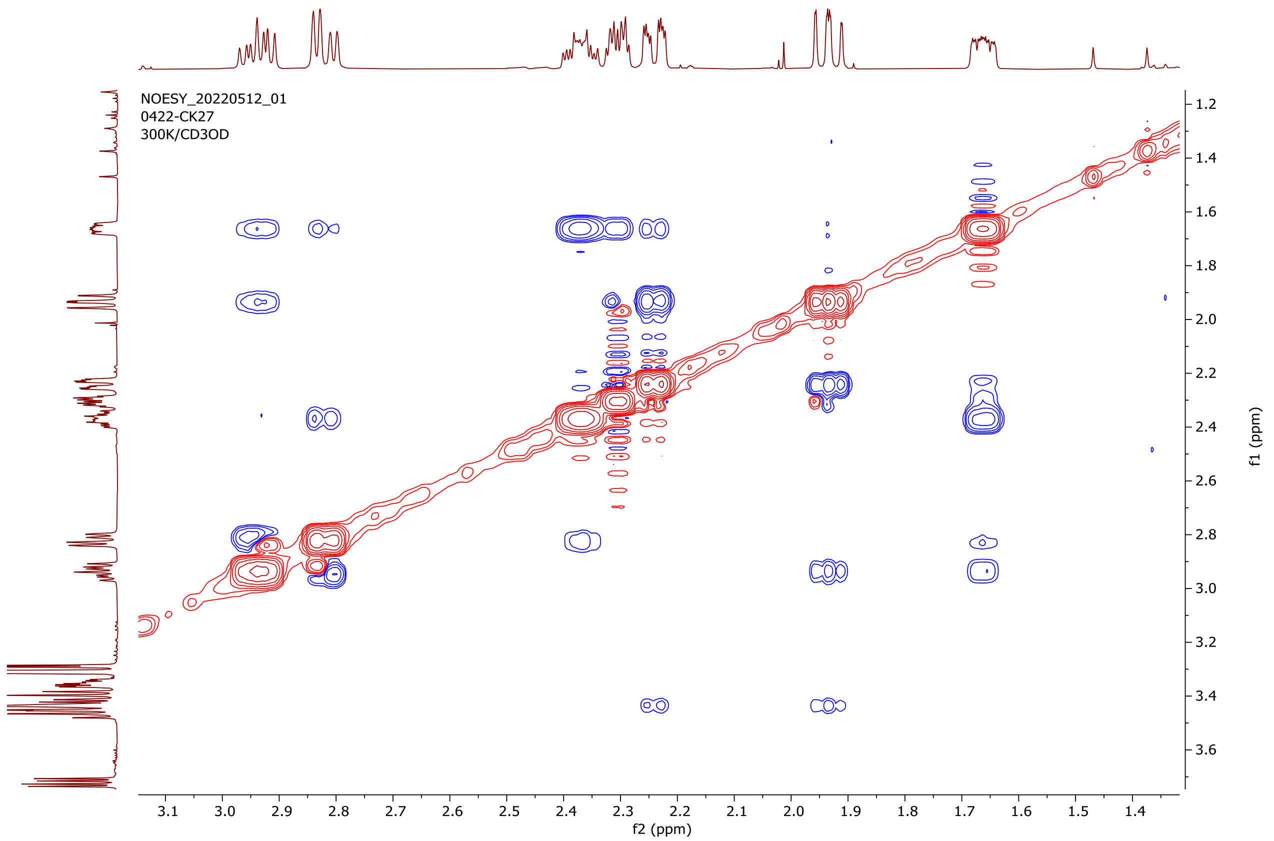

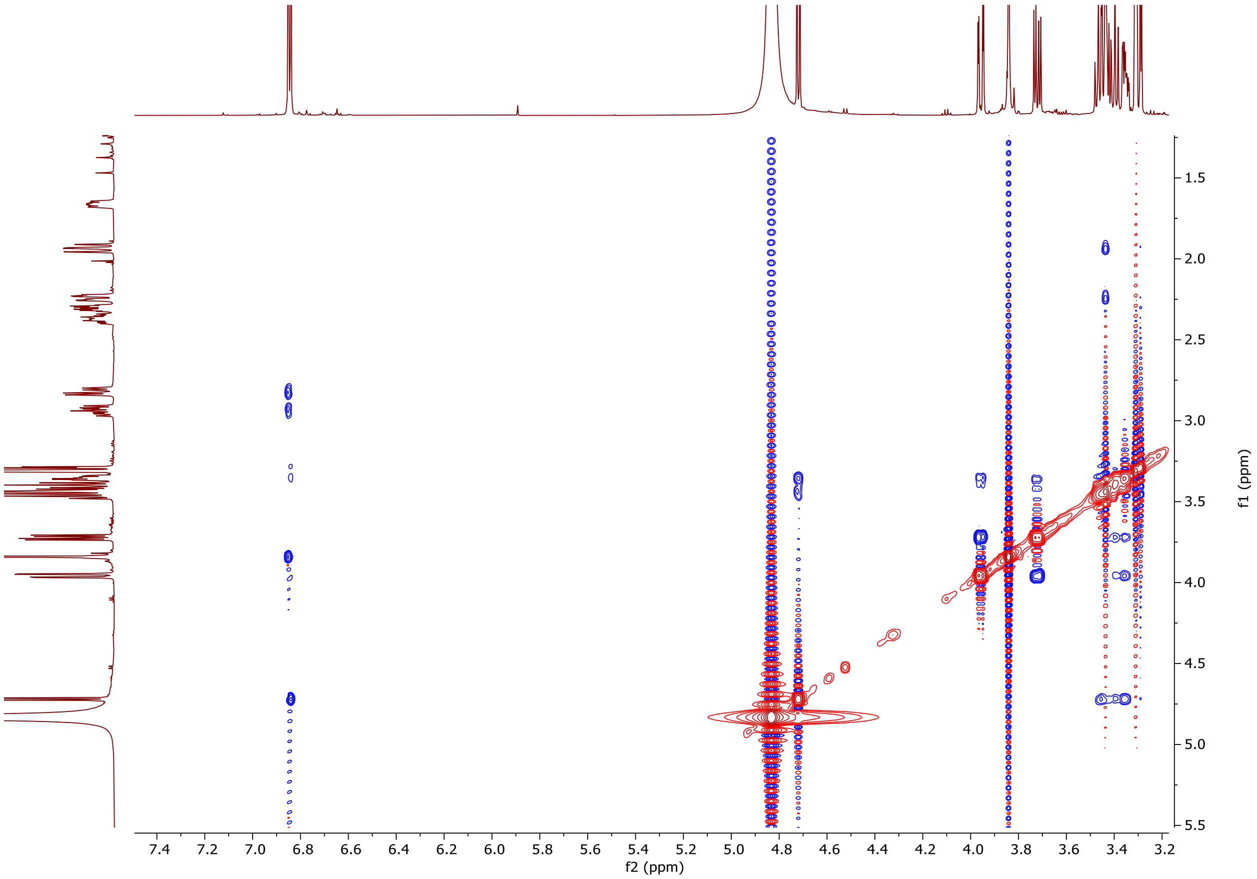

Figure S77. HR-ESI-MS spectrum of compound 8

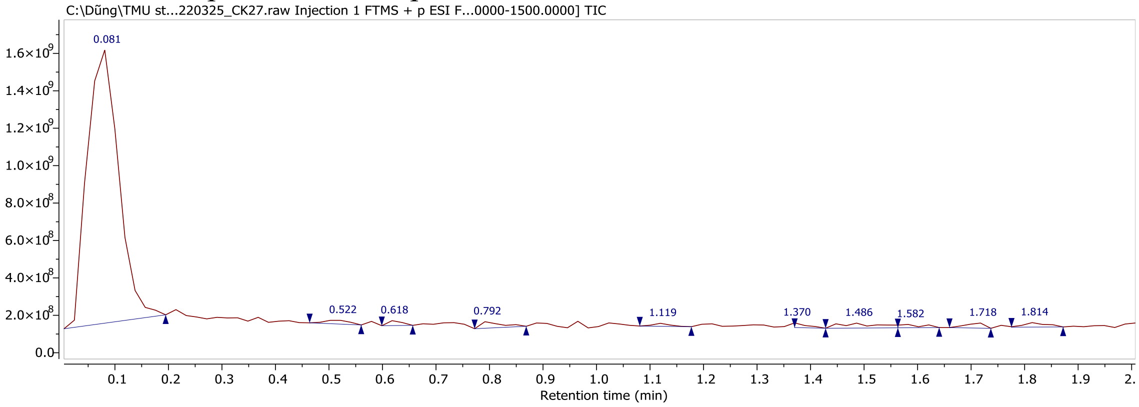

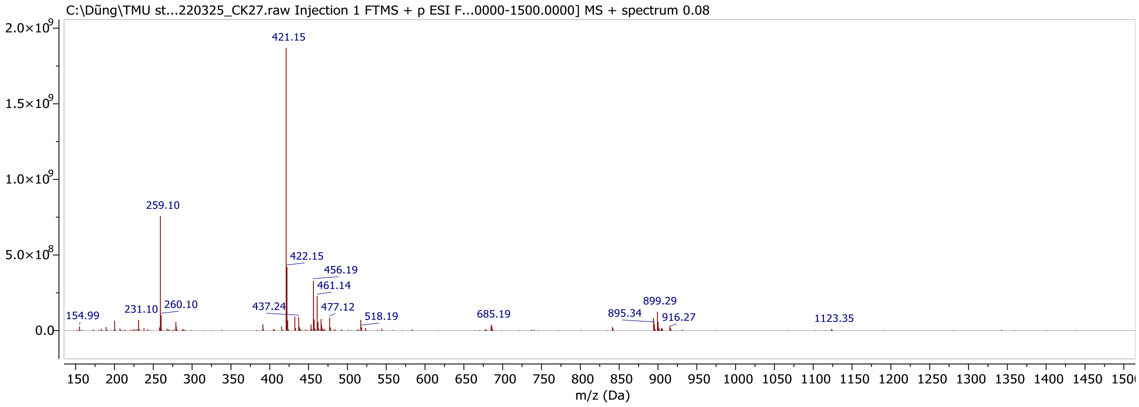

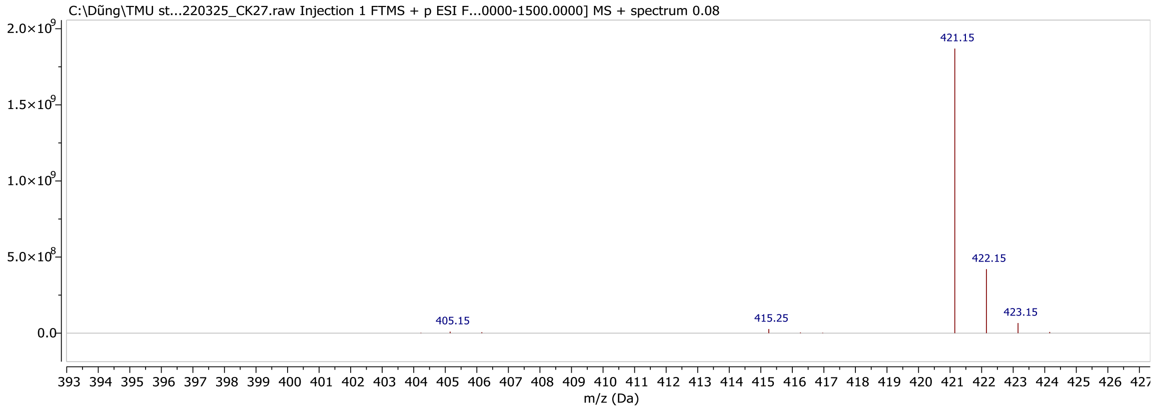

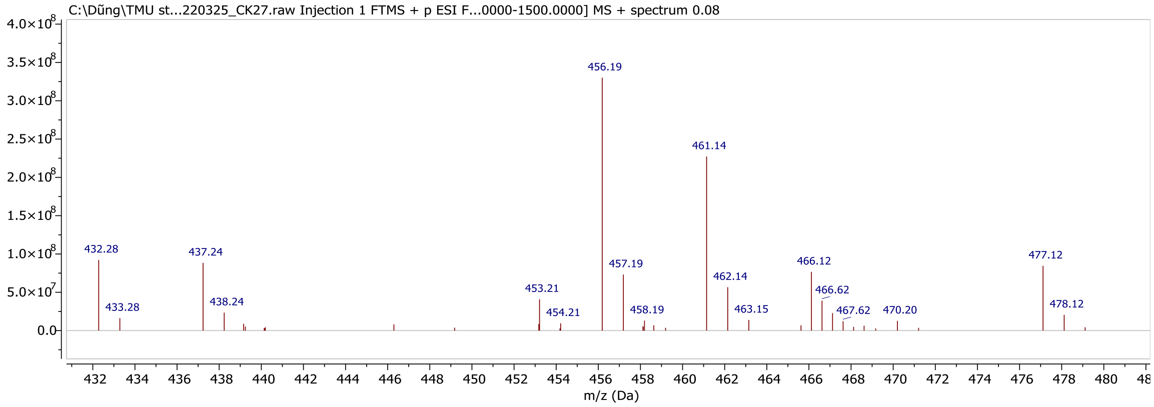

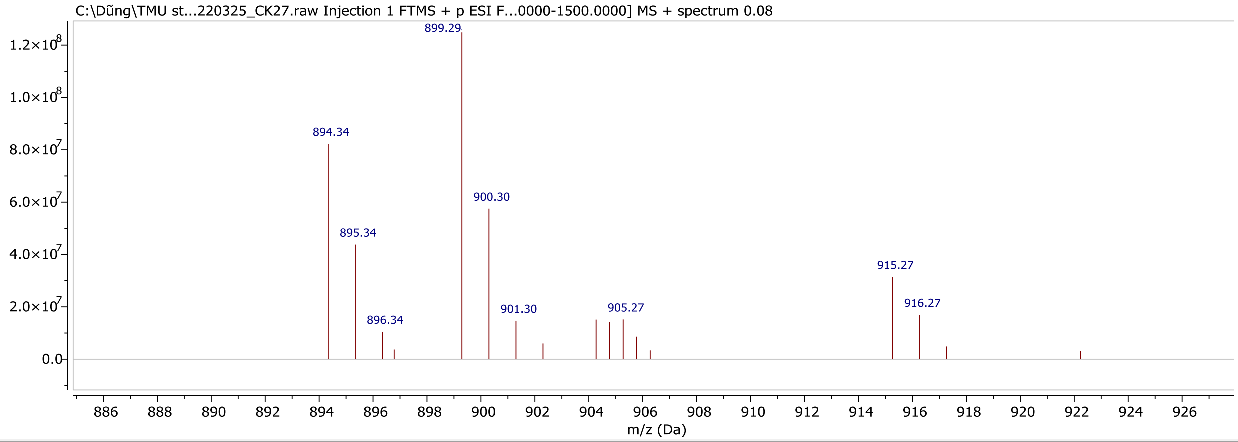

	Formula	Calculated Mass	Target Mass	Double Bond Equivalence	Absolute Error (ppm)	Error (mDa)	Error (ppm)	Fitness
1	C21 H25 O9	421.14931	421.14903	10.0	0.67	-0.28	-0.67	1.000

	Formula	Calculated Mass	Target Mass	Double Bond Equivalence	Absolute Error (ppm)	Error (mDa)	Error (ppm)	Fitness
1	C21 H26 O10 Na	461.14182	461.14154	9.0	0.61	-0.28	-0.61	0.999

	Formula	Calculated Mass	Target Mass	Double Bond Equivalence	Absolute Error (ppm)	Error (mDa)	Error (ppm)	Fitness
1	C42 H52 O20 Na	899.29442	899.29362	17.0	0.88	-0.79	-0.88	0.998

	Formula	Calculated Mass	Target Mass	Double Bond Equivalence	Absolute Error (ppm)	Error (mDa)	Error (ppm)	Fitness
1	C42 H52 O20 K	915.26835	915.26745	17.0	0.98	-0.90	-0.98	0.978

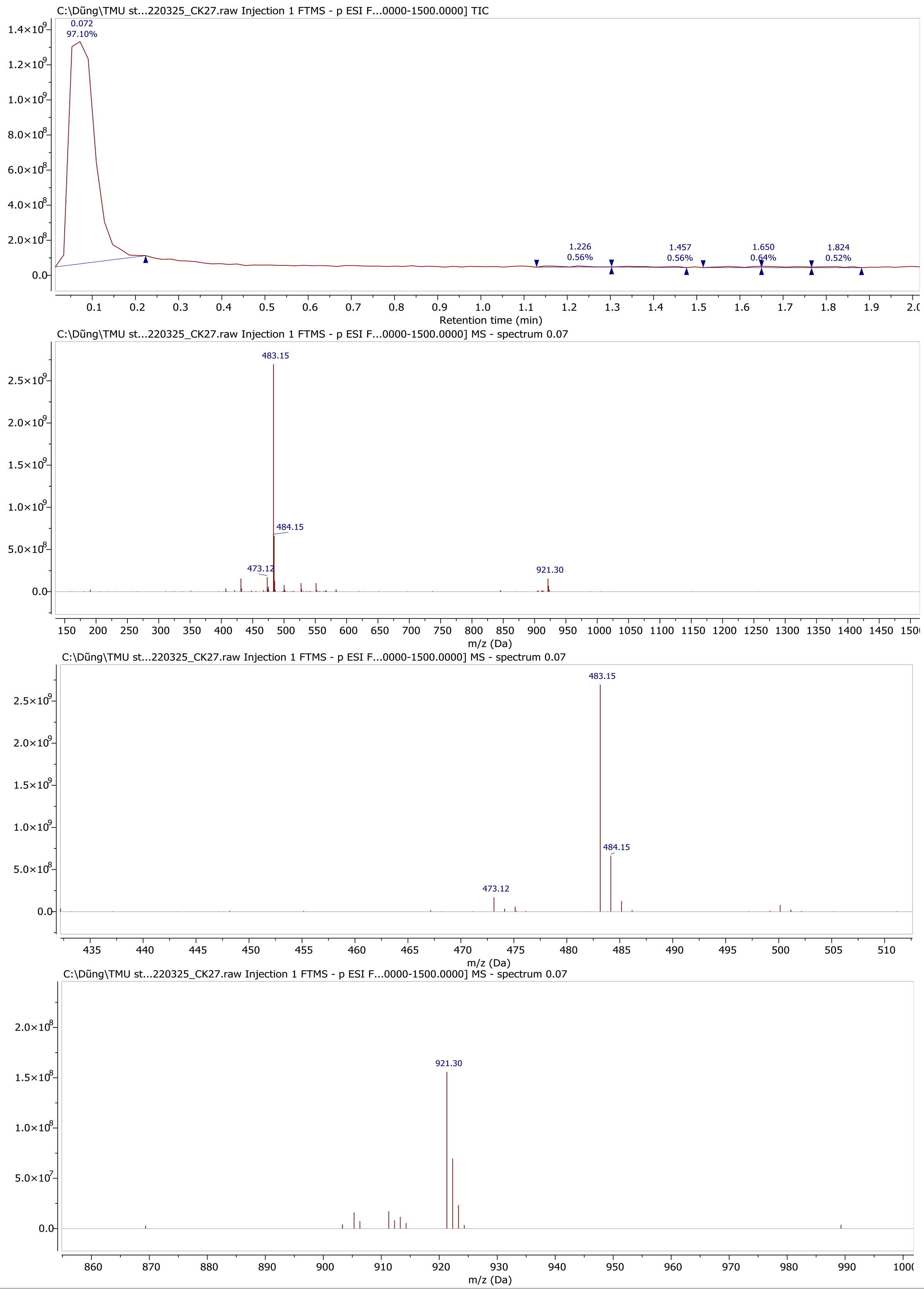

	Formula	Calculated Mass	Target Mass	Double Bond Equivalence	Absolute Error (ppm)	Error (mDa)	Error (ppm)	Fitness
1	C22 H27 O12	483.14970	483.14933	10.0	0.77	-0.37	-0.77	1.000

	Formula	Calculated Mass	Target Mass	Double Bond Equivalence	Absolute Error (ppm)	Error (mDa)	Error (ppm)	Fitness
1	C43 H53 O22	921.30230	921.30106	18.0	1.35	-1.24	-1.35	0.998

Figure S78. UV spectrum of compound 8

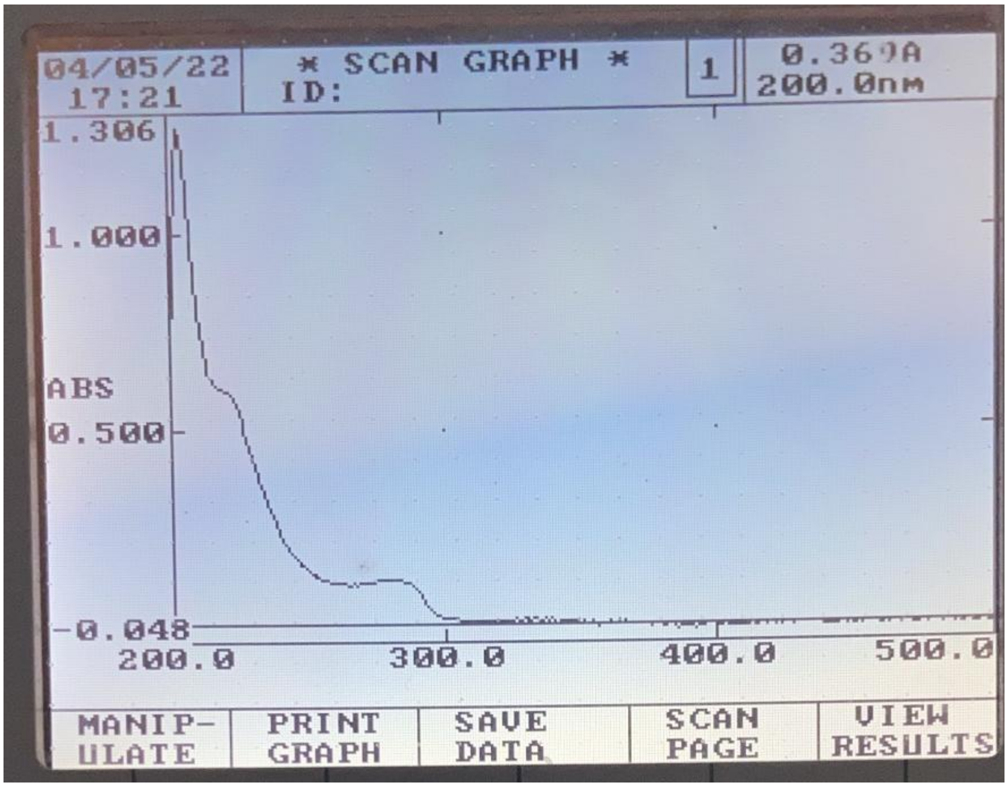

Figure S79. IR spectrum of compound 8

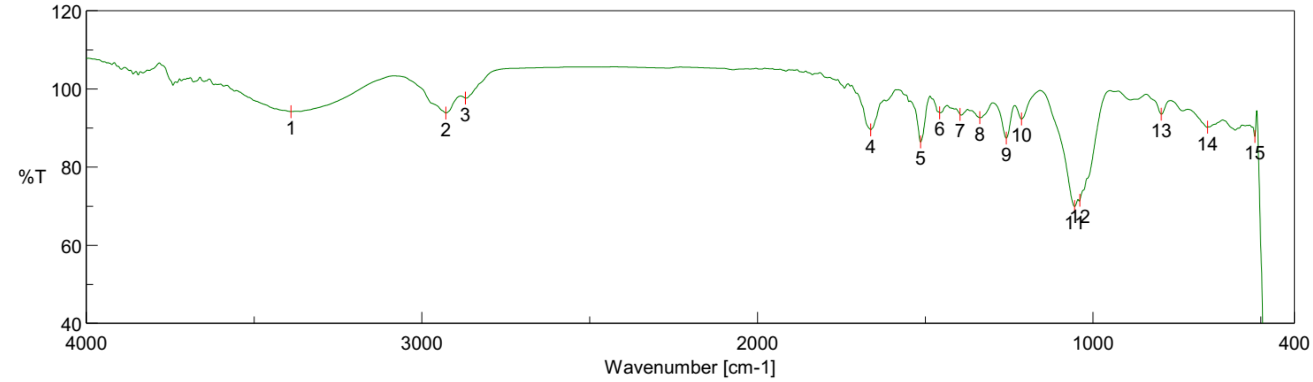

[Result of Peak Picking]

No.	Position	Intensity	No.	Position	Intensity	No.	Position	Intensity
1	3390.24	94.175	2	2928.38	93.8125	3	2870.52	97.6464
4	1662.34	89.5262	5	1513.85	86.4017	6	1456.96	93.8745
7	1396.21	93.4287	8	1337.39	92.6207	9	1258.32	87.3935
10	1213.01	92.3006	11	1054.87	69.8864	12	1039.44	71.4792
13	796.457	93.5176	14	658.571	90.2083	15	517.793	87.7855

Figure S80. ^{1}H-NMR spectrum of compound 9

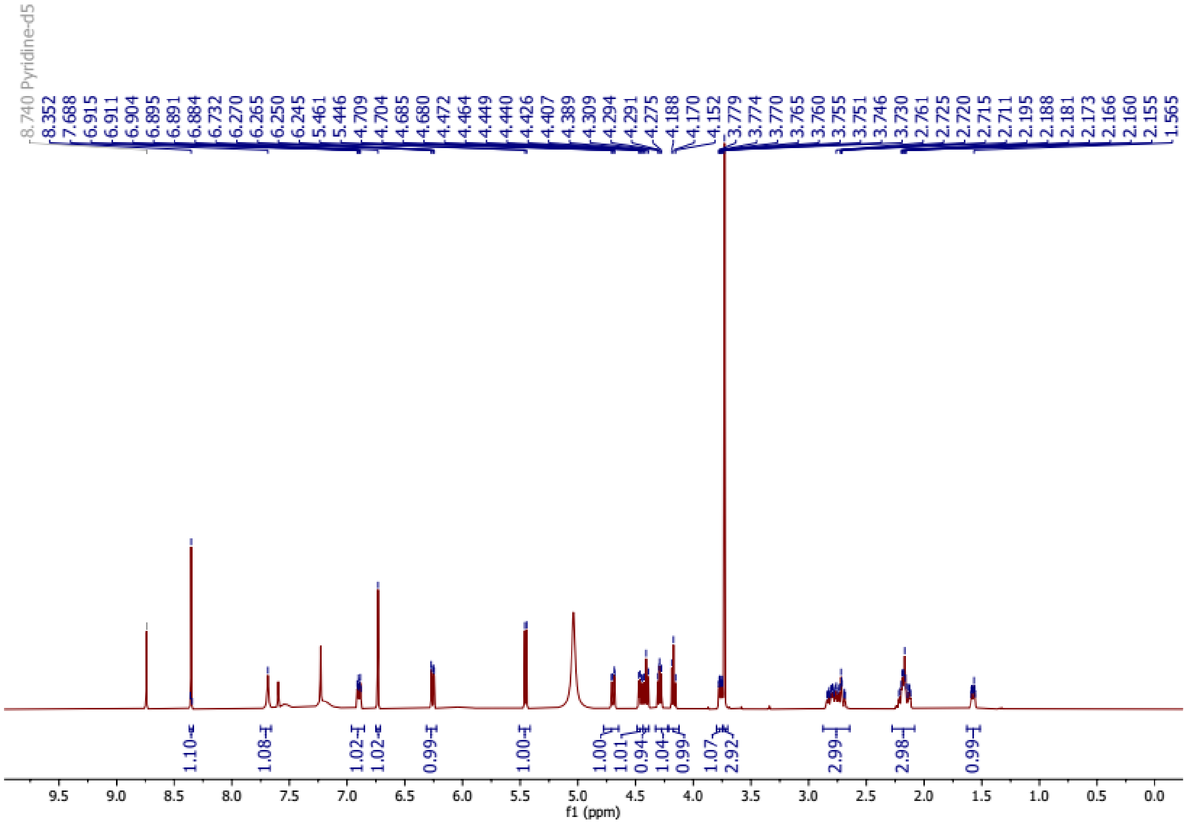

Figure S81. ^{13}C-NMR spectrum of compound 9

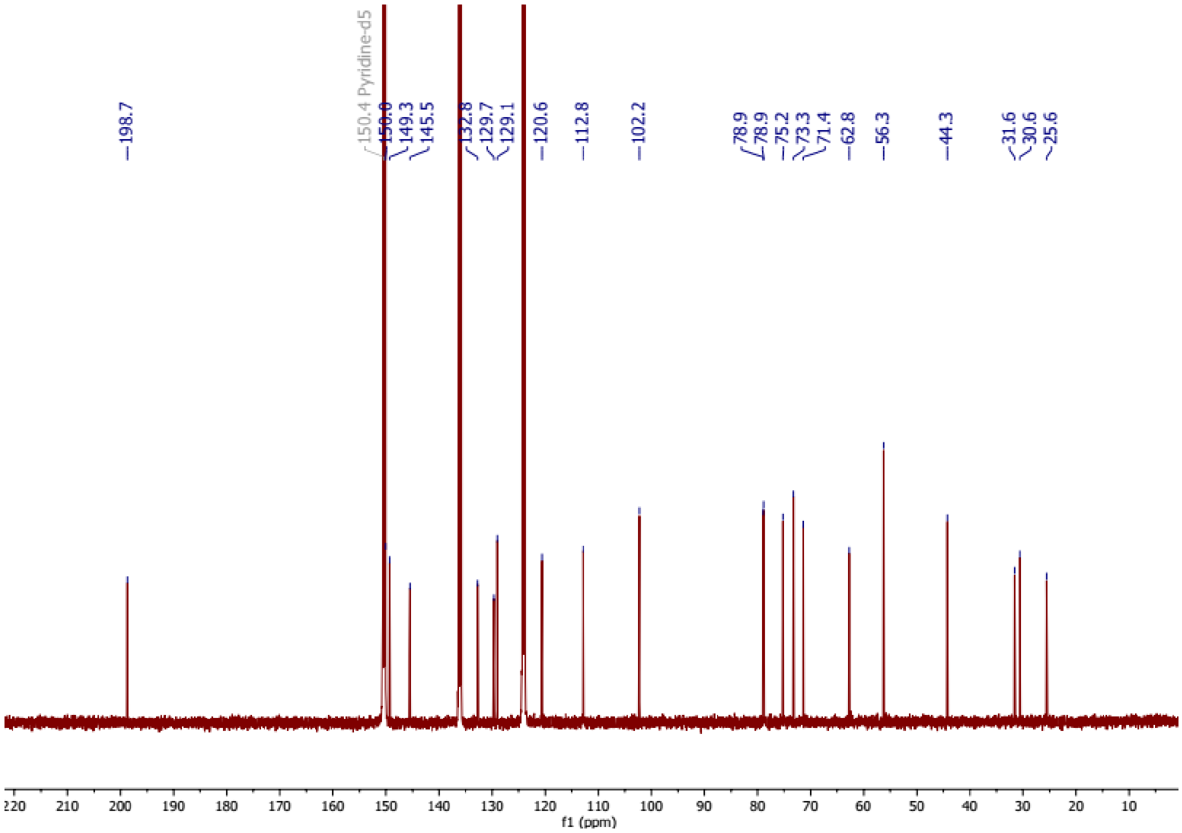

Figure S82. DEPT NMR spectrum of compound 9

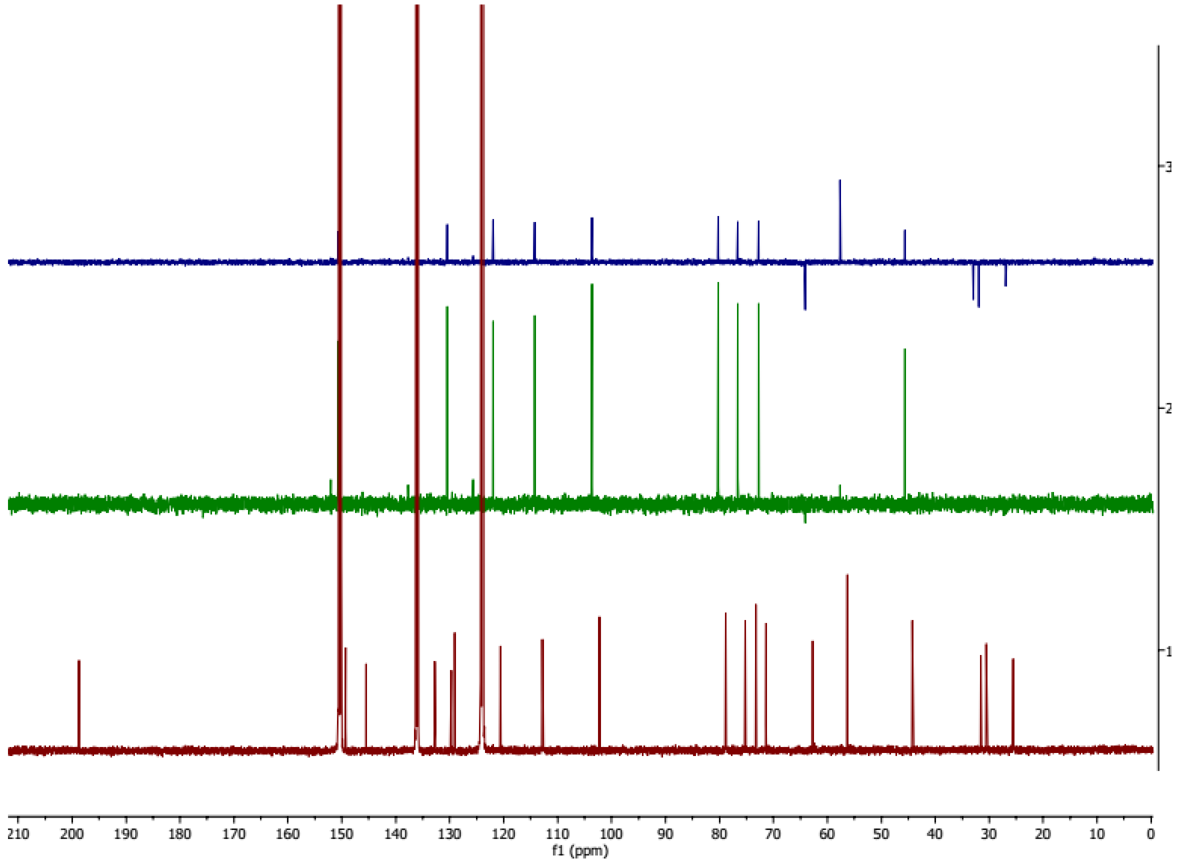

Figure S83. HSQC NMR spectrum of compound 9

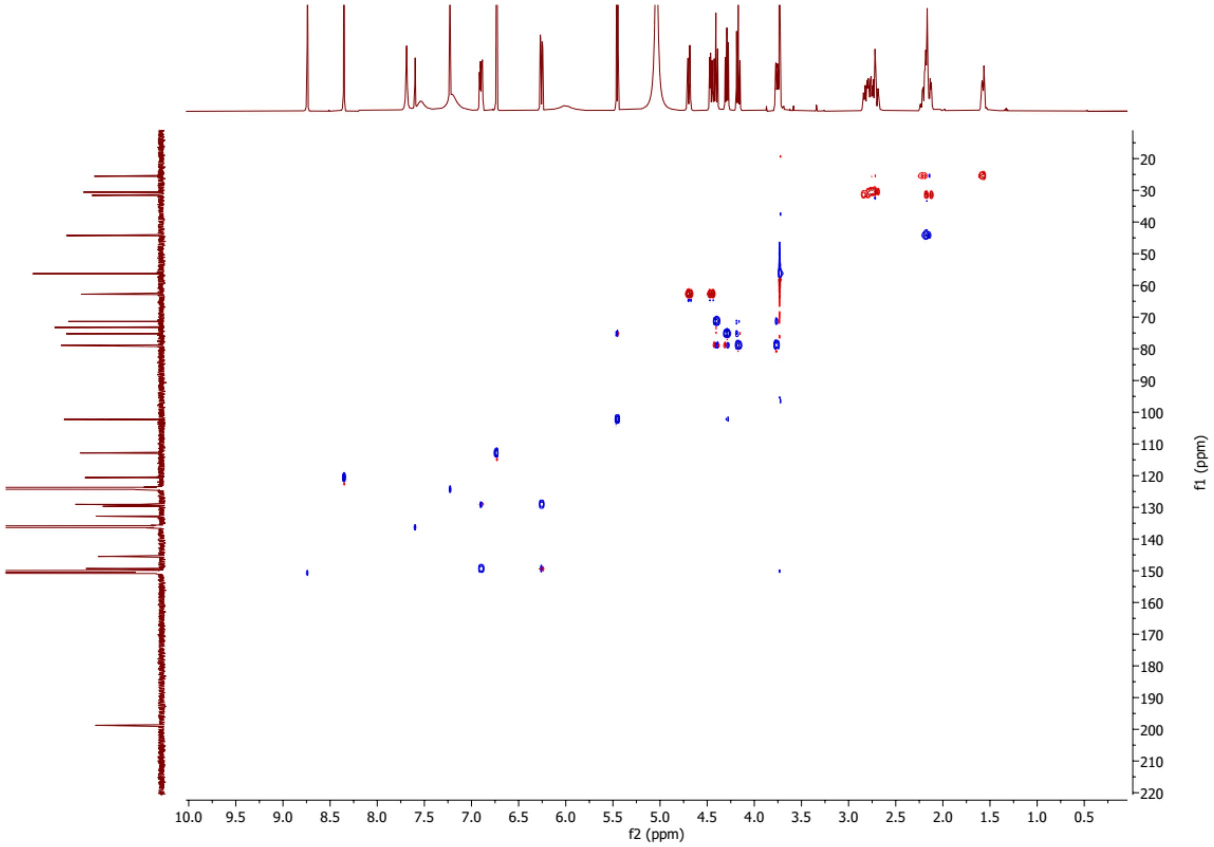

Figure S84. HMBC NMR spectrum of compound 9

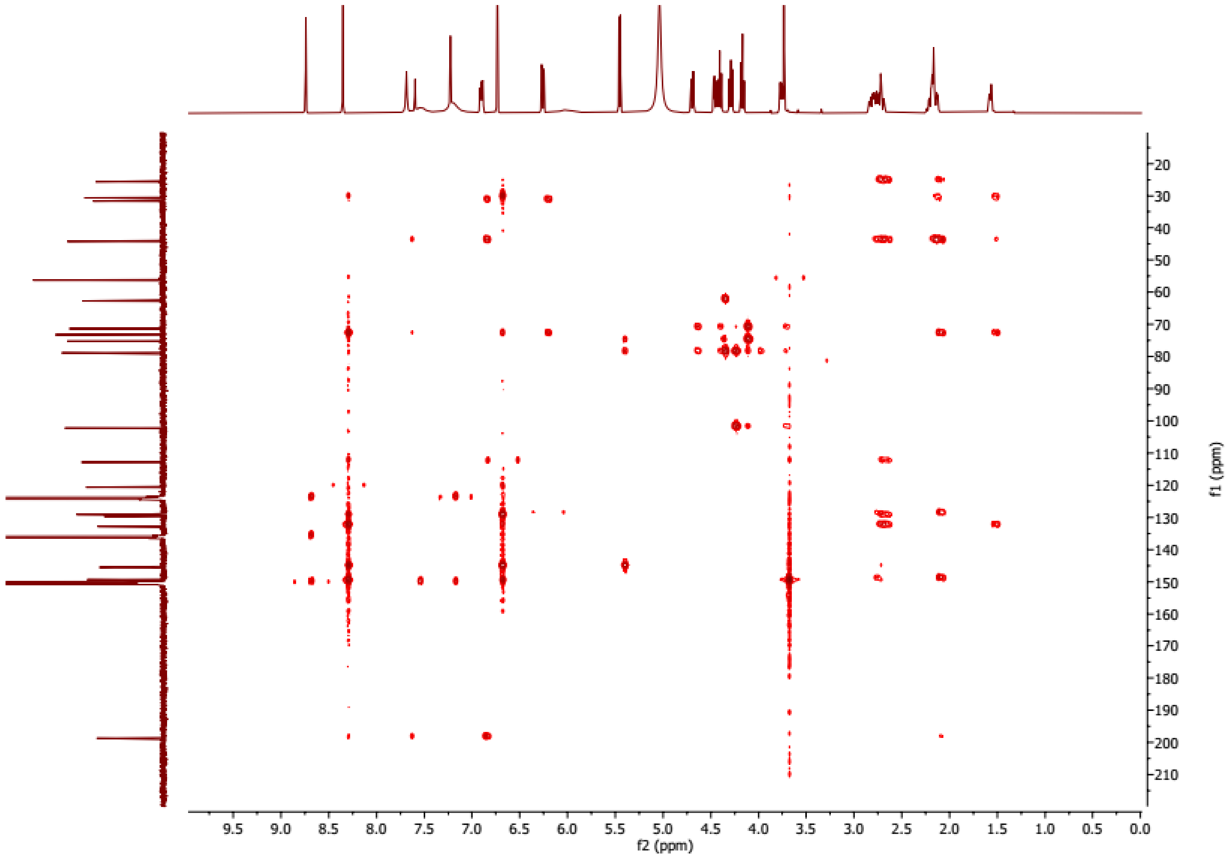

Figure S85. COSY NMR spectrum of compound 9

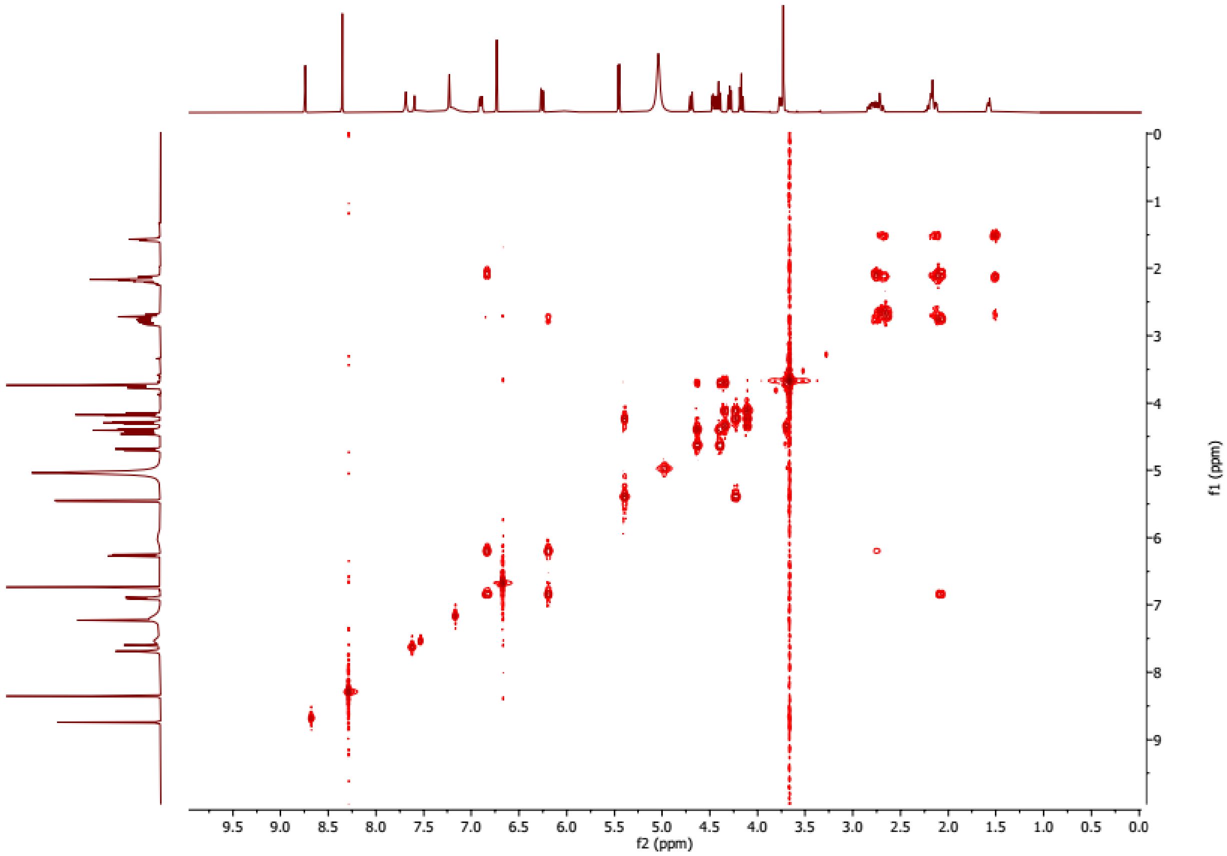

Figure S86. NOESY NMR spectrum of compound 9

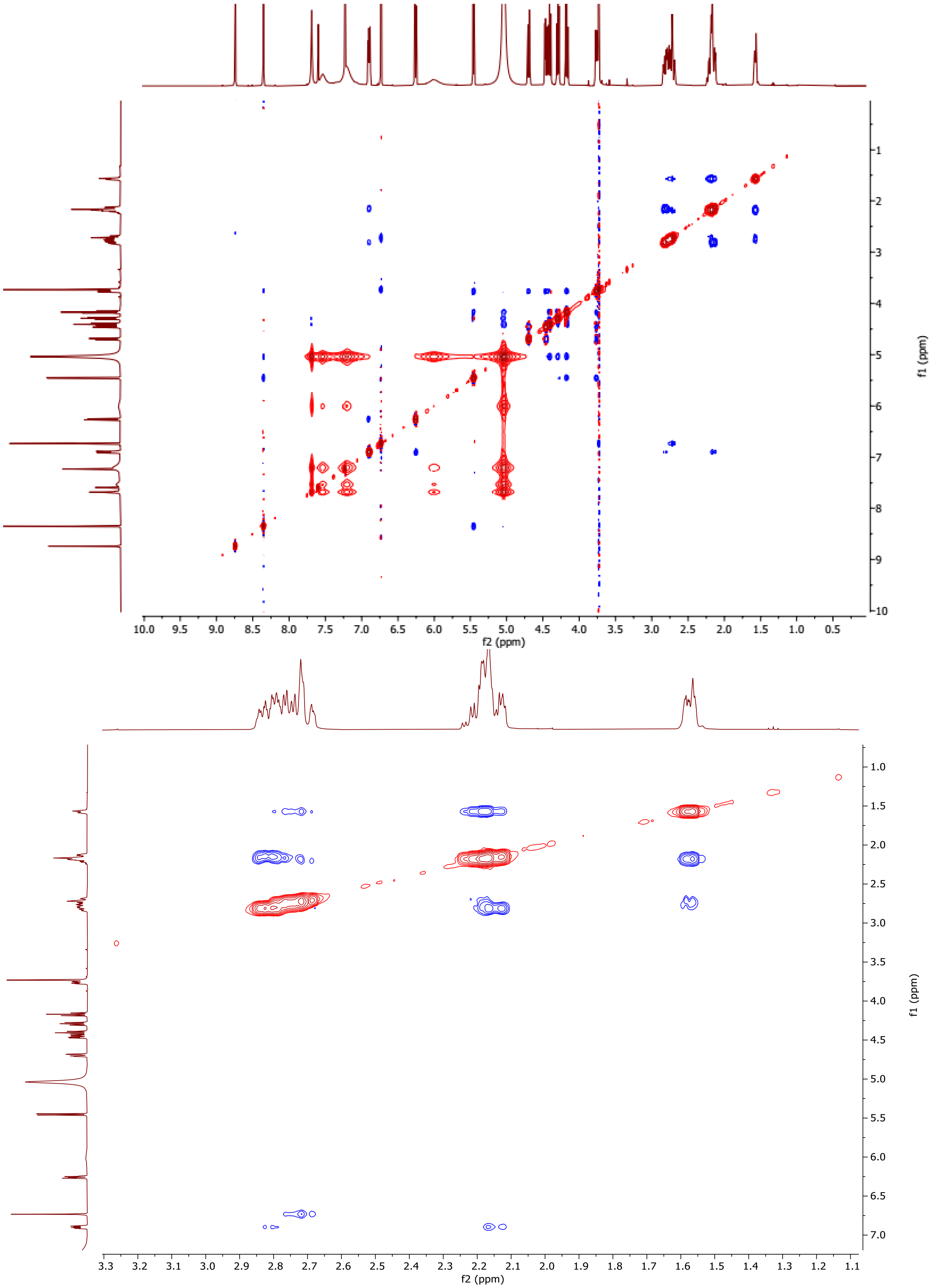

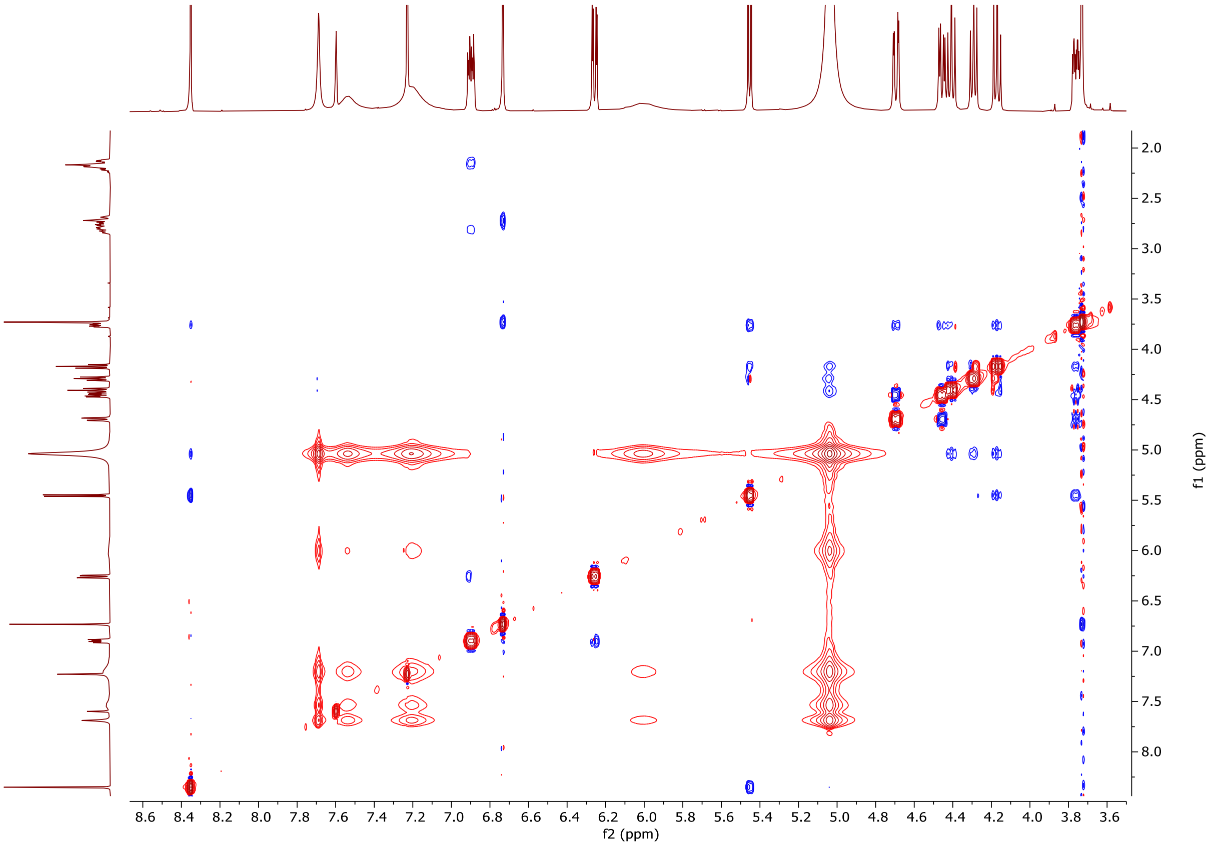

Figure S87. HR-ESI-MS spectrum of compound 9

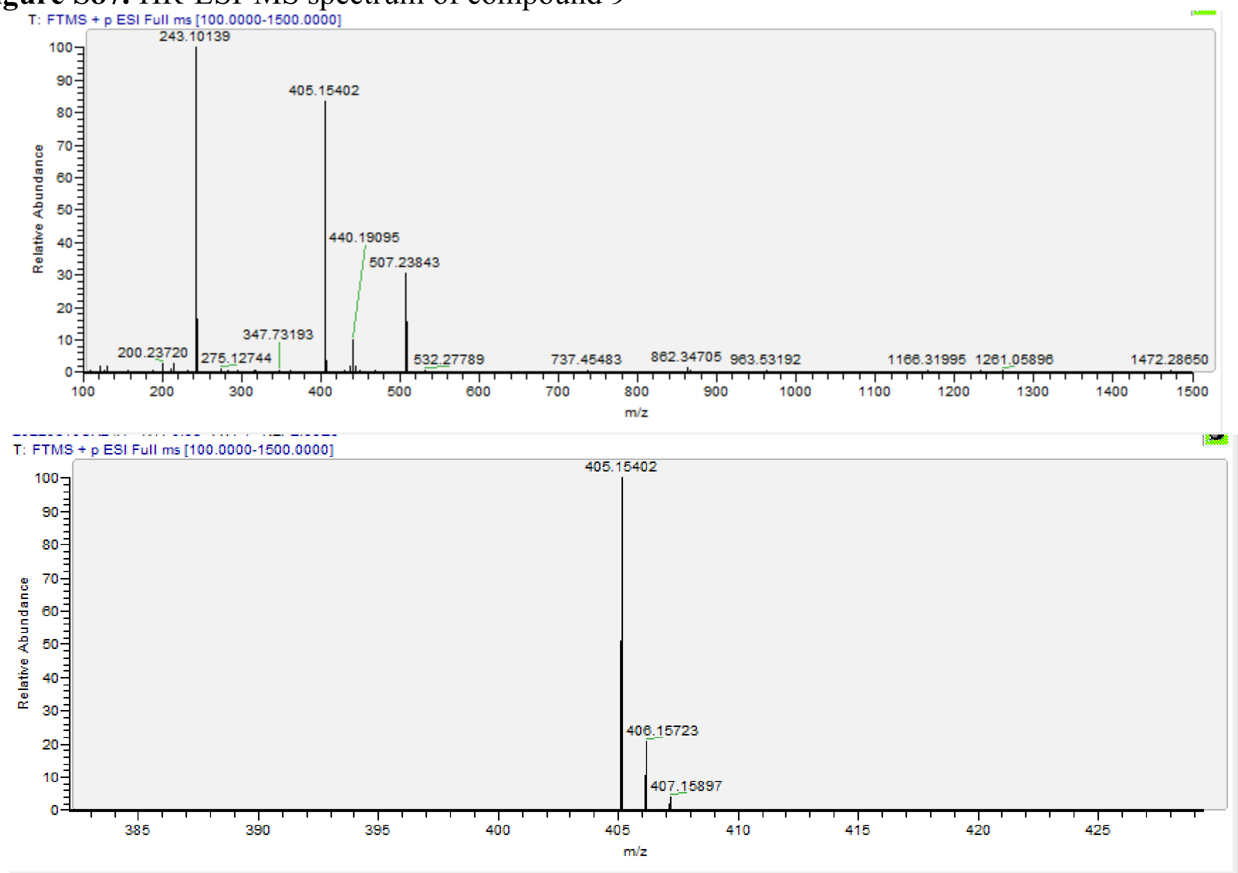

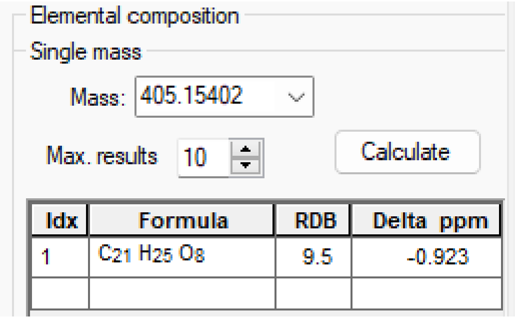

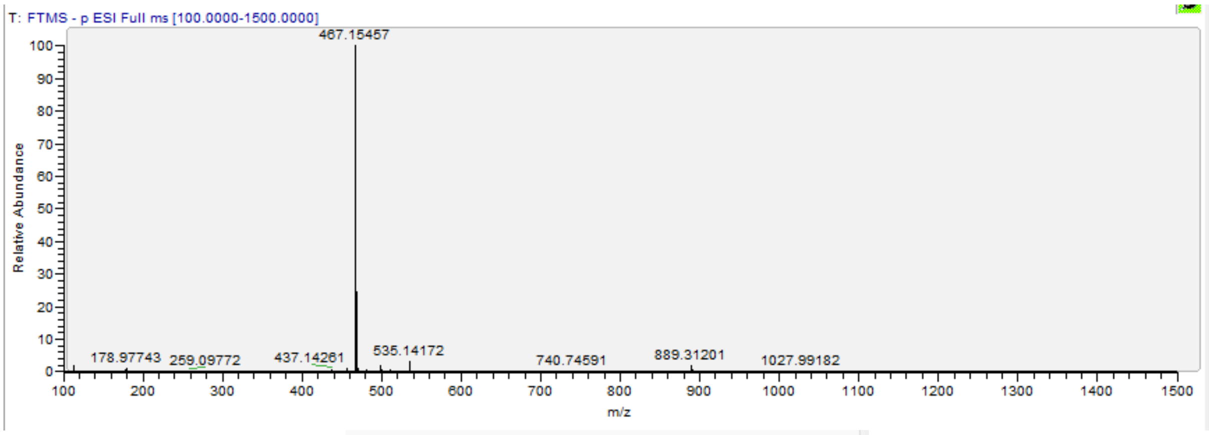

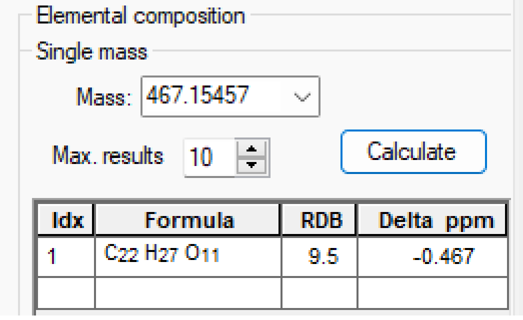

Figure S88. UV spectrum of compound 9

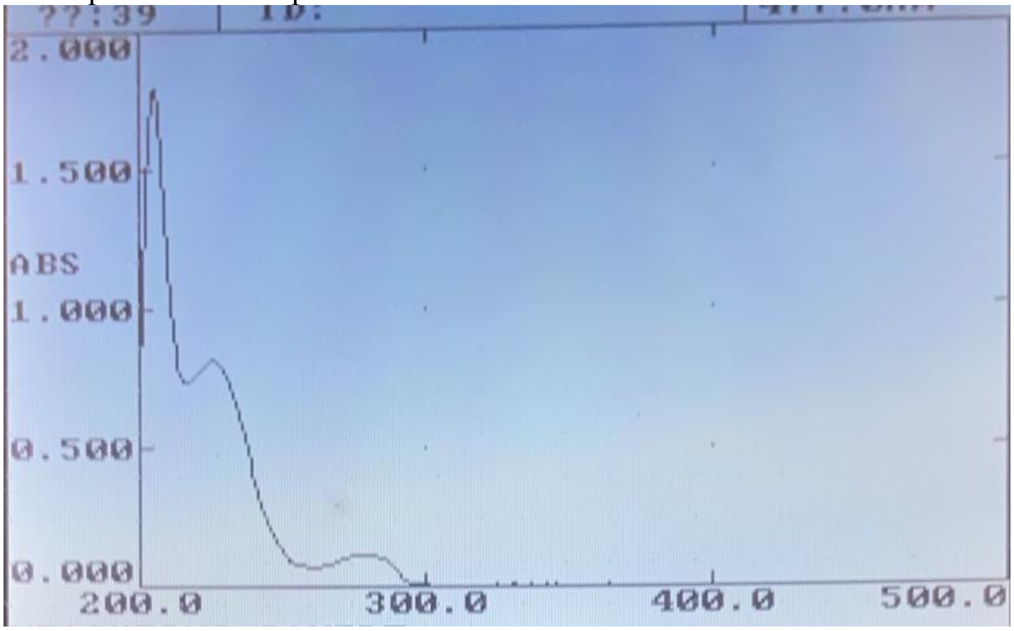

Figure S89. IR spectrum of compound 9

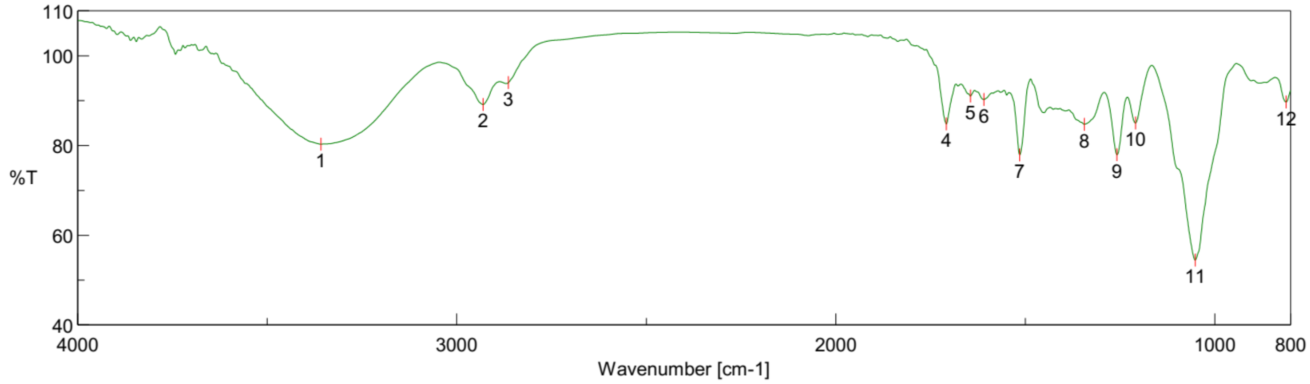

[Result of Peak Picking]

No.	Position	Intensity	No.	Position	Intensity	No.	Position	Intensity
1	3358.43	80.2595	2	2930.31	89.0845	3	2863.77	93.9723
4	1708.62	84.7836	5	1644.98	90.9692	6	1609.31	90.2271
7	1514.81	77.8697	8	1344.14	84.7083	9	1258.32	77.9366
10	1209.15	84.9743	11	1051.98	54.4407	12	811.885	89.6517
13	631.573	81.4473						

Figure S90. ^{1}H-NMR spectrum of compound 10

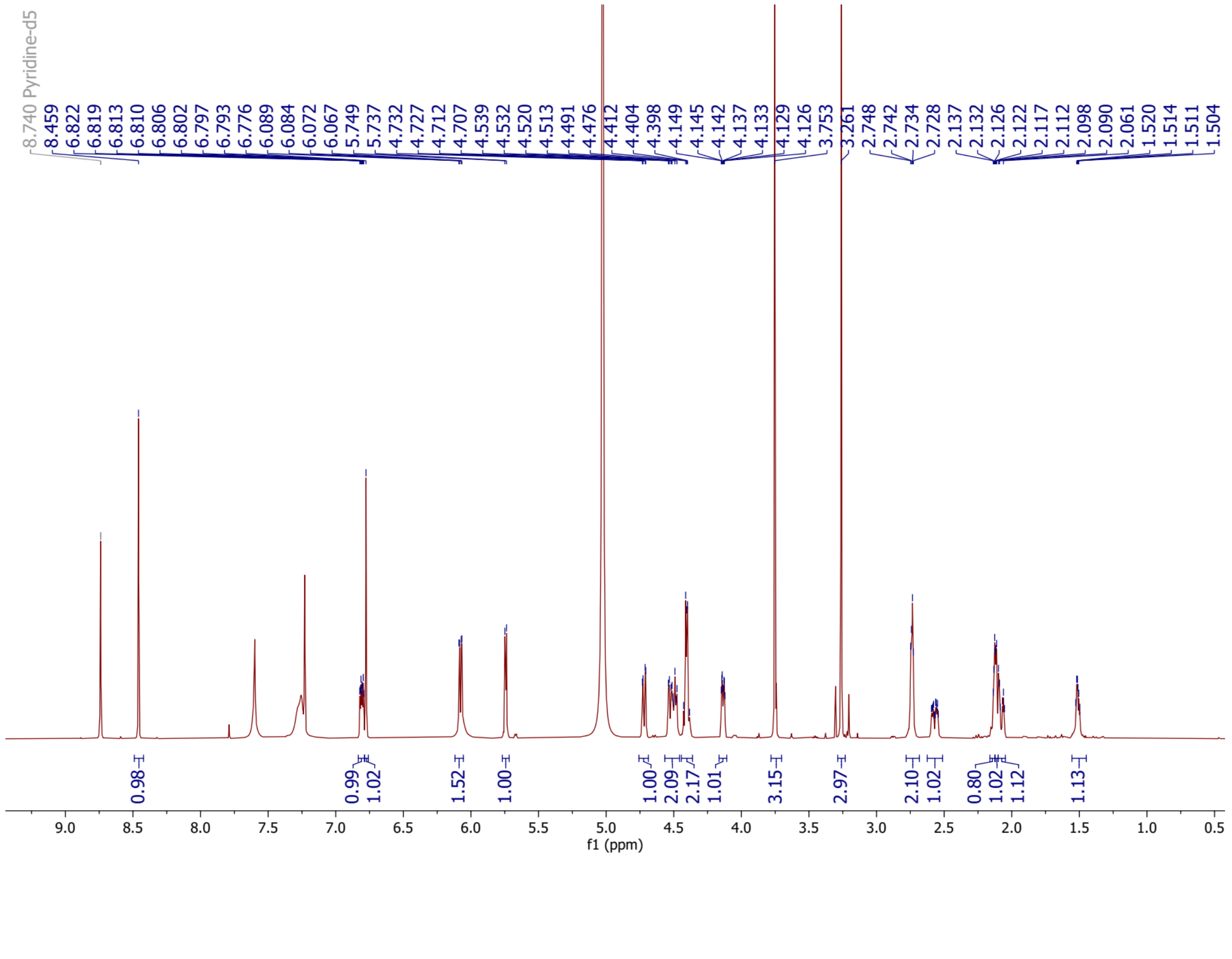

Figure S91. ^{13}C-NMR spectrum of compound 10

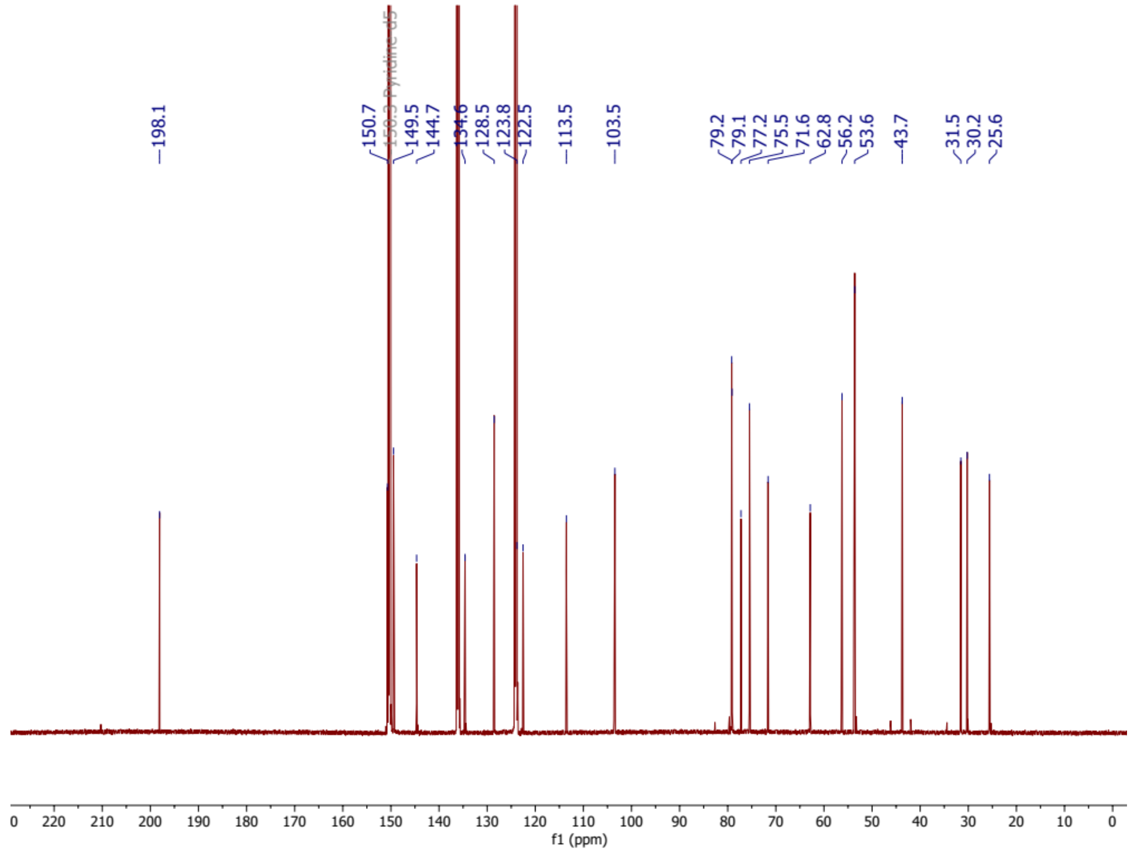

Figure S92. DEPT NMR spectrum of compound 10

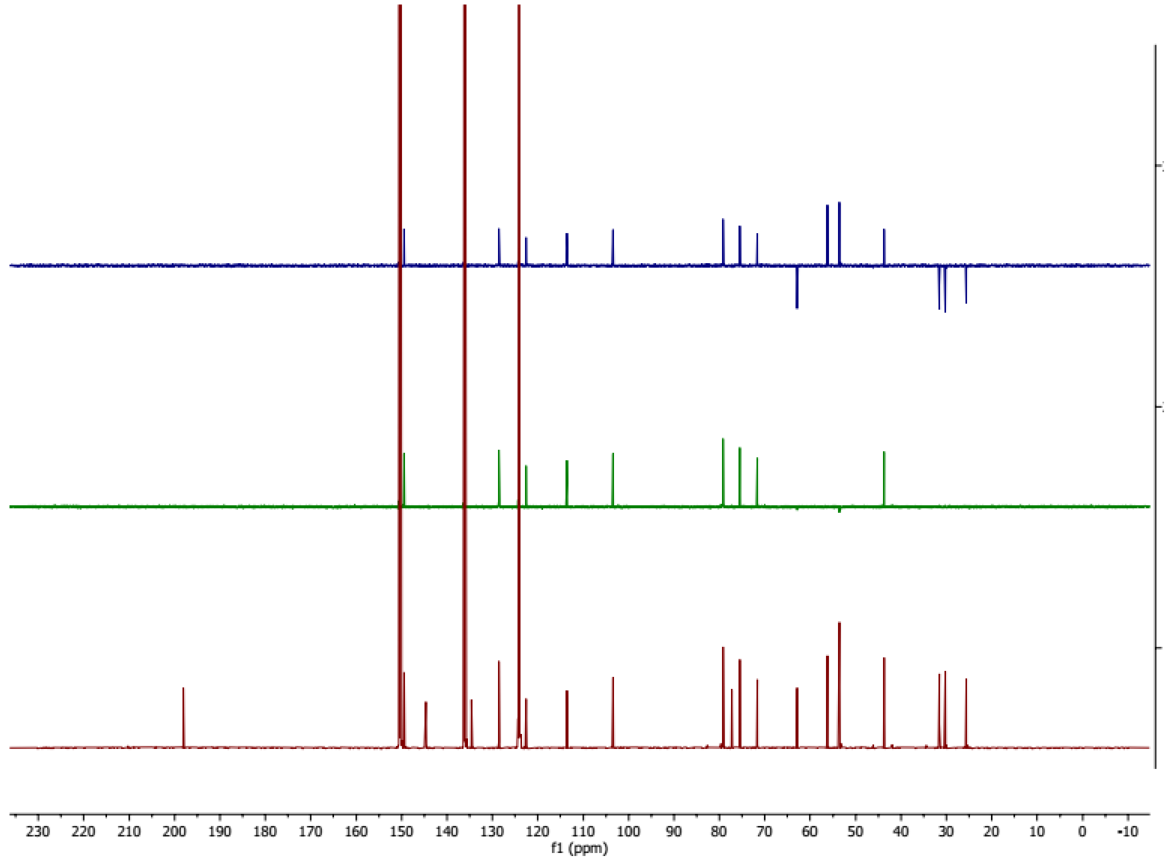

Figure S93. HSQC NMR spectrum of compound 10

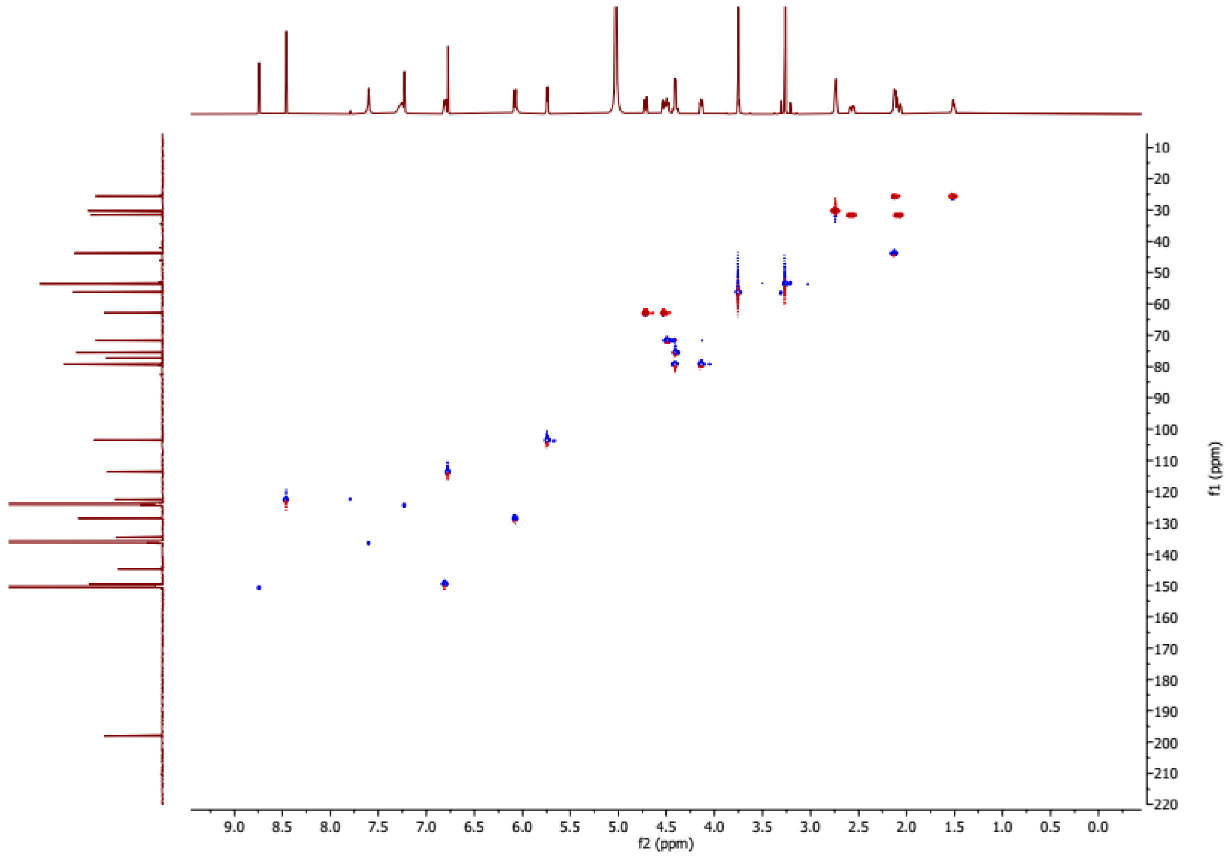

Figure S94. HMBC NMR spectrum of compound 10

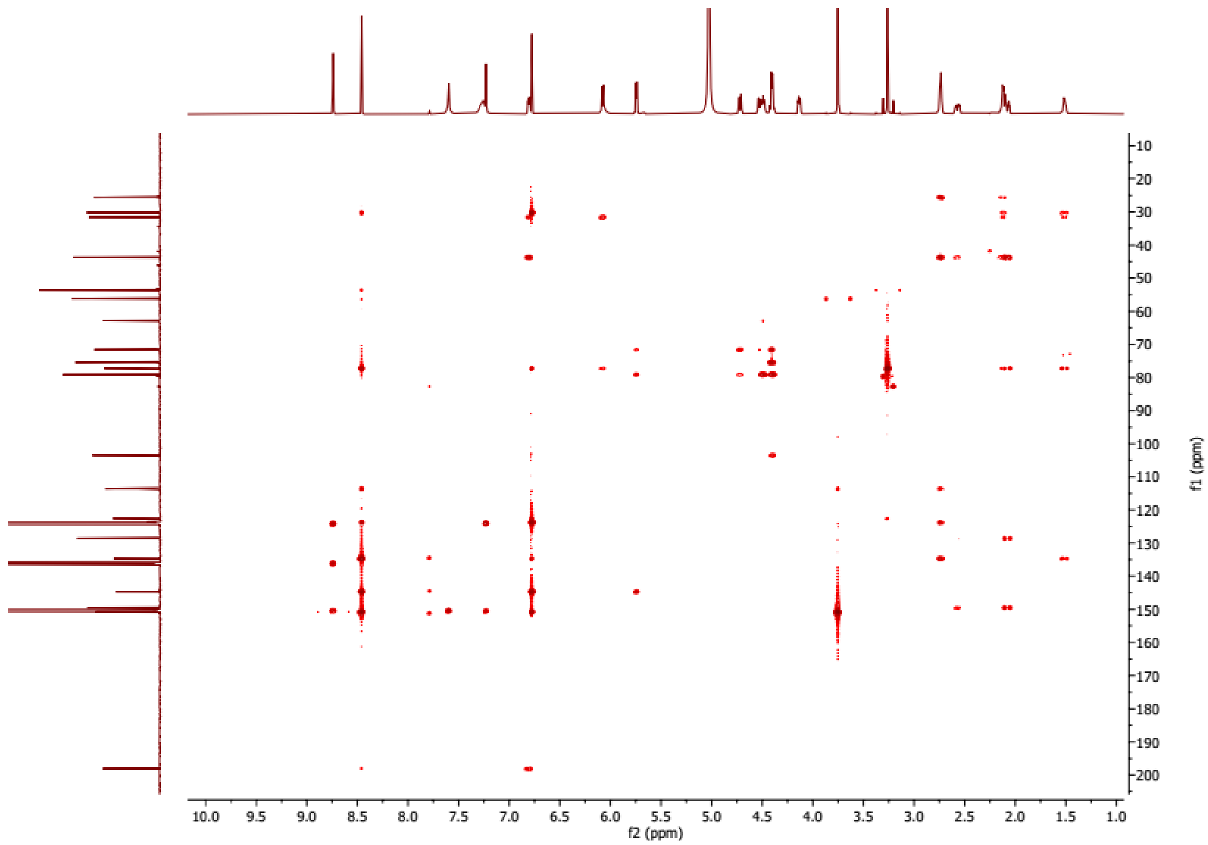

Figure S95. COSY NMR spectrum of compound 10

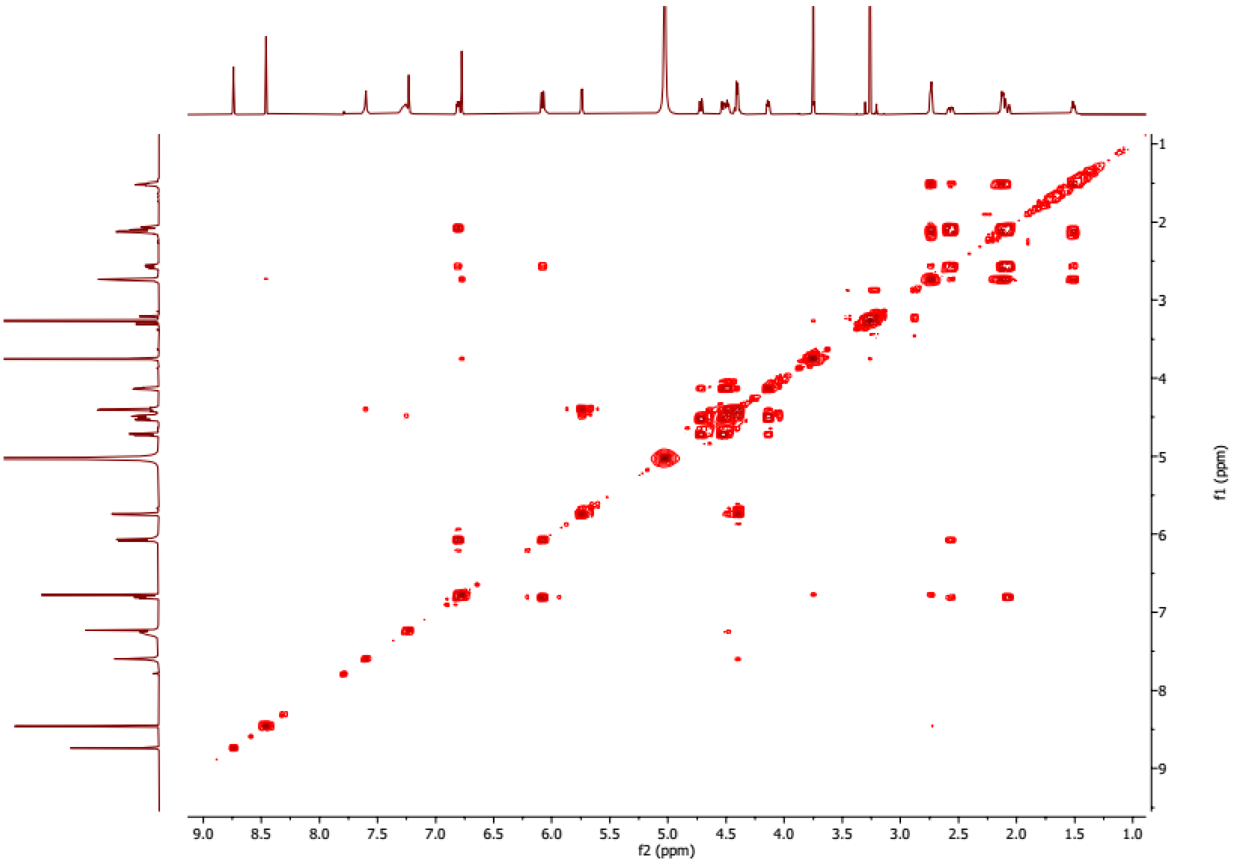

Figure S96. NOESY NMR spectrum of compound 10

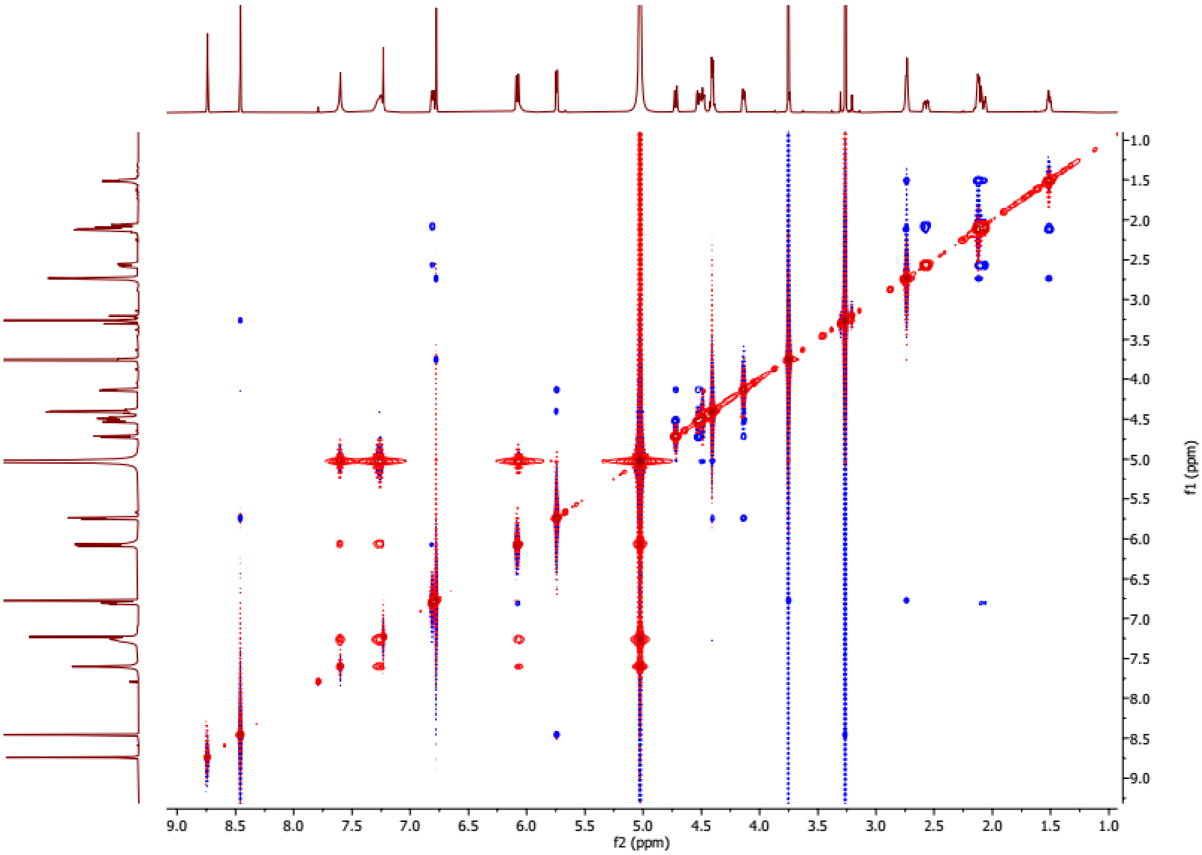

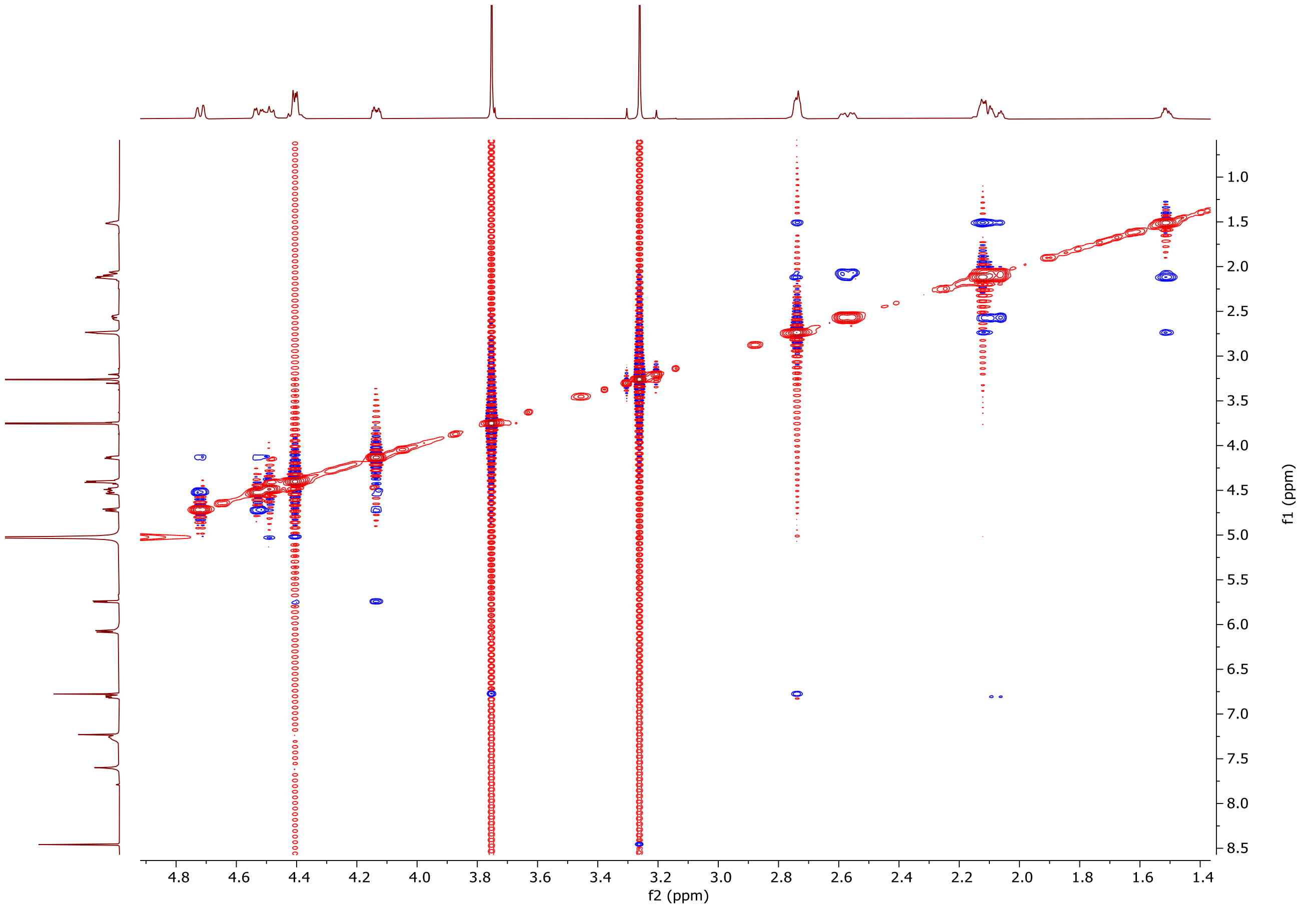

f2 (ppm)
f1 (ppm)

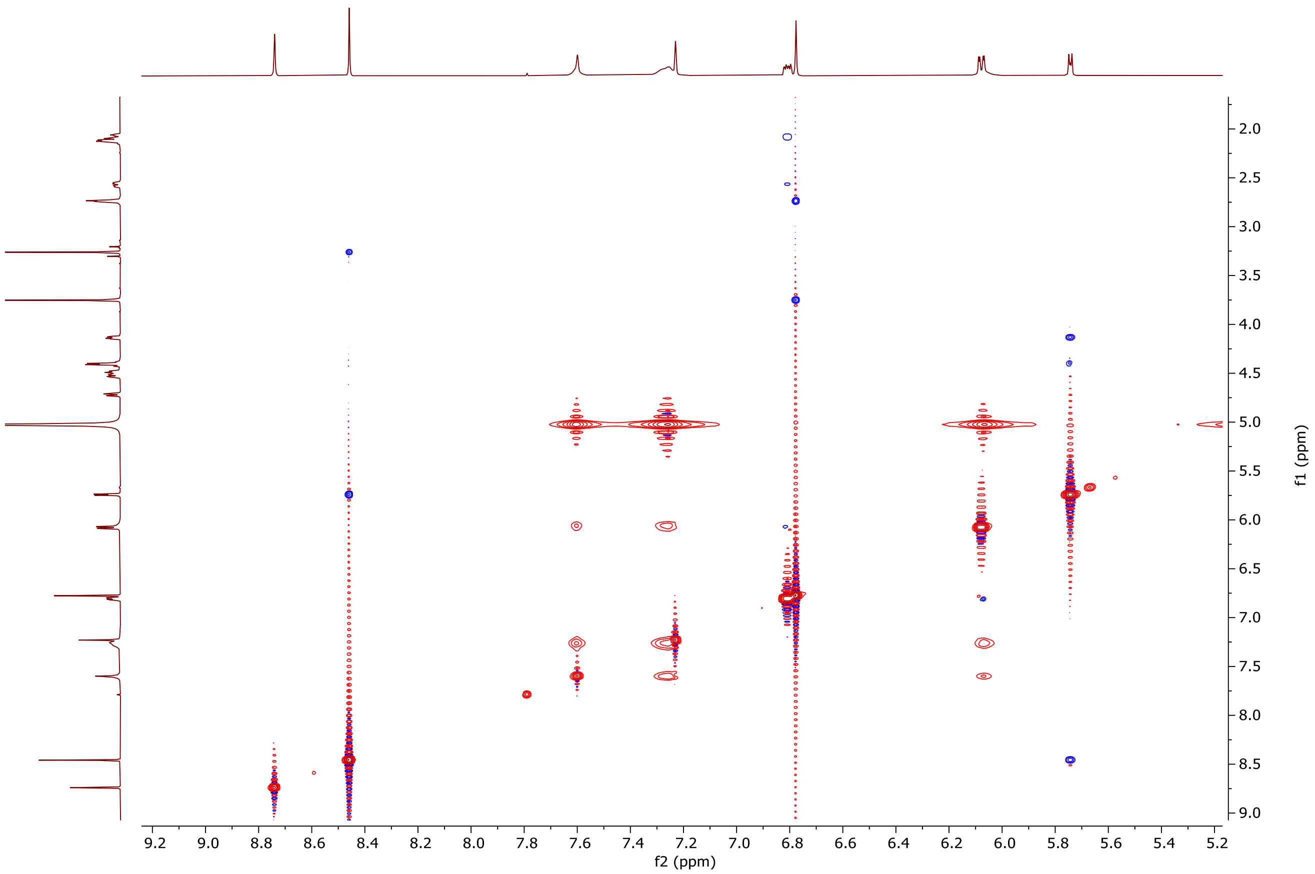

f2 (ppm)
f1 (ppm)

Figure S97. HR-ESI-MS spectrum of compound 10

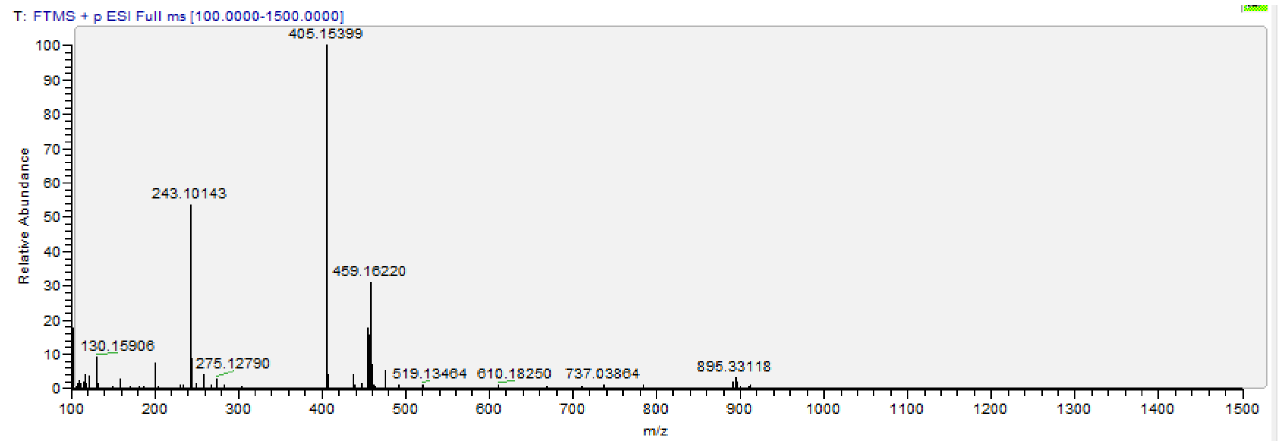

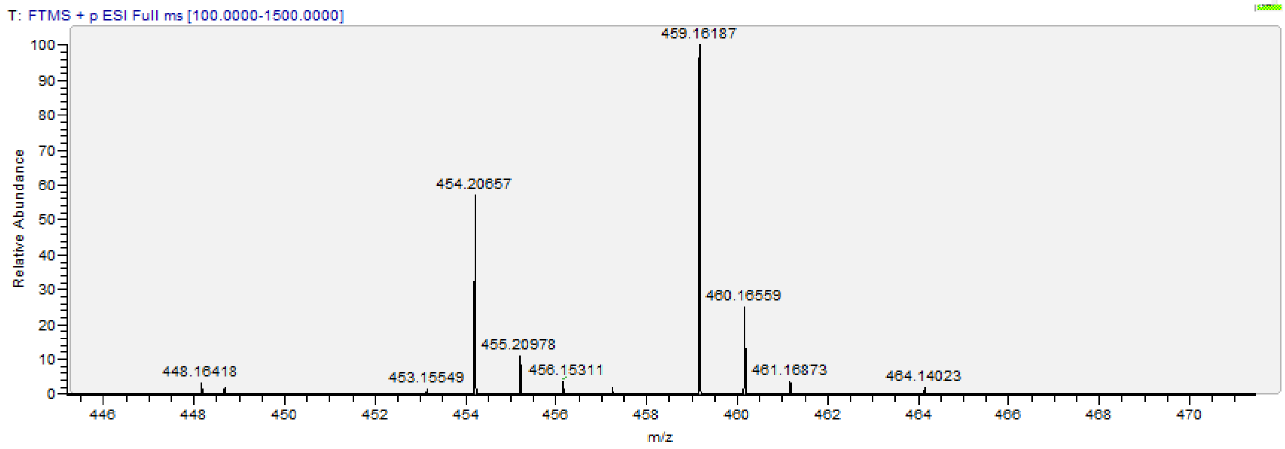

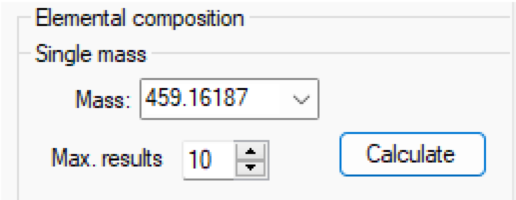

Idx	Formula	RDB	Delta ppm
1	$C_{22}H_{28}O_9Na$	8.5	-1.489

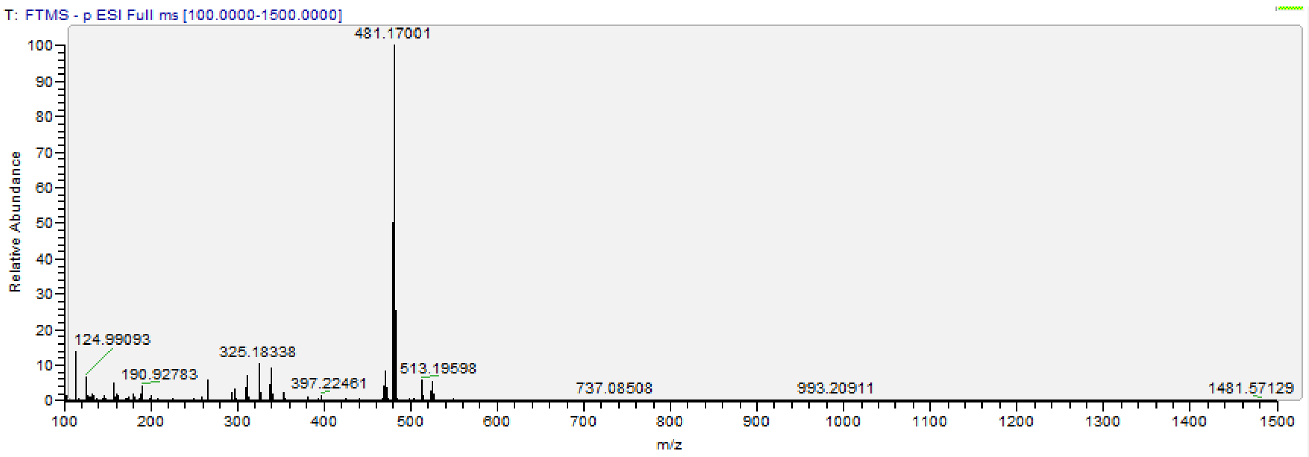

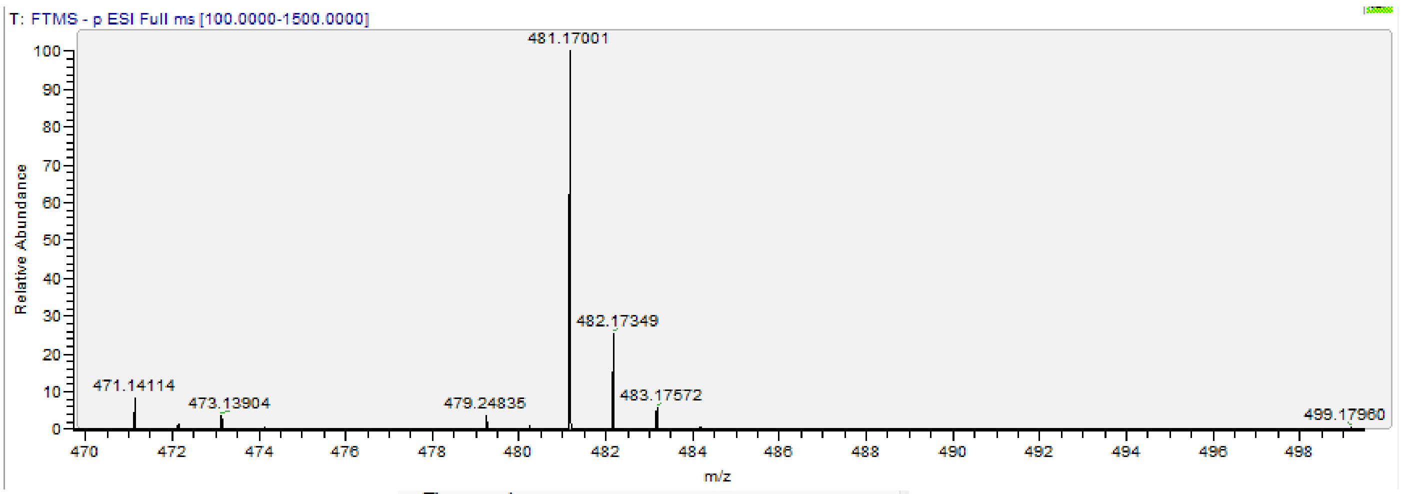

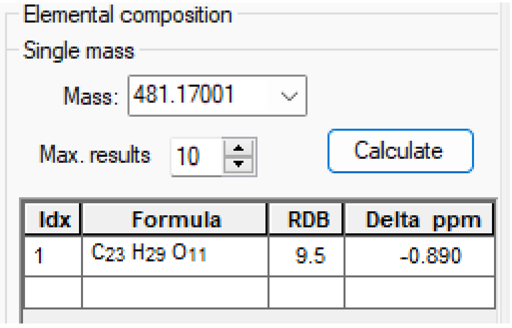

Figure S98. UV spectrum of compound 10

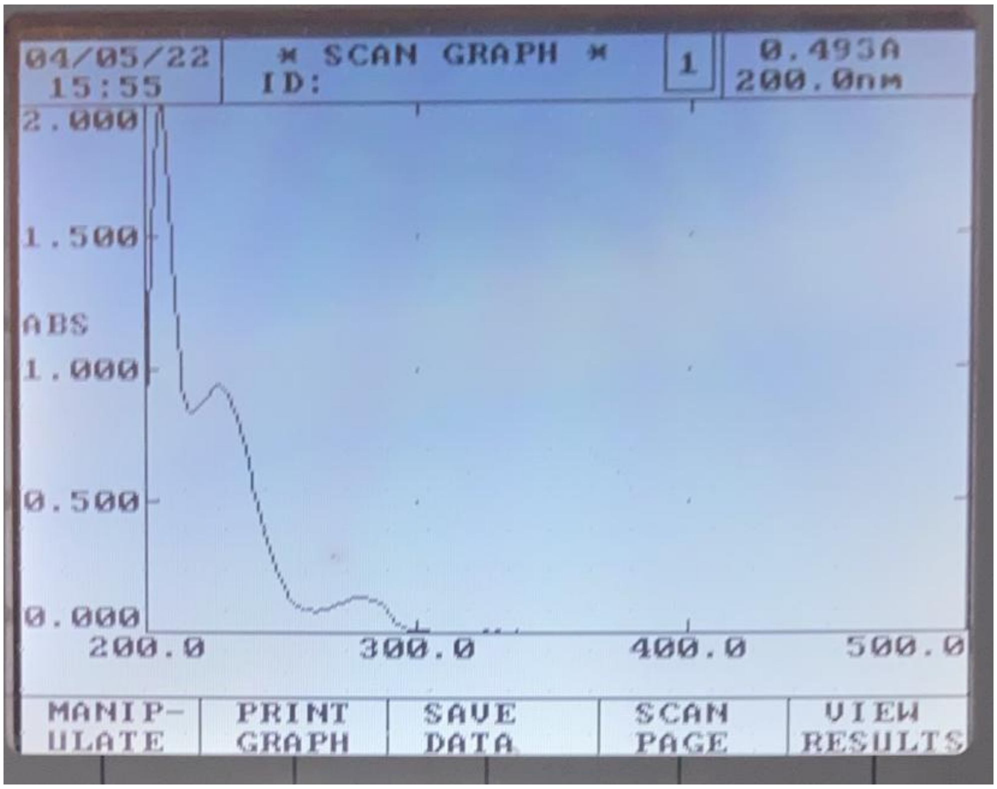

Figure S99. IR spectrum of compound 10

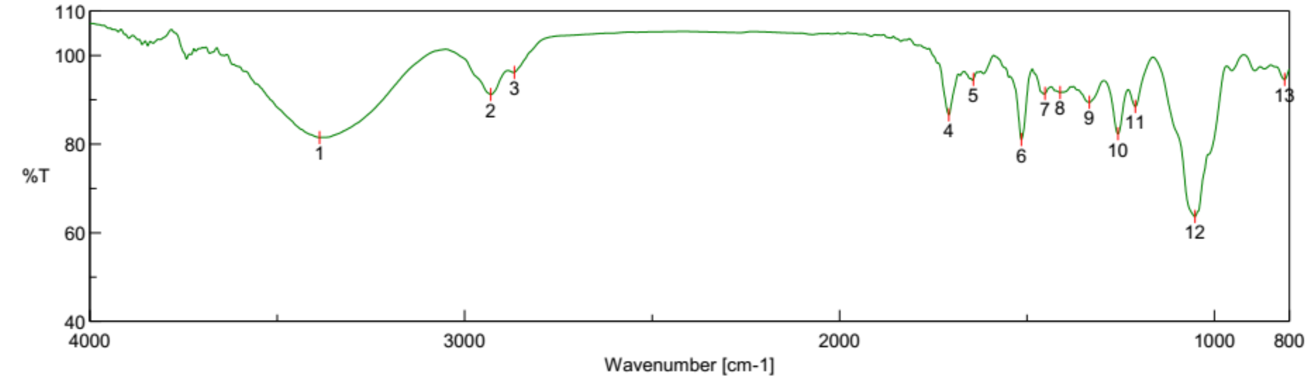

[Result of Peak Picking]

No.	Position	Intensity	No.	Position	Intensity	No.	Position	Intensity
1	3387.35	81.451	2	2930.31	91.1524	3	2867.63	96.1389
4	1708.62	86.5894	5	1643.05	94.5388	6	1514.81	80.9933
7	1452.14	91.3833	8	1411.64	91.5883	9	1333.53	89.3975
10	1257.36	82.2853	11	1211.08	88.4551	12	1051.98	63.6391
13	812.849	94.541	14	589.147	88.9637	15	486.938	61.383
16	454.154	47.0112						

Figure S100. ^{1}H-NMR spectrum of compound 11

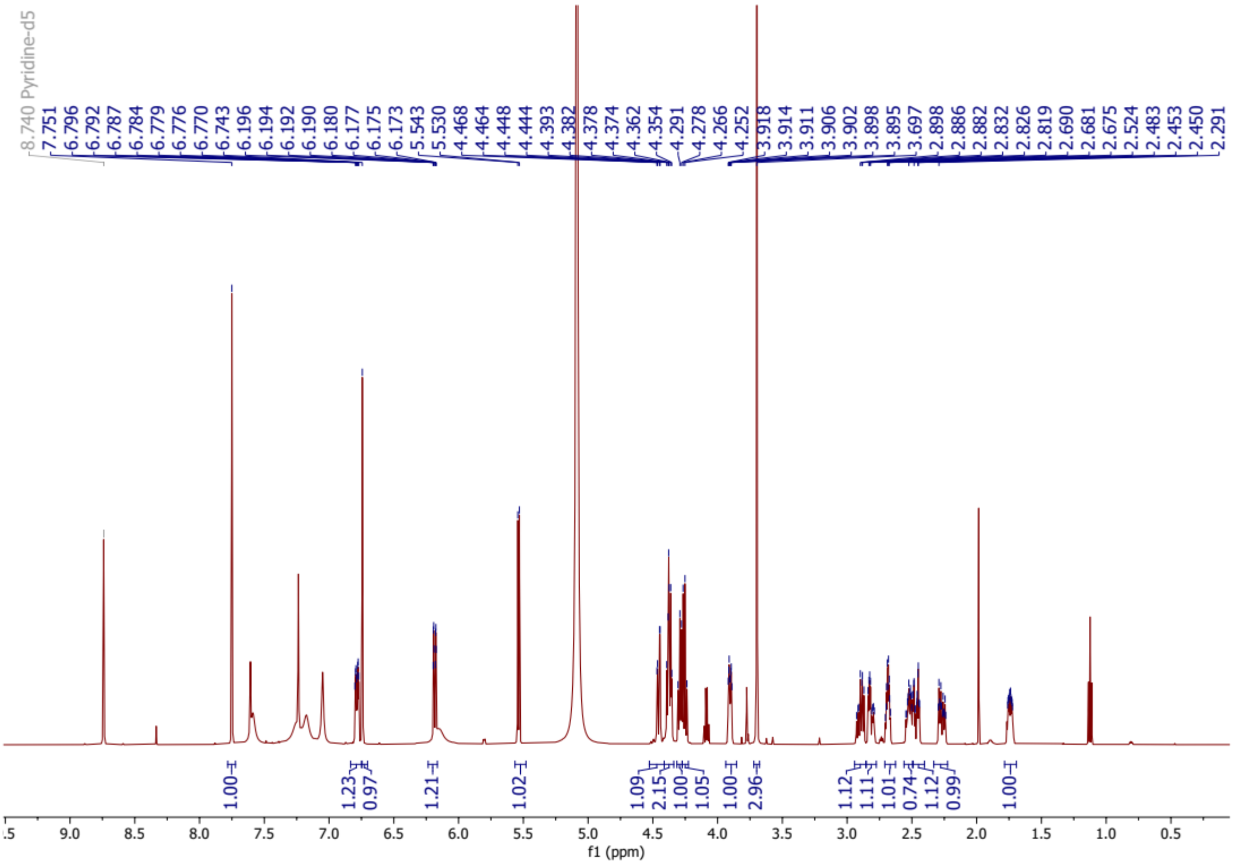

Figure S101. ^{13}C-NMR spectrum of compound 11

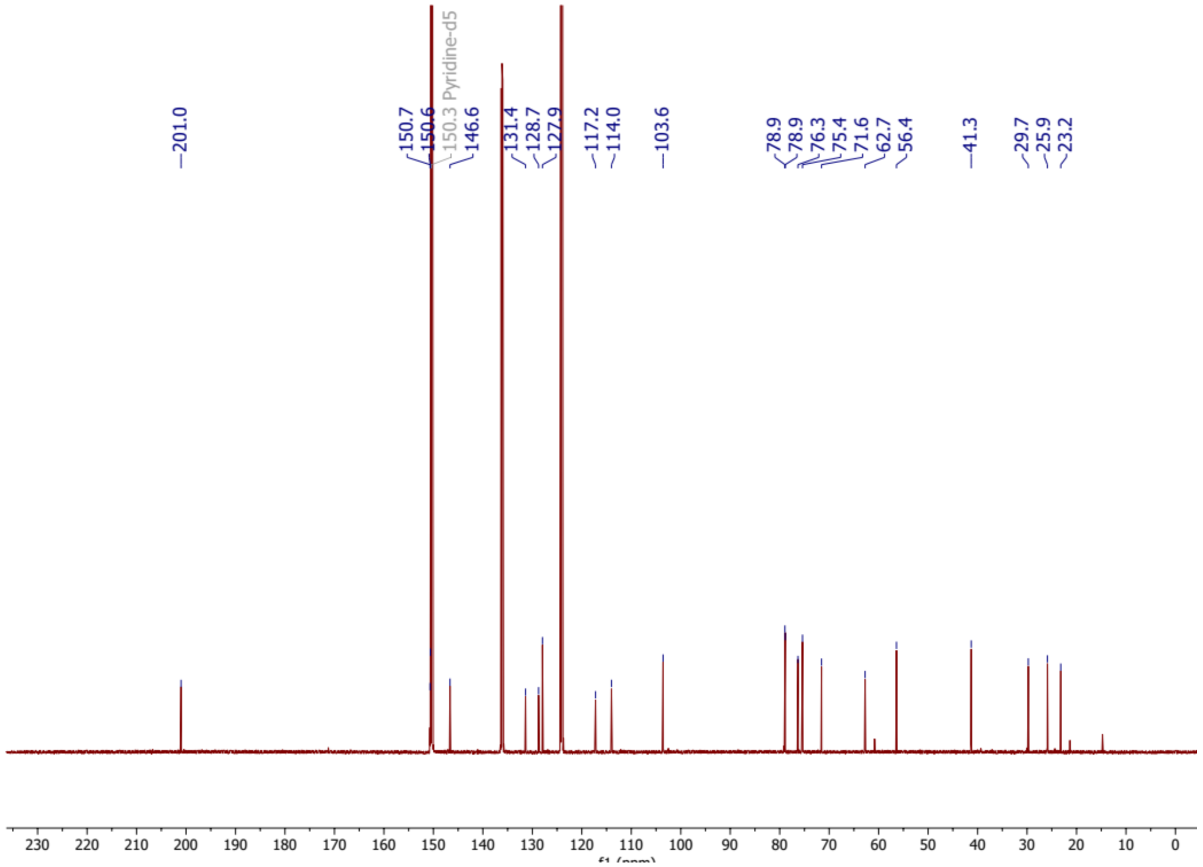

Figure S102. DEPT NMR spectrum of compound 11

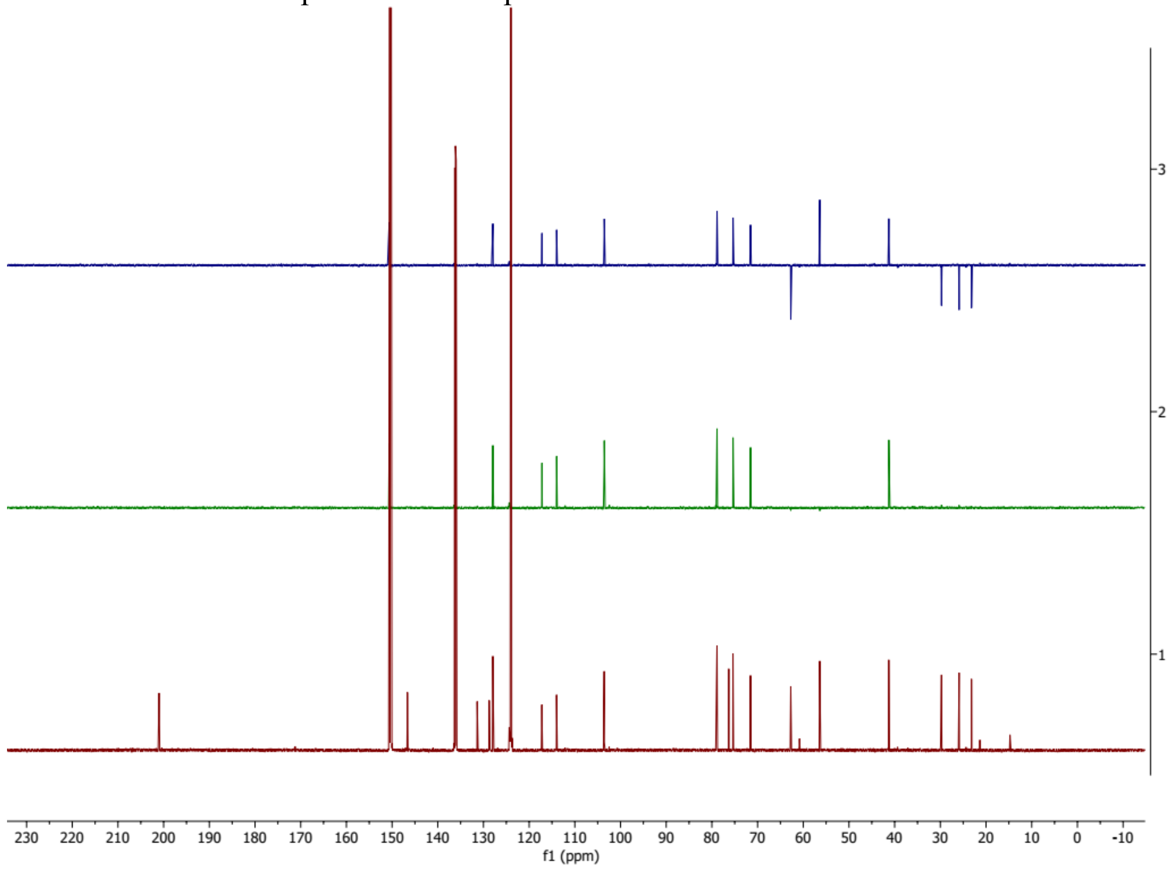

Figure S103. HSQC NMR spectrum of compound 11

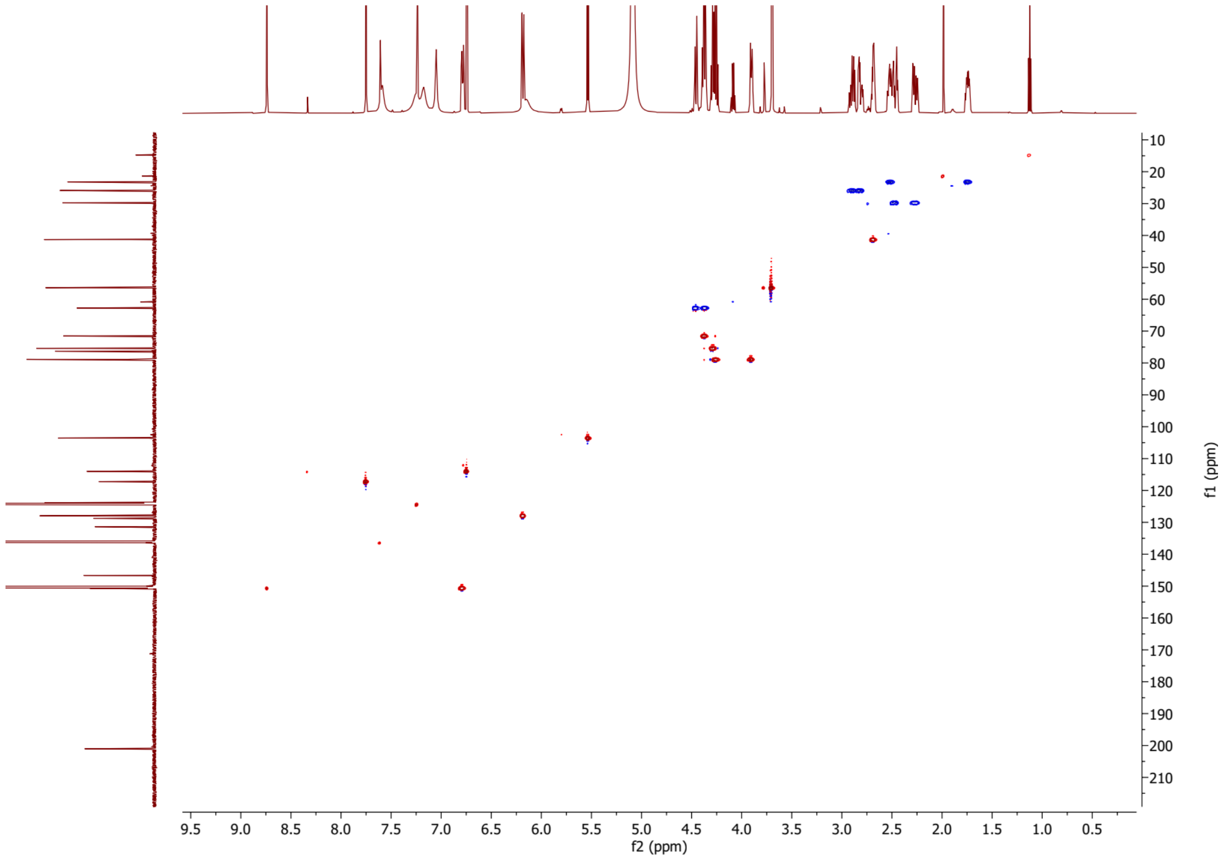

Figure S104. HMBC NMR spectrum of compound 11

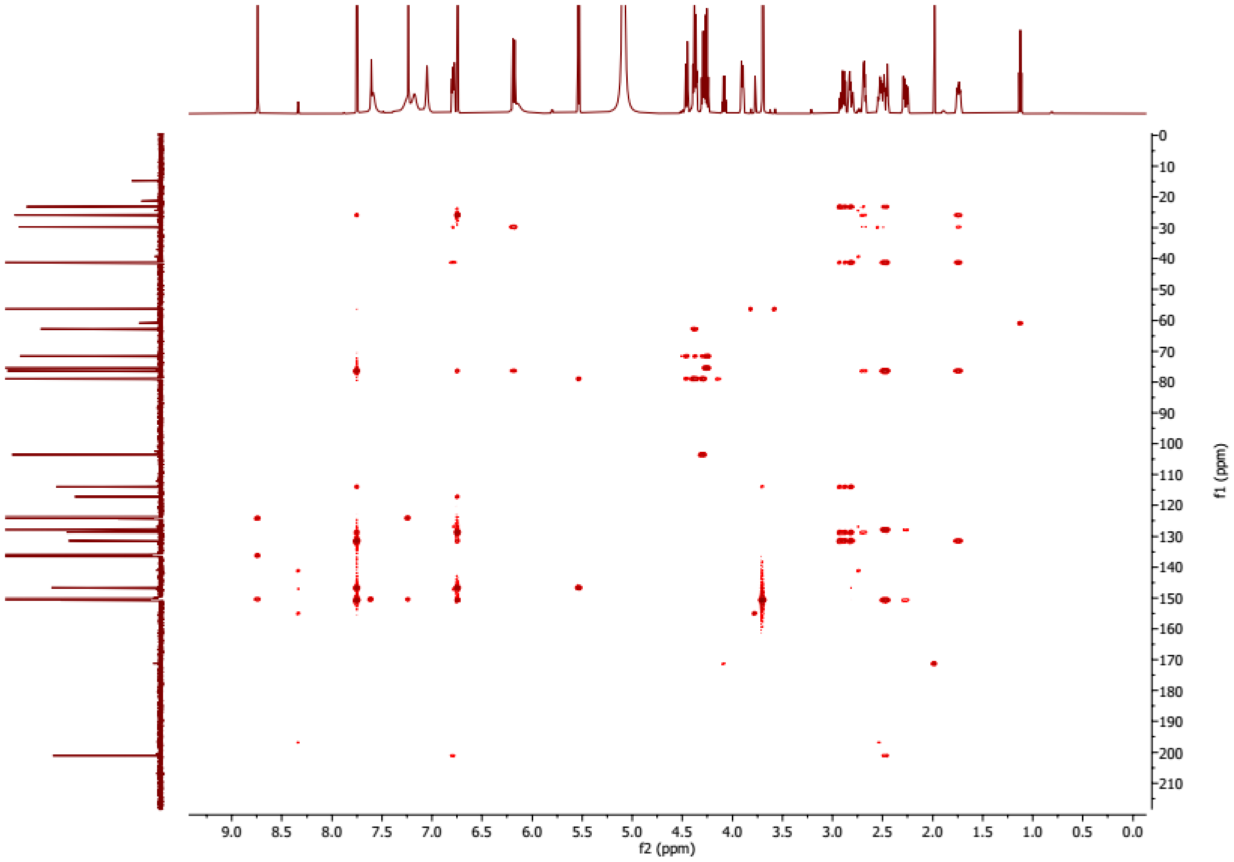

Figure S105. COSY NMR spectrum of compound 11

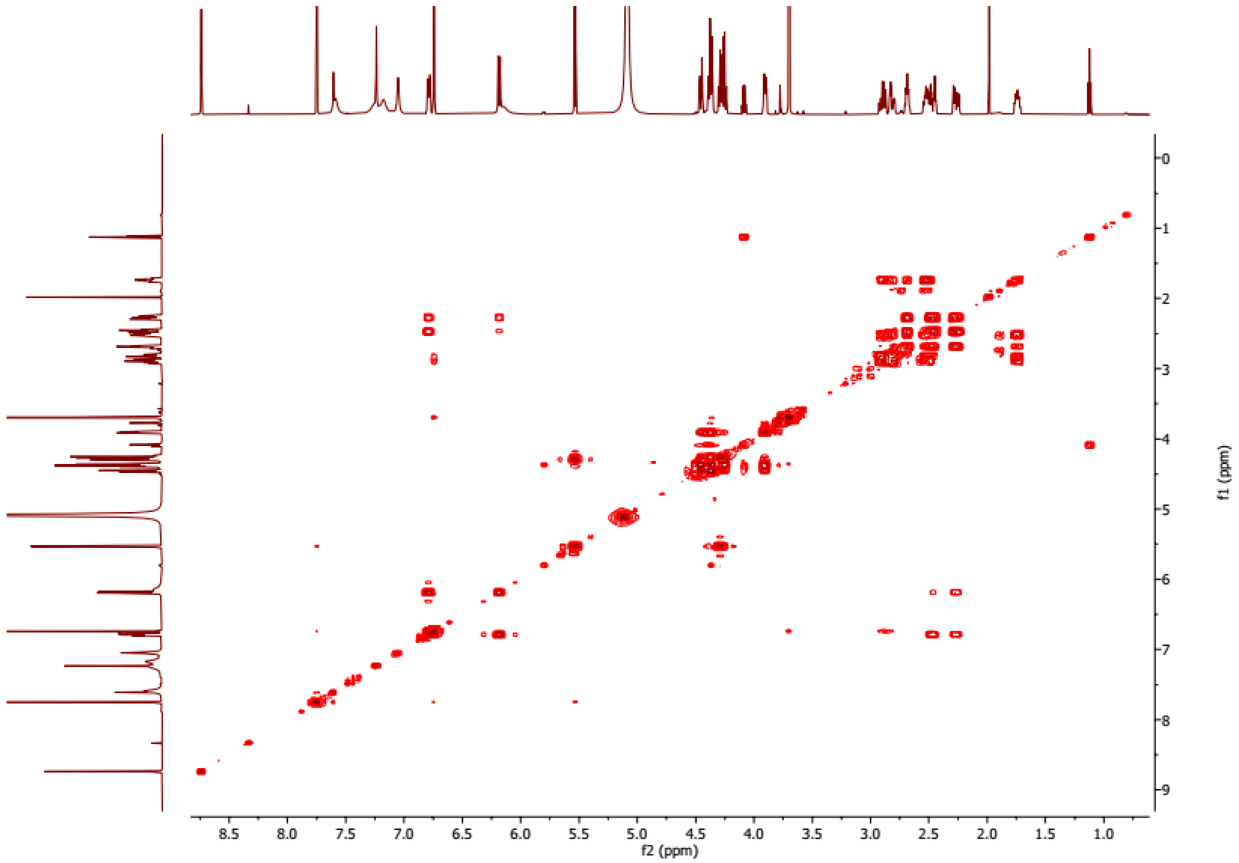

Figure S106. NOESY NMR spectrum of compound 11

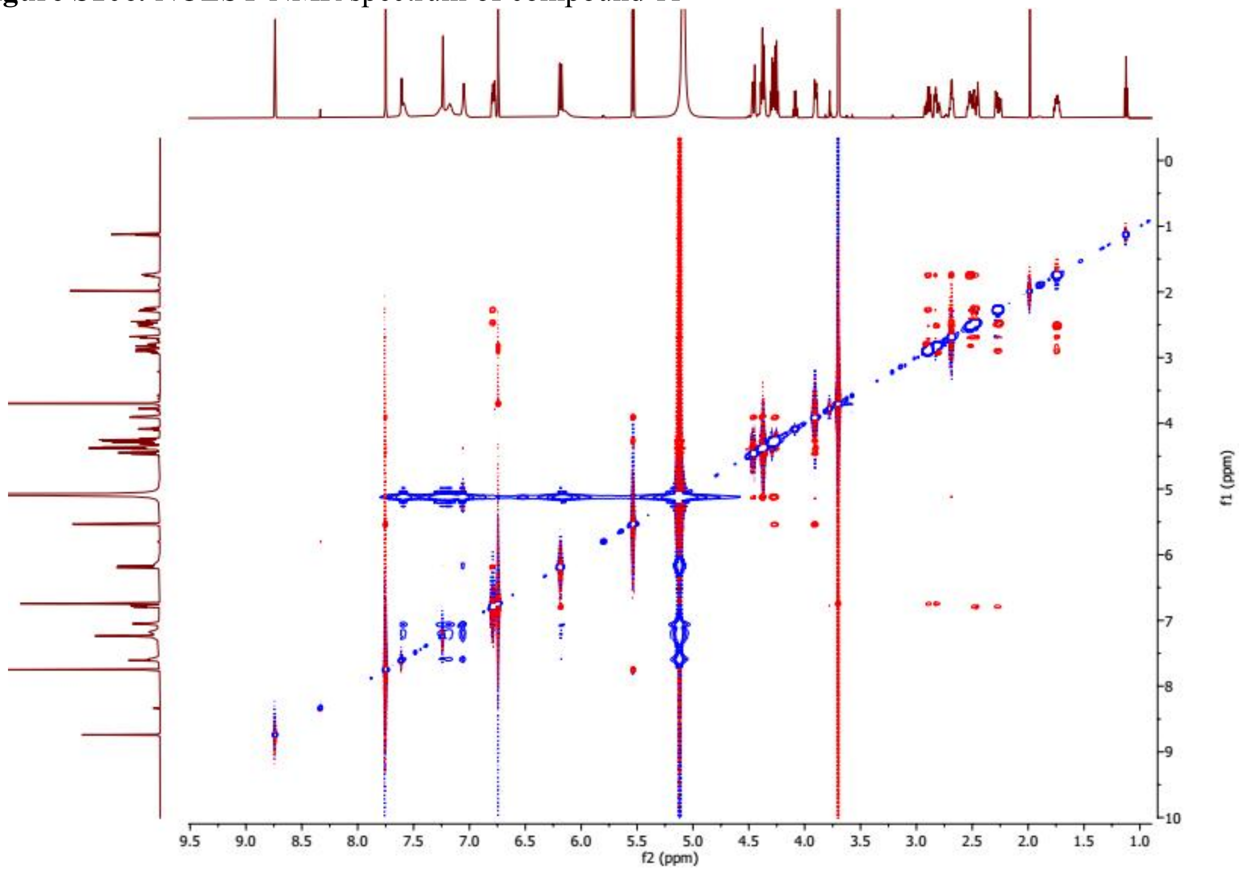

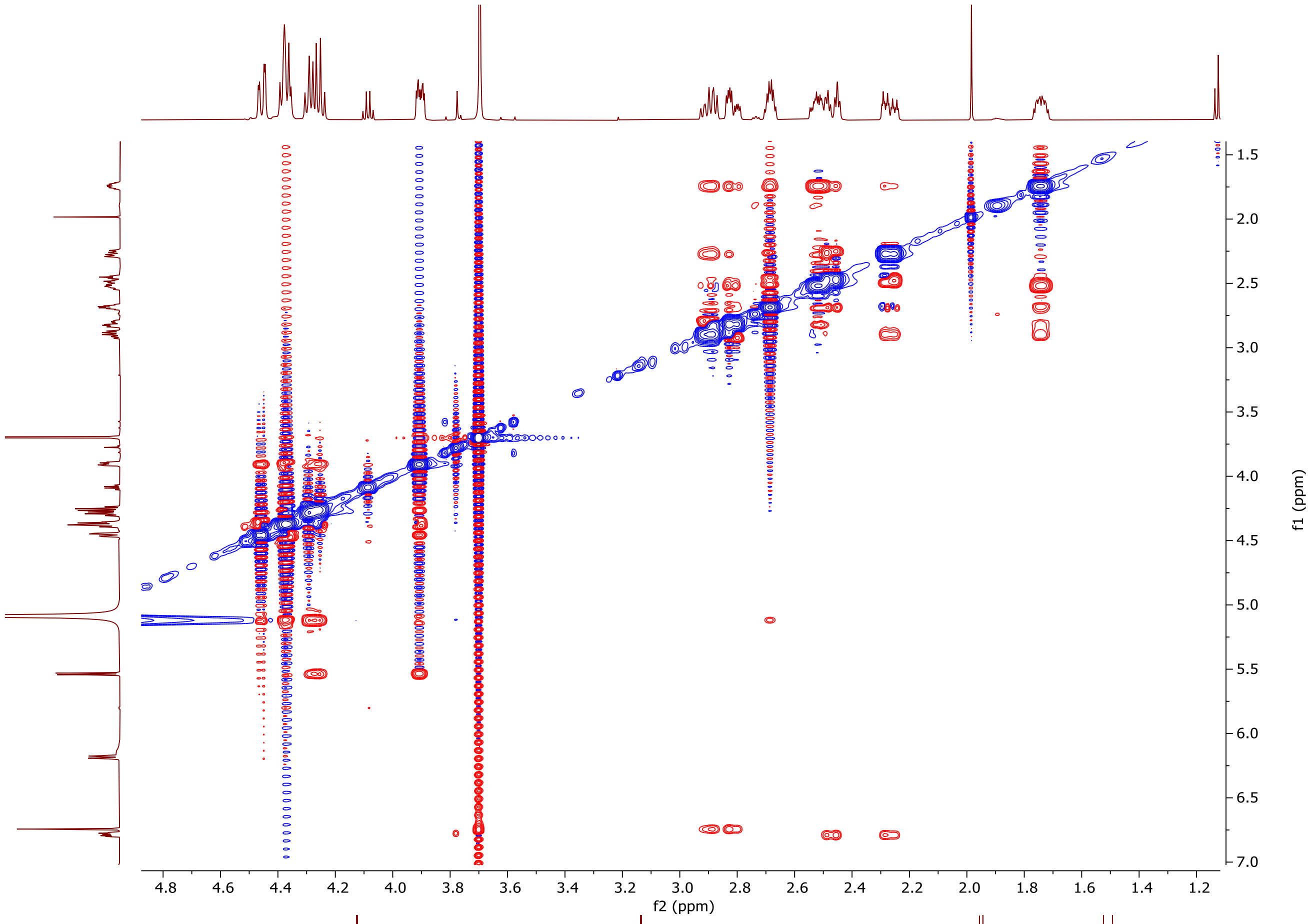

f2 (ppm)
4.8 4.6 4.4 4.2 4.0 3.8 3.6 3.4 3.2 3.0 2.8 2.6 2.4 2.2 2.0 1.8 1.6 1.4 1.2
f1 (ppm)
1.5 2.0 2.5 3.0 3.5 4.0 4.5 5.0 5.5 6.0 6.5 7.0

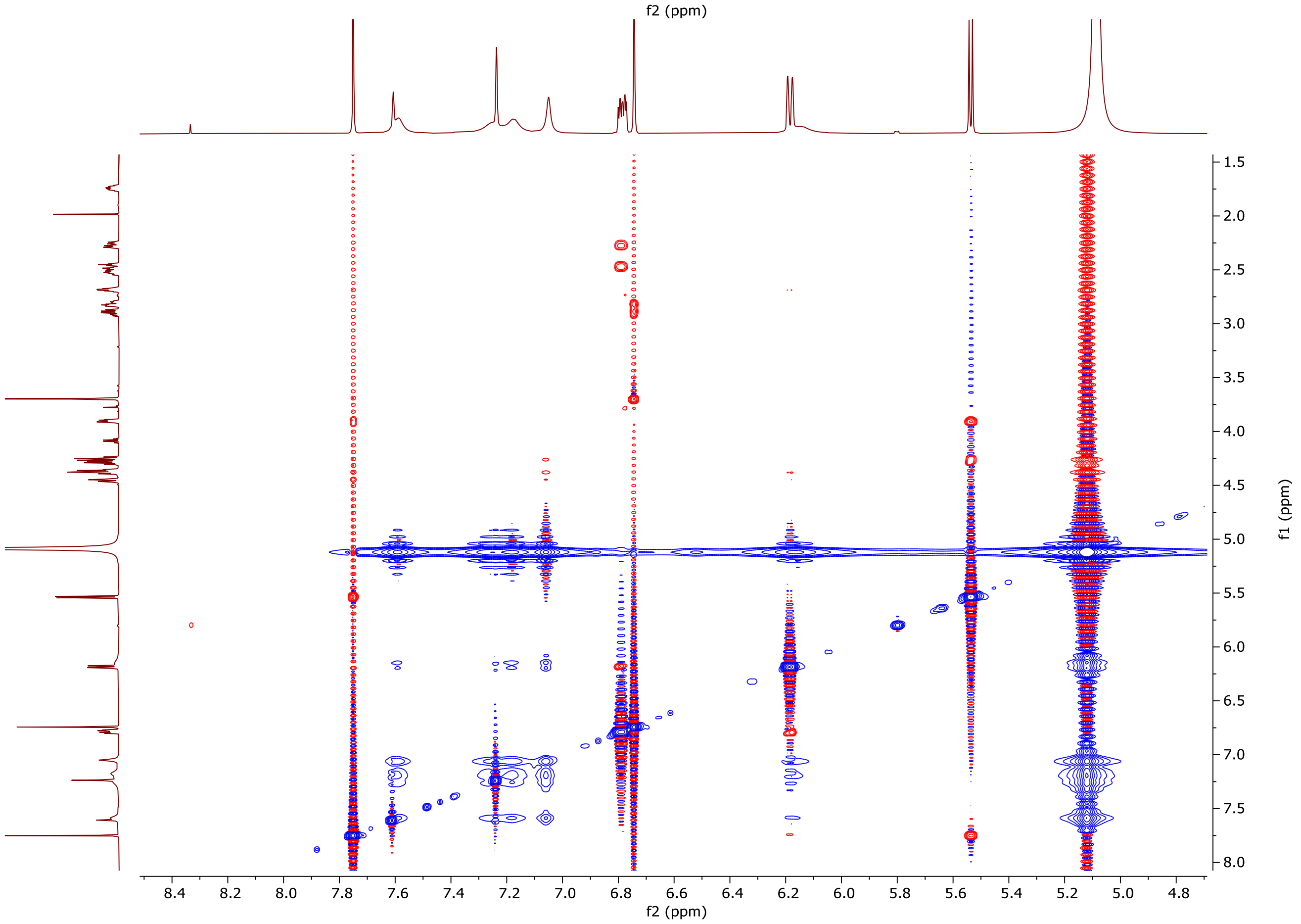

f2 (ppm)
8.4 8.2 8.0 7.8 7.6 7.4 7.2 7.0 6.8 6.6 6.4 6.2 6.0 5.8 5.6 5.4 5.2 5.0 4.8
f1 (ppm)
1.5 2.0 2.5 3.0 3.5 4.0 4.5 5.0 5.5 6.0 6.5 7.0 7.5 8.0

Figure S107. HR-ESI-MS spectrum of compound 11

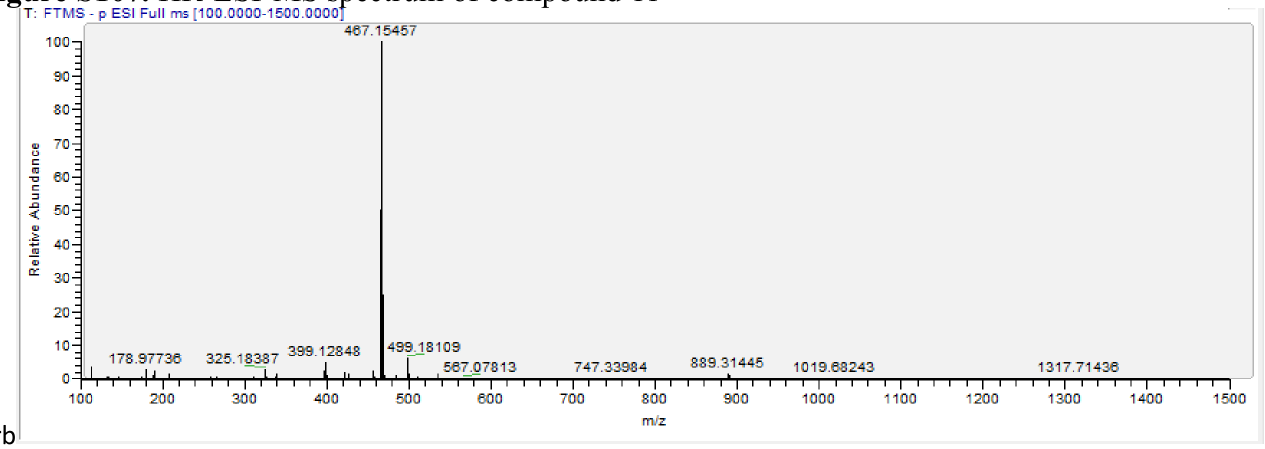

rb

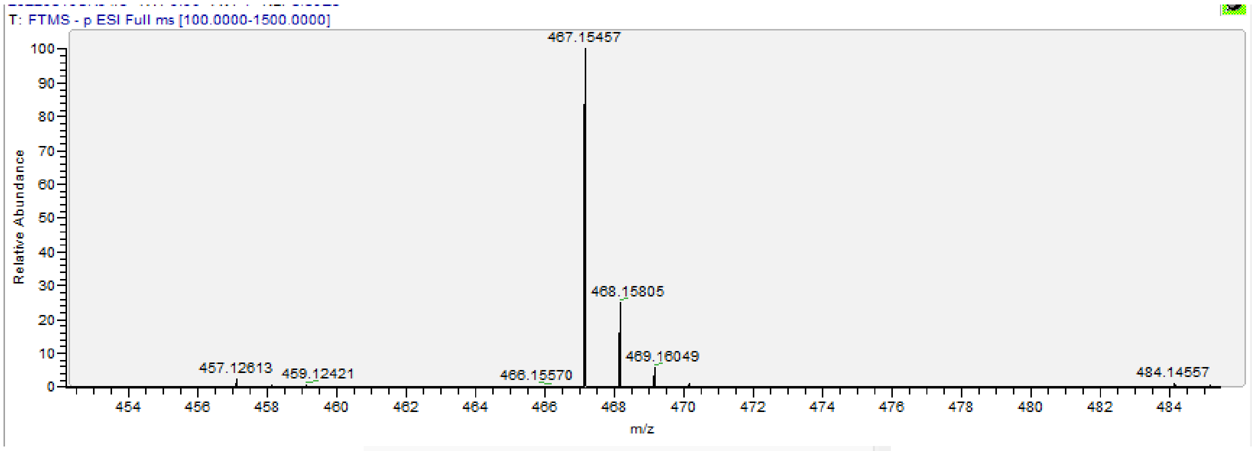

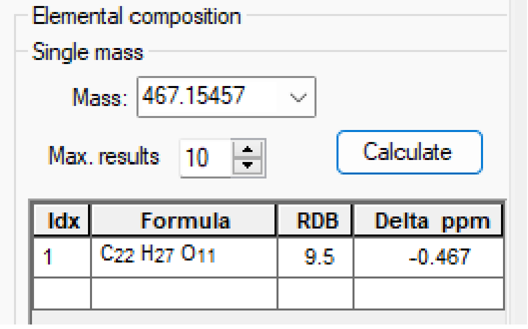

Idx	Formula	RDB	Delta ppm
1	$C_{22}H_{27}O_{11}$	9.5	-0.467

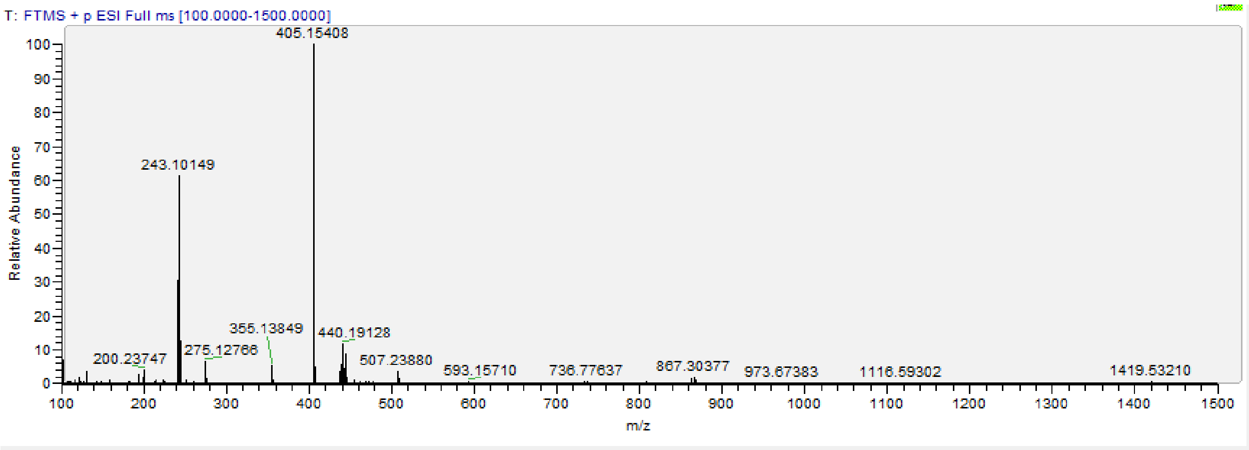

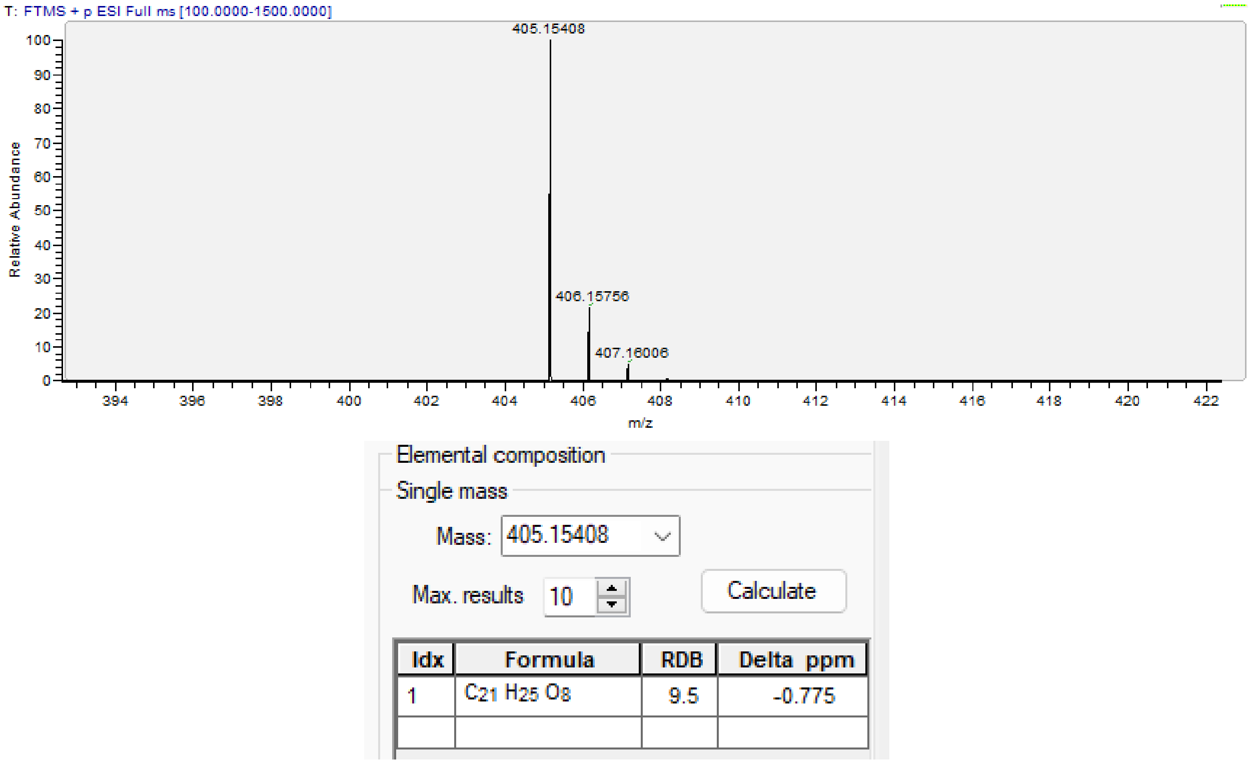

Figure S108. UV spectrum of compound 11

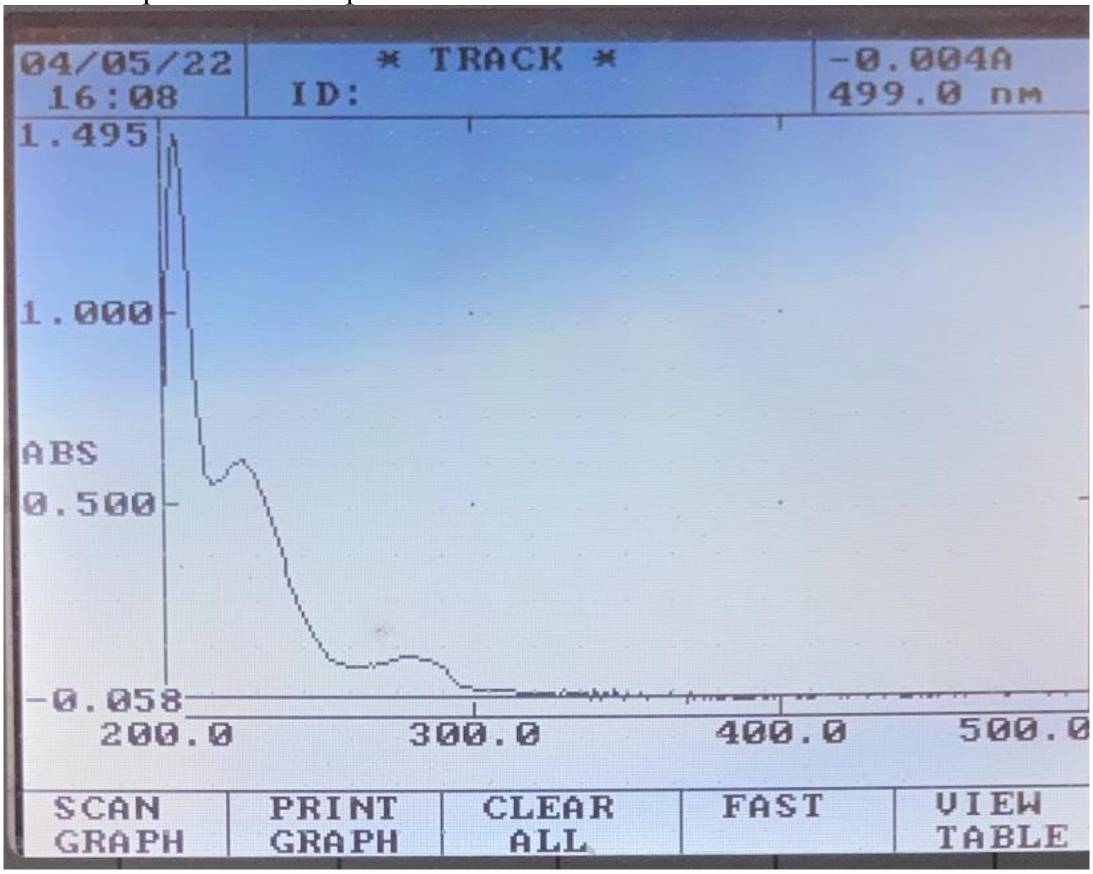

Figure S109. IR spectrum of compound 11

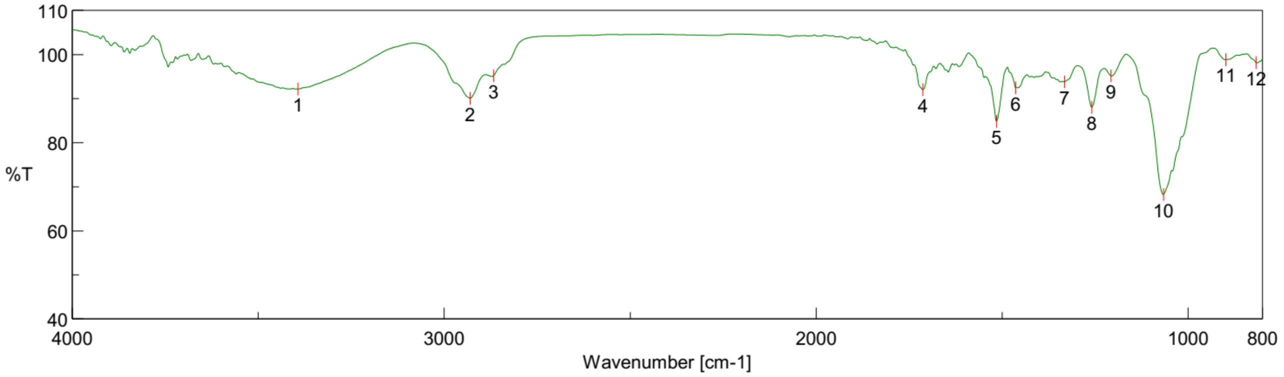

[Result of Peak Picking]

No.	Position	Intensity	No.	Position	Intensity	No.	Position	Intensity
1	3393.14	92.1475	2	2930.31	90.0377	3	2867.63	95.1485
4	1713.44	91.9985	5	1514.81	84.7888	6	1463.71	92.4195
7	1332.57	93.772	8	1259.29	88.0071	9	1207.22	95.144
10	1066.44	68.224	11	898.666	98.823	12	817.67	98.1719
13	676.892	95.0048	14	559.255	93.3342			

Figure S110. ^{1}H-NMR spectrum of compound 12

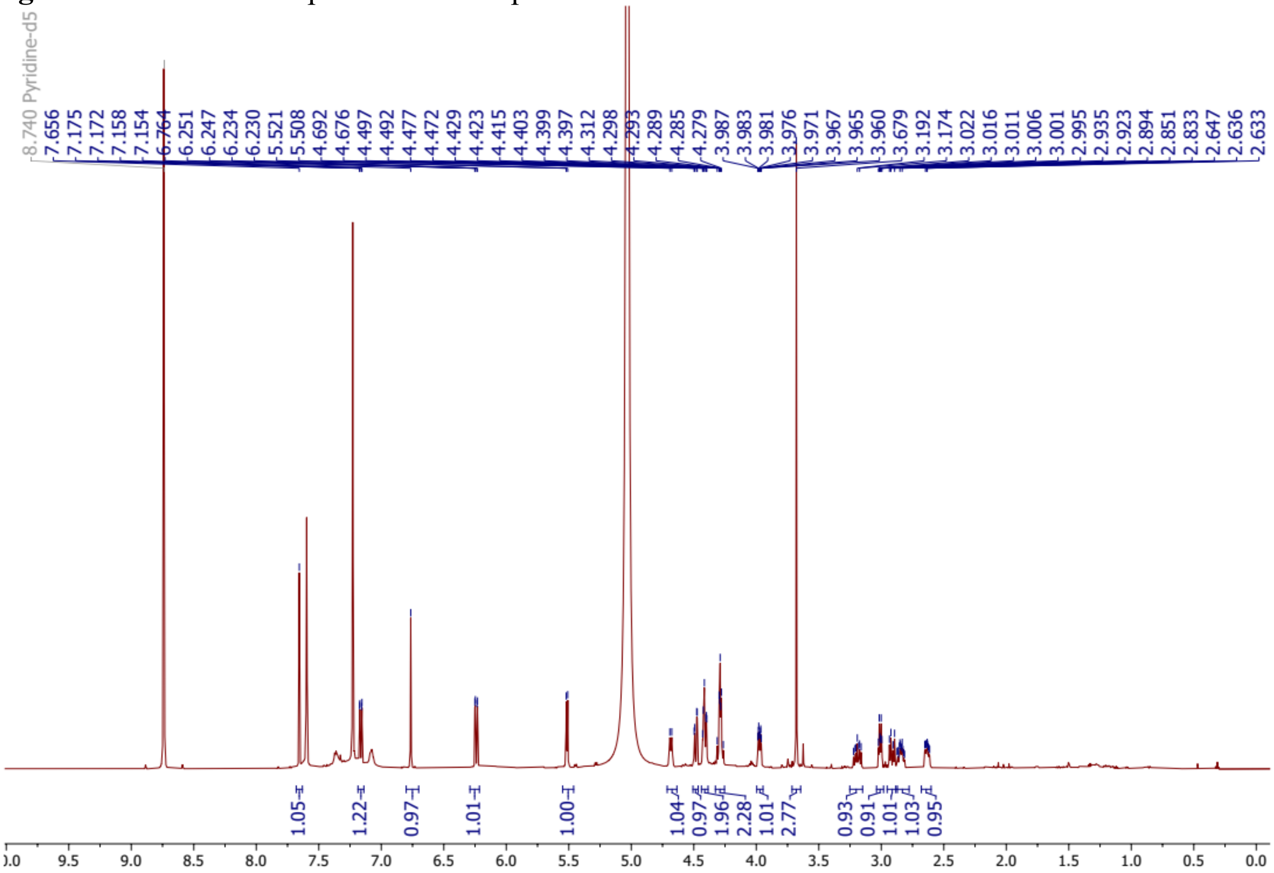

Figure S111. ^{13}C-NMR spectrum of compound 12

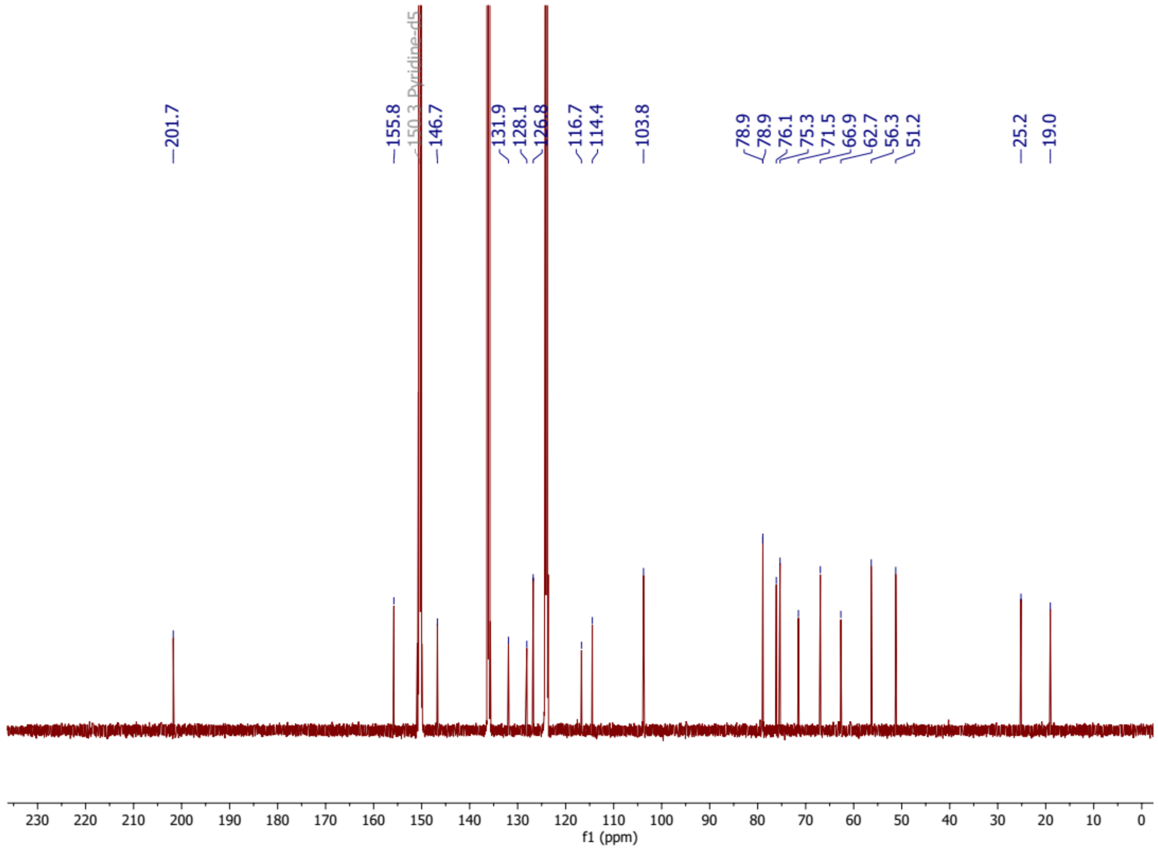

Figure S112. DEPT NMR spectrum of compound 12

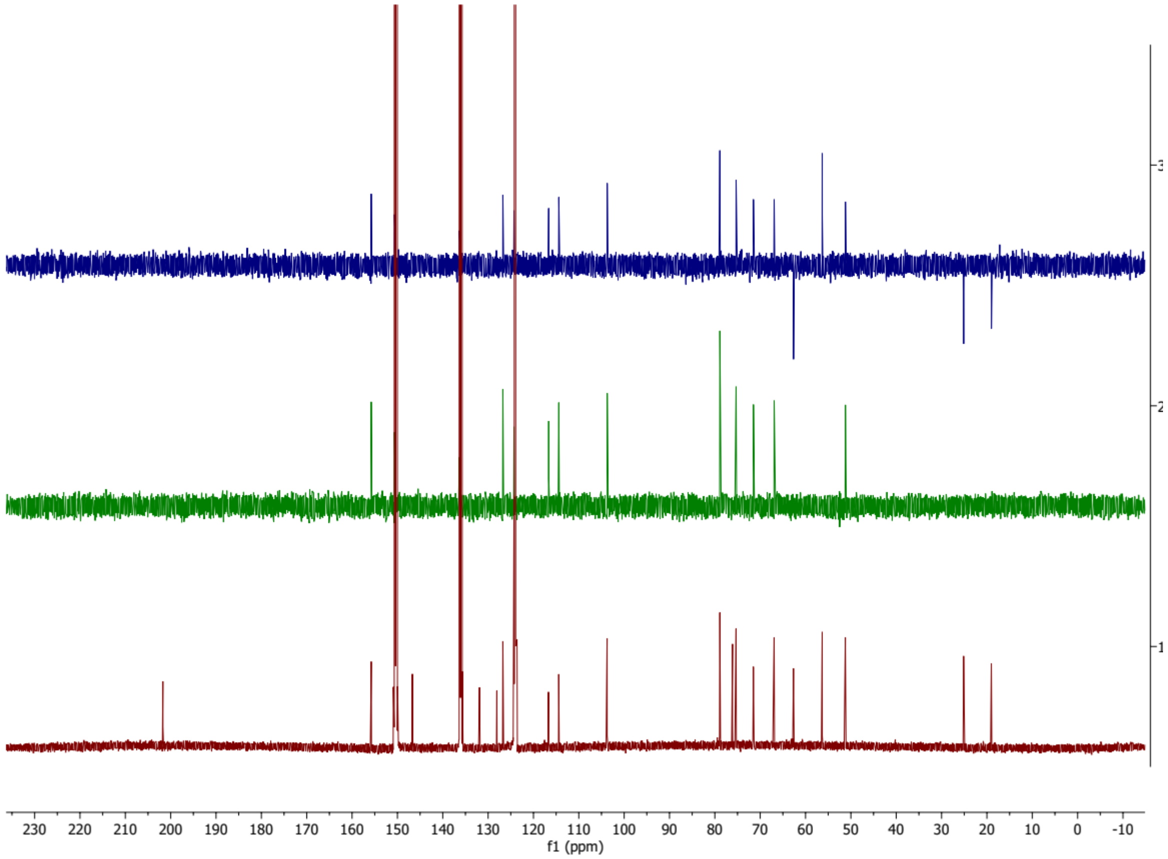

Figure S113. HSQC NMR spectrum of compound 12

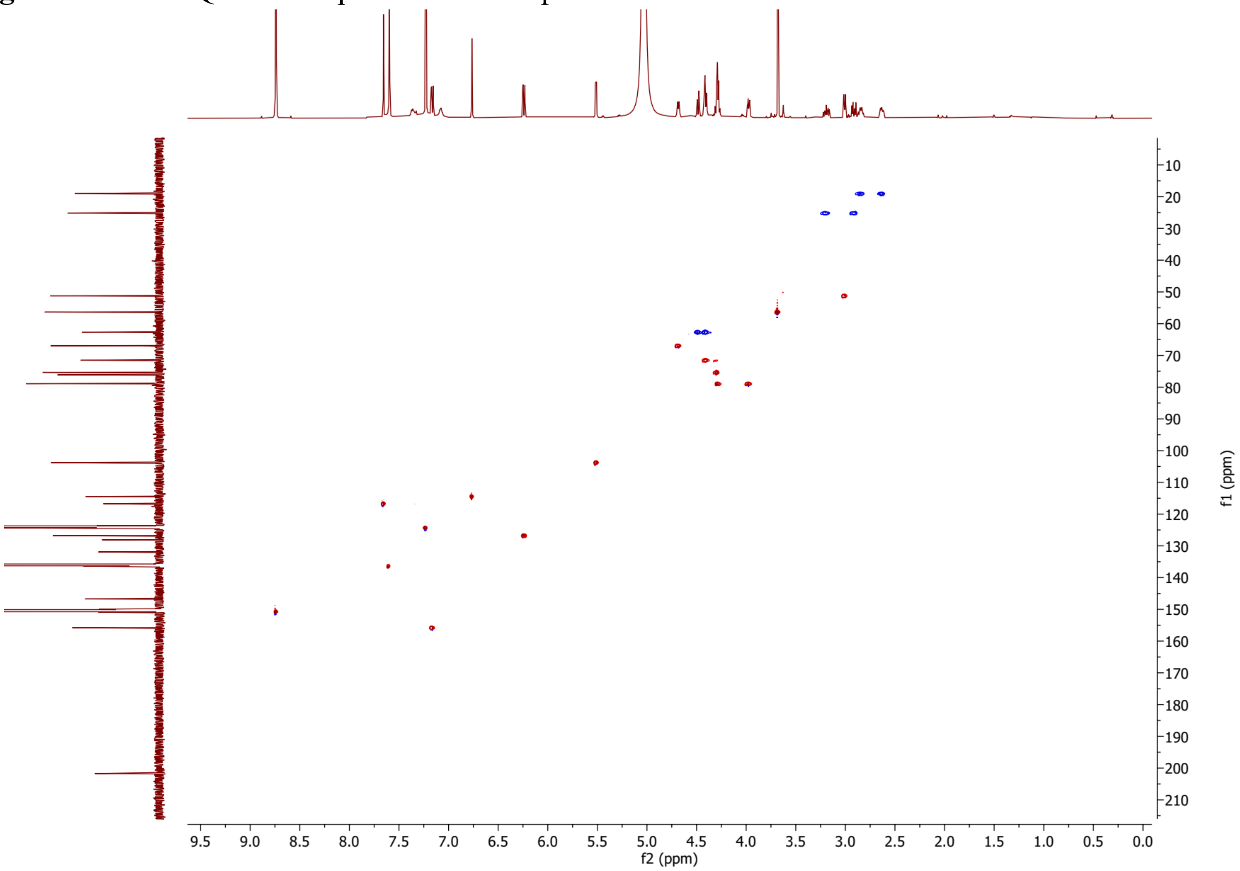

Figure S114. HMBC NMR spectrum of compound 12

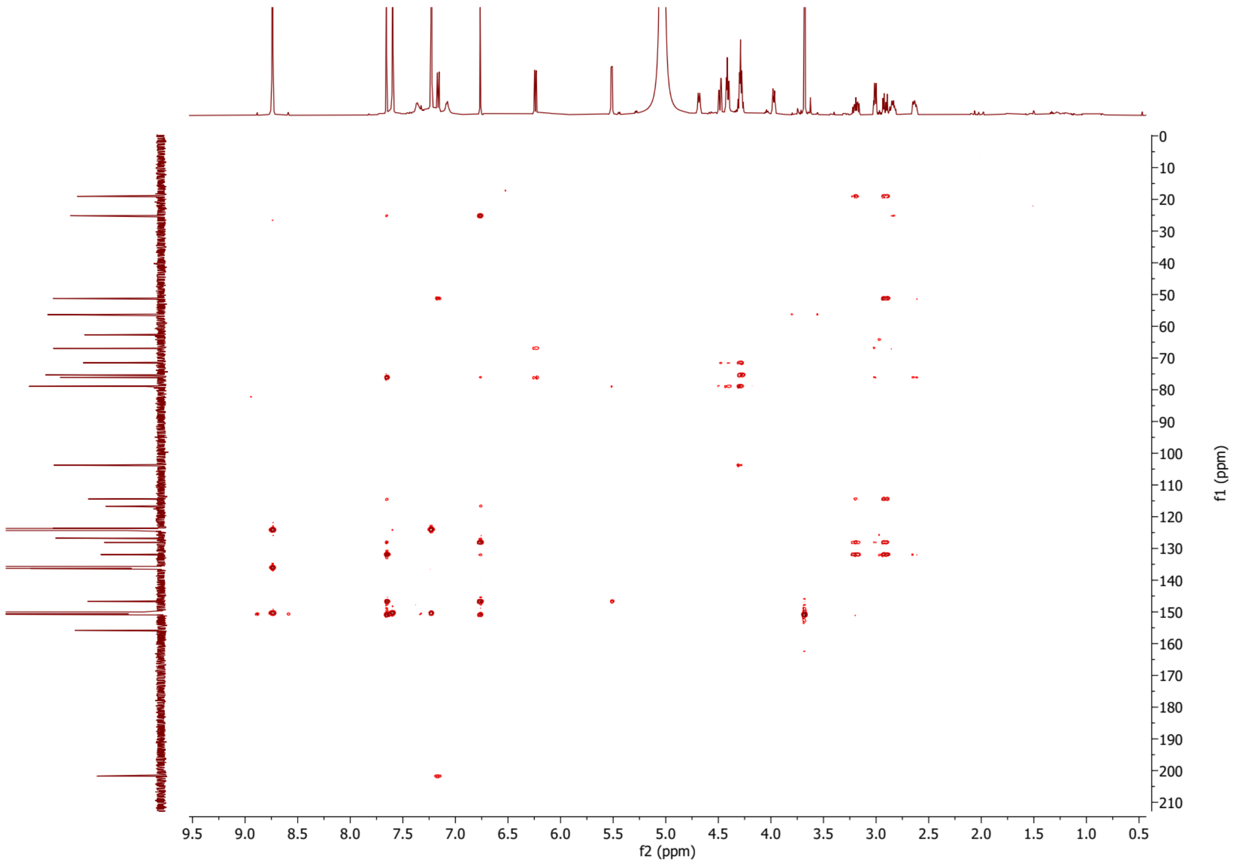

Figure S115. COSY NMR spectrum of compound 12

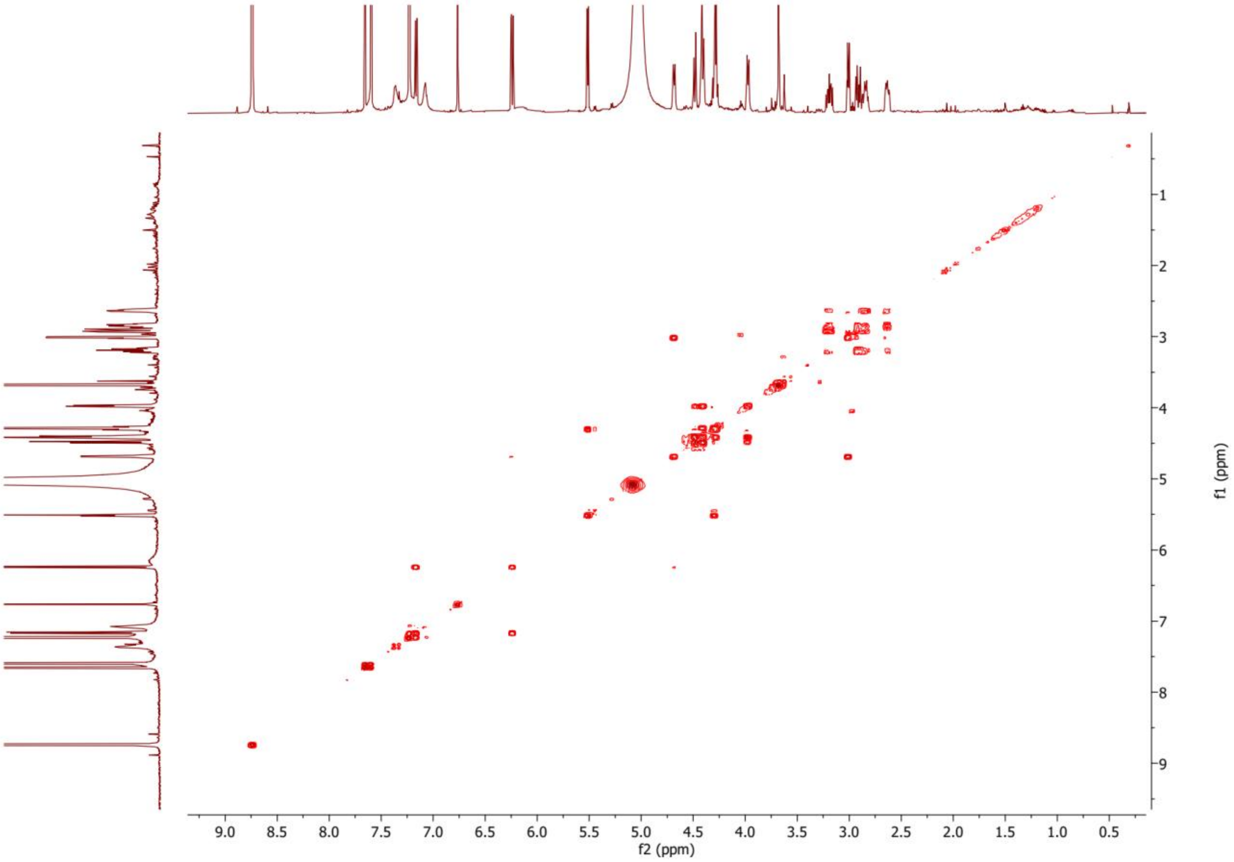

Figure S116. NOESY NMR spectrum of compound 12

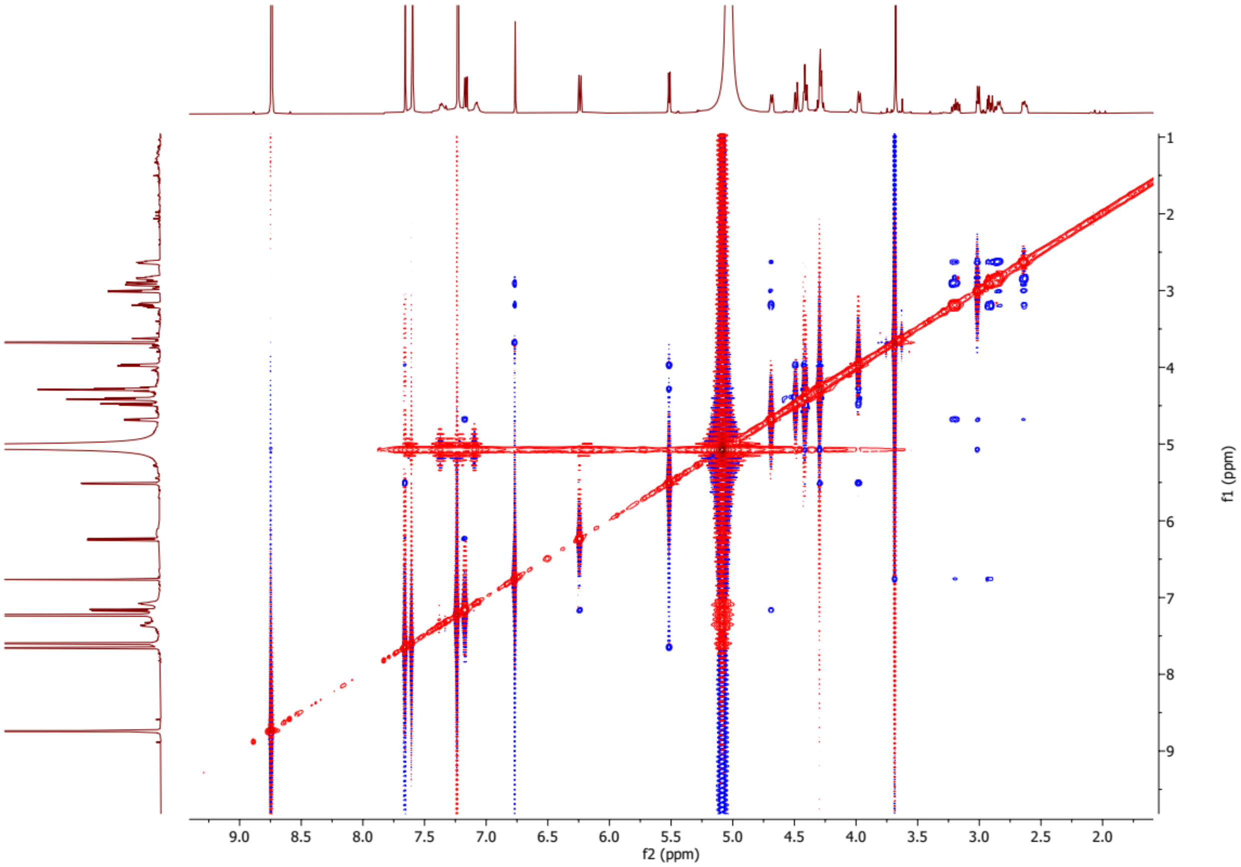

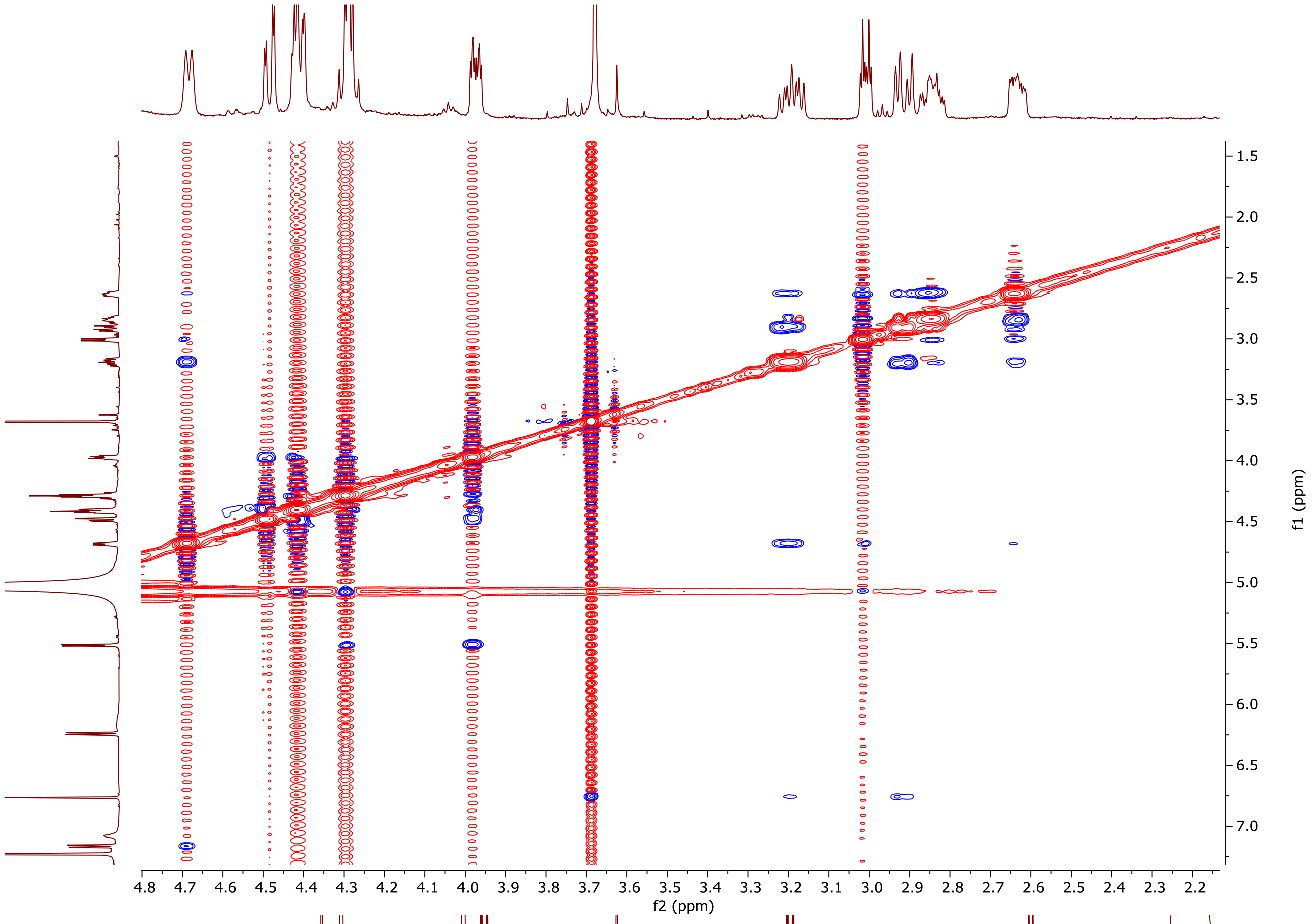
4.8
4.7
4.6
4.5
4.4
4.3
4.2
4.1
4.0
3.9
3.8
3.7
3.6
3.5
3.4
3.3
3.2
3.1
3.0
2.9
2.8
2.7
2.6
2.5
2.4
2.3
2.2
f2 (ppm)
f1 (ppm)
1.5
2.0
2.5
3.0
3.5
4.0
4.5
5.0
5.5
6.0
6.5
7.0

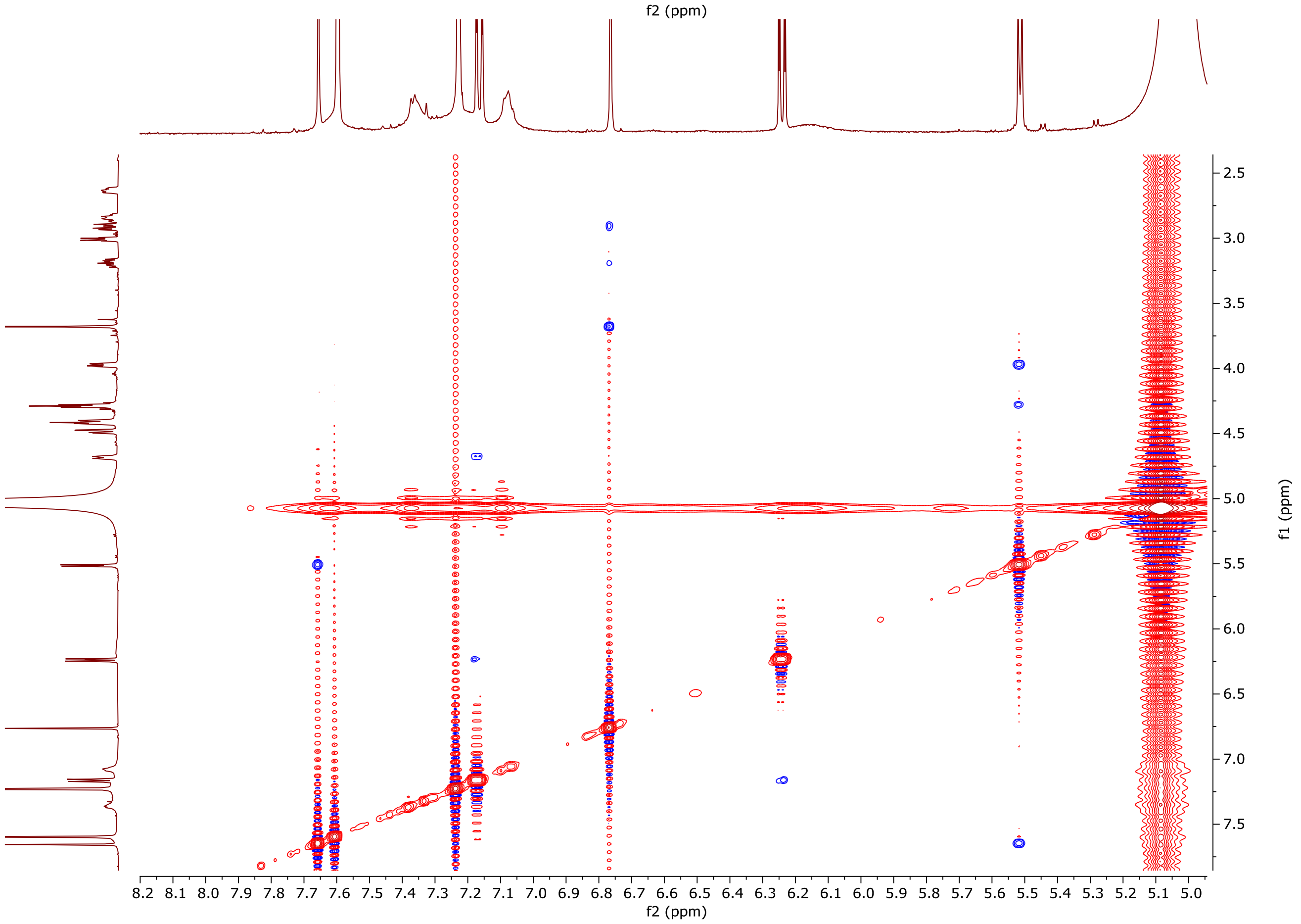
8.2
8.1
8.0
7.9
7.8
7.7
7.6
7.5
7.4
7.3
7.2
7.1
7.0
6.9
6.8
6.7
6.6
6.5
6.4
6.3
6.2
6.1
6.0
5.9
5.8
5.7
5.6
5.5
5.4
5.3
5.2
5.1
5.0
f2 (ppm)
f1 (ppm)
2.5
3.0
3.5
4.0
4.5
5.0
5.5
6.0
6.5
7.0
7.5

Figure S117. HR-ESI-MS spectrum of compound 12

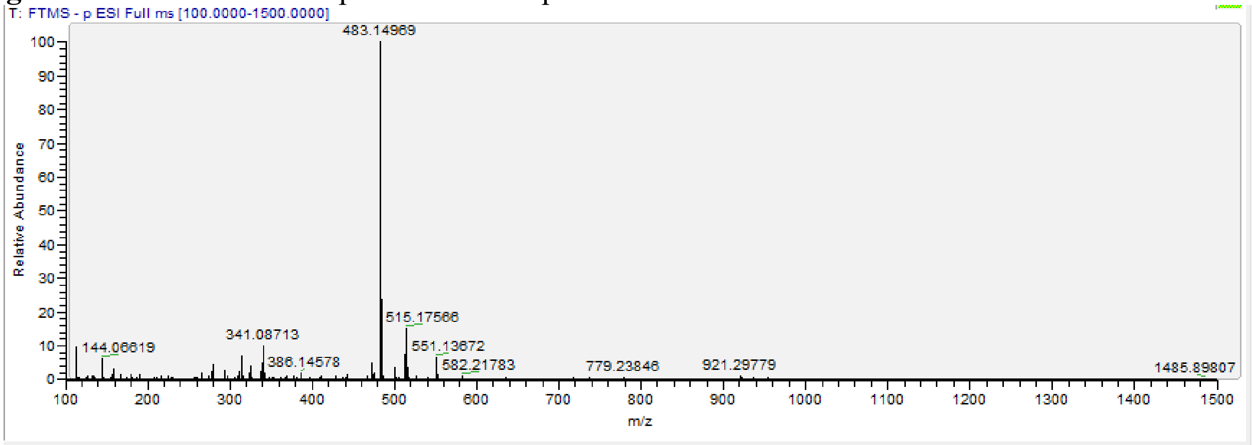

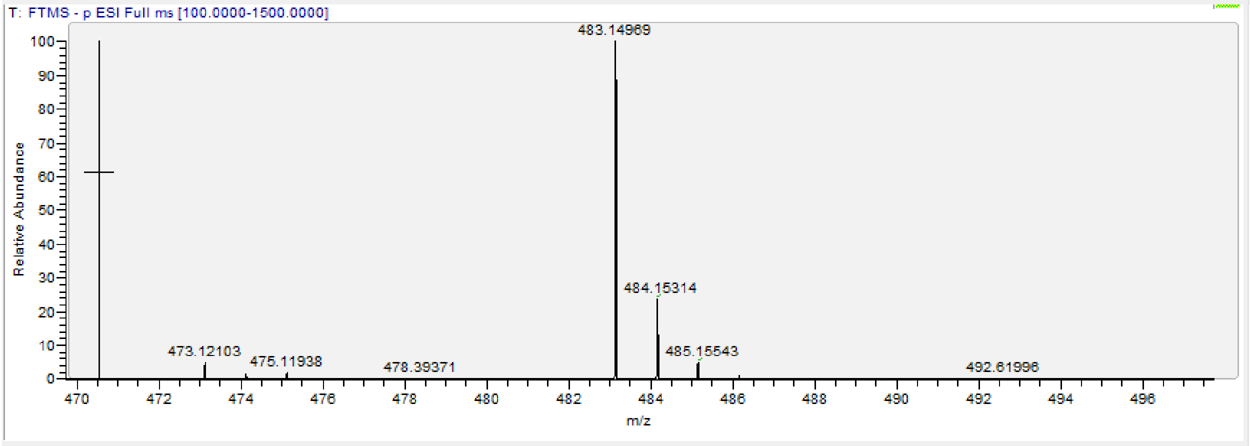

Elemental composition

Single mass

Mass: 483.14954

Max. results 10 Calculate

Idx	Formula	RDB	Delta ppm
1	$C_{22}H_{27}O_{12}$	9.5	-0.337

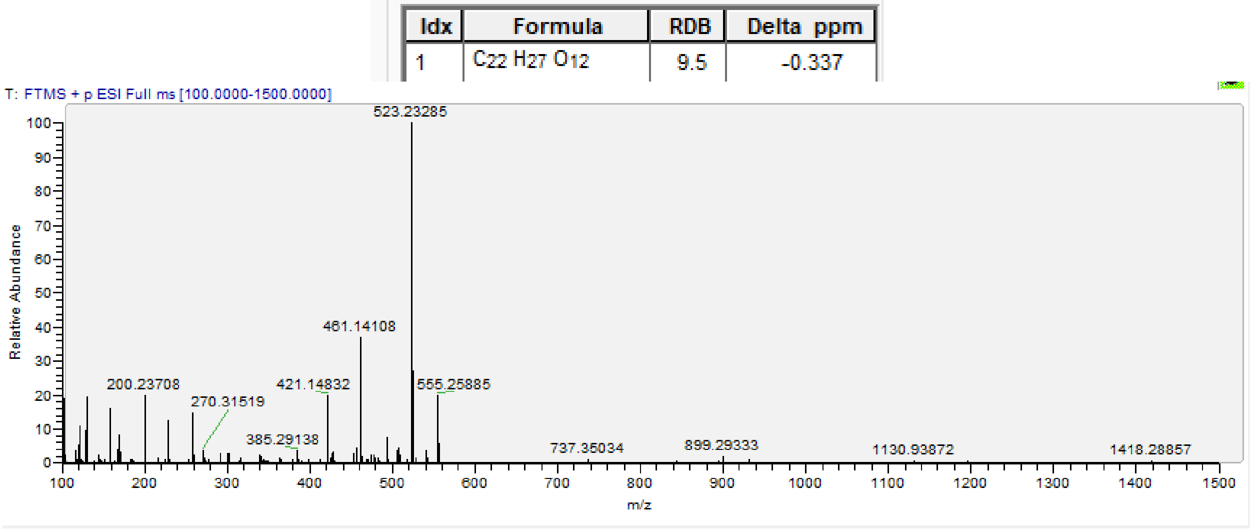

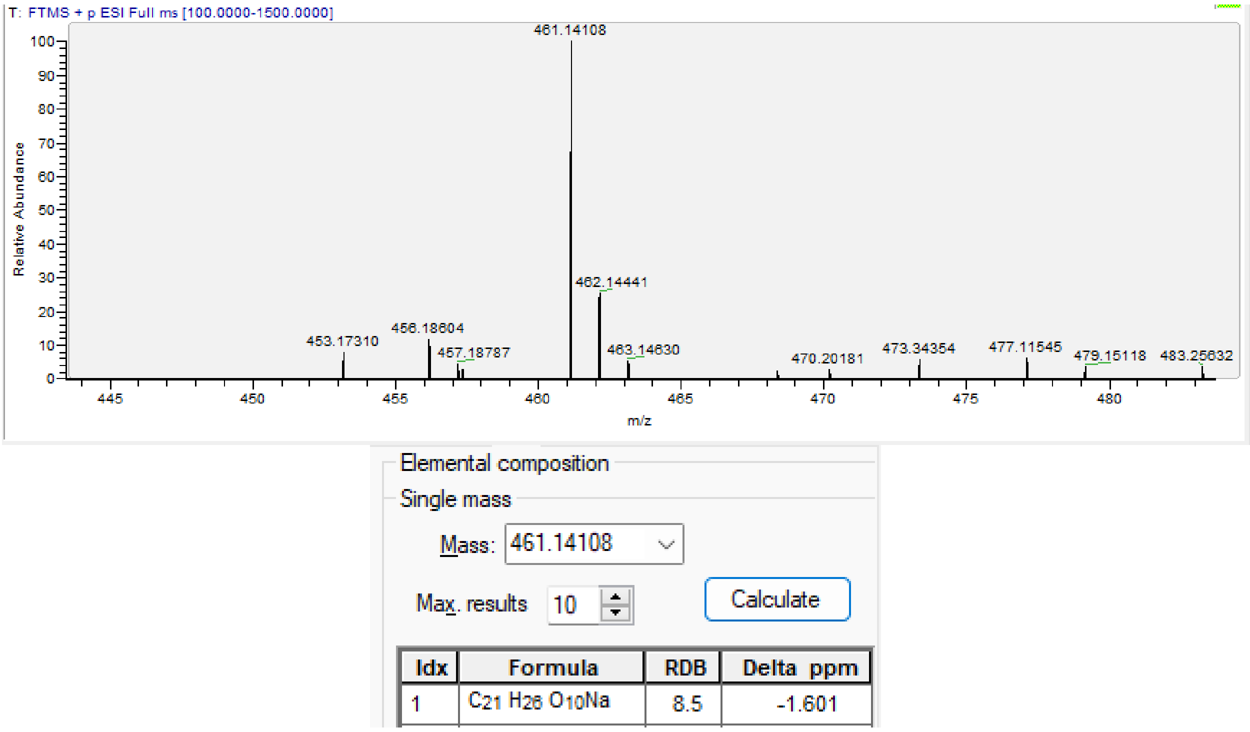

Figure S118. UV spectrum of compound 12

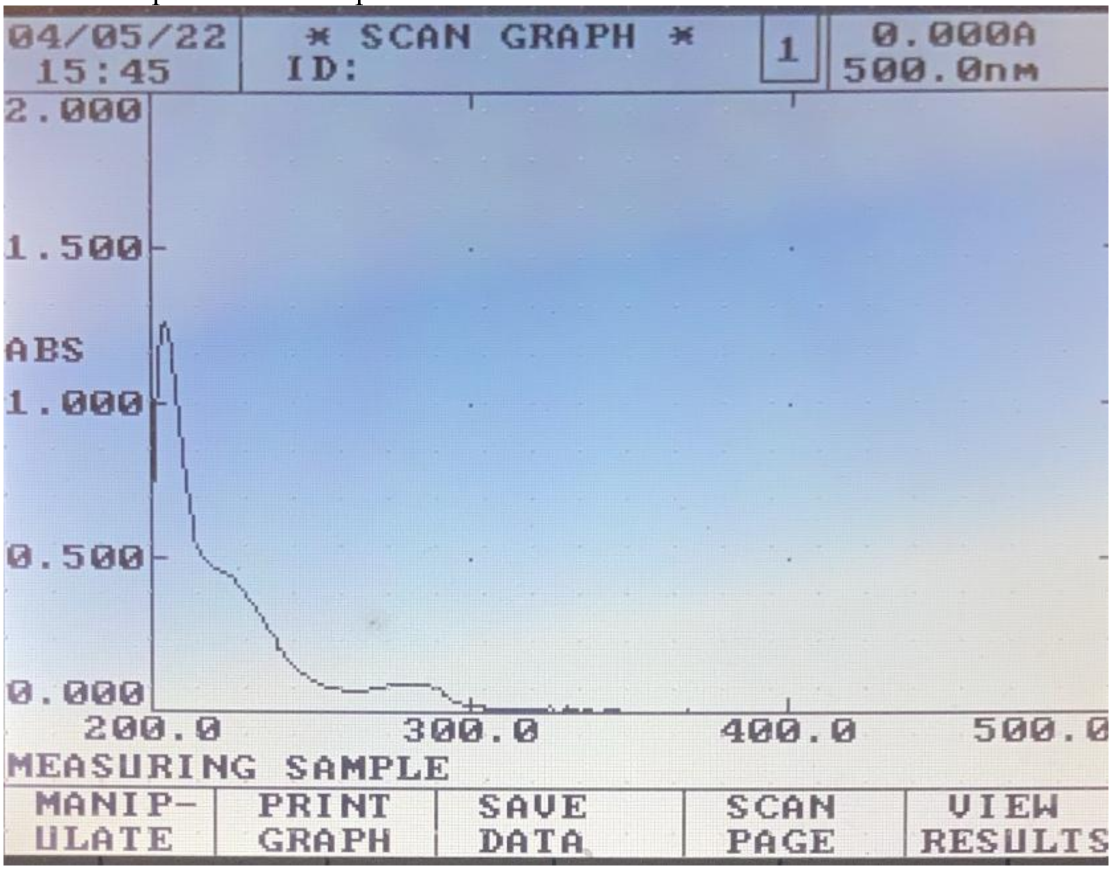

Figure S119. IR spectrum of compound 12

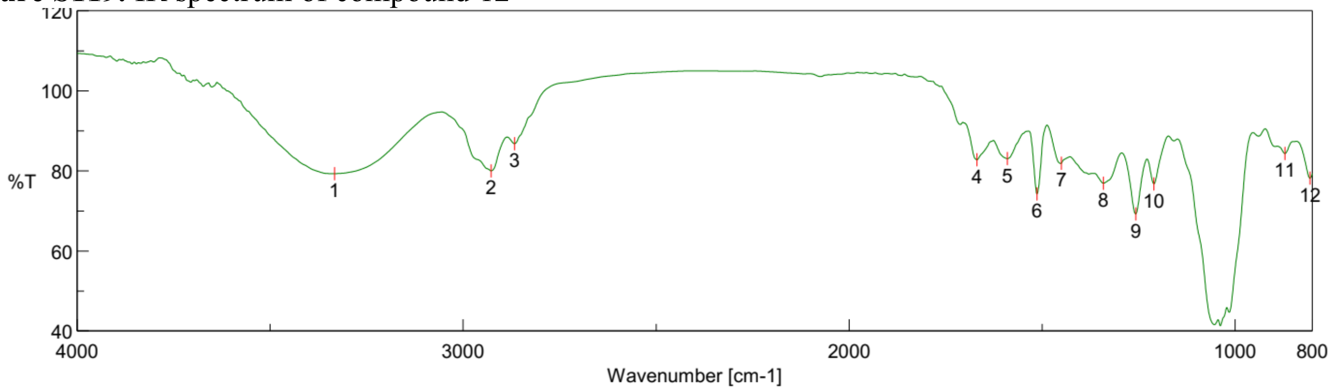

[Result of Peak Picking]

No.	Position	Intensity	No.	Position	Intensity	No.	Position	Intensity
1	3333.36	79.2504	2	2927.41	79.9783	3	2866.67	86.7888
4	1669.09	82.7441	5	1590.02	83.0363	6	1512.88	74.2073
7	1450.21	81.8839	8	1341.25	76.9121	9	1257.36	69.2145
10	1210.11	76.7436	11	870.703	84.3186	12	806.099	78.1557
13	596.861	66.158						

Figure S120. ^{1}H-NMR spectrum of compound 13

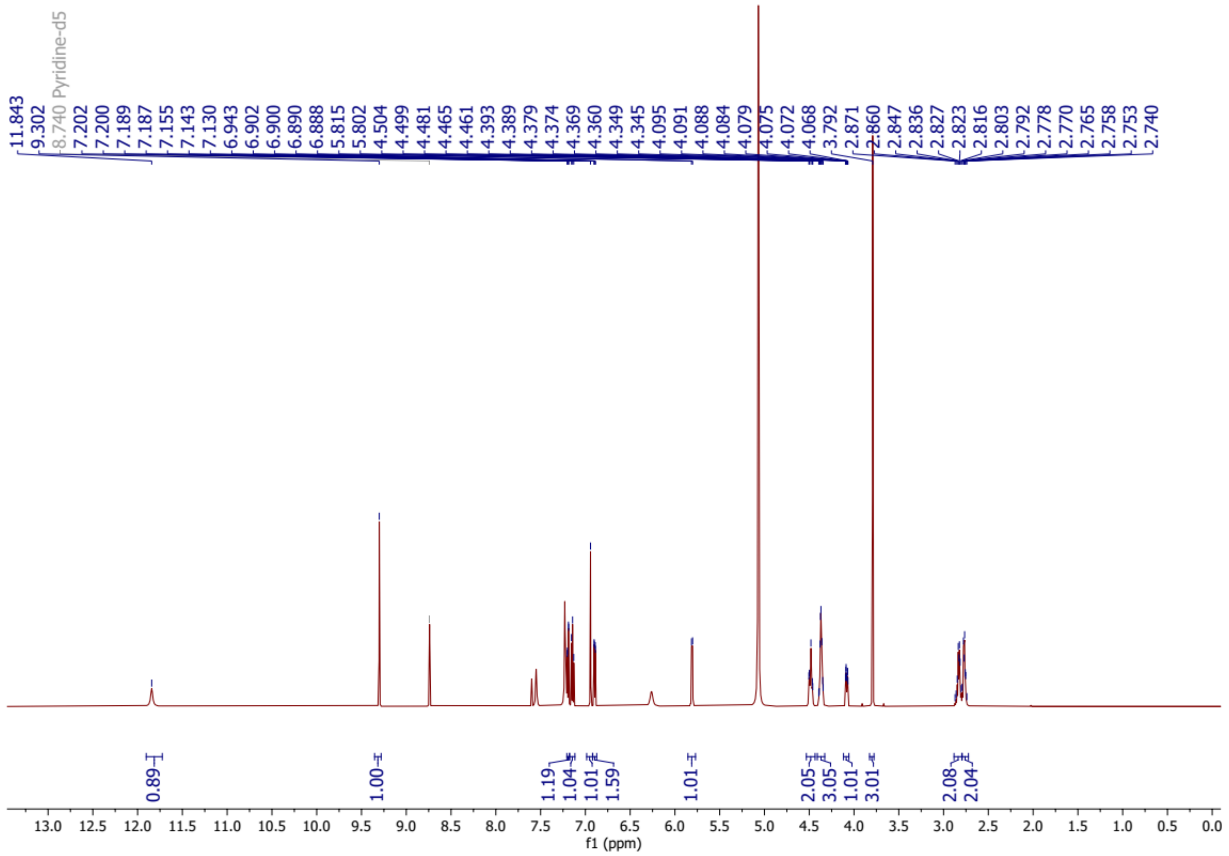

Figure S121. ^{13}C-NMR spectrum of compound 13

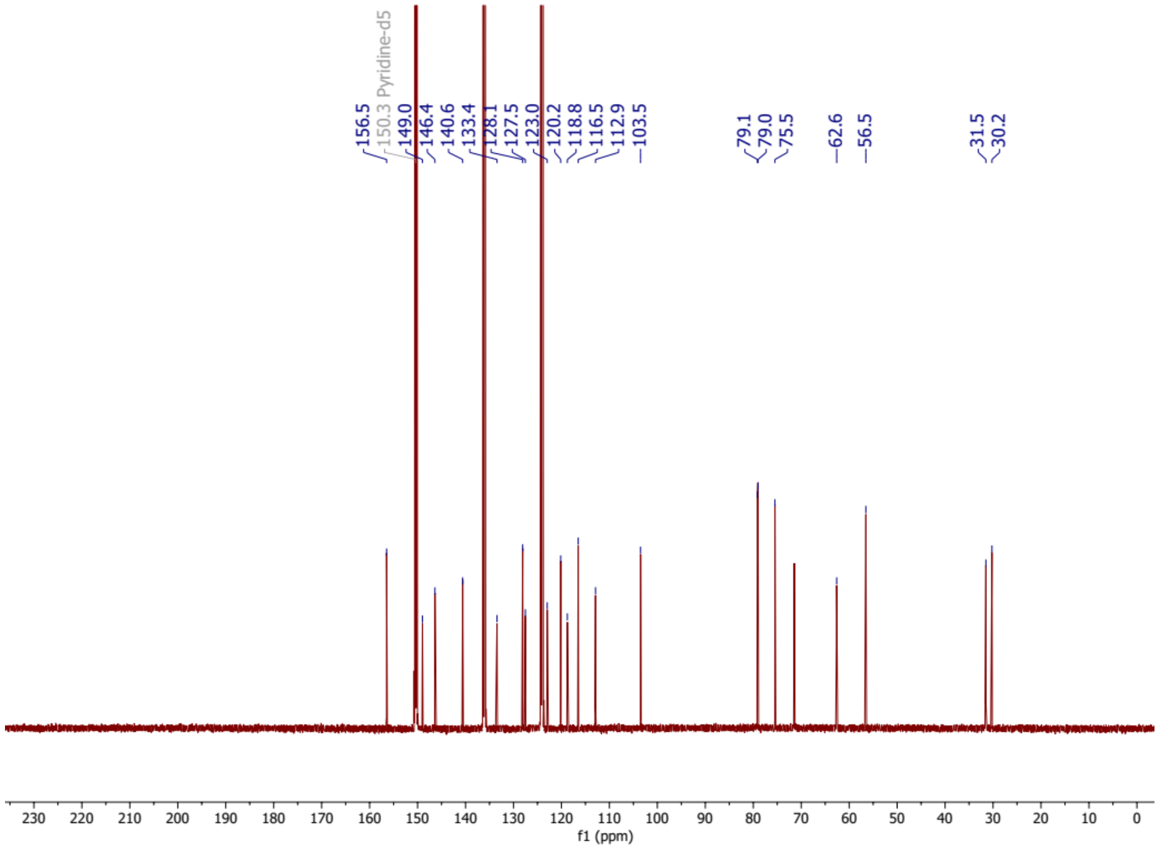

Figure S122. DEPT NMR spectrum of compound 13

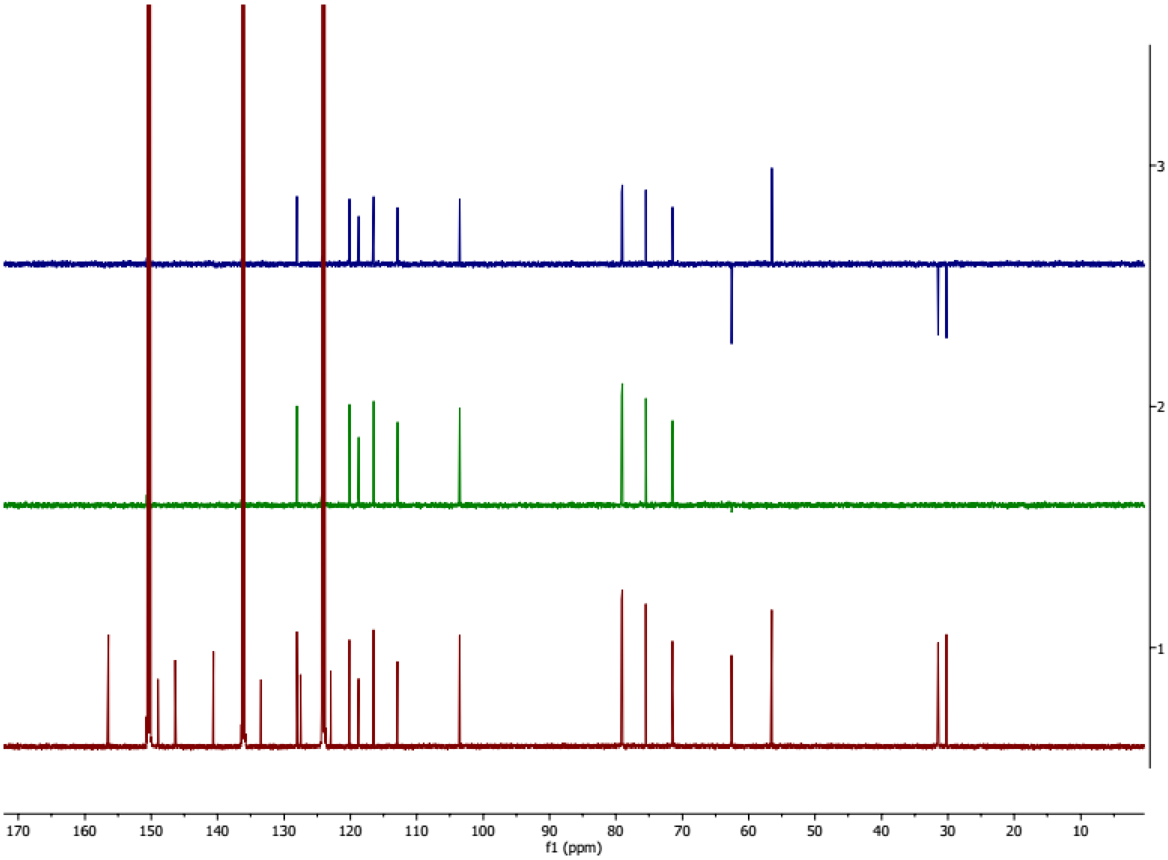

Figure S123. HSQC NMR spectrum of compound 13

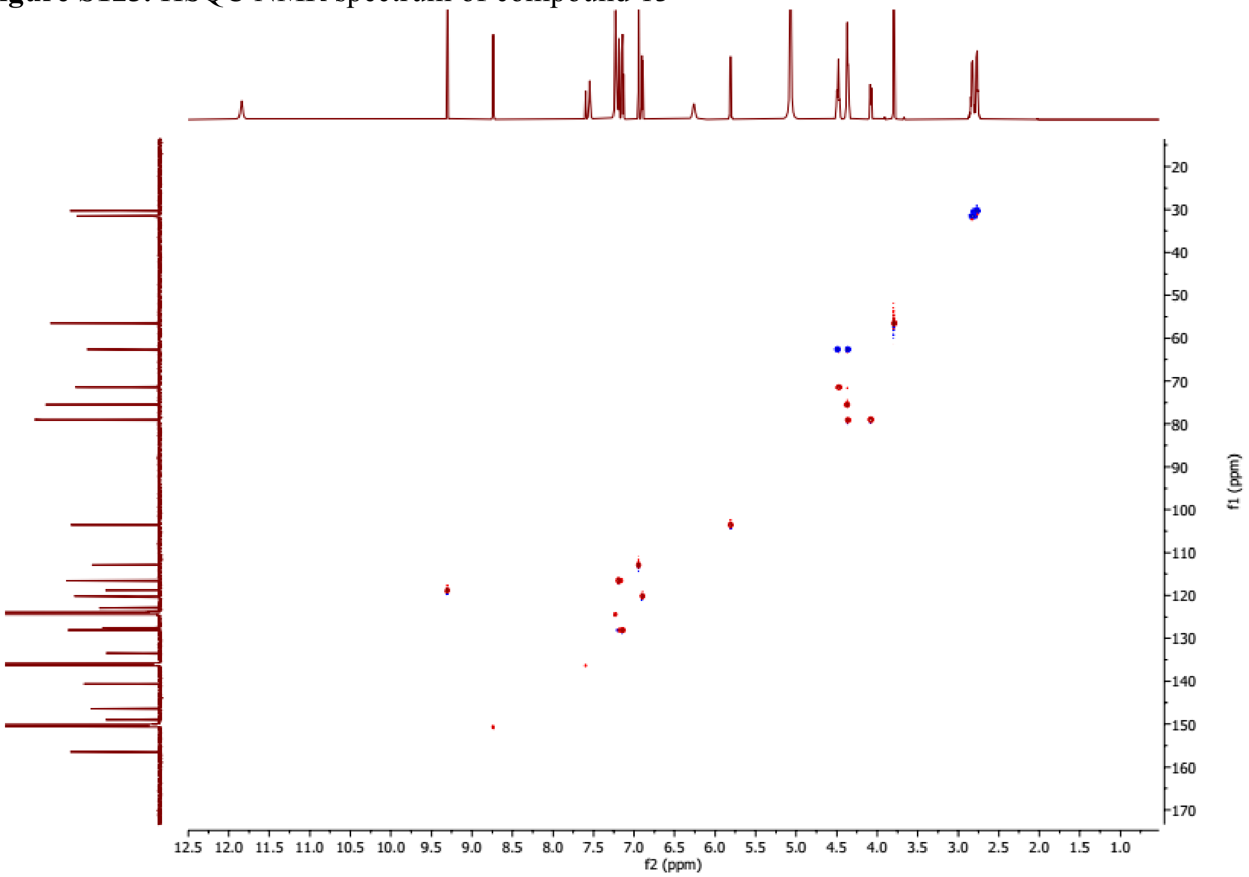

Figure S124. HMBC NMR spectrum of compound 13

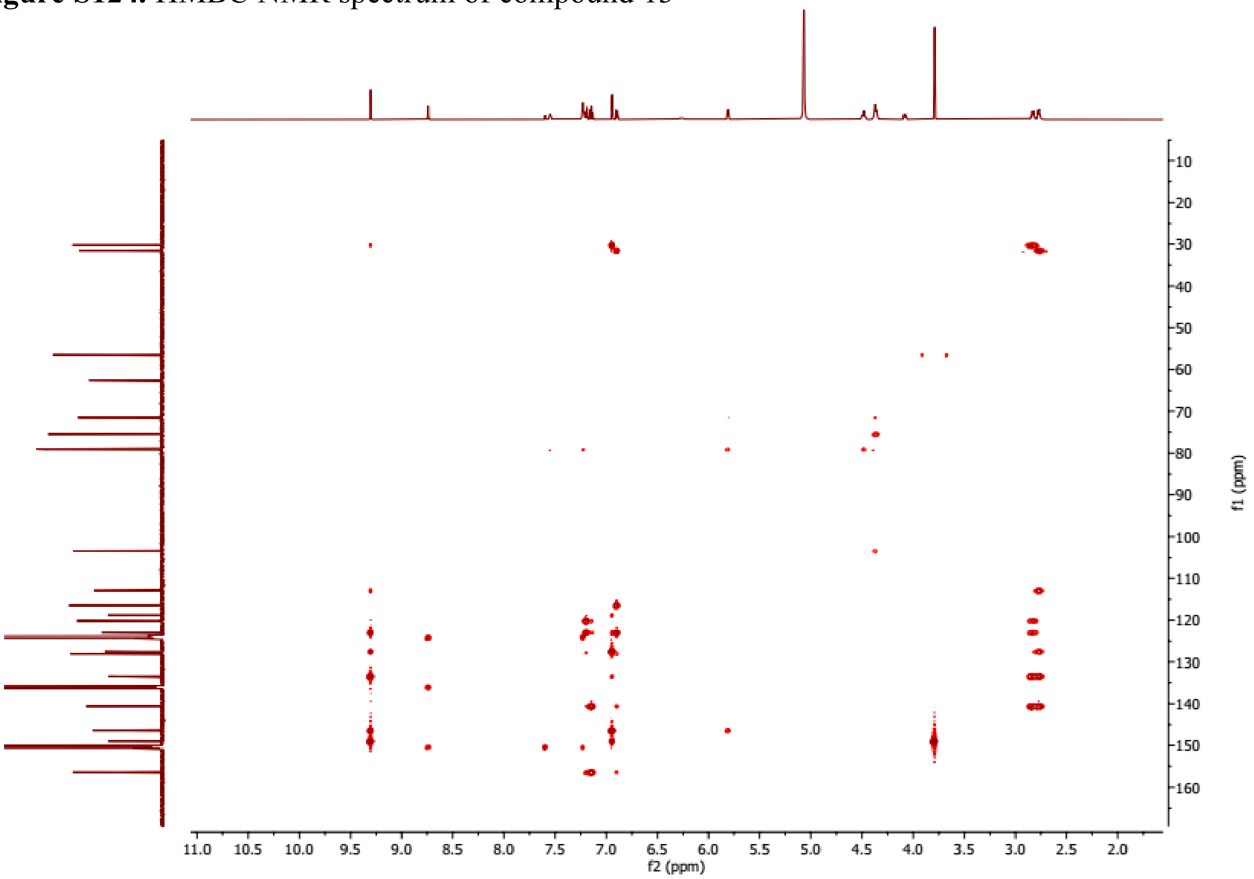

Figure S125. COSY NMR spectrum of compound 13

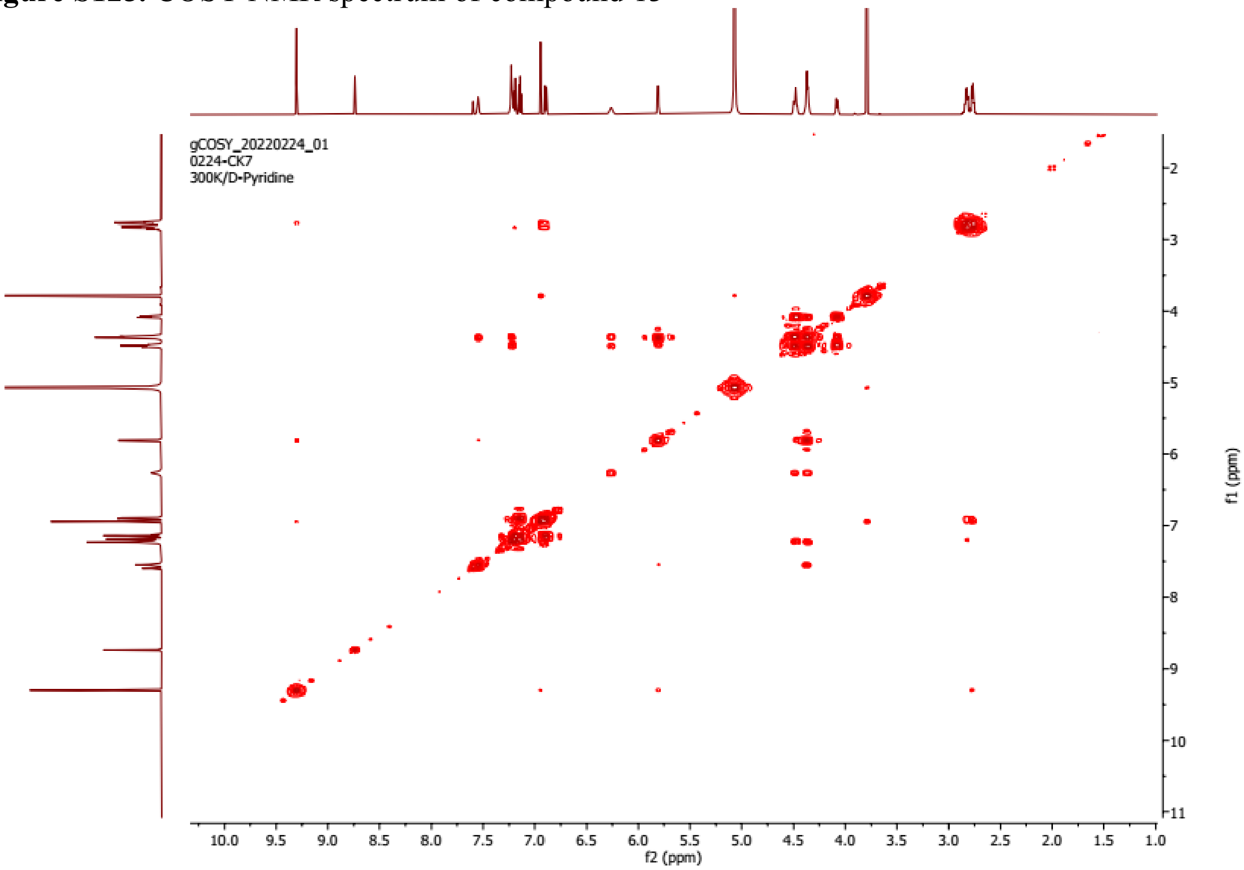

Figure S126. NOESY NMR spectrum of compound 13

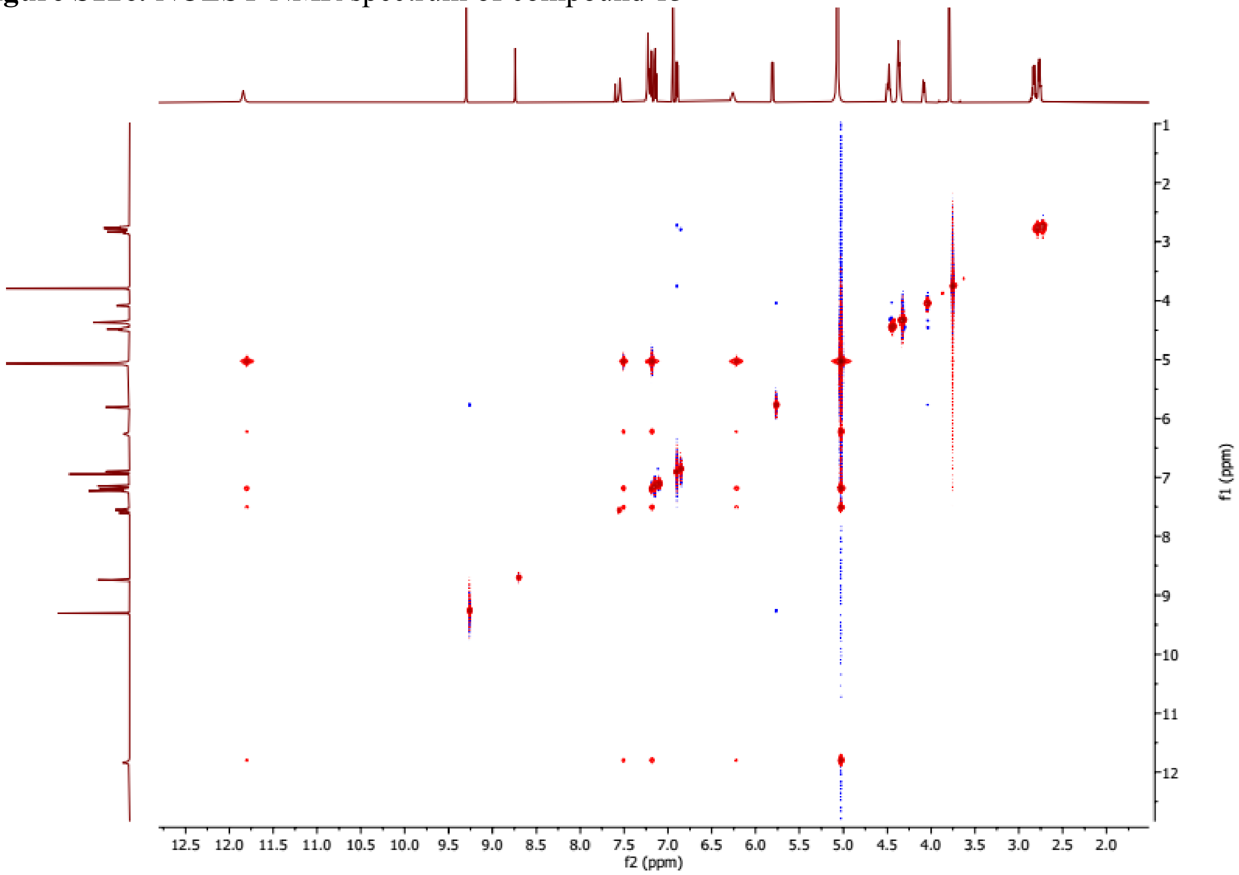

Figure S127. HR-ESI-MS spectrum of compound 13

1) Negative

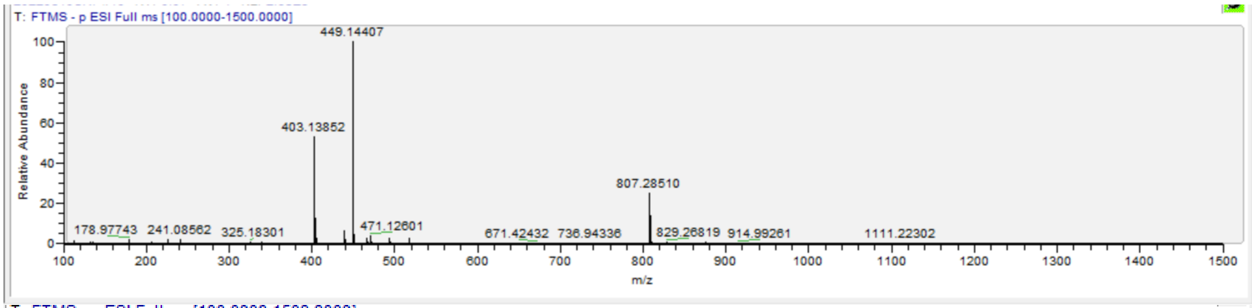

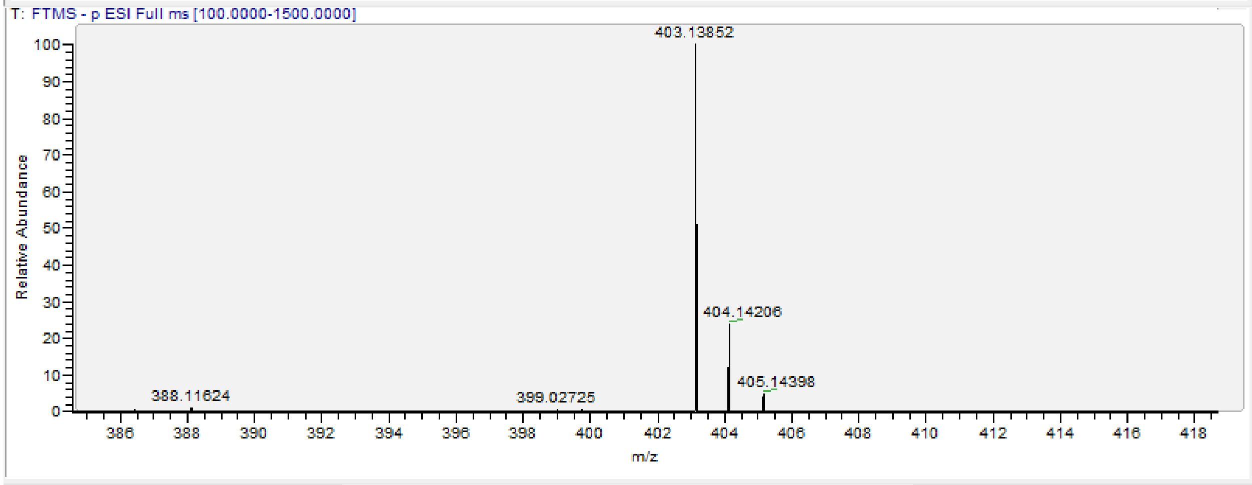

Elemental composition

Single mass

Mass: 403.13852

Max. results 10

Calculate

Idx	Formula	RDB	Delta ppm
1	$C_{21}H_{23}O_8$	10.5	-0.556

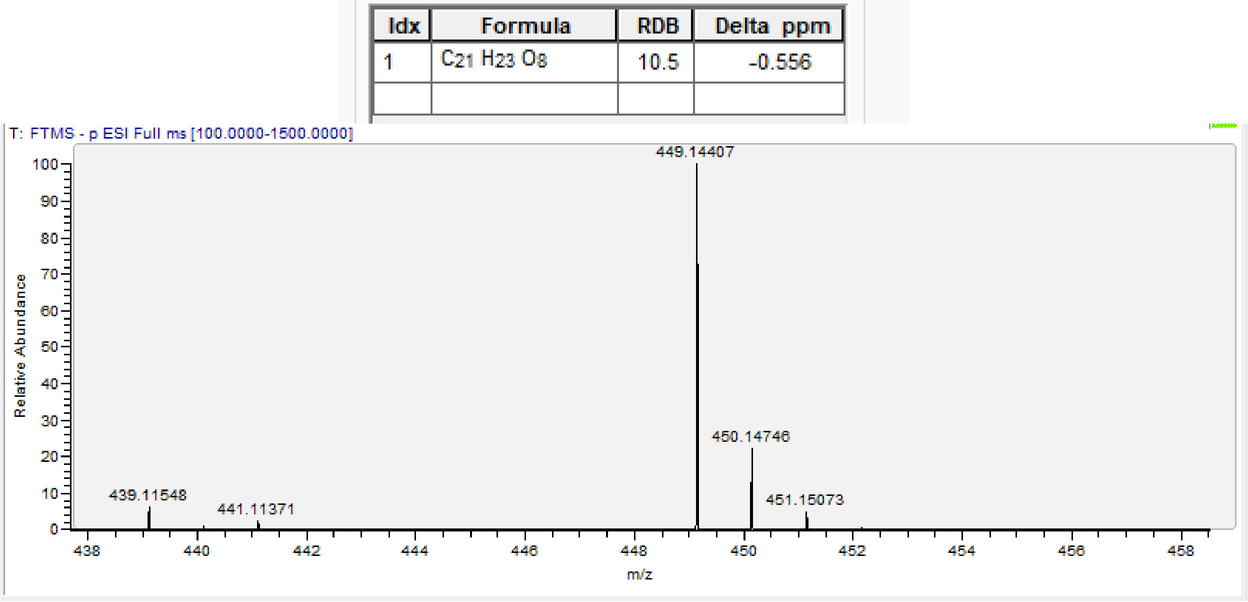

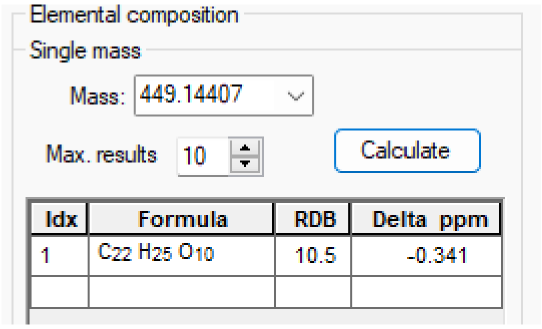
Elemental composition
Single mass
Mass: 449.14407
Max. results 10
Calculate
Idx | Formula | RDB | Delta ppm
1 | C22 H25 O10 | 10.5 | -0.341

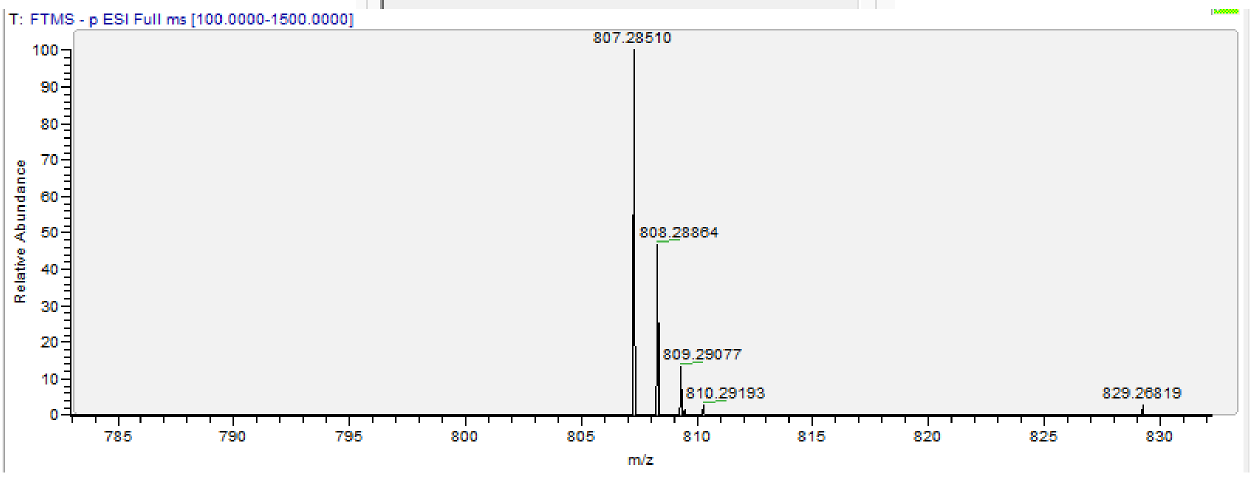
T: FTMS - p ESI Full ms [100.0000-1500.0000]
807.28510
808.28864
809.29077
810.29193
829.26819
Relative Abundance
m/z

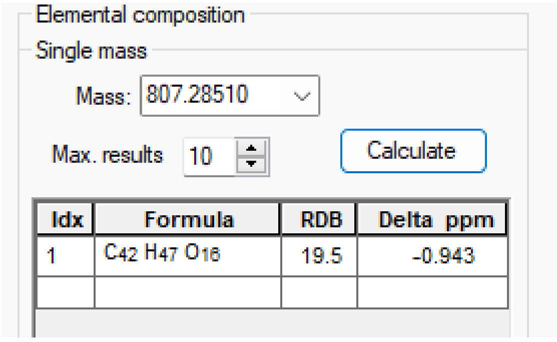
Elemental composition
Single mass
Mass: 807.28510
Max. results 10
Calculate
Idx | Formula | RDB | Delta ppm
1 | C42 H47 O16 | 19.5 | -0.943

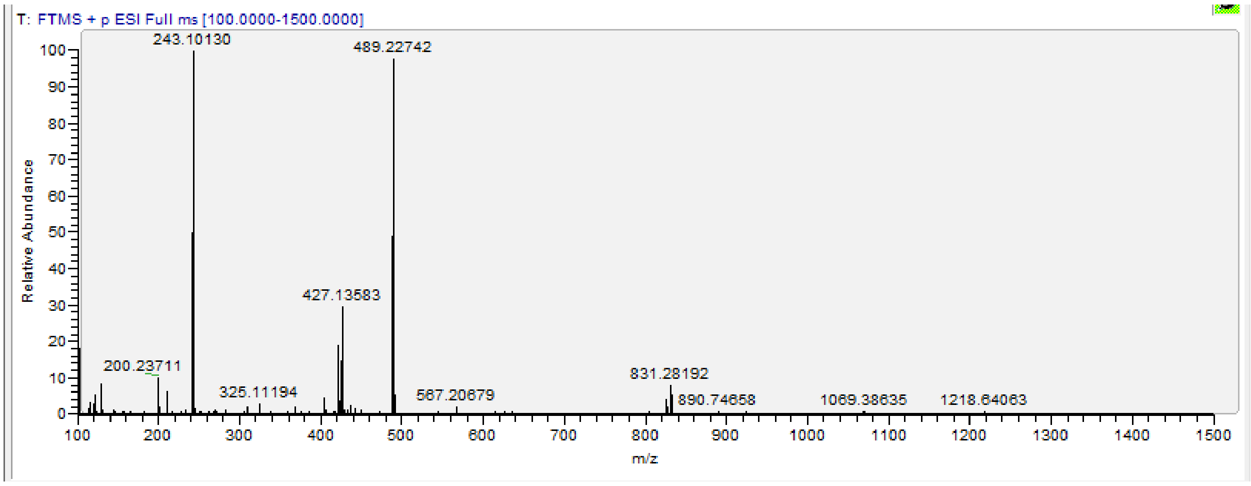
T: FTMS + p ESI Full ms [100.0000-1500.0000]
243.10130
489.22742
427.13583
200.23711
325.11194
567.20679
831.28192
890.74658
1069.38635
1218.64063
Relative Abundance
m/z

Elemental composition

Single mass

Mass: 427.13583

Max. results 10

Calculate

Idx	Formula	RDB	Delta ppm
1	$C_{21}H_{24}O_8Na$	9.5	-1.191

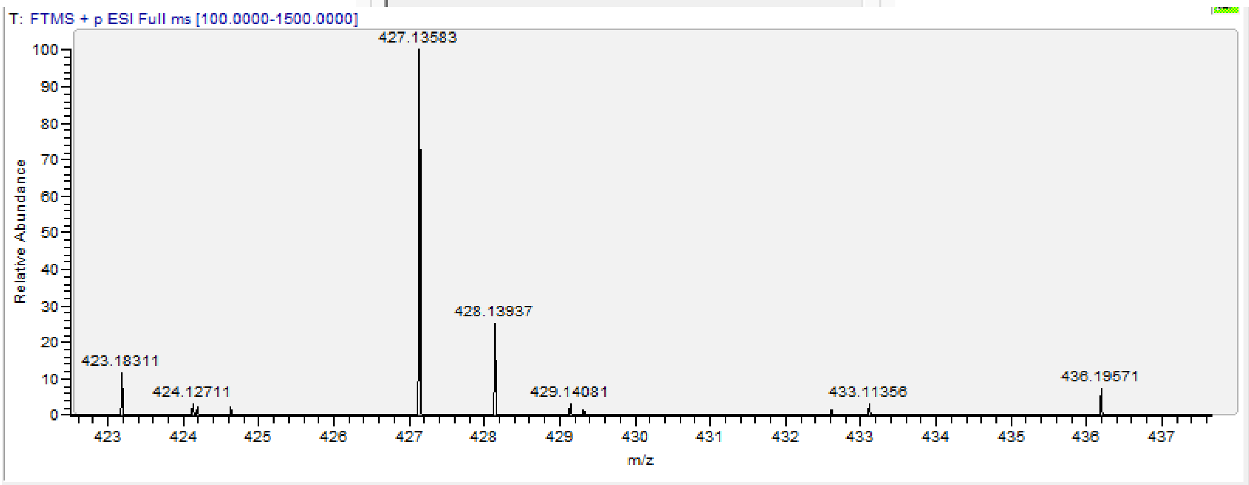

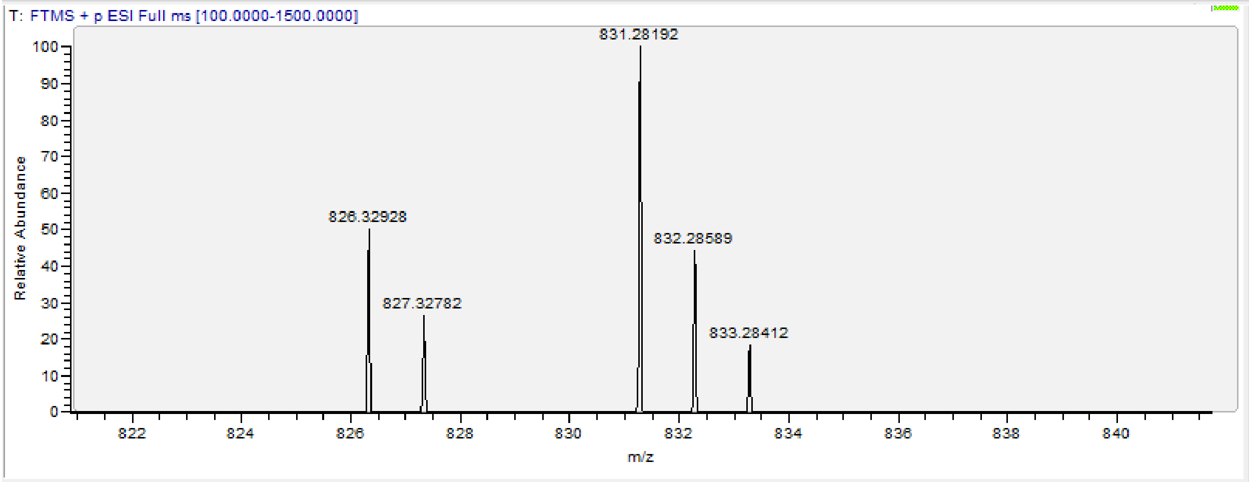

Elemental composition

Single mass

Mass: 831.28192

Max. results 10

Calculate

Idx	Formula	RDB	Delta ppm
1	$C_{42}H_{48}O_{16}Na$	18.5	-1.848

Figure S128. UV spectrum of compound 13

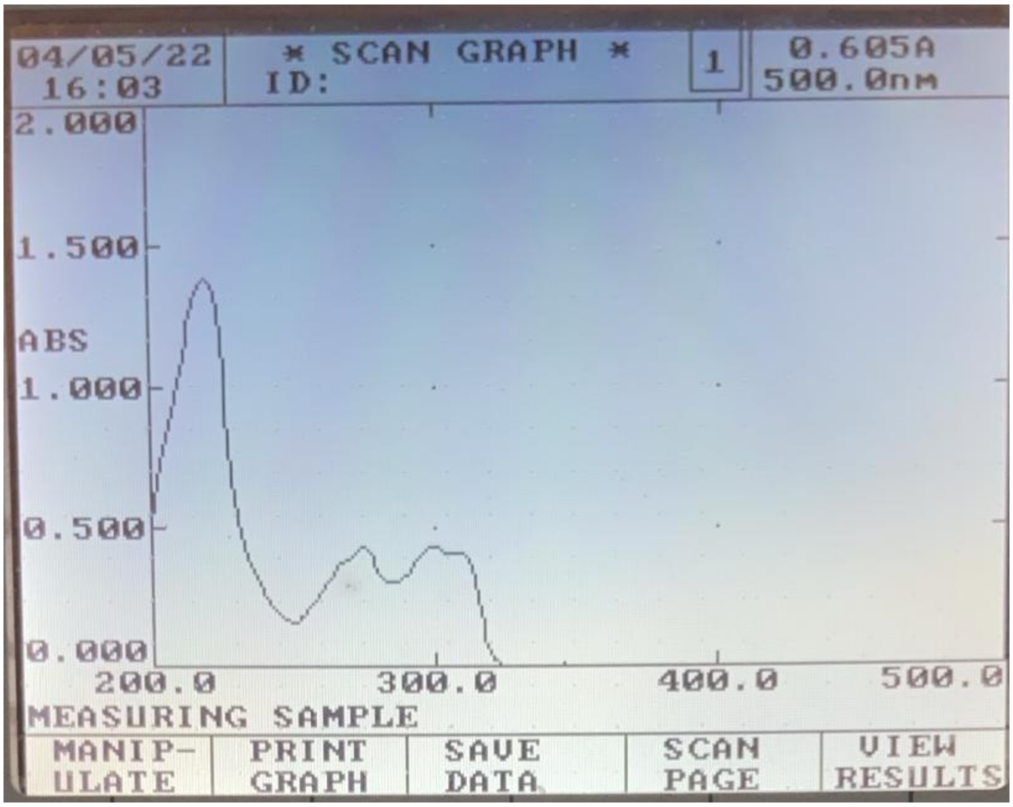

Figure S129. IR spectrum of compound 13

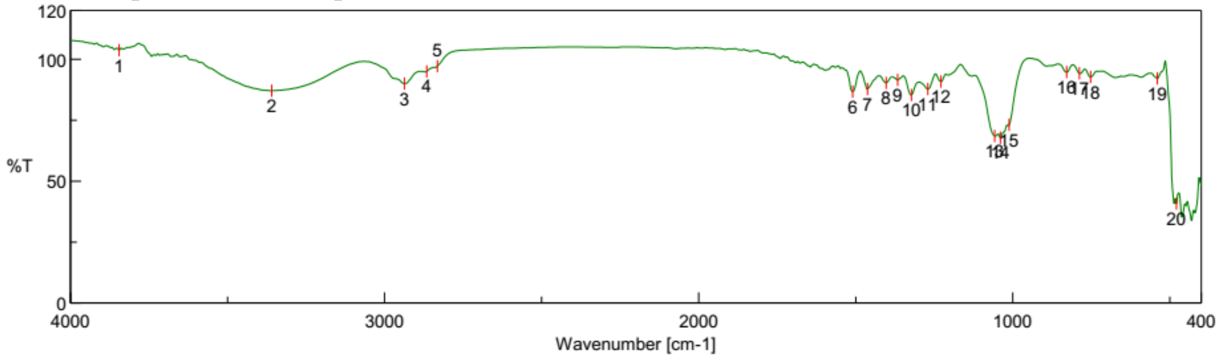

[Result of Peak Picking]

No.	Position	Intensity	No.	Position	Intensity	No.	Position	Intensity
1	3845.36	103.721	2	3359.39	87.0457	3	2937.06	90.0537
4	2865.7	94.9553	5	2831.95	97.2086	6	1509.99	86.7484
7	1462.74	87.7539	8	1402.96	90.294	9	1366.32	91.4898
10	1322.93	85.278	11	1270.86	87.7599	12	1228.43	90.842
13	1057.76	68.5196	14	1039.44	67.8312	15	1011.48	73.0801
16	828.277	94.6972	17	787.779	94.162	18	752.102	92.7154
19	539.971	92.078	20	479.224	40.7483			

Figure S130. ^{1}H-NMR spectrum of compound 14

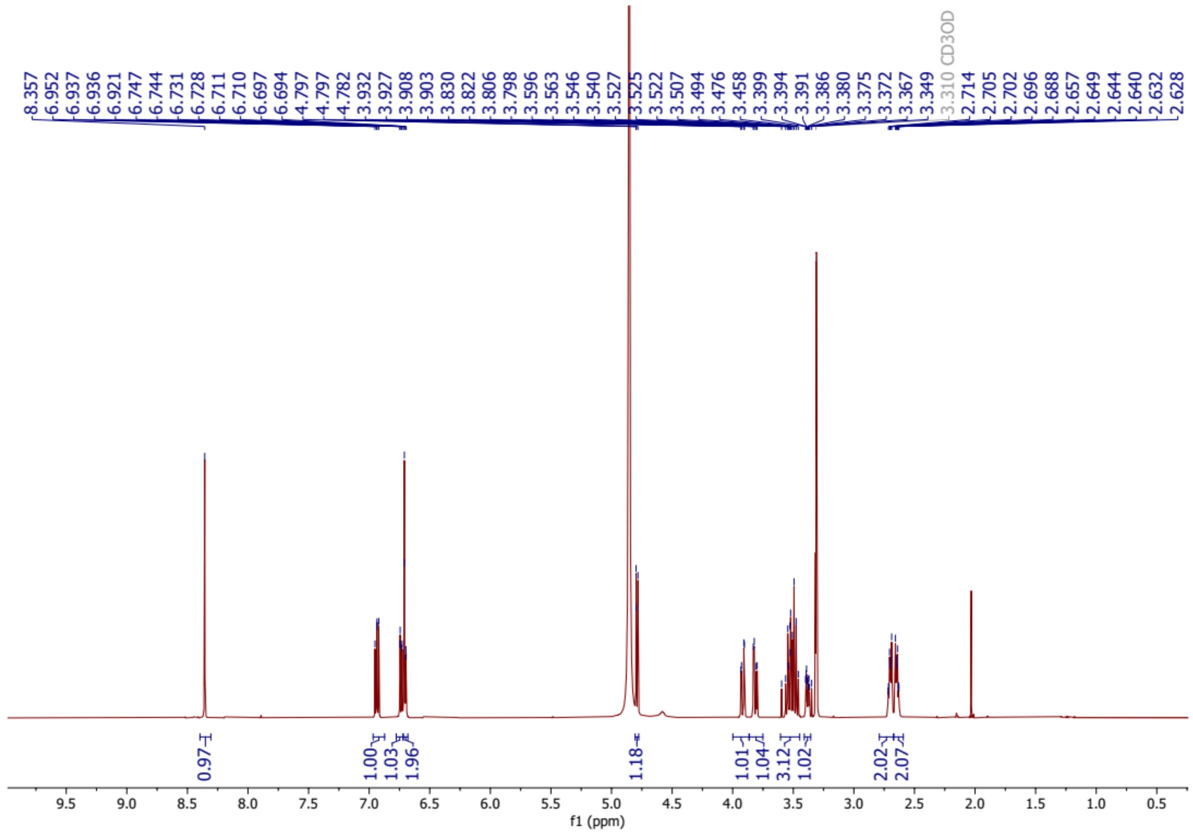

Figure S131. ^{13}C-NMR spectrum of compound 14

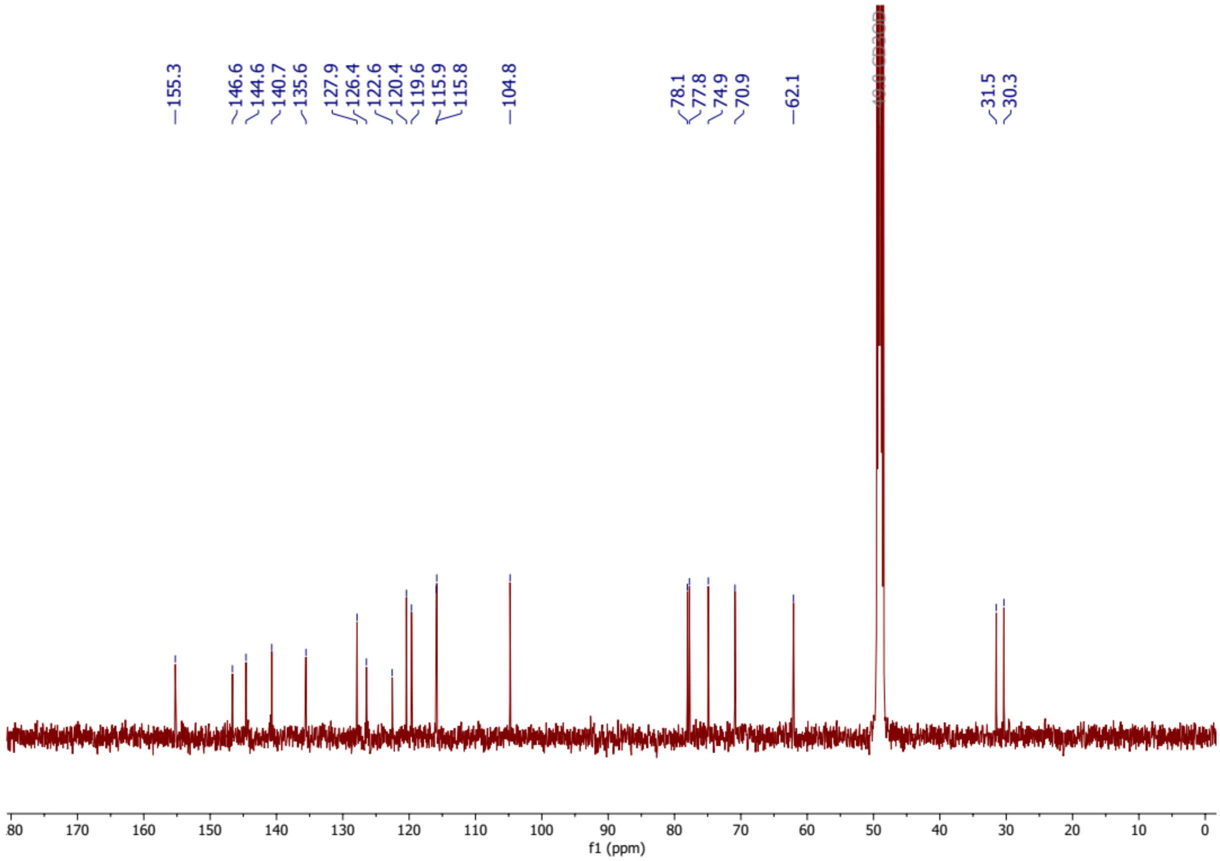

Figure S132. HSQC NMR spectrum of compound 14

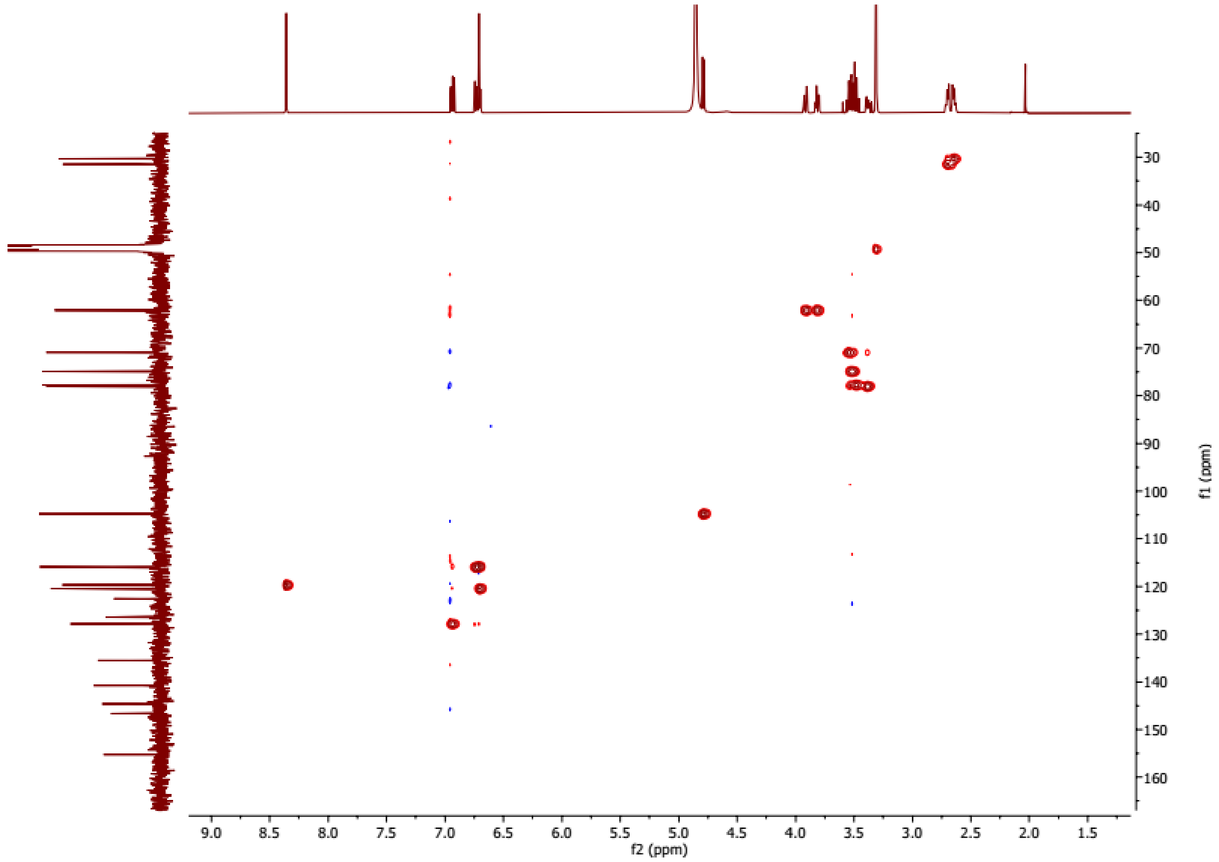

Figure S133. HMBC NMR spectrum of compound 14

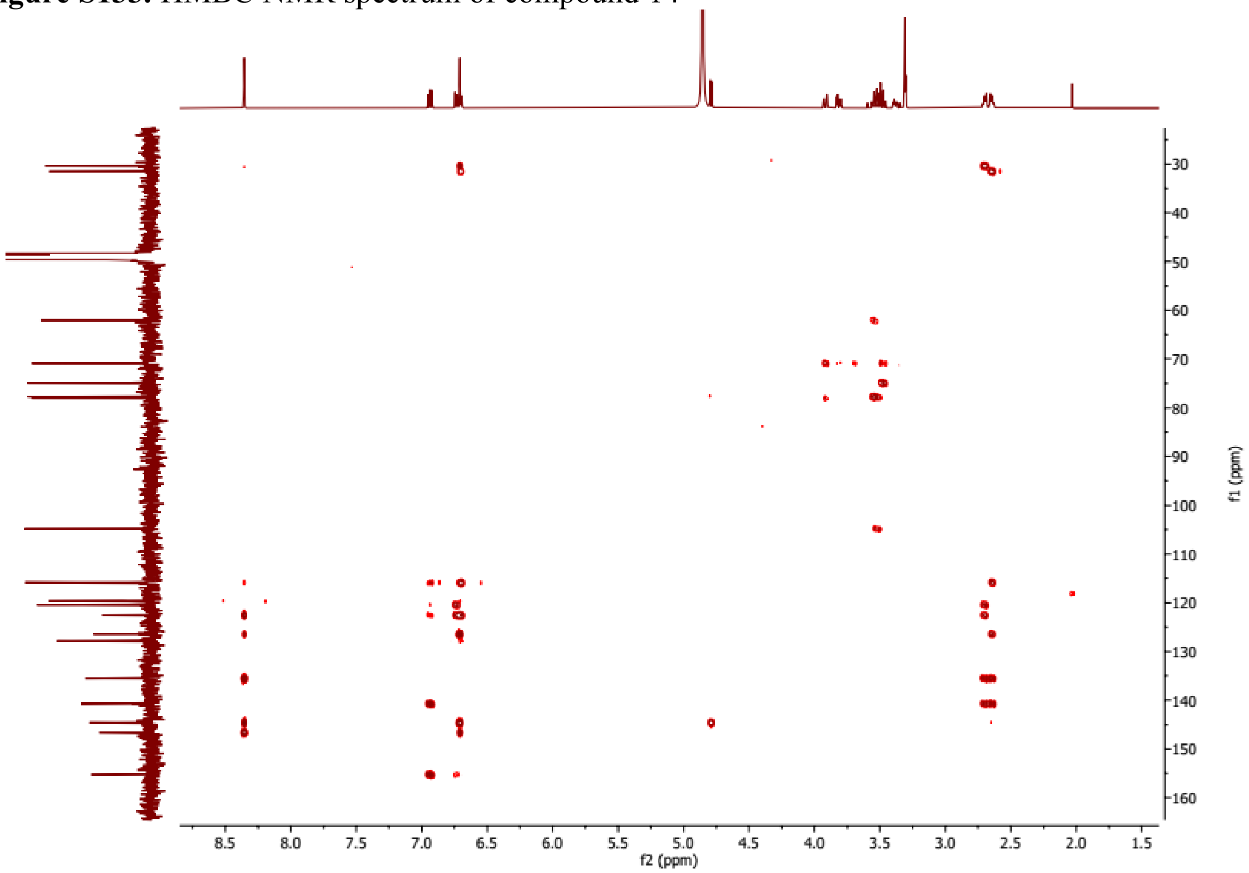

Figure S134. COSY NMR spectrum of compound 14

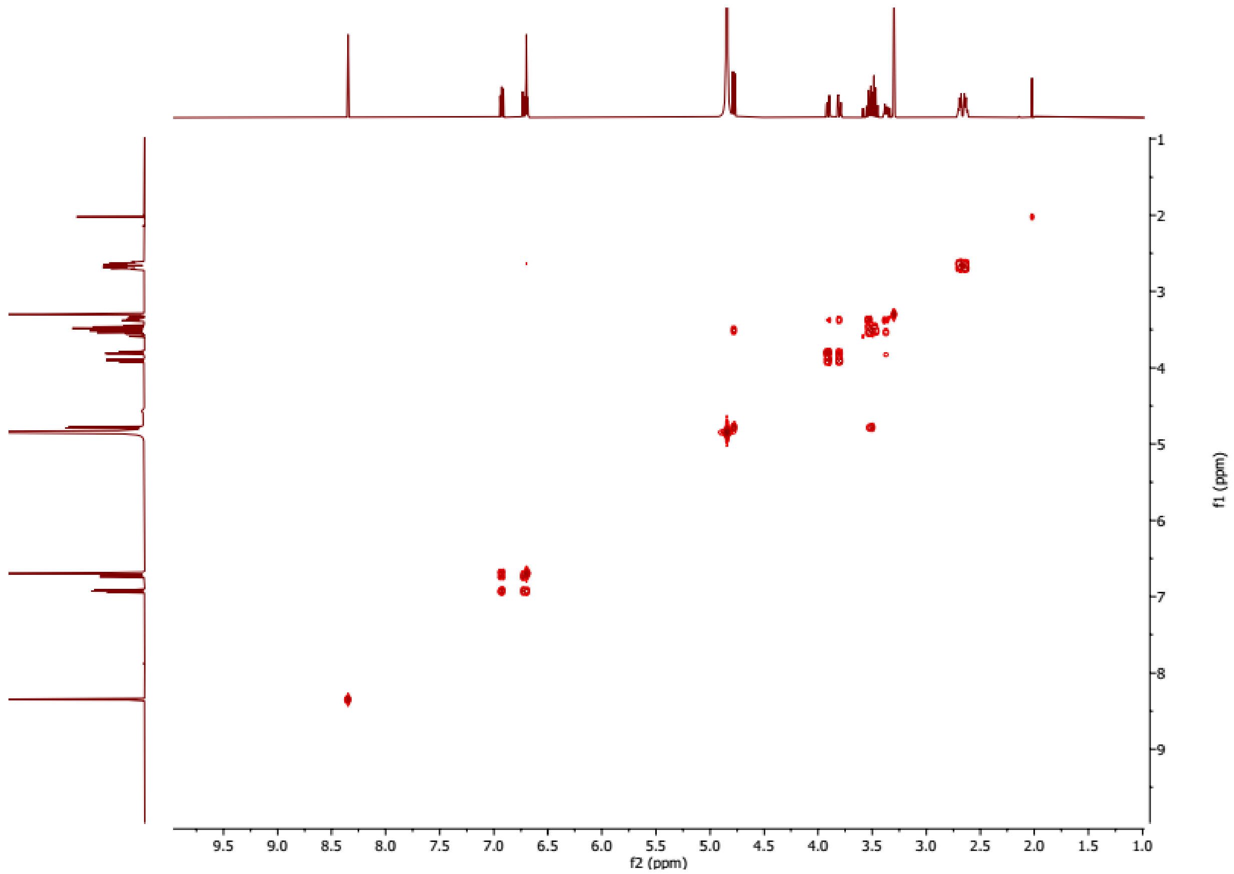

Figure S135. NOESY NMR spectrum of compound 14

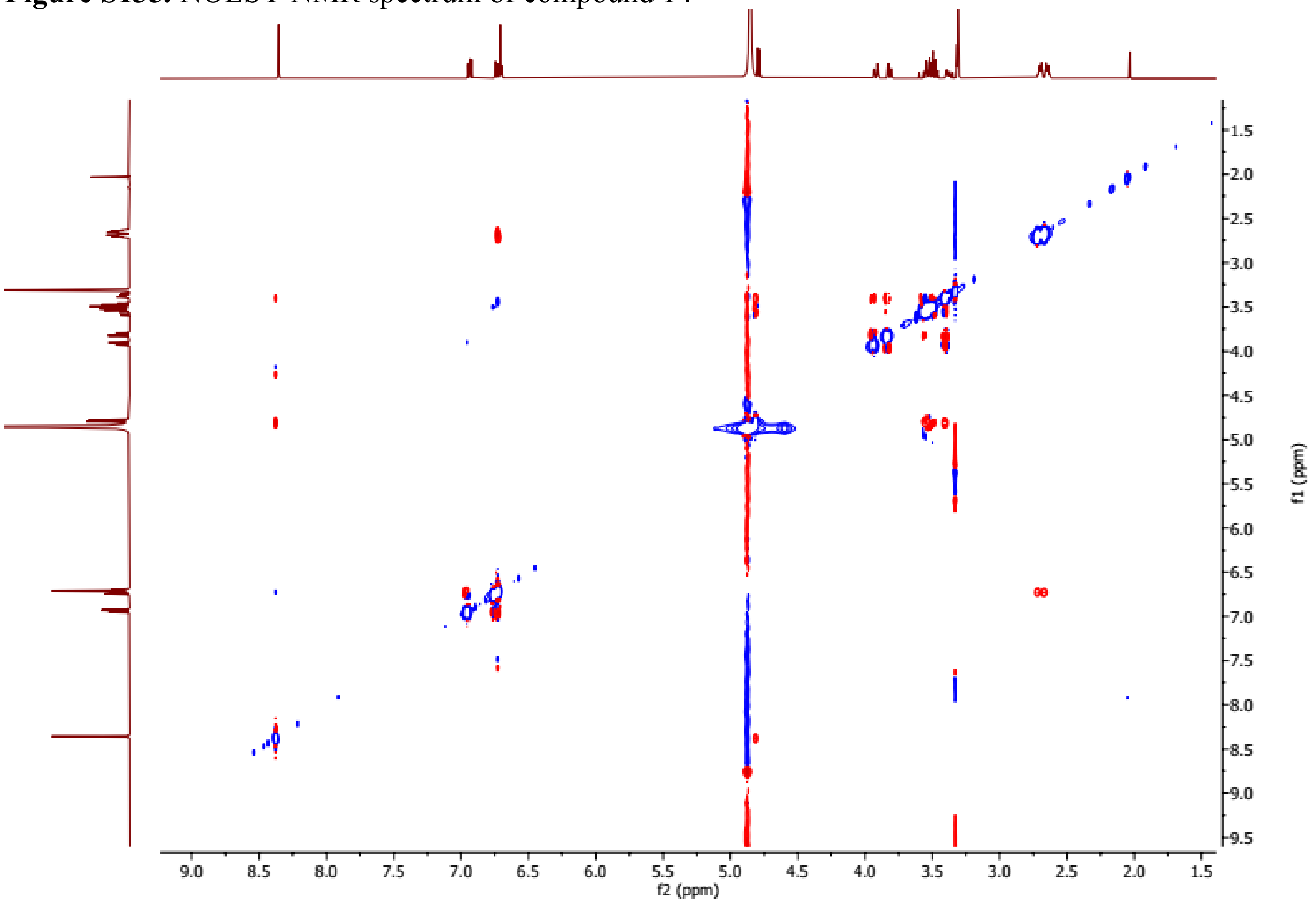

Figure S136. HR-ESI-MS spectrum of compound 14

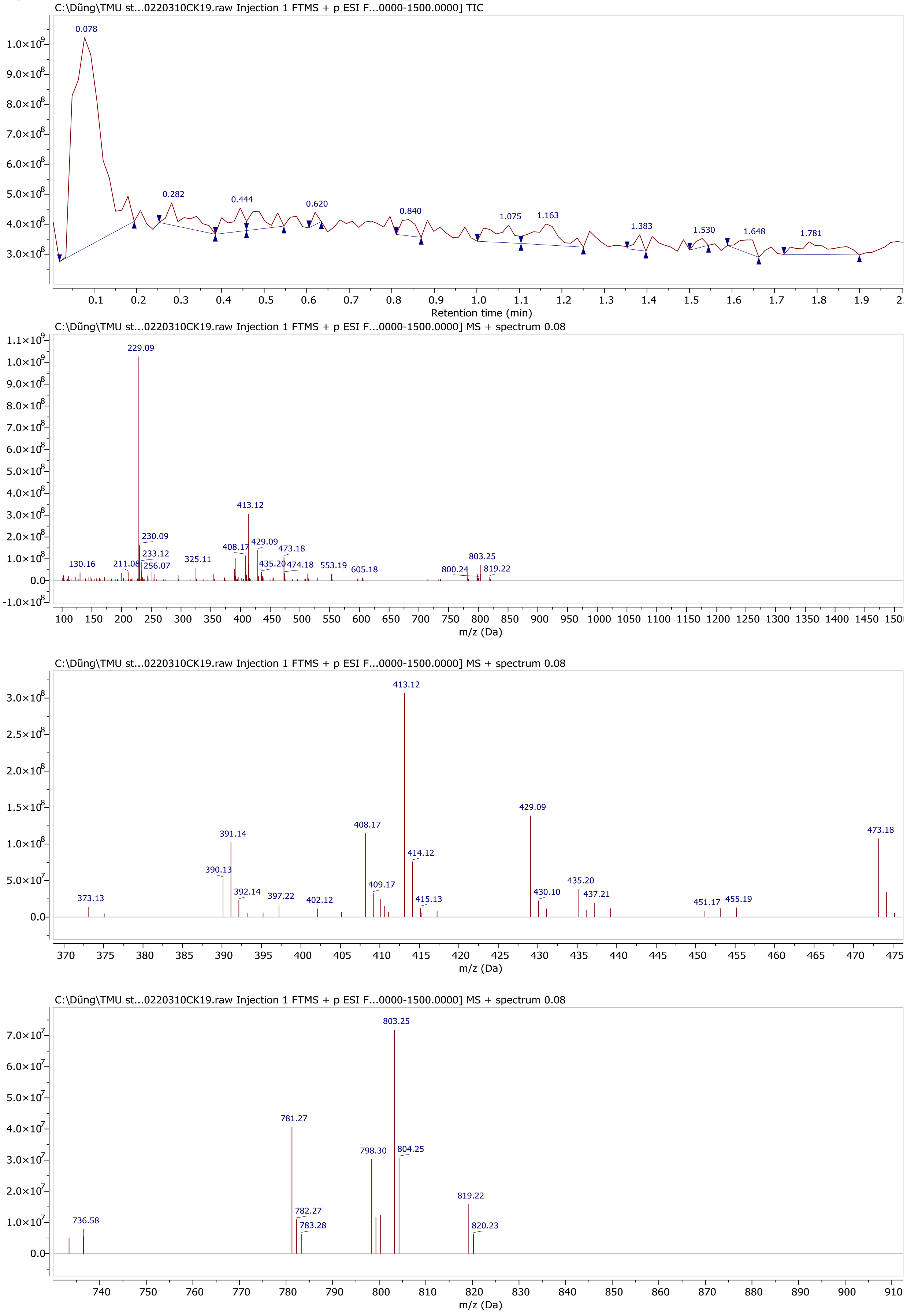

	Formula	Calculated Mass	Target Mass	Double Bond Equivalence	Absolute Error (ppm)	Error (mDa)	Error (ppm)	Fitness
1	C20 H23 O8	391.13874	391.13830	10.0	1.14	-0.44	-1.14	0.997

	Formula	Calculated Mass	Target Mass	Double Bond Equivalence	Absolute Error (ppm)	Error (mDa)	Error (ppm)	Fitness
1	C20 H22 O8 Na	413.12069	413.12048	10.0	0.50	-0.21	-0.50	0.999

	Formula	Calculated Mass	Target Mass	Double Bond Equivalence	Absolute Error (ppm)	Error (mDa)	Error (ppm)	Fitness
1	C40 H44 O16 Na	803.25216	803.25250	19.0	0.43	0.35	0.43	1.000

	Formula	Calculated Mass	Target Mass	Double Bond Equivalence	Absolute Error (ppm)	Error (mDa)	Error (ppm)	Fitness
1	C40 H45 O16	781.27021	781.27065	19.0	0.56	0.44	0.56	0.952

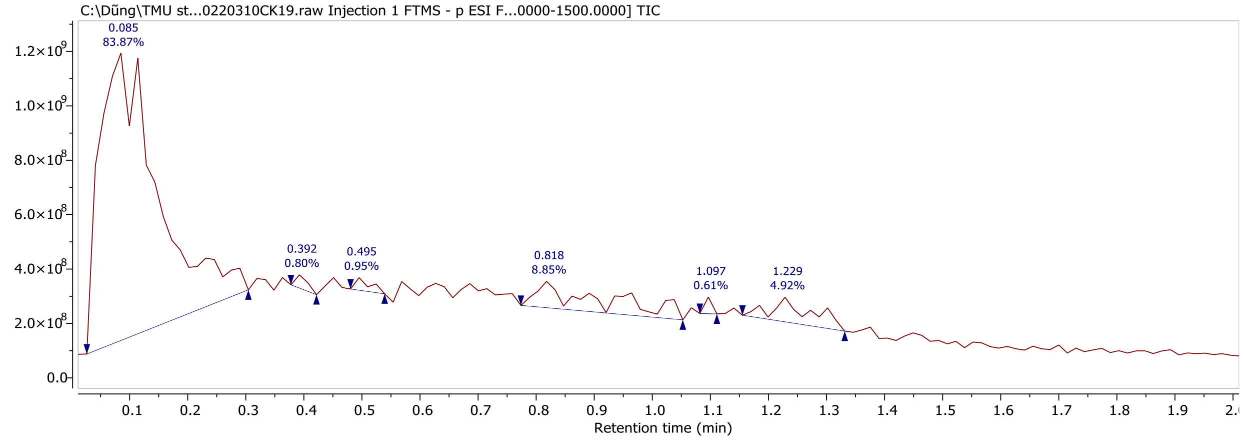
C:\Dũng\TMU st...0220310CK19.raw Injection 1 FTMS - p ESI F...0000-1500.0000] TIC
0.085
83.87%
0.392
0.80%
0.495
0.95%
0.818
8.85%
1.097
0.61%
1.229
4.92%
Retention time (min)

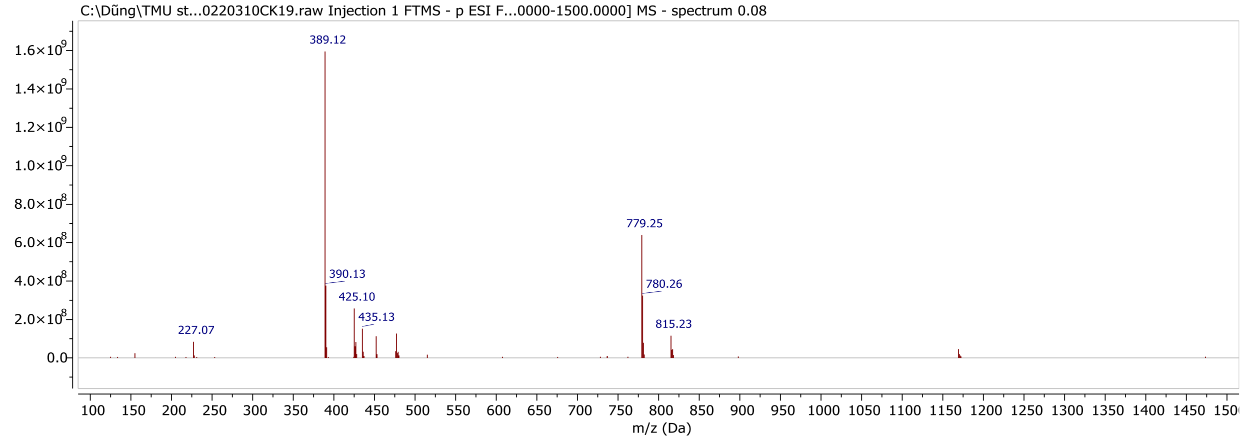
C:\Dũng\TMU st...0220310CK19.raw Injection 1 FTMS - p ESI F...0000-1500.0000] MS - spectrum 0.08
389.12
390.13
425.10
435.13
227.07
779.25
780.26
815.23
m/z (Da)

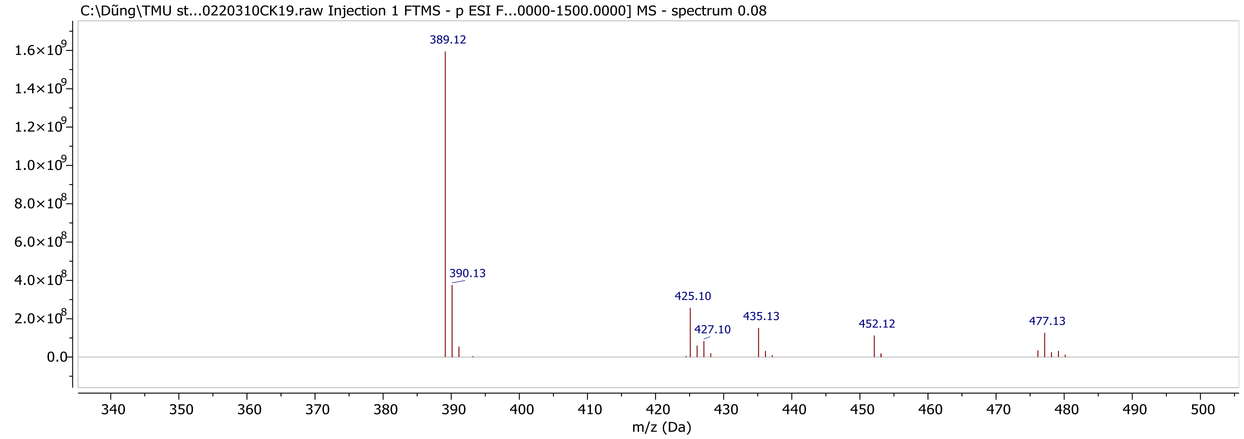
C:\Dũng\TMU st...0220310CK19.raw Injection 1 FTMS - p ESI F...0000-1500.0000] MS - spectrum 0.08
389.12
390.13
425.10
427.10
435.13
452.12
477.13
m/z (Da)

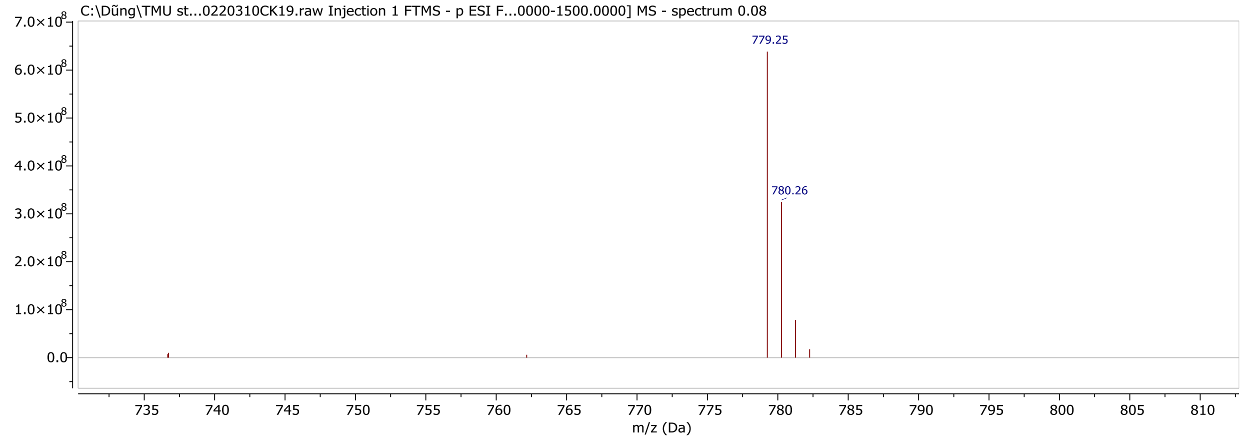
C:\Dũng\TMU st...0220310CK19.raw Injection 1 FTMS - p ESI F...0000-1500.0000] MS - spectrum 0.08
779.25
780.26
m/z (Da)

	Formula	Calculated Mass	Target Mass	Double Bond Equivalence	Absolute Error (ppm)	Error (mDa)	Error (ppm)	Fitness
1	C20 H21 O8	389.12309	389.12322	11.0	0.32	0.12	0.32	1.000

	Formula	Calculated Mass	Target Mass	Double Bond Equivalence	Absolute Error (ppm)	Error (mDa)	Error (ppm)	Fitness
1	C40 H43 O16	779.25456	779.25384	20.0	0.93	-0.73	-0.93	0.994

Figure S137. UV spectrum of compound 14

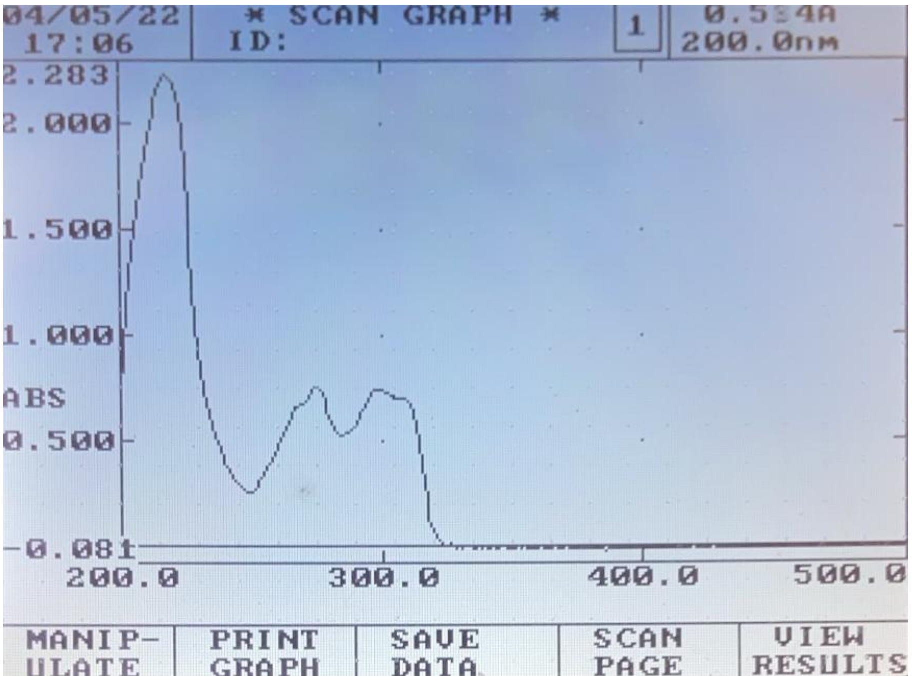

Figure S138. IR spectrum of compound 14

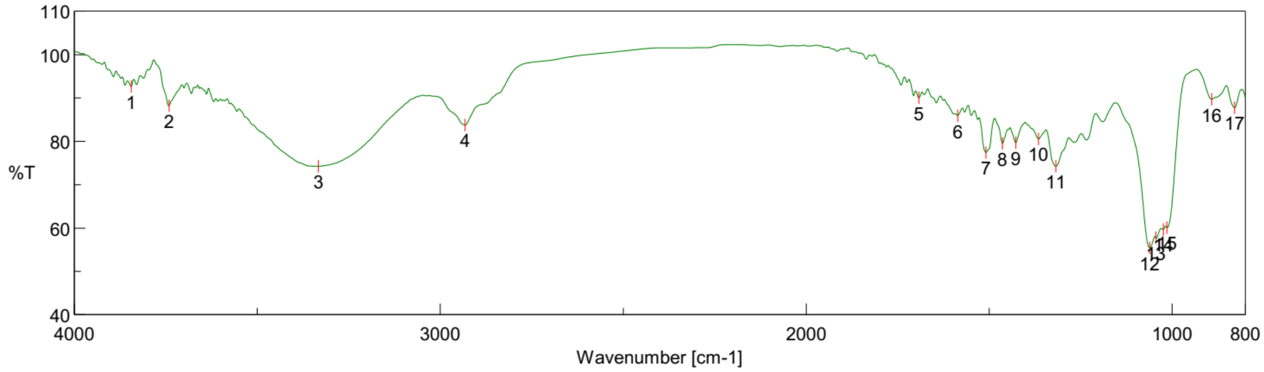

[Result of Peak Picking]

No.	Position	Intensity	No.	Position	Intensity	No.	Position	Intensity
1	3844.4	92.6321	2	3741.23	88.1781	3	3333.36	74.1973
4	2932.23	83.7266	5	1692.23	90.0149	6	1586.16	85.8786
7	1509.03	77.3864	8	1463.71	79.51	9	1427.07	79.6485
10	1365.35	80.5273	11	1318.11	74.1922	12	1061.62	55.3784
13	1044.26	57.7467	14	1024.02	59.6235	15	1014.37	60.0871
16	891.916	89.6533	17	829.241	87.7208	18	748.245	83.1063
19	676.892	81.0417	20	489.831	50.8738			

Figure S139. ^{1}H-NMR spectrum of compound 15

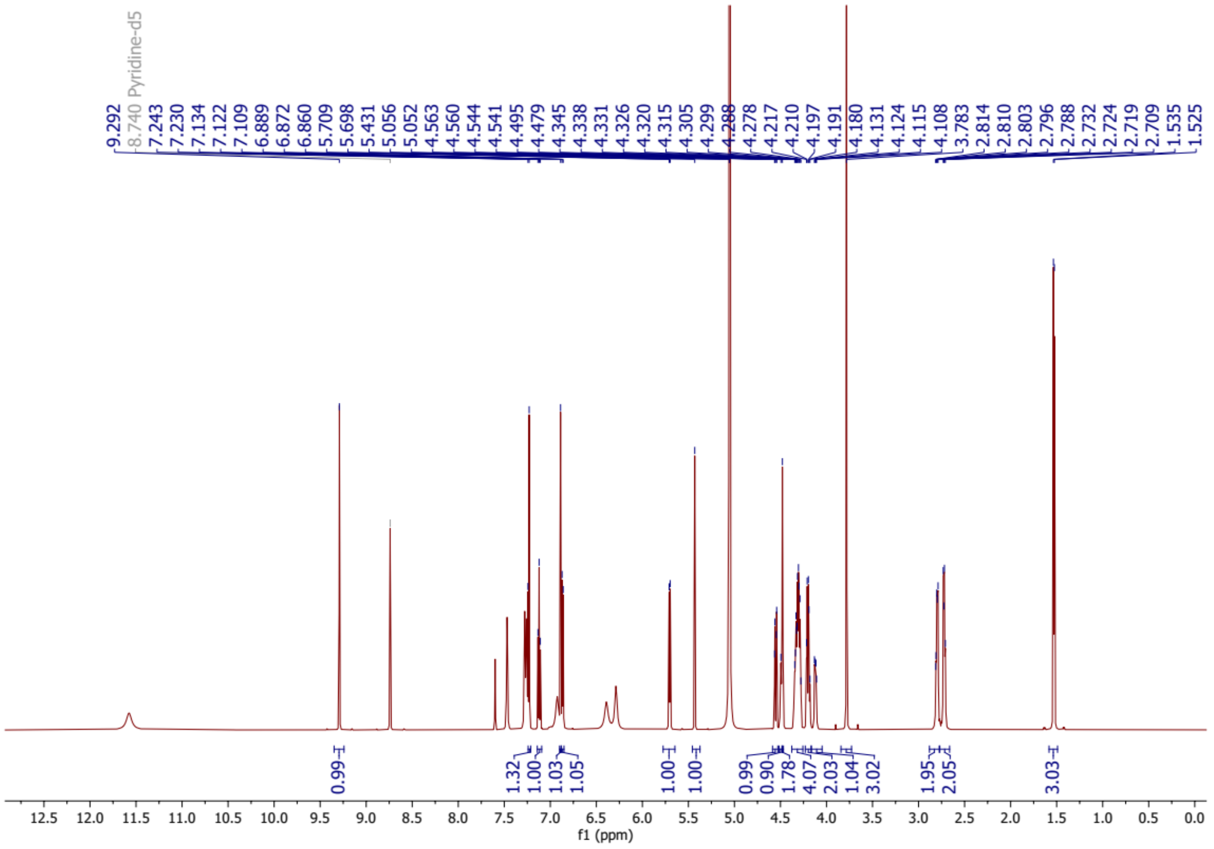

Figure S140. ^{13}C-NMR spectrum of compound 15

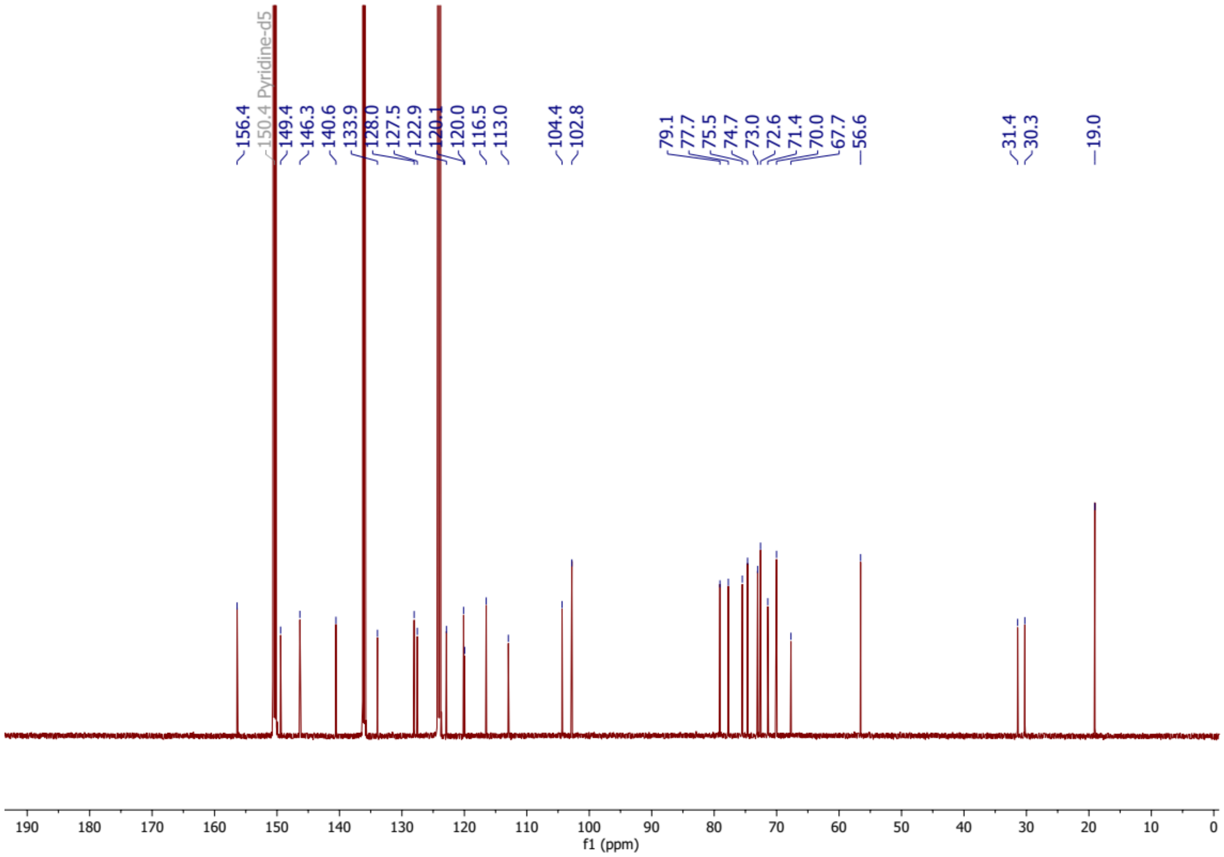

Figure S141. DEPT NMR spectrum of compound 15

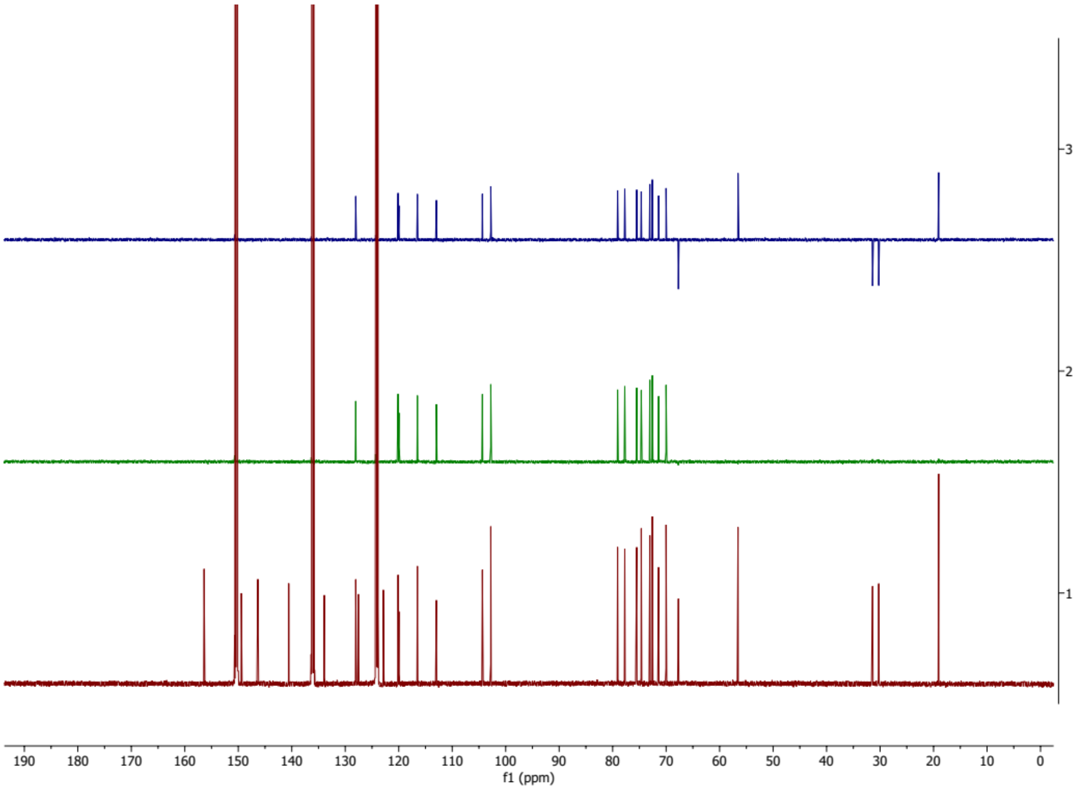

Figure S142. HSQC NMR spectrum of compound 15

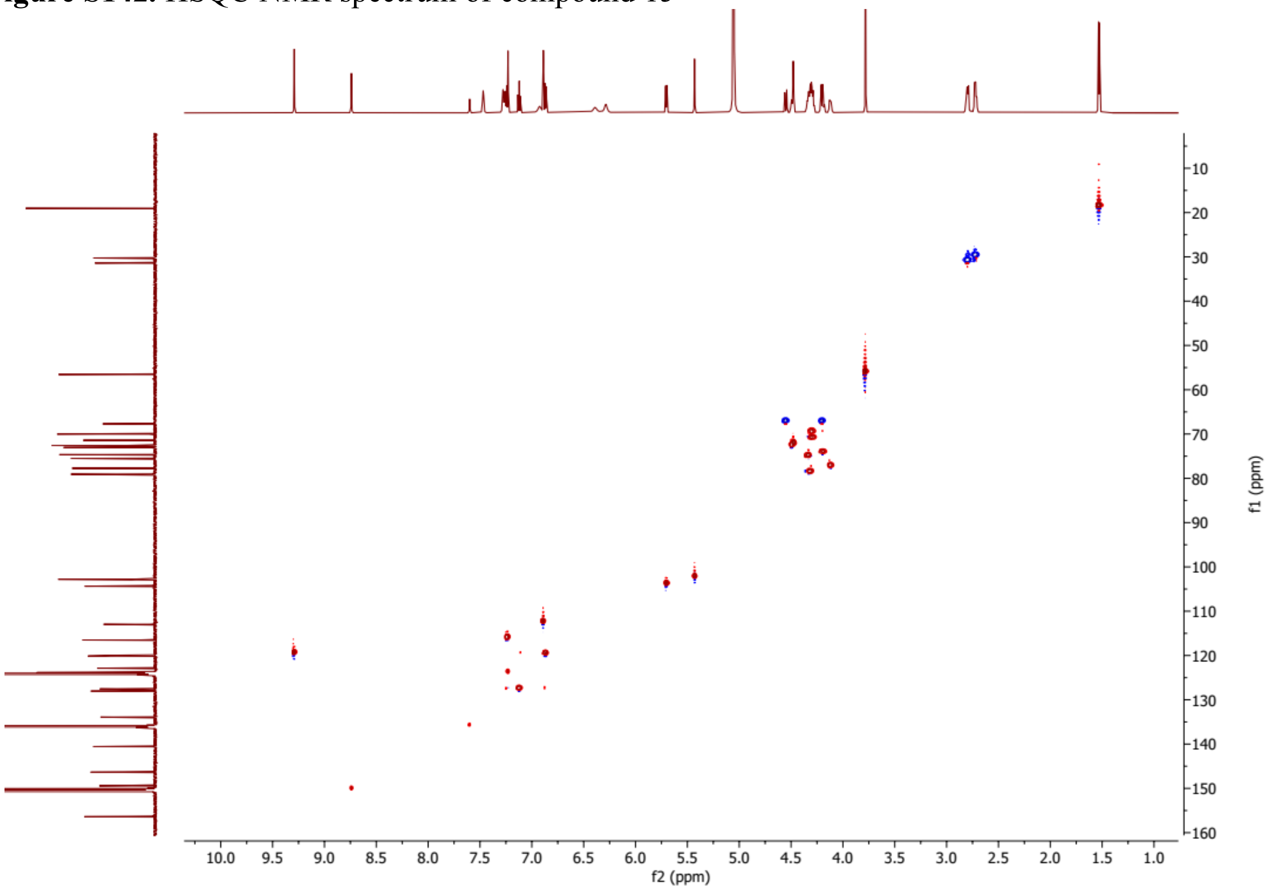

Figure S143. HMBC NMR spectrum of compound 15

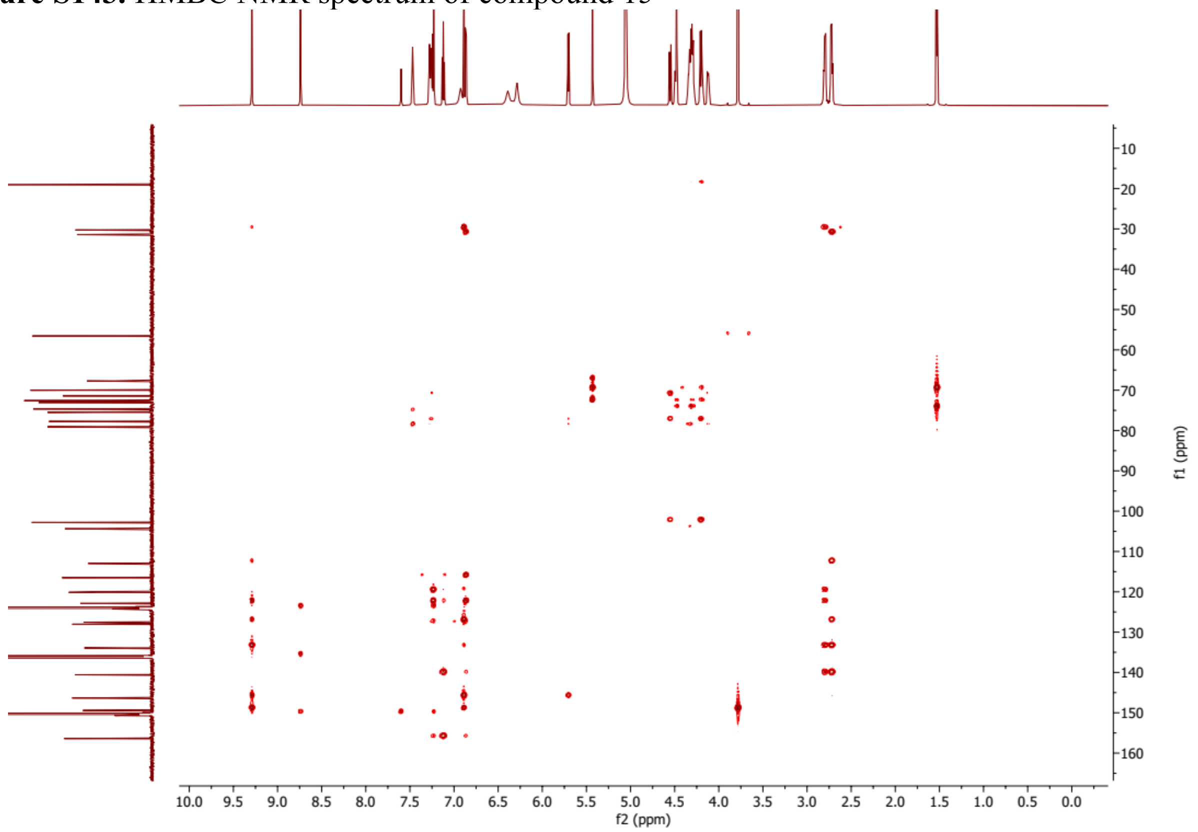

Figure S144. COSY NMR spectrum of compound 15

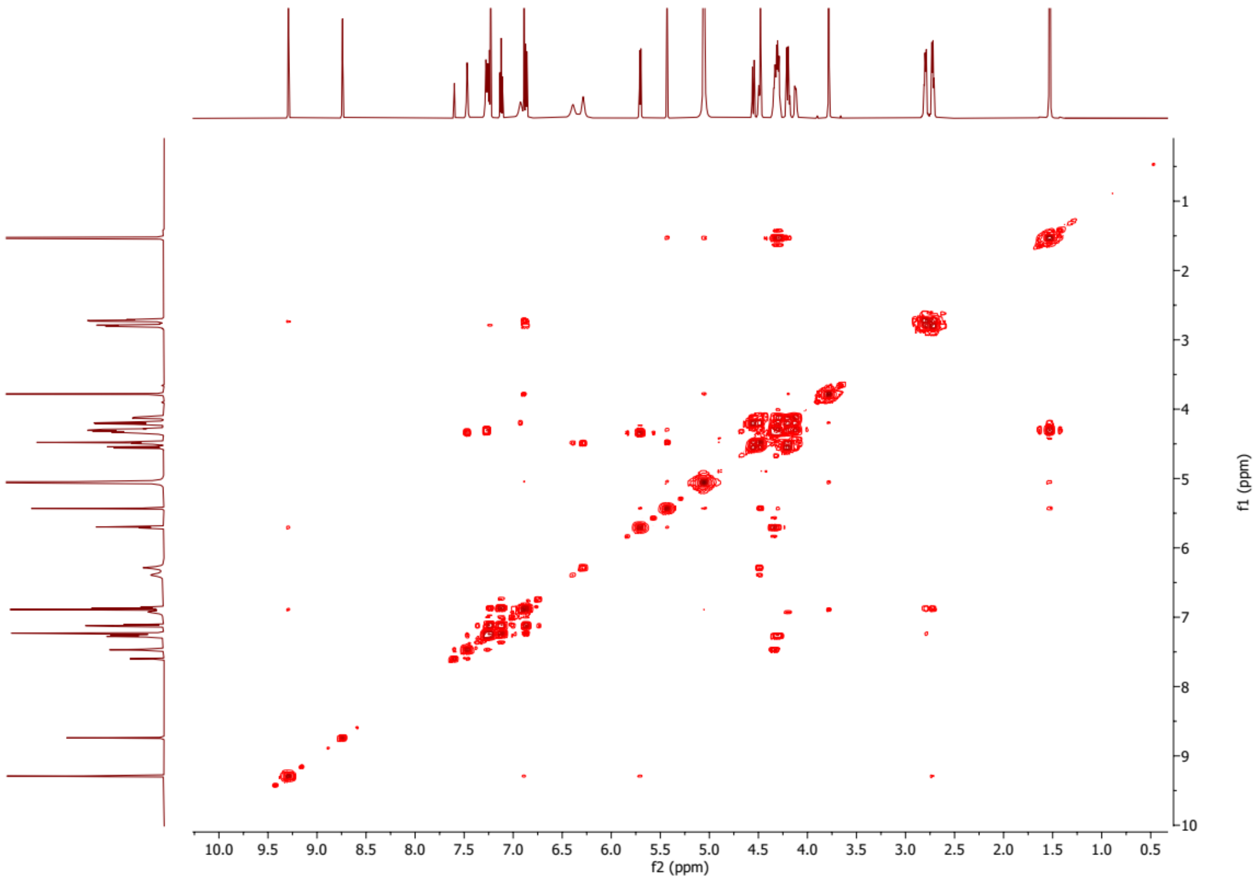

Figure S145. NOESY NMR spectrum of compound 15

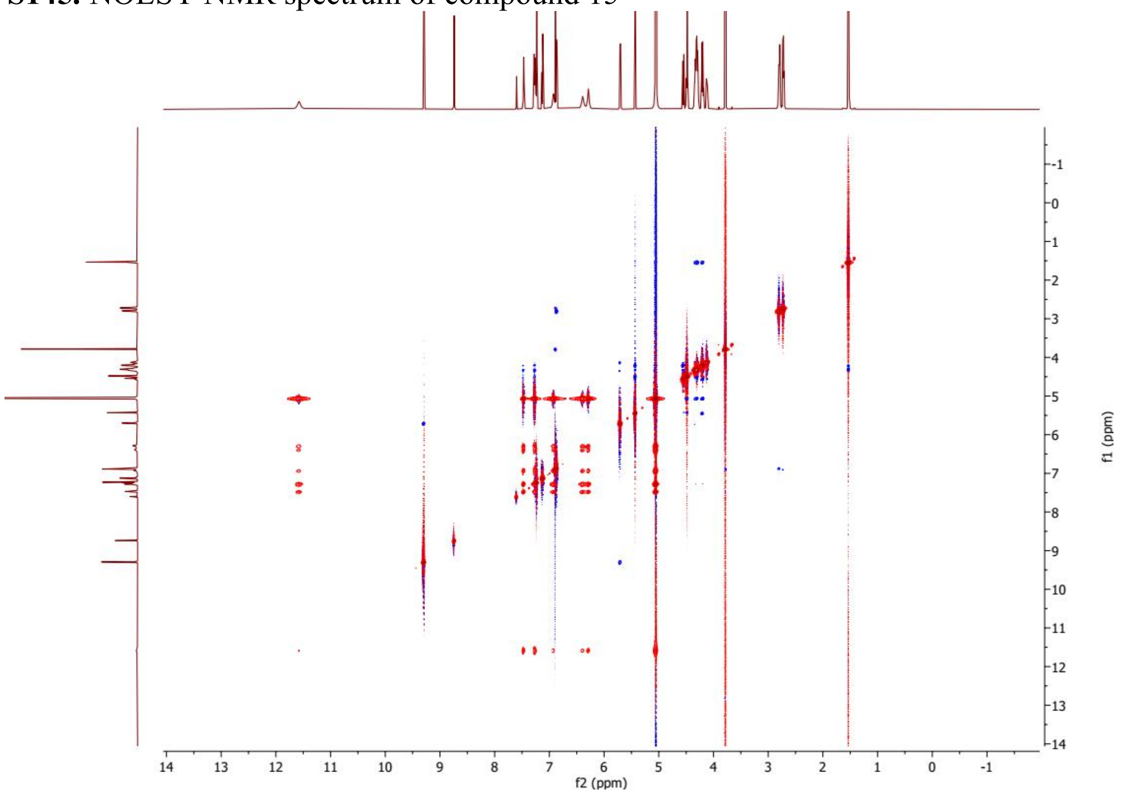

Figure S146. HR-ESI-MS spectrum of compound 15

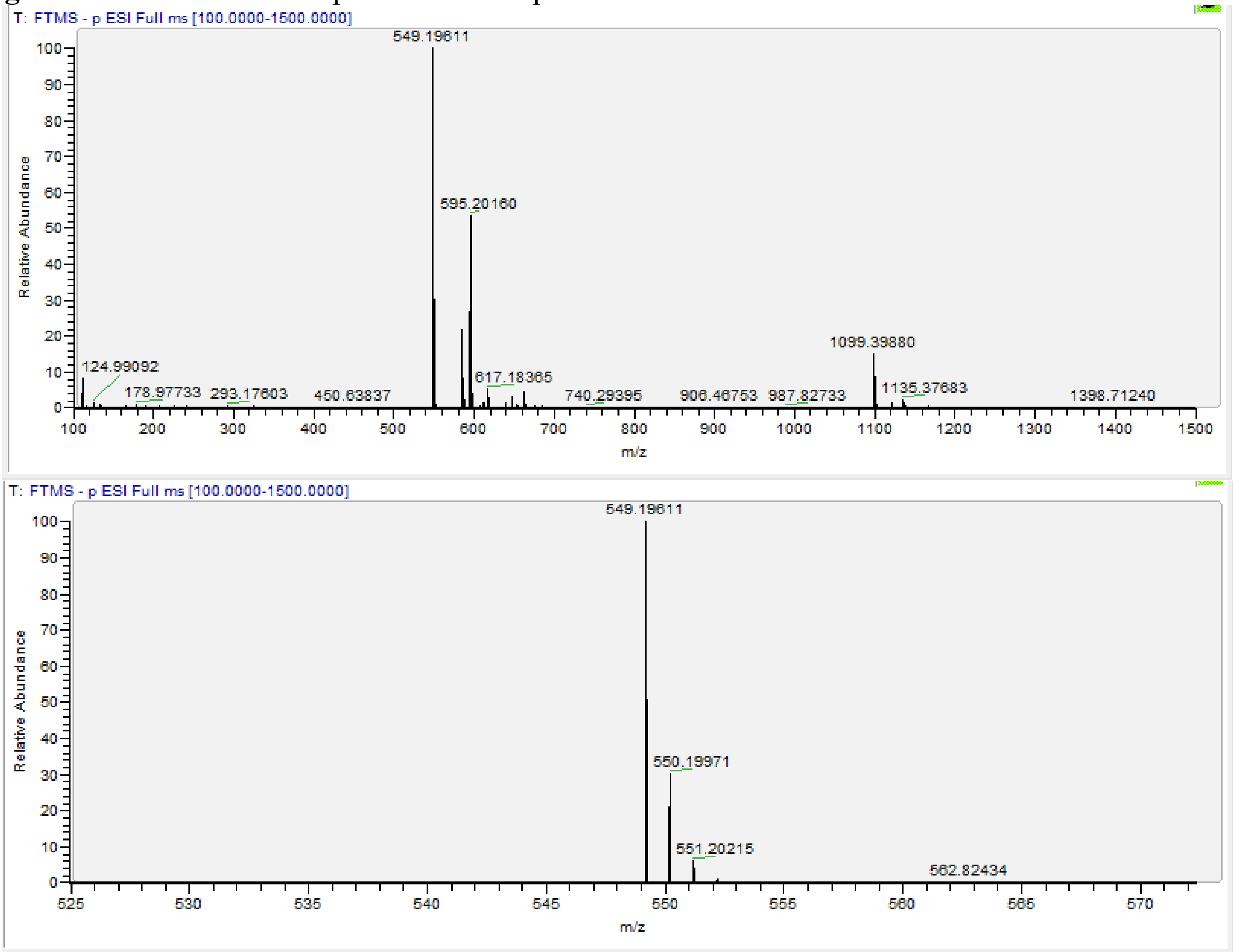

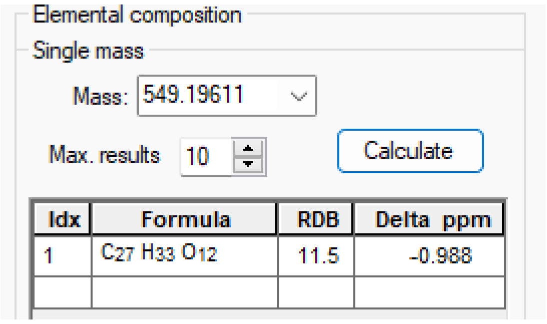

Elemental composition

Single mass

Mass: 549.19611

Max. results 10 Calculate

Idx	Formula	RDB	Delta ppm
1	$C_{27}H_{33}O_{12}$	11.5	-0.988

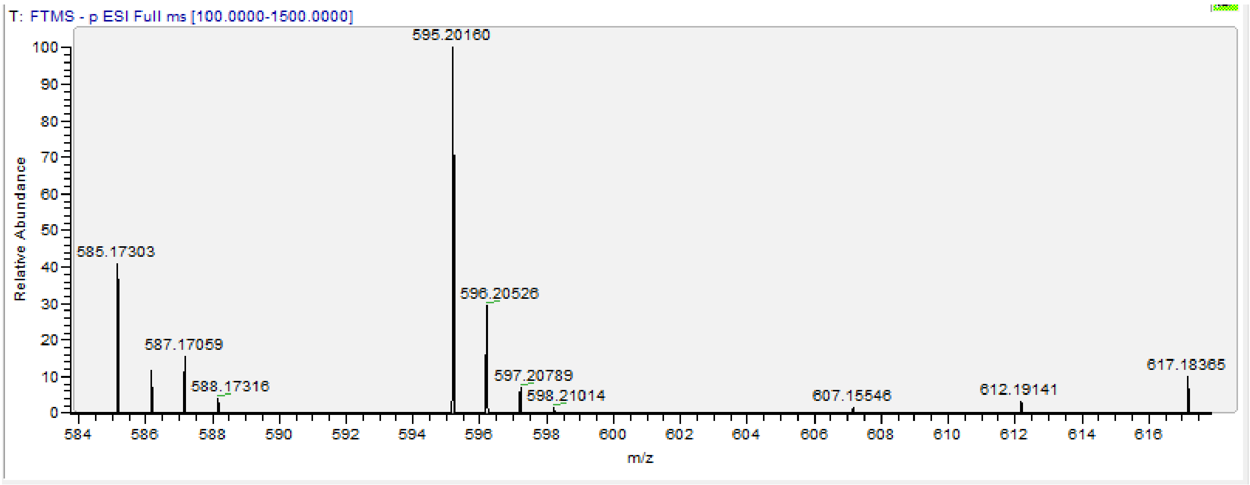

Elemental composition

Single mass

Mass: 595.20160

Max. results 10 Calculate

Idx	Formula	RDB	Delta ppm
1	$C_{28}H_{35}O_{14}$	11.5	-0.894

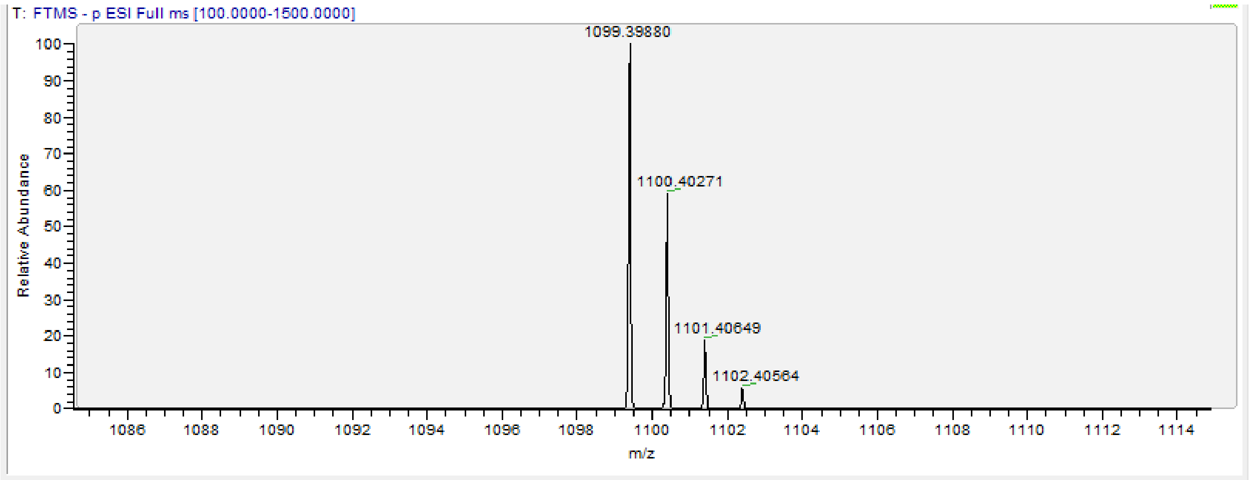

Elemental composition

Single mass

Mass: 1099.39880

Max. results 10 Calculate

Idx	Formula	RDB	Delta ppm
1	$C_{54}H_{67}O_{24}$	21.5	-2.619

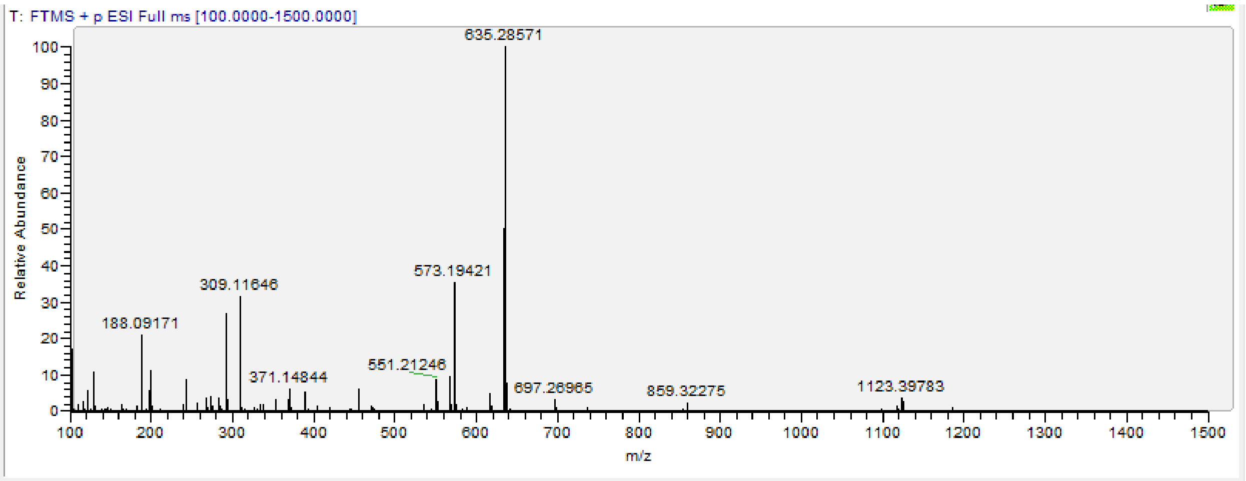

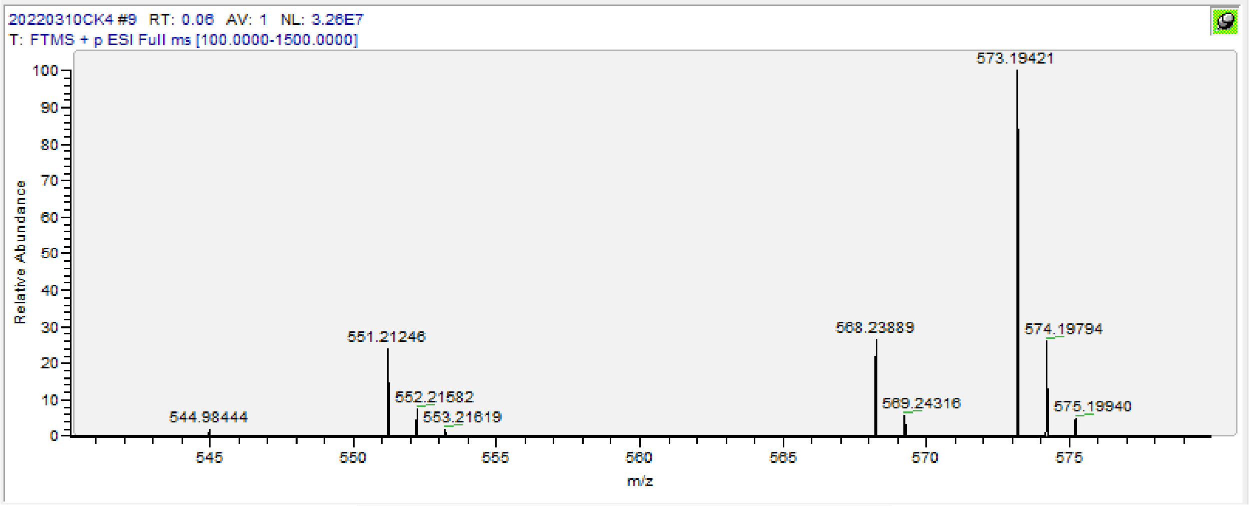

Elemental composition

Single mass

Mass: 551.21246

Max. results 10

Calculate

Idx	Formula	RDB	Delta ppm
1	$C_{27}H_{35}O_{12}$	10.5	0.285

Elemental composition

Single mass

Mass: 573.19421

Max. results 10

Calculate

Idx	Formula	RDB	Delta ppm
1	$C_{27}H_{34}O_{12}Na$	10.5	-0.065

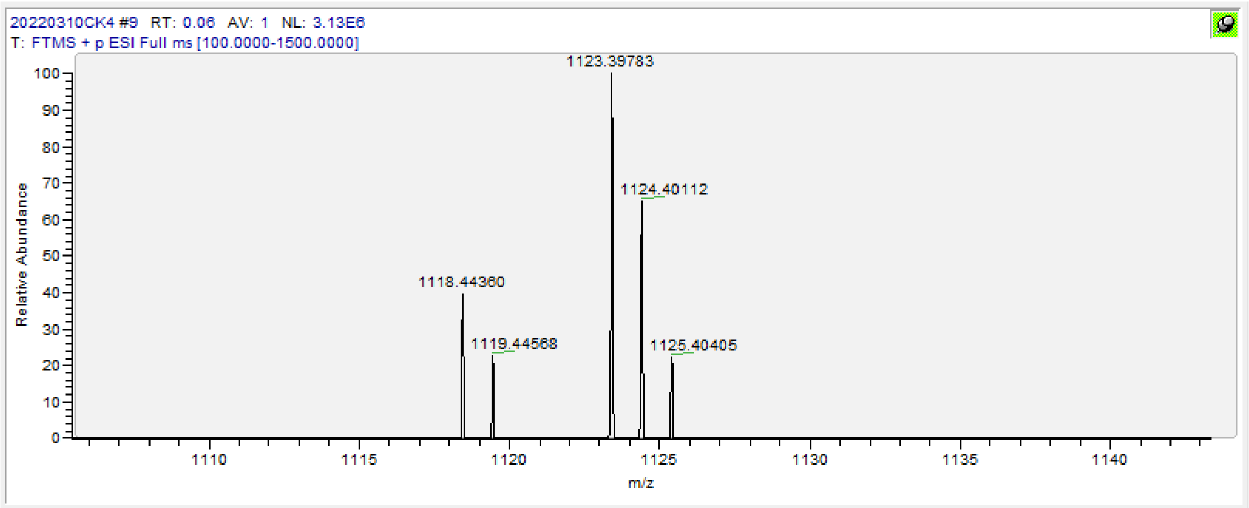

Elemental composition

Single mass

Mass: 1123.39783

Max. results 10

Calculate

Idx	Formula	RDB	Delta ppm
1	$C_{54}H_{68}O_{24}Na$	20.5	-1.285

Figure S147. UV spectrum of compound 15

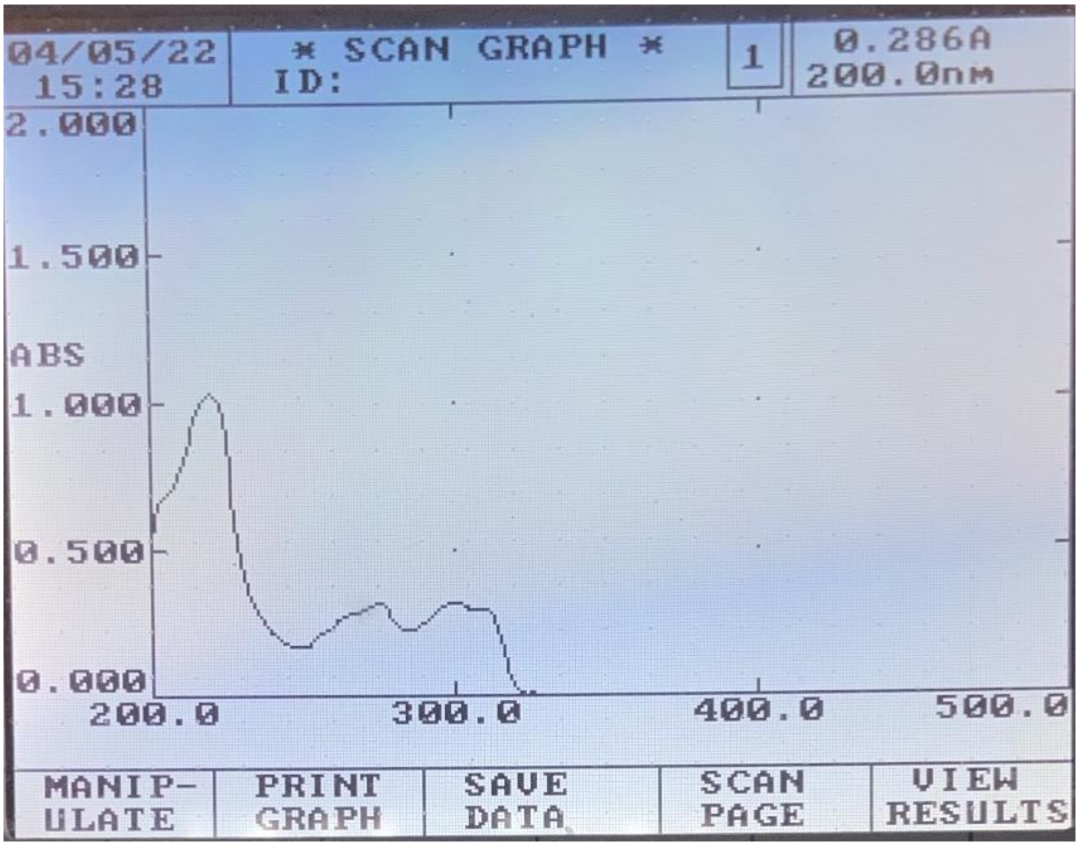

Figure S148. IR spectrum of compound 15

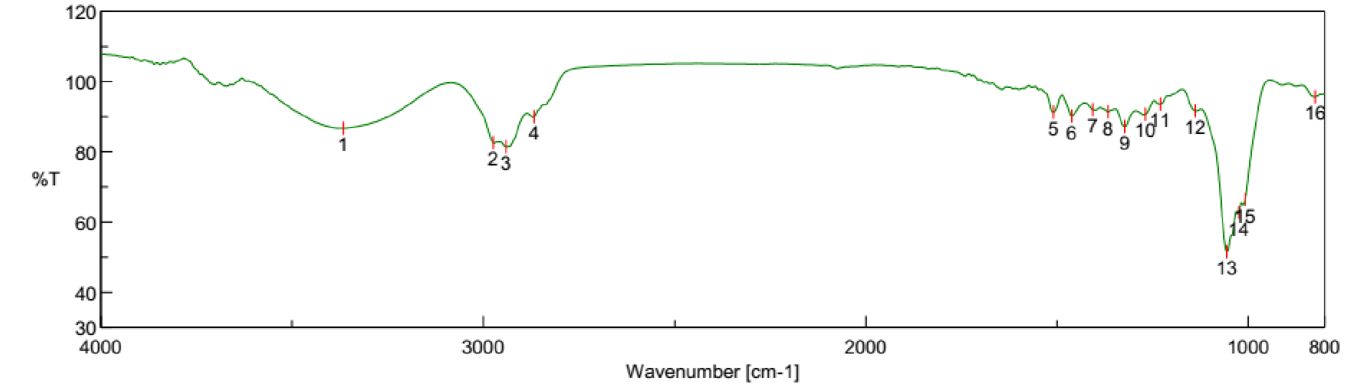

[Result of Peak Picking]

No.	Position	Intensity	No.	Position	Intensity	No.	Position	Intensity
1	3366.14	86.6383	2	2974.66	82.5674	3	2940.91	81.3968
4	2867.63	89.9041	5	1509.03	91.2351	6	1461.78	90.1536
7	1406.82	91.9897	8	1366.32	91.3362	9	1322.93	87.136
10	1268.93	90.6227	11	1229.4	93.5472	12	1138.76	91.6145
13	1055.84	51.4783	14	1024.02	62.7098	15	1007.62	66.4658
16	824.42	95.6163	17	674.963	93.1204			

Figure S149. ^{1}H-NMR spectrum of compound 16

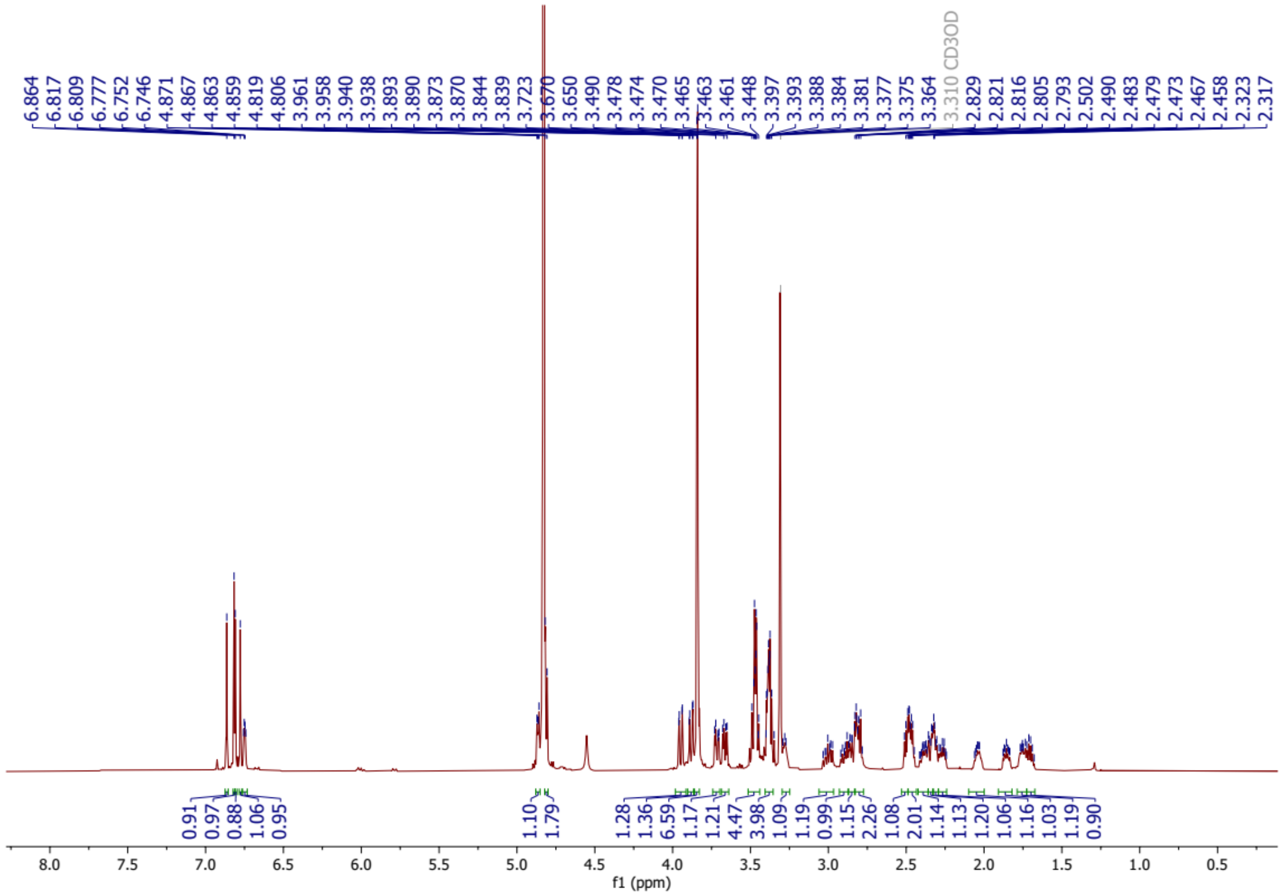

Figure S150. ^{13}C-NMR spectrum of compound 16

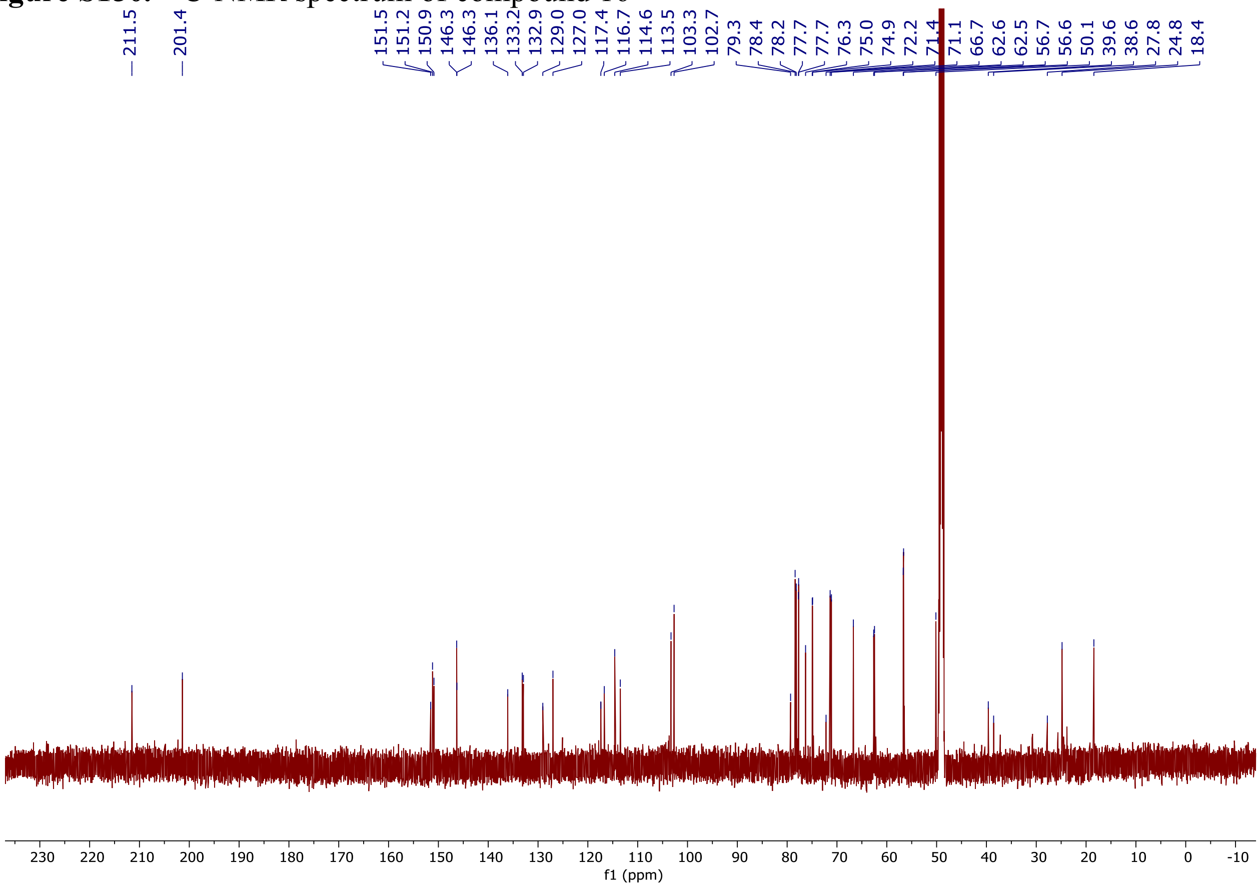

Figure S151. DEPT NMR spectrum of compound 16

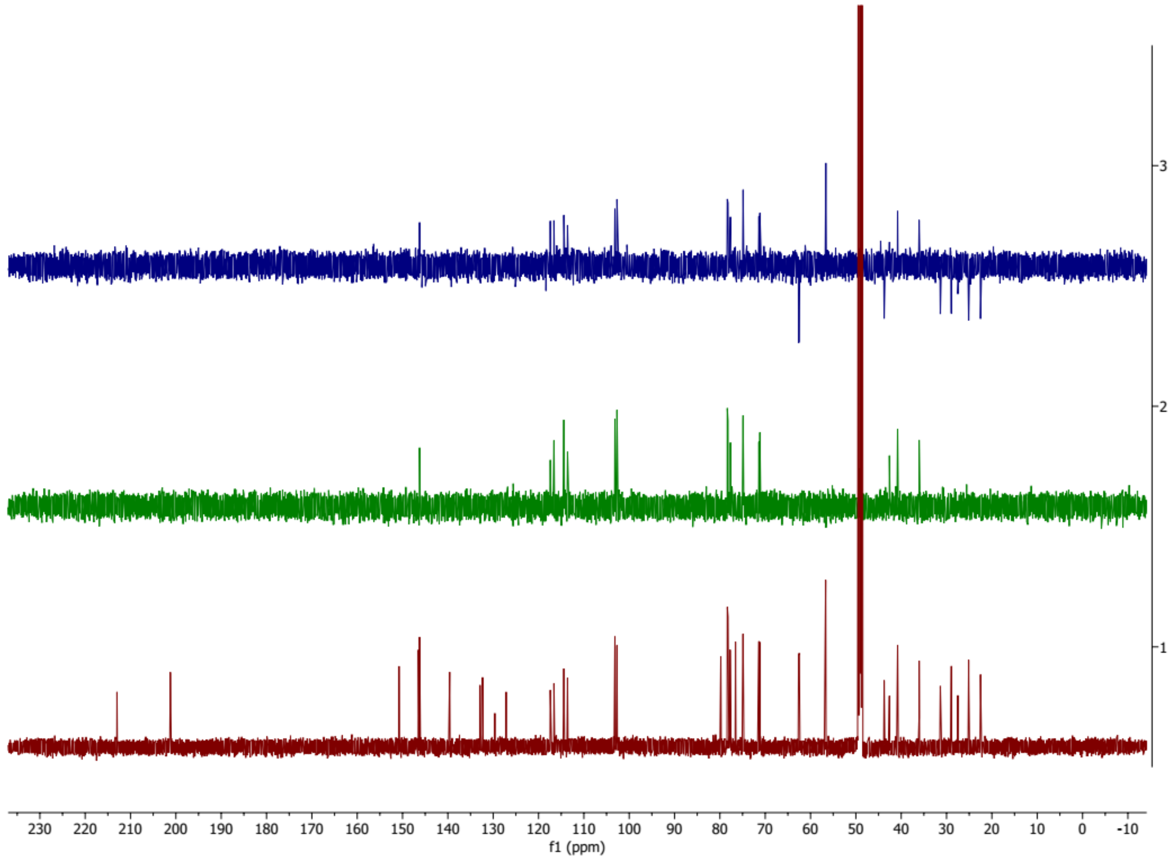

Figure S152. HSQC NMR spectrum of compound 16

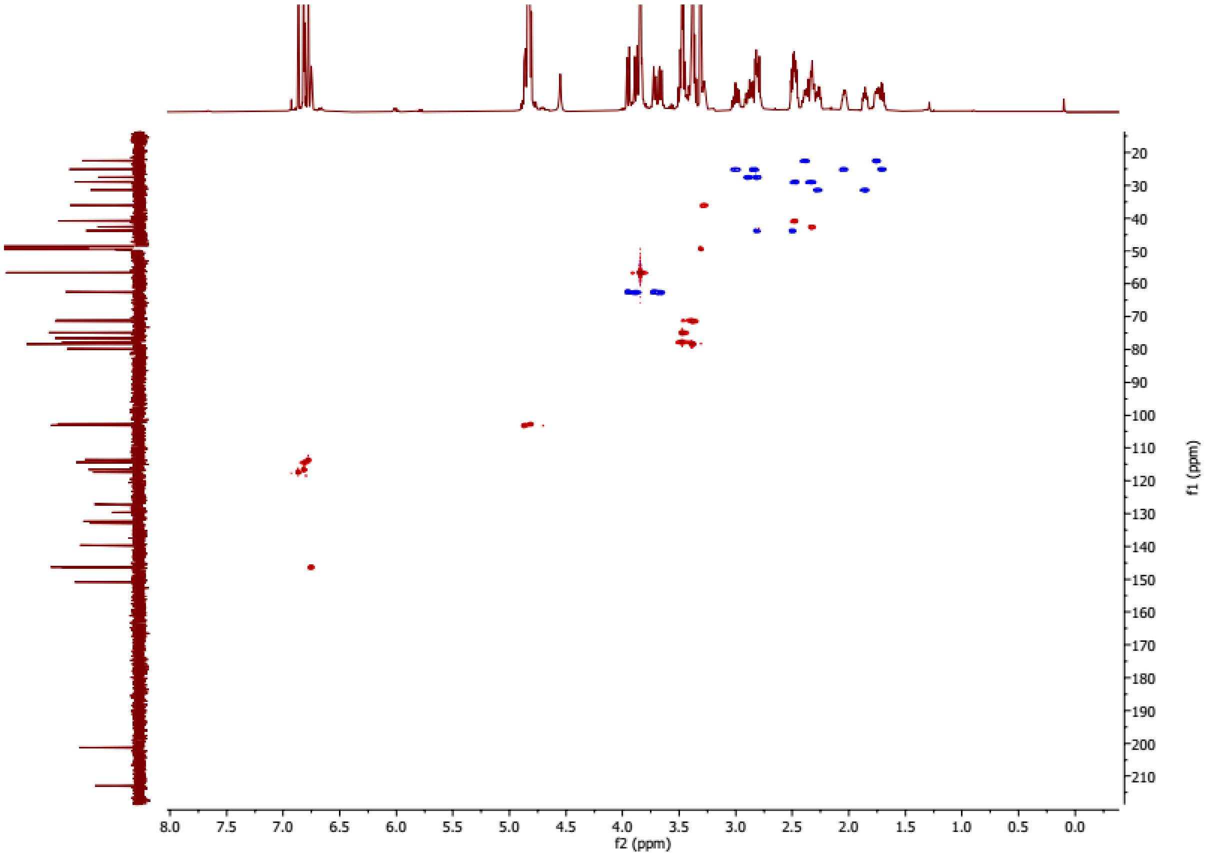

Figure S153. HMBC NMR spectrum of compound 16

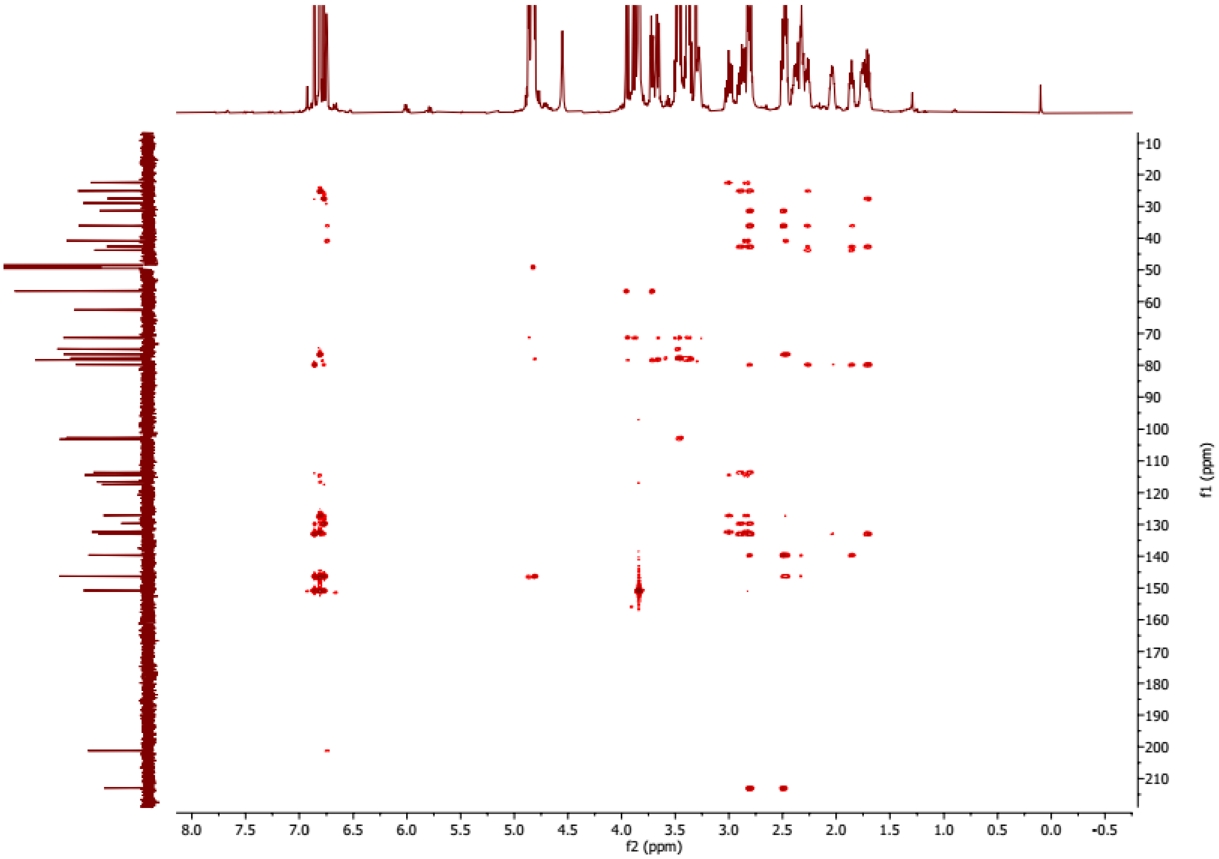

Figure S154. COSY NMR spectrum of compound 16

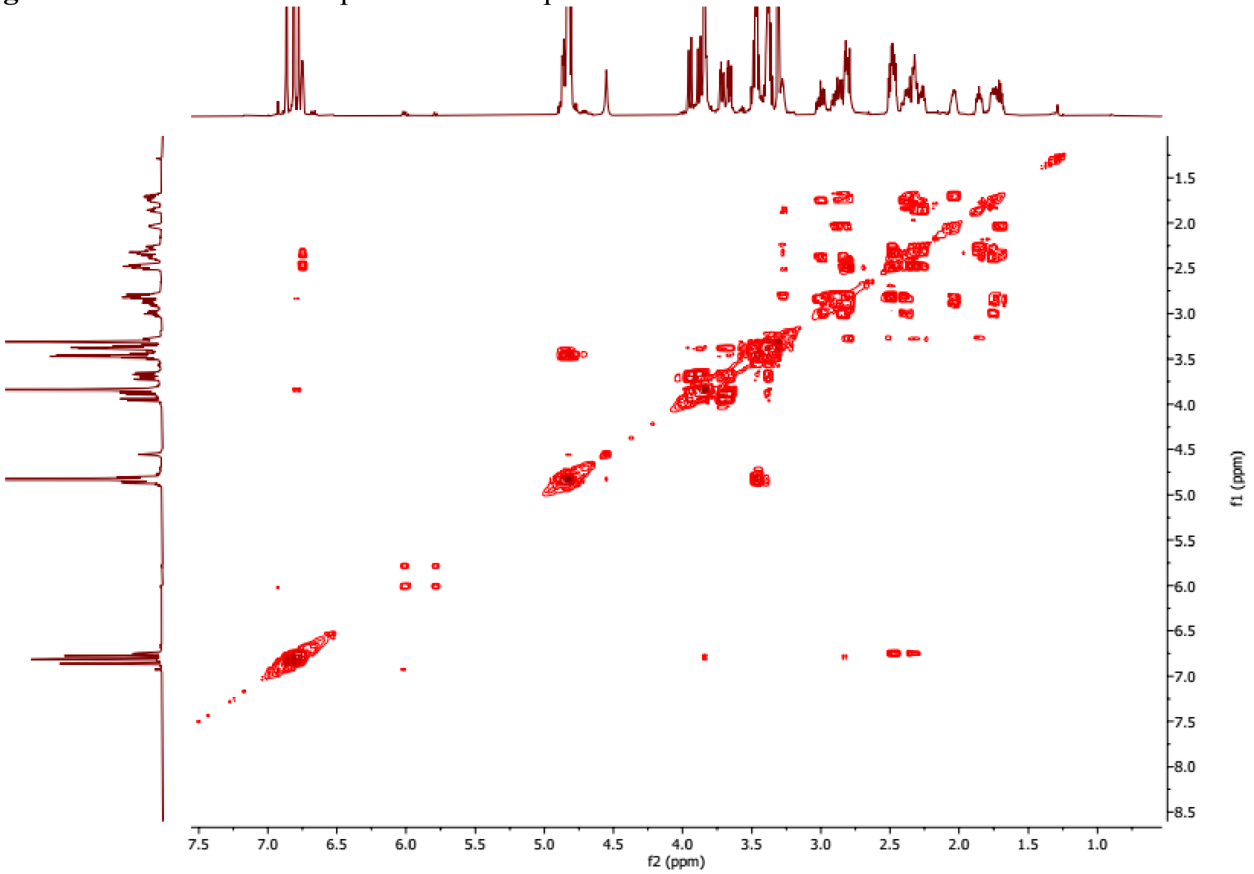

Figure S155. NOESY NMR spectrum of compound 16

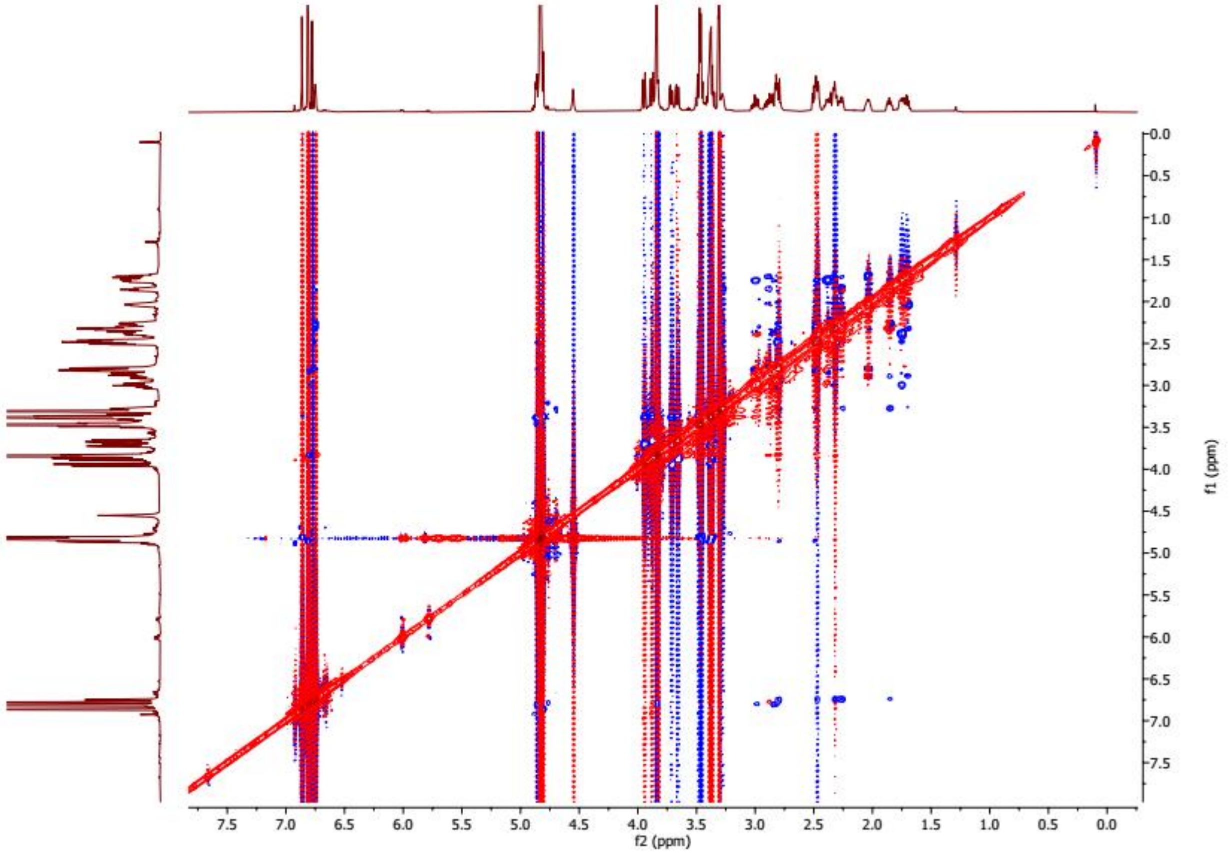

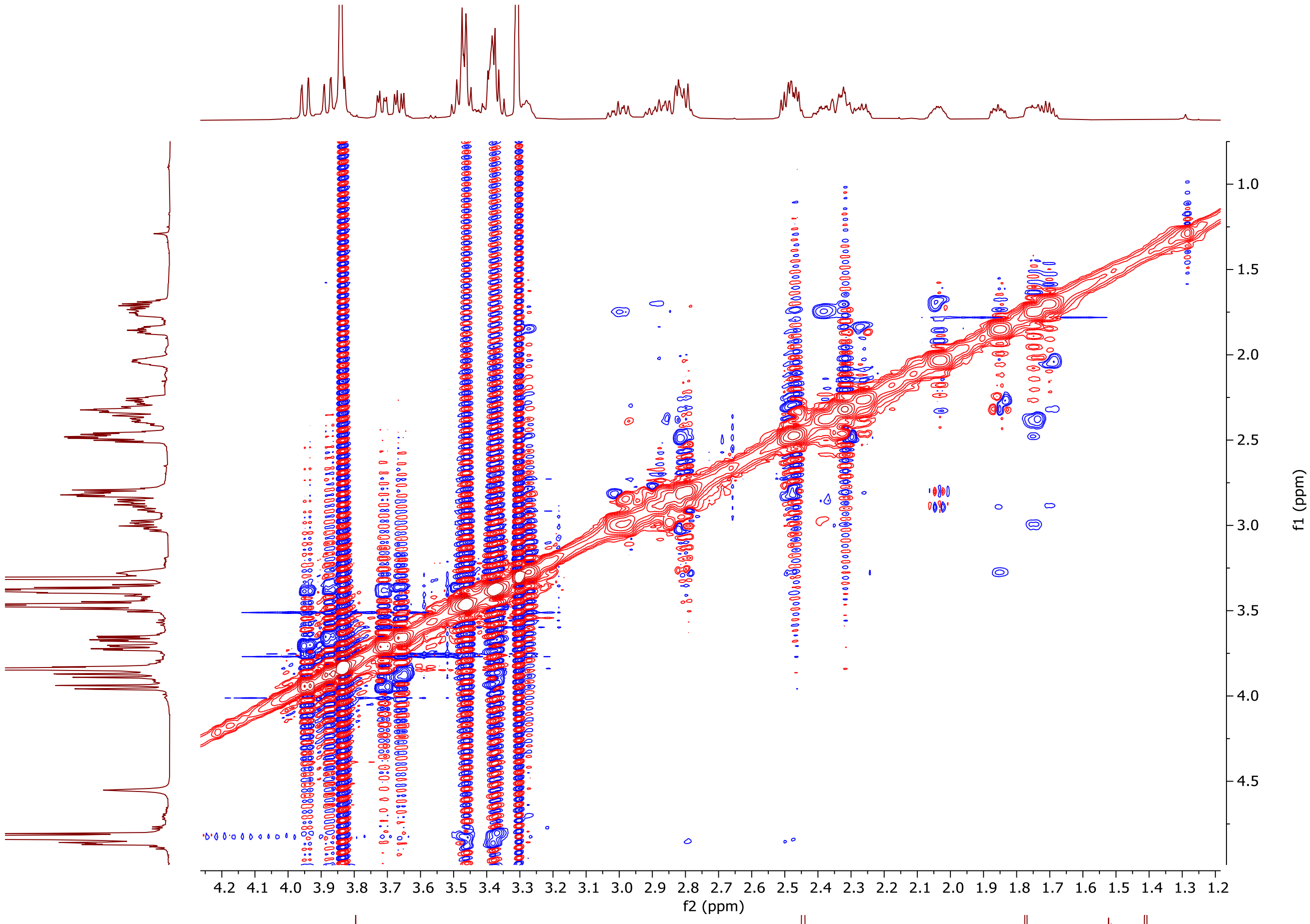

f2 (ppm)
f1 (ppm)

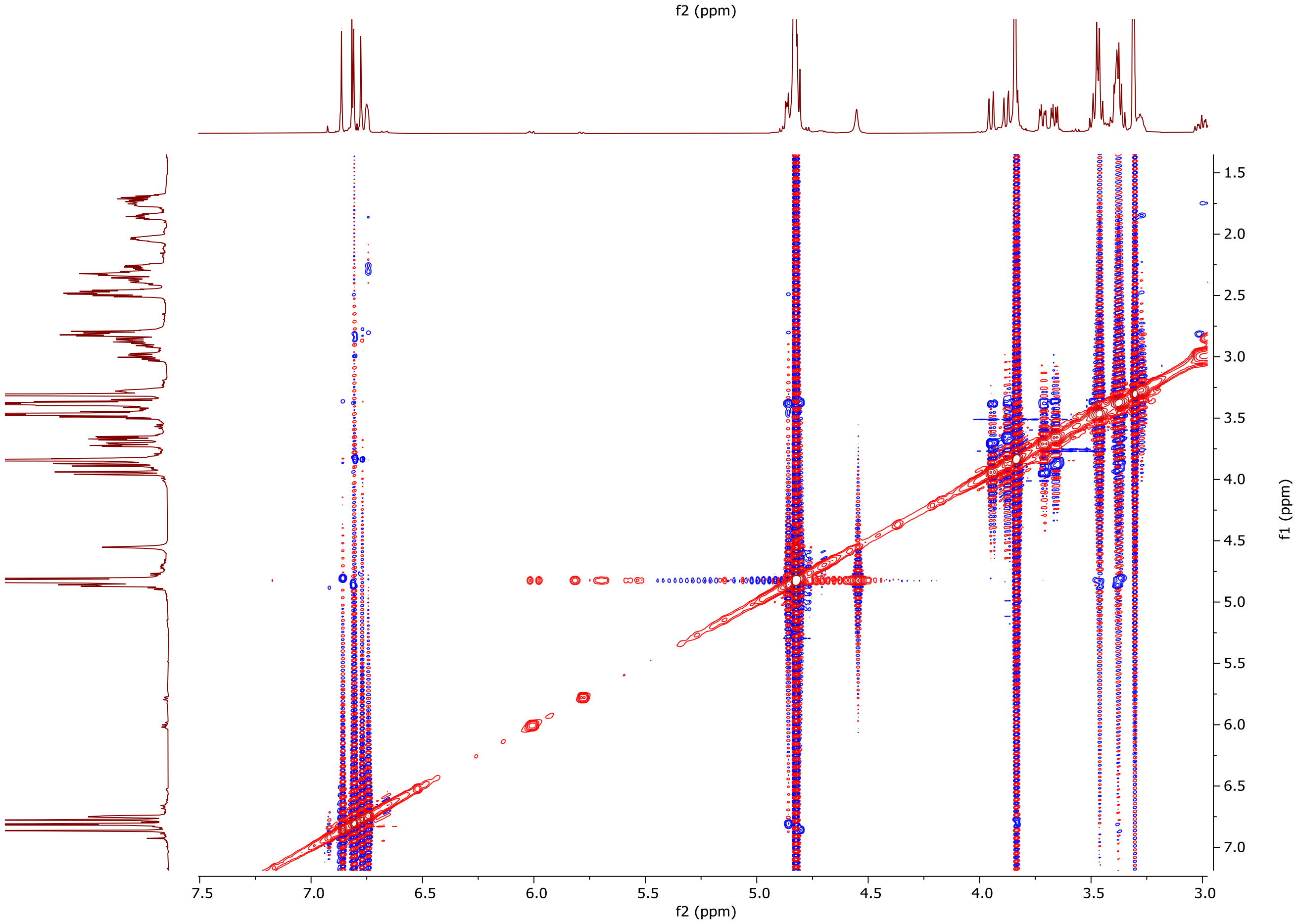

f2 (ppm)
f1 (ppm)

Figure S156. HR-ESI-MS spectrum of compound 16

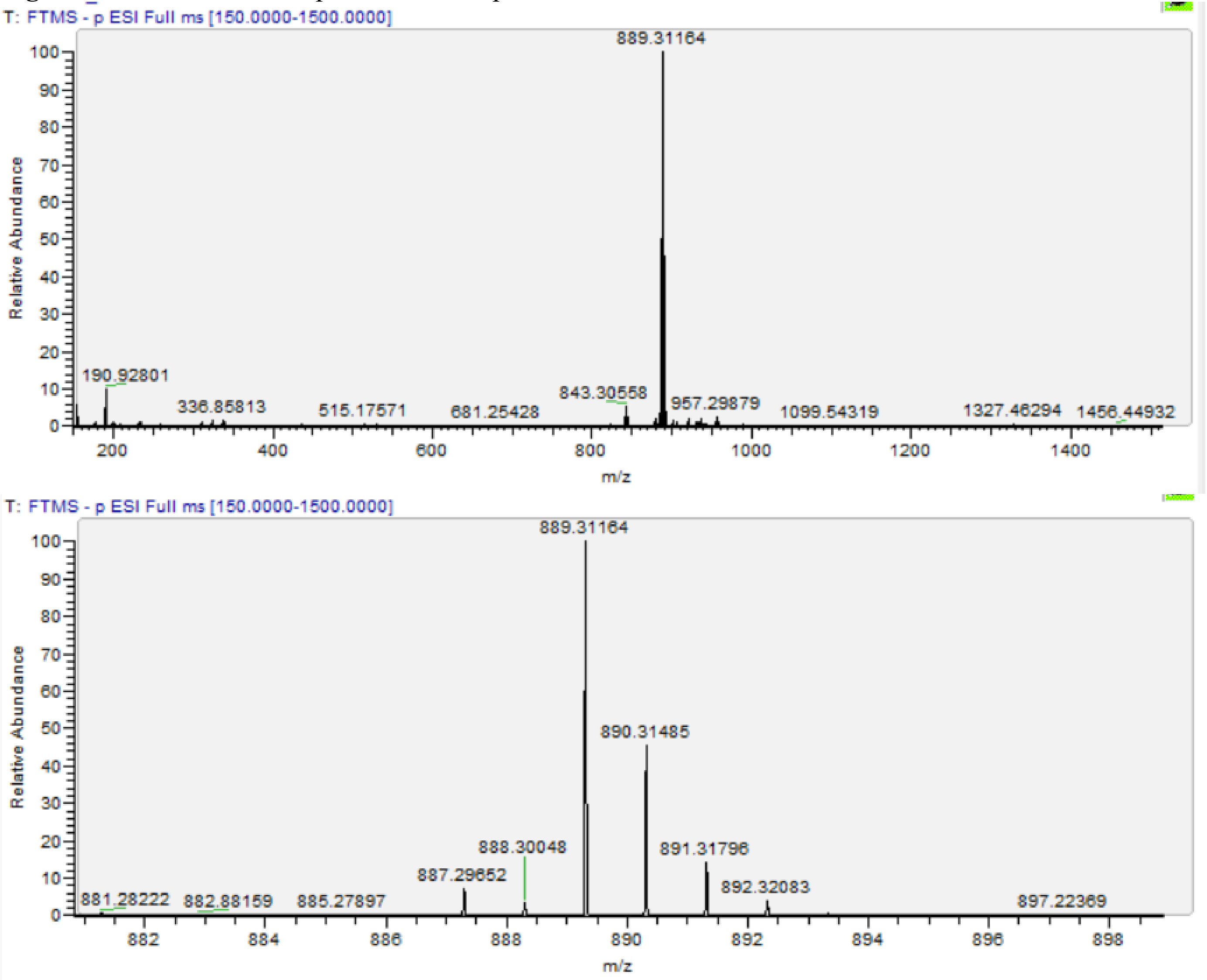

Single mass

Mass: 889.31164

Max. results 10

Calculate

Idx	Formula	RDB	Delta ppm
1	$C_{43}H_{53}O_{20}$	17.5	-0.934

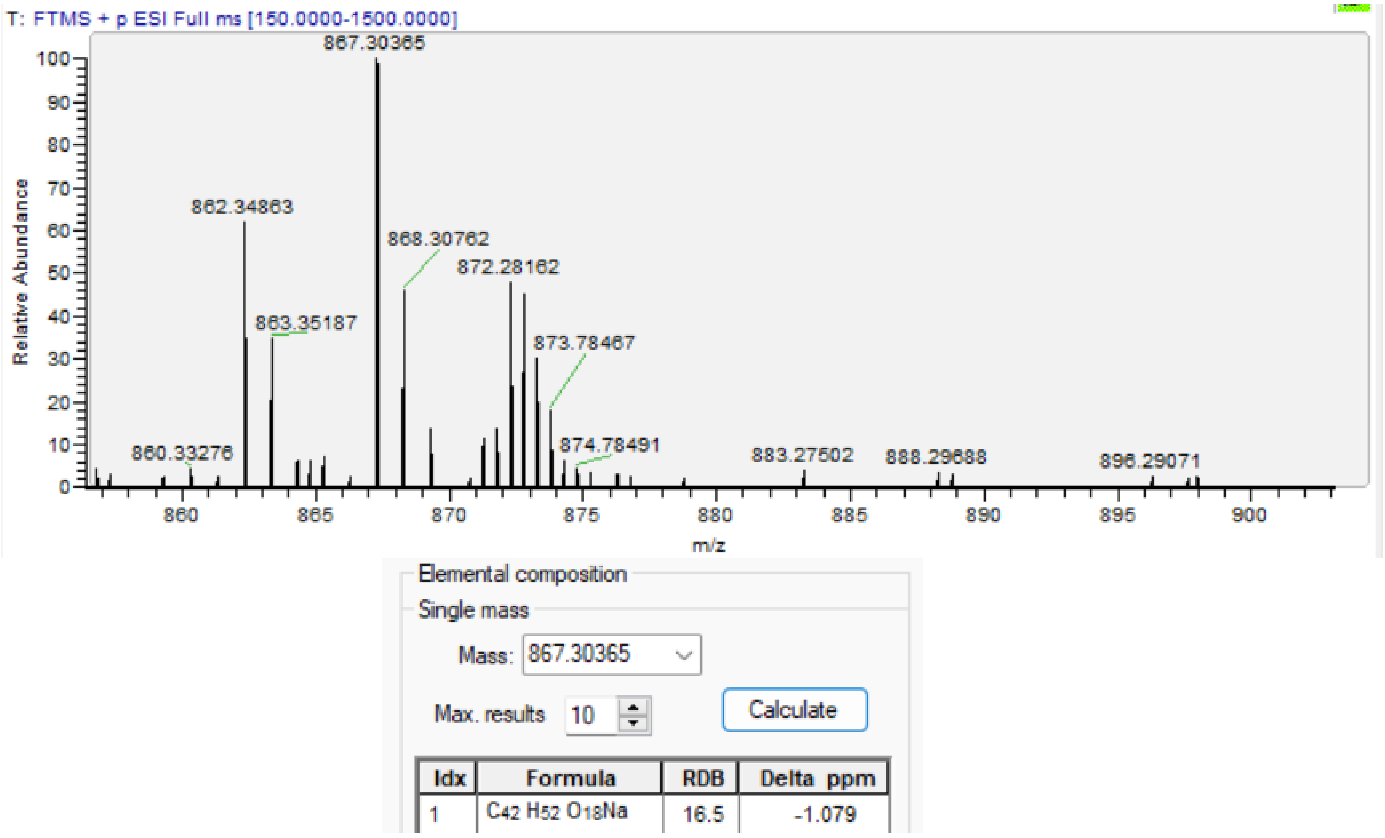

Figure S157. UV spectrum of compound 16

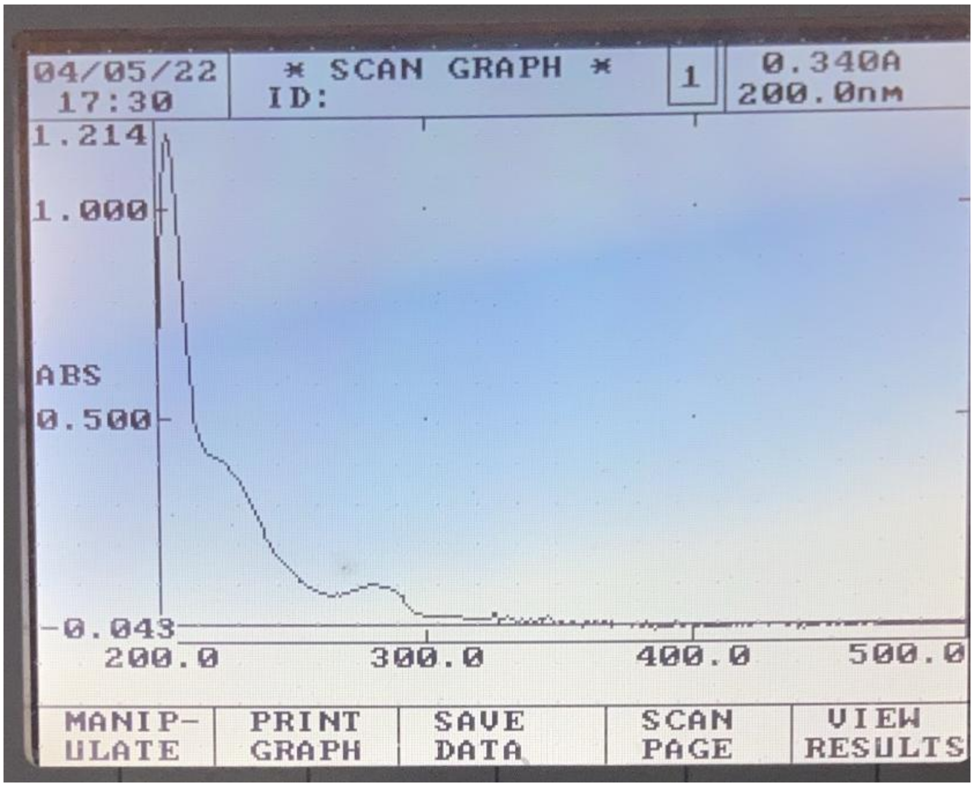

Figure S158. IR spectrum of compound 16

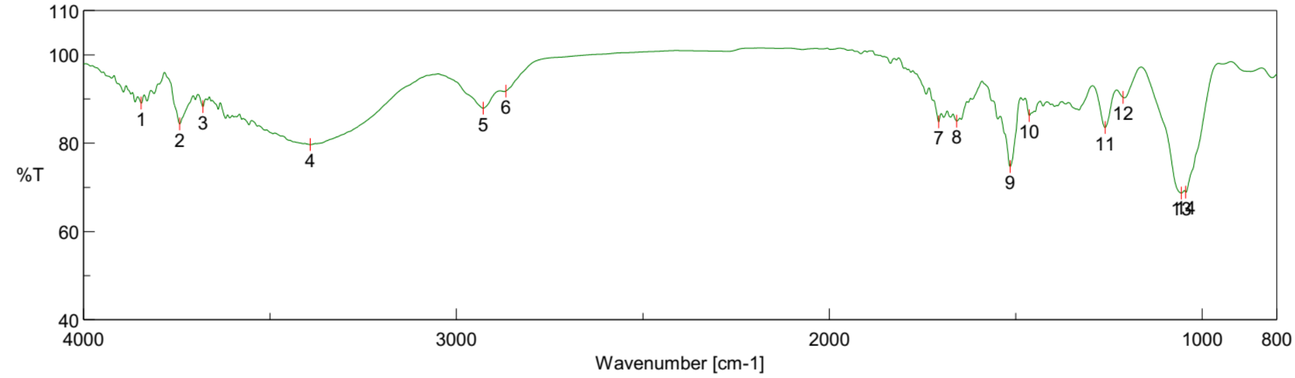

[Result of Peak Picking]

No.	Position	Intensity	No.	Position	Intensity	No.	Position	Intensity
1	3845.36	89.0493	2	3742.19	84.3395	3	3679.51	88.2448
4	3392.17	79.6371	5	2928.38	87.8687	6	2867.63	91.7352
7	1706.69	84.798	8	1658.48	84.9477	9	1514.81	74.6452
10	1463.71	86.2151	11	1260.25	83.5346	12	1212.04	90.3313
13	1055.84	68.6868	14	1044.26	68.8987			

Figure S159. ^{1}H-NMR spectrum of compound 17

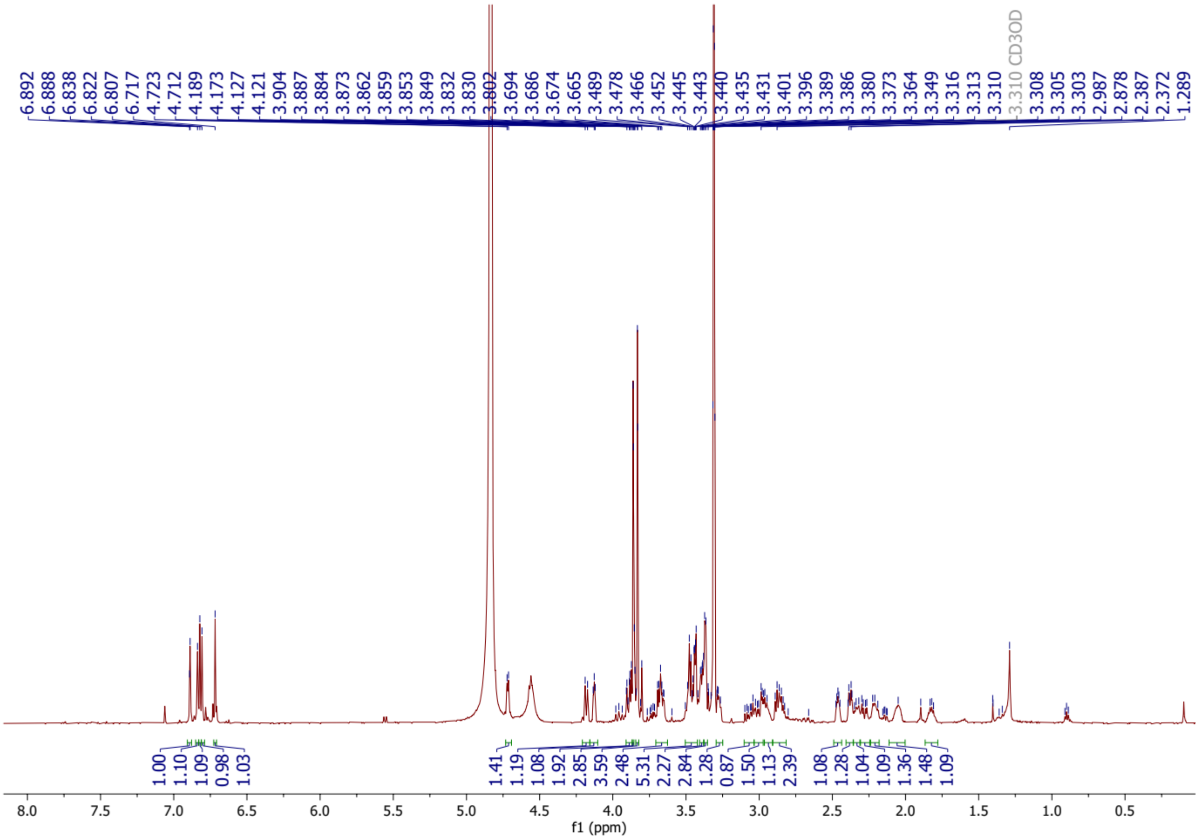

Figure S160. ^{13}C-NMR spectrum of compound 17

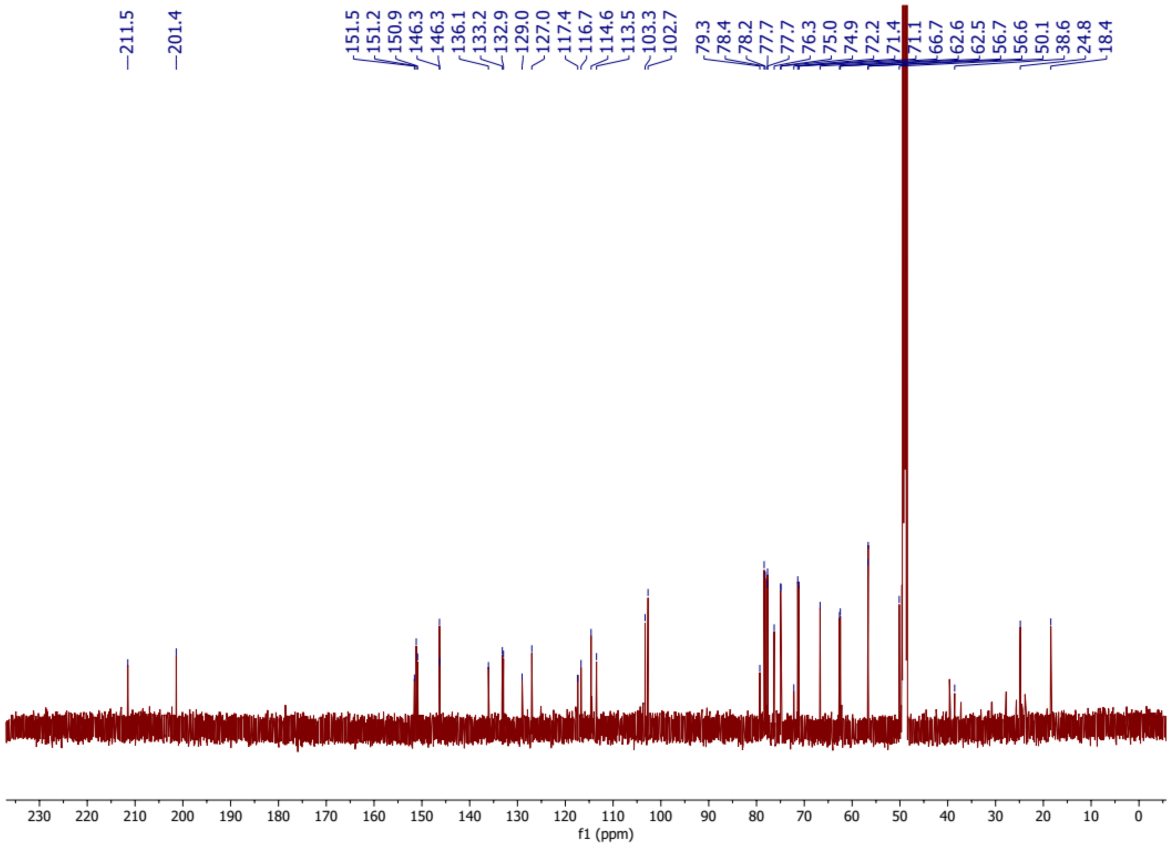

Figure S161. DEPT NMR spectrum of compound 17

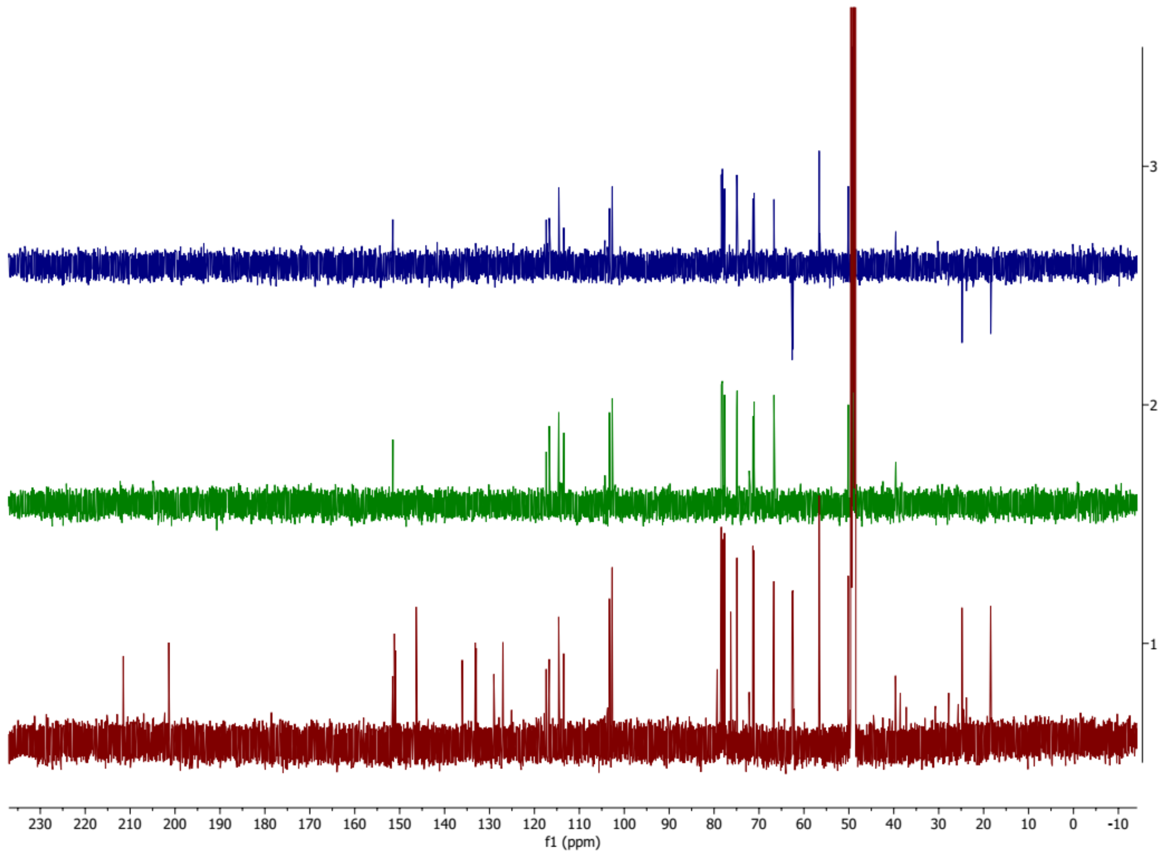

Figure S162. HSQC NMR spectrum of compound 17

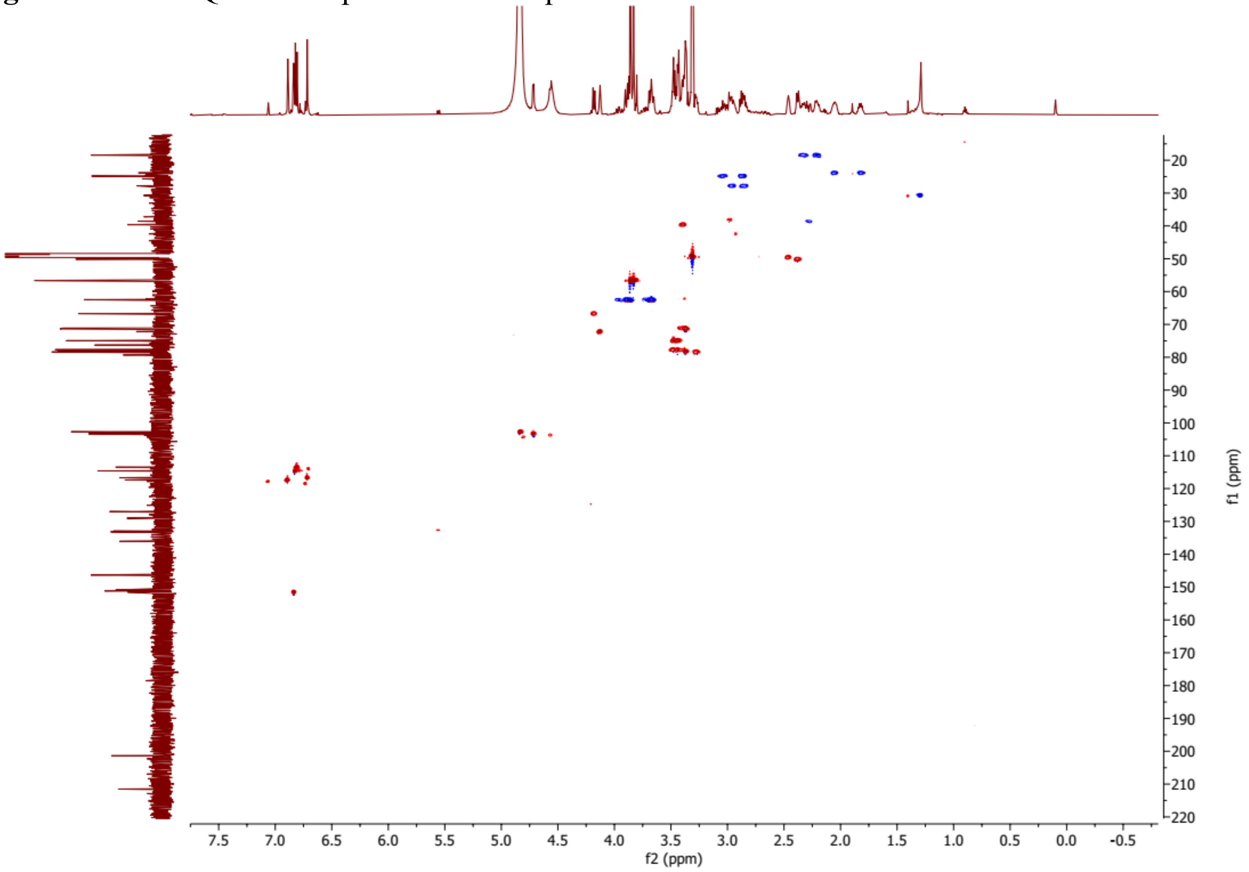

Figure S163. HMBC NMR spectrum of compound 17

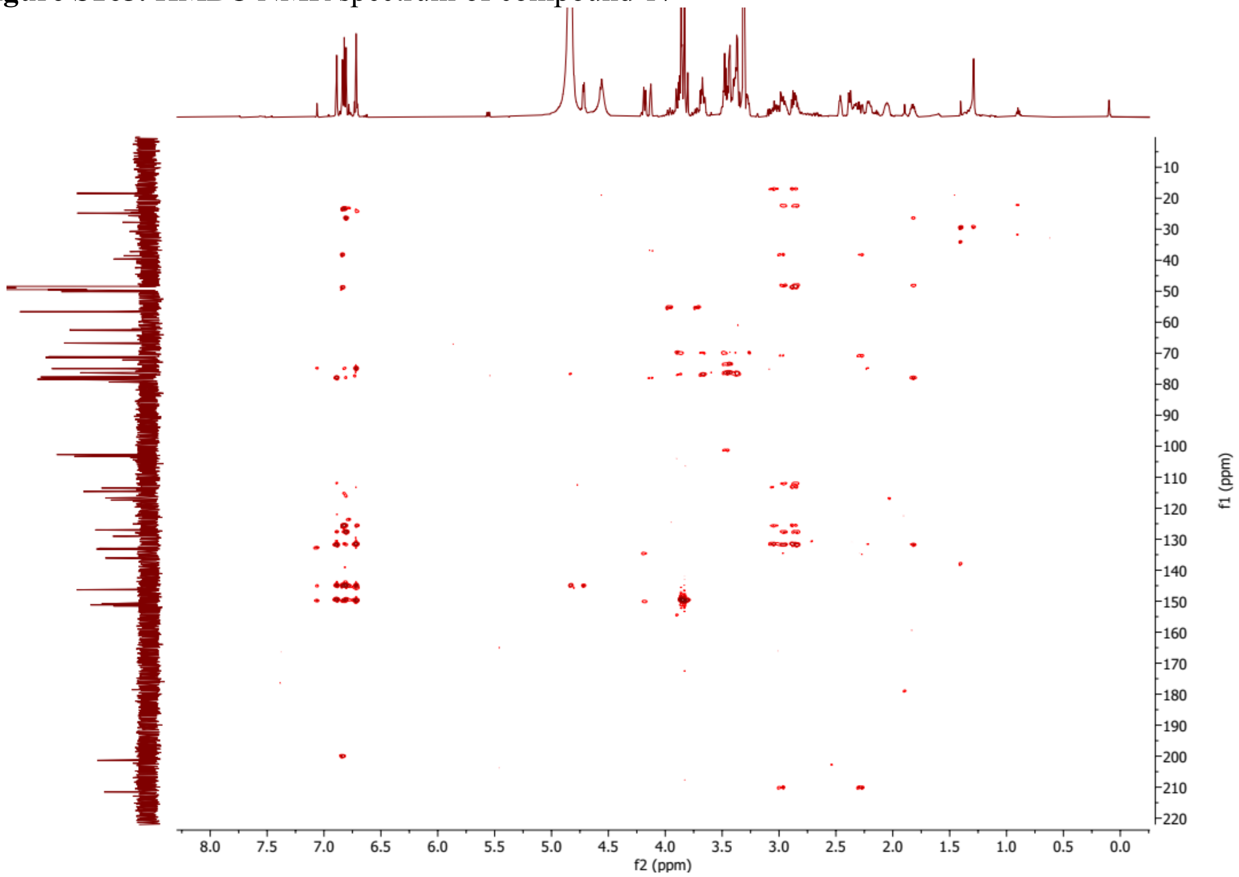

Figure S164. COSY NMR spectrum of compound 17

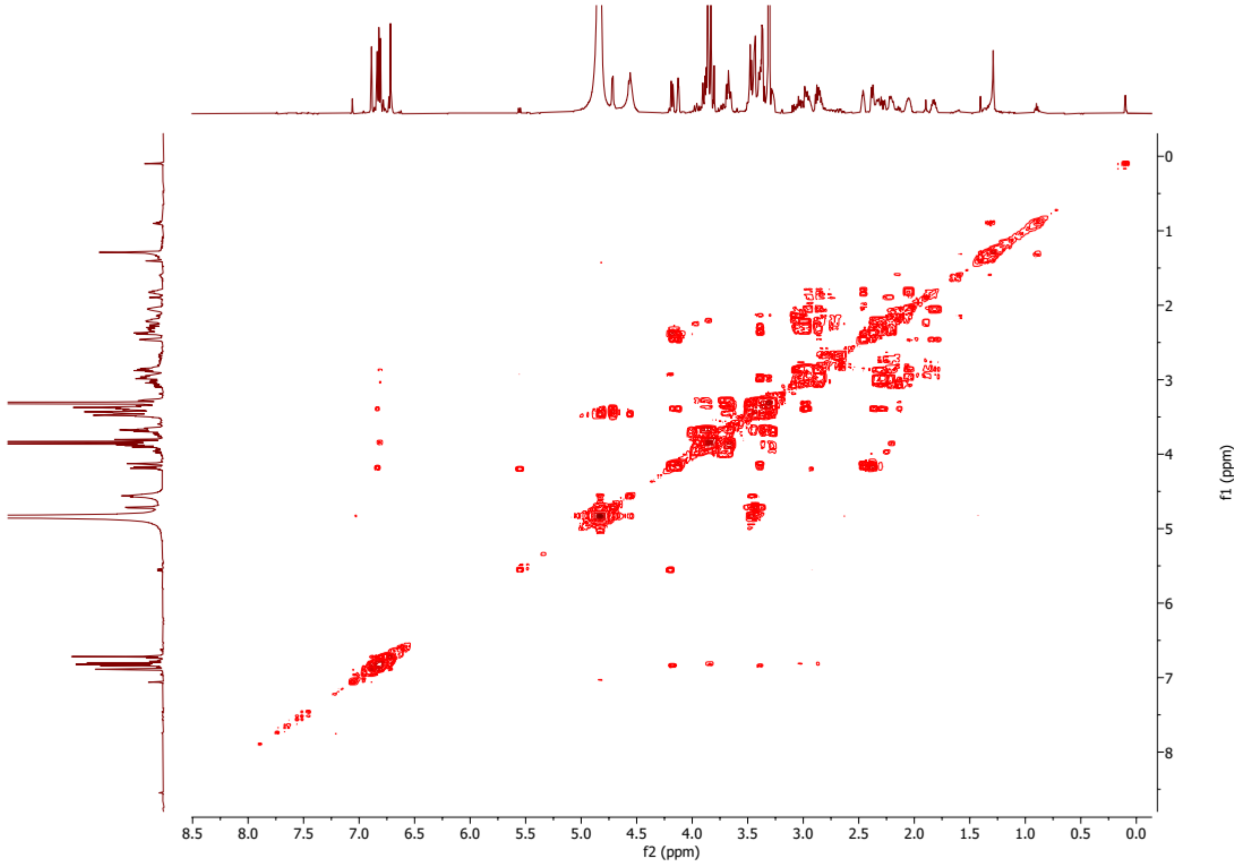

Figure S165. NOESY NMR spectrum of compound 17

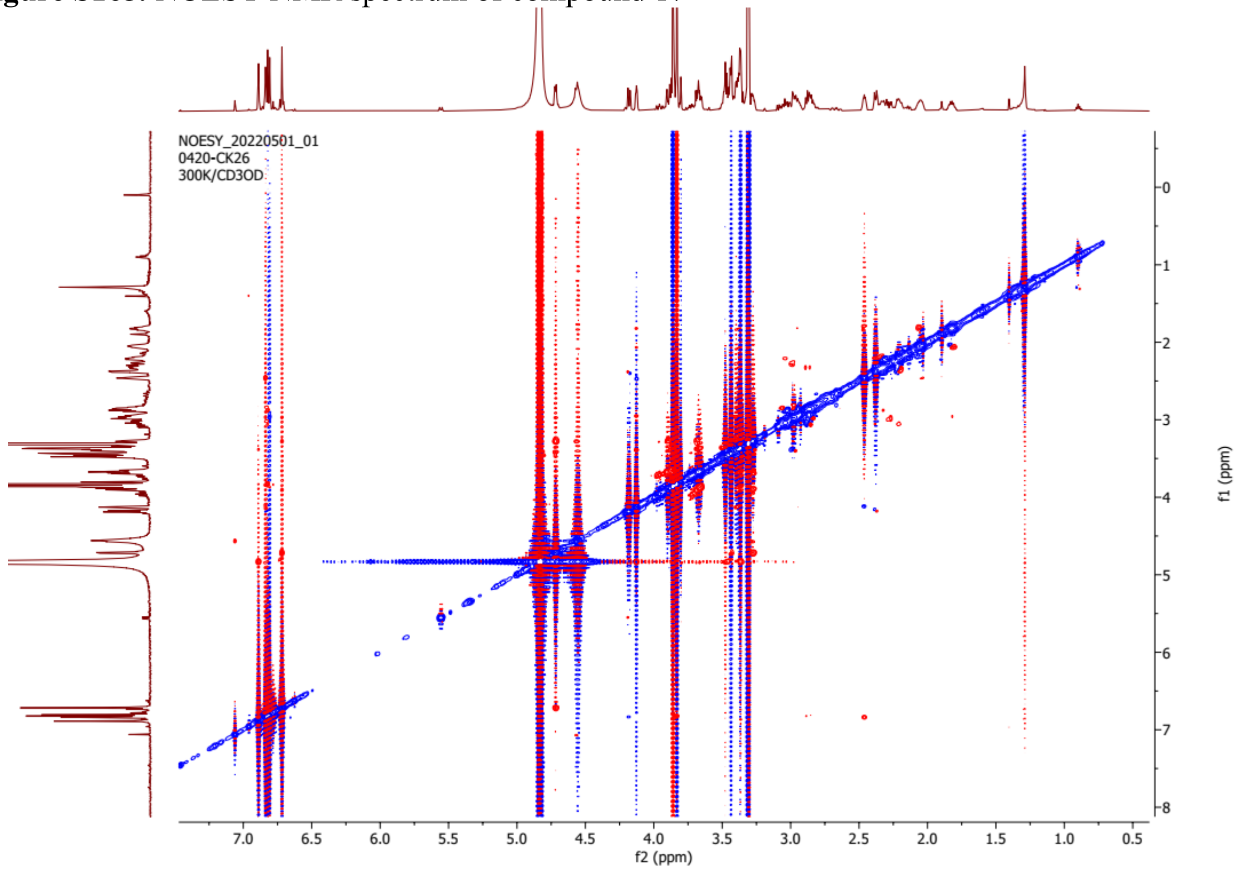

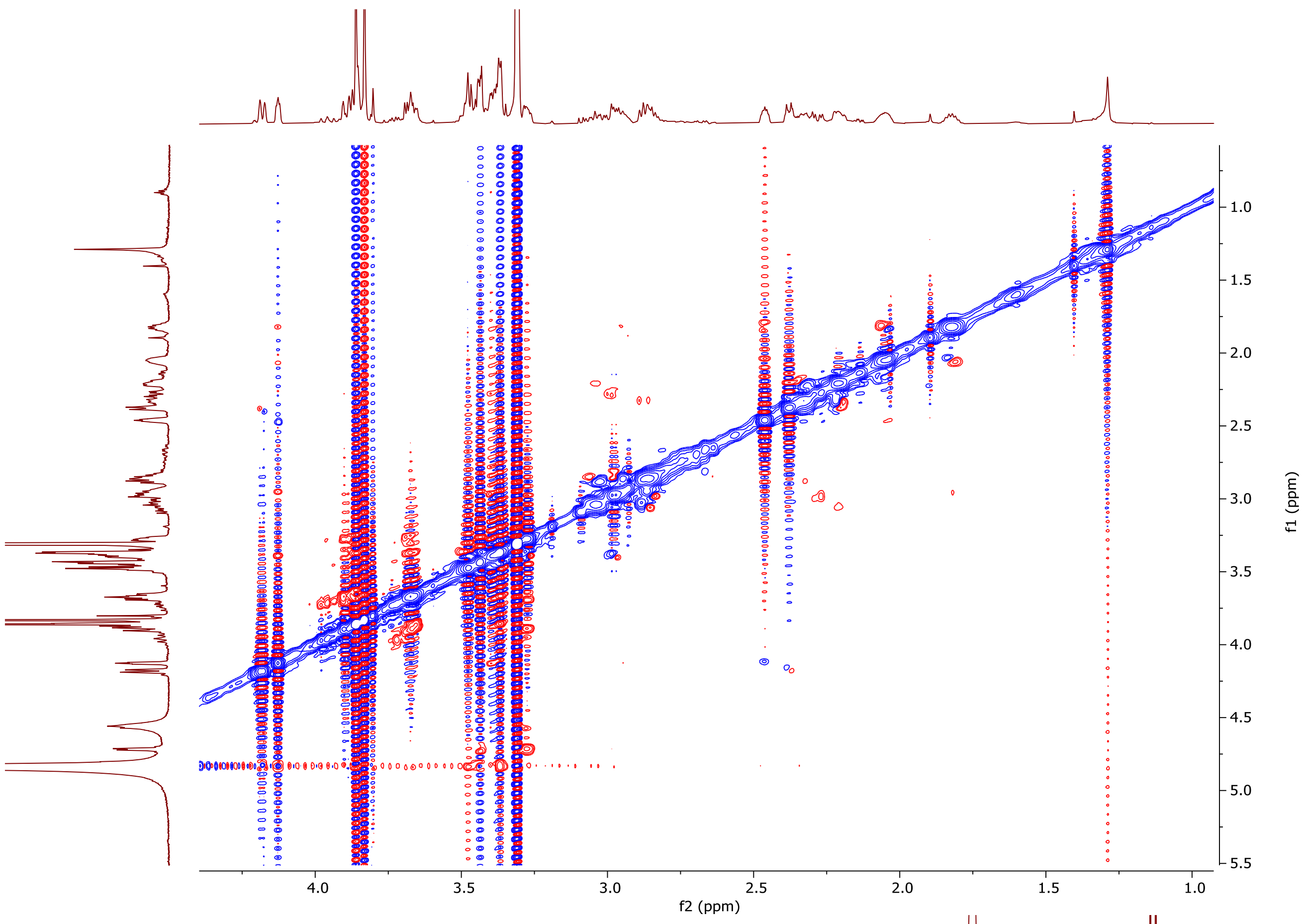
f2 (ppm)
f1 (ppm)

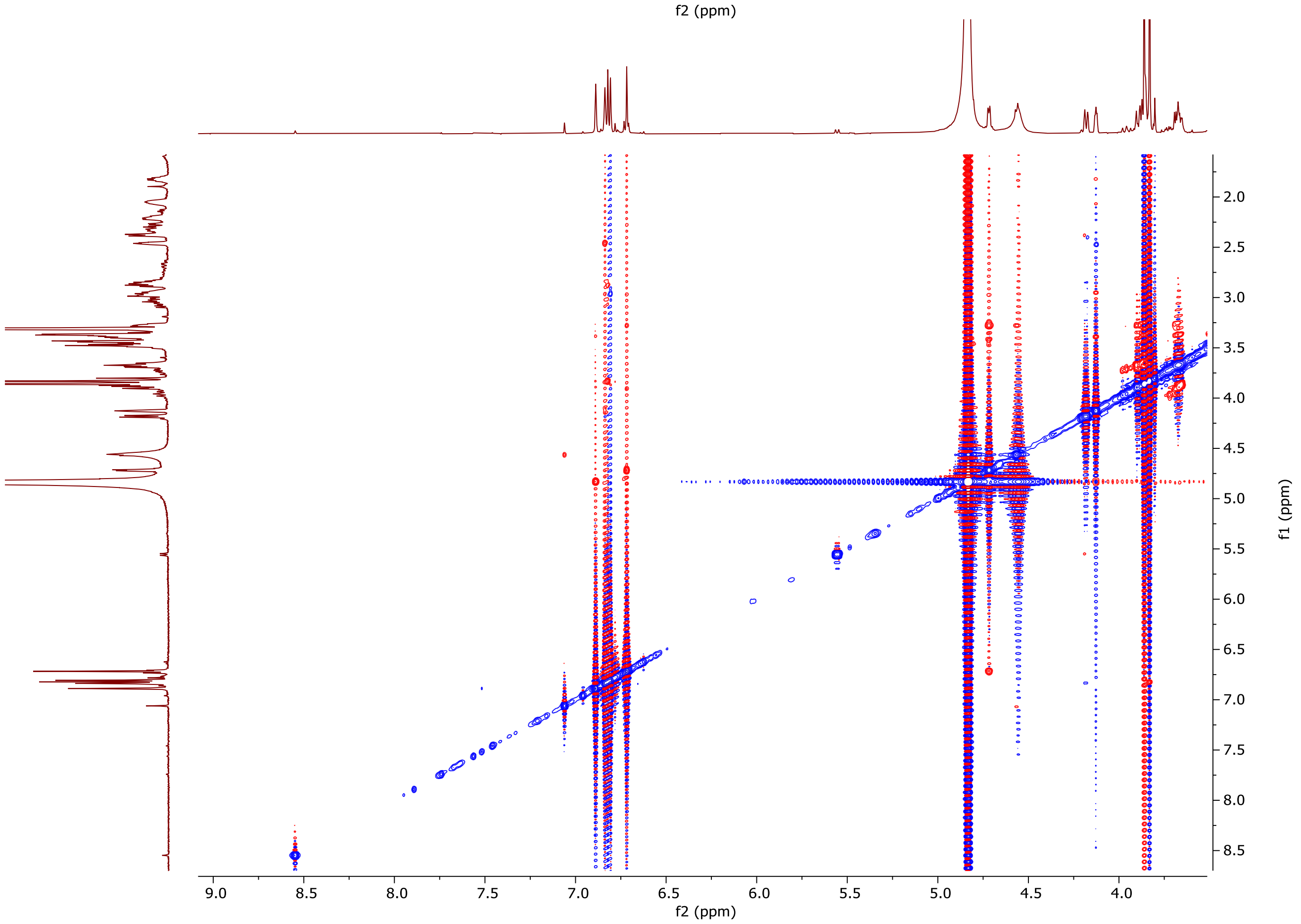
f2 (ppm)
f1 (ppm)

Figure S166. HR-ESI-MS spectrum of compound 17

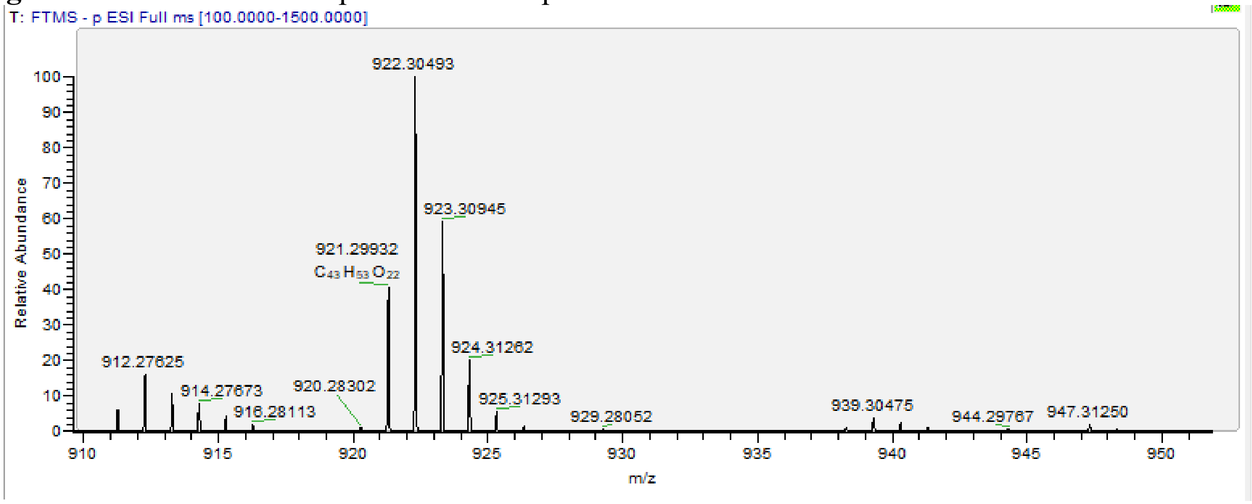

Idx	Formula	RDB	Delta ppm
1	$C_{43}H_{53}O_{22}$	17.5	-2.637

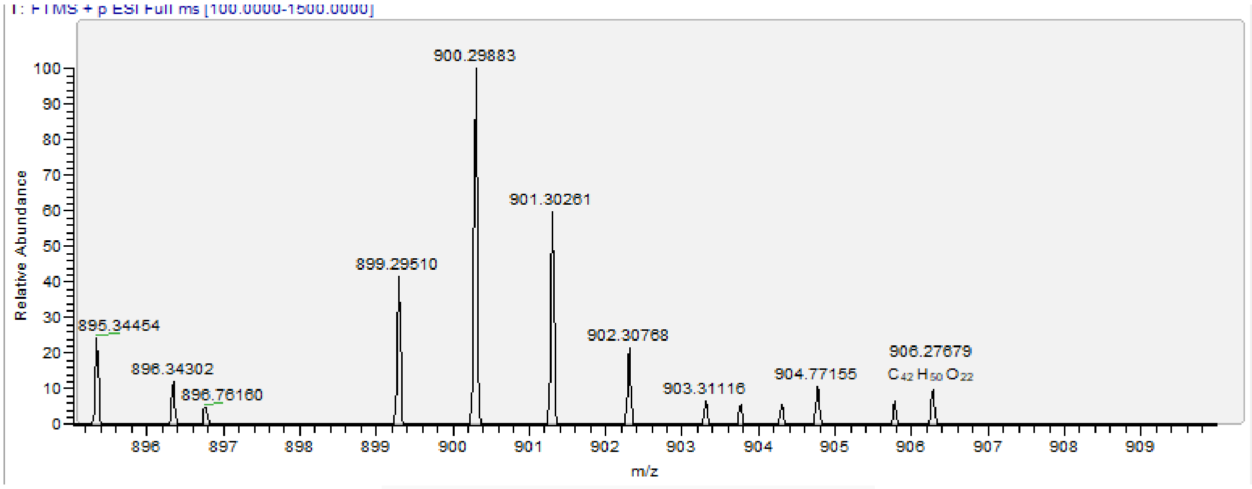

Idx	Formula	RDB	Delta ppm
1	$C_{42}H_{52}O_{20}Na$	16.5	0.762

Figure S167. UV spectrum of compound 17

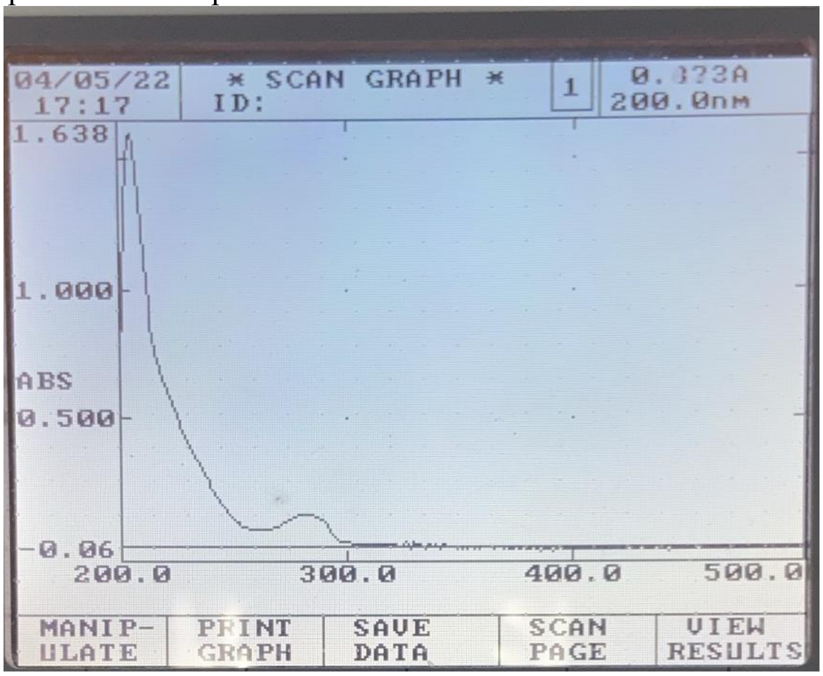

Figure S168. IR spectrum of compound 17

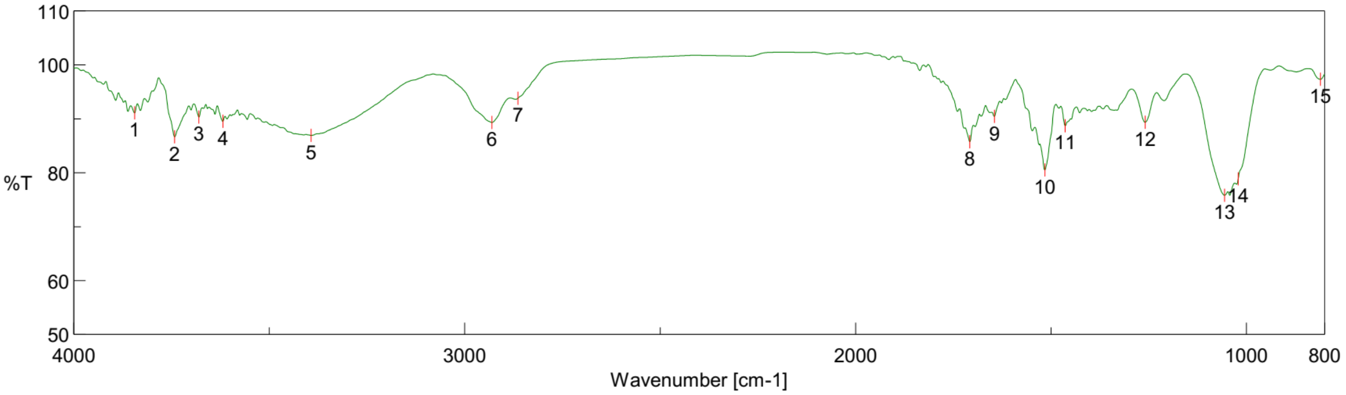

[Result of Peak Picking]

No.	Position	Intensity	No.	Position	Intensity	No.	Position	Intensity
1	3844.4	91.0863	2	3742.19	86.6656	3	3680.48	90.3082
4	3618.77	89.5177	5	3393.14	86.9081	6	2930.31	89.2929
7	2863.77	93.7957	8	1707.66	85.71	9	1644.98	90.4307
10	1515.78	80.4961	11	1463.71	88.6807	12	1259.29	89.337
13	1055.84	75.8144	14	1021.12	78.8332	15	810.92	97.3032
16	672.071	92.4948	17	427.155	78.0542	18	414.62	80.0236

Figure S169. Key NOESY correlation for distinguishing (4a*R*,10a*R*) form and (4a*S*,10a*S*) form of compound 5

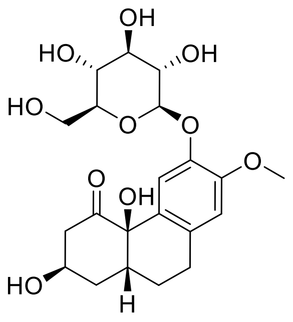

(2*R*,4a*R*,10a*R*)

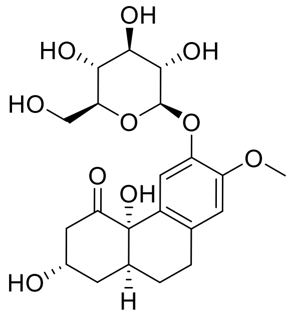

(2*S*,4a*S*,10a*S*)

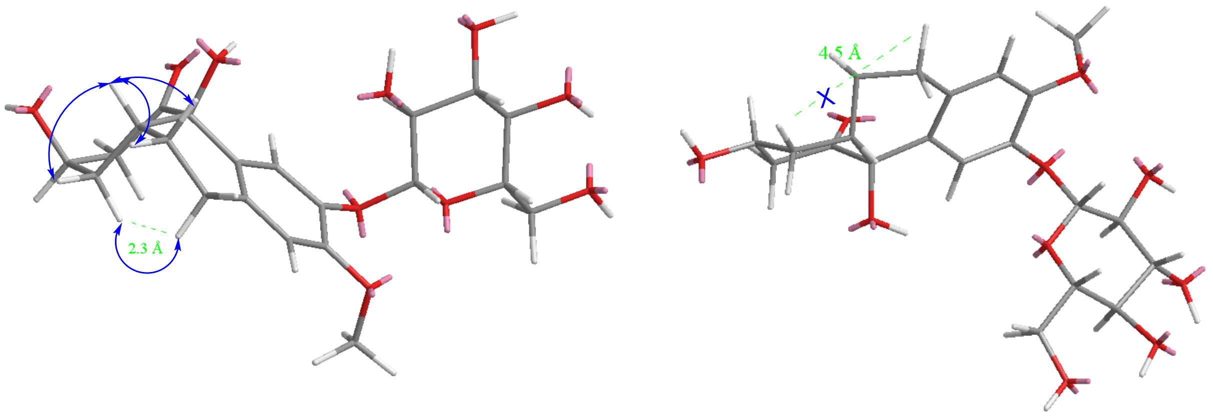

Figure S170. Key NOESY correlation for distinguishing (4a*R*,10a*R*) form and (4a*S*,10a*S*) form of compounds 11 – 12

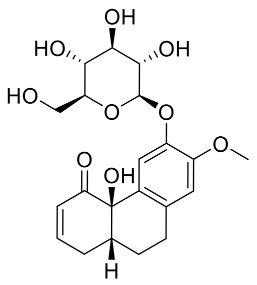

(4aR,10aR)

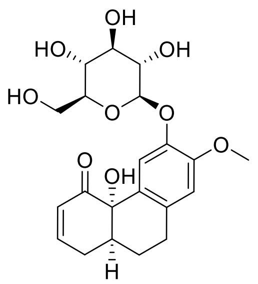

(4aS,10aS)

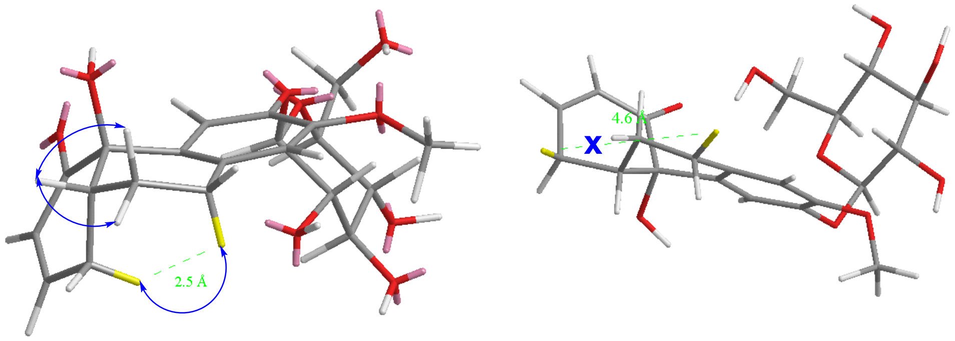

Compound 11

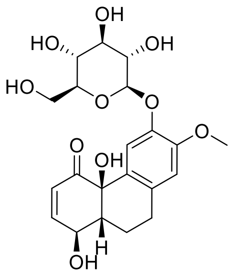

(1S,4aR,10aR)

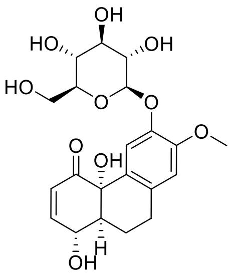

(1R,4aS,10aS)

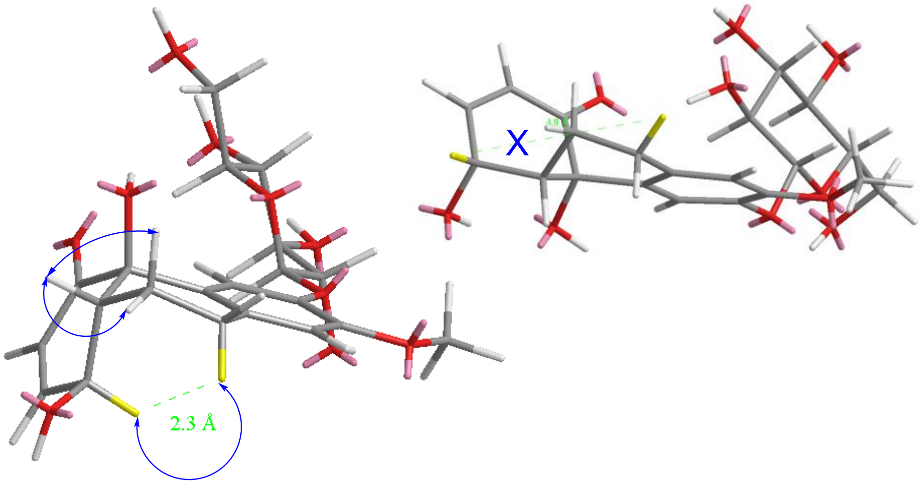

Compound 12

2. Materials and methods

2.1. General experimental procedures

Spectral data such as optical rotation, infrared spectroscopy, UV, and NMR were collected from a JASCO P-2000 polarimeter (Japan), a JASCO FT/IR 4100 spectrometer (Japan), a Thermo UV-Vis Helios Spectrophotometer (USA) and an Agilent DD2 600 MHz spectrometer (USA) or Bruker DRX-500 NMR spectrometer (Germany), respectively. Preparative HPLC was conducted using a Shimadzu HPLC-20AR system, while Hitachi L-7000 system was used for analytical experiments. MPLC was performed on the Isolera ONE system (Uppsala, Sweden) with a Biotage C18 or Biotage KP-SIL column. Additionally, precoated silica gel plates 60 F254 were purchased from Supleco (USA). Thermo Q-ExactiveTM Plus Hybrid Quadrupole Orbitrap (USA) was used to obtain high resolution mass spectrometry data. An ACQUITY UPLC BEH C18 column (2.1 × 100 mm, 1.7 µm; Waters) was utilized for confirming monosaccharides in hydrolysates. D-glucose and L-rhamnose standards were purchased from Sigma-Aldrich (US). A Polysaccharide Compositional Assay Kit (comprising A, B, and H reagents) was obtained from Sugar Light Co., Taiwan (R.O.C.). DPP-IV experiments and cell viability were assessed using a Varioskan LUX spectrophotometer (Thermo Fisher Scientific, Bremen, Germany).

2.2. Materials

The aerial parts of *E. tenuicaudatum* were harvested in June 2020 in Hoa Binh province, Vietnam. Their scientific names were verified by Dr. Vo Thanh Hoa – University of Health Sciences, Vietnam National University Ho Chi Minh City. The material voucher was deposited at the Pharmacognosy laboratory, Taipei Medical University. These materials were dried at $60^{o}C$ until their humanity reached below 12%.

2.3. UPLC MS/MS and Molecular network

3.3.1. UPLC MS/MS condition

We conducted analyzing sample on Thermo UPLC system with the mobile phase consisting of channel A (HCOOH 0.1%/MeOH) and channel B (HCOOH 0.1%/H_2O). The gradient condition was: 0 min (channel 5 %), 2 min (channel A 5%), 3 min (channel A 30%), 7 min (channel A 60%), 9 min (channel A 90%), 11 min (channel A 100%), and 13 min (channel A 100%) followed the flow rate at 0.3 ml/min. The electrospray ionization (ESI) parameters were configured as follows: spray voltages were used at -3.2 kV negative ion mode; the temperature values of capillary were maintained at 320°C. The mass spectrometer executed full scan cycles across the m/z range of 100-1500.

3.3.2. Molecular network

The raw data was firstly converted to .mzML file by MSConvert software[1] and processed by

Mzmine 4.2.0[1], followed by feature-based molecular networking (FBMN) analysis on the GNPS platform. [2,3] The parameter of FBMN was set up as: the Precursor and Fragment Ion Mass Tolerance were both 0.02 Da, the Min Pair Cos was 0.7 and Minimum Matched Fragment Ions was 4. Then, the MN was visualized by Cytoscape 3.9.1 software[4] and ClassyFire algorihm in Sirius 5.8.3 software was used to predict the chemical class of nodes in MN.[5,6]

The molecular networking data involved in this work are publicly available through GNPS repository via https://gnps.ucsd.edu/ProteoSAFe/status.jsp?task=580c7a563671449d8c95dbd1614c3ac7.

2.4. Extraction and Isolation

2.0 kg of dried *Elatostema tenuicaudatum* aerial parts were reflux extracted with 40 liters of ethanol 96% in 4 hours and followed by evaporating the solvent in *vacuo* to yield 117 g extract. This residue was diffused into water and partitioned with *n*-hexane. The water fraction was then coated with silica gel and applied onto MPLC with silica gel as stationary phase and *n*-hexan – ethyl acetate – methanol (gradient) as mobile phase to get eleven fractions (Fr.1 – Fr.11). Fractions and subfractions were subsequently analyzed using LC-MS/MS under identical conditions to construct the molecular network. Isolation efforts were then concentrated on the targeted clusters and compounds identified through the molecular network analysis.. Fraction Fr.8 (9.2 g) was purified o MPLC (C-18 column) by CH_3OH – H_2O (0:100 – 100:0) to acquire 10 subfractions Fr.8.1 -Fr.8.10. Fr.8.4 (834 mg) was then chromatographed on preparative HPLC (C-18 column, CH_3OH – H_2O (30:70), 6 ml/min) to obtain compound **1** (8.8 mg, 35 min) and compound **4** (40 mg, 82 min). Fraction Fr.5 (3.4 g) was loaded onto MPLC with C-18 column and conducted by CH_3OH – H_2O (10:90 – 40:60) as mobile phase to yield subfraction Fr.5.1 (84 mg), Fr.5.2 (300 mg) and compound **13** (1393 mg). Fr.5.2 was loaded onto preparative HPLC (C-18 column, CH_3CN 24%, 9 ml/min) to yield compound **14** (83 mg, 51 min). 1.7 g of Fr.7 was applied to preparative HPLC (C-18 column, CH_3OH – H_2O (30:70), 8 ml/min) to obtain compound **11** (218 mg, 25 min) and compound **9** (737 mg, 55 min). 2.5 g of fraction Fr.10 was treated with preparative HPLC using the C-18 column as stationary phase and CH_3OH – H_2O mobile phase condition (0 – 50 min: CH_3OH 18%, 50 – 120 min: CH_3OH 50%) to yield compound **12** (3.3 mg, 20 min), compound **6** (34 mg, 38 min), compound **5** (168 mg, 45 min), compound **2** (7 mg, 49 min) and compound **15** (704 mg, 95 min). Compound **7** (14 mg, 120 min) was isolated from Fr.8.2 (344 mg) by preparative HPLC (C-18 column, CH_3OH 25%, 8 ml/min). Compound **10** (50 mg, 65 min) and compound **3** (25 mg, 75 min) were separated from Fr.8.7 (128 mg) by preparative HPLC (C-18 column, 8 ml/min) and CH_3OH 40% as mobile phase. Fr.8.3 (1397 mg) was separated on preparative HPLC (C-18 column, CH_3OH 28% (0-50 min) – CH_3OH 48% (50 -100 min), 8 ml/min) to yield compound

17 (2.2 mg, 22 min), compound **8** (57 mg, 27 min), and compound **16** (61 mg, 70 min). These isolated spectroscopic data were shown in Figures S1 – S168.

Compound 1: white powder; $[\alpha]^{20}_{D}$ +133.6 (c 0.10, MeOH); UV (MeOH) λ_{max} nm 206, 226, 278; CD (MeOH) λ_{max} nm ($\Delta\varepsilon$) 240 (31.14), 280 (-6.37), 292 (0.84) and 309 (-2.12); HR-ESI-MS *m/z* 463.15732 $[M+Na]^+$ (Calcd. for $C_{21}H_{28}O_{10}Na$, 463.15802) and *m/z* 485.16531 $[M+HCOO]^-$ (Calcd. for $C_{22}H_{29}O_{12}$, 485.16591); IR data see Supplementary S10; 1D NMR data are presented in Table 1.

Compound 2: white, amorphous powder; $[\alpha]^{20}_{D}$ +107.6 (c 0.10, MeOH); UV (MeOH) λ_{max} nm 206, 226, 280; CD (MeOH) λ_{max} nm ($\Delta\varepsilon$) 238 (22.60), 280 (-4.04), 292 (0.35) and 309 (-2.52); HR-ESI-MS *m/z* 423.16458 $[M-H_2O+H]^+$ (calcd. for $C_{21}H_{27}O_9$, 423.16551), *m/z* (485.16525 $[M+HCOO]^-$, calcd. for $C_{22}H_{29}0_{12}$, 485.16591); IR data see Supplementary S20; 1D NMR NMR data are presented in Table 1.

Compound 3: white amorphous powder; $[\alpha]^{20}_{D}$ +123 (c 0.10, MeOH); UV (MeOH) λ_{max} nm 206, 226, 280; CD (MeOH) λ_{max} nm ($\Delta\varepsilon$) 238 (28.26), 280 (-3.73), 291 (-1.56) and 316 (-6.55); HR-ESI-MS *m/z* (491.18829 $[M+Na]^+$, calcd. for $C_{23}H_{32}O_{10}Na$, 491.18932), and *m/z* (513.19617 $[M+HCOO]^-$, 513.19720); IR data see Supplementary S30; 1D NMR data are presented in Table 1.

Compound 4: white amorphous powder; $[\alpha]^{20}_{D}$ +141.5 (c 0.10, MeOH); UV (MeOH) λ_{max} nm 206, 226, 280; CD (MeOH) λ_{max} nm ($\Delta\varepsilon$) 238 (24.03), 281 (-5.57), 292 (-2.07) and 306 (-5.06); HR-ESI-MS *m/z* 477.17300 $[M+Na]^+$, (Calcd. for $C_{22}H_{30}0_{10}Na$, 477.17367); *m/z* 499.18122 $[M+HCOO]^-$ (calcd. for $C_{23}H_{31}0_{12}$, 499.18156); IR data see Supplementary S39; 1D NMR data are presented in Table 1.

Compound 5: white amorphous powder, $[\alpha]^{20}_{D}$ +50.8 (c 0.10, MeOH); UV (MeOH) λ_{max} nm 206, 282; CD (MeOH) λ_{max} nm ($\Delta\varepsilon$) 246 (-49.50), 296 (66.3); HR-ESI-MS *m/z* 463.15714 ($[M+Na]^+$, Calcd. for $C_{21}H_{28}0_{10}Na$, 463.15802), *m/z* 423.16473 ($[M-H_2O+H]^+$, Calcd. for $C_{21}H_{27}0_9$, 423.16551), *m/z* 485.16513 ($[M+HCOO]^-$, Calcd. for $C_{22}H_{29}0_{12}$, 485.16591); IR data see Supplementary S49; 1D NMR data are presented in Table 2.

Compound 6: white amorphous powder; $[\alpha]^{20}_{D}$ +37.8 (c 0.10, MeOH); UV (MeOH) λ_{max} nm 206, 282; CD (MeOH) λ_{max} nm ($\Delta\varepsilon$) 246 (-37.23), 295 (76.47); HR-ESI-MS *m/z* 485.16522 ($[M+HCOO]^-$, Calcd. for $C_{22}H_{29}O_{12}$, 485.16591); IR data see Supplementary S59; 1D NMR data are presented in Table 2.

Compound 7: white amorphous powder; $[\alpha]^{20}_{D}$ -45.5 (c 0.10, MeOH); UV (MeOH) λ_{max} nm 206, 282; CD (MeOH) λ_{max} nm ($\Delta\varepsilon$) 246 (-44.70), 296 (59.30); HR-ESI-MS *m/z* 477.17307 ($[M+Na]^+$ (calcd. for $C_{22}H_{30}O_{10}Na$, 477.17367), *m/z* 499.18091 ($[M+HCOO]^-$, Calcd. for $C_{23}H_{31}O_{12}$, 499.18156); IR data see Supplementary S69; 1D NMR data are presented in Table 2.

Compound 8: white amorphous powder; $[\alpha]^{20}_{D}$ +21 (c 0.10, MeOH); UV (MeOH) λ_{max} nm 205, 223, 286; CD (MeOH) λ_{max} nm ($\Delta\varepsilon$) 242 (-12.97), 310 (20.86); HR-ESI-MS *m/z* 421.14903 [M-

$H_2O+H]^+$ (Calcd. for $C_{21}H_{25}0_9$, 421.14986), *m/z* 461.14154 $[M-H_2O+Na]^+$ (Calcd. for $C_{21}H_{26}0_{10}Na$, 461.14237); IR data see Supplementary S79; 1D NMR data are presented in Table 2.

Compound 9: white amorphous powder; $[\alpha]^{20}_D$ +286 (c 0.10, MeOH); UV (MeOH) λ_{max} nm 206, 226, 280; CD (MeOH) λ_{max} nm ($\Delta\varepsilon$) 254 (18.36), 343 (-15.78); HR-ESI-MS *m/z* 467.15457 $[M+HCOO]^-$ (Calcd. for $C_{22}H_{27}0_{11}$, 467.15534); IR data see Supplementary S89; 1D NMR data are presented in Table 3.

Compound 10: white amorphous powder; $[\alpha]^{20}_D$ +159.8 (c 0.10, MeOH); UV (MeOH) λ_{max} nm 206, 226, 280; CD (MeOH) λ_{max} nm ($\Delta\varepsilon$) 250 (39.42), 349 (-18.45); HR-ESI-MS *m/z* 459.16220 $[M+Na]^+$ (Calcd. for $C_{22}H_{28}O_9Na$, 459.16310) and *m/z* 481.17001 $[M+HCOO]^-$ (Calcd. for $C_{23}H_{29}O_{11}$, 481.17099); IR data see Supplementary S99; 1D NMR data are presented in Table 3.

Compound 11: white amorphous powder; $[\alpha]^{20}_D$ +40.3 (c 0.10, MeOH); UV (MeOH) λ_{max} nm 206, 226, 280; CD (MeOH) λ_{max} nm ($\Delta\varepsilon$) 258 (-26.38), 334 (16.95); HR-ESI-MS *m/z* 467.15457 $[M+HCOO]^-$ (Calcd. for $C_{22}H_{27}O_{11}$, 467.15534); IR data see Supplementary S109; 1D NMR data are presented in Table 3.

Compound 12: white amorphous powder; $[\alpha]^{20}_D$ +50.8 (c 0.10, MeOH); UV (MeOH) λ_{max} nm 206, 226, 280; CD (MeOH) λ_{max} nm ($\Delta\varepsilon$) 244 (-43.85), 292 (15.87); HR-ESI-MS *m/z* 461.14108 $[M+Na]^+$, (Calcd. for $C_{21}H_{26}O_{10}Na$, 461.14237), *m/z* 483.14954 $[M+HCOO]^-$ (Calcd. for $C_{22}H_{27}O_{12}$, 483.15026); IR data see Supplementary S119; 1D NMR data are presented in Table 3.

Compound 13: white amorphous powder; $[\alpha]^{20}_D$ -36.8 (c 0.10, MeOH); UV (MeOH) λ_{max} nm 220, 275, 300; HR-ESI-MS *m/z* 427.13583 $M+Na]^+$ (Calcd. for $C_{21}H_{24}O_8Na$, 427.13689), *m/z* 403.13852 ($[M-H]^-$, Calcd. for $C_{21}H_{23}O_8$, 403.13930), *m/z* 449.14407 $[M+HCOO]^-$ (Calcd. for $C_{22}H_{25}O_{10}$, 449.14478); IR data see Supplementary S129; 1D NMR data are presented in Table 4.

Compound 14: white amorphous powder; $[\alpha]^{20}_D$ -30.6 (c 0.10, MeOH); UV (MeOH) λ_{max} nm 220, 275, 300; HR-ESI-MS *m/z* 551.21246 ($[M+H]^+$, Calcd. for $C_{27}H_{35}O_{12}$, 551.21286), *m/z* 573.18421 $[M+Na]^+$ (Calcd. for $C_{27}H_{34}O_{12}Na$, 573.19480), *m/z* 549.19611 $[M-H]^-$ (Calcd. for $C_{27}H_{33}O_{12}$, 549.19720); IR data see Supplementary S138; 1D NMR data are presented in Table 4.

Compound 15: white amorphous powder; $[\alpha]^{20}_D$ -28.8 (c 0.10, MeOH); UV (MeOH) λ_{max} nm 220, 275, 300; HR-ESI-MS *m/z* 391.13830 ($[M+H]^+$, Calcd. for $C_{20}H_{23}O_8$, 391.13930), *m/z* 413.12048 $[M+Na]^+$ (Calcd. for $C_{20}H_{22}O_8Na$, 413.12124), *m/z* 389.12322 $[M-H]^-$ (Calcd. for $C_{20}H_{21}O_8$, 389.12365); IR data see Supplementary S148; 1D NMR data are presented in Table 4.

Compound 16: white amorphous powder; $[\alpha]^{20}_D$ +38.8 (c 0.10, MeOH); UV (MeOH) λ_{max} nm 206, 226, 282; HR-ESI-MS *m/z* 867.30370 $[M+Na]^+$ (Calcd. for $C_{42}H_{52}O_{18}Na$, 867.30514), *m/z* 889.31169 $[M+HCOO]^-$ (Calcd. for $C_{43}H_{53}0_{20}$, 889.31302); IR data see Supplementary S158; 1D NMR data are presented in Table 5.

Compound 17: white amorphous powder; $[\alpha]^{20}_{D}$ +10.8 (c 0.10, MeOH); UV (MeOH) λ_{max} nm 206, 282; HR-ESI-MS *m/z* 899.29510 $[M+Na]^+$ (Calcd. for $C_{42}H_{52}O_{20}Na$, 899.29497), *m/z* 921.29932 $[M+HCOO]^-$ (Calcd. for $C_{43}H_{53}0_{22}$, 921.30285); IR data see Supplementary S168; 1D NMR data are presented in Table 5.

2.5. ECD and DP4+ calculation

The structures of the stereoisomers were initially determined using ChemBio 3D with the MM2 force field. Subsequently, conformers were generated and preliminarily optimized using SPARTAN 14. onformers within a 3.5 kcal/mol window were then optimized, and their frequencies calculated with Gaussian 16W at the B3LYP/6-31G(d,p) level in the gas phase. Conformers without imaginary frequencies were further calculated for ECD spectra using the time-dependent DFT (TDDFT) method at the B3LYP/6-311G(d,p) or CAM- B3LYP/6-311G(d,p) level in methanol.[7] The ECD spectra were rendered using Specdis, based on a Boltzmann-weighted average distribution.[8] NMR calculations were performed using the GIAO method combined with time-dependent density functional theory (TDDFT) at the mPW1PW91/6-311+G(d,p) level in methanol. The resulting NMR data were weighted based on the Boltzmann distribution and analyzed using the DP4+ method.[15]

2.6. Determination of sugar by hydrolysis

The sugar analysis was conducted by applying an assay kit for polysaccharide based on the reference method.[9] 0.5 mL of hydrolysis reagent was used for hydrolyzing the samples (1.0 – 2.0 mg) at 80°C for 60 minutes. The reaction product was dried *in vacuo*. Subsequently, adding 1.5 mg of A (naphthimidazole) and 1.0 mL of B (iodine) reagent into vial that contained the dry product, and stirring for 60 minutes to make reaction to yield a product solution that was dried down after that. The residue was diffused into 1.0 mL of dd-water and centrifugated at 3000 rpm for 20 minutes. The supernatant was then filtered through a 0.22 μm membrane before analysis by LC-MS. For standards, sugar standards were prepared similarly without undergoing hydrolysis. The UPLC-MS mobile phase contained channel A (HCOOH 0.1%/CH_3CN) and channel B HCOOH 0.1%/H_2O. Mobile phase condition: 0 min (channel A 7%), 6 min (channel A 7%), 8 min (channel A 13%), 10 min (channel A 18%), 10.5 min (channel A 50%), 12 min (channel A 50%), 12.1 min (channel A 7%) and 17 min (channel A 7%). The flow rate at 0.3 μL/min and injected volume at 10.0 μL were used in this method. For the mass spectrometry (MS) method, an electrospray interface controlled by Xcalibur software operated in both positive and negative ion modes. The electrospray ionization (ESI) parameters were configured as follows: spray voltages were used at 3.5 kV for the positive mode; the temperature values of capillary and source heater were maintained at 360°C and 350°C, respectively. The mass spectrometer executed at the SIM mode (m/z 319 for D-glucose-NAIM and m/z 303 for L-rhamnose-

NAIM).

2.7. DPP-IV inhibition activity

A commercially available DPP-IV Activity Assay Kit (K779-100, BioVision) was employed to evaluate DPP-IV activity. The procedures were conducted according to the manufacturer's instructions. DPP-IV enzymatically cleaves substrates, leading to the release of the quenched fluorescent group AMC (7-Amino-4-Methyl Coumarin). AMC detection was measured with a fluorescence reader at Ex/Em = 360/460 nm. In this study, sitagliptin was used as an positive control.

2.8. Molecular docking

To investigate the binding affinity between DPP-IV and compounds, we utilized the CDOCKER Receptor-Ligand Interactions protocol in Discovery Studio software (DS 2021, Accelrys Software Inc., USA). The crystal structure of DPP-IV (PDB ID: 4FFW) was obtained from the Protein Data Bank (https://www.rcsb.org/pdb/); this protein originates from the species *Mus musculus*. The resolution of the Crystal structure deposited in the PDB is 2.90 Å.

The DPP-IV protein[10] was prepared by removing water molecules, adding hydrogen atoms and removing the original ligands. A specialized module was used to model any missing loop regions within the protein structure, the steps included calculating protein ionization, protonating the protein, and conducting a final energy minimization with CHARMm force field for molecular docking. The 2D structures of potential inhibitory DPP-IV were created using ChemBioDraw Ultra 13.3 and subsequently converted to standard 3D formats in DS 2021. These 3D structures then underwent energy minimization using the CHARMm force field and the conjugate gradient method, with a convergence criterion of 0.001 kcal/mol.[11] The energy-minimized structures were then used in molecular docking experiments.

The binding site was defined using PDB site records and the edit binding site module, with the binding site sphere (x: 52.3289, y: -32.7775, z: 23.7011, radius 26) chosen for analysis. After energy minimization, the DPP-IV-inhibited compounds were docked into the binding site of 4FFW using the CDOCKER program in DS 2021. The pose with the lowest binding free energy was selected as the optimal candidate for potential DPP-IV inhibitors. The ligand-binding free energy of the complex was then calculated using the Molecular Mechanics-Generalized Born with Molecular Volume (MM/GBMV) method.[11] Finally, the pose with the lowest ΔG (binding free energy) for each compound was selected as the most appropriate conformation for further analysis of its interactions with key residues in the proteins.

2.9. Hepatoprotective effects against acetaminophen-induced hepatotoxicity in the HepG2 cell model

3.9.1. Screening cell viability of compounds on HepG2

Human hepatocellular carcinoma HepG2 cells were obtained from the Bioresource Collection and Research Center (BCRC, Hsinchu, Taiwan). Cells culture was conducted with a complete medium consisting of DMEM (Dulbecco's Modified Eagle Medium, Gibco), 10% (v/v) fetal bovine serum (FBS) (Gibco), and 1% (v/v) antibiotic-antimycotic BSA (Gibco, 15240062). Upon reaching at 80% confluence, cells were rinsed with PBS and detached using trypsin-EDTA (Gibco, 25200072) at 37^oC for 5 minutes. Subsequently, cells were seeded into specialized 96-well plates (3×10^4/well, 100 μL/well) and incubated for 24 hours at 37^oC with 5% CO_2. Afterward, 100 µL of fresh medium containing the test samples was added to replace the previous medium. Following a 48-hour incubation, cell viability was assessed using the MTT assay, following established protocols.[12,13] Untreated or control cells were used as a reference under the same conditions. The maximum absorbance value depended on the solvent used in the sample solution, and the percentage viability of cells was determined using the formula: % viability = (total cells – viable cells)/total cells × 100. The compounds were screened at a concentration of 100 μM.

3.9.2. Assess hepatoprotective effects of compounds

Compounds exhibiting cell viability above 80% were further assessed for their hepatoprotective effects using an acetaminophen-induced hepatotoxicity HepG2 model. Cells were initially seeded in 96-well plates at a density of 3×10^4 cells/well and then incubated under identical conditions with 5% CO_2 for 24 hours. Following this, cells were exposed to the test samples. After 8 hours, hepatotoxicity was induced using acetaminophen (Sigma Aldrich, A7085) at a concentration of 6 mM. The protective effects were assessed by determining cell viability using the MTT method 36 hours post-acetaminophen induction. Silymarin (at concentration of 50 µg/mL) was used as positive control.

Reference

(1) Holman, J. D.; Tabb, D. L.; Mallick, P. Employing ProteoWizard to Convert Raw Mass Spectrometry Data. *Curr Protoc Bioinformatics* **2014**, No. SUPPL.46. https://doi.org/10.1002/0471250953.bi1324s46.

(2) Nothias, L. F.; Petras, D.; Schmid, R.; Dührkop, K.; Rainer, J.; Sarvepalli, A.; Protsyuk, I.; Ernst, M.; Tsugawa, H.; Fleischauer, M.; Aicheler, F.; Aksenov, A. A.; Alka, O.; Allard, P. M.; Barsch, A.; Cachet, X.; Caraballo-Rodriguez, A. M.; Da Silva, R. R.; Dang, T.; Garg, N.; Gauglitz, J. M.; Gurevich, A.; Isaac, G.; Jarmusch, A. K.; Kameník, Z.; Kang, K. Bin; Kessler, N.; Koester, I.; Korf, A.; Le Gouellec, A.; Ludwig, M.; Martin H, C.; McCall, L. I.; McSayles, J.; Meyer, S. W.; Mohimani, H.; Morsy, M.; Moyne, O.; Neumann, S.; Neuweger, H.; Nguyen, N. H.; Nothias-Esposito, M.; Paolini, J.; Phelan, V. V.; Pluskal, T.; Quinn, R. A.; Rogers, S.; Shrestha, B.; Tripathi, A.; van der Hooft, J. J. J.; Vargas, F.; Weldon, K. C.; Witting, M.; Yang, H.; Zhang, Z.; Zubeil, F.; Kohlbacher, O.; Böcker, S.; Alexandrov, T.; Bandeira, N.; Wang, M.; Dorrestein, P. C. Feature-Based Molecular Networking in the GNPS Analysis Environment. *Nat Methods* **2020**, *17* (9). https://doi.org/10.1038/s41592-020-0933-6.

(3) Wang, M.; Carver, J. J.; Phelan, V. V.; Sanchez, L. M.; Garg, N.; Peng, Y.; Nguyen, D. D.; Watrous, J.; Kapono, C. A.; Luzzatto-Knaan, T.; Porto, C.; Bouslimani, A.; Melnik, A. V.; Meehan, M. J.; Liu, W. T.; Crüsemann, M.; Boudreau, P. D.; Esquenazi, E.; Sandoval-Calderón, M.; Kersten, R. D.; Pace, L. A.; Quinn, R. A.; Duncan, K. R.; Hsu, C. C.; Floros, D. J.; Gavilan, R. G.; Kleigrewe, K.; Northen, T.; Dutton, R. J.; Parrot, D.; Carlson, E. E.; Aigle, B.; Michelsen, C. F.; Jelsbak, L.; Sohlenkamp, C.; Pevzner, P.; Edlund, A.; McLean, J.; Piel, J.; Murphy, B. T.; Gerwick, L.; Liaw, C. C.; Yang, Y. L.; Humpf, H. U.; Maansson, M.; Keyzers, R. A.; Sims, A. C.; Johnson, A. R.;

Sidebottom, A. M.; Sedio, B. E.; Klitgaard, A.; Larson, C. B.; Boya, C. A. P.; Torres-Mendoza, D.; Gonzalez, D. J.; Silva, D. B.; Marques, L. M.; Demarque, D. P.; Pociute, E.; O'Neill, E. C.; Briand, E.; Helfrich, E. J. N.; Granatosky, E. A.; Glukhov, E.; Ryffel, F.; Houson, H.; Mohimani, H.; Kharbush, J. J.; Zeng, Y.; Vorholt, J. A.; Kurita, K. L.; Charusanti, P.; McPhail, K. L.; Nielsen, K. F.; Vuong, L.; Elfeki, M.; Traxler, M. F.; Engene, N.; Koyama, N.; Vining, O. B.; Baric, R.; Silva, R. R.; Mascuch, S. J.; Tomasi, S.; Jenkins, S.; Macherla, V.; Hoffman, T.; Agarwal, V.; Williams, P. G.; Dai, J.; Neupane, R.; Gurr, J.; Rodríguez, A. M. C.; Lamsa, A.; Zhang, C.; Dorrestein, K.; Duggan, B. M.; Almaliti, J.; Allard, P. M.; Phapale, P.; Nothias, L. F.; Alexandrov, T.; Litaudon, M.; Wolfender, J. L.; Kyle, J. E.; Metz, T. O.; Peryea, T.; Nguyen, D. T.; VanLeer, D.; Shinn, P.; Jadhav, A.; Müller, R.; Waters, K. M.; Shi, W.; Liu, X.; Zhang, L.; Knight, R.; Jensen, P. R.; Palsson, B.; Pogliano, K.; Linington, R. G.; Gutiérrez, M.; Lopes, N. P.; Gerwick, W. H.; Moore, B. S.; Dorrestein, P. C.; Bandeira, N. Sharing and Community Curation of Mass Spectrometry Data with Global Natural Products Social Molecular Networking. *Nature Biotechnology*. 2016. https://doi.org/10.1038/nbt.3597.

(4) Shannon, P.; Markiel, A.; Ozier, O.; Baliga, N. S.; Wang, J. T.; Ramage, D.; Amin, N.; Schwikowski, B.; Ideker, T. Cytoscape: A Software Environment for Integrated Models of Biomolecular Interaction Networks. *Genome Res* **2003**, *13* (11). https://doi.org/10.1101/gr.1239303.

(5) Djoumbou Feunang, Y.; Eisner, R.; Knox, C.; Chepelev, L.; Hastings, J.; Owen, G.; Fahy, E.; Steinbeck, C.; Subramanian, S.; Bolton, E.; Greiner, R.; Wishart, D. S. ClassyFire: Automated Chemical Classification with a Comprehensive, Computable Taxonomy. *J Cheminform* **2016**, *8* (1). https://doi.org/10.1186/s13321-016-0174-y.

(6) Dührkop, K.; Fleischauer, M.; Ludwig, M.; Aksenov, A. A.; Melnik, A. V.; Meusel, M.; Dorrestein, P. C.; Rousu, J.; Böcker, S. SIRIUS 4: A Rapid Tool for Turning Tandem Mass Spectra into Metabolite Structure Information. *Nat Methods* **2019**, *16* (4). https://doi.org/10.1038/s41592-019-0344-8.

(7) Luo, Q.; Tu, Z. C.; Cheng, Y. X. Two Rare Meroterpenoidal Rotamers from Ganoderma Applanatum. *RSC Adv* **2017**, *7* (6). https://doi.org/10.1039/C6RA26572C.

(8) Bruhn, T.; Schaumlöffel, A.; Hemberger, Y.; Bringmann, G. SpecDis: Quantifying the Comparison of Calculated and Experimental Electronic Circular Dichroism Spectra. *Chirality* **2013**, *25* (4). https://doi.org/10.1002/chir.22138.

(9) Lin, C.; Hung, W. T.; Kuo, C. Y.; Liao, K. S.; Liu, Y. C.; Yang, W. Bin. I2-Catalyzed Oxidative Condensation of Aldoses with Diamines: Synthesis of Aldo-Naphthimidazoles for Carbohydrate Analysis. *Molecules* **2010**, *15* (3). https://doi.org/10.3390/molecules15031340.

(10) Tang, J.; Majeti, J.; Sudom, A.; Xiong, Y.; Lu, M.; Liu, Q.; Higbee, J.; Zhang, Y.; Wang, Y.; Wang, W.; Cao, P.; Xia, Z.; Johnstone, S.; Min, X.; Yang, X.; Shao, H.; Yu, T.; Sharkov, N.; Walker, N.; Tu, H.; Shen, W.; Wang, Z. An Inhibitory Antibody against Dipeptidyl Peptidase IV Improves Glucose Tolerance in Vivo. *Journal of Biological Chemistry* **2013**, *288* (2). https://doi.org/10.1074/jbc.M112.396317.

(11) Lee, M. S.; Feig, M.; Salsbury, F. R.; Brooks, C. L. New Analytic Approximation to the Standard Molecular Volume Definition and Its Application to Generalized Born Calculations. *J Comput Chem* **2003**, *24* (11). https://doi.org/10.1002/jcc.10272.

(12) Siddiqui, M. A.; Singh, G.; Kashyap, M. P.; Khanna, V. K.; Yadav, S.; Chandra, D.; Pant, A. B. Influence of Cytotoxic Doses of 4-Hydroxynonenal on Selected Neurotransmitter Receptors in PC-12 Cells. *Toxicology in Vitro* **2008**, *22* (7). https://doi.org/10.1016/j.tiv.2008.07.001.

(13) Mosmann, T. Rapid Colorimetric Assay for Cellular Growth and Survival: Application to Proliferation and Cytotoxicity Assays. *J Immunol Methods* **1983**, *65* (1–2). https://doi.org/10.1016/0022-1759(83)90303-4.

(14) Dao Duc Thien Tran Duc Dai, N. H. S.; Tam, N. T. New Dihydrophenanthrenes from Elatostema Tenuicaudatum. Nat Prod Res **2024**, 0 (0), 1–7. https://doi.org/10.1080/14786419.2024.2359543.

(15) Grimblat, N., Zanardi, M. M., & Sarotti, A. M. Beyond DP4: An improved probability for the stereochemical assignment of isomeric compounds using quantum chemical calculations of NMR shifts. *The Journal of organic chemistry* **2015**, *80*(24), 12526-12534. https://doi.org/10.1021/acs.joc.5b02396.

3. Elucidate the structure of compound 13, 14

Compound **13** was obtained as white amorphous powder. Its molecular formula was $C_{21}H_{24}O_8$ which was deduced based on HRESIMS data (*m/z* 403.13852 [M-H]$^-$, Calcd. for $C_{21}H_{23}O_8$, *m/z* 427.13583 [M+Na]$^+$, Calcd. for $C_{21}H_{24}O_8Na$). Its IR spectrum showed absorption bands consistent with the presence of hydroxy (3845 cm^{-1}). On the UV spectrum, compound **13** showed the absorption maxima at 220, 275, and 300 nm. According to ^{1}H-NMR spectrum, three olefinic protons at δ_H 6.90 (1H, dd, 8.0, 1.3 H-1), δ_H 7.14 (1H, t, 8.0, H-2), δ_H 7.19 (1H, dd, 8.0, 1.3 H-3); two olefinic protons at δ_H 9.30 (1H, s, H-5) and δ_H 6.94 (1H, s, H-8); protons signal at δ_H 3.79 (3H, s) and an anomeric proton at δ_H 5.81 (1H, d, 7.4, H-1´) suggested that compound **13** may contain one ABC system benzene ring, one *para* - tetrasubstituted benzene ring, one methoxy group and one β-orientated sugar, respectively. Combined with DEPT and HSQC data, 21 carbon signals in the ^{13}C-NMR spectrum were assigned as a glucopyranosyl unit (δ_C 102.8, 79.1, 79.0, 74.7, 71.3, 62.6), two methylenes (δ_C 30.2, 31.4), five methines (δ_C 128.0, 120.1, 118.7, 116.5, 112.9), one methoxy carbon (δ_C 56.5), and seven quaternary carbons (δ_C 156.4, 148.9, 146.3, 140.6, 133.4, 127.5, 122.9). HMBC NMR spectrum showed the correlation of the anomeric proton H-1´ with C-6 and the correlation between the methoxy group and C-7 to confirm the position of the sugar and methoxy to *para* tetrasubstituted benzene ring. In addition, HMBC correlations of H-5/C-4a; H-8/C-8a, C-9; H-2/C-4 confirmed the position of C-4, C-5, C-8, C-4a, C-5a, C-8a and C-9. Furthermore, based on the HMBC correlation of H-1´/C-6; 7-OMe/C-7 and NOESY correlation of H-1´/H-5, the positions of C-6, and C-7 were determined. The aliphatic signal connection of H-9/H-10 and H-1´/H-2´/H-3´/H-4´/H-5´/H-6´ was established according to COSY spectrum (Figure S169). Besides, the J value of H-1´ is 7.4 Hz, suggesting the β orientation of the H-1´ anomer. From the above analysis, the structure of compound **13** was depicted as in Figure 1. This compound was isolated in the same plant[14] but had not been named, therefore we named it elatostemanoside M.

Compound **14**, obtain as white amorphous powder, molecular formular of $C_{20}H_{22}O_8$ on the basis of HRESIMS data (*m/z* 391.13830 [M+H]$^+$, Calcd. for $C_{20}H_{23}O_8$, *m/z* 413.12048 [M+Na]$^+$, Calcd. for $C_{20}H_{22}O_8Na$, *m/z* 389.12322 [M-H]$^-$ Calcd. for $C_{20}H_{21}O_8$) and ^{13}C-NMR data. Compound **14** also showed a CH_2 group less than compound **13**, that suggested **14** is a derivative of **13**. By analysis ^{1}H-NMR, ^{13}C-NMR and DEPT spectra, one methoxy group disappeared when compared to **13**. Combining 1D, 2D NMR data and a similarly analysis as **13** (Figure S169), the structure of compound **14** was deduced as shown in Figure 1. This compound was isolated in the same plant[14] but had not been named, therefore we named it elatostemanoside N.

4. ECD calculation data of compound 1

Figure S171. The energies and equilibrium populations of stable conformers 1_1 to 1_5

No	Conformer	Structure	Gibb free energy (Hartree)	Population (%)
1	1_1	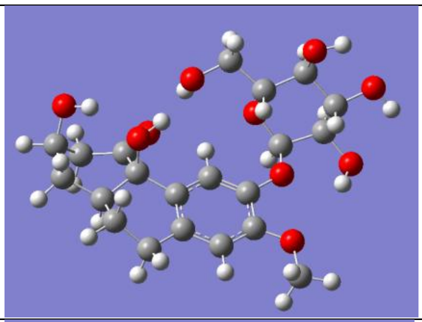	-1568.896667	2.56
2	1_2	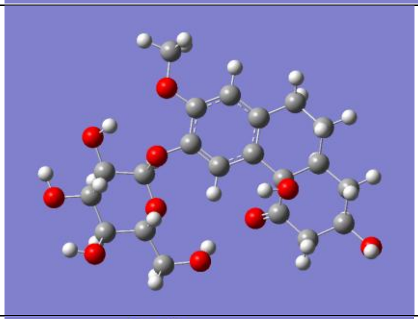	-1568.899213	38.03
3	1_3	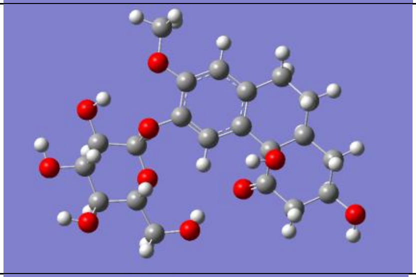	-1568.898926	28.07
4	1_4	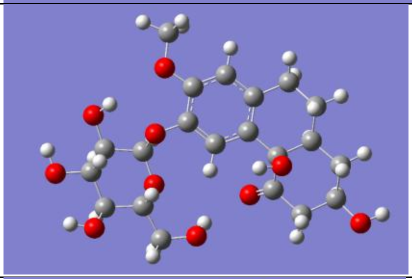	-1568.898524	18.34
5	1_5	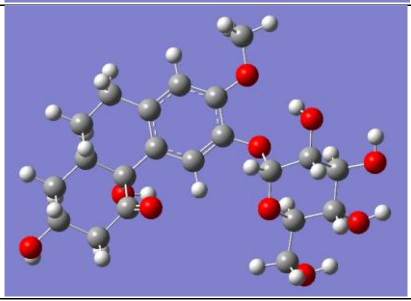	-1568.898199	13.00

Figure S172. Cartesian Coordinates of low energy conformers 1_1 to 1_5

Conformer 1_1							
Atom	X	Y	Z	Atom	X	Y	Z
C	-0.22015	0.554744	0.495583	O	5.788954	-1.06393	-0.35362
C	-1.50636	0.991602	0.137089	O	4.698539	0.83664	1.550227
C	-1.66988	2.319448	-0.28739	H	-0.08042	-0.46057	0.816342
C	-0.55137	3.170231	-0.32495	H	-0.69649	4.19515	-0.64803
C	0.727176	2.723839	0.000293	H	-4.98506	2.169693	-1.28529
C	0.886522	1.38013	0.399934	H	-3.66231	1.319907	-2.08817
C	-2.64176	-0.03246	0.045533	H	-2.87741	3.59297	-1.52624
C	-4.01663	0.660669	-0.072	H	-3.45851	3.432876	0.120769
C	-3.97898	1.760953	-1.13625	H	-4.47825	-0.70863	2.327089
C	-3.01534	2.869327	-0.71466	H	-3.99145	-2.4174	2.239372
C	-2.69693	-1.09762	1.190031	H	-5.99036	-2.01858	0.808969
C	-4.08095	-1.4922	1.666848	H	-6.10628	0.165243	0.010393
C	-5.01658	-1.67296	0.447026	H	-5.28558	-0.55411	-1.3657
C	-5.178	-0.32869	-0.30003	H	-1.5923	-1.25599	-1.14229
O	-1.70803	-1.70553	1.57058	H	-3.78708	-2.3372	-0.89119
O	-2.48407	-0.8515	-1.14603	H	1.360334	4.906524	-1.48698
O	-4.54271	-2.70733	-0.39982	H	2.761022	5.237398	-0.43286
O	2.187032	0.954466	0.57423	H	1.105895	5.403378	0.211432
O	1.86473	3.473734	-0.0517	H	2.164236	-0.13138	2.335505
C	1.750572	4.827775	-0.46461	H	1.846738	-0.86295	-1.19557
H	-4.1759	1.151589	0.900175	H	3.905398	-2.84366	-0.08994
C	2.499328	-0.2331	1.297157	H	4.147605	0.073641	-0.93163
O	1.880998	-1.40119	0.817326	H	4.362382	-1.13865	1.861501
C	2.131942	-1.71553	-0.56676	H	1.509977	-3.76372	-0.29628
C	3.62833	-1.98983	-0.73092	H	1.368146	-3.15406	-1.95692
C	4.403346	-0.76002	-0.25856	H	-0.33637	-2.51939	0.227737
C	4.025636	-0.35562	1.174207	H	4.858565	-2.25217	-2.18988
C	1.22658	-2.89441	-0.90563	H	6.258727	-0.31151	0.035356
O	-0.1537	-2.58777	-0.72408	H	4.200474	1.56214	1.139553
O	3.895159	-2.27237	-2.09447				
Conformer 1_2							
Atom	X	Y	Z	Atom	X	Y	Z
C	0.281821	0.470346	0.276926	O	-6.05473	-0.73306	0.303708
C	1.600091	0.951957	0.196369	O	-4.5658	1.591038	-0.61691
C	1.812613	2.334739	0.090689	H	0.085985	-0.59037	0.367296
C	0.709458	3.202784	0.038408	H	0.892847	4.268692	-0.04107
C	-0.59772	2.719779	0.083892	H	5.283691	2.355239	0.270562
C	-0.8007	1.331114	0.208998	H	4.233642	1.85101	1.597953
C	2.754326	-0.02782	0.329113	H	3.243536	3.862133	0.575855
C	4.105332	0.580431	-0.13259	H	3.421359	3.191496	-1.03375
C	4.290212	1.95933	0.509207	H	3.508133	-3.23766	-0.85557
C	3.209177	2.922693	0.011892	H	3.681637	-2.62774	0.812758

C	2.580894	-1.34327	-0.47999	H	5.127549	-1.56704	-1.65692
C	3.692779	-2.34996	-0.24788	H	6.21008	0.055345	-0.18692
C	5.073884	-1.73295	-0.57366	H	5.341841	-0.56171	1.22302
C	5.26487	-0.38986	0.142167	H	2.048862	-0.46435	2.108397
O	1.677498	-1.53382	-1.27377	H	6.129978	-2.81248	0.65038
O	2.930597	-0.4033	1.716077	H	-1.037	5.137108	-1.0569
O	6.127595	-2.64616	-0.30291	H	-1.04864	5.332124	0.720859
O	-2.10097	0.866447	0.282643	H	-2.58542	5.295159	-0.1849
O	-1.72565	3.48378	0.019404	H	-2.20346	0.664318	-1.77712
C	-1.57469	4.889083	-0.13365	H	-2.21304	-1.56661	1.098798
H	4.027171	0.72064	-1.22251	H	-4.43974	-2.48431	-0.79067
C	-2.58602	0.174107	-0.87655	H	-4.28728	-0.26175	1.291591
O	-2.13957	-1.15107	-0.94173	H	-4.52689	-0.0972	-1.74404
C	-2.55818	-1.99851	0.150876	H	-2.1477	-3.75674	-1.02331
C	-4.08648	-2.0729	0.169942	H	-2.15243	-4.01569	0.733346
C	-4.63466	-0.65572	0.323362	H	-0.13104	-2.66308	-0.72488
C	-4.11512	0.256279	-0.79186	H	-5.43934	-2.75121	1.360078
C	-1.84418	-3.32646	-0.05669	H	-6.37489	0.180996	0.302229
O	-0.43584	-3.18177	0.035385	H	-3.91413	2.019579	-0.03702
O	-4.48834	-2.89798	1.251668				
Conformer 1_3							
Atom	X	Y	Z	Atom	X	Y	Z
C	0.285402	0.467365	0.283798	O	-6.06017	-0.71372	0.298229
C	1.603526	0.948877	0.200713	O	-4.55623	1.602067	-0.61823
C	1.815331	2.331396	0.088476	H	0.089457	-0.59281	0.379567
C	0.71182	3.198807	0.032473	H	0.894911	4.264419	-0.05178
C	-0.59509	2.715555	0.080483	H	5.287343	2.354567	0.256227
C	-0.79733	1.327537	0.211791	H	4.245008	1.854625	1.590846
C	2.759823	-0.02818	0.34047	H	3.245792	3.860616	0.568736
C	4.107854	0.577901	-0.13391	H	3.420176	3.188254	-1.04051
C	4.295288	1.959269	0.501599	H	3.505474	-3.24399	-0.81271
C	3.211419	2.920349	0.006112	H	3.730311	-2.61266	0.843934
C	2.586424	-1.35253	-0.45291	H	5.102196	-1.55127	-1.66382
C	3.707041	-2.34842	-0.21773	H	6.2128	0.060732	-0.20076
C	5.076693	-1.7246	-0.5742	H	5.36331	-0.57018	1.212691
C	5.272066	-0.38688	0.137535	H	2.061452	-0.4545	2.124648
O	1.679035	-1.5588	-1.23853	H	6.06776	-3.40193	-0.68108
O	2.941458	-0.38696	1.729539	H	-1.0357	5.128807	-1.06921
O	6.150401	-2.5738	-0.18979	H	-1.04727	5.32965	0.707855
O	-2.09756	0.862315	0.286683	H	-2.58415	5.28919	-0.19768
O	-1.7237	3.478811	0.012608	H	-2.19684	0.663575	-1.77336
C	-1.57327	4.883605	-0.14512	H	-2.22476	-1.57288	1.099423
H	4.019794	0.714695	-1.22366	H	-4.45348	-2.47252	-0.79664
C	-2.58393	0.174374	-0.87417	H	-4.29147	-0.25432	1.289564
O	-2.14444	-1.1533	-0.94017	H	-4.5244	-0.08572	-1.74637
C	-2.57092	-2.0003	0.14989	H	-2.17009	-3.75705	-1.02891

C	-4.09976	-2.06512	0.165558	H	-2.18157	-4.02225	0.726918
C	-4.63962	-0.64499	0.320306	H	-0.14716	-2.66877	-0.71291
C	-4.11274	0.264914	-0.79306	H	-5.4591	-2.73581	1.352523
C	-1.86577	-3.3326	-0.05976	H	-6.3746	0.202327	0.29762
O	-0.45693	-3.19928	0.037098	H	-3.90227	2.027309	-0.03838
O	-4.50918	-2.88987	1.244691				
Conformer 1_4							
Atom	X	Y	Z	Atom	X	Y	Z
C	0.284924	0.466375	0.274477	O	-6.06234	-0.70838	0.285065
C	1.603835	0.947088	0.195962	O	-4.55226	1.599579	-0.64167
C	1.815917	2.330508	0.093946	H	0.087697	-0.59381	0.365393
C	0.71315	3.19893	0.039883	H	0.897162	4.264953	-0.03673
C	-0.59408	2.716268	0.081278	H	5.285422	2.355989	0.29613
C	-0.79718	1.327716	0.20509	H	4.226709	1.845966	1.614426
C	2.759615	-0.03217	0.331287	H	3.241241	3.859466	0.589633
C	4.110645	0.581591	-0.12123	H	3.430526	3.191613	-1.01996
C	4.290641	1.957954	0.526587	H	3.532278	-3.22795	-0.88712
C	3.211981	2.920875	0.023983	H	3.725942	-2.64929	0.787273
C	2.590285	-1.35116	-0.47539	H	5.123931	-1.52787	-1.67148
C	3.714847	-2.34928	-0.26591	H	6.212133	0.072682	-0.15745
C	5.080296	-1.72085	-0.5853	H	5.338162	-0.59221	1.22923
C	5.267069	-0.39119	0.155873	H	2.047221	-0.50316	2.099441
O	1.663936	-1.56249	-1.23674	H	6.926857	-2.33808	-0.47482
O	2.930541	-0.40974	1.717001	H	-1.02977	5.136292	-1.05564
O	6.061319	-2.68633	-0.22245	H	-1.04635	5.327091	0.722492
O	-2.09794	0.864223	0.276834	H	-2.58059	5.292787	-0.18766
O	-1.72196	3.480689	0.014828	H	-2.18983	0.649734	-1.782
C	-1.57016	4.886151	-0.1345	H	-2.2308	-1.5622	1.108031
H	4.036352	0.726848	-1.21104	H	-4.45231	-2.47739	-0.78906
C	-2.58073	0.167631	-0.88061	H	-4.29736	-0.24293	1.280197
O	-2.14272	-1.1607	-0.93476	H	-4.51841	-0.09623	-1.75772
C	-2.57329	-1.99819	0.161065	H	-2.16559	-3.76552	-0.99998
C	-4.10221	-2.06236	0.17118	H	-2.18639	-4.01467	0.757991
C	-4.64179	-0.64067	0.312473	H	-0.1429	-2.68426	-0.68994
C	-4.10974	0.260616	-0.80545	H	-5.46692	-2.72331	1.357497
C	-1.86667	-3.33206	-0.03325	H	-6.37589	0.207916	0.276123
O	-0.45857	-3.19674	0.070054	H	-3.90093	2.027362	-0.06072
O	-4.51647	-2.87793	1.255421				
Conformer 1_5							
Atom	X	Y	Z	Atom	X	Y	Z
C	-0.51642	0.321791	-0.11116	O	6.012738	0.261344	0.638323
C	-1.8464	0.775231	-0.20129	O	3.74094	1.816789	1.579133
C	-2.08744	2.145363	-0.37861	H	-0.29582	-0.73003	0.027802
C	-1.0038	3.038681	-0.42019	H	-1.20918	4.096098	-0.54525
C	0.309184	2.592072	-0.29995	H	-5.53454	2.03997	-0.80968
C	0.545818	1.209119	-0.16085	H	-4.37742	1.326098	-1.93692

C	-2.97543	-0.24175	-0.22888	H	-3.49819	3.520008	-1.23524
C	-4.36742	0.404618	0.005778	H	-3.78764	3.148992	0.453413
C	-4.52051	1.633549	-0.89502	H	-3.7795	-3.19013	1.481589
C	-3.49376	2.700338	-0.50733	H	-3.82155	-2.90811	-0.28209
C	-2.85338	-1.36778	0.833845	H	-5.4833	-1.4522	1.822278
C	-3.92449	-2.43835	0.703418	H	-6.46211	-0.16254	-0.00678
C	-5.33706	-1.81426	0.796956	H	-5.47175	-1.00555	-1.20288
C	-5.48678	-0.6335	-0.17075	H	-2.13028	-0.90093	-1.87413
O	-2.03216	-1.36271	1.729551	H	-6.2752	-3.12802	-0.28596
O	-3.03295	-0.87871	-1.52933	H	0.755905	5.06083	-1.35857
O	-6.35191	-2.79242	0.618417	H	2.230718	5.232555	-0.37049
O	1.862818	0.811445	-0.09627	H	0.632239	5.185535	0.421721
O	1.419489	3.388099	-0.29831	H	1.569799	0.078835	1.817512
C	1.233097	4.79391	-0.40803	H	2.844404	-1.27855	-1.35006
H	-4.37935	0.746742	1.052881	H	4.781767	-2.0358	0.896118
C	2.262842	-0.043	0.980189	H	4.425918	0.55218	-0.67485
O	2.212966	-1.40242	0.637438	H	3.976579	-0.07055	2.278594
C	3.095985	-1.81261	-0.42489	H	1.7844	-3.48318	-0.73909
C	4.540484	-1.48515	-0.02783	H	3.207398	-3.86375	0.241308
C	4.669332	0.004692	0.24931	H	4.389417	-3.46667	-1.8154
C	3.683166	0.419462	1.343615	H	6.285338	-1.542	-0.8703
C	2.86105	-3.30944	-0.64698	H	6.034643	1.180188	0.944086
O	3.467054	-3.76737	-1.83871	H	3.202746	2.233203	0.883019
O	5.408036	-1.89169	-1.08408				

5. ECD calculation data of compound 2

Figure S173. The energies and equilibrium populations of stable conformers 2_1, 2_2

No	Conformer	Structure	Gibb free energy (Hartree)	Population (%)
1	2_1	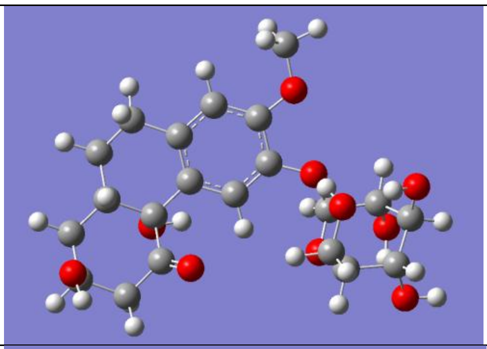	-1568.889589	94.7
2	2_2	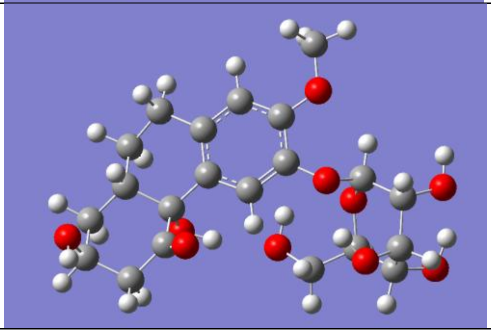	-1568.886858	5.3

Figure S174. Cartesian Coordinates of low energy conformers 2_1, 2_2

Conformer 2_1							
Atom	X	Y	Z	Atom	X	Y	Z
C	-0.41814	0.327976	-0.27848	O	5.027226	0.546905	0.846625
C	-1.77159	0.715328	-0.23718	O	4.131678	-0.66634	-2.49767
C	-2.08785	2.065967	-0.03896	H	-0.15159	-0.71442	-0.39059
C	-1.05047	2.994304	0.146652	H	-1.31022	4.035952	0.299608
C	0.289088	2.61191	0.124783	H	-5.53323	1.878465	-0.43975
C	0.598881	1.254055	-0.10709	H	-4.37	1.547163	-1.72689
C	-2.8469	-0.32622	-0.49783	H	-3.5884	3.534492	-0.48916
C	-4.26003	0.161601	-0.08186	H	-3.81434	2.720898	1.047696
C	-4.50168	1.566616	-0.63895	H	-3.43831	-3.65609	0.396706
C	-3.52322	2.55527	-0.0002	H	-3.57522	-2.93911	-1.22653
C	-2.62636	-1.68409	0.223615	H	-5.81053	-2.95632	-0.24787
C	-3.65927	-2.73582	-0.15415	H	-6.31694	-0.50337	-0.17766
C	-5.08477	-2.22854	0.147657	H	-5.34681	-0.968	-1.58266
C	-5.33092	-0.86085	-0.49356	H	-2.01887	-0.4955	-2.26998
O	-1.7482	-1.89684	1.03655	H	-4.99715	-2.86655	1.999234
O	-2.9098	-0.62297	-1.91779	H	0.564786	5.291817	-0.2752
O	-5.278	-2.05693	1.552813	H	0.495633	4.948472	1.478372
O	1.946168	0.922042	-0.08967	H	2.062895	5.284837	0.69374
O	1.34873	3.443567	0.306176	H	1.803154	-0.08182	-1.88061
C	1.087224	4.815378	0.563992	H	2.702261	-2.99509	0.640633
H	-4.26429	0.217192	1.014899	H	4.990242	-2.00886	1.256316
C	2.383466	-0.13782	-0.95556	H	5.816362	-0.50702	-0.67445
O	2.108648	-1.41603	-0.4357	H	4.043068	1.11606	-1.50938
C	2.921911	-1.92169	0.644973	H	1.384375	-1.55549	2.088101
C	4.423279	-1.79075	0.340088	H	2.947615	-2.01751	2.787676
C	4.841476	-0.3943	-0.1801	H	2.329146	0.508806	1.67785
C	3.87108	0.05739	-1.30057	H	5.638531	-2.77488	-0.84523
C	2.469161	-1.40488	2.016235	H	4.322331	0.427356	1.515386
O	2.807445	-0.05309	2.316325	H	4.111244	-1.60674	-2.25062
O	4.688345	-2.78904	-0.66398				
Conformer 2_2							
Atom	X	Y	Z	Atom	X	Y	Z
C	-0.33433	0.262454	0.543922	O	3.151944	-1.8708	1.850162
C	-1.58363	0.719882	0.100551	O	5.481749	0.343286	0.131148
C	-1.7214	2.076821	-0.22442	H	-0.20623	-0.7743	0.821513
C	-0.62211	2.937507	-0.06904	H	-0.75079	3.986611	-0.31119
C	0.620718	2.472794	0.359992	H	-4.9292	1.983993	-1.54954
C	0.764276	1.097701	0.63557	H	-3.52516	1.204116	-2.28655
C	-2.68216	-0.29516	-0.18936	H	-2.83213	3.431702	-1.46775
C	-4.06638	0.383376	-0.36972	H	-3.55998	3.126879	0.099196
C	-3.93952	1.563302	-1.33786	H	-3.98228	-3.14819	1.359437
C	-3.03318	2.640913	-0.73558	H	-3.57286	-2.95422	-0.36182

C	-2.86623	-1.38066	0.902843	H	-5.96418	-2.52992	-0.13203
C	-3.90853	-2.42815	0.537558	H	-6.09719	-0.14839	-0.89579
C	-5.26935	-1.764	0.246754	H	-4.86241	-1.08059	-1.75715
C	-5.12713	-0.646	-0.78847	H	-1.45174	-1.23556	-1.44442
O	-2.2625	-1.4025	1.957886	H	-5.76913	-1.80736	2.141792
O	-2.39516	-0.98328	-1.42263	H	2.591598	5.075301	0.513433
O	-5.80372	-1.15874	1.426413	H	1.347577	4.866288	-0.74853
O	1.978221	0.551294	1.040177	H	0.864292	5.099109	0.957311
O	1.733661	3.250954	0.523054	H	3.221677	1.654731	-0.18353
C	1.611949	4.649045	0.293603	H	3.137537	-1.62107	-2.30545
H	-4.35768	0.772947	0.614988	H	3.789963	-3.17938	-0.28228
C	3.062929	0.607656	0.09151	H	5.1372	-1.85063	1.37565
O	2.790894	-0.03787	-1.12179	H	4.29472	0.492971	1.846303
C	2.886397	-1.48885	-1.24932	H	1.539749	-3.16359	-1.33983
C	4.030442	-2.12132	-0.43423	H	1.264845	-2.11706	0.058101
C	4.190842	-1.48247	0.965465	H	0.713789	-0.51471	-1.72145
C	4.279638	0.046669	0.843817	H	5.596134	-1.18519	-1.07685
C	1.512586	-2.12043	-1.00973	H	2.469255	-1.1811	1.844286
O	0.511735	-1.46205	-1.7856	H	5.528577	1.295784	-0.02595
O	5.254316	-2.09101	-1.15197				

6. ECD calculation data of compound 3

Figure S175. The energies and equilibrium populations of stable conformers 3_1 - 3_3

No	Conformer	Structure	Gibb free energy (Hartree)	Population (%)
1	3_1	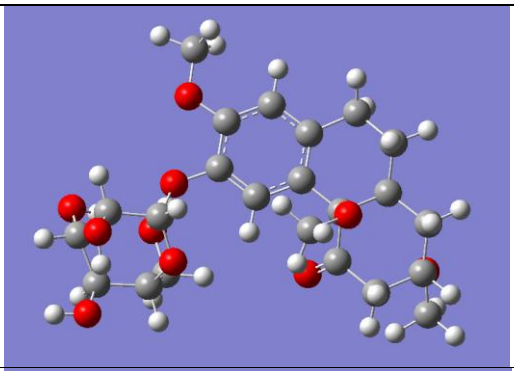	-1647.444580	67.7
2	3_2	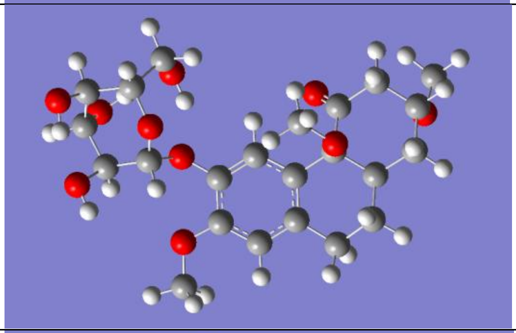	-1647.442722	9.4
3	3_3	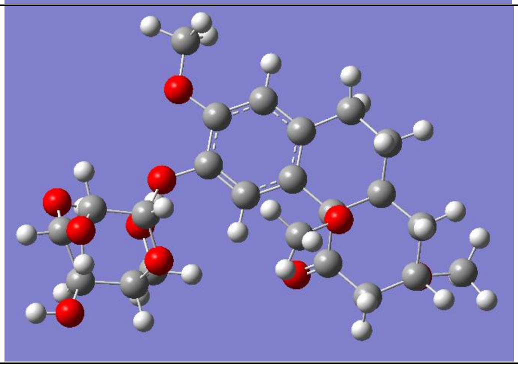	-1647.443552	22.9

Figure S176. Cartesian Coordinates of low energy conformers 3_1 - 3_3

Conformer 3_1							
Atom	X	Y	Z	Atom	X	Y	Z
C	0.148118	0.506769	0.241869	C	1.725625	-0.46638	2.826433
C	1.470513	0.990976	0.246148	H	-0.05158	-0.53874	0.425284
C	1.689417	2.344993	-0.05049	H	0.787814	4.217776	-0.5761
C	0.596239	3.174864	-0.34872	H	5.148764	2.433887	0.249208
C	-0.71104	2.694154	-0.35602	H	4.081231	2.111572	1.617848
C	-0.92475	1.33461	-0.04727	H	3.069431	3.943723	0.346551
C	2.627599	0.065837	0.609575	H	3.335624	3.112694	-1.1732
C	3.98448	0.61545	0.07476	H	3.455717	-3.29543	0.083594
C	4.152689	2.06955	0.525781	H	3.672979	-2.35477	1.575863
C	3.079595	2.949333	-0.11584	H	5.80894	-2.35179	0.389945
C	2.510109	-1.3806	0.047976	H	6.088896	0.12346	0.071864
C	3.642952	-2.29673	0.483589	H	5.259752	-0.27972	1.583066
C	4.994276	-1.71862	0.000744	H	-1.02817	4.916579	-1.87461
C	5.162168	-0.28037	0.49332	H	-1.19709	5.362999	-0.15153
O	1.648275	-1.74869	-0.7263	H	-2.64785	5.182672	-1.1745
O	2.823793	-0.02759	2.042057	H	-2.14443	0.147494	1.808546
O	5.078929	-1.66728	-1.42027	H	-2.69462	-3.14163	-0.32021
O	-2.23863	0.885184	-0.11052	H	-4.99517	-2.41455	-1.19575
O	-1.8195	3.428281	-0.6395	H	-6.03797	-0.7255	0.454541
C	-1.64728	4.796591	-0.97667	H	-4.4291	1.117707	1.152645
H	3.921866	0.59146	-1.021	H	-1.3798	-1.83079	-1.88481
C	-2.66039	-0.07517	0.870773	H	-2.86748	-2.48549	-2.59557
O	-2.27171	-1.39048	0.551677	H	-2.46821	0.201908	-1.81121
C	-2.98033	-2.09726	-0.48867	H	-5.72891	-2.92607	0.956573
C	-4.50365	-2.03361	-0.28919	H	-4.46165	0.008003	-1.75514
C	-5.04154	-0.61266	0.005096	H	-4.36773	-1.47709	2.262258
C	-4.17662	0.055761	1.10333	H	5.936245	-3.55817	-1.64183
C	-2.47388	-1.74362	-1.89255	H	4.194014	-3.46952	-2.02914
O	-2.87004	-0.47004	-2.39443	H	5.384012	-2.7233	-3.11714
O	-4.77027	-2.90247	0.828635	H	1.344902	-1.4474	2.505443
O	-5.21834	0.162896	-1.15342	H	2.101584	-0.55592	3.848518
O	-4.46816	-0.51625	2.373533	H	0.895086	0.248212	2.813271
C	5.145387	-2.92267	-2.0698				
Conformer 3_2							
Atom	X	Y	Z	Atom	X	Y	Z
C	0.322635	0.208102	-0.0808	C	1.737908	-0.83448	2.573391
C	1.559633	0.847198	0.123975	H	0.251028	-0.8703	-0.04157
C	1.609492	2.247486	0.017532	H	0.506613	4.045151	-0.3653
C	0.441631	2.965572	-0.28532	H	4.976515	2.750779	0.709283
C	-0.77818	2.324712	-0.48594	H	3.837213	2.141738	1.912362
C	-0.82558	0.920747	-0.38079	H	2.707848	3.956861	0.717968
C	2.792285	0.049558	0.543862	H	3.23236	3.337487	-0.83538

C	4.106206	0.825914	0.231255	H	4.142525	-3.10343	-0.15927
C	4.020327	2.230465	0.836325	H	4.073868	-2.29587	1.422037
C	2.901653	3.028668	0.167376	H	6.295276	-1.89061	0.482882
C	2.940925	-1.33939	-0.13917	H	6.247322	0.617977	0.43123
C	4.144939	-2.1334	0.342051	H	5.332226	-0.04949	1.791529
C	5.439599	-1.33456	0.065031	H	-2.95604	4.63522	-1.25183
C	5.349314	0.052651	0.702217	H	-1.26345	4.619751	-1.81319
O	2.212833	-1.74638	-1.02239	H	-1.63076	4.899987	-0.08601
O	2.832469	-0.15445	1.978108	H	-2.80944	1.254661	0.970203
O	5.652085	-1.12193	-1.32688	H	-3.72507	-2.47293	1.861178
O	-2.01372	0.25204	-0.64051	H	-5.30715	-2.76246	-0.12544
O	-1.95915	2.950108	-0.76993	H	-6.13914	-0.49275	-0.93342
C	-1.9354	4.355197	-0.98832	H	-4.25174	1.238911	-1.1784
H	4.164291	0.914369	-0.8614	H	-1.66413	-2.89899	0.68911
C	-3.04394	0.367908	0.373187	H	-3.00913	-3.8431	0.029691
O	-3.00346	-0.69341	1.277292	H	-1.91246	-1.52062	-1.16219
C	-3.58351	-1.96891	0.898883	H	-5.83732	-0.55953	1.532095
C	-4.99913	-1.80291	0.30559	H	-3.79161	-1.69093	-1.92752
C	-5.07773	-0.73137	-0.80707	H	-4.94169	1.969508	0.963493
C	-4.40289	0.572266	-0.32326	H	6.260319	-1.94388	-3.07131
C	-2.59506	-2.83235	0.109172	H	6.776263	-2.85559	-1.62871
O	-2.31986	-2.40631	-1.22113	H	5.083389	-2.94545	-2.19494
O	-5.9204	-1.50988	1.348246	H	1.576397	-1.83161	2.136789
O	-4.60077	-1.15619	-2.05978	H	1.995527	-0.95498	3.628301
O	-5.31518	1.142733	0.630167	H	0.803326	-0.26765	2.49645
C	5.951235	-2.28291	-2.07989				
Conformer 3_3							
Atom	X	Y	Z	Atom	X	Y	Z
C	0.108187	0.452323	0.255495	C	1.634415	-0.69954	2.78809
C	1.440414	0.908633	0.288145	H	-0.11393	-0.59528	0.392898
C	1.685977	2.270494	0.053065	H	0.824774	4.181991	-0.39449
C	0.611345	3.133885	-0.2156	H	5.139582	2.289163	0.415729
C	-0.70485	2.679662	-0.25334	H	4.035889	1.923782	1.743985
C	-0.9463	1.312468	-0.00557	H	3.087822	3.828672	0.526186
C	2.57716	-0.05614	0.614596	H	3.362467	3.048605	-1.01904
C	3.951544	0.49816	0.135688	H	3.395275	-3.37421	-0.17133
C	4.131842	1.928536	0.65293	H	3.548722	-2.59597	1.429241
C	3.08684	2.851827	0.027788	H	5.72698	-2.50476	0.349966
C	2.436496	-1.47216	-0.02044	H	6.051883	-0.00301	0.196473
C	3.563601	-2.42861	0.347443	H	5.162997	-0.49687	1.632372
C	4.932673	-1.84478	-0.03551	H	-2.58699	5.239263	-0.9739
C	5.104319	-0.43277	0.541353	H	-0.96728	4.972985	-1.6738
O	1.536844	-1.80137	-0.76731	H	-1.14115	5.345815	0.06621
O	2.749721	-0.2352	2.043072	H	-2.2169	0.094372	1.797286
O	4.973569	-1.84981	-1.46299	H	-2.78568	-3.12227	-0.43419
O	-2.26705	0.889879	-0.10043	H	-5.05797	-2.33016	-1.3282

O	-1.79662	3.447114	-0.51168	H	-6.10083	-0.67086	0.35148
C	-1.5956	4.825347	-0.78642	H	-4.47369	1.122533	1.131804
H	3.911898	0.522585	-0.96199	H	-1.42164	-1.79205	-1.93914
C	-2.72071	-0.09216	0.845099	H	-2.90804	-2.39967	-2.69282
O	-2.34867	-1.40387	0.494076	H	-2.47524	0.256865	-1.82584
C	-3.05076	-2.06887	-0.57805	H	-5.83835	-2.88976	0.795463
C	-4.57597	-1.98402	-0.40278	H	-4.47205	0.097317	-1.80772
C	-5.09453	-0.56282	-0.07676	H	-4.47585	-1.50413	2.165384
C	-4.23832	0.058252	1.055674	H	6.160831	-1.85271	-3.09957
C	-2.51366	-1.68518	-1.96246	H	6.736941	-0.7625	-1.81839
O	-2.87924	-0.3908	-2.43424	H	6.939136	-2.52511	-1.6425
O	-4.87733	-2.87956	0.684824	H	1.238867	-1.65155	2.404692
O	-5.23659	0.248958	-1.21506	H	1.99761	-0.8575	3.806513
O	-4.56205	-0.54535	2.303266	H	0.817902	0.030763	2.810083
C	6.268453	-1.73695	-2.01864				

7. ECD calculation data of compound 5

Figure S177. The energies and equilibrium populations of stable conformers 5_1 - 5.6

No	Conformer	Structure	Gibb free energy (Hartree)	Population (%)
1	5_1	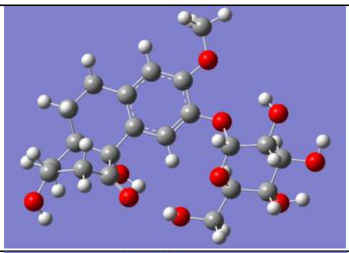	-1568.895744	0.2
2	5_2	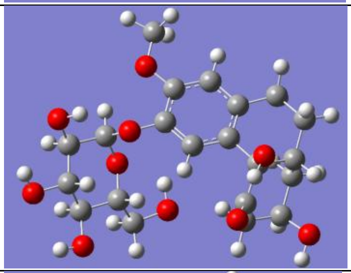	-1568.901201	65.24
3	5_3	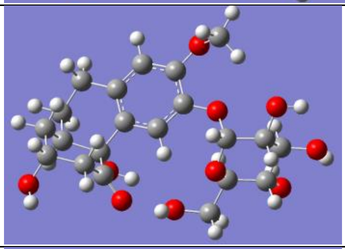	-1568.894031	0.03
4	5_4	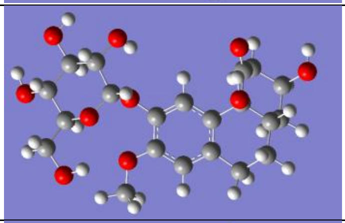	-1568.900599	34.48
5	5_5	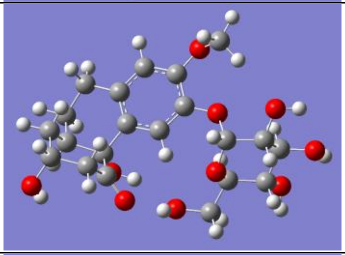	-1568.892339	0.01
6	5_6	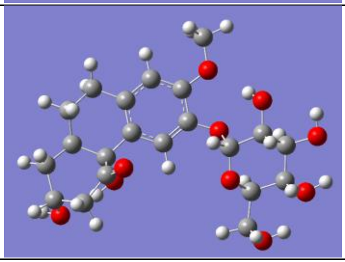	-1568.894285	0.04

Figure S178. Cartesian Coordinates of low energy conformers 5_1 - 5_6

Conformer 5_1							
Atom	X	Y	Z	Atom	X	Y	Z
C	-0.22704	0.426317	-0.08017	O	6.000104	-0.6546	0.082852
C	-1.54913	0.792636	-0.36549	O	4.459818	1.362824	1.496308
C	-1.85876	2.1474	-0.5267	H	0.031763	-0.61486	0.023112
C	-0.83904	3.105079	-0.3842	H	-1.09394	4.152442	-0.50303
C	0.477928	2.738237	-0.10483	H	-5.30116	1.85842	-0.77318
C	0.779637	1.367128	0.039844	H	-4.34825	1.586342	0.675961
C	-2.55655	-0.34621	-0.58835	H	-3.33448	2.694264	-1.98271
C	-3.9864	0.157226	-0.94292	H	-3.46948	3.572607	-0.46864
C	-4.30473	1.567597	-0.41954	H	-3.56244	0.060459	2.0521
C	-3.26127	2.582638	-0.89088	H	-3.62369	-1.6473	2.519939
C	-2.59165	-1.2335	0.687757	H	-5.75633	-0.32016	1.444085
C	-3.6992	-0.96026	1.674779	H	-6.01144	-0.66882	-0.91588
C	-5.10727	-1.08656	1.010356	H	-4.71263	-1.8556	-0.96415
C	-5.01885	-0.89984	-0.51614	H	-1.37028	-1.67178	-1.44728
O	-1.78122	-2.13323	0.854185	H	-5.26079	-3.02753	0.917266
O	-2.16822	-1.1682	-1.68555	H	2.228043	5.488152	0.038698
O	-5.74481	-2.30986	1.349969	H	0.89582	5.220154	-1.11748
O	2.105166	1.024228	0.219111	H	0.551171	5.354186	0.631899
O	1.523837	3.600281	0.043165	H	1.96694	0.222202	2.121084
C	1.270004	4.989227	-0.11242	H	2.227292	-1.08384	-1.25899
H	-3.98803	0.209923	-2.03785	H	4.282409	-2.58963	0.441802
C	2.4446	0.00425	1.159702	H	4.343544	0.157205	-0.87601
O	2.003193	-1.28154	0.804191	H	4.292229	-0.57091	2.086751
C	2.494415	-1.78367	-0.45692	H	1.994594	-3.80198	0.109785
C	4.019096	-1.8958	-0.37447	H	2.162059	-3.5315	-1.63589
C	4.590109	-0.51831	-0.0416	H	-0.05643	-2.82464	-0.00967
C	3.976354	0.05202	1.243057	H	5.470556	-2.23209	-1.59452
C	1.773375	-3.1028	-0.7089	H	6.329568	0.204868	0.384206
O	0.37007	-2.9368	-0.87401	H	3.920587	1.954514	0.945976
O	4.511934	-2.36712	-1.61764				
Conformer 5_2							
Atom	X	Y	Z	Atom	X	Y	Z
C	0.402955	0.508663	0.803389	O	-5.49319	-1.25689	1.194637
C	1.756081	0.786161	0.556404	O	-4.55753	1.482231	1.186574
C	2.091966	1.986408	-0.08513	H	0.112957	-0.40027	1.321064
C	1.070431	2.878954	-0.44941	H	1.347493	3.813146	-0.92577
C	-0.27224	2.599373	-0.20147	H	4.636996	1.801797	1.360223
C	-0.60493	1.374642	0.413901	H	5.54828	1.615043	-0.13818
C	2.837202	-0.16543	1.090229	H	3.699012	3.406422	-0.1897
C	4.169638	-0.00316	0.309118	H	3.669036	2.254672	-1.50364
C	4.559831	1.481629	0.316407	H	1.526486	-1.84309	-0.9718
C	3.528414	2.347429	-0.4173	H	2.104351	-3.3404	-0.24618

C	2.370604	-1.63229	0.989719	H	3.591886	-2.44666	-2.12531
C	2.337868	-2.27778	-0.37324	H	3.369038	-0.06262	-1.72723
C	3.687236	-2.08187	-1.09124	H	5.068312	-0.51684	-1.59246
C	4.090244	-0.60646	-1.10656	H	2.711322	-0.64312	2.941088
O	2.071692	-2.22685	2.014256	H	4.465221	-3.7062	-0.31957
O	3.06507	0.129192	2.460747	H	-0.38524	5.298002	-0.43139
O	4.731445	-2.78208	-0.41377	H	-0.5286	4.582929	-2.06405
O	-1.91873	1.033501	0.703482	H	-1.98067	5.190132	-1.22307
O	-1.31381	3.429221	-0.50666	H	-2.83232	1.771925	-1.00484
C	-1.01978	4.692097	-1.08922	H	-1.6379	-1.53243	0.088476
H	4.917664	-0.56765	0.874011	H	-4.43117	-1.86381	-1.11716
C	-2.80363	0.857183	-0.411	H	-3.48514	-0.93245	1.626857
O	-2.37942	-0.15339	-1.29066	H	-4.9213	0.588423	-0.5939
C	-2.33644	-1.49612	-0.75559	H	-2.50524	-2.35203	-2.72348
C	-3.72787	-1.898	-0.26779	H	-1.66537	-3.38026	-1.54121
C	-4.18597	-0.88643	0.778133	H	-0.62431	-0.92772	-2.44467
C	-4.17189	0.532954	0.203439	H	-4.47782	-3.34586	0.757261
C	-1.78761	-2.35217	-1.88824	H	-5.80857	-0.54202	1.767122
O	-0.51403	-1.88071	-2.30996	H	-3.76023	1.66159	1.709699
O	-3.65113	-3.20615	0.272668				
Conformer 5_3							
Atom	X	Y	Z	Atom	X	Y	Z
C	-0.36907	0.39538	0.136681	O	5.858589	-0.42805	0.196179
C	-1.65892	0.656051	-0.33993	O	4.289083	1.240239	1.842849
C	-1.96442	1.93042	-0.84327	H	-0.11431	-0.59772	0.471507
C	-0.96138	2.90795	-0.82161	H	-1.16375	3.908515	-1.19367
C	0.334199	2.650157	-0.37139	H	-3.70496	0.566283	-2.69828
C	0.638172	1.353055	0.091006	H	-5.1685	1.269904	-2.00874
C	-2.62333	-0.53632	-0.4858	H	-3.22704	2.912869	-2.27318
C	-4.09088	-0.07231	-0.68824	H	-3.89764	2.844423	-0.66156
C	-4.12867	1.001551	-1.78854	H	-2.72837	-0.12298	2.427265
C	-3.33561	2.257036	-1.40181	H	-3.14476	-1.80902	2.761097
C	-2.49081	-1.43168	0.767415	H	-5.16549	-0.25501	2.633952
C	-3.23512	-1.00813	2.019431	H	-4.30236	1.315265	0.990651
C	-4.69864	-0.65421	1.71968	H	-5.81619	0.626426	0.406115
C	-4.76535	0.395951	0.613306	H	-1.37593	-1.5801	-1.60313
O	-1.77396	-2.4219	0.774541	H	-5.32521	-2.50156	1.910399
O	-2.30169	-1.28485	-1.6487	H	2.553493	4.912158	0.414934
O	-5.41662	-1.79654	1.255921	H	2.77486	3.20415	0.900021
O	1.963343	1.073347	0.36546	H	1.396307	4.149836	1.534804
O	1.258483	3.650672	-0.49104	H	1.808627	0.118981	2.19112
C	2.043243	3.979726	0.664984	H	2.017719	-0.82744	-1.28181
H	-4.6288	-0.95687	-1.0419	H	4.217666	-2.48539	0.070589
C	2.305875	0.000432	1.221804	H	4.208939	0.467561	-0.701
O	1.898232	-1.27064	0.746747	H	4.07985	-0.81526	2.057068
C	2.352199	-1.60327	-0.5768	H	1.892637	-3.67071	-0.19504

C	3.886946	-1.65661	-0.56602	H	2.124722	-3.26024	-1.90325
C	4.453071	-0.3486	-0.00092	H	-0.20842	-2.84881	-0.34449
C	3.832583	-0.00489	1.353697	H	4.11328	-1.25669	-2.46638
C	1.678359	-2.91677	-0.96493	H	6.233055	-0.77386	-0.62721
O	0.282984	-2.78169	-1.18172	H	5.249767	1.239539	1.719692
O	4.452326	-1.92356	-1.8505				
Conformer 5_4							
Atom	X	Y	Z	Atom	X	Y	Z
C	0.876418	-0.30143	0.219477	O	-4.76836	-3.17055	-0.50807
C	2.076168	0.423284	0.316136	O	-1.99534	-3.06426	0.320883
C	2.052538	1.800169	0.0549	H	0.855285	-1.36469	0.443532
C	0.844558	2.409799	-0.32203	H	0.844645	3.475727	-0.52198
C	-0.34336	1.686506	-0.42963	H	4.233017	1.953145	1.9867
C	-0.31283	0.302849	-0.15202	H	5.37518	2.531081	0.773763
C	3.345723	-0.28412	0.8115	H	3.04229	3.601075	0.667511
C	4.630478	0.51618	0.449014	H	3.625211	2.946516	-0.84311
C	4.445208	1.967881	0.912897	H	3.00394	-1.33525	-1.87069
C	3.298507	2.660232	0.166521	H	3.873665	-2.84805	-1.54515
C	3.440751	-1.6916	0.194332	H	5.334458	-1.04823	-2.60446
C	3.800101	-1.79098	-1.26976	H	4.263412	0.915928	-1.6723
C	5.120724	-1.03616	-1.52434	H	5.963823	0.912577	-1.20768
C	5.004254	0.409998	-1.04301	H	3.188462	-1.38405	2.375779
O	3.261845	-2.67064	0.900912	H	6.239024	-2.56821	-1.02723
O	3.27373	-0.42463	2.222519	H	-1.27203	4.208413	-0.22761
O	6.193854	-1.63042	-0.79807	H	-1.06922	3.855432	-1.96867
O	-1.45673	-0.46713	-0.30748	H	-2.69809	3.795392	-1.20798
O	-1.54754	2.209099	-0.77454	H	-1.62571	-0.68855	1.742828
C	-1.63634	3.600728	-1.06497	H	-3.73164	0.713653	-0.90665
H	5.434657	0.056208	1.030988	H	-5.49738	-1.06732	0.860298
C	-2.26582	-0.68586	0.855157	H	-3.55729	-1.72924	-1.39231
O	-3.21602	0.322154	1.069759	H	-3.40068	-2.35191	1.591592
C	-4.22503	0.471213	0.040575	H	-5.56149	1.404988	1.440525
C	-5.0068	-0.83562	-0.10024	H	-5.86117	1.825381	-0.25999
C	-4.02905	-1.95939	-0.42299	H	-3.50254	2.584069	1.058444
C	-2.938	-2.05191	0.645543	H	-6.30857	-1.5614	-1.32054
C	-5.07786	1.65212	0.481634	H	-4.11621	-3.88341	-0.57651
O	-4.30828	2.839969	0.58586	H	-1.43554	-2.67999	-0.37238
O	-5.96735	-0.67517	-1.13165				
Conformer 5_5							
Atom	X	Y	Z	Atom	X	Y	Z
C	-0.372	0.400548	0.13544	O	5.865182	-0.41097	0.221872
C	-1.66215	0.656401	-0.34275	O	4.28029	1.258809	1.829259
C	-1.97091	1.928184	-0.85024	H	-0.11365	-0.59052	0.472708
C	-0.97098	2.908443	-0.8296	H	-1.17597	3.90776	-1.20339
C	0.32526	2.655947	-0.37752	H	-3.7003	0.553414	-2.70343
C	0.633305	1.359858	0.086757	H	-5.16987	1.253475	-2.02055

C	-2.62141	-0.53844	-0.48247	H	-3.23515	2.902369	-2.28457
C	-4.09046	-0.07783	-0.69082	H	-3.90845	2.836115	-0.67356
C	-4.12946	0.990725	-1.79731	H	-2.75231	-0.15495	2.441619
C	-3.34225	2.249507	-1.41093	H	-3.1901	-1.84815	2.747789
C	-2.48751	-1.43192	0.775816	H	-5.17633	-0.26195	2.626669
C	-3.25731	-1.03543	2.020608	H	-4.30048	1.30264	0.981082
C	-4.72071	-0.66854	1.717891	H	-5.82064	0.627691	0.395118
C	-4.77312	0.386485	0.606674	H	-1.36925	-1.5674	-1.61045
O	-1.75931	-2.41504	0.778619	H	-5.1193	-2.27338	0.65652
O	-2.30383	-1.29839	-1.64042	H	1.412451	4.136504	1.53252
O	-5.50358	-1.82156	1.420121	H	2.785732	3.208371	0.862867
O	1.957223	1.083405	0.363022	H	2.544013	4.920642	0.401459
O	1.242236	3.661433	-0.49659	H	1.799568	0.119143	2.182227
C	2.04533	3.981564	0.649634	H	2.077064	-0.80734	-1.29685
H	-4.62454	-0.96096	-1.06548	H	4.212233	-2.477	0.141676
C	2.301048	0.00698	1.214236	H	4.217688	0.447181	-0.7139
O	1.899779	-1.26329	0.731948	H	4.073181	-0.79299	2.067708
C	2.373869	-1.58981	-0.58757	H	1.906893	-3.66464	-0.24055
C	3.909086	-1.66571	-0.5373	H	2.106049	-3.21587	-1.94838
C	4.460295	-0.3469	0.006869	H	-0.18942	-2.81774	-0.33943
C	3.827555	0.008147	1.352716	H	4.500602	-2.77597	-2.04798
C	1.68519	-2.89103	-0.98988	H	6.259369	-0.63445	-0.63323
O	0.288257	-2.7526	-1.18495	H	5.238456	1.264045	1.685847
O	4.500814	-1.83668	-1.82735				
Conformer 5_6							
Atom	X	Y	Z	Atom	X	Y	Z
C	-0.60107	0.251688	-0.21137	O	5.947991	0.528106	0.48176
C	-1.93451	0.690539	-0.27754	O	3.611749	2.05003	1.292476
C	-2.19138	2.053189	-0.45504	H	-0.37747	-0.80266	-0.10546
C	-1.1209	2.961602	-0.53208	H	-1.34279	4.01614	-0.6527
C	0.197005	2.52678	-0.44142	H	-5.63357	1.985147	-0.08362
C	0.449049	1.147571	-0.29269	H	-4.44624	1.649974	1.173578
C	-3.02563	-0.36774	-0.1957	H	-3.86474	2.671326	-1.65184
C	-4.4667	0.194656	-0.42265	H	-3.67848	3.577694	-0.16039
C	-4.60771	1.636025	0.087032	H	-4.25141	-2.37752	2.326869
C	-3.60706	2.570413	-0.58692	H	-3.44659	-3.22003	1.00931
C	-3.00346	-1.11463	1.164035	H	-6.06742	-2.67889	0.885507
C	-3.97527	-2.29675	1.272026	H	-5.82706	-0.29187	1.206673
C	-5.23098	-2.16104	0.397059	H	-6.50529	-0.58371	-0.37909
C	-5.58384	-0.6833	0.209427	H	-3.43849	-1.97047	-1.27753
O	-2.28155	-0.81943	2.093276	H	-5.72135	-2.68128	-1.44444
O	-2.69257	-1.3454	-1.1919	H	2.092288	5.187332	-0.57638
O	-4.96515	-2.82166	-0.85811	H	0.596114	4.990531	-1.52774
O	1.771491	0.756317	-0.24917	H	0.513455	5.130092	0.253603
O	1.302144	3.335346	-0.46963	H	1.532786	0.246631	1.74275
C	1.098977	4.737197	-0.58663	H	2.847062	-1.40676	-1.26038

H	-4.58761	0.199613	-1.51407	H	4.851271	-1.78975	1.022945
C	2.225524	0.062447	0.916692	H	4.330481	0.577732	-0.82546
O	2.248251	-1.32938	0.73916	H	3.950281	0.262541	2.185373
C	3.139571	-1.81316	-0.28412	H	1.919726	-3.57553	-0.39109
C	4.568459	-1.36317	0.046517	H	3.355026	-3.75602	0.629966
C	4.616171	0.153256	0.149595	H	4.522001	-3.54412	-1.47159
C	3.62205	0.634651	1.208187	H	6.303775	-1.41355	-0.81515
C	2.985229	-3.33618	-0.32097	H	5.914897	1.473819	0.689057
O	3.62282	-3.90803	-1.44548	H	3.03098	2.367522	0.577357
O	5.448217	-1.83944	-0.97073				

8. ECD calculation data of compound 9

Figure S179. The energies and equilibrium populations of stable conformers 9_1 - 3_7

No	Conformer	Structure	Gibb free energy (Hartree)	Population (%)
1	9_1	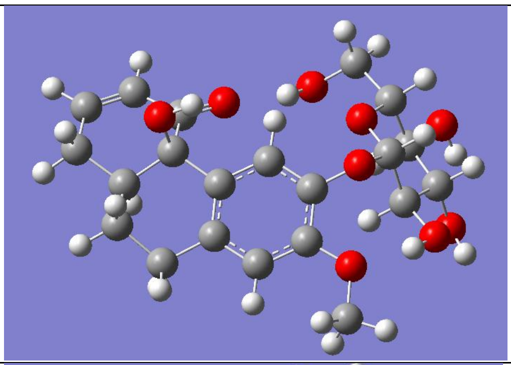	-1492.480304	36.57
2	9_2	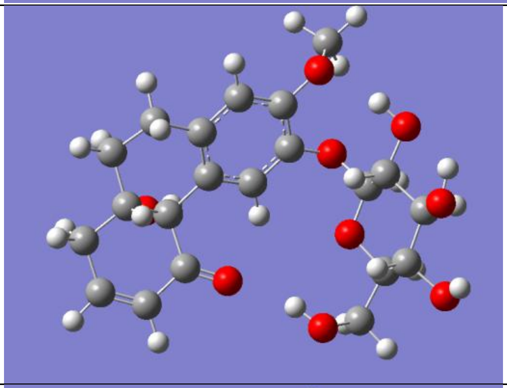	-1492.479302	12.65
3	9_3	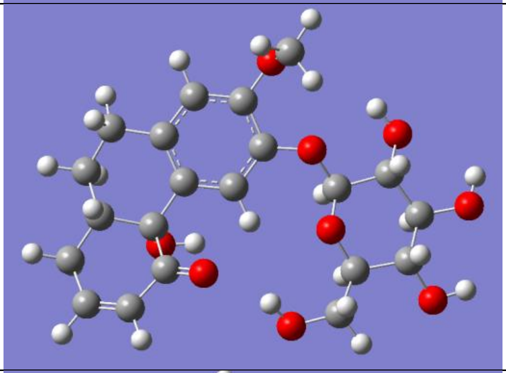	-1492.477999	3.19
4	9_4	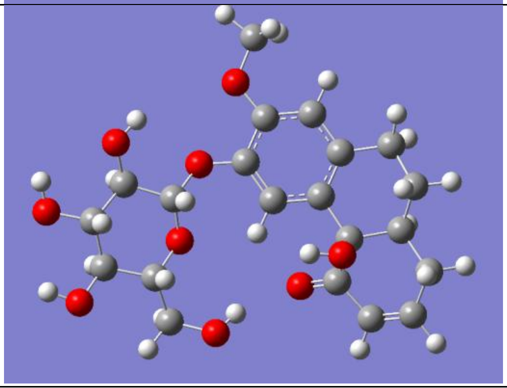	-1492.480115	29.93

No	Conformer	Structure	Gibb free energy (Hartree)	Population (%)
5	9_5	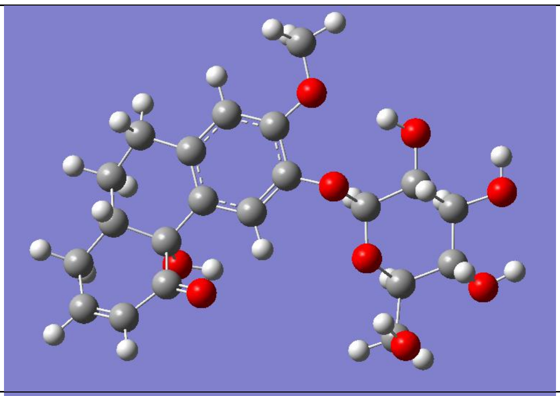	-1492.479119	10.42
6	9_6	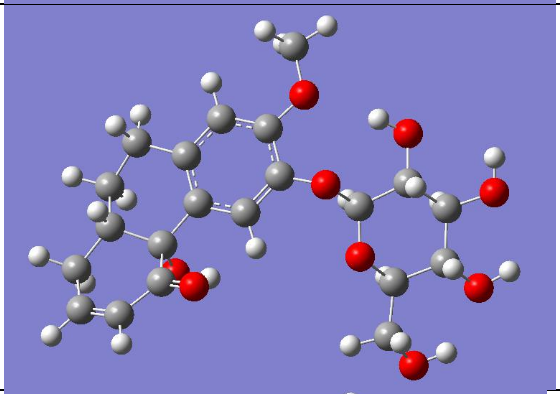	-1492.478772	7.22
7	9_7	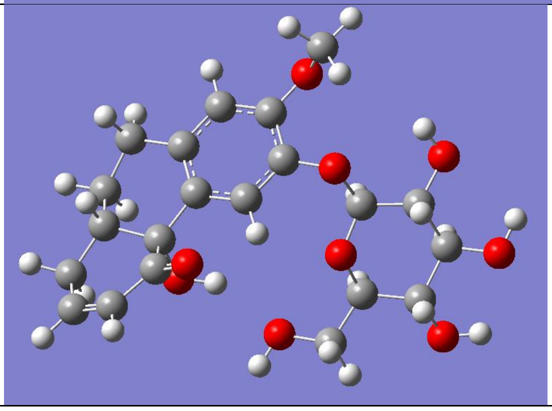	-1492.473341	0.02

Figure S180. Cartesian Coordinates of low energy conformers 9_1 - 9_7

Conformer 9_1							
Atom	X	Y	Z	Atom	X	Y	Z
C	0.687386	0.398807	0.901709	O	-4.65096	-2.57351	-0.88878
C	1.89173	0.632577	0.213532	O	-4.69928	0.11483	-1.84349
C	2.080743	1.881099	-0.40363	O	-3.68643	2.031751	-0.01492
C	1.054989	2.836861	-0.35867	H	0.504072	-0.54158	1.400597
C	-0.14923	2.582566	0.294019	H	1.221489	3.794036	-0.83998
C	-0.32891	1.342479	0.937666	H	4.820036	1.615664	0.371524
C	3.004695	-0.40904	0.264259	H	5.420787	1.555706	-1.28934
C	4.145505	-0.10521	-0.73077	H	3.591515	3.299386	-0.96513
C	4.542804	1.369417	-0.65993	H	3.231267	2.109592	-2.2023
C	3.370241	2.236974	-1.11818	H	3.297177	-3.899	-0.24699
C	2.541831	-1.87984	0.027712	H	5.634324	-3.26431	-0.6552
C	3.610724	-2.85913	-0.24613	H	5.833801	-0.82637	0.425261
C	4.882007	-2.49701	-0.48057	H	6.066385	-0.92574	-1.31336
C	5.323696	-1.06596	-0.5179	H	2.896304	-0.29158	2.222642
O	1.379111	-2.24729	0.136057	H	-0.87149	4.672615	-1.31255
O	3.607816	-0.42346	1.581412	H	-1.96478	5.287473	-0.04261
O	-1.47245	1.133296	1.685985	H	-0.20131	5.299381	0.224076
O	-1.19332	3.469405	0.368682	H	-3.24966	0.288213	1.967158
C	-1.03403	4.751577	-0.23115	H	-3.69024	-2.09497	1.445599
H	3.740403	-0.30339	-1.73491	H	-2.85238	-1.68497	-1.45813
C	-2.47751	0.270572	1.183911	H	-4.95635	-0.20988	0.191397
O	-1.94754	-1.02945	1.027549	H	-2.28093	0.764221	-0.89958
C	-2.9195	-2.02332	0.65949	H	-1.72935	-3.56959	1.550616
C	-3.60502	-1.6396	-0.65869	H	-2.93065	-4.13521	0.368387
C	-4.1593	-0.22274	-0.57146	H	-0.46808	-2.87983	-0.21858
C	-3.07768	0.757845	-0.13967	H	-5.12594	-2.25373	-1.66886
C	-2.1845	-3.36103	0.570392	H	-4.95909	1.045554	-1.78429
O	-1.23741	-3.4124	-0.47358	H	-2.97774	2.655598	0.217412
Conformer 9_2							
Atom	X	Y	Z	Atom	X	Y	Z
C	-0.68413	0.49658	-0.83688	O	4.603079	-2.62143	0.829345
C	-1.88308	0.677741	-0.12726	O	4.484983	-0.06608	2.107507
C	-2.07557	1.873532	0.594654	O	3.528692	2.030573	0.489106
C	-1.05262	2.829564	0.612459	H	-0.49916	-0.4074	-1.39829
C	0.143997	2.627684	-0.06542	H	-1.17851	3.75691	1.164008
C	0.33165	1.445768	-0.79694	H	-4.82123	1.653691	-0.17608
C	-2.99399	-0.36348	-0.25081	H	-5.40491	1.454739	1.479696
C	-4.1252	-0.14653	0.777212	H	-3.58773	3.23155	1.282593
C	-4.53201	1.326444	0.829252	H	-3.21	1.947316	2.414805
C	-3.36054	2.161579	1.346188	H	-3.26496	-3.8856	-0.01426
C	-2.52316	-1.84794	-0.14257	H	-5.59978	-3.29821	0.471334

C	-3.58344	-2.85075	0.070777	H	-5.82292	-0.7843	-0.41385
C	-4.85342	-2.51532	0.348203	H	-6.03528	-1.02053	1.313927
C	-5.30097	-1.09424	0.501998	H	-2.90214	-0.11692	-2.19824
O	-1.36225	-2.20057	-0.30601	H	1.68653	3.696595	-1.98604
O	-3.61022	-0.26864	-1.55745	H	2.172224	5.050154	-0.92186
O	1.494765	1.284282	-1.52836	H	0.468846	4.892466	-1.4352
O	1.148628	3.576348	0.035953	H	3.321017	0.524223	-1.74406
C	1.37628	4.343139	-1.16147	H	3.78069	-1.89685	-1.48741
H	-3.70746	-0.42158	1.757763	H	2.742233	-1.85867	1.379671
C	2.488917	0.400988	-1.0355	H	4.895016	-0.13266	0.073561
O	1.98663	-0.91946	-1.07306	H	2.11201	0.612292	1.071562
C	2.957355	-1.9322	-0.75405	H	1.858326	-3.37607	-1.89711
C	3.54217	-1.69446	0.644352	H	3.004881	-4.06351	-0.72512
C	4.048404	-0.26264	0.76975	H	0.492747	-2.91747	-0.10837
C	2.962636	0.735478	0.385076	H	5.009663	-2.3953	1.677802
C	2.252238	-3.28361	-0.87329	H	4.677834	0.880162	2.184087
O	1.250947	-3.4852	0.099551	H	2.796935	2.672443	0.447904
Conformer 9_3							
Atom	X	Y	Z	Atom	X	Y	Z
C	-0.6726	0.509171	0.063073	O	4.902871	-2.67029	-0.52559
C	-2.06492	0.646403	-0.07801	O	5.917288	-0.02803	-0.63048
C	-2.6263	1.936058	-0.1158	O	3.915231	1.986421	-1.07912
C	-1.78371	3.045101	0.041237	H	-0.22659	-0.47175	0.12322
C	-0.41096	2.910535	0.210206	H	-2.19193	4.051392	0.007733
C	0.148046	1.621243	0.210226	H	-4.48557	0.828118	-1.98774
C	-2.91764	-0.60294	-0.28775	H	-5.88752	1.088002	-0.94413
C	-4.42837	-0.31064	-0.1623	H	-4.26262	3.055941	-0.93524
C	-4.8	0.949751	-0.94481	H	-4.58294	2.382303	0.651388
C	-4.11165	2.162753	-0.31847	H	-3.26027	-3.78657	1.221779
C	-2.57744	-1.80573	0.649579	H	-5.48161	-3.62346	0.188159
C	-3.5785	-2.888	0.701271	H	-5.19123	-1.69959	-1.64368
C	-4.7938	-2.78085	0.141166	H	-6.31709	-1.36098	-0.33816
C	-5.25721	-1.54031	-0.55853	H	-1.76832	-1.0796	-1.80893
O	-1.51741	-1.91556	1.254595	H	1.529155	3.478313	1.966832
O	-2.71693	-1.11872	-1.62535	H	1.490721	5.209522	1.525629
O	1.515173	1.542686	0.385961	H	0.08036	4.462997	2.324628
O	0.38254	4.030667	0.300541	H	1.836659	0.298564	-1.22205
C	0.896349	4.297399	1.610042	H	2.399474	-1.99327	-1.00614
H	-4.61757	-0.11961	0.905154	H	4.551976	-1.40758	1.095242
C	2.211467	0.4712	-0.19829	H	4.249668	-0.46729	-1.78923
O	2.043244	-0.69224	0.586163	H	3.978185	1.104471	0.814047
C	2.72185	-1.8355	0.035345	H	2.902738	-3.90047	0.534985
C	4.231878	-1.56334	0.051837	H	2.494526	-2.85672	1.911687
C	4.522976	-0.28576	-0.73655	H	0.357843	-2.76865	1.065276

C	3.685293	0.878537	-0.22241	H	5.820136	-2.38862	-0.65635
C	2.289446	-3.05087	0.847461	H	6.086234	0.795644	-1.10938
O	0.940168	-3.39949	0.615045	H	3.332813	2.699984	-0.78
Conformer 9_4							
Atom	X	Y	Z	Atom	X	Y	Z
C	0.649696	0.441795	-0.15791	O	-5.01138	-2.55433	0.632531
C	2.044228	0.556558	0.001801	O	-5.97734	0.103687	0.436684
C	2.616548	1.838006	0.013538	O	-3.94874	2.123848	0.7261
C	1.794795	2.963563	-0.16438	H	0.185529	-0.53099	-0.20379
C	0.418741	2.84671	-0.3428	H	2.254651	3.945384	-0.14149
C	-0.15159	1.557244	-0.33977	H	4.460472	0.761845	1.919526
C	2.877032	-0.69673	0.258835	H	5.866423	0.975873	0.870563
C	4.391552	-0.42732	0.127347	H	4.265873	2.96103	0.807219
C	4.77726	0.850486	0.874041	H	4.579216	2.241328	-0.76087
C	4.103987	2.053609	0.213886	H	3.191247	-3.93048	-1.15241
C	2.520559	-1.93361	-0.6264	H	5.418255	-3.75526	-0.133
C	3.51841	-3.02001	-0.65879	H	5.135953	-1.7807	1.650142
C	4.736347	-2.90697	-0.10609	H	6.27042	-1.48793	0.340664
C	5.208133	-1.65217	0.561267	H	1.717183	-1.12823	1.785685
O	1.446101	-2.07025	-1.2002	H	0.581459	5.459372	0.410828
O	2.668636	-1.16165	1.615185	H	-0.75946	5.8684	-0.69225
O	-1.51119	1.490585	-0.56156	H	0.803794	5.325288	-1.35794
O	-0.44831	3.886826	-0.50486	H	-1.92097	0.398777	1.134585
C	0.088828	5.200841	-0.53516	H	-2.50883	-1.886	1.110181
H	4.584016	-0.26973	-0.9449	H	-4.5943	-1.46679	-1.09599
C	-2.25035	0.489317	0.084634	H	-4.3541	-0.24373	1.686072
O	-2.0824	-0.74638	-0.58407	H	-3.96255	1.038859	-1.05807
C	-2.80021	-1.81866	0.049793	H	-2.99859	-3.92143	-0.25003
C	-4.30384	-1.52206	-0.03396	H	-2.56888	-3.01911	-1.7171
C	-4.59199	-0.16578	0.612062	H	-0.44137	-2.87465	-0.87766
C	-3.71452	0.922437	0.007533	H	-5.92678	-2.24696	0.705156
C	-2.3746	-3.11131	-0.63736	H	-6.13833	0.983318	0.806401
O	-1.02964	-3.44701	-0.36183	H	-3.34035	2.784146	0.361891
Conformer 9_5							
Atom	X	Y	Z	Atom	X	Y	Z
C	-0.91853	0.132602	0.453399	O	5.404392	-1.92335	-1.35777
C	-2.24268	0.446583	0.088894	O	5.914617	0.678622	-0.34081
C	-2.5717	1.795879	-0.13136	O	3.555681	2.282083	0.018276
C	-1.5969	2.791439	0.042486	H	-0.62647	-0.88857	0.657105
C	-0.29319	2.473999	0.414782	H	-1.87622	3.823259	-0.13957
C	0.037034	1.121193	0.618098	H	-4.32261	0.829809	-2.17799
C	-3.24718	-0.67337	-0.17758	H	-5.7896	1.38649	-1.36537
C	-4.69431	-0.14866	-0.29737	H	-3.88852	3.078786	-1.24004
C	-4.7489	1.083708	-1.20075	H	-4.5134	2.588759	0.323573

C	-3.96279	2.224887	-0.55695	H	-4.31464	-3.63042	1.452562
C	-3.23359	-1.84191	0.858757	H	-6.31926	-3.21486	0.095511
C	-4.40612	-2.74225	0.833882	H	-5.45815	-1.52187	-1.79248
C	-5.49992	-2.49789	0.096545	H	-6.68513	-0.9056	-0.69628
C	-5.64934	-1.26516	-0.74139	H	-1.98868	-1.43785	-1.47886
O	-2.28832	-2.08171	1.595819	H	0.104449	4.951787	-0.64347
O	-2.94609	-1.30649	-1.44633	H	1.358467	5.291144	0.579103
O	1.323462	0.827391	1.028426	H	-0.34316	5.106126	1.082134
O	0.719152	3.377923	0.587434	H	1.844386	0.50778	-0.94386
C	0.428032	4.756383	0.385875	H	2.861253	-1.69468	-1.64894
H	-4.99621	0.154936	0.716665	H	4.893283	-1.51684	0.621994
C	2.195802	0.271252	0.074062	H	4.311922	0.447697	-1.6422
O	2.225815	-1.1314	0.264689	H	3.839003	0.708964	1.364775
C	3.148178	-1.82705	-0.59283	H	2.014895	-3.6504	-0.48969
C	4.571152	-1.28317	-0.40299	H	3.757987	-3.87726	-0.77675
C	4.589717	0.229765	-0.59698	H	2.688349	-2.91459	1.641673
C	3.569489	0.895586	0.313781	H	6.264834	-1.48414	-1.29727
C	3.021297	-3.30059	-0.21209	H	5.895246	1.644374	-0.39868
O	3.282598	-3.51131	1.163385	H	2.784112	2.663724	0.469819
Conformer 9_6							
Atom	X	Y	Z	Atom	X	Y	Z
C	-0.96389	0.179289	0.532124	O	5.489607	-1.90167	-0.87847
C	-2.27444	0.439346	0.085484	O	5.919252	0.734154	0.01362
C	-2.598	1.750151	-0.30732	O	3.530828	2.36538	0.116411
C	-1.63373	2.766718	-0.21578	H	-0.67453	-0.80834	0.864909
C	-0.34465	2.504402	0.240483	H	-1.90897	3.767897	-0.52839
C	-0.01753	1.186946	0.611231	H	-4.25712	0.531137	-2.29161
C	-3.26572	-0.71112	-0.0805	H	-5.75982	1.170448	-1.61614
C	-4.70774	-0.21557	-0.32393	H	-3.8719	2.881142	-1.61362
C	-4.72651	0.897773	-1.37157	H	-4.55993	2.575866	-0.02955
C	-3.97184	2.114223	-0.837	H	-4.39279	-3.45275	1.855281
C	-3.29592	-1.73694	1.096573	H	-6.33649	-3.22612	0.370629
C	-4.46026	-2.64811	1.128646	H	-5.40265	-1.76725	-1.67068
C	-5.52121	-2.5063	0.319938	H	-6.67739	-1.03259	-0.71002
C	-5.63931	-1.38599	-0.6678	H	-1.95571	-1.63444	-1.21918
O	-2.38941	-1.86475	1.906215	H	-0.42787	5.197849	0.590235
O	-2.91231	-1.49561	-1.24626	H	1.291634	5.334062	0.134248
O	1.255021	0.948894	1.096236	H	0.086997	4.84268	-1.08615
O	0.6574	3.430785	0.345849	H	1.874402	0.530394	-0.82903
C	0.370805	4.773319	-0.02926	H	2.913999	-1.5956	-1.40797
H	-5.05353	0.206828	0.631917	H	4.779666	-1.3846	1.009678
C	2.174429	0.348647	0.21669	H	4.408408	0.464499	-1.38756
O	2.204768	-1.04247	0.479576	H	3.742717	0.875023	1.565646
C	3.144026	-1.74702	-0.34068	H	1.940587	-3.50545	-0.08197

C	4.55523	-1.20902	-0.0543	H	3.332357	-3.42149	1.007596
C	4.608804	0.290786	-0.31741	H	4.592092	-3.6977	-1.02029
C	3.531667	0.999105	0.492208	H	6.338698	-1.44834	-0.77113
C	3.000738	-3.23987	-0.02813	H	5.923538	1.695742	-0.09391
O	3.68781	-4.04526	-0.96445	H	2.720148	2.760991	0.479088
Conformer 9_7							
Atom	X	Y	Z	Atom	X	Y	Z
C	-0.64565	0.611944	0.611595	O	4.414764	-2.93781	-1.05006
C	-1.93378	0.758507	0.080021	O	5.859409	-0.79314	0.0995
C	-2.25933	1.952193	-0.5929	O	4.437724	1.690202	0.172093
C	-1.30316	2.973473	-0.66936	H	-0.36606	-0.29477	1.123079
C	-0.01602	2.815261	-0.16071	H	-1.53433	3.903302	-1.18153
C	0.314962	1.603732	0.462159	H	-5.29064	0.979805	-1.96979
C	-2.86986	-0.44799	0.090916	H	-3.71214	0.305673	-2.39377
C	-4.30156	-0.08041	-0.35859	H	-3.49177	2.768746	-2.14856
C	-4.27197	0.813095	-1.59963	H	-4.27148	2.712194	-0.57809
C	-3.60986	2.148452	-1.25309	H	-4.07372	-2.78044	2.461563
C	-2.97672	-1.19068	1.458276	H	-5.87755	-2.96946	0.805929
C	-4.10003	-2.14836	1.578084	H	-4.81319	-1.90442	-1.41133
C	-5.08689	-2.23248	0.672932	H	-6.19925	-1.0637	-0.73004
C	-5.15657	-1.34303	-0.53171	H	-1.4332	-1.56644	-0.71475
O	-2.1621	-1.07824	2.362677	H	0.458222	5.006399	1.296814
O	-2.39146	-1.44338	-0.83822	H	2.051875	5.239167	0.528735
O	1.627247	1.386209	0.886939	H	1.7572	3.812508	1.56686
O	0.91721	3.80828	-0.35416	H	2.19868	0.716266	-0.97695
C	1.312453	4.495619	0.834488	H	2.241501	-1.5354	-1.57885
H	-4.74335	0.50029	0.466092	H	3.910202	-2.31079	0.87252
C	2.317875	0.455059	0.088224	H	4.476033	-0.33873	-1.38116
O	1.788057	-0.83524	0.330086	H	3.853716	0.234472	1.555159
C	2.33734	-1.83692	-0.5235	H	2.024008	-3.92169	-0.84825
C	3.825801	-1.98836	-0.17814	H	1.507083	-3.33844	0.759322
C	4.510643	-0.62581	-0.31697	H	-0.30452	-3.68502	-0.69545
C	3.792987	0.463254	0.480678	H	5.371659	-2.86355	-0.91966
C	1.527559	-3.10219	-0.31409	H	6.28173	0.074749	0.0296
O	0.209075	-2.87703	-0.81991	H	3.930429	2.394274	0.598962

9. ECD calculation data of compound 11

Figure S181. The energies and equilibrium populations of stable conformers 11_1 - 11_9

No	Conformer	Structure	Gibb free energy (Hartree)	Population (%)
1	11_1	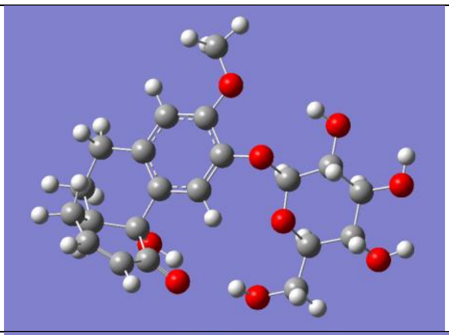	-1492.48498	76.39
2	11_2	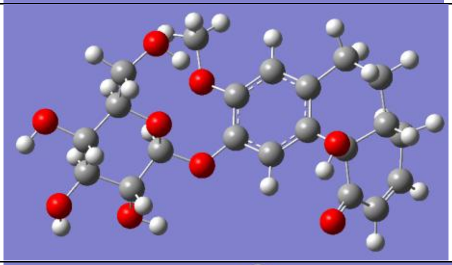	-1492.479082	0.15
3	11_3	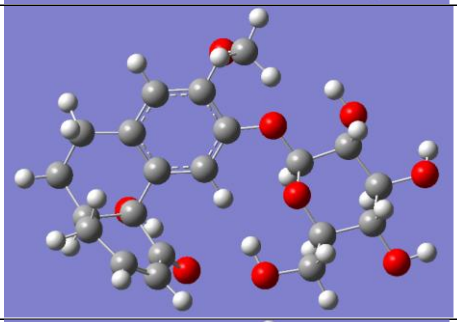	-1492.479811	0.32
4	11_4	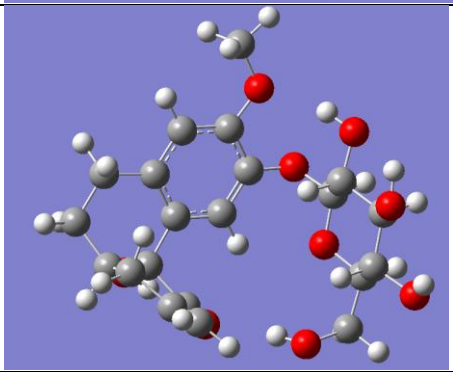	-1492.483549	16.76

No	Conformer	Structure	Gibb free energy (Hartree)	Population (%)
5	11_5	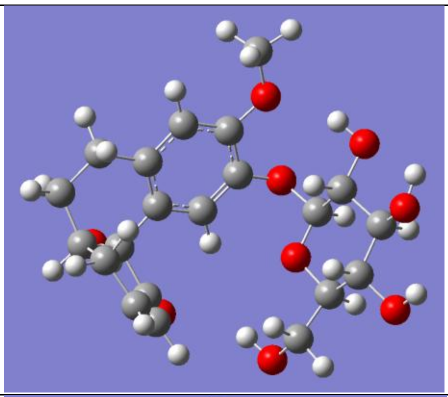	-1492.482066	3.48
6	11_6	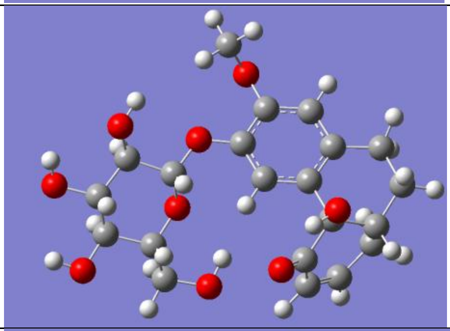	-1492.478996	0.13
7	11_7	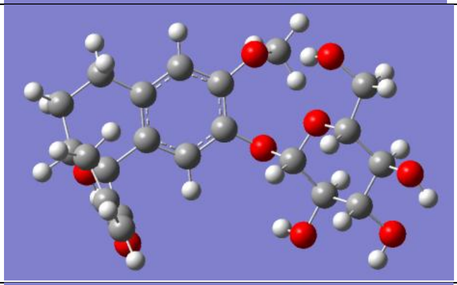	-1492.480347	0.56
8	11_8	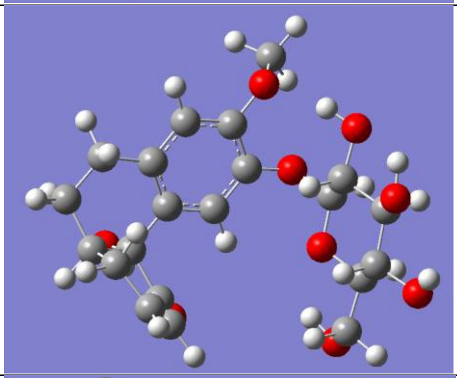	-1492.481574	2.06
9	11_9	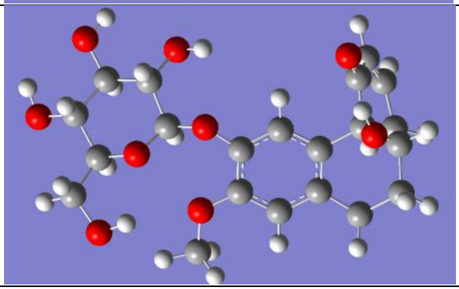	-1492.479114	0.15

Figure S182. Cartesian Coordinates of low energy conformers 11_1 - 11_9

Conformer 11_1							
Atom	X	Y	Z	Atom	X	Y	Z
C	-0.78741	0.213506	0.346541	O	5.167652	-2.09639	-0.88252
C	-2.09366	0.257746	-0.15945	O	5.932353	0.376024	0.262514
C	-2.63738	1.498914	-0.52278	O	3.82824	2.358731	0.283856
C	-1.86482	2.661756	-0.35996	H	-0.32843	-0.72478	0.62241
C	-0.55776	2.612274	0.127555	H	-2.29786	3.613064	-0.65001
C	-0.02153	1.358162	0.478542	H	-5.7322	0.400793	-1.66968
C	-2.83722	-1.05902	-0.44065	H	-4.22658	-0.00802	-2.49037
C	-4.36084	-0.83488	-0.52216	H	-4.68514	2.101024	-0.32671
C	-4.65079	0.289966	-1.52714	H	-4.03363	2.325803	-1.92983
C	-4.04303	1.628599	-1.08378	H	-3.02243	-2.90112	2.595304
C	-2.49422	-2.10517	0.640609	H	-5.10791	-1.62024	2.818736
C	-3.35457	-2.21598	1.821127	H	-4.75362	0.447241	1.206502
C	-4.49876	-1.51906	1.922039	H	-6.07435	-0.62098	0.793922
C	-4.98042	-0.57304	0.863885	H	-1.60931	-2.08572	-1.52949
O	-1.50216	-2.81865	0.496917	H	0.578209	5.671567	0.127457
O	-2.42481	-1.58492	-1.69977	H	-1.10461	5.241066	0.532776
O	1.275889	1.303665	0.958092	H	-0.49923	5.017287	-1.13476
O	0.268764	3.686202	0.282225	H	2.00859	0.785107	-0.89693
C	-0.23098	4.967989	-0.07207	H	2.700749	-1.35732	-1.47945
H	-4.78123	-1.77119	-0.9085	H	4.458936	-1.61878	1.018354
C	2.180272	0.565944	0.17042	H	4.512449	0.412375	-1.25366
O	2.006133	-0.81805	0.410392	H	3.687897	0.794019	1.662543
C	2.854051	-1.62567	-0.42231	H	2.489073	-3.3372	0.827465
C	4.313843	-1.34165	-0.03892	H	3.151797	-3.70552	-0.7764
C	4.60005	0.154823	-0.18516	H	0.469768	-3.05653	-0.16319
C	3.585446	0.992855	0.584925	H	6.06215	-1.75985	-0.72617
C	2.431931	-3.07998	-0.24118	H	6.098424	1.326068	0.18562
O	1.153129	-3.34607	-0.78516	H	3.083631	2.858846	0.651235
Conformer 11_2							
Atom	X	Y	Z	Atom	X	Y	Z
C	1.195905	-0.68451	-0.06931	O	-5.9539	0.344371	1.026261
C	2.35445	0.080313	0.132089	O	-5.5176	-2.32323	0.135761
C	2.362757	1.418211	-0.28986	O	-3.27183	-2.62474	-1.61041
C	1.231441	1.932413	-0.94037	H	1.134237	-1.70362	0.296484
C	0.099859	1.15561	-1.18784	H	1.250462	2.965119	-1.27206
C	0.078185	-0.17291	-0.71112	H	5.577529	2.291914	0.709799
C	3.534322	-0.49952	0.929553	H	4.331283	1.876464	1.885147
C	4.844397	0.247233	0.601839	H	3.999428	2.557679	-1.07107
C	4.634399	1.748614	0.84192	H	3.21914	3.295956	0.303857
C	3.561096	2.331006	-0.08879	H	4.496423	-3.46924	-0.76553
C	3.69797	-2.00473	0.637608	H	5.885857	-1.86854	-2.00888

C	4.515321	-2.41407	-0.5095	H	4.756498	0.471739	-1.57245
C	5.282525	-1.52982	-1.16813	H	6.37523	0.290723	-0.93915
C	5.346305	-0.07214	-0.82034	H	2.891555	-1.20907	2.591522
O	3.143773	-2.81567	1.374603	H	-2.14362	3.110029	-2.54206
O	3.286904	-0.35977	2.324611	H	-0.4015	3.454161	-2.63573
O	-0.99019	-1.03999	-0.86372	H	-1.24677	3.491773	-1.04993
O	-0.99607	1.593212	-1.87607	H	-2.62331	0.009386	-1.57171
C	-1.18939	2.996904	-2.02582	H	-3.92875	1.396514	-0.286
H	5.589007	-0.12493	1.31614	H	-4.38509	-0.75427	1.848915
C	-2.30629	-0.56233	-0.69071	H	-4.98416	-0.77229	-1.13865
O	-2.35742	0.256748	0.460304	H	-2.8482	-2.31257	0.412609
C	-3.64559	0.86368	0.635053	H	-3.27171	1.347793	2.699861
C	-4.6686	-0.2427	0.914574	H	-4.4064	2.442003	1.881033
C	-4.63238	-1.27002	-0.21954	H	-1.65865	2.271623	1.283571
C	-3.21275	-1.77384	-0.4742	H	-6.58429	-0.39044	1.044586
C	-3.47793	1.880083	1.758009	H	-5.45152	-2.98809	-0.56445
O	-2.44791	2.807143	1.451648	H	-2.37488	-2.94934	-1.7706
Conformer 11_3							
Atom	X	Y	Z	Atom	X	Y	Z
C	-0.78575	0.440104	-0.42905	O	4.934619	-2.33615	-0.0601
C	-2.18815	0.384818	-0.44765	O	5.769616	0.279567	-0.77124
C	-2.93465	1.512591	-0.07407	O	3.649863	2.03117	-1.5617
C	-2.24415	2.671064	0.310095	H	-0.2218	-0.43944	-0.70915
C	-0.85598	2.731963	0.33323	H	-2.78954	3.565769	0.598304
C	-0.11602	1.595243	-0.04246	H	-6.12857	0.151817	-0.28029
C	-2.89377	-0.87945	-0.96701	H	-5.14721	0.360957	-1.72884
C	-4.29639	-1.01938	-0.34121	H	-4.76428	1.608429	1.025062
C	-5.09851	0.255253	-0.64132	H	-4.83735	2.402398	-0.52628
C	-4.45349	1.506393	-0.0243	H	-1.43168	-3.48386	0.940842
C	-2.05409	-2.13294	-0.65439	H	-3.24862	-2.85572	2.47971
C	-2.17514	-2.73851	0.675546	H	-3.98168	-0.45255	1.747357
C	-3.17718	-2.38641	1.499682	H	-5.19602	-1.68737	1.521356
C	-4.21402	-1.35414	1.161546	H	-2.29424	-1.31967	-2.73087
O	-1.3054	-2.57247	-1.52308	H	0.835629	4.917704	2.065635
O	-3.03784	-0.79627	-2.37902	H	1.197007	3.169749	2.006843
O	1.260131	1.726366	-0.00253	H	-0.32479	3.746471	2.748529
O	-0.23331	3.912149	0.666128	H	1.712413	0.196308	-1.31142
C	0.404004	3.921822	1.946806	H	2.381398	-1.9784	-0.54608
H	-4.77361	-1.86426	-0.85301	H	4.548821	-0.76493	1.252556
C	2.05184	0.625437	-0.35433	H	4.109648	-0.4956	-1.75113
O	1.970044	-0.34104	0.677835	H	3.780782	1.573833	0.473021
C	2.726405	-1.5344	0.400187	H	2.827946	-2.07837	2.477528
C	4.210432	-1.16864	0.283765	H	2.899122	-3.45229	1.352974
C	4.394693	-0.07761	-0.77147	H	0.605374	-1.8525	1.688505

C	3.492334	1.119568	-0.48663	H	5.829708	-2.04202	-0.28405
C	2.413881	-2.49271	1.544119	H	5.873477	0.990785	-1.41928
O	1.020633	-2.72652	1.65622	H	3.073318	2.787905	-1.38451
Conformer 11_4							
Atom	X	Y	Z	Atom	X	Y	Z
C	-0.76877	0.314015	-1.08097	O	4.511662	-1.93516	1.016833
C	-2.02951	0.227752	-0.47253	O	3.848952	0.51569	2.2879
C	-2.59578	1.380701	0.089178	O	2.854789	2.536629	0.546042
C	-1.85152	2.573195	0.096849	H	-0.3241	-0.54368	-1.56428
C	-0.56657	2.631619	-0.44018	H	-2.29154	3.454795	0.550585
C	-0.03102	1.484881	-1.06054	H	-5.1796	0.071713	-0.54434
C	-2.7823	-1.11404	-0.48918	H	-5.6082	0.017226	1.164096
C	-3.86436	-1.15983	0.608546	H	-3.89853	1.580767	1.781115
C	-4.7639	0.077653	0.467589	H	-4.55678	2.225911	0.300118
C	-3.98545	1.381417	0.703937	H	-0.56165	-3.35421	1.146962
C	-1.75241	-2.24415	-0.29832	H	-1.83619	-2.60039	3.109722
C	-1.40421	-2.67585	1.054225	H	-4.05112	-1.59584	2.727196
C	-2.11532	-2.25693	2.114948	H	-2.90036	-0.32811	2.388709
C	-3.26408	-1.29911	2.022219	H	-2.76753	-1.75496	-2.30141
O	-1.22442	-2.72909	-1.30295	H	-1.1481	5.276062	-0.39982
O	-3.42811	-1.31121	-1.74194	H	0.527898	5.675582	0.062577
O	1.206139	1.576344	-1.67182	H	-0.52942	4.813346	1.213943
O	0.253421	3.730938	-0.39714	H	3.151008	1.149432	-1.71035
C	-0.26721	4.935218	0.155934	H	4.013794	-1.18042	-1.40082
H	-4.45909	-2.05735	0.401009	H	2.493505	-1.45423	1.229445
C	2.278457	0.838322	-1.11797	H	4.571773	0.601603	0.342478
O	2.031254	-0.54707	-1.28075	H	1.595722	0.913845	0.918514
C	3.094118	-1.40084	-0.8321	H	3.521747	-3.48592	-0.82569
C	3.381984	-1.15476	0.654876	H	2.557258	-2.95015	-2.21779
C	3.65477	0.327211	0.890884	H	0.743675	-3.04487	-0.93253
C	2.507834	1.174024	0.360501	H	4.753915	-1.65385	1.910737
C	2.689	-2.84531	-1.13215	H	3.938233	1.470317	2.421134
O	1.53936	-3.27335	-0.42548	H	2.116666	3.072486	0.208574
Conformer 11_5							
Atom	X	Y	Z	Atom	X	Y	Z
C	-0.84408	0.375249	-1.09221	O	4.41167	-2.33066	1.162924
C	-2.06064	0.504805	-0.41215	O	4.670784	0.442726	1.716561
C	-2.32965	1.700842	0.270275	O	3.575672	2.190054	-0.23554
C	-1.35484	2.713147	0.28895	H	-0.61933	-0.51312	-1.66445
C	-0.12162	2.552039	-0.34459	H	-1.57654	3.63312	0.819076
C	0.128958	1.357484	-1.04592	H	-5.50437	0.91699	1.453829
C	-3.09591	-0.62974	-0.49884	H	-5.16457	1.058964	-0.26955
C	-4.10768	-0.55065	0.661143	H	-3.44302	1.976205	2.081331
C	-4.70604	0.863907	0.704429	H	-4.0332	2.921488	0.738831

C	-3.6415	1.931487	1.001209	H	-1.48368	-3.55389	0.725229
C	-2.35355	-1.98315	-0.49881	H	-2.42894	-2.73836	2.849883
C	-2.1023	-2.66225	0.773418	H	-2.85979	-0.17621	2.419717
C	-2.6353	-2.20952	1.920816	H	-4.28473	-1.13215	2.745254
C	-3.48798	-0.97884	2.006529	H	-3.30443	-1.03858	-2.36613
O	-1.9692	-2.44531	-1.57319	H	1.578239	5.294718	0.213945
O	-3.82806	-0.53753	-1.7172	H	0.47393	4.558993	1.407086
O	1.311765	1.194724	-1.74499	H	-0.17596	5.258548	-0.10605
O	0.890277	3.478893	-0.33485	H	3.031801	0.21912	-1.99354
C	0.664329	4.71285	0.338142	H	3.322305	-2.17707	-1.16805
H	-4.90236	-1.2641	0.412315	H	2.719384	-1.2365	1.685251
C	2.292268	0.337057	-1.18879	H	4.776812	-0.14227	-0.27396
O	1.705711	-0.90329	-0.84554	H	2.194831	1.052122	0.833655
C	2.620122	-1.90189	-0.36675	H	1.100434	-2.81243	0.834056
C	3.414989	-1.37484	0.840028	H	2.449113	-3.88375	0.407572
C	4.033479	-0.01513	0.530968	H	0.37407	-3.07847	-1.34205
C	2.959276	0.947992	0.048143	H	4.986086	-1.90751	1.81761
C	1.775307	-3.11646	0.017063	H	4.951765	1.351677	1.53663
O	1.066679	-3.69353	-1.05904	H	2.853837	2.797443	-0.4689
Conformer 11_6							
Atom	X	Y	Z	Atom	X	Y	Z
C	0.780044	0.470798	0.343073	O	-4.89967	-2.39948	0.14069
C	2.182372	0.405226	0.328421	O	-5.78476	0.262024	0.575701
C	2.92403	1.465089	-0.21534	O	-3.72317	2.10931	1.245268
C	2.228843	2.574304	-0.71699	H	0.220525	-0.36294	0.745541
C	0.841386	2.645534	-0.70424	H	2.76967	3.41374	-1.14587
C	0.103536	1.569824	-0.17527	H	6.117579	0.12171	0.089573
C	2.897274	-0.78594	0.989585	H	5.172525	0.535676	1.518269
C	4.283321	-1.01729	0.353781	H	4.717339	1.385463	-1.37446
C	5.097004	0.280725	0.457262	H	4.845261	2.391858	0.044226
C	4.440245	1.437283	-0.31211	H	1.381613	-3.61288	-0.51862
C	2.045735	-2.06444	0.866551	H	3.162726	-3.21056	-2.16799
C	2.134552	-2.84529	-0.37094	H	3.919693	-0.73601	-1.78457
C	3.117072	-2.6133	-1.25864	H	5.136442	-1.93521	-1.42186
C	4.163999	-1.55116	-1.08764	H	2.34057	-0.98702	2.809682
O	1.314756	-2.37705	1.802984	H	-0.79816	5.455932	-0.91126
O	3.077009	-0.51861	2.374878	H	0.440818	5.080777	0.319414
O	-1.27236	1.694418	-0.22105	H	-1.10194	4.183906	0.303845
O	0.212621	3.734535	-1.26372	H	-1.7429	0.293078	1.223062
C	-0.3391	4.656621	-0.32602	H	-2.36637	-1.96233	0.64882
H	4.77042	-1.78824	0.963592	H	-4.50216	-0.95566	-1.30813
C	-2.06112	0.623042	0.219974	H	-4.14118	-0.39317	1.664351
O	-1.94268	-0.4384	-0.71039	H	-3.77928	1.458742	-0.74029
C	-2.69171	-1.61087	-0.34236	H	-2.73433	-2.33993	-2.36358

C	-4.18269	-1.25909	-0.29734	H	-2.83056	-3.60727	-1.12173
C	-4.40669	-0.07532	0.642317	H	-0.53336	-2.03405	-1.55453
C	-3.51302	1.102838	0.265262	H	-5.80548	-2.10086	0.308213
C	-2.34374	-2.66605	-1.38629	H	-5.90838	1.036803	1.142369
O	-0.94776	-2.90172	-1.44093	H	-3.23105	2.892993	0.966359
Conformer 11_7							
Atom	X	Y	Z	Atom	X	Y	Z
C	-1.24318	-0.27683	-0.84861	O	5.533398	-0.81992	1.668517
C	-2.47749	0.219807	-0.41157	O	4.994611	-2.64226	-0.43936
C	-2.58999	1.591824	-0.1201	O	2.277686	-2.67549	-1.31845
C	-1.46668	2.409172	-0.28068	H	-1.12708	-1.32732	-1.09481
C	-0.23344	1.917615	-0.70977	H	-1.53067	3.473921	-0.07449
C	-0.12662	0.540773	-0.98277	H	-5.46853	1.290642	-0.78697
C	-3.71232	-0.69637	-0.3643	H	-5.92293	1.669985	0.87298
C	-4.76789	-0.15509	0.622173	H	-3.74986	2.52328	1.415039
C	-5.10662	1.293801	0.245335	H	-4.06848	3.146441	-0.18216
C	-3.8859	2.21661	0.368617	H	-2.82323	-3.45335	1.69177
C	-3.30842	-2.1321	0.027952	H	-3.65196	-1.93916	3.440896
C	-3.23882	-2.47955	1.450504	H	-5.16187	-0.12342	2.758251
C	-3.71183	-1.64765	2.393587	H	-3.56609	0.455556	2.346709
C	-4.31348	-0.30837	2.087253	H	-3.92576	-1.55505	-2.06538
O	-3.05751	-2.93975	-0.86283	H	0.786065	3.082698	-2.85502
O	-4.30649	-0.75709	-1.65626	H	2.162899	3.762368	-1.94552
O	1.062176	-0.04145	-1.42253	H	2.075871	2.003149	-2.24018
O	0.801707	2.814202	-0.78522	H	1.412344	-0.84024	0.447074
C	1.495229	2.904063	-2.03835	H	3.07947	0.068493	1.92468
H	-5.66	-0.77653	0.477098	H	5.231845	-0.07345	-0.25358
C	1.96499	-0.41996	-0.40975	H	3.471513	-2.26582	0.922102
O	2.692893	0.721945	-0.01452	H	3.417966	-1.01084	-1.86304
C	3.616504	0.484108	1.057785	H	4.751658	2.241346	0.572249
C	4.671833	-0.52072	0.584379	H	4.825322	1.773086	2.283611
C	3.981667	-1.78598	0.070148	H	2.473956	2.726573	1.041155
C	2.927788	-1.45799	-0.98538	H	6.081114	-1.56434	1.37942
C	4.159099	1.862298	1.421909	H	4.545708	-3.42225	-0.79543
O	3.114066	2.746594	1.771342	H	1.633829	-2.47688	-2.0123
Conformer 11_8							
Atom	X	Y	Z	Atom	X	Y	Z
C	-0.86414	0.286551	-0.92765	O	4.621308	-1.98936	1.315666
C	-2.12365	0.352026	-0.31523	O	4.022553	0.562629	2.414249
C	-2.52066	1.539017	0.328677	O	2.972383	2.449102	0.600368
C	-1.6351	2.624846	0.347262	H	-0.54374	-0.60773	-1.4469
C	-0.38171	2.54977	-0.24821	H	-1.91292	3.55285	0.839527
C	0.01273	1.364462	-0.88807	H	-5.65593	0.537459	1.45551
C	-3.10611	-0.82493	-0.45679	H	-5.30854	0.698516	-0.26487

C	-4.14604	-0.82345	0.682454	H	-3.69471	1.749963	2.103087
C	-4.8479	0.541109	0.714708	H	-4.31672	2.64078	0.738676
C	-3.8665	1.681654	1.019721	H	-1.39413	-3.69172	0.75573
C	-2.34678	-2.16774	-0.47708	H	-2.35203	-2.90561	2.878211
C	-2.04458	-2.82323	0.799093	H	-2.94031	-0.37319	2.453044
C	-2.59025	-2.39165	1.948445	H	-4.3054	-1.41702	2.765872
C	-3.51403	-1.21255	2.033619	H	-3.29902	-1.26445	-2.31302
O	-2.02154	-2.64543	-1.56024	H	1.348248	5.184505	-1.13444
O	-3.80223	-0.70732	-1.69167	H	1.094077	3.734003	-2.15079
O	1.235606	1.329322	-1.5392	H	-0.29417	4.778025	-1.70768
O	0.483888	3.628555	-0.14829	H	3.172046	0.873349	-1.57663
C	0.664094	4.368002	-1.37129	H	4.013863	-1.36798	-1.15472
H	-4.88133	-1.59315	0.418021	H	2.62178	-1.46265	1.574193
C	2.311071	0.64179	-0.93399	H	4.660217	0.498485	0.439627
O	2.035226	-0.75027	-0.95242	H	1.71124	0.869929	1.118674
C	3.138377	-1.56619	-0.51709	H	1.830376	-3.2099	-0.07345
C	3.488993	-1.22565	0.935316	H	3.514298	-3.67184	-0.40226
C	3.768962	0.26875	1.048052	H	1.755239	-2.65631	-2.35457
C	2.600324	1.086691	0.507868	H	4.927871	-1.61487	2.154425
C	2.702853	-3.01191	-0.71798	H	4.083295	1.527934	2.470017
O	2.427091	-3.29444	-2.07561	H	2.172429	2.984066	0.440908
Conformer 11_9							
Atom	X	Y	Z	Atom	X	Y	Z
C	1.366559	-0.30537	0.707551	O	-6.14048	-0.69643	-0.89194
C	2.582457	0.302619	0.361775	O	-4.68971	-3.11025	-0.44446
C	2.557236	1.592532	-0.18813	O	-1.85408	-2.77986	-0.41118
C	1.320594	2.224795	-0.38339	H	1.353138	-1.29133	1.159788
C	0.111349	1.620779	-0.03881	H	1.313163	3.225707	-0.80109
C	0.143364	0.318552	0.511415	H	5.268097	1.909866	0.964473
C	3.916002	-0.39108	0.686232	H	5.973117	2.06205	-0.64394
C	5.051656	0.139812	-0.21353	H	3.844669	2.417355	-1.68834
C	5.112003	1.668639	-0.09101	H	3.770974	3.363247	-0.2241
C	3.82156	2.331858	-0.59267	H	3.805678	-3.57368	-0.8912
C	3.783587	-1.91954	0.527506	H	4.7405	-2.27283	-2.75437
C	4.032788	-2.51665	-0.78865	H	5.830145	-0.13442	-2.22273
C	4.560073	-1.79612	-1.79222	H	4.120832	0.226765	-2.19191
C	4.904685	-0.34168	-1.67055	H	3.91553	-0.88633	2.540278
O	3.473587	-2.59255	1.507044	H	-0.79065	3.465741	-1.83127
O	4.274394	-0.13211	2.039547	H	-2.25765	3.720757	-0.83446
O	-0.97277	-0.37434	0.94907	H	-0.66034	4.247106	-0.22783
O	-1.10991	2.205261	-0.19398	H	-1.91781	-0.1101	-0.86975
C	-1.19307	3.486047	-0.81092	H	-4.00725	0.838052	-1.09048
H	5.977979	-0.28505	0.191967	H	-5.27961	-1.03173	0.976877
C	-2.14078	-0.40765	0.164865	H	-3.81003	-1.64263	-1.62138

O	-3.09583	0.459293	0.740957	H	-2.89472	-2.08025	1.260748
C	-4.28179	0.571723	-0.05728	H	-5.41092	1.441352	1.556413
C	-4.99696	-0.78218	-0.05875	H	-5.94426	1.942229	-0.06349
C	-4.03075	-1.85677	-0.56192	H	-3.41896	2.607675	0.976849
C	-2.71056	-1.82882	0.206144	H	-6.47065	-1.6014	-0.98987
C	-5.06625	1.7279	0.550109	H	-4.0506	-3.78408	-0.71698
O	-4.26254	2.89688	0.594102	H	-1.04413	-2.81772	0.115769

10. ECD calculation data of compound 12

Figure S183. The energies and equilibrium populations of stable conformers 12_1 - 12_8

No	Conformer	Structure	Gibb free energy (Hartree)	Population (%)
1	12_1	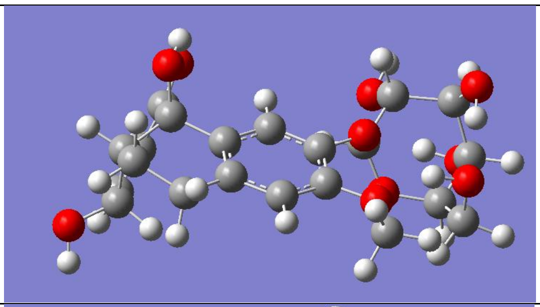	-1568.093183	1.63
2	12_2	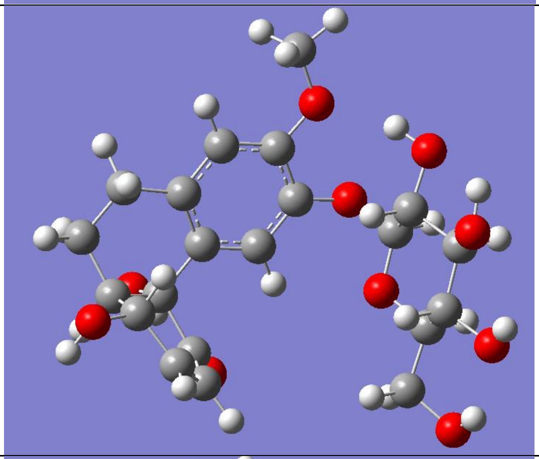	-1568.095683	23.02
3	12_3	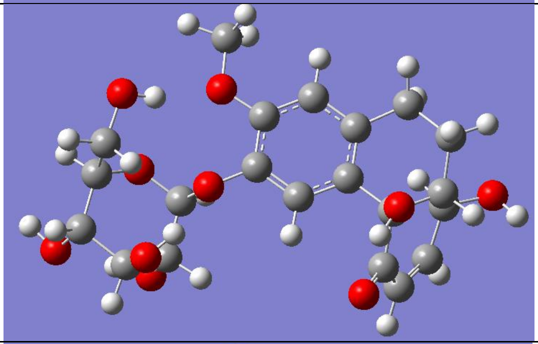	-1568.089636	0.04
4	12_4	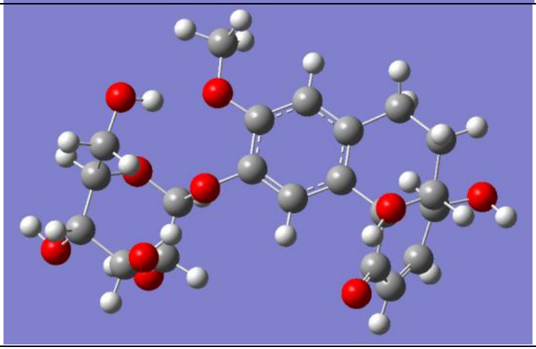	-1568.089636	0.04

No	Conformer	Structure	Gibb free energy (Hartree)	Population (%)
5	12_5	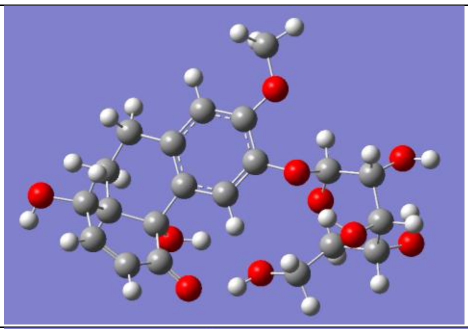	-1568.088579	0.01
6	12_6	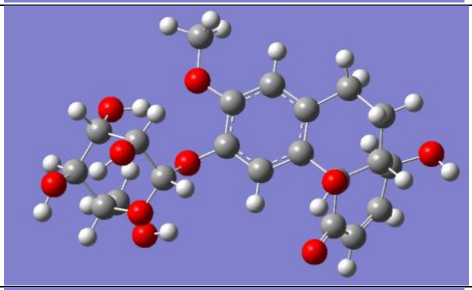	-1568.088871	0.02
7	12_7	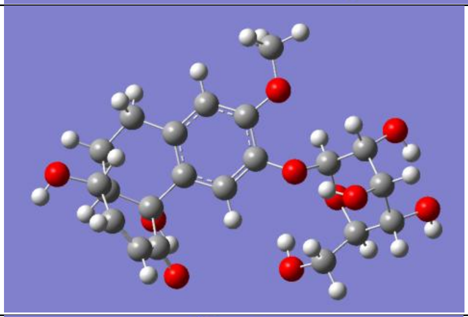	-1568.088249	0.01
8	12_8	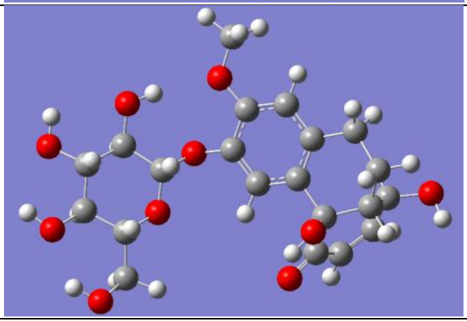	-1568.0968	75.24

Figure S184. Cartesian Coordinates of low energy conformers 12_1 - 12_8

Conformer 12_1							
Atom	X	Y	Z	Atom	X	Y	Z
C	-0.77846	-0.18751	-0.68555	O	4.581734	-0.06297	-1.75792
C	-2.10714	0.251191	-0.55148	O	2.991598	-2.97994	-0.17441
C	-2.35502	1.602578	-0.26822	O	-5.80222	-0.20438	1.884227
C	-1.26782	2.477194	-0.10692	H	-0.57672	-1.21667	-0.96255
C	0.044821	2.030149	-0.21195	H	-1.46868	3.521517	0.107204
C	0.293268	0.673296	-0.49919	H	-4.94946	1.218859	-1.66179
C	-3.26983	-0.71257	-0.85184	H	-5.82987	1.512152	-0.15879
C	-4.56685	-0.24503	-0.15675	H	-3.83713	3.103649	-0.66852
C	-4.8666	1.20082	-0.57141	H	-3.91577	2.43209	0.940097
C	-3.76028	2.160868	-0.1142	H	-2.87024	-3.54203	1.261123
C	-2.91487	-2.1469	-0.4146	H	-4.22963	-2.13068	2.762899
C	-3.22546	-2.56141	0.958561	H	-3.78827	0.314775	1.787765
C	-3.97404	-1.78908	1.760567	H	-2.99517	-1.49897	-2.5809
C	-4.48827	-0.42691	1.368998	H	1.995732	4.630857	0.15704
O	-2.40133	-2.90762	-1.22953	H	0.464546	4.43994	1.05768
O	-3.51316	-0.74205	-2.25344	H	0.438358	4.666782	-0.71777
O	1.605433	0.281022	-0.66122	H	1.212265	-1.46477	0.379812
O	1.153025	2.818212	-0.06412	H	4.253562	-0.09746	2.440043
C	0.986787	4.219473	0.119166	H	5.912072	-0.2867	0.497469
H	-5.3728	-0.89811	-0.51092	H	5.086988	-1.94103	-1.22745
C	2.068797	-0.84245	0.109286	H	2.583715	-1.69976	-1.78901
O	2.621081	-0.44815	1.331604	H	5.054619	1.900684	1.466722
C	3.976914	0.056914	1.390621	H	3.327634	2.046997	1.840098
C	4.956559	-0.81643	0.578684	H	2.890972	2.042266	-0.32388
C	4.452928	-1.12673	-0.8501	H	4.413186	-2.55817	1.214293
C	3.00798	-1.67267	-0.77868	H	4.331924	0.770819	-1.30551
C	4.052703	1.570942	1.167896	H	3.512256	-3.5677	-0.73892
O	3.853103	2.024477	-0.16614	H	-5.73415	-0.03103	2.831957
O	5.222393	-2.02395	1.283998				
Conformer 12_2							
Atom	X	Y	Z	Atom	X	Y	Z
C	-0.69127	0.357404	-1.15913	O	4.250766	0.680385	2.230654
C	-1.98006	0.399117	-0.60473	O	3.247812	2.550012	0.327866
C	-2.41168	1.572045	0.03427	O	-4.49457	-1.38599	2.622194
C	-1.53658	2.666265	0.121	H	-0.33767	-0.51962	-1.68684
C	-0.25119	2.613105	-0.41234	H	-1.88321	3.566259	0.617004
C	0.175095	1.436197	-1.0599	H	-5.17562	0.728257	-0.69471
C	-2.94723	-0.77839	-0.81466	H	-5.58076	0.534583	1.014885
C	-4.03732	-0.79441	0.279248	H	-4.23092	2.661964	0.371045
C	-4.75313	0.561861	0.300179	H	-3.67315	1.753508	1.7533
C	-3.79017	1.701164	0.661746	H	-1.30442	-3.67359	0.427185

C	-2.18727	-2.11916	-0.82384	H	-2.39509	-2.91471	2.522388
C	-1.94849	-2.79947	0.456105	H	-2.8693	-0.36135	2.059577
C	-2.5527	-2.38913	1.582352	H	-3.05732	-1.18038	-2.68708
C	-3.46335	-1.1913	1.655253	H	-0.02553	4.769247	1.274051
O	-1.82372	-2.59015	-1.89622	H	-0.61737	5.292195	-0.33183
O	-3.59449	-0.64593	-2.07455	H	1.087844	5.555188	0.121472
O	1.412602	1.426343	-1.68118	H	3.322881	0.885496	-1.76373
O	0.657458	3.641483	-0.35182	H	4.097755	-1.3755	-1.26987
C	0.237291	4.879245	0.215202	H	2.726169	-1.28717	1.465719
H	-4.75267	-1.57546	-0.01488	H	4.852657	0.528112	0.24812
C	2.472062	0.707405	-1.09065	H	1.9355	1.056643	0.962606
O	2.143841	-0.67227	-1.05605	H	2.340933	-3.1152	-1.73852
C	3.220057	-1.50868	-0.61763	H	1.926791	-3.12582	-0.01729
C	3.601622	-1.11762	0.818384	H	4.273932	-3.62211	0.250393
C	3.961955	0.360026	0.876011	H	4.998732	-1.55921	2.086493
C	2.81908	1.201322	0.321496	H	4.384021	1.63895	2.258914
C	2.748766	-2.96155	-0.73516	H	2.491417	3.083317	0.026702
O	3.80792	-3.87995	-0.56075	H	-5.06919	-2.09317	2.296024
O	4.694968	-1.92683	1.243548				
Conformer 12_3							
Atom	X	Y	Z	Atom	X	Y	Z
C	0.779081	-0.21197	0.586522	O	-3.24973	-1.81291	1.734133
C	2.09118	0.280468	0.494532	O	-3.11693	-2.29747	-1.92421
C	2.289279	1.609669	0.090263	O	5.904031	-0.17702	-1.79191
C	1.175409	2.405248	-0.22238	H	0.592126	-1.22565	0.925175
C	-0.1238	1.91032	-0.13715	H	1.342928	3.433227	-0.5248
C	-0.3177	0.573408	0.269284	H	4.815523	1.50527	1.658337
C	3.276852	-0.58835	0.948057	H	5.762445	1.70042	0.178645
C	4.589303	-0.11634	0.28519	H	3.668996	3.219692	0.423257
C	4.79496	1.376122	0.572526	H	3.87941	2.401203	-1.1039
C	3.672615	2.225668	-0.03895	H	3.124274	-3.60581	-0.92351
C	3.021219	-2.07387	0.625718	H	4.498269	-2.25082	-2.48204
C	3.425877	-2.58929	-0.6883	H	3.943373	0.225734	-1.76153
C	4.179387	-1.85313	-1.52038	H	2.935183	-1.22931	2.724641
C	4.62409	-0.44552	-1.22183	H	-2.12868	4.341963	-1.00562
O	2.503364	-2.7865	1.479819	H	-0.5494	4.04177	-1.78577
O	3.436542	-0.47901	2.357707	H	-0.62106	4.595301	-0.08387
O	-1.59352	0.059567	0.419631	H	-1.6757	-0.16306	-1.65611
O	-1.24957	2.629482	-0.41755	H	-5.34784	0.907172	-0.62475
C	-1.11231	3.981293	-0.84742	H	-5.63413	-1.10464	1.0318
H	5.394604	-0.69085	0.764315	H	-4.13693	-3.02927	0.358215
C	-2.32317	-0.31659	-0.78974	H	-1.79575	-2.36073	-0.38457
O	-3.4277	0.508845	-0.9862	H	-3.63924	0.681571	1.891058
C	-4.54507	0.395885	-0.07687	H	-5.28248	1.299008	1.72576

C	-4.96496	-1.06168	0.161241	H	-3.01133	2.417857	0.485063
C	-3.76529	-2.00124	0.425728	H	-6.30272	-0.98429	-1.28827
C	-2.69684	-1.80601	-0.66794	H	-2.5085	-1.19023	1.67507
C	-4.31781	1.208825	1.211951	H	-4.0523	-2.04672	-2.02081
O	-3.87436	2.514786	0.921356	H	6.551464	-0.72472	-1.32551
O	-5.62418	-1.60747	-0.99661				
Conformer 12_4							
Atom	X	Y	Z	Atom	X	Y	Z
C	0.779444	-0.21243	0.586553	O	-3.25011	-1.81352	1.733572
C	2.091441	0.280272	0.494663	O	-3.11849	-2.29629	-1.9251
C	2.289282	1.609514	0.09043	O	5.903989	-0.17637	-1.79224
C	1.175266	2.404873	-0.22229	H	0.592603	-1.22619	0.925058
C	-0.12388	1.909694	-0.13716	H	1.342642	3.432886	-0.52467
C	-0.31748	0.572717	0.269216	H	4.815736	1.505403	1.658375
C	3.277281	-0.58836	0.948106	H	5.76244	1.700856	0.178589
C	4.589588	-0.11609	0.285089	H	3.668704	3.219753	0.423708
C	4.795049	1.376369	0.57255	H	3.87922	2.401608	-1.10362
C	3.672499	2.225815	-0.0387	H	3.124898	-3.60567	-0.92369
C	3.021885	-2.0739	0.625691	H	4.498451	-2.25033	-2.48234
C	3.426382	-2.58913	-0.68845	H	3.94327	0.226087	-1.7615
C	4.179654	-1.85277	-1.52059	H	2.936055	-1.22969	2.724597
C	4.624181	-0.44512	-1.22196	H	-0.62176	4.594821	-0.08527
O	2.504332	-2.7867	1.479834	H	-2.1294	4.340458	-1.00674
O	3.436986	-0.47907	2.357736	H	-0.55008	4.04011	-1.78679
O	-1.5931	0.05832	0.419534	H	-1.67615	-0.1631	-1.65626
O	-1.24981	2.628593	-0.41754	H	-5.34753	0.908316	-0.62392
C	-1.11292	3.98017	-0.84836	H	-5.63446	-1.10392	1.03202
H	5.395046	-0.69053	0.764053	H	-4.13829	-3.02899	0.357418
C	-2.32333	-0.31661	-0.78966	H	-1.79704	-2.36135	-0.38564
O	-3.42753	0.509527	-0.98544	H	-3.63882	0.68077	1.891653
C	-4.54492	0.396476	-0.07627	H	-5.28176	1.299117	1.726966
C	-4.9654	-1.06098	0.161376	H	-3.01028	2.417383	0.486401
C	-3.76611	-2.00115	0.425243	H	-6.30315	-0.98249	-1.28812
C	-2.69788	-1.80595	-0.66861	H	-2.50863	-1.19111	1.674677
C	-4.31725	1.208744	1.212912	H	-4.05401	-2.04581	-2.02093
O	-3.87321	2.514579	0.922835	H	6.551568	-0.72404	-1.326
O	-5.62508	-1.60623	-0.99653				
Conformer 12_5							
Atom	X	Y	Z	Atom	X	Y	Z
C	-0.43472	0.086692	0.444761	O	3.569473	-1.18604	2.060956
C	-1.71759	0.342	-0.05675	O	5.092042	1.19684	-0.3263
C	-2.06841	1.663274	-0.37577	O	-6.1824	0.044103	0.479578
C	-1.13619	2.689262	-0.15181	H	-0.13062	-0.92384	0.666287
C	0.1511	2.422999	0.318541	H	-1.42293	3.70738	-0.3913

C	0.505533	1.086121	0.591928	H	-3.67052	0.54267	-2.54185
C	-2.58787	-0.84764	-0.48528	H	-5.18465	1.05631	-1.78366
C	-4.06377	-0.42412	-0.67482	H	-3.29015	2.793334	-1.73053
C	-4.13777	0.806558	-1.58955	H	-4.05198	2.466023	-0.19429
C	-3.41985	2.01363	-0.97152	H	-3.33967	-2.93193	2.304219
C	-2.48274	-2.01841	0.5254	H	-5.29514	-1.41084	2.436815
C	-3.49572	-2.13311	1.584959	H	-4.417	0.726061	1.134028
C	-4.55452	-1.3121	1.645427	H	-1.17981	-1.3698	-1.74934
C	-4.78844	-0.19559	0.6669	H	0.557777	4.856307	-0.83084
O	-1.57843	-2.84834	0.450087	H	1.725275	5.279247	0.450568
O	-2.15128	-1.31687	-1.75881	H	-0.00165	5.083192	0.85265
O	1.793903	0.757483	0.984396	H	2.694525	1.788985	-0.57568
O	1.124721	3.3572	0.511536	H	3.051041	-1.84099	-1.98343
C	0.819358	4.715651	0.225038	H	4.376677	-2.71772	0.085837
H	-4.5595	-1.27091	-1.17085	H	5.449297	-0.85573	1.347333
C	2.771402	0.813831	-0.09037	H	4.099865	1.315757	1.51022
O	2.538648	-0.11878	-1.09772	H	1.57837	-2.07178	0.67223
C	2.916483	-1.51336	-0.94819	H	2.224716	-3.4093	-0.27576
C	4.279404	-1.67978	-0.24719	H	-0.05972	-2.79973	-0.70492
C	4.412544	-0.76954	0.99554	H	5.320521	-0.50944	-1.37744
C	4.131034	0.691884	0.608154	H	2.748216	-0.67029	2.015574
C	1.815374	-2.38974	-0.34935	H	5.960417	1.159475	0.097868
O	0.671767	-2.37754	-1.19273	H	-6.55285	-0.72911	0.030454
O	5.356765	-1.45236	-1.14733				
Conformer 12_6							
Atom	X	Y	Z	Atom	X	Y	Z
C	0.769903	-0.35981	0.199981	O	-3.98933	1.571862	-0.84224
C	2.047953	0.194915	0.38186	O	-3.62398	0.200492	2.56993
C	2.231931	1.569288	0.162533	O	6.192788	0.165336	-1.29281
C	1.140167	2.352098	-0.24735	H	0.602214	-1.41893	0.366885
C	-0.12235	1.795726	-0.42825	H	1.295206	3.412827	-0.41329
C	-0.30675	0.417345	-0.1998	H	4.480242	1.376979	2.101591
C	3.197338	-0.67443	0.92102	H	5.636481	1.785201	0.828517
C	4.567948	-0.07559	0.536981	H	3.446077	3.180608	0.894921
C	4.639309	1.378354	1.019554	H	3.942679	2.559755	-0.65897
C	3.580427	2.249978	0.331238	H	3.516235	-3.45021	-1.27548
C	3.082599	-2.11968	0.397436	H	5.03567	-1.86502	-2.42942
C	3.717582	-2.45797	-0.88242	H	4.231177	0.483309	-1.53614
C	4.547644	-1.59821	-1.49386	H	2.621722	-1.53768	2.535832
C	4.857207	-0.21998	-0.97167	H	-2.1494	4.2185	-1.27954
O	2.479398	-2.94933	1.071044	H	-0.45735	4.12412	-1.84748
O	3.12801	-0.72852	2.34135	H	-0.80867	4.409818	-0.1154
O	-1.54962	-0.12894	-0.4463	H	-1.62748	-0.94064	1.476275
O	-1.23721	2.487719	-0.81153	H	-4.72658	-2.47334	-0.45902

C	-1.14132	3.892681	-1.02281	H	-5.8619	-0.15707	-0.80598
H	5.320018	-0.67168	1.07294	H	-5.05413	1.519397	0.917064
C	-2.31701	-0.59236	0.701613	H	-2.54539	1.434722	1.378989
O	-3.05298	-1.70808	0.311853	H	-3.36935	-0.37585	-2.20694
C	-4.20374	-1.51125	-0.54364	H	-4.68923	-1.47091	-2.63768
C	-5.12661	-0.40152	-0.02664	H	-2.14755	-2.32434	-1.80087
C	-4.36358	0.888945	0.342225	H	-6.15655	-1.69433	1.051536
C	-3.18079	0.548769	1.272332	H	-3.02081	1.61598	-0.89836
C	-3.79088	-1.3671	-2.01837	H	-4.39442	-0.38175	2.449189
O	-2.90778	-2.40602	-2.3948	H	6.789987	-0.40777	-0.79125
O	-5.79814	-0.80615	1.179776				
Conformer 12_7							
Atom	X	Y	Z	Atom	X	Y	Z
C	-0.63892	-0.18579	-0.10808	O	3.601359	-0.5053	2.276258
C	-1.95943	0.210313	-0.38722	O	4.903458	1.667129	-0.41388
C	-2.25413	1.57849	-0.48385	O	-6.08745	0.245541	1.315359
C	-1.2348	2.519515	-0.26345	H	-0.37183	-1.23742	-0.06246
C	0.064561	2.127392	0.046061	H	-1.48026	3.573814	-0.33199
C	0.361097	0.748663	0.107307	H	-4.47478	0.766194	-2.30194
C	-3.03279	-0.84466	-0.70235	H	-5.6611	1.371411	-1.13952
C	-4.4478	-0.28067	-0.44032	H	-3.57694	2.870022	-1.5736
C	-4.63463	1.009274	-1.24769	H	-4.0405	2.609739	0.08869
C	-3.64813	2.096292	-0.80016	H	-3.11405	-3.03356	2.094569
C	-2.82093	-2.12239	0.133576	H	-4.74307	-1.3363	2.882863
C	-3.39955	-2.18281	1.482806	H	-4.16256	0.783167	1.433083
C	-4.28985	-1.26746	1.895668	H	-2.42971	-2.03994	-2.0746
C	-4.72191	-0.08701	1.066048	H	1.819813	4.850775	0.451925
O	-2.20526	-3.06084	-0.35906	H	0.117882	4.689172	0.965639
O	-2.94996	-1.21521	-2.07313	H	0.534816	4.677556	-0.77379
O	1.632273	0.331532	0.464714	H	2.436134	1.403994	-1.13487
O	1.104004	2.974328	0.295094	H	4.043014	-2.19673	-1.4991
C	0.865213	4.374568	0.227122	H	5.038802	-2.16268	0.923914
H	-5.15114	-1.04194	-0.80651	H	5.41954	0.217236	1.690041
C	2.72537	0.614085	-0.44197	H	3.551182	1.84791	1.09047
O	3.013085	-0.50223	-1.24813	H	2.191242	-2.14717	0.922391
C	3.635958	-1.65623	-0.63379	H	3.034858	-3.53667	0.229017
C	4.797793	-1.2904	0.300563	H	1.359592	-1.9753	-1.37699
C	4.476001	-0.10157	1.234074	H	6.137926	-1.54856	-1.12677
C	3.919526	1.075026	0.409804	H	2.698106	-0.26356	2.017828
C	2.572731	-2.57227	-0.01117	H	5.428852	0.93983	-0.78957
O	1.53442	-2.8233	-0.94175	H	-6.6294	-0.48333	0.981155
O	5.95549	-0.88312	-0.4502				
Conformer 12_8							
Atom	X	Y	Z	Atom	X	Y	Z

C	0.797034	-0.01149	-0.23318	O	-5.98259	1.192136	-0.16743
C	2.093029	0.142318	0.287864	O	-3.43651	2.571833	-0.10852
C	2.556048	1.431865	0.593169	O	6.406279	-0.32817	-0.77781
C	1.72057	2.538108	0.363711	H	0.397823	-0.9923	-0.46769
C	0.437919	2.382992	-0.15557	H	2.091974	3.525988	0.613664
C	-0.01682	1.085089	-0.4613	H	4.319837	0.113971	2.596263
C	2.940011	-1.09588	0.627142	H	5.752594	0.623298	1.694916
C	4.444034	-0.74554	0.644394	H	3.875652	2.396001	1.98558
C	4.680832	0.421471	1.610742	H	4.54205	2.202869	0.384032
C	3.946694	1.688335	1.151239	H	3.166895	-3.02477	-2.34827
C	2.673209	-2.23584	-0.37538	H	5.137659	-1.55623	-2.68529
C	3.465428	-2.28527	-1.61103	H	4.618955	0.504916	-1.12432
C	4.53728	-1.49505	-1.77949	H	1.925774	-2.27068	1.75499
C	4.986187	-0.46822	-0.77325	H	0.843282	5.018782	-0.68236
O	1.824519	-3.07918	-0.10497	H	0.198708	4.844589	0.977652
O	2.586911	-1.57414	1.920031	H	-0.87269	5.372785	-0.3477
O	-1.27191	0.961215	-1.02212	H	-2.02861	0.542238	0.852998
O	-0.44547	3.401717	-0.39105	H	-3.29864	-1.48264	1.30029
C	-0.03145	4.729333	-0.08823	H	-5.01774	-0.99893	-1.18664
H	4.960027	-1.63903	1.023452	H	-4.5768	0.725514	1.290542
C	-2.2925	0.427871	-0.21129	H	-3.72992	1.152412	-1.61346
O	-2.44978	-0.94105	-0.53223	H	-2.46363	-3.43729	-0.04635
C	-3.49357	-1.57352	0.21965	H	-3.77696	-3.16955	-1.20364
C	-4.82736	-0.88208	-0.10791	H	-5.16843	-3.38295	0.747322
C	-4.74124	0.606736	0.206945	H	-6.65789	-0.95618	0.520263
C	-3.55996	1.223988	-0.52802	H	-5.89741	2.144553	-0.01973
C	-3.48632	-3.0613	-0.14523	H	-2.58634	2.899846	-0.44758
O	-4.30419	-3.82316	0.719463	H	6.781124	-1.15562	-0.44389
O	-5.86621	-1.49933	0.647756				

11. UHPLC-MS data for sugar identification

Figure S185. UHPLC-MS of D-Glc-NAIM (4.92 min) and L-Rha-NAIM (7.16 min)

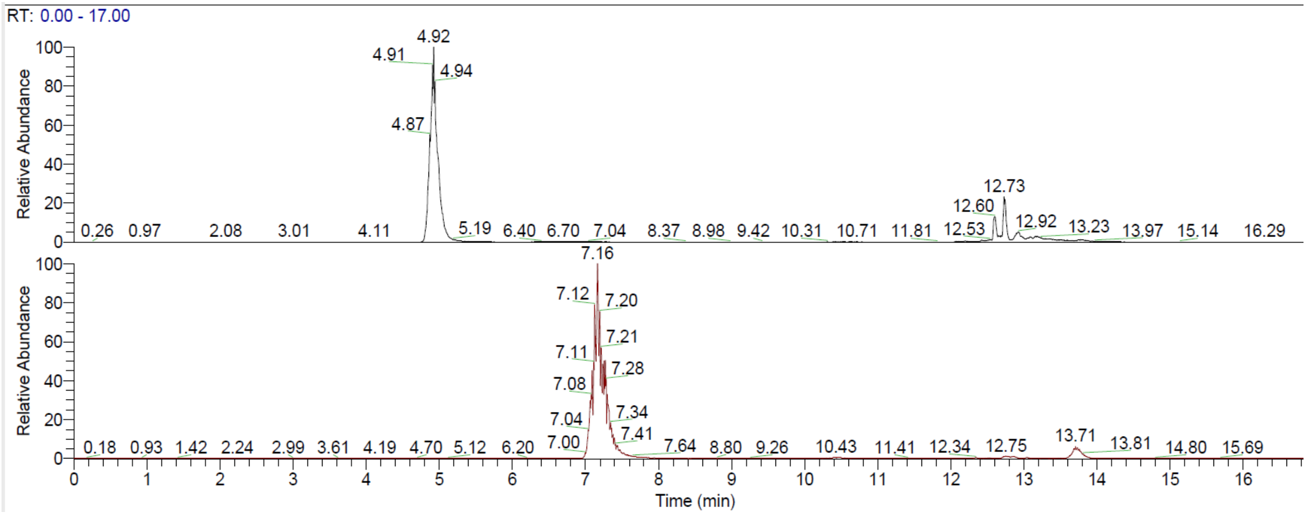

Figure S186. UHPLC-MS of compound **1**'s sugar - NAIM

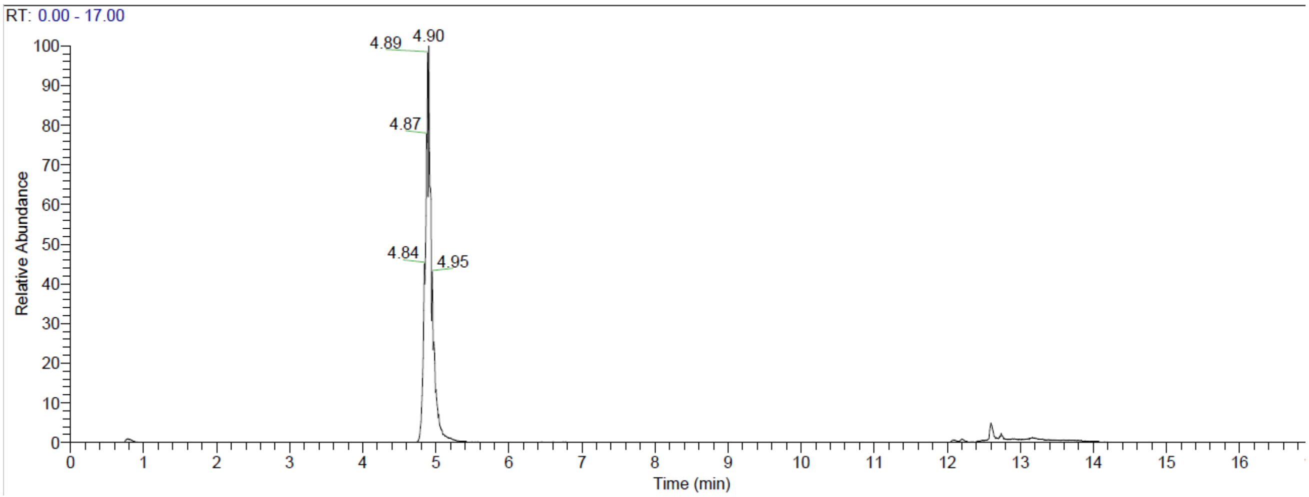

Figure S187. UHPLC-MS of compound **2**'s sugar - NAIM

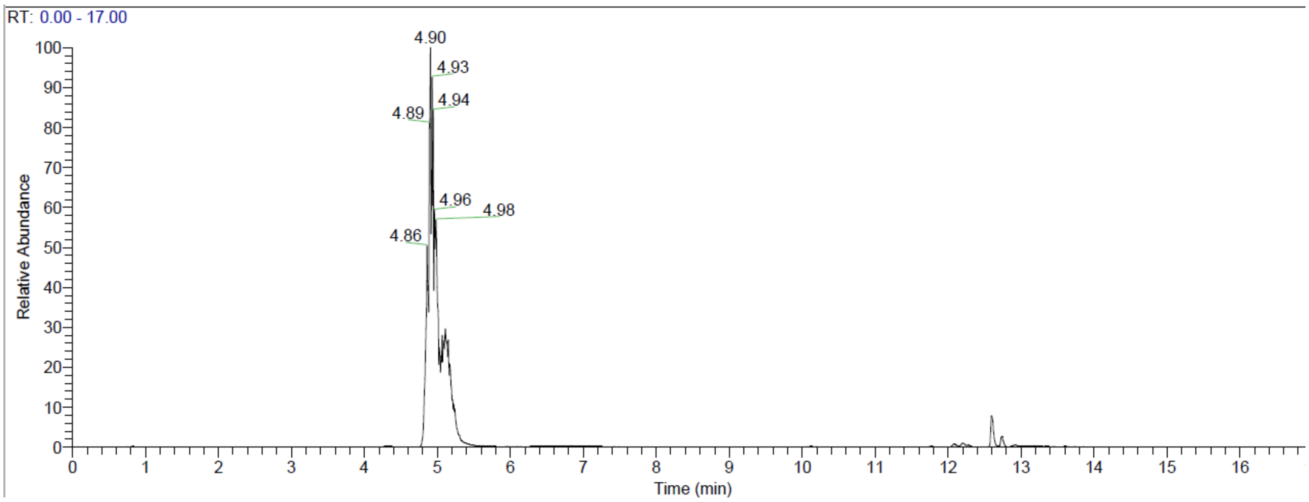

Figure S188. UHPLC-MS of compound **3**’s sugar - NAIM

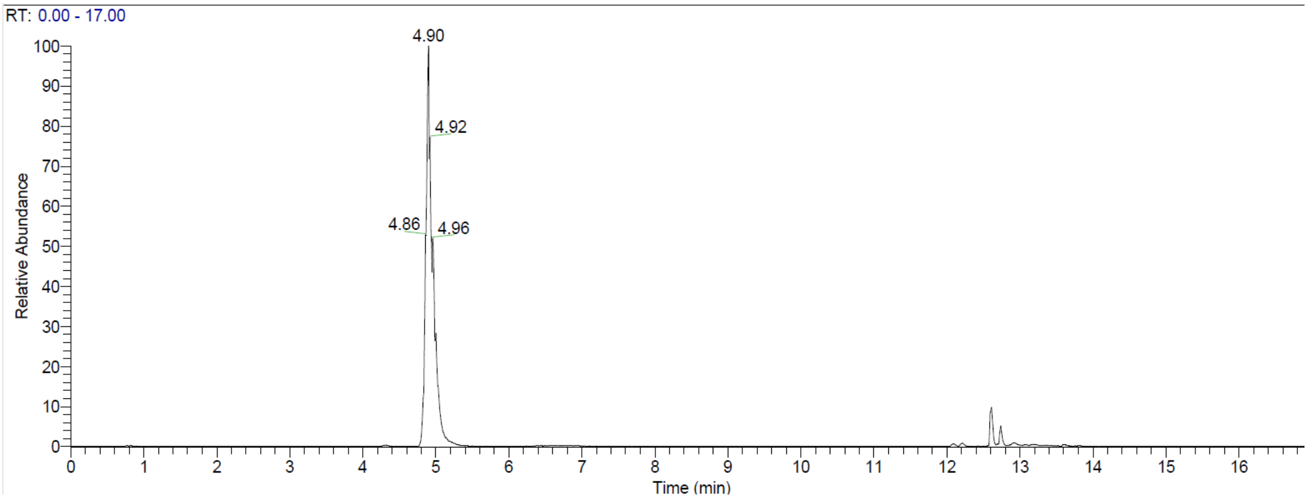

Figure S189. UHPLC-MS of compound **4**’s sugar - NAIM

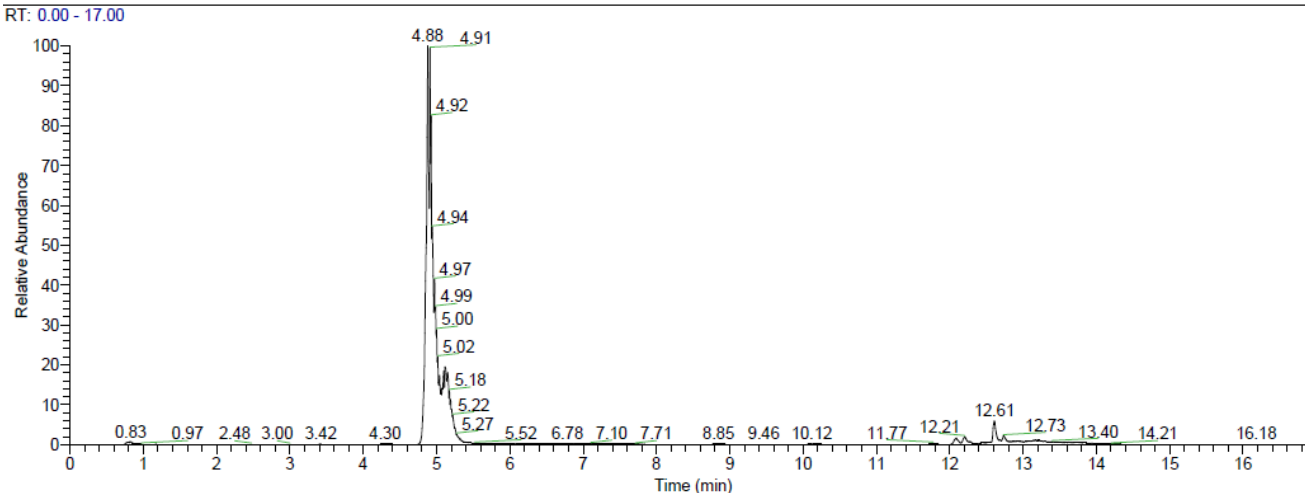

Figure S190. UHPLC-MS of compound **5**’s sugar - NAIM

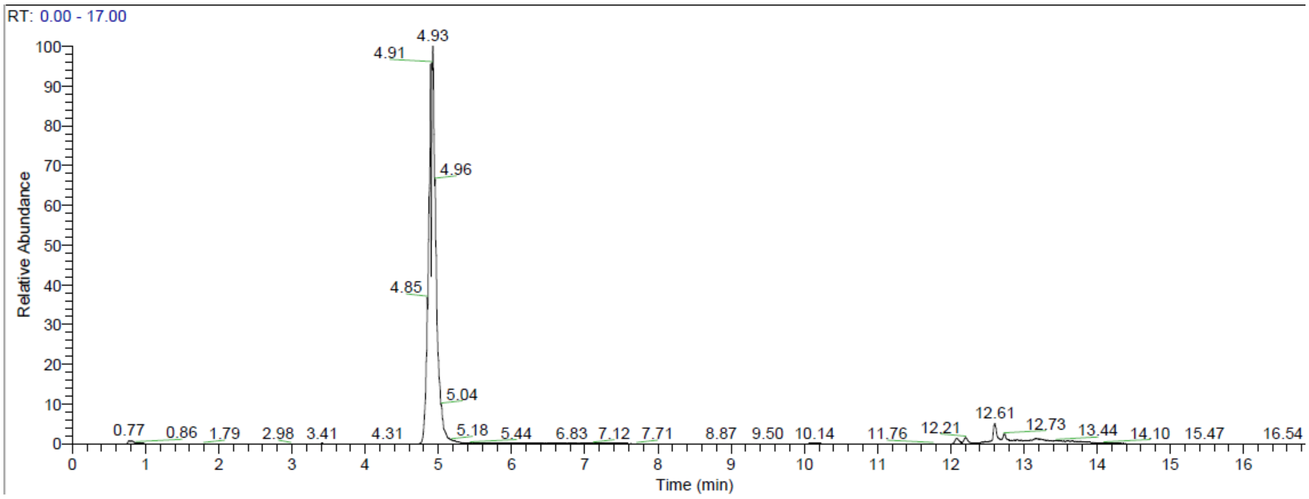

Figure S191. UHPLC-MS of compound **6**'s sugar - NAIM

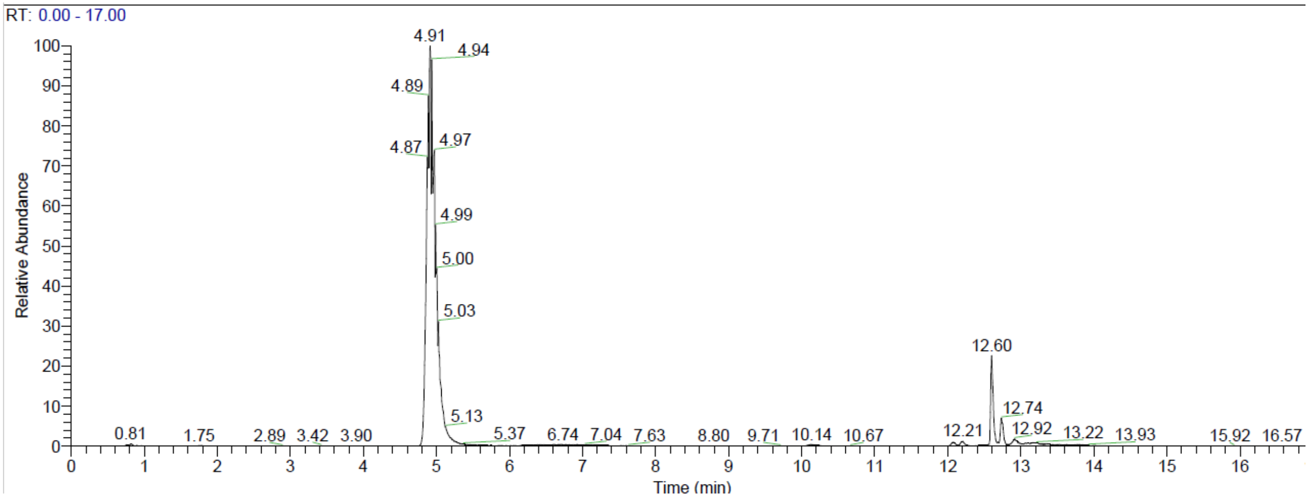

Figure S192. UHPLC-MS of compound **7**'s sugar - NAIM

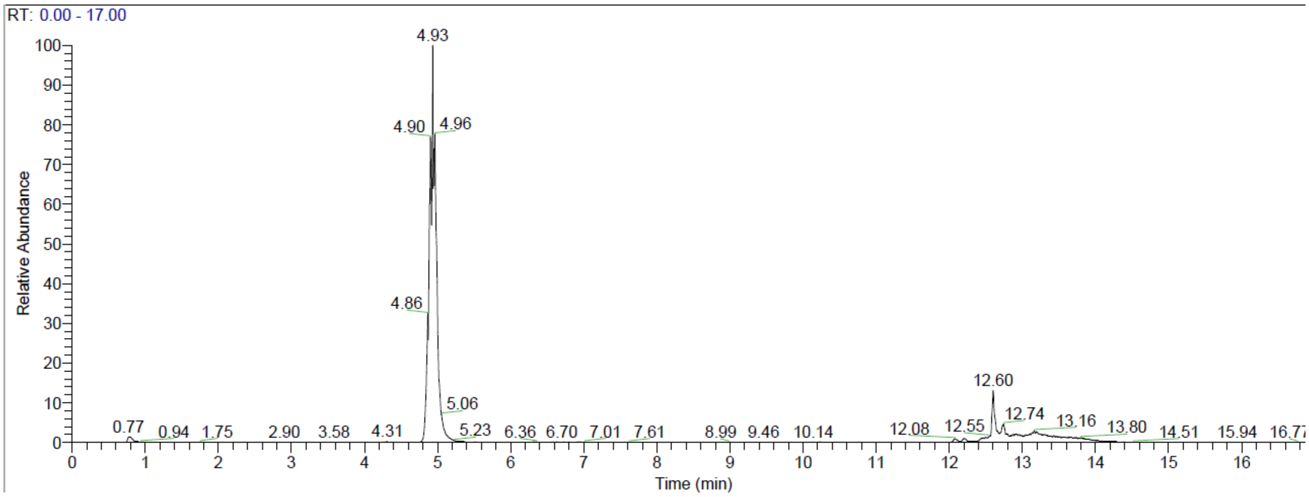

Figure S193. UHPLC-MS of compound **8**'s sugar - NAIM

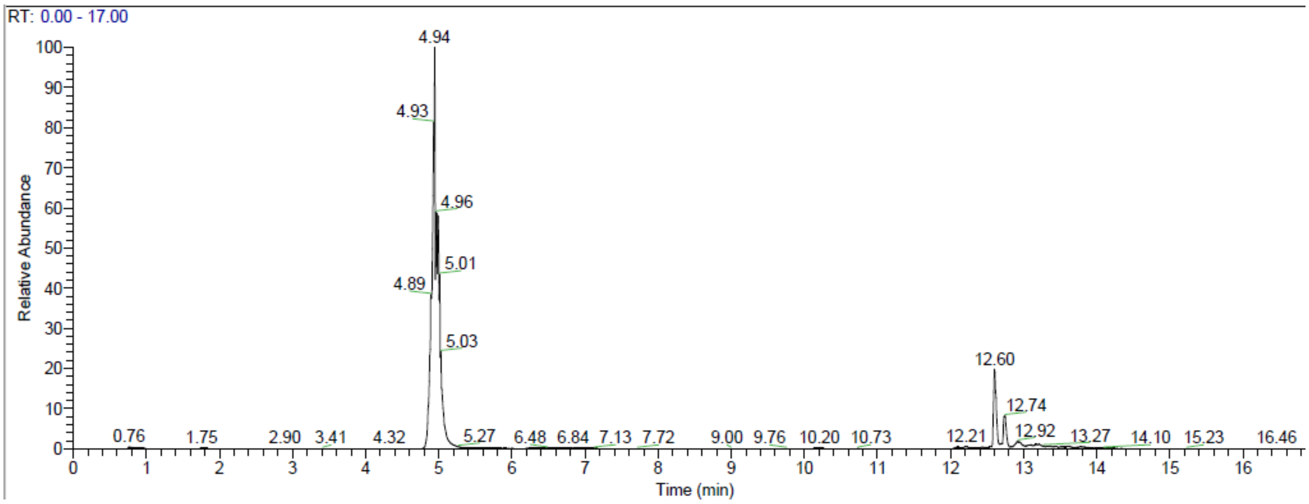

Figure S194. UHPLC-MS of compound **9**'s sugar - NAIM

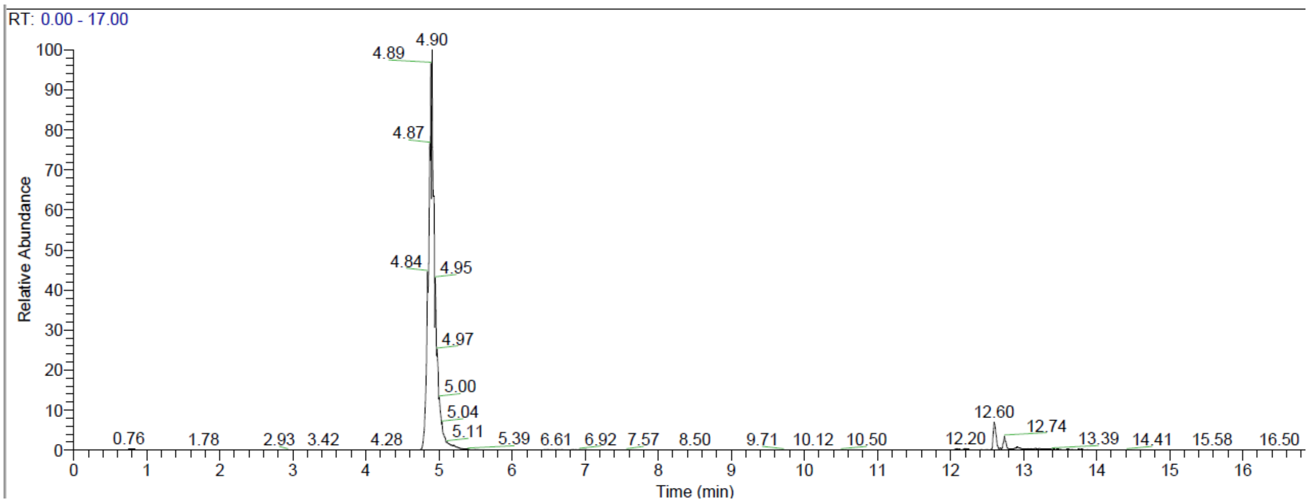

Figure S195. UHPLC-MS of compound **10**'s sugar - NAIM

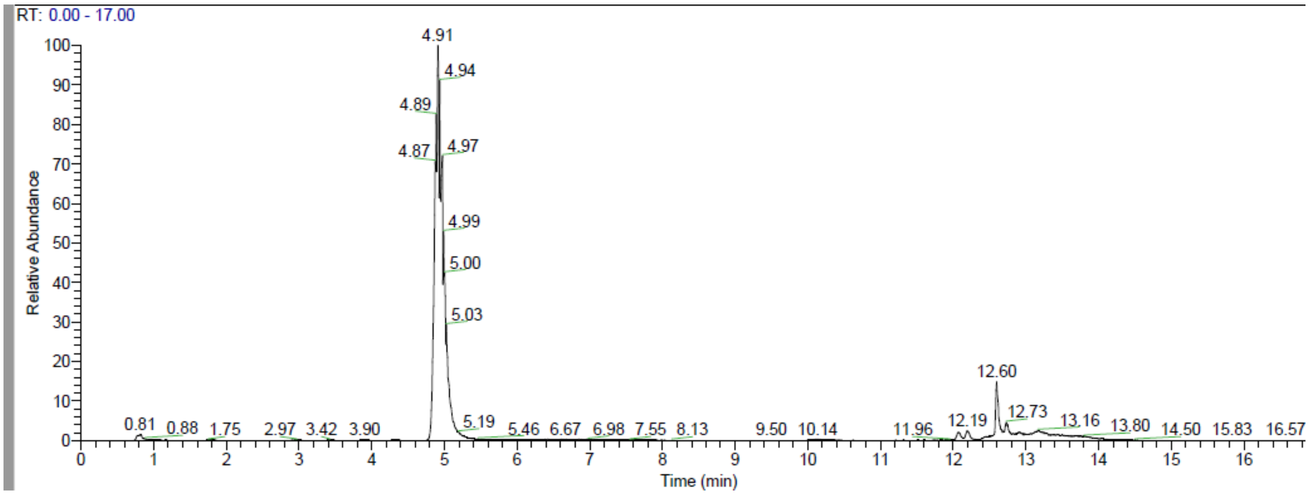

Figure S196. UHPLC-MS of compound **11**'s sugar – NAIM

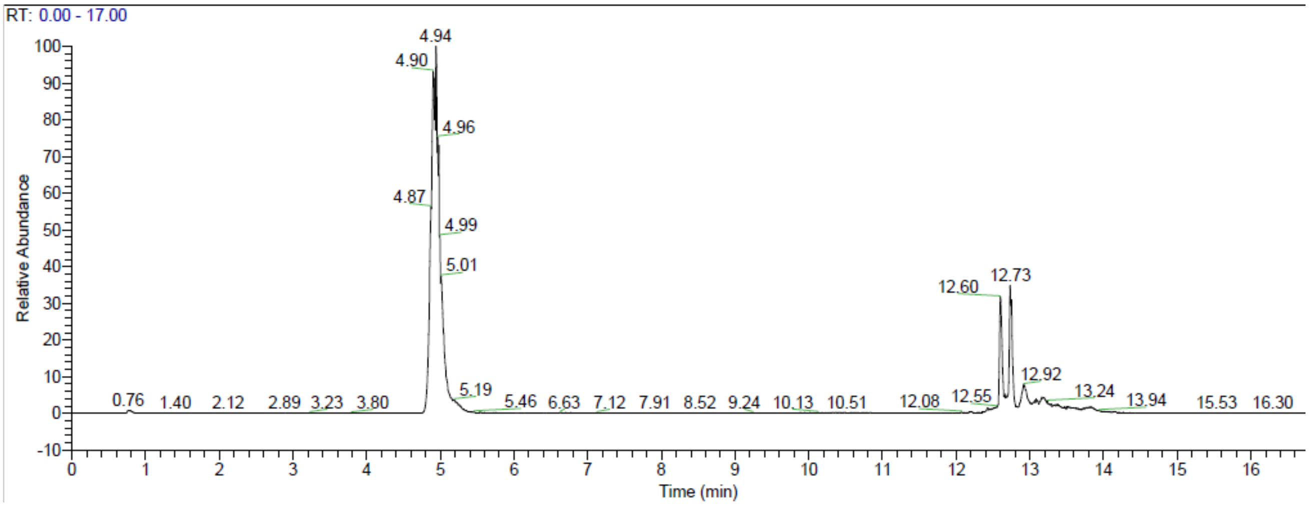

Figure S197. UHPLC-MS of compound **12**’s sugar – NAIM

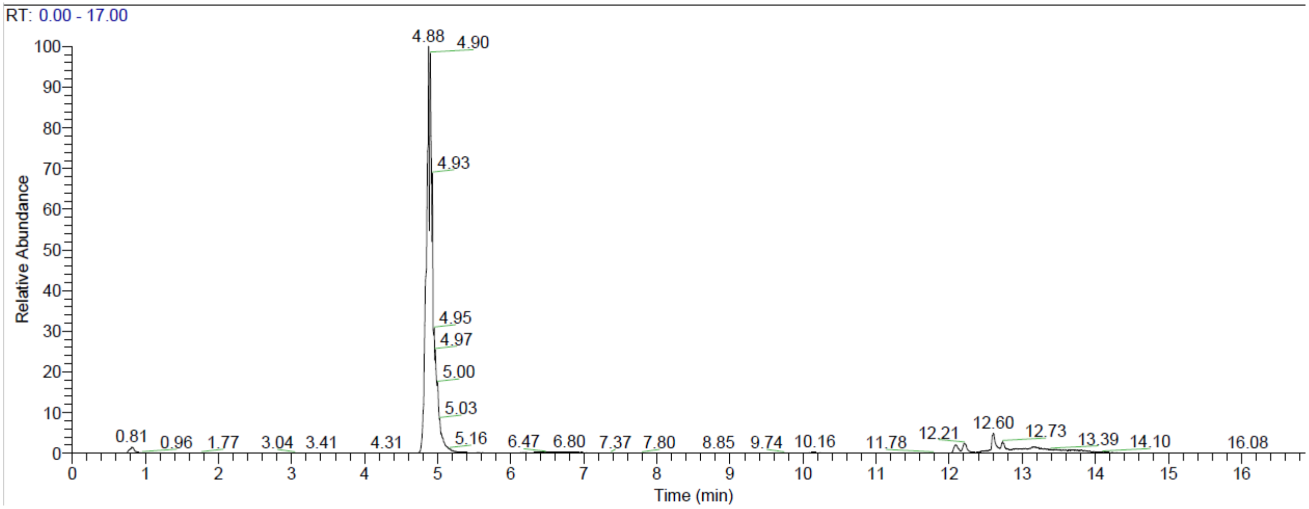

Figure S198. UHPLC-MS of compound **13**’s sugar – NAIM

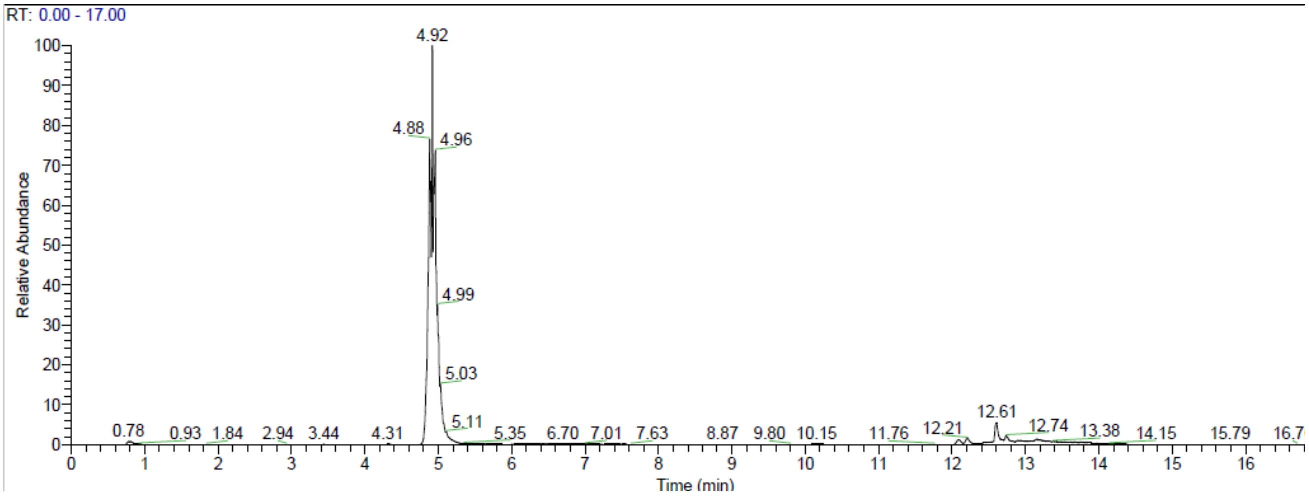

Figure S199. UHPLC-MS of compound **14**’s sugar – NAIM

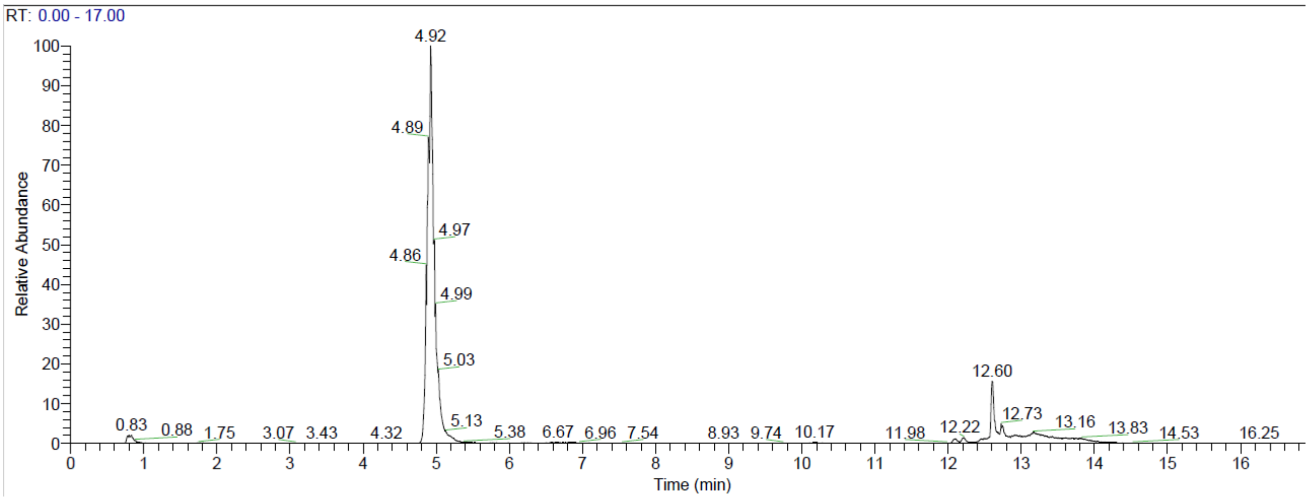

Figure S200. UHPLC-MS of compound **15**'s sugar – NAIM

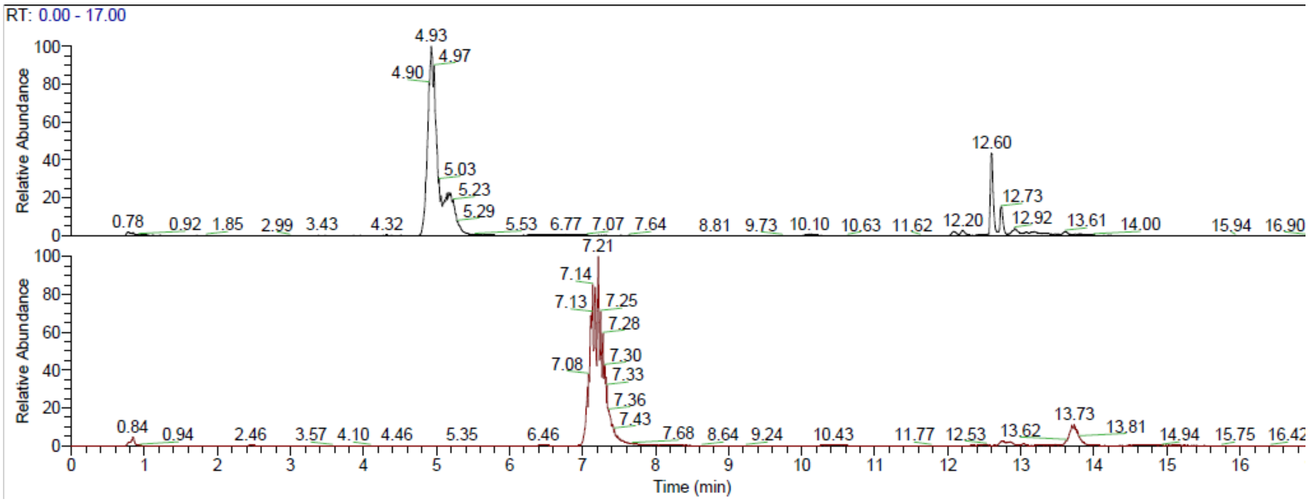

Figure S201. UHPLC-MS of compound **16**'s sugar – NAIM

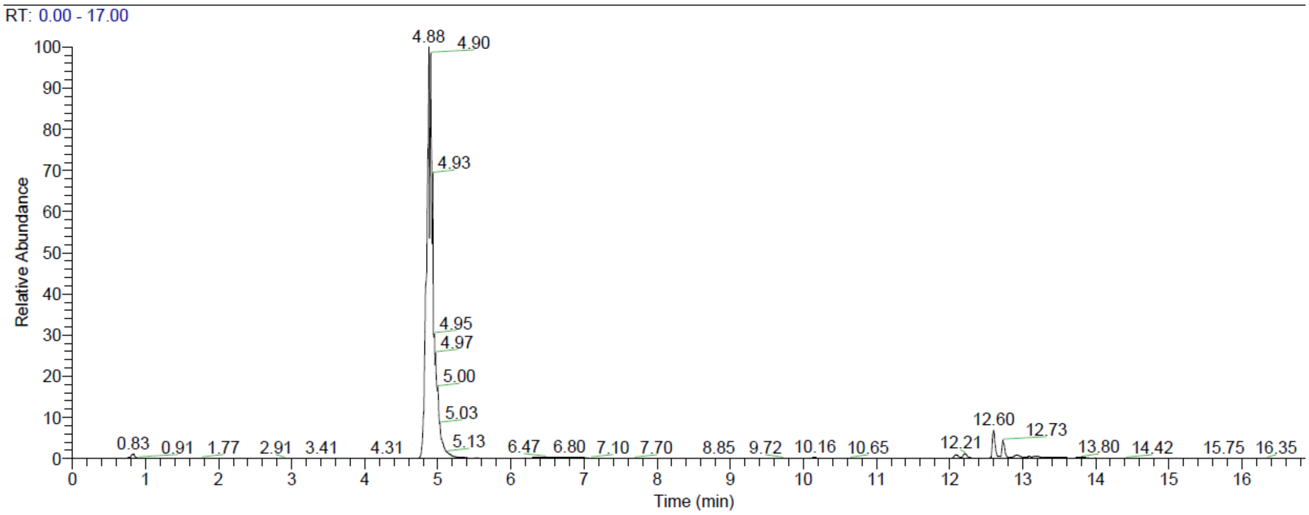

Figure S202. UHPLC-MS of compound **17**'s sugar – NAIM

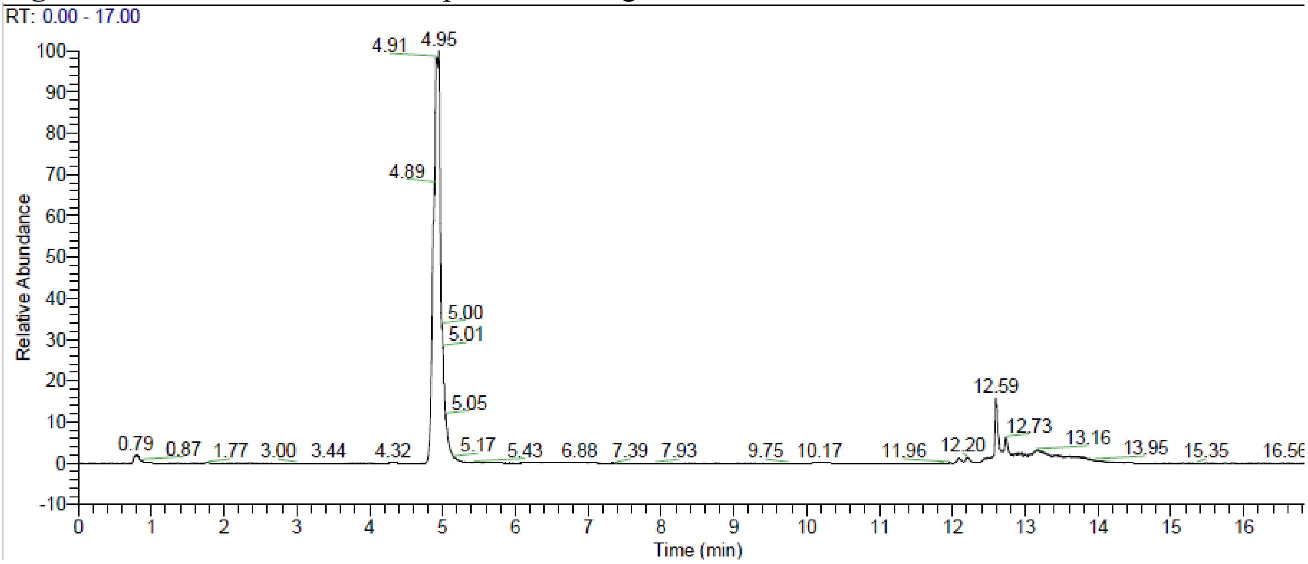

12. DP4+ analysis data of compound 8

Figure S203. The energies and equilibrium populations of the stable conformers of the (3*R*)-**8** and (3*S*)-**8** configurations

No	Conformer	Structure	Gibb free energy (Hartree)	Population (%)
1	(3*R*)-**8**-1	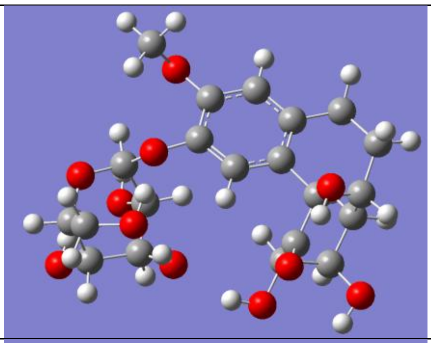	-1644.615856	0.177533
2	(3*R*)-**8**-2	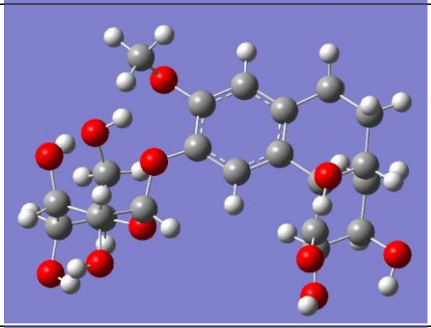	-1644.621712	87.7466
3	(3*R*)-**8**-3	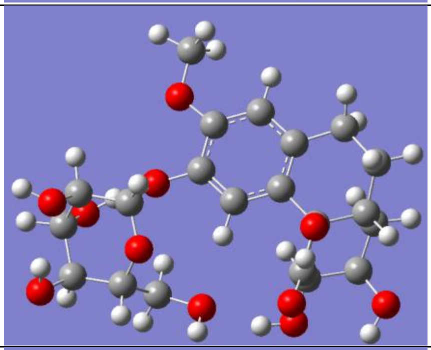	-1644.619840	12.0759
4	(3*S*)-**8**-1	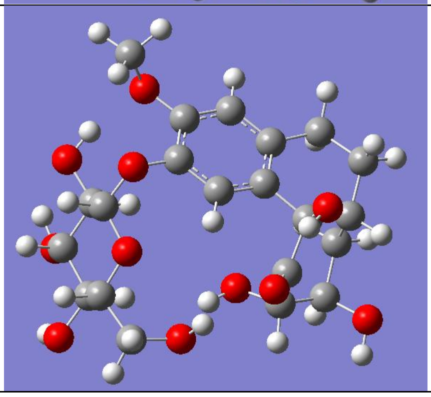	-1644.618174	2.33451

No	Conformer	Structure	Gibb free energy (Hartree)	Population (%)
5	(3*S*)-**8**-2	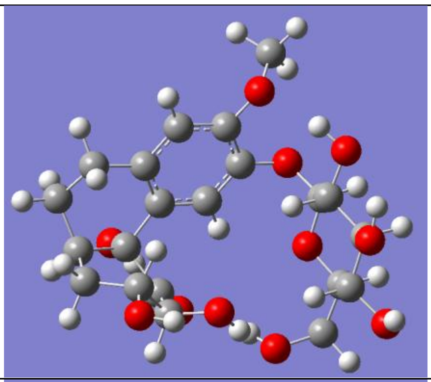	-1644.617248	0.875362
6	(3*S*)-**8**-3	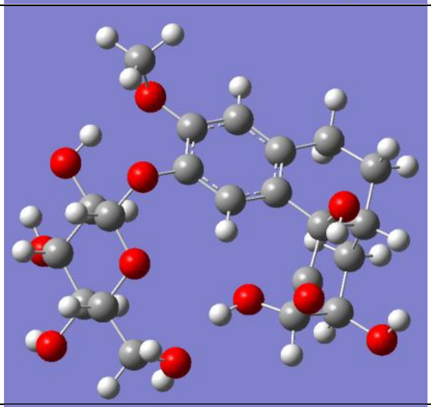	-1644.618044	2.03374
7	(3*S*)-**8**-4	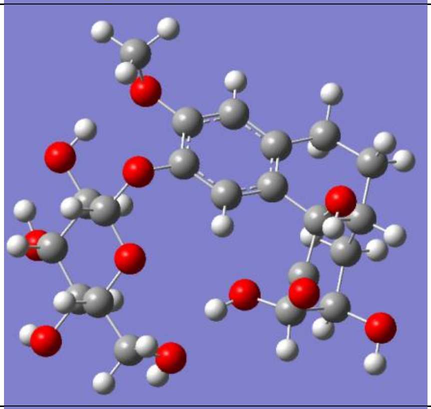	-1644.620702	33.9578
8	(3*S*)-**8**-5	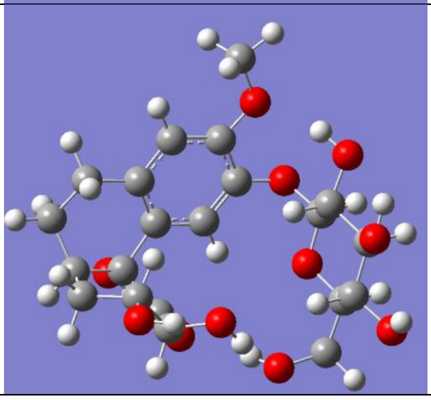	-1644.621252	60.7986

Figure S204. Cartesian coordinates of low energy conformers (3*R*)-**8**-1 to (3*R*)-**8**-3 and (3*S*)-**8**-1 to (3S)-**8**-5

Conformer (3*R*)-**8**-1							
C	0.46403	0.8096	0.86468	O	-1.17685	-1.91516	0.31206
C	1.77589	1.07071	0.50627	O	-2.81988	-0.318	-2.51403
C	2.06484	2.20182	-0.23719	O	1.26893	-3.06738	-0.45604
C	1.01572	3.03308	-0.6233	H	0.2227	-0.06912	1.42026
C	-0.29216	2.73562	-0.31019	H	1.20653	3.91253	-1.20493
C	-0.56377	1.59869	0.43134	H	5.47444	1.67649	-0.57083
C	2.84366	0.07807	0.93883	H	4.71252	1.90802	0.99419
C	4.07376	0.13213	0.02037	H	3.71145	3.5489	-0.43218
C	4.53943	1.59551	-0.02751	H	3.53831	2.43032	-1.75998
C	3.48683	2.51672	-0.67911	H	1.1904	-1.04581	-0.96387
C	2.2207	-1.30406	0.88239	H	2.96534	-2.27356	-2.24729
C	1.84257	-1.76847	-0.49934	H	3.13156	0.16713	-1.94253
C	3.15519	-1.85536	-1.26685	H	4.72935	-0.55578	-1.90515
C	3.79248	-0.47074	-1.36819	H	2.98363	-0.39599	2.83909
O	2.0532	-1.94783	1.89474	H	3.56994	-3.49771	-0.25607
O	3.25078	0.35468	2.28231	H	-2.44367	3.73145	0.98105
O	4.04489	-2.68968	-0.50626	H	-1.28875	5.03907	0.67603
O	-1.87158	1.1956	0.71472	H	-2.75434	4.90561	-0.30911
O	-1.3264	3.5297	-0.77772	H	-2.86091	1.7084	-1.00611
C	-1.99598	4.35712	0.22447	H	-5.28916	-1.01603	0.48389
H	4.82898	-0.47514	0.4978	H	-3.59658	-2.91849	0.25769
C	-2.71667	0.81928	-0.42151	H	-1.91259	-2.35138	-1.53141
O	-3.9687	0.44535	0.06824	H	-1.02612	-0.00986	-1.47564
C	-4.22225	-0.89715	0.58343	H	-4.16254	-1.92334	2.46023
C	-3.58842	-1.98817	-0.28992	H	-4.4046	-0.18512	2.58554
C	-2.14431	-1.68261	-0.71936	H	-2.17787	0.07852	2.06578
C	-2.05559	-0.26017	-1.28301	H	-4.16588	-1.5079	-2.11605
C	-3.87058	-0.96506	2.06072	H	-1.53657	-1.66188	1.19575
O	-2.45008	-0.83	2.29673	H	-2.64835	0.43051	-3.09975
O	-4.39948	-2.18168	-1.45643	H	0.38556	-2.99506	-0.04717
Conformer (3*R*)-**8**-2							
C	0.65292	0.46621	0.80267	O	-4.7217	0.83893	0.48555
C	1.88141	1.0197	0.44988	O	-3.48738	-2.20744	2.12621
C	1.92381	2.25546	-0.17273	O	3.05496	-3.16257	-0.64304
C	0.72225	2.88397	-0.48409	H	0.63606	-0.46867	1.32207
C	-0.48392	2.31367	-0.17139	H	0.71756	3.83132	-0.98403
C	-0.52566	1.09739	0.49537	H	5.36092	2.67353	-0.2644
C	3.15908	0.30592	0.88632	H	4.4394	2.57615	1.22806
C	4.38665	0.7787	0.09162	H	3.16057	3.99051	-0.24222
C	4.44158	2.30788	0.17959	H	3.37133	2.91689	-1.60078
C	3.23317	2.94604	-0.52455	H	2.09983	-1.39239	-1.23224
C	2.98099	-1.1856	0.7029	H	4.28424	-1.64101	-2.39358

C	2.97951	-1.73184	-0.70141	H	3.52343	0.67078	-1.90419
C	4.26015	-1.2613	-1.38013	H	5.27369	0.55551	-1.84969
C	4.35873	0.25736	-1.35295	H	3.19084	-0.22787	2.78814
O	2.83427	-1.9222	1.66021	H	5.20367	-2.72916	-0.48192
O	3.39627	0.56968	2.27481	H	-3.15657	2.65907	0.87761
O	5.35119	-1.77918	-0.60374	H	-1.94742	3.89987	1.2854
O	-1.78364	0.63528	0.82173	H	-3.14732	4.21403	0.02116
O	-1.6619	2.93597	-0.55683	H	-1.33553	-1.21393	1.64161
C	-2.54633	3.45949	0.50108	H	-2.93404	-2.20042	-1.81145
H	5.24736	0.35062	0.58288	H	-5.1738	-1.09657	-1.28546
C	-2.05999	-0.75654	0.99317	H	-5.49163	-0.91073	1.18782
O	-1.98015	-1.44969	-0.22326	H	-3.43562	-0.12988	2.4811
C	-3.03881	-1.30811	-1.21401	H	-3.32393	-0.3381	-3.07874
C	-4.42742	-1.39934	-0.56749	H	-1.75355	-0.04956	-2.34721
C	-4.58695	-0.55549	0.71313	H	-2.6465	1.78064	-1.41327
C	-3.42056	-0.83078	1.66168	H	-4.15046	-3.01057	0.53004
C	-2.81459	-0.12246	-2.15016	H	-4.29745	1.07798	-0.37006
O	-3.3334	1.12949	-1.66163	H	-4.15762	-2.31361	2.81412
O	-4.68758	-2.77685	-0.24455	H	2.71102	-3.46956	0.20994
Conformer (3*R*)-**8**-3							
C	-0.31909	-0.57919	0.31909	O	3.74721	0.23058	-1.85557
C	-1.64519	-0.98487	0.41527	O	4.89592	-0.33647	1.62369
C	-1.977	-2.27484	0.05925	O	-2.12036	2.99084	-1.25359
C	-0.9753	-3.13305	-0.39804	H	-0.03123	0.41863	0.5579
C	0.33554	-2.72486	-0.50645	H	-1.24557	-4.13334	-0.66719
C	0.64977	-1.41467	-0.1511	H	-5.38608	-2.07495	0.65915
C	-2.66708	-0.01141	0.98152	H	-4.19083	-1.91914	1.93745
C	-4.09172	-0.34482	0.51641	H	-3.43666	-3.74716	0.58156
C	-4.35492	-1.81502	0.8729	H	-3.76823	-2.9004	-0.90812
C	-3.41524	-2.77013	0.11049	H	-1.78746	0.93847	-1.50069
C	-2.28653	1.38149	0.525	H	-4.03964	1.64206	-2.33822
C	-2.41276	1.63016	-0.95466	H	-3.72418	-0.6943	-1.59952
C	-3.88157	1.41009	-1.29267	H	-5.34632	-0.14392	-1.21425
C	-4.29738	-0.02247	-0.97237	H	-2.2761	0.78764	2.7346
O	-1.89766	2.20757	1.32617	H	-4.23584	3.16758	-0.48544
O	-2.63463	-0.05601	2.41354	H	0.73895	-5.47094	-0.6176
O	-4.64146	2.28735	-0.44386	H	0.42971	-4.8503	-2.24373
O	1.96175	-0.97166	-0.3107	H	2.08154	-5.21386	-1.73591
O	1.36627	-3.50453	-0.94789	H	2.4562	-1.01224	1.70073
C	1.12289	-4.84387	-1.41217	H	2.74637	2.8414	1.05059
H	-4.75242	0.29126	1.08712	H	4.37219	2.57271	-0.94274
C	2.67278	-0.43267	0.82143	H	5.54513	0.40573	-0.87237
O	2.28645	0.88714	1.1217	H	4.27162	-1.57314	0.04662
C	2.74991	2.02729	0.34038	H	1.75029	1.56029	-1.51048
C	4.19844	1.87677	-0.13743	H	2.12647	3.2573	-1.27717
C	4.48866	0.46538	-0.65263	H	0.38368	2.82034	0.61828

C	4.13022	-0.56927	0.41806	H	5.10328	1.5146	1.57107
C	1.78218	2.35434	-0.79065	H	3.00182	-0.36896	-1.68529
O	0.42715	2.55702	-0.31081	H	5.7674	-0.75116	1.57639
O	5.10686	2.22754	0.91308	H	-1.18621	3.15104	-1.0248
Conformer (3S)-**8**-1							
C	-0.39769	0.88445	-1.07446	O	3.99481	-0.67131	2.37203
C	-1.62815	1.07715	-0.43252	O	3.68693	1.72322	0.88715
C	-1.80026	2.18894	0.41212	O	-0.86331	-1.74152	1.10217
C	-0.71308	3.05079	0.62118	H	-0.24452	0.05239	-1.74825
C	0.52161	2.82411	0.02386	H	-0.81701	3.91024	1.27742
C	0.67886	1.72848	-0.83914	H	-5.16066	1.7663	1.173
C	-2.78989	0.11078	-0.74589	H	-4.57431	2.12125	-0.44965
C	-3.92695	0.21234	0.29985	H	-3.32175	3.55222	1.06023
C	-4.28812	1.68936	0.51538	H	-3.01041	2.2458	2.17688
C	-3.11593	2.47719	1.10856	H	-1.98556	-3.2101	0.14036
C	-2.24001	-1.32918	-0.82192	H	-2.99755	-2.45879	2.3552
C	-2.05728	-2.15903	0.45269	H	-4.41451	-0.42489	2.30817
C	-3.25289	-1.98498	1.3975	H	-2.70529	-0.04806	2.09904
C	-3.57141	-0.50905	1.61411	H	-2.83734	-0.10391	-2.66543
O	-1.8971	-1.77448	-1.91278	H	-4.25951	-3.54695	0.77384
O	-3.33589	0.43377	-2.02606	H	1.15474	5.23905	-0.91522
O	-4.40667	-2.59167	0.81242	H	2.29764	4.03551	-1.59141
O	1.90314	1.52727	-1.46215	H	2.82254	5.1357	-0.28326
O	1.5988	3.63322	0.34535	H	3.65518	0.57362	-1.52889
C	1.98594	4.56468	-0.68742	H	3.75579	-1.97921	-1.46393
H	-4.78517	-0.30003	-0.14489	H	2.14984	-2.00314	1.13857
C	2.71054	0.46957	-0.97809	H	4.77092	-0.6117	0.4459
O	2.07179	-0.75691	-1.30087	H	2.00081	0.50814	1.05221
C	2.79157	-1.93729	-0.93148	H	1.92569	-3.14948	-2.48465
C	3.09325	-1.94417	0.57501	H	2.44211	-4.0437	-1.04146
C	3.79837	-0.64919	0.96421	H	0.0283	-2.73033	-1.52853
C	2.96875	0.55414	0.53307	H	4.20014	-2.98677	1.76588
C	1.95515	-3.12924	-1.39094	H	4.34839	0.19891	2.61035
O	0.62349	-3.11159	-0.86378	H	3.07242	2.47727	0.80355
O	3.90951	-3.07527	0.84611	H	-0.12895	-2.1163	0.57419
Conformer (3S)-**8**-2							
C	-0.60844	0.82905	-1.19117	O	3.7905	-0.23258	2.41081
C	-1.87771	0.76892	-0.59987	O	3.14959	2.17909	1.05642
C	-2.27632	1.77789	0.29364	O	-0.27184	-2.17489	0.94631
C	-1.36901	2.80425	0.59557	H	-0.28455	0.07492	-1.89545
C	-0.08761	2.82567	0.05321	H	-1.64842	3.59466	1.28613
C	0.29597	1.82457	-0.85414	H	-5.58309	0.8171	0.68156
C	-2.79152	-0.41887	-0.98381	H	-4.84764	1.36154	-0.82447
C	-4.07407	-0.51845	-0.10741	H	-4.02845	2.79196	0.99767
C	-4.63281	0.89231	0.14274	H	-3.57253	1.40677	1.96228
C	-3.65102	1.76625	0.92667	H	-2.00265	-3.26666	0.53041

C	-1.89342	-1.65097	-0.82134	H	-1.97506	-0.52386	1.82394
C	-1.63796	-2.22583	0.5751	H	-4.46352	-0.89068	2.01709
C	-2.441	-1.50438	1.65409	H	-4.30076	-2.35016	1.06084
C	-3.89	-1.34376	1.20253	H	-2.55574	-0.86767	-2.84281
O	-1.36503	-2.13657	-1.81692	H	-1.49164	-2.42418	3.06345
O	-3.17831	-0.30597	-2.34704	H	0.20159	5.35547	-0.75133
O	-2.42491	-2.26081	2.86026	H	1.56577	4.38306	-1.38889
O	1.56568	1.85595	-1.41156	H	1.81987	5.48783	-0.00603
O	0.81746	3.78191	0.47736	H	3.45008	1.20062	-1.42168
C	1.11478	4.81189	-0.49072	H	4.01372	-1.21596	-1.50956
H	-4.78772	-1.05385	-0.73908	H	2.29321	-1.76809	0.95621
C	2.50764	0.90809	-0.93913	H	4.6539	0.11666	0.55408
O	2.10712	-0.38606	-1.37049	H	1.69777	0.6801	1.04031
C	3.03632	-1.42883	-1.04578	H	2.49951	-2.61363	-2.755
C	3.24423	-1.50244	0.47423	H	3.24072	-3.5166	-1.4202
C	3.67593	-0.1359	0.99662	H	0.54538	-2.74428	-1.72178
C	2.66751	0.9315	0.58781	H	4.45761	-2.40744	1.67318
C	2.52531	-2.72753	-1.66591	H	3.9806	0.66218	2.7303
O	1.24698	-3.15268	-1.18726	H	2.42352	2.82571	0.97163
O	4.23775	-2.48586	0.73305	H	0.27795	-2.66821	0.30206
Conformer (3S)-**8**-3							
C	0.48974	0.86389	1.05458	O	-4.39578	-0.47312	-2.16066
C	1.69341	1.07168	0.36844	O	-3.82698	1.83444	-0.61489
C	1.80049	2.1419	-0.53616	O	0.96888	-1.74067	-0.8528
C	0.68848	2.97557	-0.72897	H	0.38772	0.05388	1.76687
C	-0.51058	2.75223	-0.06333	H	0.74772	3.81541	-1.41543
C	-0.61664	1.67141	0.8273	H	5.12633	1.74389	-1.46103
C	2.90534	0.19751	0.71913	H	4.62983	2.2052	0.16561
C	3.9928	0.23323	-0.39448	H	3.25117	3.49204	-1.35853
C	4.29798	1.69756	-0.74602	H	2.91171	2.10359	-2.36233
C	3.0704	2.41217	-1.3203	H	2.09969	-3.19664	0.13351
C	2.51016	-1.27618	0.96264	H	2.95892	-2.60498	-2.16777
C	2.17145	-2.16642	-0.23461	H	4.41709	-0.57891	-2.35819
C	3.30785	-2.07607	-1.27527	H	2.71405	-0.20043	-2.10543
C	3.60538	-0.61353	-1.62332	H	3.29575	-0.00179	2.59161
O	2.54792	-1.71547	2.10382	H	4.90982	-2.28716	-0.14663
O	3.47213	0.69446	1.93053	H	-2.73756	5.11391	0.36252
O	4.45816	-2.79616	-0.8336	H	-1.01924	5.19784	0.84491
O	-1.80451	1.46922	1.51697	H	-2.12214	4.03863	1.65204
O	-1.60179	3.56705	-0.32465	H	-3.54339	0.51836	1.71834
C	-1.88001	4.53605	0.70919	H	-3.72563	-1.93621	1.57437
H	4.89685	-0.19005	0.06336	H	-2.45654	-1.90151	-1.20938
C	-2.67122	0.45176	1.05332	H	-4.9347	-0.47943	-0.15447
O	-2.01898	-0.8008	1.20747	H	-2.21727	0.58335	-1.04527
C	-2.8469	-1.93159	0.90938	H	-1.5931	-3.08521	2.20602
C	-3.33219	-1.85683	-0.54445	H	-2.69974	-4.05316	1.20963

C	-4.03299	-0.52568	-0.78741	H	-1.28191	-3.85958	-0.47644
C	-3.1115	0.63007	-0.40722	H	-4.59099	-2.82029	-1.64924
C	-2.03307	-3.18608	1.21081	H	-4.73472	0.42144	-2.31616
O	-0.94527	-3.39368	0.30135	H	-3.16701	2.55434	-0.61035
O	-4.19609	-2.9608	-0.77584	H	0.2254	-2.13811	-0.35323
Conformer (3S)-**8**-4							
C	0.49589	0.86018	1.05357	O	-4.41473	-0.45862	-2.14703
C	1.69727	1.06871	0.36361	O	-3.83238	1.8393	-0.59398
C	1.79925	2.13902	-0.54196	O	0.97573	-1.74528	-0.87785
C	0.68613	2.97233	-0.72962	H	0.39707	0.05001	1.76611
C	-0.51032	2.74825	-0.05932	H	0.74225	3.81273	-1.41573
C	-0.61233	1.66652	0.8303	H	5.12229	1.74651	-1.47702
C	2.91287	0.19548	0.70905	H	4.62943	2.20925	0.14976
C	3.99721	0.2339	-0.40458	H	3.24265	3.48959	-1.37631
C	4.2959	1.69774	-0.75944	H	2.90303	2.09516	-2.37184
C	3.06558	2.40923	-1.33186	H	2.08647	-3.19173	0.13136
C	2.51181	-1.27512	0.95236	H	3.02501	-2.63293	-2.15941
C	2.17201	-2.16361	-0.24631	H	4.42045	-0.58533	-2.36232
C	3.32594	-2.0726	-1.2632	H	2.71312	-0.21855	-2.11889
C	3.60697	-0.61659	-1.62917	H	3.28973	-0.00223	2.58395
O	2.52595	-1.71114	2.09491	H	4.37583	-3.55489	-0.52933
O	3.47854	0.69073	1.92299	H	-2.73227	5.11231	0.38
O	4.51885	-2.61232	-0.69341	H	-1.01112	5.19338	0.85276
O	-1.79768	1.46167	1.52424	H	-2.11109	4.03525	1.66527
O	-1.60258	3.5642	-0.31455	H	-3.53439	0.50814	1.73051
C	-1.87358	4.5329	0.72132	H	-3.71551	-1.94529	1.57368
H	4.89288	-0.20856	0.04248	H	-2.46773	-1.89254	-1.2193
C	-2.6664	0.44676	1.05951	H	-4.93776	-0.47723	-0.13673
O	-2.01245	-0.80659	1.20091	H	-2.22628	0.58968	-1.04114
C	-2.84198	-1.93587	0.90194	H	-1.57523	-3.09605	2.18001
C	-3.33842	-1.85218	-0.54764	H	-2.6922	-4.05895	1.19026
C	-4.04105	-0.51969	-0.77706	H	-1.29234	-3.85432	-0.51074
C	-3.11633	0.63348	-0.39711	H	-4.60689	-2.8077	-1.64826
C	-2.02527	-3.19203	1.18883	H	-4.75115	0.43821	-2.29492
O	-0.94672	-3.39591	0.26757	H	-3.17113	2.55814	-0.59169
O	-4.20377	-2.95496	-0.77974	H	0.22794	-2.14162	-0.38428
Conformer (3S)-**8**-5							
C	-0.62032	0.64821	-1.31832	O	3.90201	0.18408	2.28573
C	-1.89362	0.60863	-0.73026	O	3.188	2.39927	0.62704
C	-2.33102	1.70643	0.02422	O	-0.17371	-2.03603	1.16543
C	-1.46862	2.80058	0.20418	H	-0.26351	-0.16901	-1.92999
C	-0.17863	2.80553	-0.32728	H	-1.81881	3.6443	0.78756
C	0.24277	1.70783	-1.10669	H	-4.88281	1.05332	-1.03068
C	-2.7587	-0.64918	-0.9659	H	-5.60112	0.67764	0.53419
C	-4.03898	-0.68801	-0.08171	H	-4.12524	2.73635	0.58941
C	-4.65281	0.72038	-0.0118	H	-3.62197	1.50609	1.72557

C	-3.70839	1.7253	0.65198	H	-1.85462	-3.24474	0.89723
C	-1.81314	-1.81249	-0.65082	H	-1.94872	-0.36232	1.83308
C	-1.53582	-2.19201	0.80711	H	-4.42061	-0.80578	2.07365
C	-2.37255	-1.37486	1.788	H	-4.19276	-2.36593	1.30845
C	-3.82534	-1.3331	1.3217	H	-2.49855	-1.31798	-2.75436
O	-1.26249	-2.3994	-1.57822	H	-1.39162	-2.07074	3.30039
O	-3.14618	-0.72549	-2.33226	H	-0.5027	5.46174	0.11807
O	-2.33007	-1.97331	3.07967	H	0.07135	4.69877	1.63303
O	1.50466	1.72741	-1.68432	H	1.20578	5.62158	0.60855
O	0.7397	3.80178	-0.13833	H	3.40344	1.11809	-1.6951
C	0.33964	4.96086	0.60462	H	4.04361	-1.26314	-1.48377
H	-4.72974	-1.3266	-0.63856	H	2.39748	-1.54612	1.07632
C	2.48857	0.86558	-1.14156	H	4.70736	0.31383	0.37491
O	2.11341	-0.48296	-1.39122	H	1.75887	0.89818	0.88184
C	3.08573	-1.44869	-0.96988	H	2.54377	-2.84968	-2.50433
C	3.33158	-1.32608	0.54088	H	3.36312	-3.55642	-1.09881
C	3.74704	0.10342	0.87354	H	0.62975	-2.94063	-1.4017
C	2.70766	1.09234	0.36056	H	4.60169	-2.0345	1.80938
C	2.60786	-2.83032	-1.41099	H	4.12666	1.1042	2.48776
O	1.36396	-3.24088	-0.8389	H	2.47396	3.01996	0.39569
O	4.35225	-2.24864	0.89806	H	0.39618	-2.58913	0.59125

Figure S205. DP4+ probability distribution for the stereoisomers (3*R*)-**8** and (3*S*)-**8**

Functional		Solvent?		Basis Set	
mPW1PW91		PCM		6-311+G(d,p)	
		DP4+	0.00%	100.00%	-
Nuclei	sp2?	Experimental	Isomer 1	Isomer 2	Isomer 3
C		25	160.8	160.3	
C	x	205.8	-41.4	-43.2	
C		53.5	108.1	107.2	
C		55	110.0	113.1	
C		26.8	157.0	156.4	
C	x	117.7	67.4	52.7	
C		56.7	124.6	127.8	
C		103.2	89.4	80.7	
C		78.4	101.6	107.7	
C		71.3	114.3	117.0	
C		77.9	117.5	108.9	
C		74.9	113.56	114.56	
C		62.6	119.74	122.93	
C	x	133.4	48.78	52.82	
C	x	125.3	48.82	43.66	
C	x	115	57.94	65.62	
C	x	151.4	31.75	28.14	
C	x	146.2	33.40	40.06	
C		77	105.35	106.51	
C		32.4	142.72	147.37	
C		21.7	162.83	160.04	
H		2.31	29.24	29.26	
H	x	6.84	24.66	24.55	
H	x	6.85	24.53	24.58	
H		1.66	29.98	29.80	
H		2.37	29.42403333	29.70353255	
H		2.82	28.89786667	28.91239054	
H		2.94	28.7801	28.72799875	
H		3.29	27.36916667	27.96951946	
H		3.43	27.40216667	28.05300801	
H		1.93	30.02823333	29.97216699	
H		2.24	29.89666667	29.79381065	
H		3.84	27.70946667	27.95637548	
H		3.84	28.24643333	28.0445558	
H		3.84	27.79883333	27.66572918	
H		4.72	26.12376667	26.92363176	
H		3.36	27.65496667	28.42768692	
H		3.4	28.03403333	28.31370895	
H		3.43	27.96376667	28.26198777	
H		3.46	27.8888	28.99399246	
H		3.72	27.97326667	27.70954116	
H		3.97	27.93786667	27.67747596	

	Isomer 1	Isomer 2
sDP4+ (H data)	0.00%	100.00%
sDP4+ (C data)	97.71%	2.29%
sDP4+ (all data)	0.00%	100.00%
uDP4+ (H data)	0.00%	100.00%
uDP4+ (C data)	0.18%	99.82%
uDP4+ (all data)	0.00%	100.00%
DP4+ (H data)	0.00%	100.00%
DP4+ (C data)	7.02%	92.98%
DP4+ (all data)	0.00%	100.00%
